# “Store-and-Release” Strategy Enables Nickel-Catalyzed Deaminative Migratory Arylation of Aliphatic Amines

**DOI:** 10.1021/jacs.6c09936

**Published:** 2026-09-08

**Authors:** Abhishek A. Kadam, Kevin Lee, Yumeng Liao, Aleksandr Knyazev, M. Joana Lourenço, Pinku Tung, Anthony N. Cauley, Jesús M. Dones, Martins S. Oderinde, James Kempson, Jose B. Roque

**Affiliations:** † Department of Chemistry, 6740 Princeton University, Princeton, New Jersey 08544, United States; ‡ Global Discovery Chemistry, Bristol Myers Squibb Research & Early Development, Route 206 & Province Line Road, Princeton, New Jersey 08543, United States

## Abstract

While migratory functionalization reactions provide a powerful means to navigate broader chemical space, their application to aliphatic amines remains particularly elusive. Herein, we describe the nickel-catalyzed migratory arylation of aliphatic amines using Katritzky salts enabled by a “store-and-release” strategy. This strategy leverages dihydropyridines, which are traditionally viewed as chemical dead-ends under reductive cross-coupling conditions, as latent reservoirs to store and release translocated alkyl radicals, thereby decoupling the chain-walking event from the cross-coupling cycle. The developed protocol enables migratory functionalization of Katritzky salts derived from a wide range of primary amines, and a variety of aryl and heteroaryl bromides. The reaction exhibits broad functional group tolerance, accommodating alkenes, polar functionalities, and substrates derived from pharmaceutically relevant compounds. This methodology enables expanded small-molecule library synthesis through regiodivergent deaminative arylations. Preliminary mechanistic studies support the “store-and-release” design principle, rendering it as a platform that can be applied for other migratory functionalization reactions.

## Graphical abstract

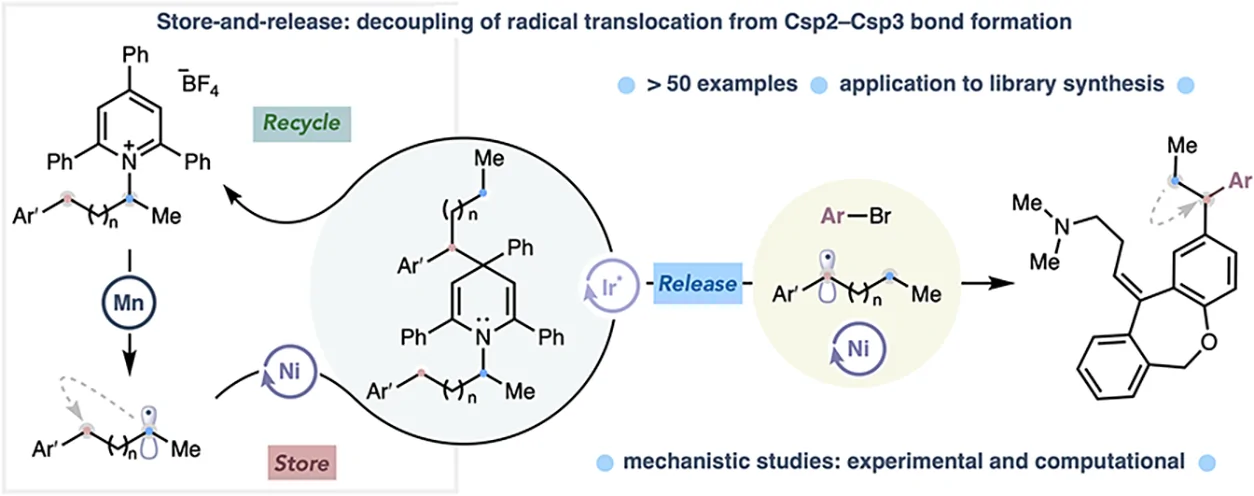

## Introduction

The need in medicinal chemistry to explore vast chemical space by evaluating an expansive set of small molecules has continually driven the development of new synthetic transformations.^1^ Decades of research have established transition metal-catalyzed *ipso*-coupling reactions, including cross- and cross-electrophile couplings, as a cornerstone of modern synthetic chemistry.^2^ These reactions are prized for their reliability, predictability, and ability to forge C–C bonds from a variety of precursors (Figure 1A). Alternatively, redirecting functionalization to sites beyond the *ipso*-position would allow access to greater chemical space from a defined starting point. In this context, migratory functionalization reactions,^3^ in which the bond formation occurs at a position other than the functional group’s native site, offer a complementary approach for accessing broader chemical space without the need for *de novo* synthesis (Figure 1A). Pioneering studies from the laboratories of Martin, Zhu, Yin, and others have established a blueprint for migratory functionalization reactions using alkenes^4^ and alkyl bromides^5^ with a variety of coupling partners to achieve benzylic functionalization, including but not limited to arylation, carboxylation, amination, and amidation. However, to realize the full potential of migratory functionalization reactions in medicinal chemistry campaigns, it is imperative to expand the scope of such reactions beyond alkene and alkyl bromide to abundant functional groups.

**Figure 1:**
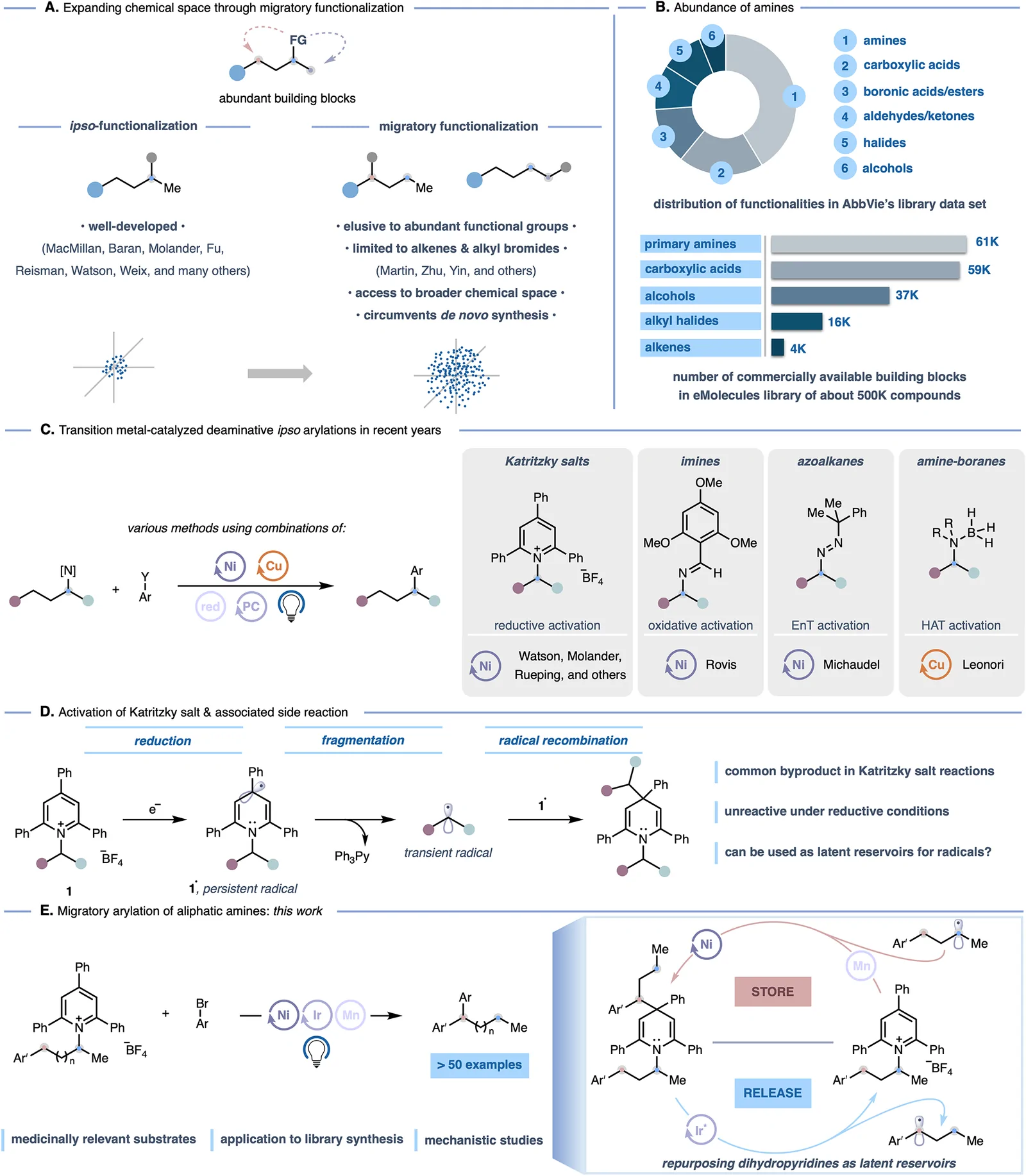
Introduction. (A) Accessing wider chemical space through migratory functionalization. (B) Abundance of amine building blocks in medicinal chemistry. (C) Recent examples of transition metal-catalyzed deaminative arylations. (D) Activation of Katritzky salts and associated challenges. (E) Nickel-catalyzed migratory arylations of aliphatic amines (this work).

Among these abundant functional groups, amines have become popular as C*sp* ^3^ synthons in recent years.^6^ Analysis of AbbVie’s internal libraries, containing approximately 18,000 building blocks, revealed amines to be the most prevalent functionality (Figure 1B).^7^ An in-depth analysis of eMolecules’ library of roughly 500,000 compounds showed that primary amines are more readily available than carboxylic acids, alcohols, alkyl halides, and alkenes (Figure 1B).^8^ Given the prevalence of primary amines, methods that employ them as precursors are critical for the expansion of the synthetic chemists’ toolbox.

In an effort to enrich molecular architectures with C*sp* ^3^ fragments, transition metal-catalyzed deaminative *ipso* arylations of primary amines have attracted continued attention in recent years (Figure 1C).^9^ Current efforts focus on the choice and activation mode of amine derivatives to generate a radical coupling partner. Notable examples of such activation modes include reductive activation of Katritzky salts,^9c,9j^ oxidative activation of imines,^9f^ triplet-energy transfer to diazoalkanes,^9h^ and H-atom transfer from amine-boranes.^9g^ Although these methods have enabled the coupling of alkyl radicals with aryl coupling partners, the functionalization site of these methods is typically predetermined by the native site of the amine functional group. As a result, such *ipso* coupling reactions generally provide access to a single constitutional isomer of the product. Developing migratory functionalization reactions using primary amines as alkyl radical precursors would provide a complementary platform for a diversified portfolio of regioisomers.

Katritzky salts have gained popularity as amine surrogates in deaminative *ipso*-coupling reactions,^9a,9j^ because of their facile synthesis and reductive activation mode.^11^ The activation mechanism for such salts is depicted in Figure 1D. Single-electron reduction of **1** generates a persistent radical **1** ^•^, which upon homolytic C–N bond cleavage releases a transient alkyl radical and triphenylpyridine. If the radical capture by a low-valent metal intermediate is kinetically competent, the resulting metal alkyl intermediate undergoes subsequent productive bond-forming steps. However, if this radical capture event is slow, the transient alkyl radical may recombine with the persistent radical **1** ^•^ to generate dihydropyridines.^9c,10b,10c,12^ Such dihydropyridines are typically unreactive under reductive coupling conditions, which are necessary for Katritzky salt activation.

Herein, we report the nickel-catalyzed migratory arylation of aliphatic amines enabled by the “store-and-release” strategy (Figure 1E). In this strategy, dihydropyridines are leveraged as latent reservoirs for translocated alkyl radicals and Katritzky salt substrates. The controlled store and release of alkyl radicals enable high yields of migratory arylated products and is particularly crucial for regioselective arylations using electron-deficient aryl bromides. Moreover, this protocol gives access to benzylic arylated products, providing a solution to an outstanding synthetic problem posed by *ipso* deaminative arylations due to a challenging synthesis of Katritzky salts of α-secondary benzylic amines, as noted by Katritzky^13^ and Watson^9b^ groups. This methodology allows for regiodivergent deaminative arylations, as demonstrated by its application for small-molecule library synthesis.

## Results and Discussion

To develop the migratory arylation of aliphatic amines, we chose 2,4,6-triphenyl-1-(4-phenylbutan-2-yl)­pyridin-1-ium tetrafluoroborate **1a** and 1-bromo-4-fluorobenzene **2a** as model substrates. Initial reaction development studies revealed that the reaction conducted with a nickel catalyst system consisting of NiBr_2_(dme) ((1,2-dimethoxyethane)nickel dibromide) and bathocuproine (2,9-dimethyl-4,7-diphenyl-1,10-phenanthroline, BC) in the presence of Mn (manganese) as a sacrificial reductant generated the arylated products **3a** and **4a** in a total of 6% yield and a 5:1 regioisomeric ratio (Table 1A, entry 1; see Figure S2 in supporting information). However, analysis of this reaction mixture showed that a significant portion of **1a** was consumed in the formation of byproducts such as alkene(s) **5**, and dihydropyridines **6** and **7**. During the initial optimization campaign, we consistently observed dihydropyridines and alkenes as the major byproducts, with the dihydropyridines accounting for a significant portion of the consumed starting material.^9c,10b,10c,14^ Since extensive evaluation of reductive cross-coupling conditions did not lead to the suppression of these byproducts, we focused on reengaging them in the productive arylation reaction.

**Table 1:**
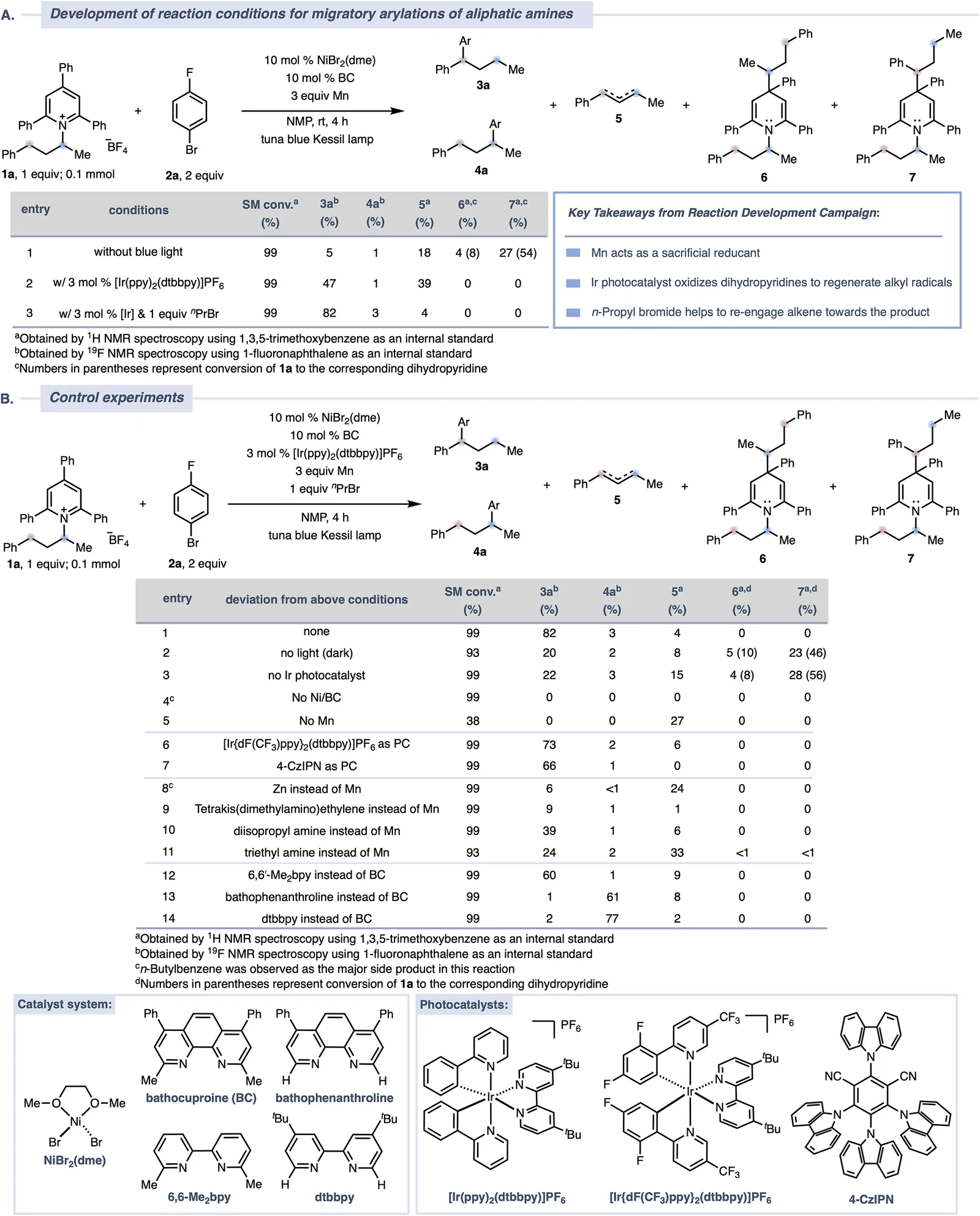
(A) Development of Reaction Conditions. (B) Control Experiments

In pursuit of this strategy, further analysis of the dihydropyridine formation revealed that the model reaction preferentially generated dihydropyridine **7** (containing the migrated alkyl fragment) over dihydropyridine **6** (containing the non-migrated alkyl fragment). Based on this observation, we hypothesized that dihydropyridines could be used as latent reservoirs for *storing* and eventually *releasing* the “migratory information”. This “store-and-release” strategy would then effectively allow for decoupling the chain-walking events, a series of *β*-hydride elimination and reinsertion events, from the coupling cycle. We recognized that structurally related Hantzsch ester derivatives have been previously reported to generate alkyl radicals through both chemical oxidation^15^ and photocatalytic oxidation.^16^ This concept was further exploited by Chen and co-workers, who demonstrated that a single-electron oxidation of dihydropyridines led to *in situ* alkyl radical release in electrophotochemical Minisci-type reactions.^17^ We envisioned that this reactivity could be uniquely leveraged for *migrated* alkyl radicals. Building on these precedents, the inclusion of a photocatalyst would allow for dihydropyridines **7** and **6** to be re-engaged in our model reaction.^18^ To test our hypothesis, we evaluated a series of photocatalysts (see Figure S3 in supporting information). Among these photocatalysts, [Ir­(ppy)_2_(dtbbpy)]­PF_6_ emerged as the optimal photocatalyst. The reaction conducted with the inclusion of 3 mol % [Ir­(ppy)_2_(dtbbpy)]­PF_6_ under visible light irradiation afforded the arylated products in 48% yield with a 47:1 rr, favoring migratory product **3a** over *ipso* product **4a** (Table 1A, entry 2). The majority of the dihydropyridines were converted to the arylated products, however, we also observed increase in the yield of alkenes **5** to 39% yield.

We next turned our attention to productively channeling the mixture of alkenes **5** into the migratory product. Presumably, alkenes **5** are observed due to the dissociation of the alkene from an intermediate nickel hydride olefin complex generated from *β*-hydride elimination of an alkyl nickel species. We hypothesized that the addition of an alkyl bromide, specifically *n*-propyl bromide, might promote the formation of a nickel hydride intermediate which can channel the alkene mixture productively into the migratory product.^5a,19^ This propenyl nickel hydride intermediate may be generated through oxidative addition of *n*-propyl bromide into a low-valent nickel species and subsequent *β*-hydride elimination. Ligand exchange between **5** and the propenyl nickel hydride intermediate can reengage **5** in the productive reaction. Zhu and co-workers have previously employed such approach in the migratory functionalization of alkenes and alkyl bromides.^5a^ To evaluate this hypothesis, we conducted the model reaction with varying amounts of *n*-propyl bromide (see Figure S4 in supporting information). Addition of 1 equiv of *n*-propyl bromide to the model reaction along with 3 mol % [Ir­(ppy)_2_(dtbbpy)]­PF_6_ and blue light irradiation improved the yield of **3a** to 82% while maintaining high regioselectivity (Table 1A, entry 3).

Control experiments revealed that both the iridium photocatalyst and blue light irradiation are required to achieve high yields (Table 1B, entries 2–3). The reaction did not form arylated products in the absence of Mn or the nickel­(II) salt and bathocuproine (Table 1B, entries 4–5). The reaction performed well in amide solvents, but lower yields were obtained in other solvents (see Figure S5 in supporting information). When the model reaction was conducted with [Ir­{dF­(CF_3_)­ppy}_2_(dtbbpy)]­PF_6_ or 4-CzIPN, an organic photocatalyst, in place of [Ir­(ppy)_2_(dtbbpy)]­PF_6_, the reaction generated migratory product **3a** in slightly lower yields (73–66%) (Table 1B, entries 6–7). Replacing the heterogeneous reductant, Mn, with Zn or commonly used homogeneous reductant such as triethyl amine, diisopropyl amine, and TDAE (Tetrakis­(dimethylamino)­ethylene) lowered the yield of the migratory product (Table 1B, entries 8–11, see Figure S7 for other reductants). 6,6′-Me_2_bpy is often featured as a ligand in nickel-catalyzed migratory arylation reactions.^3c^ When the model reaction is performed with 6,6′-Me_2_bpy in place of bathocuproine, the reaction formed product **3a** in 60% yield with a 60:1 rr (Table 1B, entry 12).

The developed protocol allows for reversal in regioselectivity by substituting bathocuproine for bathophenanthroline or 4,4′-di-*tert*-butyl-2,2′-bipyridine (dtbbpy) (Table 1B, entries 13–14). The reaction conducted with bathophenanthroline gave *ipso* product **4a** in 61% yield with 1:61 rr, highlighting the importance of the *ortho*-methyl substituents on bathocuproine for migratory arylations. Dtbbpy is routinely employed in nickel-catalyzed *ipso*-coupling reactions.^2c^ The reaction conducted with dtbbpy gave **4a** in 77% yield with a 1:38 rr, favoring the formation of **4a** over **3a**. These results demonstrate the applicability of our protocol for rapidly accessing regioisomers without drastic changes to reaction conditions and redesign of the starting materials.

With optimized reaction conditions identified, we next evaluated the scope for the migratory arylation of aliphatic amines (Figure 2). First, we evaluated electronically and sterically differentiated aryl and heteroaryl bromides. Under the optimized conditions, reactions employing electron-rich and electron-neutral aryl bromides afforded corresponding products in higher yield and regioselectivity than those with electron-deficient aryl bromides. For instance, reactions with aryl bromides bearing dimethylamino-, methoxy-, methylsulfanyl-, or fluoro-substituents at the *para* position gave the corresponding products **3a**–**3d** in 52–86% yield with 20:1 rr. However, the reaction with 1-bromo-4-(trifluoromethyl)­benzene formed the corresponding product **3e** in lower yield and regioselectivity (29% NMR yield; 3:1 rr). To address this limitation with electron-deficient aryl bromides, we modified the optimized reaction conditions. Substituting bathocuproine for 6,6′-Me_2_bpy improved the (NMR) yield of **3e** to 63%; however, the reaction still yielded the arylated products with poor regioselectivity (4:1 rr). During the optimization campaign, we observed that the reaction between **1a**, Mn, and the nickel-6,6′-Me_2_bpy catalyst system provided dihydropyridines in 44% yield (88% conversion of **1a** to the dihydropyridines) in >20:1 ratio, favoring the formation of **7** over **6** (see Section 7.4 in Supporting information for details). We hypothesized that the regioselectivity of the migratory arylation reaction could be improved by first storing the migrated alkyl radical in the dihydropyridine, *then* releasing it in the presence of the aryl coupling partner. To test this hypothesis, we reacted **1a** with 10 mol % NiBr_2_(dme) and 6,6′-Me_2_bpy, and 3 equiv of Mn in NMP for 1 h. To this reaction mixture, 1-bromo-4-(trifluoromethyl)­benzene **2e**, Ir photocatalyst, and *n*-propyl bromide were added before irradiating with blue light for 4 h. These modified conditions formed product **3e** in 71% NMR (61% isolated) yield with 20:1 rr, further supporting the “store-and-release” strategy. These conditions were then used to evaluate the scope of electron-deficient aryl and heteroaryl bromides.

**Figure 2:**
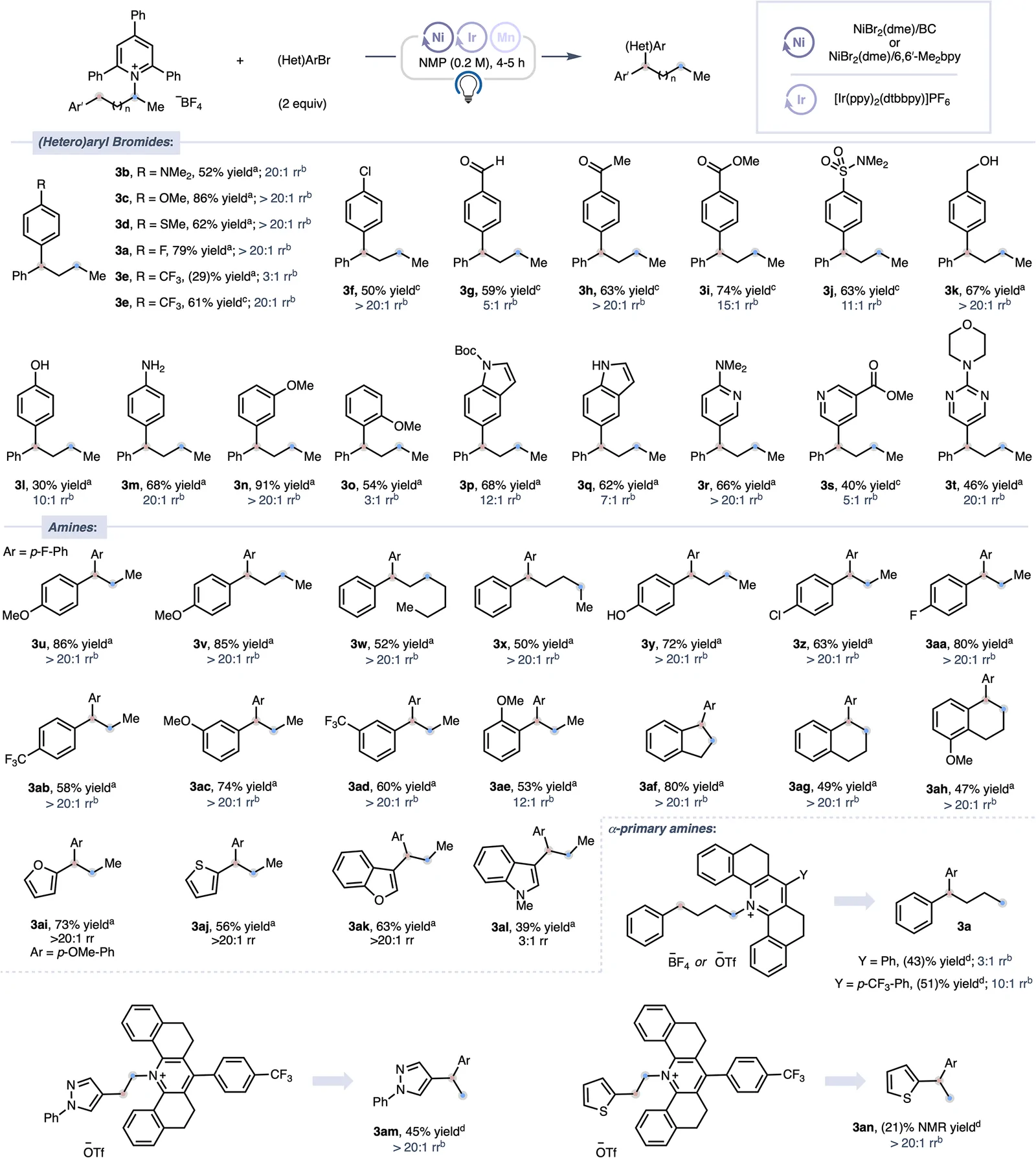
Scope of migratory arylation of aliphatic amines. Isolated yields (numbers in parentheses represent NMR yield). ^a^ **Method A**: Katritzky salt (1 equiv), (hetero)­aryl bromide (2 equiv), [Ir­(ppy)_2_(dtbbpy)]­PF_6_ (3 mol %), NiBr_2_(dme) (10 mol %), BC (10 mol %), Mn (3 equiv), *n*-propyl bromide (1 equiv), and NMP (0.2 M), tuna blue Kessil lamps (max intensity), maximum stir and fan speed, 4 h. ^b^Regioisomeric ratio (rr) was obtained by ^1^H or ^19^F NMR spectroscopic analysis of the crude reaction mixture. ^c^ **Method B**: Katritzky salt (1 equiv), NiBr_2_(dme) (10 mol %), 6,6′-dimethyl-2,2′-bipyridine (6,6′-Me_2_bpy; 10 mol %), Mn (3 equiv), and NMP (0.2 M) stirred for 1 h then (hetero)­aryl bromide (2 equiv), [Ir­(ppy)_2_(dtbbpy)]­PF_6_ (3 mol %), *n*-propyl bromide (1 equiv), tuna blue Kessil lamps (max intensity), maximum stir and fan speed, 4 h. ^d^ **Method C**: Katritzky salt (1 equiv), NiBr_2_(dme) (10 mol %), BC (10 mol %), Mn (3 equiv), and NMP (0.2 M) stirred at 50 °C for 1 h then aryl bromide (3 equiv), [Ir­(ppy)_2_(dtbbpy)]­PF_6_ (3 mol %), *n*-propyl bromide (1 equiv), ^*i*^Pr_2_NH (0.5 equiv), 425 nm (max intensity), 1100 rpm, 60 °C, 12 h.

After modifying the reaction conditions for electron-deficient aryl bromides, the migratory arylation protocol was evaluated for aryl bromides bearing secondary functionalization handles such as chloro, aldehyde, ketone, and ester groups. Reactions performed with these aryl bromides generated corresponding products **3f**–**3i** in 50–74% yield with >20:1–5:1 rr. The methodology also tolerated polar functionalities such as sulfonyl amide, hydroxy, and amine groups as demonstrated by isolation of products **3j**–**3m** in 30–68% yield (≥10:1 rr). Reactions with *meta*- and *ortho*-substituted aryl bromides such as 1-bromo-3-methoxybenzene and 1-bromo-2-methoxybenzene formed corresponding products **3n**–**3o** in 54–91% yield and >20:1–3:1 rr. The functional group tolerance observed with aryl bromides extended to heteroaryl bromides, including protected and unprotected indoles, pyridines, and pyrimidines, which afforded products **3p**–**3t** in 40–68% yield and >20:1–5:1 rr.

After evaluating the aryl coupling partners, we studied a range of primary amine derived Katritzky salts. Evaluation of their chain-length and the position of the native amine group revealed that the migratory arylation is efficient up to four carbons from the original amine site, as exemplified in products **3u**–**3x**. We next examined various substituents on the aryl ring of the Katritzky salts. The reactions using substrates bearing electron-donating, electron-neutral, or electron-withdrawing substituents on the aryl ring gave **3u–3ab** in 50–86% yield and >20:1 rr. The formation of **3y**–**3z**, featuring phenol and an aryl chloride moiety, highlights an opportunity for further downstream functionalization. The reactions of substrates containing an electron-donating methoxy-substituent on the *meta-* and *ortho-* positions of the aryl ring yielded the corresponding products **3ac** and **3ae** in 74 and 53% yields, respectively. Similarly, the reactions conducted with Katritzky salts containing *meta*-trifluoromethyl afforded product **3ad** in 60% yield. Reactions involving *meta*-substituted aryl rings showed higher regioisomeric ratios (>20:1 rr) than those involving *ortho*-substituted aryl ring (12:1 rr). The formation of migratory products **3af** (80% yield; >20:1 rr), **3ag** (49% yield; >20:1 rr) and **3ah** (47% yield; >20:1 rr) demonstrates the applicability of this method to substrates derived from cyclic amines. Particularly, the formation of **3ah** showcases the effect of an *ortho*-substitution on the aryl ring on the regioselectivity of migratory arylation. After evaluation of electronics and sterics on the aryl ring of the Katritzky salt, we next applied the developed protocol to the migratory arylations of Katritzky salts bearing heteroaryl rings. The migratory arylation of furan, thiophene, benzofuran, and indole-containing Katritzky salts formed the corresponding products **3ai–3al** in 39–73% yields (3:1–>20:1 rr).

Katritzky salts derived from non-benzylic, *α*-primary amines have traditionally been challenging substrates in deaminative functionalization reactions and typically require elevated reaction temperatures (>60 °C) for cross-couplings.^20^ Electrochemical studies show that their reduction is fully reversible, indicating that back electron transfer outcompetes C–N fragmentation to release the primary alkyl radical, in contrast to secondary pyridinium salts that show quasi-reversible or irreversible traces consistent with faster fragmentation.^21^ Consistent with these reports, we observed that Katritzky salt of 4-phenylbutan-1-amine failed to deliver the migratory product under our optimized conditions, even at 60 °C. However, when we employed the tetrahydrodibenzoacridinium salt of the *α*-primary amine, the corresponding product **3a** was formed in 43% NMR yield with 3:1 regioisomeric ratio.^22^ Presumably, the tethered pyridinium salt should increase the rate of C–N bond (in the pyridinium salt) and C–C bond (in dihydropyridine) cleavage and generate the corresponding alkyl radical. Changing the identity of the aryl group at the *para* position of the tetrahydrodibenzoacridinium salt from phenyl to 4-trifluorophenyl resulted in increased regioselectivity (10:1 rr) with slightly improved yield of the migrated product. We next applied our method to migratory arylations of tetrahydrodibenzoacridinium salts of *α*-primary amine containing heteroaryl groups, such as pyrazole (**3am**, 45% yield, >20:1 rr) and thiophene (**3an**, 21% NMR yield, >20:1 rr).^23^ These results show that the store-and-release strategy can be applied to pyridinium salts beyond 2,4,6-triphenylpyridinium salts.

We next applied our methodology to the substrates derived from medicinally relevant compounds (Figure 3). We evaluated aryl bromides derived from drug molecules such as Celecoxib, Ketorolac, and strychnine. Migratory arylation of **1a** with these aryl bromides gave corresponding products **3ao–3aq** in 48–62% yields with ≥13:1 rr. The access to products **3ao–3aq** demonstrates the compatibility of our method with functionalities such as sulfonyl amides, amides, ketones, alkenes, and basic nitrogens. The reaction of **1a** with aryl bromide derived from Thalidomide, a CELMoD candidate, provided product **3ar** in 66% yield with >20:1 rr. We demonstrated that keeping the aryl bromide **2ao** (*from* Celecoxib) as the limiting reagent and using Katritzky salt **1a** in excess gave **3ao** in 73% NMR yield and 15:1 rr, highlighting applicability of the method when valuable aryl bromides are used. Migratory arylation of Katritzky salts derived from Olopatadine, Nabumetone, 3-methoxytyramine (a dopamine metabolite), phenylalaninol, Lumacaftor, Naproxen, and Febuxostat with 1-bromo-4-fluorobenzene **2a** generated products **3as–3ay** in 39–82% yield with >10:1 rr. The results obtained from the reactions of Olopatadine derivative, Nabumetone derivative, 3-methoxytyramine, and phenylalaninol showcase the applicability of the method to functionalize medicinally relevant compounds. Additionally, the formation of migratory products **3aw–3ay** demonstrates robust functional group tolerance towards functionalities such as basic nitrogens, esters, geminal dihalides, and nitriles.

**Figure 3:**
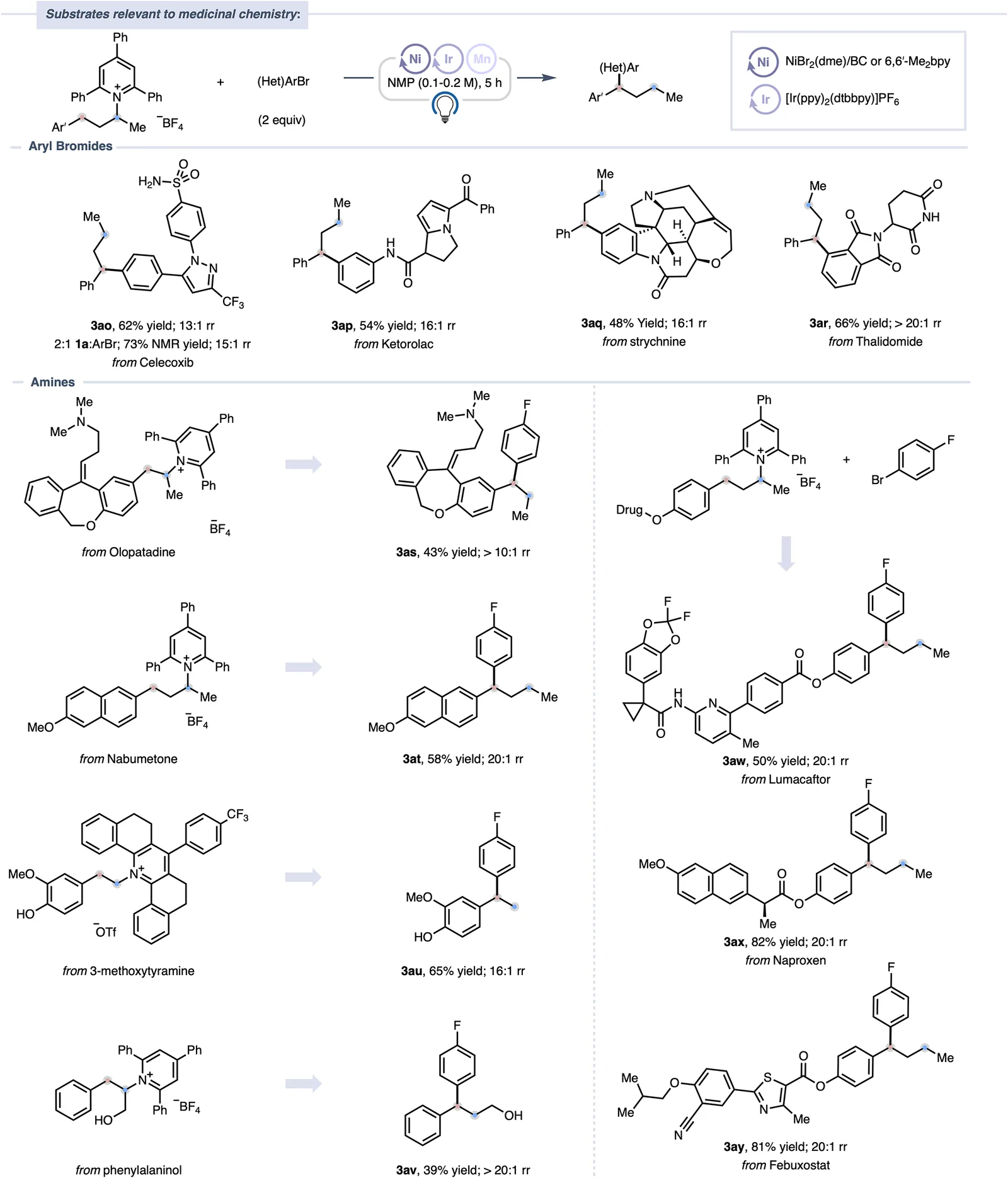
Migratory arylations of substrates relevant to medicinal chemistry (see Supporting information for reaction details).

After establishing the methodology for migratory arylations, we next evaluated its adoption to regiodivergent deaminative arylations for small-molecule library synthesis (Figure 4). The library synthesis experiment involved simultaneous screening of six (hetero)­aryl bromides and four Katritzky salts in a plate-based, matrixed library for migratory and *ipso* arylations. All reaction components, except Mn, were efficiently dosed as NMP stock solutions with only the Ni precatalyst stock solution differing from the first half (migratory arylations with nickel-6,6′-Me_2_bpy) to the second half (*ipso* arylations using nickel-dtbbpy). This experiment led to successful formation of 39 arylation products, of which 16 pairs of products were regioisomers. These results demonstrate the potential of the developed protocol for accessing regioisomers and thus, providing access to wider chemical space using the same set of starting materials.

**Figure 4:**
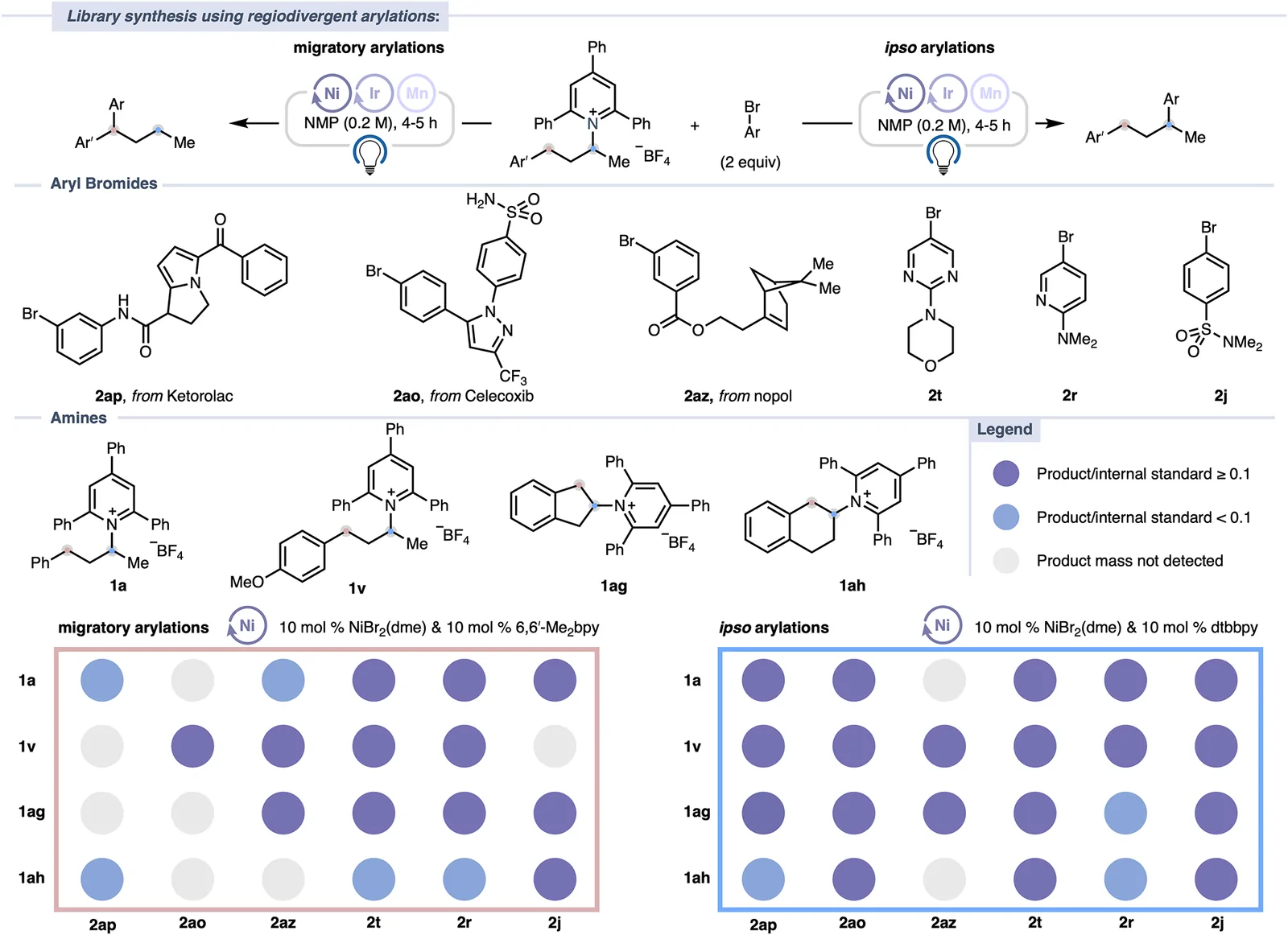
Application of migratory arylation of aliphatic amines for small-molecule library synthesis (selectivity of representative library reactions was determined by LC and ^1^H NMR spectroscopy; see Section 8 in the Supporting information for details on experimental set up).

After evaluating scope and synthetic applications of the developed protocol, we next pursued mechanistic studies. The initial mechanistic studies began with the evaluation of canonical mechanistic pathways (Figure 5A–B). Nickel-catalyzed, cross-electrophile coupling reactions have been previously proposed to occur through two distinct mechanistic pathways: (1) the nickel(0) species reacts with the aryl bromide first by oxidative addition followed by radical capture to form a nickel­(III) intermediate;^9^ (2) the nickel(0) species captures the alkyl radical first to form a nickel­(I) alkyl intermediate, which then undergoes oxidative addition with the aryl bromide to form a nickel­(III) intermediate.^9,16^ To test whether these manifolds could lead to the formation of the migratory product, we evaluated the steps following the radical capture in each manifold using density functional theory (DFT) at the M06-L/def2-TZVP/CPCM­(DMA)//M06-L/def2-SVP level of theory (see Section 7.1 in Supporting information for further details).

**Figure 5:**
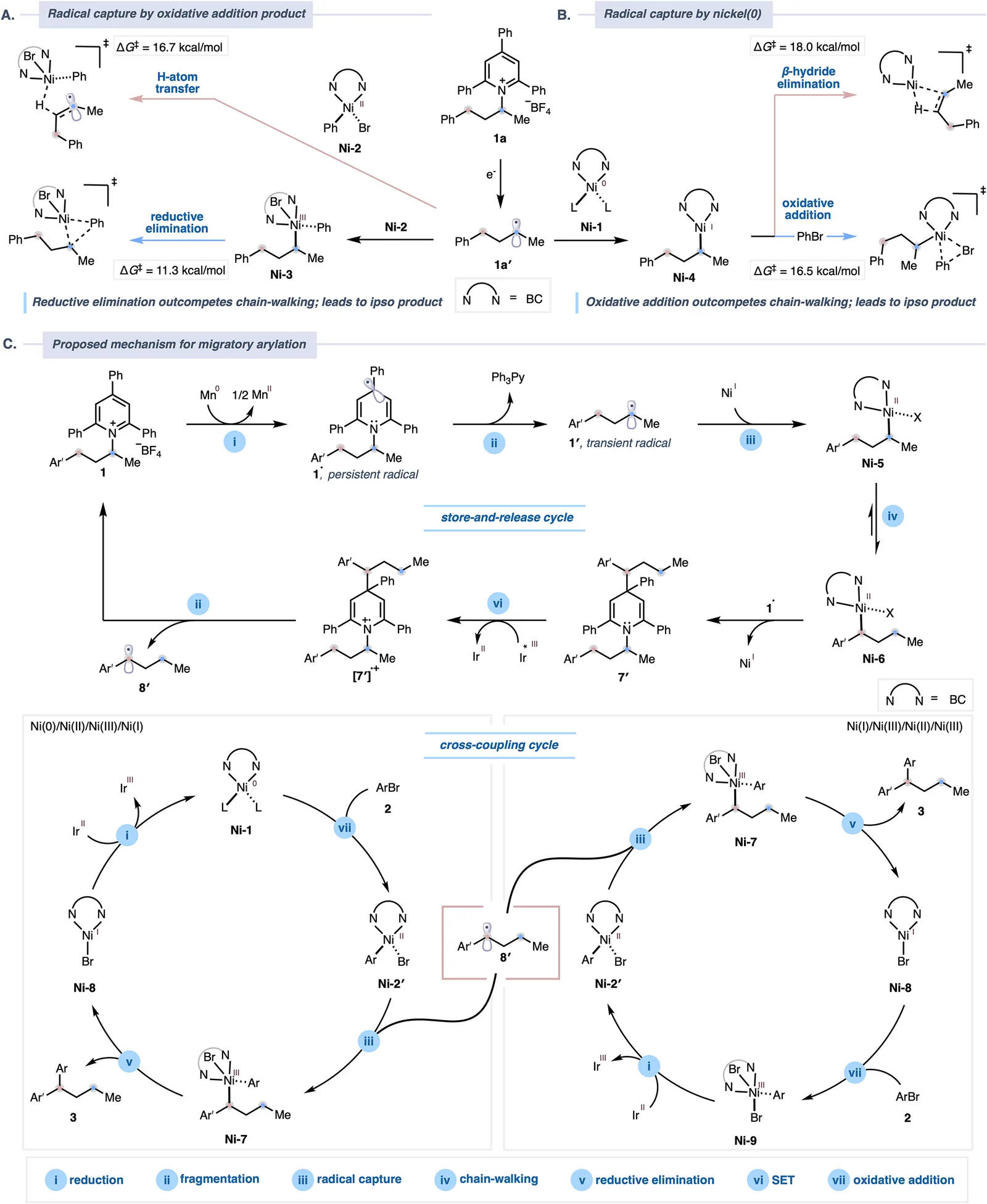
DFT Studies and Proposed Mechanism. Evaluation of mechanistic manifolds involving: (A) radical capture by oxidative addition product. (B) radical capture by a nickel(0) complex; DFT calculations were performed at M06-L/def2-TZVP/CPCM­(DMA)//M06-L/def2-SVP level of theory (see Section 7.1 in Supporting information for details). (C) Proposed mechanism for nickel-catalyzed migratory arylation of aliphatic amines.

First, we considered the capture of **1a**′ by the oxidative addition product **Ni-2**, which was obtained from the reaction between phenyl bromide and **Ni-1** (Figure 5A). Upon the capture of **1a**′, a nickel­(III) species, **Ni-3**, was generated. To investigate if **Ni-3** could lead to the formation of the migratory product, we investigated the barriers for reductive elimination and β-hydride elimination from **Ni-3**. DFT studies showed that the barrier for reductive elimination from **Ni-3** was 11.3 kcal/mol. Although the transition-state for β-hydride elimination could not be located, the transition-state for a hydrogen-atom transfer pathway, which would lead to the intermediate of the chain-walking process, was found to have a higher energy barrier (Δ*G* ^‡^ = 16.7 kcal/mol).^24^ These results suggest that the formation of the migratory product from the radical capture of **1a**′ by **Ni-2** is unlikely and thus, this pathway may favor the formation of the *ipso* product.

In another mechanistic manifold, the alkyl radical could be captured by **Ni-1** first to form **Ni-4** (Figure 5B). To test whether **Ni-4** could lead to the formation of the migratory product, we evaluated the barriers for *β*-hydride elimination and oxidative addition of phenyl bromide to **Ni-4**. The barrier for oxidative addition was 1.5 kcal/mol lower than that for β-hydride elimination, suggesting that oxidative addition may outcompete β-hydride elimination. The oxidative addition of phenyl bromide to **Ni-4** would lead to **Ni-3** and consequently favor the formation of the *ipso* product, based on the results presented in Figure 5A. Taken together, these results suggest that the formation of the migratory product may not occur through the intermediacy of **Ni-3** or **Ni-4**. Since these canonical mechanistic manifolds^25^ may not account for the formation of the migratory product, we propose an alternative mechanism in which the radical translocation event is decoupled from and precedes the cross-coupling cycle.

A mechanistic proposal for the formation of the migratory product is depicted in Figure 5C. In line with the previous reports on reductive activation of Katritzky salts, **1** undergoes a single-electron reduction first by Mn. Although Mn (–1.50 V vs Fc^+^/Fc in NMP)^26^ can reduce **1a** and act as a sacrificial reductant, we found that **1a** can also be reduced by a nickel(0) species, (BC)_2_Ni,^27^ to form the persistent radical and a nickel­(I) species (see Section 7.5 in Supporting information for further details). This persistent radical, upon fragmentation, generates a transient alkyl radical **1**′, which is captured by a nickel­(I) species to form an alkyl nickel­(II) species, **Ni-5**. **Ni-5** then undergoes chain-walking to form the thermodynamically stable benzylic nickel­(II) species, **Ni-6** (Δ*G* = –8.5 kcal/mol for Ar′ = Ph). In an alternative scenario, the reduction of (BC)­Ni­(II)­Br_2_ to Ni(0) by Mn could be relatively slow and would result in an accumulation of (BC)­Ni­(I)­Br. In this scenario, the transient radical **1′** could be captured by (BC)­Ni­(I)Br to generate a similar nickel­(II) intermediate. Nonetheless, both scenarios would lead to the thermodynamically preferred benzylic nickel­(II) regioisomer (see Figure S15 in supporting information). The reaction of **1** ^•^ with **Ni-6** then leads to the formation of dihydropyridine **7′** and regenerates a nickel­(I) species, demonstrating *storage* of the translocated alkyl radical. To probe the formation of **7′**, we reacted **1a** with Mn in the presence and the absence of catalytic amount of NiBr_2_(dme) and bathocuproine. The reaction provided the dihydropyridine **6** exclusively in the absence of the precatalyst, whereas the formation of **7** (43% yield; 43:1 rr) was observed in the presence of the precatalyst (Figure 6A). These observations support that the radical capture of **1**′ by a nickel/bathocuproine species leads to the formation of **7′**.

**Figure 6:**
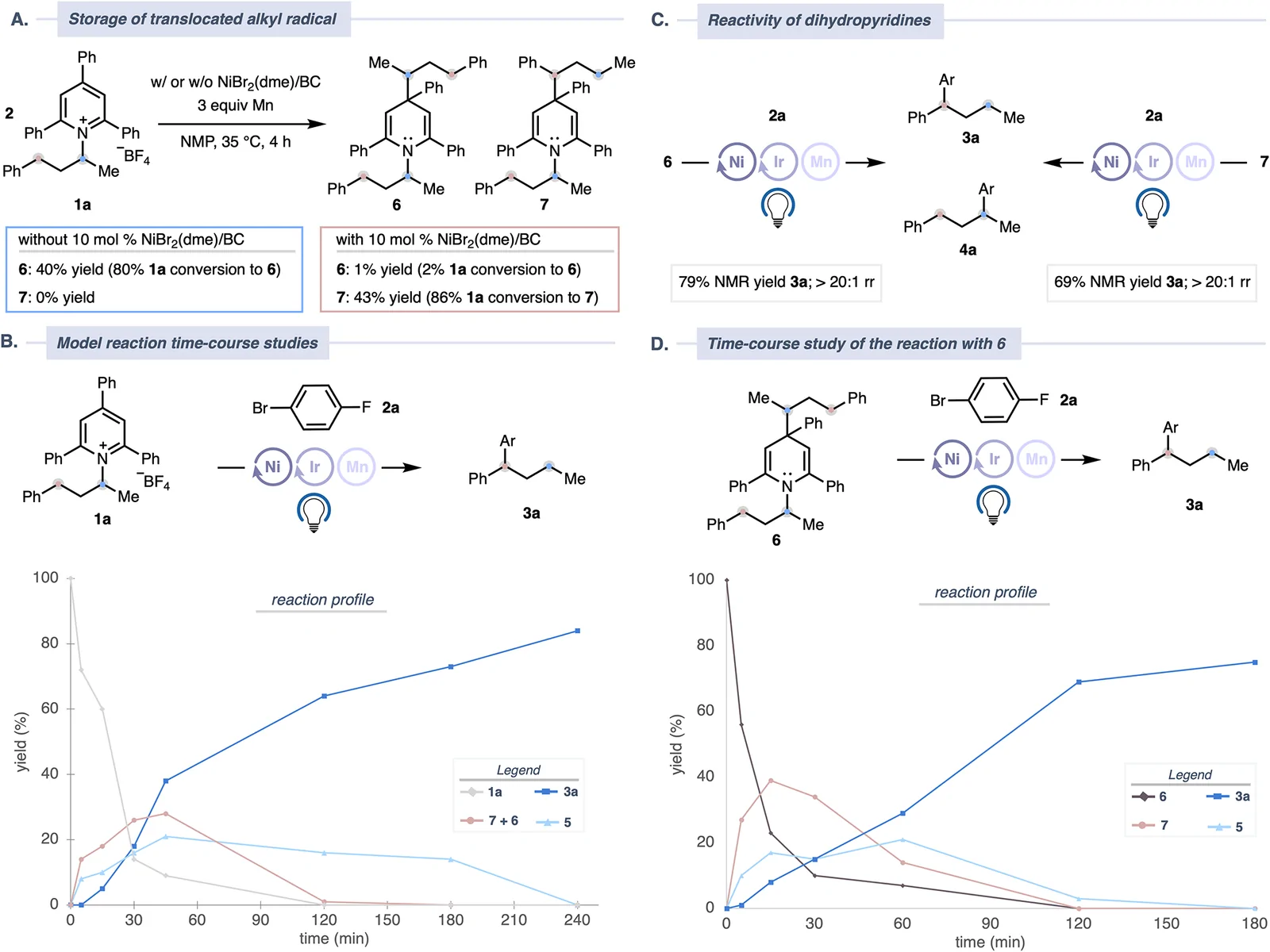
Preliminary mechanistic studies. (A) Formation of dihydropyridines. (B) Model reaction time-course studies. (C) Reactivity of dihydropyridines. (D) Time-course study of the reaction with **6**. **5**, Mixture of alkenes.

A single-electron oxidation of **7′** by an excited state photocatalyst Ir*­(III) (*E*_1/2_ Ir*­(III)/Ir­(II) = +0.28 V vs Fc^+^/Fc in MeCN)^28^ results in the formation of amine radical cation **[7′]** ^•**+**^. This radical cation then undergoes fragmentation to generate alkyl radical **8′** and Katritzky salt **1**. To evaluate this hypothesis, we conducted cyclic voltammetry (CV) studies of dihydropyridine **7** (see Section 7.2 in Supporting information for further details). The CV studies of **7** revealed an irreversible oxidation peak (*E*_p/2_ = +0.23 V vs Fc^+^/Fc in MeCN), when scanned in the positive direction and an irreversible reduction peak (*E*_p/2_ = -1.27 V vs Fc^+^/Fc in MeCN) when followed with scanning in the negative direction. This reduction peak matched with the peak observed in the independent CV of **1a** (*E*_p/2_ = -1.30 V vs Fc^+^/Fc in MeCN), supporting its generation from **7** upon oxidation. The same characteristic peaks were observed in the cyclic voltammogram of **6**. Taken together, the CV studies support the *release* of the translocated alkyl radical and the regeneration of Katritzky salt upon single-electron oxidation of the dihydropyridine **7**. Additionally, the Stern–Volmer luminescence quenching studies support quenching of the excited state of the Ir photocatalyst by dihydropyridine **7** (see Section 7.3 in Supporting information for further details).

Upon the release of the translocated alkyl radical **8′** from **7**, it then enters the cross-coupling cycle. For the cross-coupling cycle, two plausible catalytic manifolds are proposed. In the first cycle, after the reduction of (BC)­NiBr_2_ by Mn to **Ni-1**,^27^ the oxidative addition of aryl bromide **2** into **Ni-1** results in the formation of an aryl nickel­(II) bromide, **Ni-2**′. The radical capture of **8′** by **Ni-2**′ forms a nickel­(III) intermediate, **Ni-7**. This nickel­(III) species then undergoes reductive elimination to form product **3** and generates a nickel­(I) bromide, **Ni-8**. Single-electron reduction of **Ni-8** by Ir­(II) (*E*_1/2_ Ir­(III)/Ir­(II) = -1.89 V vs Fc^+^/Fc in MeCN)^28^ regenerates Ir­(III) photocatalyst and **Ni-1**. In the second cycle,^29^ oxidative addition of **2** into **Ni-8** leads to another nickel­(III) intermediate **Ni-9**, which then gets reduced by Ir­(II) to generate **Ni-2**′.^30^ The radical capture of **8′** by **Ni-2**′ then generates **Ni-7**, which undergoes reductive elimination to form the product **3** and regenerate **Ni-8**. Regardless of the cross-coupling cycle, the overall proposed mechanism underscores the decoupling of the radical translocation event from the cross-coupling cycle.

This proposed mechanism is consistent with the following observations: (1) Time-course study of the model reaction revealed that at the early time points of the model reaction, the majority of **1a** is converted to the dihydropyridines, **7** being the major dihydropyridine (Figure 6B; see Section 7.6 in Supporting information for details). The initial accumulation and eventual consumption of the dihydropyridine leads to an increased amount of the migratory product **3a**, suggesting that the primary source of the alkyl radical for migratory product formation is **7** and not **1a**. Additionally, this study also supports the funneling of the alkenes **5** towards the migratory product under the model reaction conditions. (2) When **6** and **7** are exposed to the model reaction conditions in place of **1a**, the reactions afforded **3a** in 79 and 69% yield respectively, with >20:1 rr (Figure 6C). These results suggest that both dihydropyridines are competent substrates under the model reaction conditions. To investigate the high regioselectivity observed in the reaction with **6**, we conducted time-course studies (Figure 6D). These studies revealed that at early time points of the reaction, **6** primarily was converted to **7** first, similar to the model reaction with **1a**. Consistent with the observations stated above, the high regioselectivity could be the result of decoupled storage of the translocated alkyl radical in **7′**. (3) Faster rate of oxidative addition of aryl bromides leads to lower regioselectivity for the migratory product due to low concentration of Ni­(I) available for translocation of the alkyl radical (as depicted in the store-and-release cycle).^29,31^ For instance, the reaction with electron-deficient aryl bromide **2e** leads to lower regioselectivity (*vide supra*), requiring separation of the storage and the release events in the reaction set up (described as Method B in Figure 2). Additionally, increasing the concentration of electron-rich aryl bromide, such as **2c**, leads to lower regioselectivity (2 equiv: 86% yield, > 20:1 rr vs 5 equiv: 53% ^1^H NMR yield, 3:1 rr).

## Conclusions

In conclusion, we report a “store-and-release” strategy for nickel-catalyzed migratory arylations of aliphatic amines. The strategy relies on leveraging dihydropyridines for the storage and release of translocated alkyl radicals, effectively decoupling the chain-walking process from the cross-coupling cycle. This protocol showcases a broad functional group tolerance for both coupling partners, including substrates derived from medicinally relevant compounds, thus making it suitable for late-stage functionalization. The developed methodology was applied for small-molecule library synthesis, demonstrating its application in regiodivergent arylations. Preliminary mechanistic studies demonstrate that under the model reaction conditions there is accumulation of dihydropyridine **7**, which stores the translocated radical, meanwhile, its oxidation by the iridium photocatalyst releases the radical for the arylation reaction. Although studies to further evaluate the intricacies of the cross-coupling cycle are currently on-going in our laboratory, these preliminary studies provide strong support for the decoupled migration and arylation cycles. Ultimately, this work provides a blueprint for the “store-and-release” strategy, which can be applied to other migratory functionalization reactions.

## Supplementary Material





## Notes

Additional experimental details, optimization and control studies, substrate synthesis and substrate scope protocols, mechanistic studies, and spectra (PDF)
Coordinates from calculated structures using Density Functional Theory (XYZ)

## Abbreviations

BC: bathocuproine
dme: 1,2-dimethoxyethane
Mn: manganese
NMP: *N*-methyl-2-pyrrolidone
DMA: dimethylacetamide
MeCN: acetonitrile
dtbbpy: 4,4′-di-*tert*-butyl-2,2′-dipyridyl
rr: regioisomeric ratio
DFT: density functional theory
CV: cyclic voltammetry.
