# Supplementary material for: “Store-and-Release” Strategy Enables Nickel-Catalyzed Deaminative Migratory Arylation of Aliphatic Amines
Source: J Am Chem Soc. 2026 Sep 8;148(37):39776–89. doi: 10.1021/jacs.6c09936 (PMC13614205; doi:10.1021/jacs.6c09936)
Supplement: Supplementary file 1 [file ja6c09936_si_001.pdf]

Supporting Information

"Store-and-Release" Strategy Enables Nickel-Catalyzed Deaminative Migratory Arylation of Aliphatic Amines

Abhishek A. Kadam[1‡], Kevin Lee[1‡], Yumeng Liao[1], Aleksandr Knyazev[1], M. Joana Lourenço[1], Pinku Tung[1], Anthony N. Cauley[2], Jesús M. Dones[2], Martins S. Oderinde[2], James Kempson[2], and Jose B. Roque[1, *]

[1]Department of Chemistry, Princeton University, Princeton, New Jersey 08544, United States.

[2] Global Discovery Chemistry, Bristol Myers Squibb Research & Early Development, Route 206 & Province Line Road, Princeton, New Jersey 08543, United States.

[‡]Equal Authors

Corresponding Author: jroque@princeton.edu*

Table of contents

1. General Considerations, Materials, and Instrumentation 3

1.1. General Considerations 3

1.2. Materials 3

1.3. Instrumentation 4

2. Standard Reaction Setup 5

3. Reaction Optimization and Control Studies 6

3.1. General Procedure A for Reaction Optimization and Control Studies 6

3.2 Evaluation of Reductants for Reductive Cross-Coupling Conditions 7

3.3 Effect of Addition of Photocatalyst 8

3.4 Effect of Addition of *n*-Propyl Bromide 9

3.5 Evaluation of Solvents 10

3.6 Evaluation of Ligands 11

3.7 Control Studies 12

3.8 Evaluations of aryl halide coupling partner 14

4. Synthesis of Substrates 15

4.1 Preparation of Katritzky Salts 15

4.2 Characterization of Unknown Katritzky Salts 19

4.3 Synthesis and Characterization of Aryl Bromides 36

5. Synthesis and Characterization of Dihydropyridines 6 and 7 39

6. Evaluation of Substrate Scope for Nickel-Catalyzed Migratory Arylation of Aliphatic Amines 42

6.1 General Procedure 1 for Evaluation of (Hetero)Aryl Bromides for Nickel-Catalyzed Migratory Arylation of Aliphatic Amines 42

6.2 General Procedure 2 for Evaluation of Katritzky Salts for Nickel-Catalyzed Migratory Arylation of Aliphatic Amines 44

6.3 Characterization *of Nickel-Catalyzed Migratory Arylation of Aliphatic Amines Products* 47

7. Mechanistic Studies 88

7.1 Computational Studies 88

7.2 Cyclic Voltammetry ... 99
7.3 Stern-Volmer Luminescence Quenching Studies ... 103
7.4 Dihydropyridine Formation ... 105
7.5 Reduction of Katritzky salt 1a by a nickel(0) complex of bathocuproine 107
7.6 Time-Course Studies ... 109
7.7 Reactivity of dihydropyridines ... 110
7.8 Time-course study of the reaction of dihydropyridine 6 under model reaction conditions ... 113
7.9 Other pathways considered ... 114
8. Library Synthesis ... 116
9. Limitations of the method ... 126
10. Preliminary Attempts at Enantioselective Reaction ... 128
11. NMR Spectra ... 130
12. References ... 266

1. General Considerations, Materials, and Instrumentation

1.1. General Considerations

All air- and moisture-sensitive techniques were carried out using standard Schlenk techniques on a Schlenk line or a high-vacuum line or in an M. Braun glovebox containing an atmosphere of N_2. The glovebox was equipped with vacuum feedthroughs, a cold well, and a freezer for storing samples at -35 °C. Colors are described in comparison to the complete list of Prismacolor colored pencils. Flash column chromatography was performed on SiliFlash® P60 silica gel (40-63 µm, 60 Å) using a Yamazen Smart Flash W-Prep 2XY automatic system. HPLC purification was performed on a Teledyne ISCO ACCQPrep HP150 system. Thin-layer chromatography (TLC) was performed using aluminum-backed plates pre-coated with silica gel and a fluorescent indicator for visualization upon UV irradiation. Preparative thin-layer chromatography was performed using TLC Silica gel 60G F_{254} glass plates (20 X 20 cm).

1.2. Materials

Deuterated solvents for NMR spectroscopy analysis of air sensitive nickel complexes (benzene-d6) were distilled from sodium metal under high vacuum and stored in the glovebox over potassium carbonate. All liquid reagents used in the synthesis of metal precursors were dried by stirring over CaH_2 under static vacuum at 23°C for 24 hours and then distilled under high vacuum and stored in the glovebox. Solid substrates and synthesized ligands were dried under high vacuum for 24 hours at 23°C and then stored in the glovebox. $NiBr_2$(dme) (CAS # 28923-39-9) was purchased from TCI. Bathocuporine and 6,6′-dimethyl-2,2′-bipyridine were purchased from Ambeed. Anhydrous *N*-methyl-2-pyrrolidone (NMP) was purchased from Sigma Aldrich and used without further purification. [Ir(ppy)$_2$(dtbbpy)]PF_6 and 2,4,6-triphenylpyrylium tetrafluoroborate were purchased from AA Blocks and used without further purification. Manganese (Mn) powder (-325 mesh, 99.3% metal basis) was purchased from Thermo Scientific and used as received.

1.3. Instrumentation

^{1}H NMR spectra were recorded on either Bruker BioSpin AG, Bruker AVANCE 400 or 500 spectrophotometers operating at 300 MHz, 399.8 MHz, and 500.46 MHz, respectively. ^{13}C NMR spectra were recorded on Bruker AVANCE 500 spectrometer operating at 125.85 MHz. ^{19}F NMR spectra were recorded on either Bruker BioSpin AG or Bruker AVANCE 500 spectrometer operating at 282 and 471 MHz, respectively, and are reported based on indirect reference to $CDCl_3$.[1] ^{11}B {^{1}H} NMR spectra were recorded on Bruker AVANCE 500 spectrometer operating at 160 MHz. ^{1}H and ^{13}C {^{1}H} NMR chemical shifts are reported in ppm using the ^{1}H (chloroform-d_3: 7.26 ppm, acetone-d_6: 2.05 ppm) and ^{13}C (chloroform-d_3: 77.16 ppm, acetone-d_6: 29.84 ppm) chemical shifts of the solvent as a reference. ^{1}H NMR data for diamagnetic compounds are reported as follows: chemical shift, multiplicity (s = singlet, d = doublet, t = triplet, q = quartet, p = pentet, h = hextet, sept = septet, dd = doublet of doublets, td = triplet of doublets, dt = doublet of triplets, tt = triplet of triplets, dq = doublet of quartets, tdd = triplet of doublet of doublets, dtd = doublet of triplet of doublets, ddt = doublet of doublet of triplets, qd = quartet of doublets, qt = quartet of triplets, pd = pentet of doublets, br = broad, m = multiplet), coupling constants (Hz), integration. ^{13}C NMR data for diamagnetic compounds are reported as follows: chemical shift.

High-resolution mass spectra (HRMS) were obtained at Princeton University mass spectrometry facilities using either an Agilent 6230 TOF LC/MS, Agilent 7200 Accurate-Mass Q-TOF GC/MS or 7250 GC/Q-TOF.

2. Standard Reaction Setup

All reactions were carried out as described in general procedures in the following sections. The photoreactor used for these reactions is shown in Figure S1 below. The photoreactor is an in-house set up which consists of 4 tuna blue Kessil lamps placed in a wooden frame on top of IKA® C-MAG MS 7 equipped with 2 Noctua NF-F12 iPPC cooling fans. For all reactions, maximum intensity on tuna blue Kessil lamps was used with maximum stir rate (6 on the stir dial, 1500 RPM) and maximum fan speed (3000 RPM). The reaction vials were placed roughly 1.0-1.5 cm away from the Kessil lamps. With this photoreactor setup, 12 simultaneous reactions can be conducted for multi-reaction setups. When all 4 Kessil lamps are turned on, with maximum fan speed, the photoreactor temperature was found to be approximately 35 °C. We thank Thomas Martin (Knowles lab) for help with the photoreactor set up.

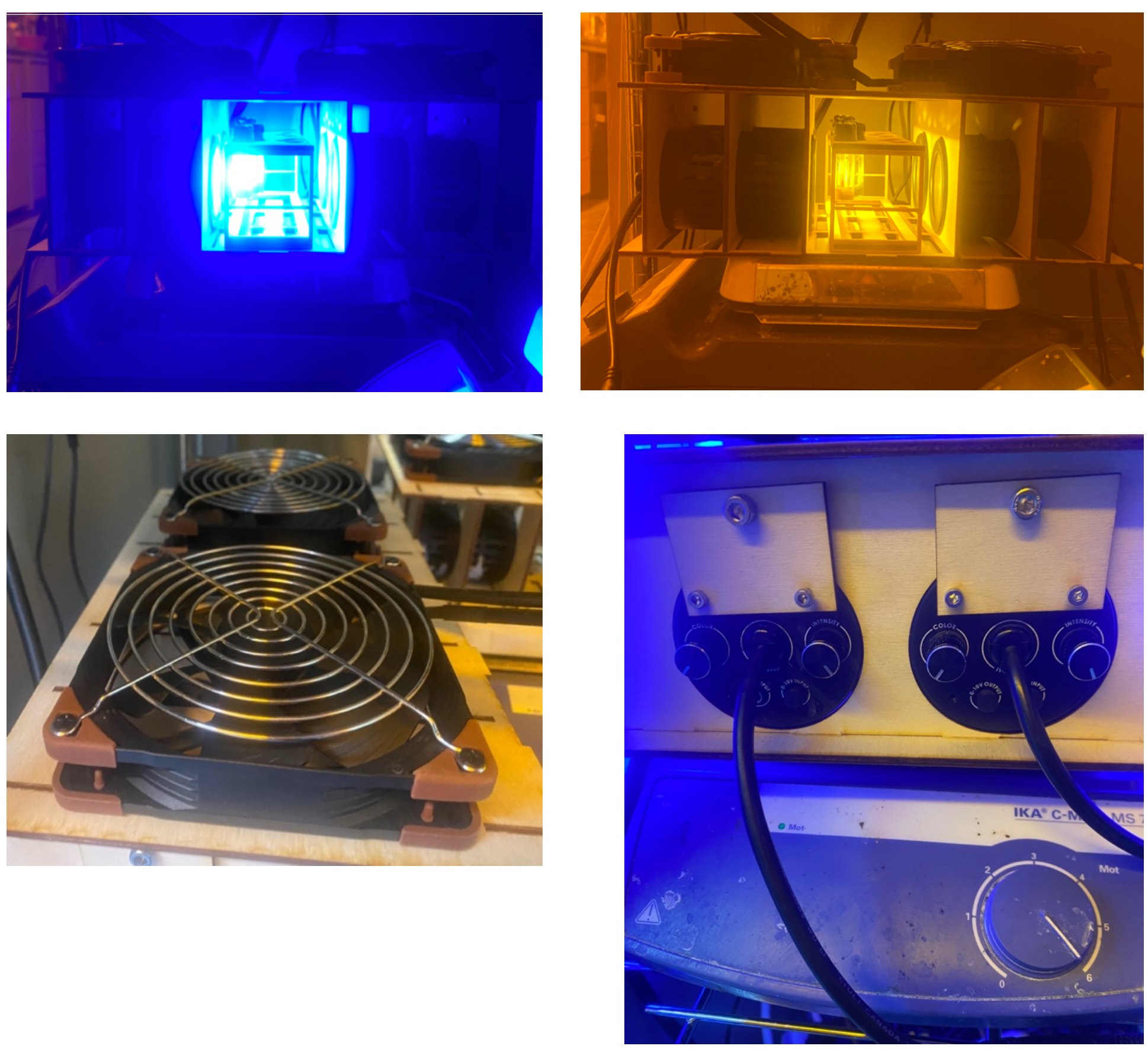

Figure S1: Photoreactor setup.

3. Reaction Optimization and Control Studies

3.1. General Procedure A for Reaction Optimization and Control Studies

3.1.1 *Preparation of a stock solution of the precatalyst (0.20 M)*:

Inside a nitrogen-filled glovebox, a 1-dram vial equipped with a PTFE-coated magnetic stir bar was charged with $NiBr_2$(dme) (10 mol %), bathocuproine (BC) (10 mol %), and NMP (0.20 M). The vial was hermetically sealed with a syringeable cap (PP/PTFE/silicone liner septum), and the resulting solution was stirred for 10 min. The formation of a homogeneous “process red” (PC 994) solution indicated the generation of the bathocuproine-ligated nickel precatalyst. The precatalyst vial was then removed from the glovebox for use in the reaction setup described below.

3.1.2 *Reaction setup*:

Inside a nitrogen-filled glovebox, a 1-dram vial equipped with a PTFE-coated magnetic stir bar was charged with $[Ir(ppy)_2(dtbbpy)]PF_6$ photocatalyst (3 mol %, 0.003 mmol, 2.70 mg), followed by 2,4,6-triphenyl-1-(4-phenylbutan-2-yl)pyridin-1-ium tetrafluoroborate **1a** (1.00 equiv, 0.100 mmol, 52.7 mg) and manganese powder (3.00 equiv, 0.300 mmol, 16.5 mg). The vial was then hermetically sealed with a syringeable cap (PP/PTFE/silicone liner septum) and removed from the glovebox. Under a positive nitrogen pressure, the reaction vial was charged with 1-bromo-4-fluorobenzene **2a** (2.00 equiv, 0.200 mmol, 22 µL) and *n*-propyl bromide (1.00 equiv, 0.100 mmol, 9 µL), followed by 0.500 mL of the precatalyst solution (described in 3.1.1). The vial was then sealed with M grease, secured with electrical tape, and irradiated with tuna blue Kessil lamps with maximum intensity, fan speed, and stir rate.

After completion of the reaction, 1-fluoronaphthalene (0.100 mmol) was added to the reaction mixture, followed by 1.00 mL of a 1 M solution of trimethoxybenzene in ethyl acetate. The mixture was diluted with ~0.5 mL of 10% aqueous LiCl solution and gently shaken. The resulting biphasic mixture was allowed to settle, and approximately 0.3 mL of the organic layer was withdrawn for analysis by ^{19}F NMR spectroscopy. The remaining mixture was diluted with ~2 mL of ethyl acetate, shaken, and allowed to settle. The aqueous layer was decanted, and the organic layer was washed with ~1 mL of brine,

shaken, and allowed to settle. After decanting the aqueous layer, the organic phase was dried over Na_2SO_4, filtered through a short pad of Celite® topped with Na_2SO_4, concentrated under reduced pressure, and analyzed by ^1H NMR spectroscopy.

3.2 Evaluation of Reductants for Reductive Cross-Coupling Conditions

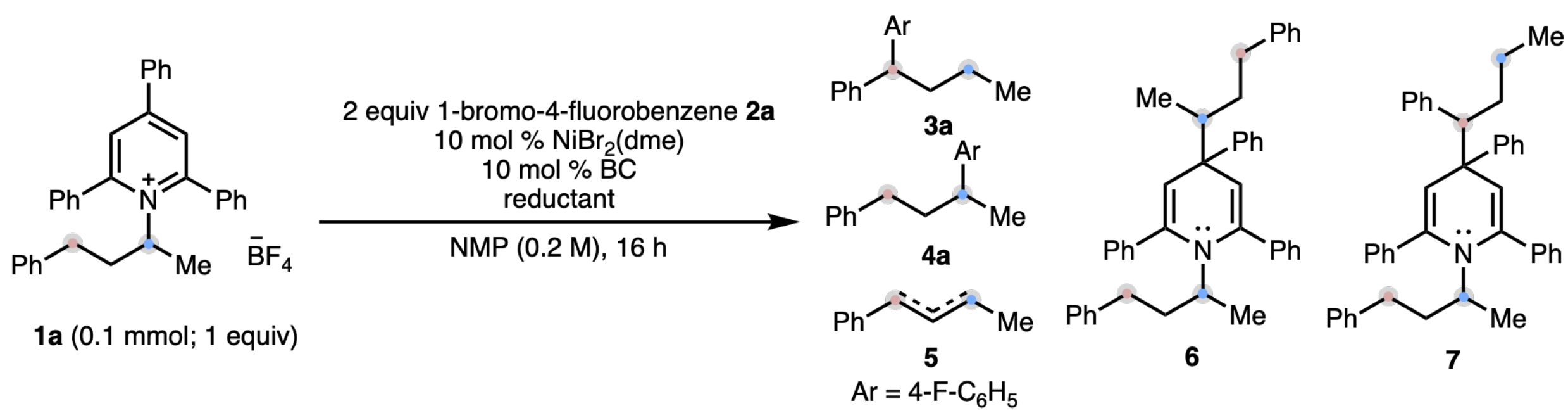

Entry	reductant	1a conv.[a] (%)	3a[b] (%)	4a[b] (%)	5[a] (%)	6[a] (%)	7[a] (%)
1	No reductant	0	0	0	0	0	0
2	1 equiv Mn	83	7	7	20	3 (6)[c]	17 (34)[c]
3	3 equiv Mn	99	5	1	18	4 (8)[c]	27 (54)[c]
4	3 equiv Zn	99	1	0	16	10 (20)[c]	26 (52)[c]
5	3 equiv Tetrakis(dimethylamino)ethylene (TDAE)	99	3	1	3	21 (42)[c]	15 (30)[c]

[a]Obtained by ^1H NMR spectroscopy using 1,3,5-trimethoxybenzene as an internal standard
[b]Obtained by ^{19}F NMR spectroscopy using 1-fluoronaphthalene as an internal standard
[c]Numbers based on conversion of **1a**

Figure S2: Evaluation of reductants.

Note: The above reactions were conducted according to General Procedure **A**, except for all reactions the addition of photocatalyst and *n*-propyl bromide was omitted. After all the reagents and precatalyst were added, the reaction was stirred at room temperature for 24 hours with 1500 RPM and without exposure to Kessil lamps.

3.3 Effect of Addition of Photocatalyst

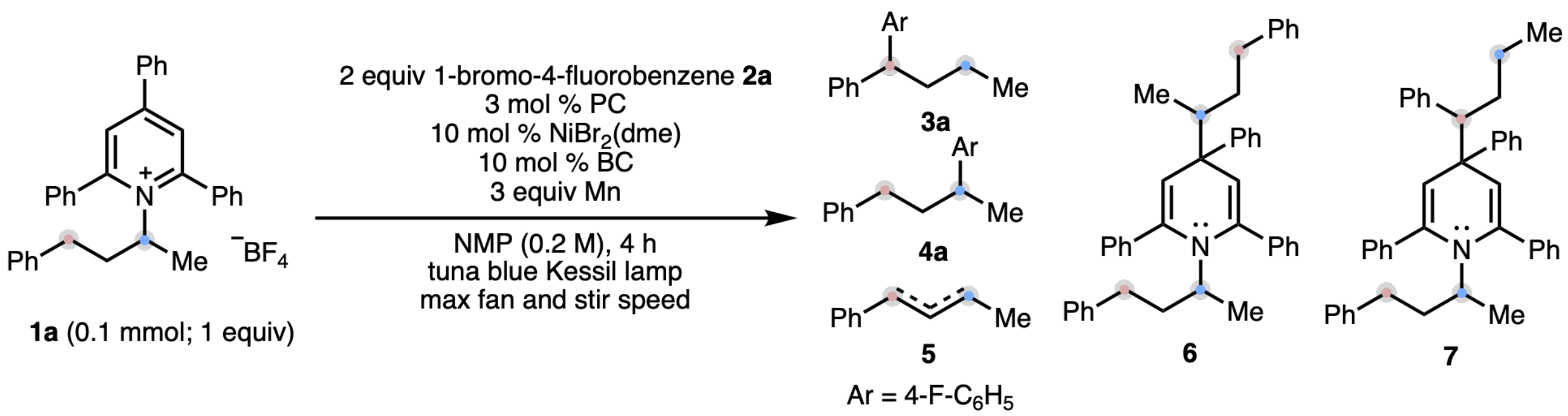

Entry	photocatalyst (PC)	1a conv.[a] (%)	3a[b] (%)	4a[b] (%)	5[a] (%)	6[a] (%)	7[a] (%)
1	None	99	4	1	2	3 (6)[c]	34 (68)[c]
2	$[Ir(ppy)_2(dtbbpy)]PF_6$	99	47	1	39	0	0
3	$[Ir\{dF(CF_3)ppy\}_2(dtbbpy)]PF_6$	99	41	1	39	0	0
4	$[Ru(bpy)_3]PF_6$	99	18	2	28	4 (8)[c]	12 (24)[c]
5	4-CzIPN	99	45	2	39	0	1

[a]Obtained by ^{1}H NMR spectroscopy using 1,3,5-trimethoxybenzene as an internal standard
[b]Obtained by ^{19}F NMR spectroscopy using 1-fluoronaphthalene as an internal standard
[c]Numbers based on conversion of **1a**

Figure S3: Effect of Addition of Photocatalyst.

Note: The above reactions were conducted according to General Procedure **A**, except for all reactions the addition of *n*-propyl bromide was omitted.

3.4 Effect of Addition of *n*-Propyl Bromide

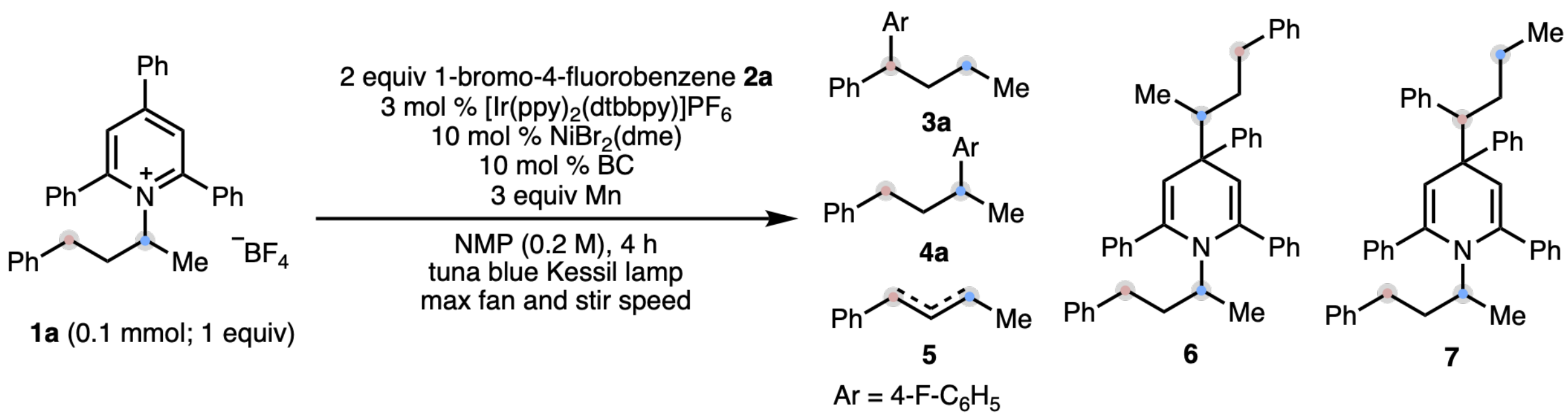

1a (0.1 mmol; 1 equiv)

Entry	deviation from above conditions	1a conv.[a] (%)	3a[b] (%)	4a[b] (%)	5[a] (%)	6[a] (%)	7[a] (%)
1	None	99	47	1	39	0	0
2	with 0.5 equiv ^{n}PrBr	99	62	3	19	0	0
3	with 1.0 equiv ^{n}PrBr	99	82	3	4	0	0

[a]Obtained by ^{1}H NMR spectroscopy using 1,3,5-trimethoxybenzene as an internal standard
[b]Obtained by ^{19}F NMR spectroscopy using 1-fluoronaphthalene as an internal standard

Figure S4: Effect of *n*-propyl bromide.

Note: The above reactions were conducted according to General Procedure **A**.

3.5 Evaluation of Solvents

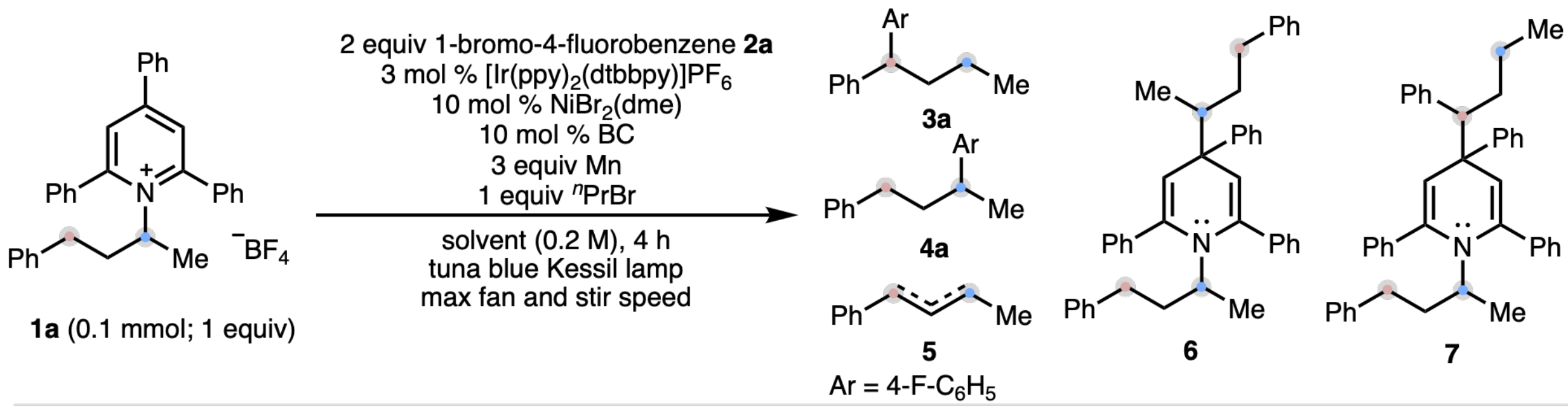

Entry	solvents	1a conv.[a] (%)	3a[b] (%)	4a[b] (%)	5[a] (%)	6[a] (%)	7[a] (%)
1	NMP	99	82	3	4	0	0
2	DMA	99	78	2	3	0	0
3	DMF	99	76	2	3	0	0
4	MeCN	57	3	1	12	2 (4)[c]	0
5	THF	98	30	3	25	1 (2)[c]	8 (16)[c]
6	PhMe	15	0	0	<1	7 (14)[c]	1 (2)[c]

[a]Obtained by ^{1}H NMR spectroscopy using 1,3,5-trimethoxybenzene as an internal standard
[b]Obtained by ^{19}F NMR spectroscopy using 1-fluoronaphthalene as an internal standard
[c]Numbers based on conversion of **1a**

Figure S5: Evaluation of solvents.

Note: The above reactions were conducted according to General Procedure **A**. For each reaction, its individual precatalyst solution was prepared in the corresponding solvent as described in General Procedure **A**.

3.6 Evaluation of Ligands

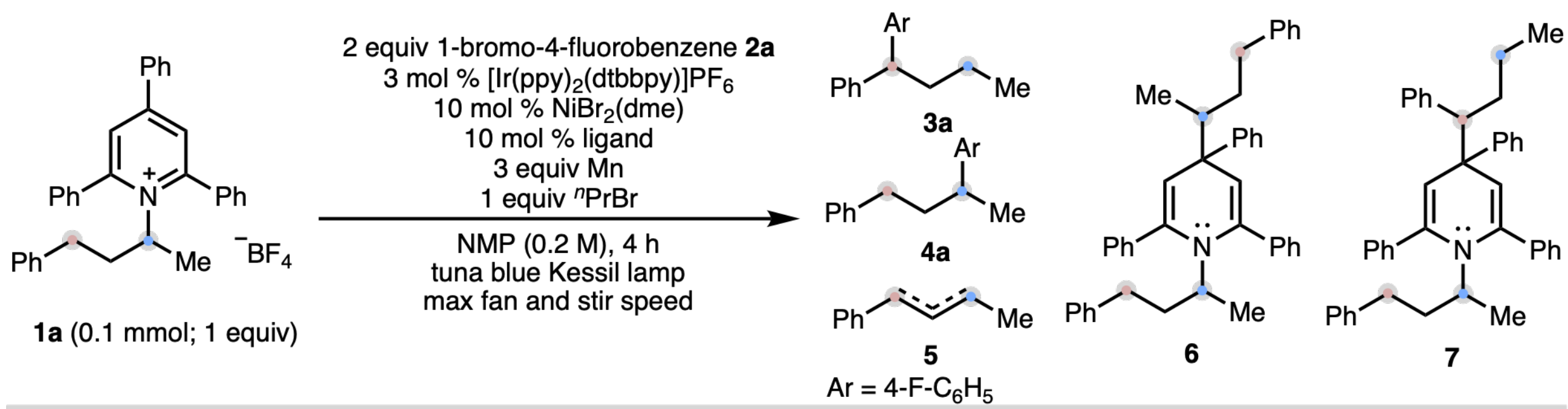

Entry	deviation from above conditions	1a conv.[a] (%)	3a[b] (%)	4a[b] (%)	5[a] (%)	6[a] (%)	7[a] (%)
1	None	99	82	3	4	0	0
2	6,6′-Me_2bpy instead of BC	99	60	1	9	0	0
3	^{n}BuBP instead of BC	99	26	<1	21	0	0
4[c]	6, 6’-^{n}HexBP instead of BC	99	39	<1	<1	0	0
5	BP instead of BC	99	1	61	8	0	0
6	dtbbpy instead of BC	99	2	77	2	0	0

[a]Obtained by ^{1}H NMR spectroscopy using 1,3,5-trimethoxybenzene as an internal standard
[b]Obtained by ^{19}F NMR spectroscopy using 1-fluoronaphthalene as an internal standard
[c]*n*-Butylbenzene was observed as the major side product in this reaction

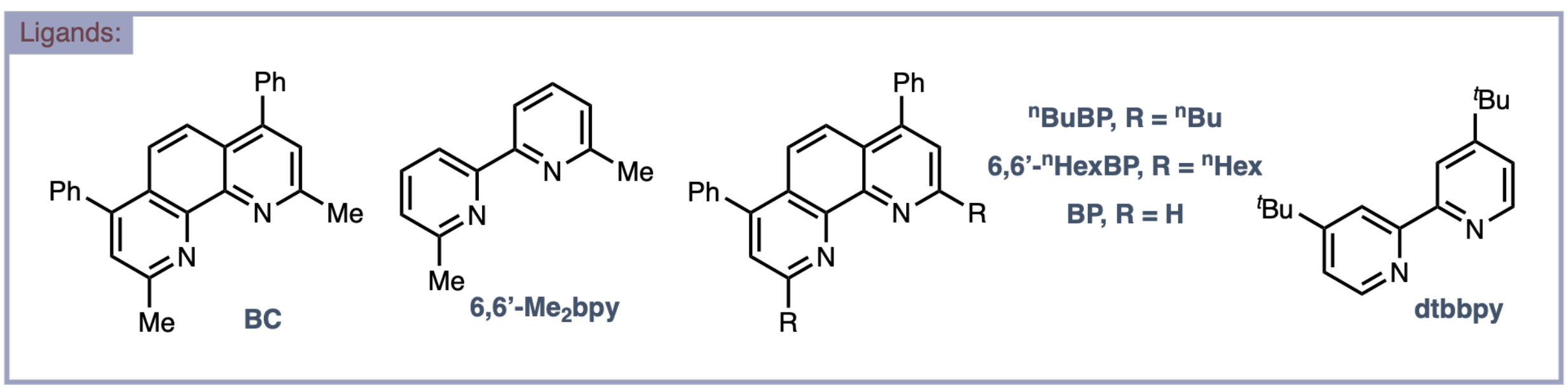

Figure S6: Evaluation of ligands.

Note: The above reactions were conducted according to General Procedure **A**. For each reaction, its individual precatalyst solution was prepared using the corresponding ligand (10 mol %) as described in General Procedure **A**.

3.7 Control Studies

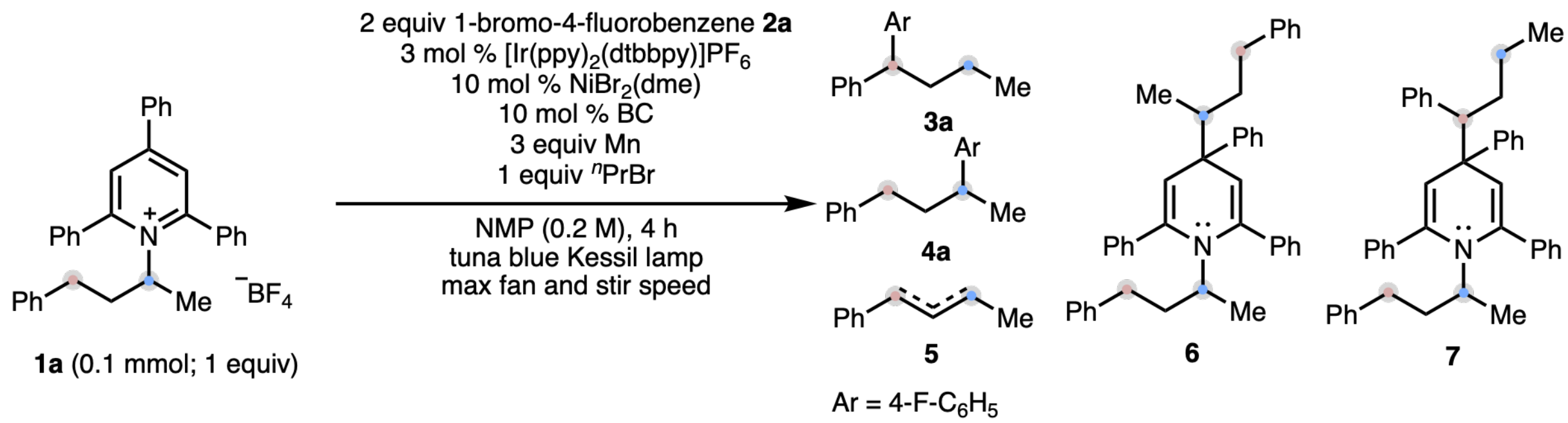

Entry	deviation from above conditions	1a conv.[a] (%)	3a[b] (%)	4a[b] (%)	5[a] (%)	6[a] (%)	7[a] (%)
1	None	99	82	3	4	0	0
2	No Ir PC	99	22	3	15	4 (8)[c]	28 (56)[c]
3	No Ir PC, 8 h	99	33	3	3	8 (16)[c]	20 (40)[c]
4[d]	No Ni/BC	99	0	0	0	0	0
5	No Mn	38	0	0	27	0	0
6	No light (dark)	93	20	2	8	5 (10)[c]	23 (46)[c]
7	[Ir{dF(CF$_3$)ppy}$_2$(dtbbpy)]PF$_6$ as PC	99	73	2	6	0	0
8	4-CzIPN as PC	99	66	1	0	0	0
9	No BC	99	14	13	0	0	0
10	Et$_3$N in place of Mn	93	24	2	33	<1	<1
11	^{i}Pr$_2$NH in place of Mn	99	39	1	6	0	0
12	tetramethylpiperidine in place of Mn	36	9	1	1	0	0
13	Hantzsch ester in place of Mn	95	0	1	6	0	0
14	Tetrakis(dimethylamino)ethylene in place of Mn	99	9	1	1	0	0
15[d]	1.0 equiv 1-bromo-4-fluorobenzene **2a**	99	45	1	0	0	0
16[d]	1.5 equiv 1-bromo-4-fluorobenzene **2a**	99	65	1	0	0	0

[a]Obtained by ^{1}H NMR spectroscopy using 1,3,5-trimethoxybenzene as an internal standard
[b]Obtained by ^{19}F NMR spectroscopy using 1-fluoronaphthalene as an internal standard
[c]Numbers based on conversion of **1a**
[d]*n*-Butylbenzene was observed as the major side product in this reaction

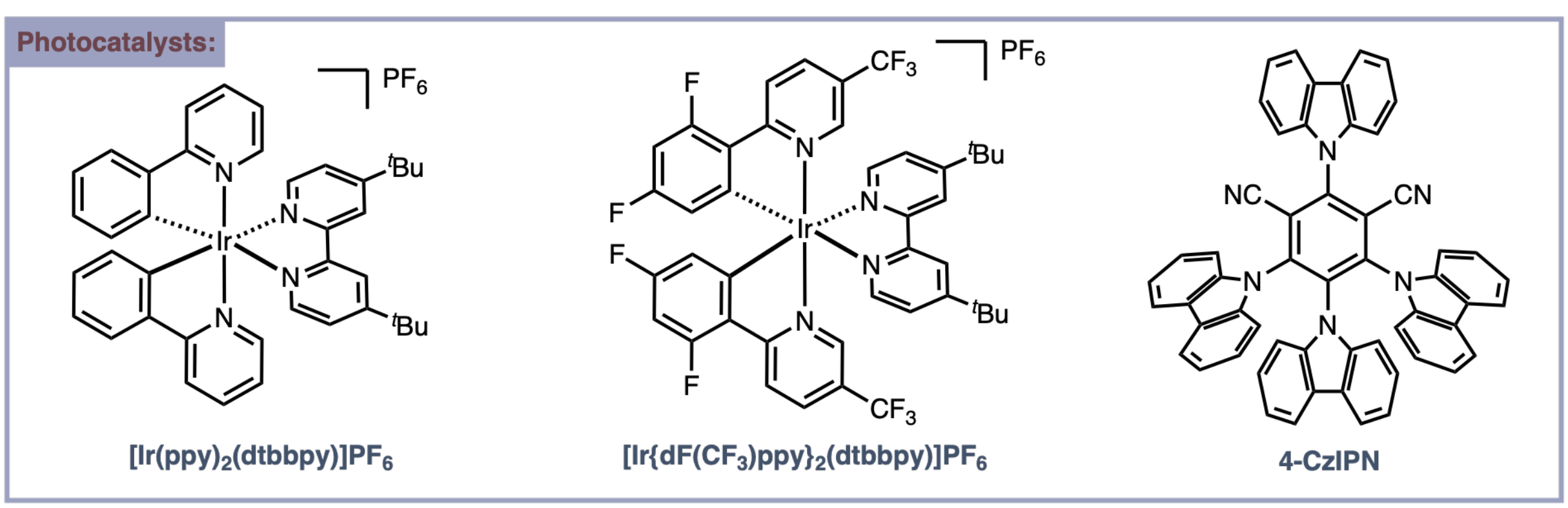

Figure S7: Control experiments.

Note: The above reactions were conducted according to General Procedure **A**.

- For entries 2-3, the reaction was conducted according to General Procedure **A** except Ir photocatalyst was omitted.
- For entry 4, the reaction was conducted according to General Procedure **A** except precatalyst solution was omitted.
- For entry 5, the reaction was conducted according to General Procedure **A** except Mn powder was omitted.
- For entry 6, the reaction was conducted according to General Procedure **A**, except after the addition of all reagents, the reaction was covered with aluminum foil and stirred at 35 °C at 1500 rpm on IKA plate without the blue lights.
- For entries 7-8, the reaction was conducted according to General Procedure **A** using the corresponding photocatalyst instead of $[Ir(ppy)_2(dtbbpy)]PF_6$.
- For entry 9, the reaction was conducted according to General Procedure **A** without bathocuproine.
- For entries 10-14, the reaction was conducted according to General Procedure **A** using the stated homogeneous reductant in place of Mn.

3.8 Evaluations of aryl halide coupling partner

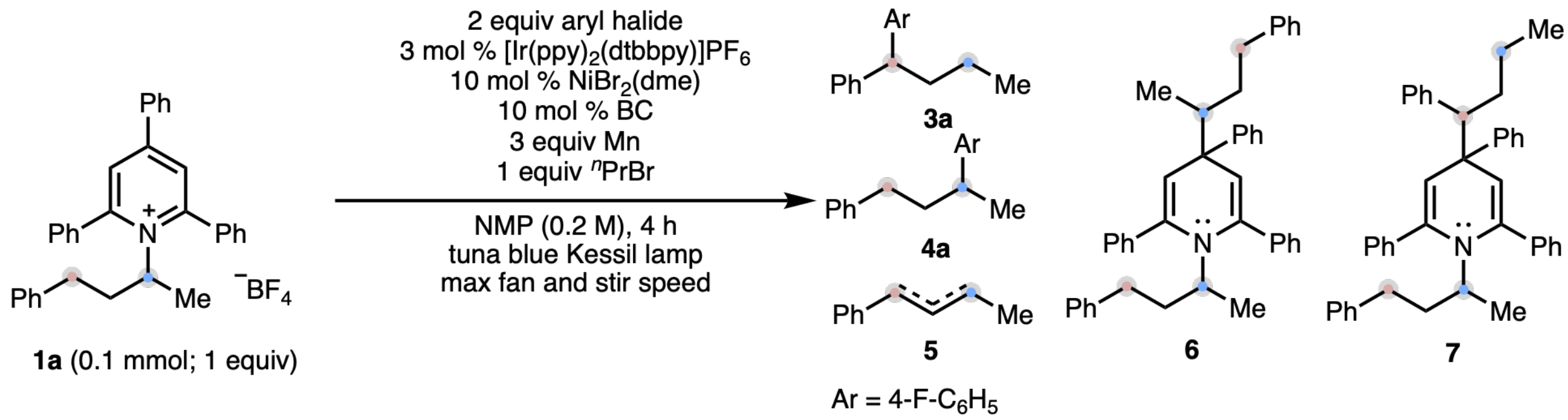

Entry	aryl halide	1a conv.[a] (%)	3a[b] (%)	4a[b] (%)	5[a] (%)	6[a] (%)	7[a] (%)
1[d]	1-fluoro-4-iodobenzene	99	9	12	10	9 (18)[c]	9 (18)[c]
2[d]	1-chloro-4-fluorobenzene	99	2	<1	20	0	5 (10)[c]

[a]Obtained by ^{1}H NMR spectroscopy using 1,3,5-trimethoxybenzene as an internal standard
[b]Obtained by ^{19}F NMR spectroscopy using 1-fluoronaphthalene as an internal standard
[c]Numbers based on conversion of **1a**
[d]*n*-Butylbenzene was observed as the major side product in this reaction

Note: The above reactions were conducted according to General Procedure **A** with the corresponding aryl iodide or chloride in place of aryl bromide.

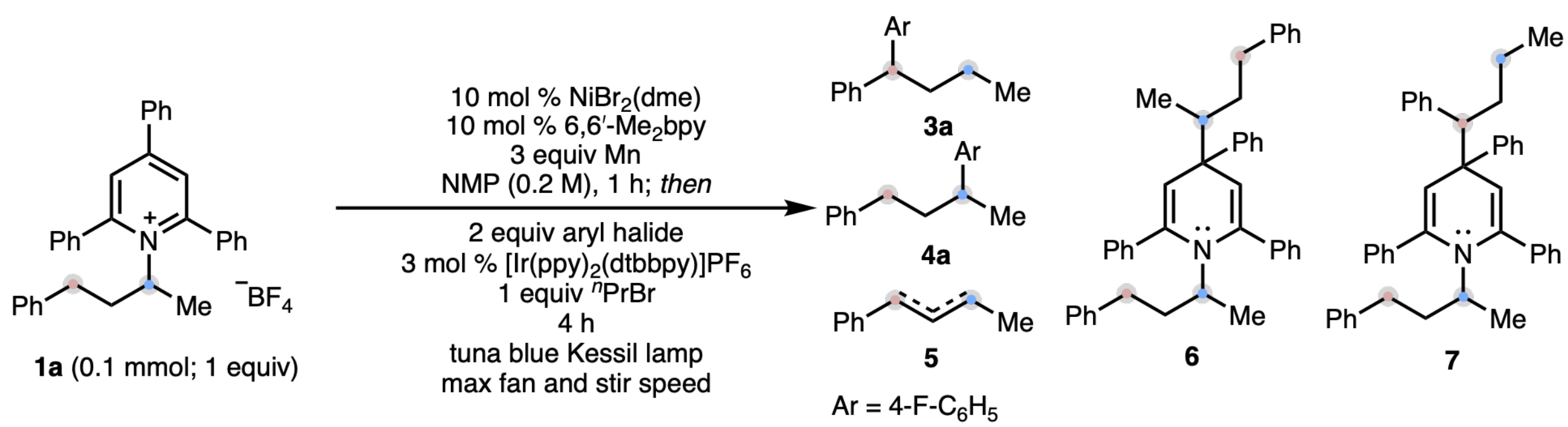

Entry	aryl halide	1a conv.[a] (%)	3a[b] (%)	4a[b] (%)	5[a] (%)	6[a] (%)	7[a] (%)
1[d]	1-fluoro-4-iodobenzene	99	30	4	20	0	6 (12)[c]
2[d]	1-chloro-4-fluorobenzene	99	2	0	46	0	7 (14)[c]

[a]Obtained by ^{1}H NMR spectroscopy using 1,3,5-trimethoxybenzene as an internal standard
[b]Obtained by ^{19}F NMR spectroscopy using 1-fluoronaphthalene as an internal standard
[c]Numbers based on conversion of **1a**
[d]*n*-Butylbenzene was observed as the major side product in this reaction

Note: The above reactions were conducted according to *Method B* described below in *General Procedure 1* with the corresponding aryl iodide or chloride in place of aryl bromide.

4. Synthesis of Substrates

4.1 Preparation of Katritzky Salts

The following Katritzky salts have been previously reported[2–5] and were prepared accordingly:

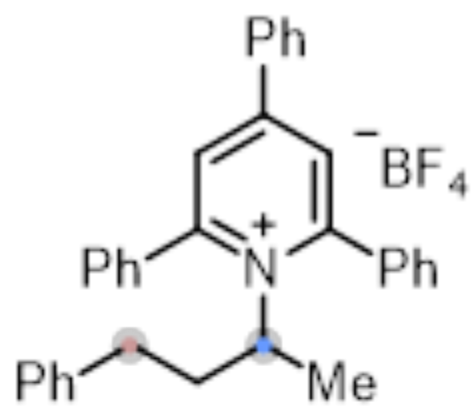

1a

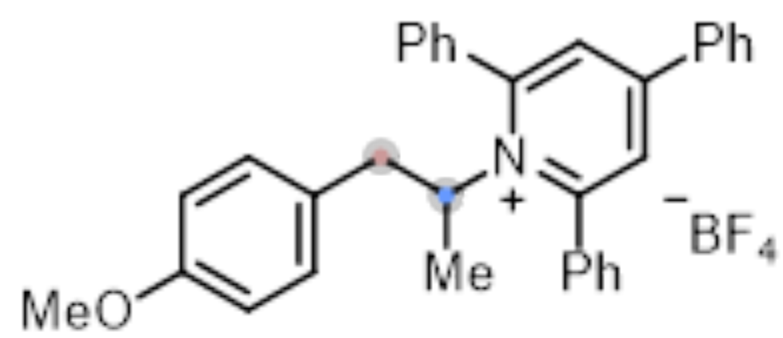

1u

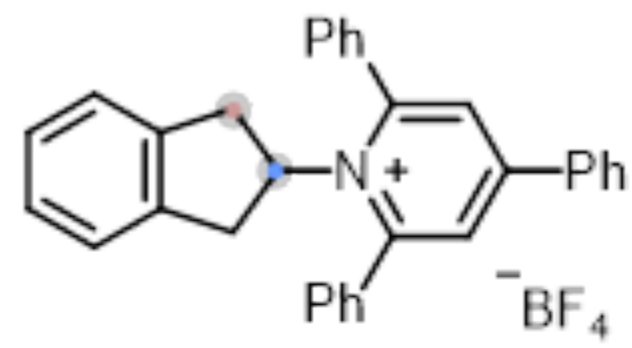

1af

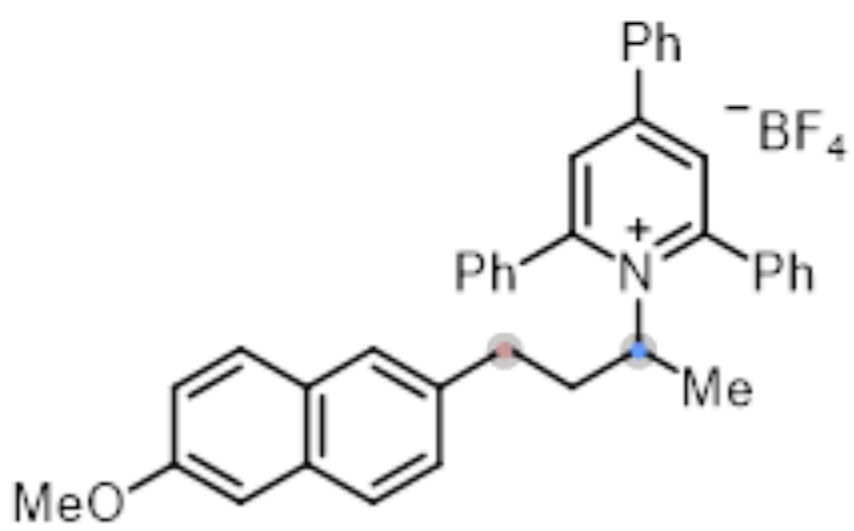

1at

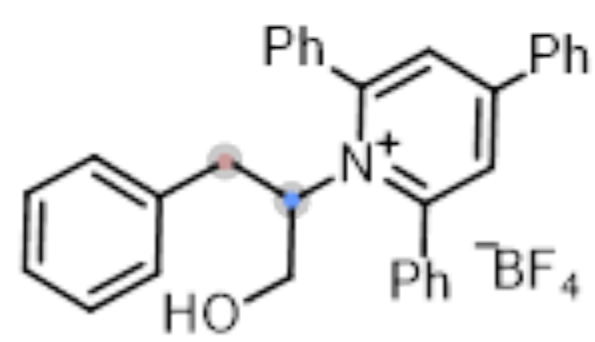

1av

General Procedure A:

This procedure was adapted from a known procedure reported by Watson and coworkers.[6] A round-bottom flask equipped with a PTFE-coated magnetic stir bar was charged with 2,4,6-Triphenylpyrylium tetrafluoroborate (1.00 equiv) followed by DCM (0.5 M), the corresponding amine (1.2 equiv), and Et_3N (1.00 equiv). The resulting mixture was stirred at room temperature for 30 minutes. Acetic acid (1.00 equiv) was then added and the mixture was stirred at room temperature for an additional 16 h. After the reaction was complete, the reaction mixture was further diluted with additional DCM and washed with HCl (1.0 M), sat. $NaHCO_3$ (aq.), and brine. The organic layer was dried over Na_2SO_4, filtered, and concentrated *in vacuo*. The crude mixture was then suspended in Et_2O and stirred for 1 h. The resulting solid was filtered, washed with Et_2O, and dried on Schlenk line before bringing into the glovebox for further use.

(Note: If diethyl ether was still present after prolonged drying, the Katritzky salt was suspended in pentane, vigorously stirred, and concentrated *in vacuo*. This process was

repeated multiple times to remove diethyl ether as much as possible. In some cases, trace diethyl ether still remained, but we observed no deleterious effect on the reaction.)

General Procedure B:

A round-bottom flask equipped with a PTFE-coated magnetic stir bar was charged with the respective acridinium salt (1.00 equiv) followed by EtOH (1.0 M). To this suspension, the corresponding amine (1.20 equiv) was added and refluxed for 4 h. The resulting mixture was then cooled to room temperature. If precipitation occurred, the precipitate was filtered, and washed with Et_2O. If no precipitation occurred, Et_2O was added, and the crude mixture was stirred for 1 h. The resulting solid was then filtered, washed further with Et_2O, and dried on Schlenk line before bringing into the glovebox for further use.

(Note: If diethyl ether was still present after prolonged drying, the Katritzky salt was suspended in pentane, vigorously stirred, and concentrated *in vacuo*. This process was repeated multiple times to remove diethyl ether as much as possible. In some cases, trace diethyl ether still remained, but we observed no deleterious effect on the reaction.)

General Procedure C:

A 100 mL round-bottom flask equipped with a PTFE-coated magnetic stir bar was charged with the acid (1.00 equiv) followed by DCM (0.1 M). Then, *N,O*-dimethylhydroxylamine hydrochloride (1.50 equiv), 1-ethyl-3-(3-dimethylaminopropyl)carbodiimide hydrochloride (1.50 equiv) were added sequentially and allowed to stir for 12 hours. After TLC analysis indicated the complete consumption of starting material, the reaction mixture was transferred to a separatory funnel and washed with HCl (1 M aq.) and sat. $NaHCO_3$ (aq.), dried with Na_2SO_4, filtered, and concentrated *in vacuo* to obtain the corresponding amide. The crude amide was used directly in the subsequent reaction without further purification.

A flame-dried 100 mL round-bottom flask equipped with a PTFE-coated magnetic stir bar was charged with the crude amide intermediate followed by anhydrous THF (0.1 M). The resulting solution was sparged with N_2 for 15 minutes. Then, the reaction was cooled to -78 ˚C in a dry ice/acetone bath and methylmagnesium bromide (3.00 M in THF, 1.50 equiv) was added dropwise. The resulting mixture was allowed to warm to 0 ^oC over one hour. Then, water (1 mL) was added dropwise to quench any remaining Grignard followed by sat. NH_4Cl (25 mL). The biphasic mixture was poured into a separatory funnel and the organic layer was separated. The aqueous layer was extracted with ethyl acetate (2 x 50 mL). The organic layers were combined, washed with brine, dried over Na_2SO_4, filtered, and concentrated *in vacuo* to obtain the corresponding ketone. The crude ketone was used directly in the subsequent reaction without further purification.

A flame-dried 100 mL round-bottom flask equipped with a PTFE-coated magnetic stir bar was charged with the ketone intermediate followed by MeOH (0.1 M). To the solution, ammonium acetate (10.0 equiv) was added and allowed to stir for 30 minutes. Then, $NaCNBH_3$ (5.00 equiv) was added portion wise, and the reaction mixture was allowed to stir for 12 hours. After the complete consumption of ketone, the reaction mixture was concentrated *in vacuo*. The crude material was redissolved in DCM (50 mL) and transferred to a separatory funnel. HCl (aq.) (50 mL) was added to the separatory funnel, shaken, and the organic layer was separated. The aqueous layer was further extracted with DCM (2 x 50 mL). The organic layers were then combined, washed with brine, dried over Na_2SO_4, filtered, and concentrated *in vacuo* to obtain the corresponding amine, which was used directly in the subsequent pyridinium synthesis without further purification.

This procedure was adapted from a known procedure reported by Watson and coworkers.[6] A round-bottom flask equipped with a PTFE-coated magnetic stir bar was charged with 2,4,6-Triphenylpyrylium tetrafluoroborate (1.00 equiv) followed by DCM (0.5 M), the corresponding amine (1.2 equiv), and Et_3N (1.00 equiv). The resulting mixture was stirred at room temperature for 30 minutes. Acetic acid (1.00 equiv) was then added and the mixture was stirred at room temperature for an additional 16 h. After the reaction

was complete, the reaction mixture was further diluted with additional DCM and washed with HCl (1.0 M), sat. $NaHCO_3$ (aq.), and brine. The organic layer was dried over Na_2SO_4, filtered, and concentrated *in vacuo*. The crude mixture was then suspended in Et_2O and stirred for 1 h. The resulting solid was filtered, washed with Et_2O, and dried on Schlenk line before bringing into the glovebox for further use.

(Note: If diethyl ether was still present after prolonged drying, the Katritzky salt was suspended in pentane, vigorously stirred, and concentrated *in vacuo*. This process was repeated multiple times to remove diethyl ether as much as possible. In some cases, trace diethyl ether still remained, but we observed no deleterious effect on the reaction.)

General Procedure D:

A round-bottom flask equipped with a PTFE-coated magnetic stir bar was charged with carboxylic acid (1.00 equiv) followed by anhydrous THF (0.05 M). To this solution, triethylamine (2.00 equiv) was added and the resulting mixture was cooled to 0 °C. To this solution, Yamaguchi's reagent (2,4,6-Trichlorobenzoyl chloride) (1.00 equiv) was added. The reaction mixture was stirred at 0 °C for one hour. A solution of **1y** (1.00 equiv) in THF was added, followed by a solution of DMAP (4.00 mol %) in THF. The reaction mixture was slowly allowed to warm to room temperature and stirred further for an additional 18 hours. Then, the reaction mixture was transferred to a separatory funnel and washed with sat. $NaHCO_3$ (aq.). The aqueous layer was separated and extracted with ethyl acetate. The combined organic layers were then washed with brine, dried over $MgSO_4$, filtered, and concentrated *in vacuo*. The crude material was then purified by flash column chromatography using DCM/MeOH was an eluent.

4.2 Characterization of Unknown Katritzky Salts

1-(4-(4-Methoxyphenyl)butan-2-yl)-2,4,6-triphenylpyridin-1-ium tetrafluoroborate **1v**:

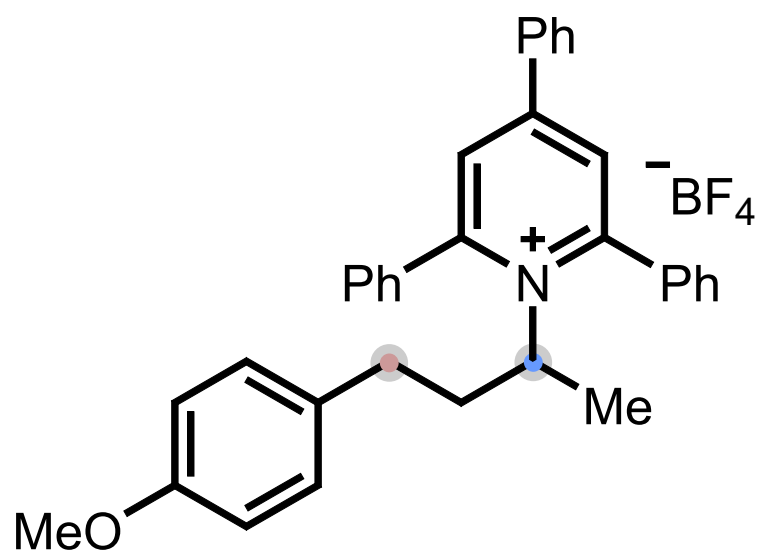

1v

Synthesized according to General Procedure A described in section 4.1 using 2,4,6-Triphenylpyrylium tetrafluoroborate (1.98 g, 5.00 mmol, 1.00 equiv) and 4-(4-methoxyphenyl)butan-2-amine (1.08 g, 6.00 mmol, 1.20 equiv) to yield **1v** as an orange solid in 83% yield (2.29 g).

^{1}H NMR (300 MHz, $CDCl_3$): δ 7.83 (s, 2H), 7.80 – 7.67 (m, 4H), 7.63 – 7.34 (m, 11H), 6.79 (d, *J* = 8.4 Hz, 2H), 6.70 (d, *J* = 8.4 Hz, 2H), 4.96 – 4.85 (m, 1H), 3.79 (s, 3H), 2.36 (dd, *J* = 12.7, 7.5 Hz, 1H), 2.17 – 2.04 (m, 2H), 1.79 – 1.70 (m, 1H), 1.47 (d, *J* = 6.9 Hz, 3H).

^{13}C NMR (101 MHz, $CDCl_3$): δ 158.3, 157.4 (br), 155.3, 134.1, 133.9, 132.1, 130.92, 130.88, 129.7 (br), 129.1 (br), 128.4, 114.3, 66.0, 55.4, 38.2, 31.7, 21.7 (3 aromatic signals are not observed due to signal broadening).

^{19}F NMR (282 MHz, $CDCl_3$): δ -153.19, -153.24.

^{11}B NMR (96 MHz, $CDCl_3$): δ -1.28.

HRMS (ESI): *m/z* calculated for $C_{34}H_{32}NO^+$ ([M - BF_4]$^+$): 470.2478; found 470.2471.

2,4,6-Triphenyl-1-(1-phenylheptan-3-yl)pyridin-1-ium tetrafluoroborate **1w:**

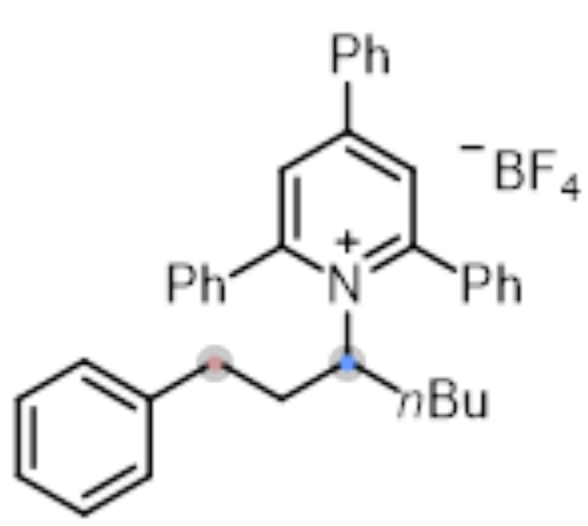

1w

Synthesized according to General Procedure A described in section 4.1 using 2,4,6-Triphenylpyrylium tetrafluoroborate (356 mg, 0.900 mmol, 1.00 equiv) and 1-phenylheptan-3-amine (207 mg, 1.100 mmol, 1.20 equiv) to yield **1w** as a pale yellow solid in 44% yield (220 mg).

^{1}H NMR (500 MHz, $CDCl_3$): δ 7.94 (br s, 1H), 7.87 – 7.80 (m, 4H), 7.76 – 7.64 (m, 2H), 7.59 (t, *J* = 6.9 Hz, 4H), 7.57 – 7.48 (m, 6H), 7.26 – 7.13 (m, 3H), 6.94 (d, *J* = 7.1 Hz, 2H),

4.75 (p, J = 7.1 Hz, 1H), 2.48 – 2.37 (m, 1H), 2.37 – 2.26 (m, 2H), 1.98 – 1.88 (m, 1H), 1.88 – 1.77 (m, 1H), 1.53 – 1.40 (m, 1H), 1.14 (hept, J = 6.7 Hz, 2H), 1.03 – 0.91 (m, 1H), 0.91 – 0.81 (m, 2H), 0.78 (t, J = 7.3 Hz, 3H).

^{13}C NMR (126 MHz, $CDCl_3$): δ 158.4, 157.0, 155.4, 139.3, 133.9, 132.3, 131.2 (br), 129.8, 128.9, 128.5 (br), 128.2 (br), 126.7, 71.8, 36.3, 35.1, 33.0, 29.0, 22.2, 13.9. (3 aromatic signals are not observed due to signal broadening).

^{19}F NMR (282 MHz, $CDCl_3$): δ -153.11.

^{11}B NMR (96 MHz, $CDCl_3$): δ -1.27.

HRMS (ESI): *m/z* calculated for $C_{36}H_{36}N^+$ ($[M - BF_4]^+$): 482.2843; found 482.2876.

2,4,6-Triphenyl-1-(5-phenylpentan-2-yl)pyridin-1-ium tetrafluoroborate **1x**:

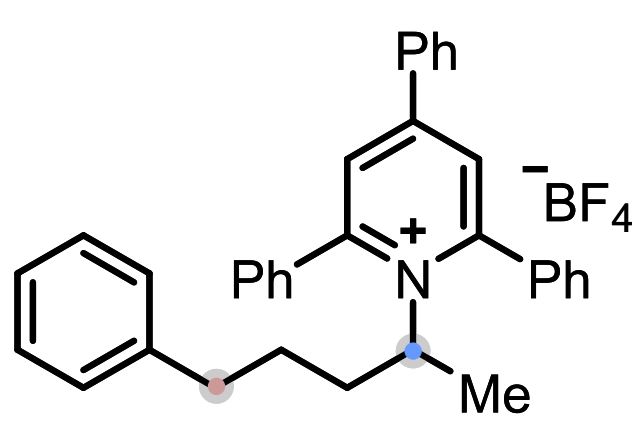

1x

Synthesized according to General Procedure A described in section 4.1 using 2,4,6-Triphenylpyrylium tetrafluoroborate (2.26 g, 5.70 mmol, 1.00 equiv) and 5-phenylpentan-2-amine (1.12 g, 6.84 mmol, 1.20 equiv) to yield **1x** as a yellow solid in 25% yield (744 mg).

^{1}H NMR (500 MHz, $(CD_3)_2CO$): δ 8.33 (br, 2H), 8.18 (d, J = 7.5 Hz, 2H), 7.83 (br, 2H), 7.75 – 7.50 (m, 11H), 7.26 (t, J = 7.4 Hz, 2H), 7.19 (t, J = 7.3 Hz, 1H), 7.07 (d, J = 7.2 Hz, 2H), 5.08 (sept, J = 7.0 Hz, 1H), 2.44 (ddt, J = 44.8, 13.8, 7.2 Hz, 2H), 1.97 – 1.86 (m, 1H), 1.61 – 1.53 (m, 1H), 1.51 (d, J = 7.0 Hz, 3H), 1.43 (qd, J = 12.2, 6.0 Hz, 1H), 1.32 – 1.16 (m, 1H).

^{13}C NMR (126 MHz, $(CD_3)_2CO$): δ 155.3, 142.2, 141.1, 135.0 (br), 134.4, 133.3, 131.8, 130.1, 129.7 (br), 129.4, 129.3, 129.2, 126.8, 68.4, 36.9, 35.6, 29.0, 22.6 (2 aromatic signals are not observed due to signal broadening).

^{19}F NMR (282 MHz, $(CD_3)_2CO$): δ -151.67, -151.73.

^{11}B NMR (96 MHz, $(CD_3)_2CO$): δ -0.92.

HRMS (ESI): *m/z* calculated for $C_{34}H_{32}N^+$ ($[M - BF_4]^+$): 454.2530; found 454.2523.

1-(4-(4-Hydroxyphenyl)butan-2-yl)-2,4,6-triphenylpyridin-1-ium tetrafluoroborate **1y**:

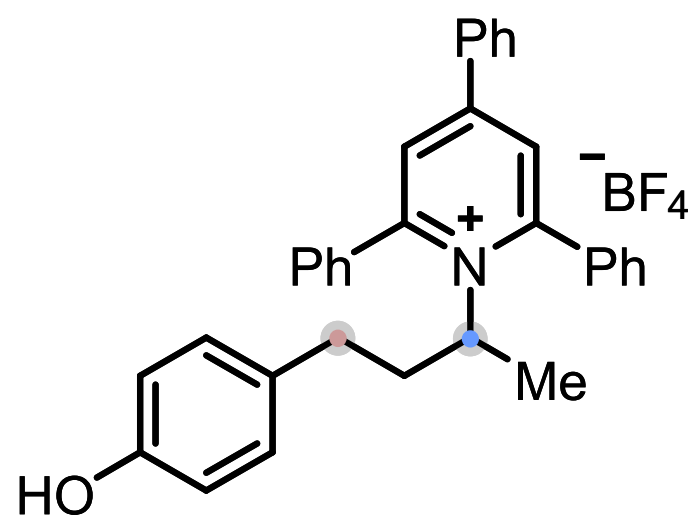

1y

Synthesized according to General Procedure A described in section 4.1 using 2,4,6-Triphenylpyrylium tetrafluoroborate (3.96 g, 10.0 mmol, 1.00 equiv) and 4-(3-aminobutyl)phenol (1.98 g, 12.0 mmol, 1.20 equiv) to yield **1y** as a white solid in 87% yield (4.70 g).

^{1}H NMR (300 MHz, $CDCl_3$): δ 7.79 (br s, 2H), 7.75 (d, *J* = 7.8 Hz, 2H), 7.66 (br s, 2H), 7.60 – 7.47 (m, 12H), 6.68 (d, *J* = 8.7 Hz, 2H), 6.6 (d, *J* = 8.7 H,z 2H), 6.37 (br s, 1H), 4.99 – 4.83 (m, 1H), 2.37 – 2.29 (m, 1H), 2.10 – 2.01 (m, 2H), 1.73 – 1.65 (m, 1H), 1.46 (d, *J* = 7.0 Hz, 3H).

^{13}C NMR (126 MHz, $CDCl_3$): δ 157.7 (br), 155.34, 155.28, 133.9, 133.6 (br), 132.2, 131.2, 129.8, 129.7 (br), 129.0, 128.4, 127.3 (br), 116.1, 65.9, 38.2, 31.5, 22.0 (2 aromatic signals are not observed due to signal broadening).

^{19}F NMR (282 MHz, $CDCl_3$): δ -152.83, -152.88.

^{11}B NMR (96 MHz, $CDCl_3$): δ -1.22.

HRMS (ESI): *m/z* calculated for $C_{33}H_{30}NO^+$ ($[M - BF_4]^+$): 456.2322; found 456.2326.

1-(1-(4-Chlorophenyl)propan-2-yl)-2,4,6-triphenylpyridin-1-ium tetrafluoroborate **1z**:

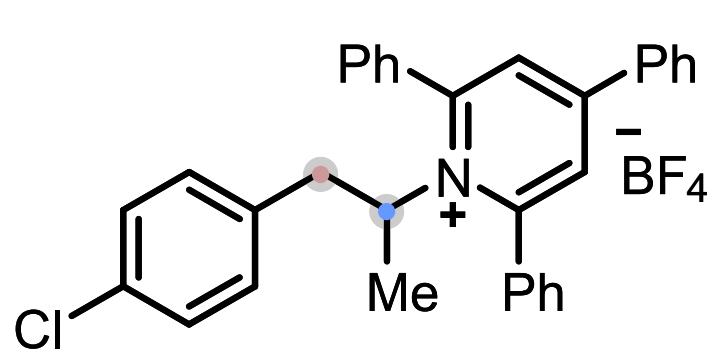

1z

Synthesized according to General Procedure A described in section 4.1 using 2,4,6-Triphenylpyrylium tetrafluoroborate (990 mg, 2.50 mmol, 1.00 equiv) and 1-(4-chlorophenyl)propan-2-amine (507 mg, 3.00 mmol, 1.20 equiv) to yield **1z** as a white solid in 68% yield (931 mg).

^{1}H NMR (300 MHz, $CDCl_3$): δ 7.83 – 7.73 (m, 8H), 7.65 – 7.44 (m, 9H), 7.07 (d, *J* = 8.0 Hz, 2H), 6.56 (d, *J* = 8.0 Hz, 2H), 5.15 – 5.04 (m, 1H), 3.45 – 3.37 (dd, *J* = 8.4 Hz, 4.3 Hz, 1H), 2.51 (dd, *J* = 13.3, 10.0 Hz, 1H), 1.31 (d, *J* = 7.3 Hz, 3H).

^{13}C NMR (126 MHz, $CDCl_3$): δ 157.2, 155.6, 135.1, 134.1, 134.0, 133.3, 132.2, 131.2, 130.2, 129.7, 129.0 (br), 128.5, 67.8, 42.3, 21.1 (3 aromatic signals are not observed due to signal broadening).

^{19}F NMR (282 MHz, $CDCl_3$): δ -152.97, -153.03.

^{11}B NMR (96 MHz, $CDCl_3$): δ -1.29.

HRMS (ESI): *m/z* calculated for $C_{32}H_{27}ClN^+$ ($[M - BF_4]^+$): 460.1827; found 460.1835.

1-(1-(4-Fluorophenyl)propan-2-yl)-2,4,6-triphenylpyridin-1-ium tetrafluoroborate **1aa**:

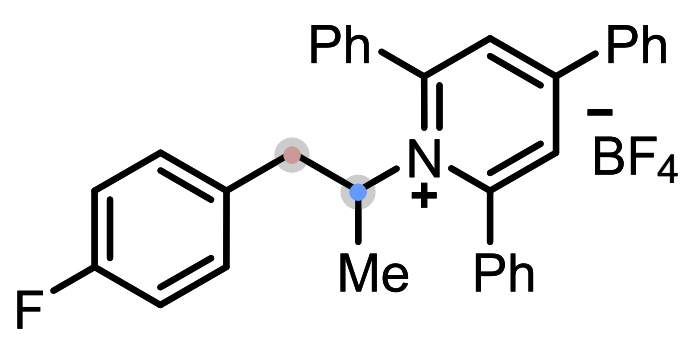

1aa

Synthesized according to General Procedure A described in section 4.1 using 2,4,6-Triphenylpyrylium tetrafluoroborate (2.06 g, 5.20 mmol, 1.00 equiv) and 1-(4-fluorophenyl)propan-2-amine (955 mg, 6.24 mmol, 1.20 equiv) to yield **1aa** as a white solid in 79% yield (2.18 g).

^{1}H NMR (300 MHz, $CDCl_3$): δ 7.84 – 7.74 (m, 8H), 7.65 – 7.56 (m, 6H), 7.54 – 7.46 (m, 3H), 6.79 (t, *J* = 8.4 Hz, 2H), 6.59 (dd, *J* = 8.4, 5.5 Hz, 2H), 5.16 – 5.04 (m, 1H), 3.42 (dd, *J* = 13.5, 4.2 Hz, 1H), 2.53 (dd, *J* = 13.5, 9.8 Hz, 1H), 1.32 (d, *J* = 7.0 Hz, 3H).

^{13}C NMR (126 MHz, $CDCl_3$): δ 162.1 (d, *J* = 246.3 Hz), 157.3, 155.5, 134.1, 134.0, 132.3 (d, *J* = 3.3 Hz), 132.2, 131.2 (br), 130.4 (d, *J* = 8.0 Hz), 129.8, 129.0 (br), 128.4, 115.8 (d, *J* = 21.4 Hz), 68.1, 42.2, 21.1 (2 aromatic signals are not observed due to signal broadening).

^{19}F NMR (282 MHz, $CDCl_3$): δ -114.94, -152.99, -153.03.

^{11}B NMR (96 MHz, $CDCl_3$): δ -1.30.

HRMS (ESI): *m/z* calculated for $C_{32}H_{27}FN^+$ ([M - BF_4]$^+$): 444.2122; found 444.2129.

2,4,6-Triphenyl-1-(1-(4-(trifluoromethyl)phenyl)propan-2-yl)pyridin-1-ium tetrafluoroborate **1ab**:

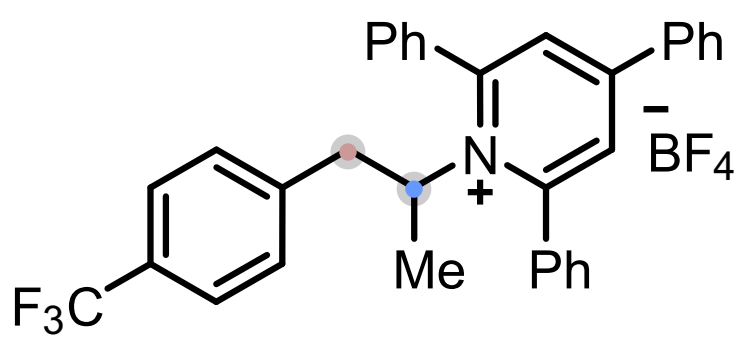

1ab

Synthesized according to General Procedure A described in section 4.1 using 2,4,6-Triphenylpyrylium tetrafluoroborate (206 mg, 0.52 mmol, 1.00 equiv) and 1-(4-(trifluoromethyl)phenyl)propan-2-amine (127 mg, 0.62 mmol, 1.20 equiv) to yield **1ab** as a yellow solid in 62% yield (160 mg).

^{1}H NMR (300 MHz, $CDCl_3$): δ 7.87 (s, 2H), 7.78 (d, *J* = 7.3 Hz, 5H), 7.68 – 7.59 (m, 6H), 7.54 (d, *J* = 7.3 Hz, 2H), 7.50 (t, *J* = 7.3 Hz, 2H), 7.36 (d, *J* = 8.3 Hz, 2H), 6.75 (d, *J* = 8.3 Hz, 2H), 5.21 – 5.10 (m, 1H), 3.59 (dd, *J* = 13.4, 4.1 Hz, 1H), 2.60 (dd, *J* = 13.4, 10.2 Hz, 1H), 1.32 (d, *J* = 7.2 Hz, 3H).

^{13}C NMR (126 MHz, $CDCl_3$): δ 157.2 (br), 155.6, 140.8, 134.0, 133.9, 132.2, 131.2, 129.7, 129.6 (q, *J* = 32.8 Hz) 129.3 (br), 128.9, 128.4, 127.5, 125.8 (q, *J* = 3.8 Hz), 124.0 (q, *J* = 272.0 Hz), 117.6, 67.4, 42.6, 21.0.

^{19}F NMR (282 MHz, $CDCl_3$): δ -62.63, -152.81, -152.87.

^{11}B NMR (96 MHz, $CDCl_3$): δ -1.28.

HRMS (ESI): *m/z* calculated for $C_{33}H_{27}F_3N^+$ ([M - BF_4]$^+$): 494.2091; found 494.2092.

1-(1-(3-Methoxyphenyl)propan-2-yl)-2,4,6-triphenylpyridin-1-ium tetrafluoroborate **1ac**:

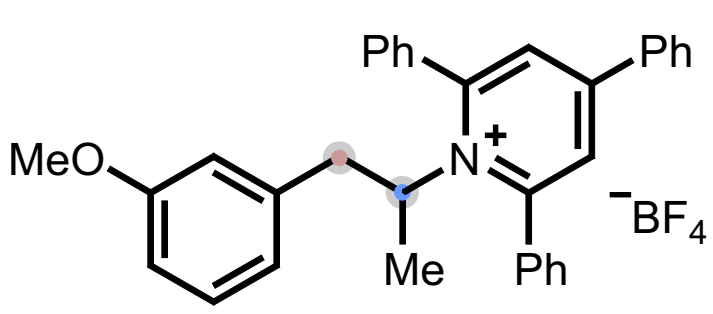

1ac

Synthesized according to General Procedure A described in section 4.1 using 2,4,6-Triphenylpyrylium tetrafluoroborate (1.09 g, 2.75 mmol, 1.00 equiv) and 1-(3-methoxyphenyl)propan-2-amine (545 mg, 3.30 mmol, 1.20 equiv) to yield **1ac** as a yellow solid in 75% yield (1.12 g).

^{1}H NMR (300 MHz, $CDCl_3$): δ 7.85 – 7.76 (m, 6H), 7.64 – 7.43 (m, 11H), 7.02 (t, *J* = 7.9 Hz, 1H), 6.69 (dd, *J* = 7.9, 2.5 Hz, 1H), 6.19 (d, *J* = 7.5 Hz, 1H), 6.16 (t, *J* = 2.3 Hz, 1H), 5.19 (h, *J* = 7.0 Hz, 1H), 3.65 (s, 3H), 3.34 (dd, *J* = 13.4, 5.6 Hz, 1H), 2.57 (dd, *J* = 13.4, 8.9 Hz, 1H), 1.37 (d, *J* = 7.0 Hz, 3H).

^{13}C NMR (126 MHz, $CDCl_3$): δ 159.8, 157.4 (br), 155.3, 137.7, 134.0, 133.9, 132.0, 131.0, 129.7, 129.6, 128.8 (br), 128.3, 120.8, 114.1, 112.9, 68.1, 55.2, 42.9, 21.2 (2 aromatic signals are not observed due to signal broadening).

^{19}F NMR (282 MHz, $CDCl_3$): δ -153.07, -153.12

^{11}B NMR (96 MHz, $CDCl_3$): δ -1.26

HRMS (ESI): *m/z* calculated for $C_{33}H_{30}NO^+$ ([M - BF_4]$^+$): 456.2322; found 456.2331.

2,4,6-Triphenyl-1-(1-(3-(trifluoromethyl)phenyl)propan-2-yl)pyridin-1-ium tetrafluoroborate **1ad**:

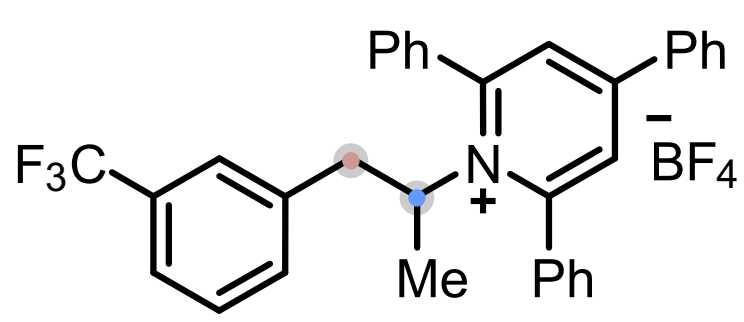

1ad

Synthesized according to General Procedure A described in section 4.1 using 2,4,6-Triphenylpyrylium tetrafluoroborate (2.22 g, 5.60 mmol, 1.00 equiv) and 1-(3-(trifluoromethyl)phenyl)propan-2-amine (1.36 g, 6.72 mmol, 1.20 equiv) to yield **1ad** as a yellow solid in 68% yield (1.87 g).

^{1}H NMR (300 MHz, $CDCl_3$): δ 7.86 – 7.60 (m, 14H), 7.57 – 7.52 (m, 1H), 7.50 (t, *J* = 7.4 Hz, 2H), 7.41 (d, *J* = 7.8 Hz, 1H), 7.25 (t, *J* = 7.8 Hz, 1H, overlaps with $CHCl_3$), 6.89 (d, *J* = 7.8 Hz, 1H), 6.78 (s, 1H), 5.21 – 5.10 (m, 1H), 3.54 (dd, *J* = 13.5, 4.6 Hz, 1H), 2.63 (dd, *J* = 13.5, 9.7 Hz, 1H), 1.33 (d, *J* = 7.2 Hz, 3H) (Note: the resolution of signals 7.86 – 7.60 was inefficient for individual integrations).

^{13}C NMR (126 MHz, $CDCl_3$): δ 155.8, 137.6, 134.1, 133.9, 132.5 (br), 132.2, 131.35, 131.31 (br), 131.1, 131.0 (q, *J* = 272.6 Hz) 129.8, 129.5 (br), 128.5, 125.3 (q, *J* = 3.6 Hz),

124.3 (q, J = 3.8 Hz), 122.8, 67.7, 42.8, 21.1 (3 aromatic signals are not observed due to signal broadening).

^{19}F NMR (282 MHz, $CDCl_3$): δ -62.65, -152.91, -152.96.

^{11}B NMR (96 MHz, $CDCl_3$): δ -1.28.

HRMS (ESI): *m/z* calculated for $C_{33}H_{27}F_3N^+$ ([M - BF_4]$^+$): 494.2091; found 494.2090.

1-(1-(2-Methoxyphenyl)propan-2-yl)-2,4,6-triphenylpyridin-1-ium tetrafluoroborate **1ae**:

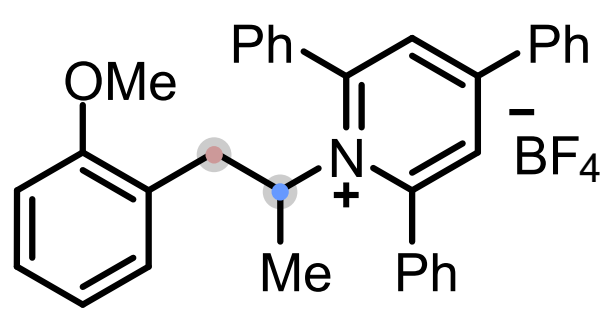

1ae

Synthesized according to General Procedure A described in section 4.1 using 2,4,6-Triphenylpyrylium tetrafluoroborate (832 mg, 2.10 mmol, 1.00 equiv) and 1-(2-methoxyphenyl)propan-2-amine (416 mg, 2.52 mmol, 1.20 equiv) to yield **1ae** as a yellow solid in 38% yield (434 mg).

^{1}H NMR (500 MHz, $CDCl_3$): δ 7.87 (br, 2H), 7.79 – 7.76 (m, 5H), 7.69 – 7.42 (m, 10H), 7.20 (td, J = 7.8, 1.7 Hz, 1H), 6.71 (q, J = 7.7 Hz, 2H), 6.57 (dd, J = 7.4, 1.7 Hz, 1H), 5.28 (h, J = 7.0 Hz, 1H), 3.44 (s, 3H), 3.26 (dd, J = 13.7, 8.0 Hz, 1H), 2.66 (dd, J = 13.7, 6.3 Hz, 1H), 1.46 (d, J = 7.1 Hz, 3H).

^{13}C NMR (126 MHz, $CDCl_3$): δ 157.4, 155.0, 134.1, 134.0, 132.2, 130.8 (br), 130.5, 129.8, 129.4 (br), 129.1, 128.9, 128.3, 124.3, 120.9, 110.3, 67.3, 54.9, 37.8, 22.1 (2 aromatic signals are not observed due to signal broadening).

^{19}F NMR (471 MHz, $CDCl_3$): δ -153.32, -153.38.

^{11}B NMR (96 MHz, $CDCl_3$): δ -1.21.

HRMS (ESI): *m/z* calculated for $C_{33}H_{30}NO^+$ ([M - BF_4]$^+$): 456.2322; found 456.2313.

2,4,6-Triphenyl-1-(1,2,3,4-tetrahydronaphthalen-2-yl)pyridin-1-ium tetrafluoroborate **1ag**:

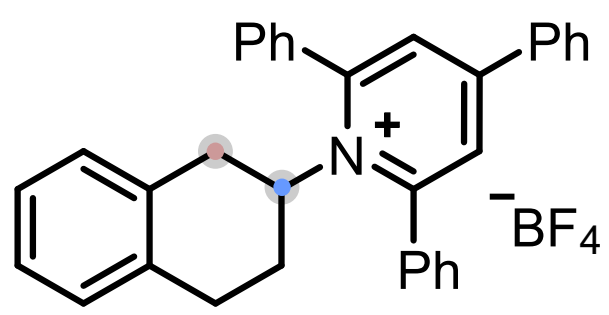

1ag

Synthesized according to General Procedure A described in section 4.1 using 2,4,6-Triphenylpyrylium tetrafluoroborate (1.07 g, 2.70 mmol, 1.00 equiv) and 1,2,3,4-tetrahydronaphthalen-2-amine (477 mg, 3.24 mmol, 1.20 equiv) to yield **1ag** as a tan solid in 51% yield (730 mg).

^{1}H NMR (300 MHz, $CDCl_3$): δ 7.93 – 7.71 (m, 8H), 7.59–7.44 (m, 9H), 7.02 (dd, *J* = 5.7, 3.3 Hz, 2H), 6.90 (dt, *J* = 14.1, 4.7 Hz, 2H), 5.22 – 5.06 (m, 1H), 3.33 (t, *J* = 15.6 Hz, 1H), 3.16 (d, *J* = 15.6 Hz, 1H), 2.70–2.53 (m, 2H), 2.45 (t, *J* = 13.9 Hz, 1H), 1.90–1.72 (m, 1H).

^{13}C NMR (126 MHz, $CDCl_3$): δ 157.6, 155.6, 134.4, 134.1, 134.0, 133.2, 132.2, 131.2, 129.8, 129.26 (br), 129.18 (br), 129.0, 128.8, 128.5, 126.7, 126.2, 68.3, 36.0, 30.7, 30.1 (1 aromatic signal is not observed due to signal broadening).

^{19}F NMR (282 MHz, $CDCl_3$): δ -153.04, -153.10.

^{11}B NMR (96 MHz, $CDCl_3$): δ -1.28.

HRMS (ESI): *m/z* calculated for $C_{35}H_{36}N^+$ ($[M - BF_4]^+$): 438.2216; found 438.2224.

1-(5-Methoxy-1,2,3,4-tetrahydronaphthalen-2-yl)-2,4,6-triphenylpyridin-1-ium tetrafluoroborate **1ah**:

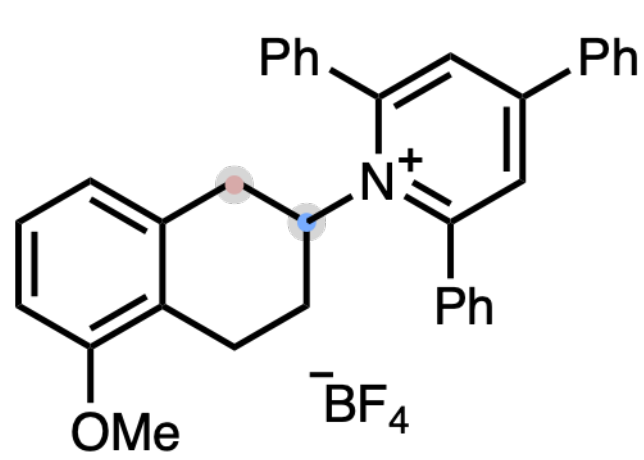

1ah

Synthesized according to General Procedure A described in section 4.1 using 2,4,6-Triphenylpyrylium tetrafluoroborate (1.07 g, 2.70 mmol, 1.00 equiv) and 5-methoxy-1,2,3,4-tetrahydronaphthalen-2-amine (573 mg, 3.24 mmol, 1.20 equiv) to yield **1ah** as a yellow solid in 51% yield (770 mg).

^{1}H NMR (300 MHz, $CDCl_3$): δ 7.89 – 7.78 (m, 6H), 7.76 (d, *J* = 7.2 Hz, 2H), 7.59 – 7.43 (m, 9H), 6.99 (t, *J* = 7.9 Hz, 1H), 6.55 (d, *J* = 8.6 Hz, 2H), 5.11 (tq, *J* = 12.6, 2.4 Hz, 1H), 3.68 (s, 3H), 3.29 (t, *J* = 14.5, 1H), 3.16 (ddd, *J* = 15.6, 4.8, 2.4 Hz, 1H), 2.79 – 2.74 (m,

1H), 2.65 – 2.57 (m, 1H), 2.18 – 2.06 (ddd, *J* = 17.7, 12.0, 5.7 Hz, 1H), 1.76 (qd, *J* = 12.0, 5.7 Hz, 1H).

^{13}C NMR (126 MHz, $CDCl_3$): δ 157.7, 157.1, 155.4, 134.7, 134.1, 134.0, 132.2, 131.1, 129.8 (br), 129.3 (br), 129.1, 128.5, 126.9, 123.2, 121.0, 107.7, 68.4, 55.3, 35.8, 30.6, 24.4 (1 aromatic signal is not observed due to signal broadening).

^{19}F NMR (282 MHz, $CDCl_3$): δ -153.12, -153.17.

^{11}B NMR (96 MHz, $CDCl_3$): δ -1.26.

HRMS (ESI): *m/z* calculated for $C_{34}H_{30}NO^+$ ([M - BF_4]$^+$): 468.2322; found 468.2325.

1-(1-(Furan-2-yl)propan-2-yl)-2,4,6-triphenylpyridin-1-ium tetrafluoroborate **1ai:**

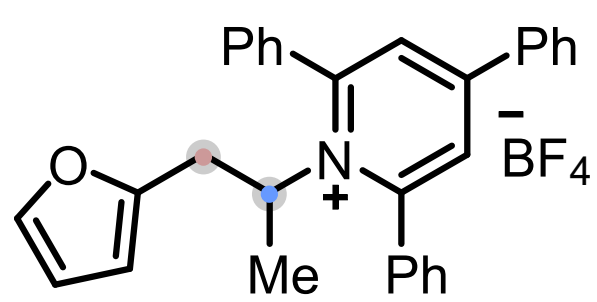

1ai

Synthesized according to General Procedure A described in section 4.1 using 2,4,6-Triphenylpyrylium tetrafluoroborate (1.20 g, 3.00 mmol, 1.00 equiv) and 1-(furan-2-yl)propan-2-amine (450 mg, 3.60 mmol, 1.20 equiv) to yield **1ai** as a white solid in 53% yield (800 mg).

^{1}H NMR (500 MHz, $CDCl_3$): δ 7.84 – 7.76 (m, 6H), 7.62 – 7.47 (m, 10H), 7.16 (d, *J* = 1.8 Hz, 1H), 6.24 (dd, *J* = 3.2, 1.9 Hz, 1H), 5.84 (d, *J* = 3.2 Hz, 1H), 5.28 (h, *J* = 7.1 Hz, 1H), 3.25 (dd, *J* = 15.5, 8.9 Hz, 1H), 2.67 (dd, *J* = 15.6, 6.4 Hz, 1H), 1.55 (d, *J* = 7.1 Hz, 3H, overlaps with water).

^{13}C NMR (126 MHz, $CDCl_3$): δ 155.5, 150.2, 142.2, 134.1, 133.8 (br), 132.2, 131.1 (br), 129.8, 128.9 (br), 128.4, 110.8, 108.0, 65.9, 34.8, 21.9. (3 aromatic signals are not observed due to signal broadening).

^{19}F NMR (282 MHz, $CDCl_3$): δ -152.88.

^{11}B NMR (96 MHz, $CDCl_3$): δ -1.26.

HRMS (ESI): *m/z* calculated for $C_{30}H_{26}NO^+$ ([M - BF_4]$^+$): 416.2009; found 416.2017.

2,4,6-Triphenyl-1-(1-(thiophen-3-yl)propan-2-yl)pyridin-1-ium tetrafluoroborate **1aj:**

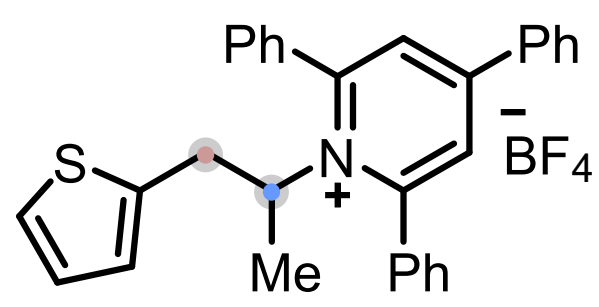

1aj

Synthesized according to General Procedure A described in section 4.1 using 2,4,6-Triphenylpyrylium tetrafluoroborate (436 mg, 1.10 mmol, 1.00 equiv) and 1-(thiophen-2-yl)propan-2-amine (186 mg, 1.32 mmol, 1.20 equiv) to yield **1aj** as a white solid in 48% yield (300 mg).

^{1}H NMR (500 MHz, $CDCl_3$): δ 7.86 – 7.74 (m, 7H), 7.66 – 7.45 (m, 10H), 7.08 (d, *J* = 5.1 Hz, 1H), 6.85 (dd, *J* = 5.2, 3.4 Hz, 1H), 6.43 (d, *J* = 4.3 Hz, 1H), 5.19 (h, *J* = 7.2 Hz, 1H), 3.47 (dd, *J* = 15.2, 7.5 Hz, 1H), 2.88 (dd, *J* = 15.2, 7.4 Hz, 1H), 1.50 (d, *J* = 7.2 Hz, 3H).

^{13}C NMR (126 MHz, $CDCl_3$): δ 155.5, 137.7, 134.1, 133.8 (br), 132.2, 131.1 (br), 129.8, 128.5, 127.4, 126.7, 125.0, 122.6 (br), 67.9, 36.7, 21.6 (3 aromatic signals are not observed due to signal broadening).

^{19}F NMR (282 MHz, $CDCl_3$): δ -153.22.

^{11}B NMR (96 MHz, $CDCl_3$): δ -1.28.

HRMS (ESI): *m/z* calculated for $C_{30}H_{26}NS^+$ ([M - BF_4]$^+$): 432.1781; found 432.1790.

1-(1-(benzofuran-3-yl)propan-2-yl)-2,4,6-triphenylpyridin-1-ium tetrafluoroborate **1ak:**

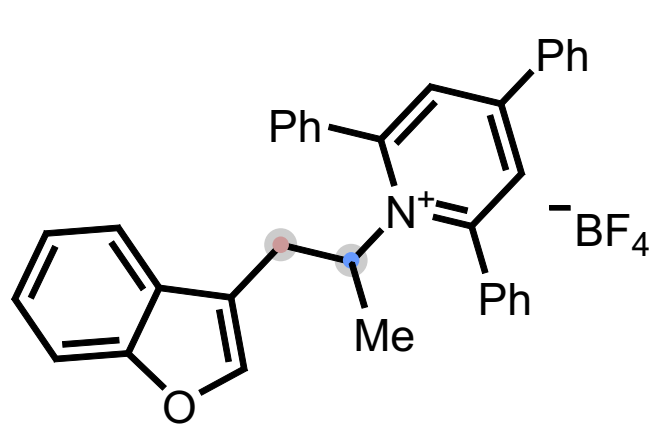

1ak

Synthesized according to General Procedure A described in section 4.1 using 2,4,6-Triphenylpyrylium tetrafluoroborate (673 mg, 1.70 mmol, 1.00 equiv) and 1-(benzofuran-3-yl)propan-2-amine (357 mg, 2.04 mmol, 1.20 equiv) to yield **1ak** as a white solid in 24% yield (250 mg).

^{1}H NMR (500 MHz, $CDCl_3$): δ 7.92 – 7.69 (m, 7H), 7.64 – 7.41 (m, 10H), 7.36 (d, *J* = 8.3 Hz, 1H), 7.21 (t, *J* = 7.2 Hz, 1H), 7.06 – 6.99 (m, 2H), 6.79 (d, *J* = 7.8 Hz, 1H), 5.34 – 5.23 (m, 1H), 3.52 – 3.44 (m, 1H), 2.74 (dd, *J* = 14.5, 9.4 Hz, 1H), 1.44 (d, *J* = 7.1 Hz, 3H).

^{13}C NMR (126 MHz, $CDCl_3$): δ 157.4, 155.4, 155.2, 142.2, 134.0, 133.9, 132.2, 131.1, 129.7, 129.1 (br), 128.4, 126.9, 124.8, 122.8, 119.0, 115.5, 111.6, 66.4, 30.9, 21.9. (2 aromatic signals are not observed due to signal broadening).

^{19}F NMR (282 MHz, $CDCl_3$): δ -152.85.

^{11}B NMR (96 MHz, $CDCl_3$): δ -1.27.

HRMS (ESI): *m/z* calculated for $C_{34}H_{28}NO^+$ ($[M - BF_4]^+$): 466.2166; found 466.2195.

1-(1-(1-methyl-1*H*-indol-3-yl)propan-2-yl)-2,4,6-triphenylpyridin-1-ium tetrafluoroborate **1al:**

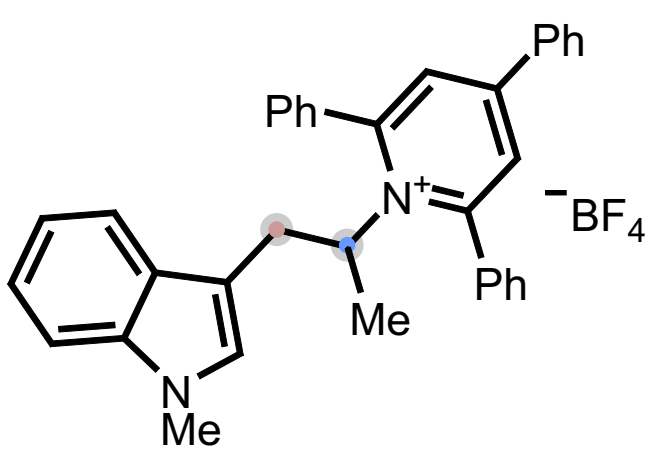

1al

Synthesized according to General Procedure A described in section 4.1 using 2,4,6-Triphenylpyrylium tetrafluoroborate (554 mg, 1.40 mmol, 1.00 equiv) and 1-(1-methyl-1*H*-indol-3-yl)propan-2-amine (316 mg, 1.68 mmol, 1.20 equiv) to yield **1al.** The crude material was taken forward without further purification.

(*Z*)-1-(1-(11-(3-(Dimethylamino)propylidene)-6,11-dihydrodibenzo[b,e]oxepin-2-yl)propan-2-yl)-2,4,6-triphenylpyridin-1-ium tetrafluoroborate **1as:**

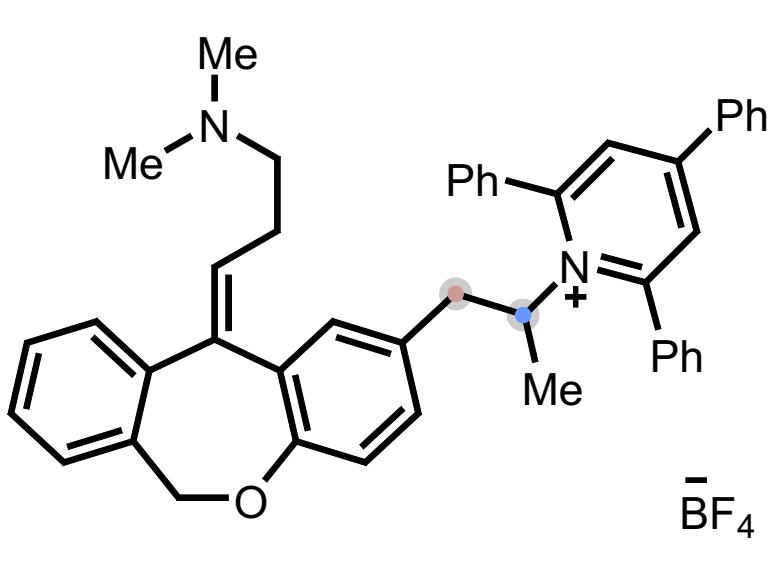

1as

Synthesized according to General Procedure C described in section 4.1 using (*Z*)-2-(11-(3-(dimethylamino)propylidene)-6,11-dihydrodibenzo[*b*,*e*]oxepin-2-yl)acetic acid hydrochloride as the acid (438 mg, 4.50 mmol, 1.50 equiv) to yield **1as** as a pale yellow solid in 40% yield (240 mg) over 4 steps.

^{1}H NMR (500 MHz, $CDCl_3$) δ 7.83 – 7.71 (m, 7H), 7.61 – 7.43 (m, 10H), 7.33 – 7.08 (m, 4H, overlaps with $CDCl_3$), 6.58 (d, J = 8.4 Hz, 1H), 6.43 (s, 1H), 6.34 (dd, J = 8.4, 2.3 Hz, 1H), 5.63 (t, J = 6.8 Hz, 1H), 5.48 – 4.54 (m, 3H, two overlapping signals), 3.33 – 3.26 (m, 1H), 2.53 – 2.28 (m, 5H), 2.20 (s, 6H), 1.36 (d, J = 6.9 Hz, 3H).

^{13}C NMR (126 MHz, $CDCl_3$) δ 155.2, 154.7, 145.6, 139.5, 134.0, 133.9, 133.5, 132.1, 131.6 (br), 131.0 (br), 130.8 (br), 129.7, 129.2 (br), 128.4, 128.1, 127.8, 126.1 (br), 124.2 (br), 120.0, 70.4, 68.4, 66.0, 59.3, 45.3, 42.2, 27.9, 21.3, 15.4. (8 aromatic signals are not observed due to signal broadening).

^{19}F NMR (282 MHz, $CDCl_3$): δ -152.85.

^{11}B NMR (96 MHz, $CDCl_3$): δ -1.27.

HRMS (ESI): *m/z* calculated for $C_{45}H_{43}N_2O^+$ ($[M - BF_4]^+$): 627.3370; found 627.3370.

1-(4-(4-((4-(6-(1-(2,2-Difluorobenzo[d][1,3]dioxol-5-yl)cyclopropane-1-carboxamido)-3-methylpyridin-2-yl)benzoyl)oxy)phenyl)butan-2-yl)-2,4,6-triphenylpyridin-1-ium tetrafluoroborate **1aw**:

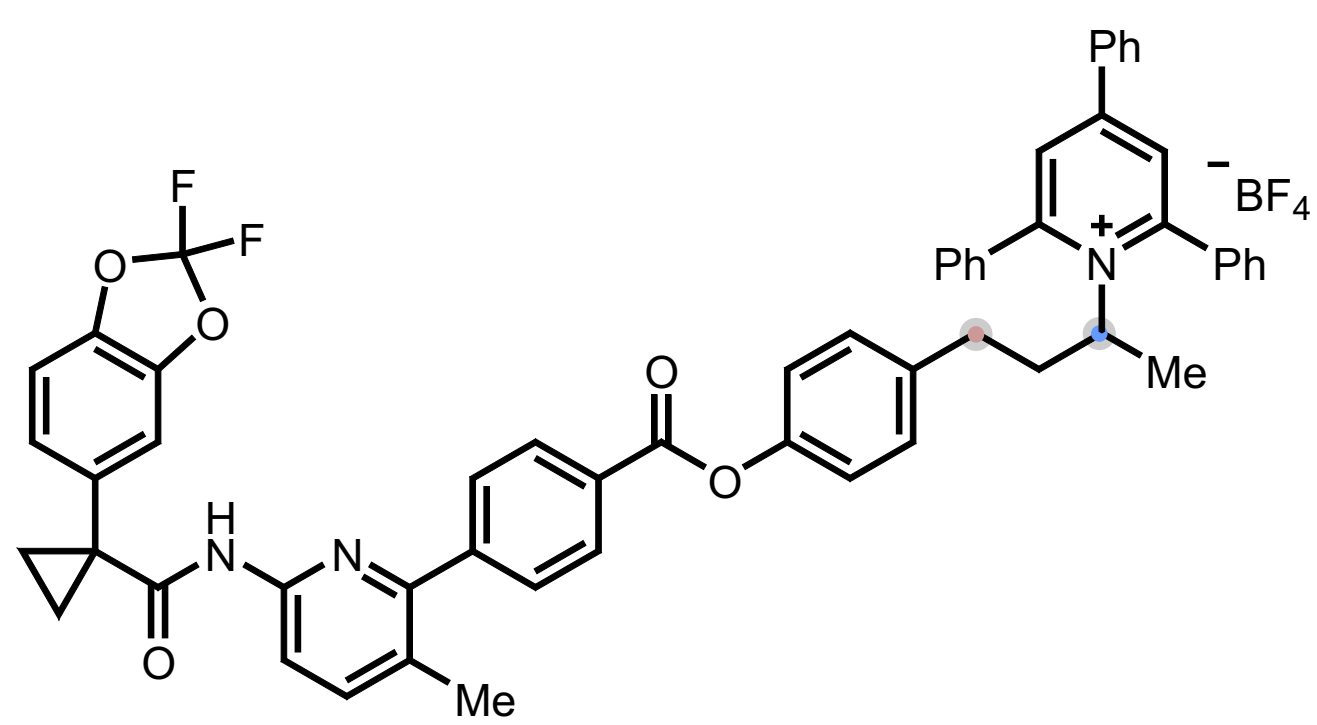

1aw

Synthesized according to General procedure D described in section 4.1 using Lumacaftor (226 mg, 0.500 mmol, 1.00 equiv) and **1y** (272 mg, 0.500 mmol, 1.00 equiv). The crude product was subjected to silica gel flash column chromatography (100% DCM) to yield **1aw** as a white solid at 30% yield (145 mg, 0.150 mmol).

^{1}H NMR (500 MHz, $(CD_3)_2CO$): δ 8.38 – 8.37 (m, 2H), 8.22 – 8.17 (m, 3H), 8.11 – 8.04 (m, 2H), 7.89 (br s, 2H), 7.78 (dt, J = 7.7, 1.5 Hz, 1H), 7.76 (s, 1H), 7.74 – 7.61 (m, 12H), 7.53 – 7.49 (m, 1H), 7.45 – 7.40 (m, 1H), 7.31 – 7.26 (m, 1H), 7.16 (s, 1H), 7.14 (d, J = 8.9 Hz, 1H), 7.11 (d, J = 8.9 Hz, 1H), 5.18 – 5.10 (m, 1H), 2.60 – 2.52 (m, 1H), 2.40 –

2.33 (m, 1H), 2.31 – 2.23 (m, 4H), 2.09 (s, 1H), 1.99 – 1.91 (m, 1H), 1.66 (d, *J* = 6.7, 3H), 1.65 – 1.63 (m, 2H), 1.24 – 1.19 (m, 2H). Mixture of rotamers.

^{13}C NMR (126 MHz, $(CD_3)_2CO$): δ 172.1, 165.4, 163.2, 155.9, 155.5, 150.6, 150.4, 149.7, 144.6, 144.0, 141.8, 141.72, 141.65, 139.3, 138.3, 137.7, 137.0, 135.0, 134.4, 134.2, 133.4, 133.2, 132.58, 132.56 (t, *J* = 253 Hz), 131.9, 131.6, 131.3, 131.1, 130.9, 130.6, 130.5, 130.4, 130.2, 130.0, 129.9, 129.5, 129.43, 129.40, 129.1, 129.0, 128.09, 128.04, 127.5, 122.8, 122.4, 113.58, 113.51, 111.09, 111.06, 67.5, 67.4, 38.5, 32.3, 32.2, 32.0, 22.8, 22.7, 19.21, 19.18, 17.11, 17.06. Mixture of rotamers.

^{19}F NMR (282 MHz, $(CD_3)_2CO$): δ -50.90, -151.81, -151.86.

^{11}B NMR (96 MHz, $(CD_3)_2CO$): δ -0.92.

HRMS (ESI): *m/z* calculated for $C_{57}H_{46}F_2N_3O_5^+$ ($[M - BF_4]^+$): 890.3401; found: 890.3404.

1-(4-(4-(((R)-2-(6-Methoxynaphthalen-2-yl)propanoyl)oxy)phenyl)butan-2-yl)-2,4,6-triphenylpyridin-1-ium tetrafluoroborate **1ax**:

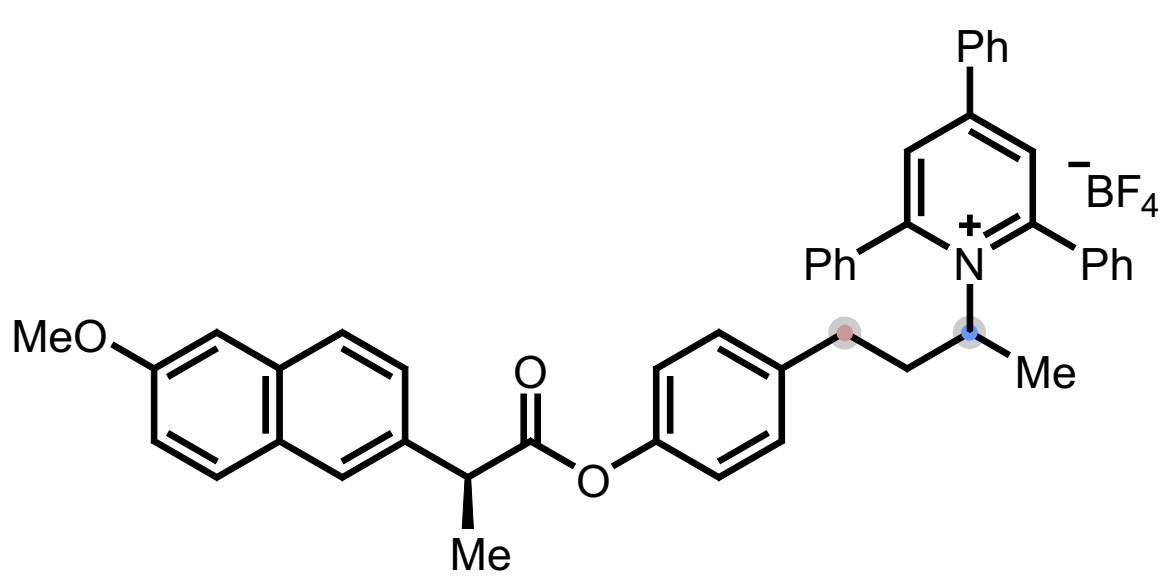

1ax

Synthesized according to General procedure D described in section 4.1 using Naproxen (200 mg, 0.874 mmol, 1.00 equiv) and 1y (500 mg, 0.920 mmol, 1.05 equiv). The crude product was purified by silica gel flash column chromatography (100% DCM → 9:91 methanol/DCM) to yield **1ax** as a white solid in 76% yield (500 mg, 0.664 mmol).

^{1}H NMR (500 MHz, $(CD_3)_2CO$): δ 8.32 (br s, 2H), 8.17 (d, *J* = 7.5 Hz, 2H), 7.90 – 7.81 (m, 5H), 7.73 – 7.58 (m, 11H), 7.56 (dd, *J* = 8.4, 2.0 Hz, 1H), 7.33 (d, *J* = 2.7 Hz, 1H), 7.18 (dd, *J* = 8.9, 2.5 Hz, 1H), 6.98 (d, *J* = 7.5 Hz, 2H), 6.85 (dd, *J* = 8.6, 1.3 Hz, 2H), 5.09 – 5.01 (m, 1H), 4.20 (q, *J* =7.1 Hz, 1H), 3.92 (s, 3H), 2.50 – 2.44 (m, 1H), 2.31 – 2.25 (m, 1H), 2.22 – 2.14 (m, 1H), 1.92 – 1.83 (m, 1H), 1.65 (d, *J* = 2.7 Hz, 3H), 1.61 (dd, *J* = 7.0, 1.7 Hz, 3H).

^{13}**C NMR** (126 MHz, $(CD_3)_2CO$): δ 173.65, 173.64, 158.9, 155.4, 150.52, 150.50, 138.0, 136.58, 136.56, 135.0, 134.9, 134.4, 133.3, 131.8, 130.6, 130.1, 129.9, 129.7 (br), 129.42 (br), 129.40, 129.38 (br), 128.3, 127.0, 126.94, 126.92, 122.4, 119.9, 106.6, 67.35, 67.32, 46.11, 46.09, 38.42, 38.38, 32.3, 32.2, 22.65, 22.64, 18.82, 18.80, 14.3. Mixture of diastereomers.

^{19}**F NMR** (282 MHz, $(CD_3)_2CO$): δ -151.69, -151.75.

^{11}**B NMR** (96 MHz, $(CD_3)_2CO$): δ -0.92.

HRMS (ESI): *m/z* calculated for $C_{47}H_{42}NO_3^+$ ($[M - BF_4]^+$): 668.3160; found: 668.3162.

1-(4-(4-((2-(3-Cyano-4-isobutoxyphenyl)-4-methylthiazole-5-carbonyl)oxy)phenyl)butan-2-yl)-2,4,6-triphenylpyridin-1-ium tetrafluoroborate **1ay**:

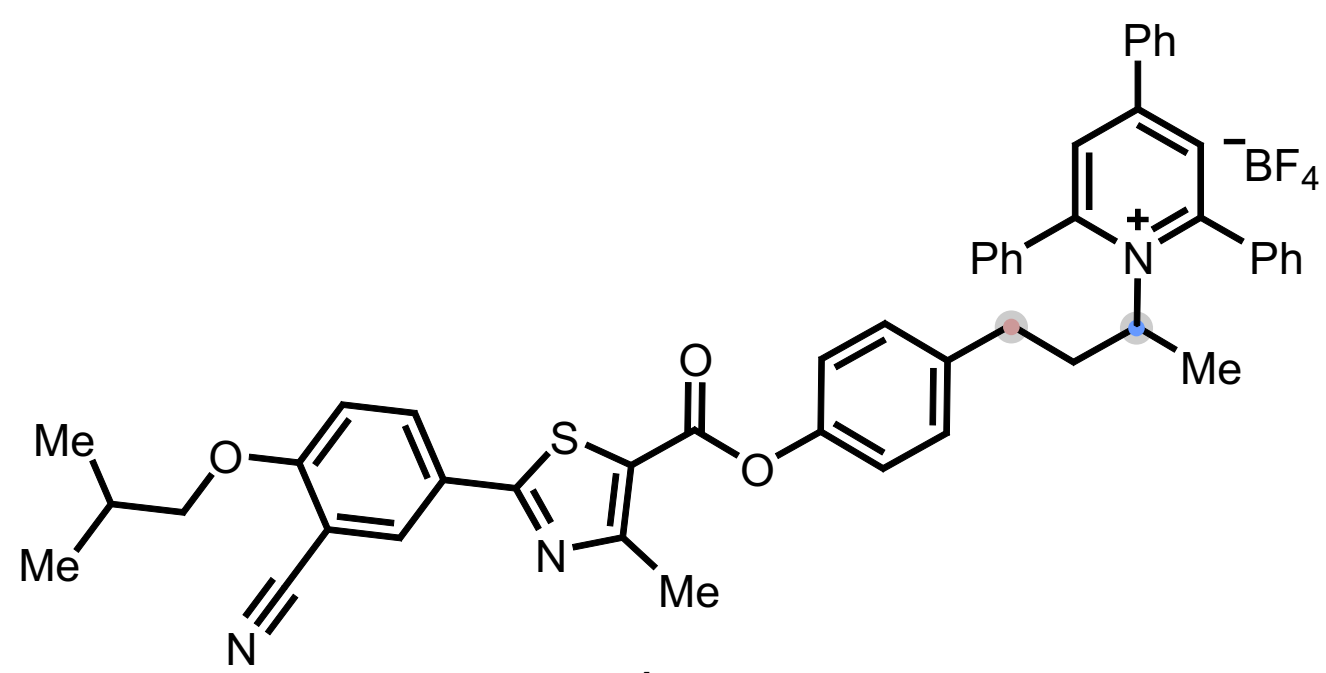

1ay

Synthesized according to General procedure D described in section 4.1 using Febuxostat (158 mg, 0.50 mmol, 1.00 equiv) and **1y** (272 mg, 0.500 mmol, 1.00 equiv) to yield **1ay** as a white solid at 77% yield (323 mg, 0.385 mmol). The crude material was used without further purification.

^{1}**H NMR** (500 MHz, $(CD_3)_2CO$): δ 8.34 (br s, 2H), 8.33 – 8.29 (m, 2H), 8.20 – 8.18 (m, 2H), 7.92 – 7.79 (m, 3H), 7.73 – 7.65 (m, 8H), 7.62 (tt, *J* = 7.7, 2.1 Hz, 2H), 7.41 (d, *J* = 8.8 Hz, 1H), 7.12 (s, 4H), 5.14 – 5.06 (m, 1H), 4.08 (d, *J* = 6.4 Hz, 2H), 2.78 (s, 3H), 2.58 – 2.49 (m, 1H), 2.39 – 2.25 (m, 2H), 2.23 – 2.13 (m, 1H), 1.97 – 1.88 (m, 1H), 1.65 (d, *J* = 7.0 Hz, 3H), 1.09 (d, *J* = 6.7 Hz, 6H).

^{13}**C NMR** (126 MHz, $(CD_3)_2CO$): δ 169.0, 163.7, 163.2, 161.0, 155.3, 150.0, 138.6, 135.0, 134.4, 133.9, 133.3, 132.7, 131.8, 130.6, 130.1, 129.7 (br), 129.4, 127.8, 126.6, 122.8,

121.6, 116.8, 115.9, 114.5, 103.5, 76.4, 67.4, 38.4, 32.3, 28.9, 22.7, 19.2, 17.8. (1 aromatic signal is not observed due to signal broadening).

^{19}F NMR (282 MHz, $(CD_3)_2CO$): δ -151.65, -151.70.

^{11}B NMR (96 MHz, $(CD_3)_2CO$): δ -0.92.

HRMS (ESI): *m/z* calculated for $C_{49}H_{44}N_3O_3S^+$ ($[M - BF_4]^+$): 754.3098; found: 754.3099.

7-Phenyl-14-(4-phenylbutyl)-5,6,8,9-tetrahydrodibenzo[c,h]acridin-14-ium tetrafluoroborate **Acridin1:**

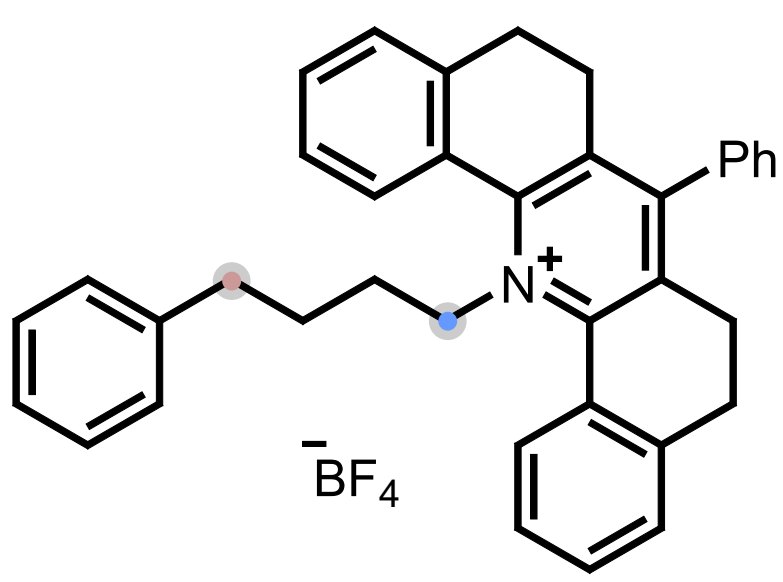

Acridin1

Synthesized according to General procedure B described in section 4.1 using 7-phenyl-5,6,8,9-tetrahydrodibenzo[*c*,*h*]xanthen-14-ium tetrafluoroborate (1.34 g, 3.00 mmol, 1.00 equiv) and 4-phenylbutan-1-amine (536 mg, 3.60 mmol, 1.20 equiv) to yield **Acridin1** as a yellow solid in 83% yield (1.44 g). The crude material was used without further purification.

^{1}H NMR (500 MHz, $CDCl_3$): δ 8.46 (d, *J* = 7.9 Hz, 2H), 7.64 (t, *J* = 7.6 Hz, 3H), 7.59 – 7.46 (m, 4H), 7.35 (d, *J* = 7.6 Hz, 3H), 7.23 – 7.09 (m, 4H), 6.82 (d, *J* = 7.8 Hz, 2H), 5.41 (t, *J* = 7.1 Hz, 2H), 2.85 – 2.61 (m, 8H), 2.26 (t, *J* = 7.3 Hz, 2H), 1.31 (q, *J* = 7.3 Hz, 2H), 1.06 (p, *J* = 7.5 Hz, 2H).

^{13}C NMR (126 MHz, $CDCl_3$): δ 155.1, 153.5, 141.0, 140.8, 137.5, 135.2, 133.0, 130.2, 129.6, 128.8, 128.5, 128.4, 128.34, 128.28, 127.6 (br), 126.2, 64.4, 34.6, 29.5, 28.1, 27.6, 26.6.

^{19}F NMR (282 MHz, $CDCl_3$): δ -153.16.

^{11}B NMR (96 MHz, $CDCl_3$): δ -0.87.

HRMS (ESI): *m/z* calculated for $C_{37}H_{34}N^+$ ($[M - BF_4]^+$): 492.2686; found 492.2696.

14-(4-Phenylbutyl)-7-(4-(trifluoromethyl)phenyl)-5,6,8,9-tetrahydrodibenzo[c,h]acridin-14-ium trifluoromethanesulfonate **Acridin2:**

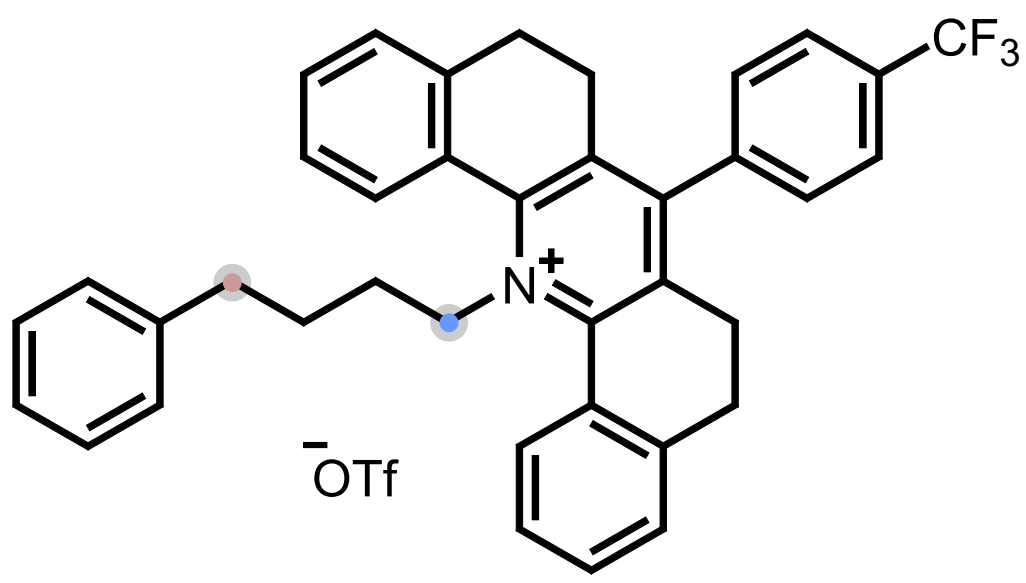

Acridin2

Synthesized according to General procedure B described in section 4.1 using 7-(4-(trifluoromethyl)phenyl)-5,6,8,9-tetrahydrodibenzo[*c*,*h*]xanthen-14-ium trifluoromethanesulfonate (578 mg, 1.00 mmol, 1.00 equiv) and 4-phenylbutan-1-amine (179 mg, 1.20 mmol, 1.20 equiv) to yield **Acridin2.** The crude material was used without further purification.

14-(2-(1-Phenyl-1H-pyrazol-4-yl)ethyl)-7-(4-(trifluoromethyl)phenyl)-5,6,8,9-tetrahydrodibenzo[c,h]acridin-14-ium trifluoromethanesulfonate **Acridin3:**

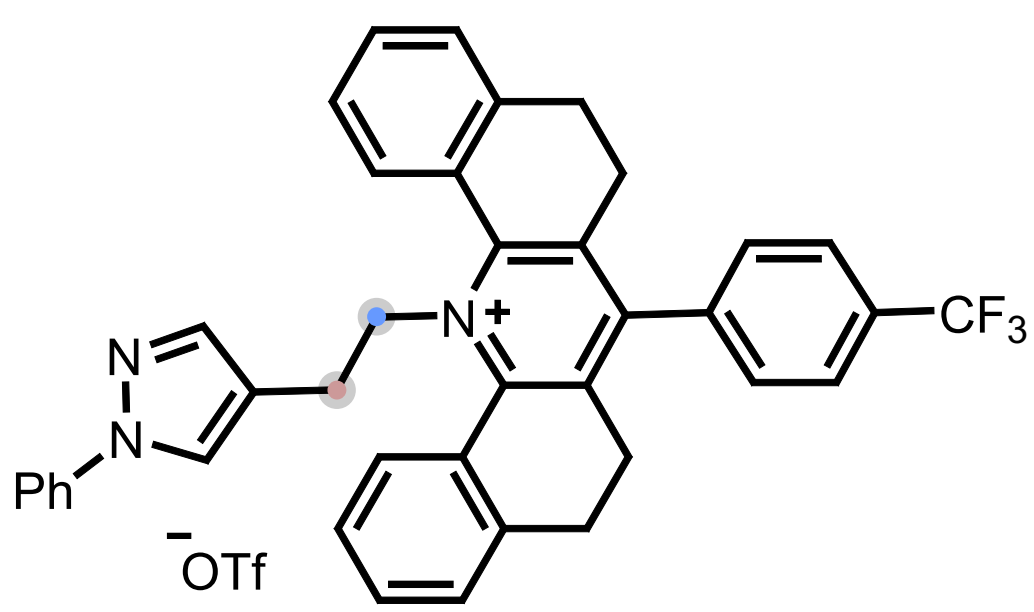

Acridin3

Synthesized according to General procedure B described in section 4.1 using 7-(4-(trifluoromethyl)phenyl)-5,6,8,9-tetrahydrodibenzo[*c*,*h*]xanthen-14-ium trifluoromethanesulfonate (1.33 g, 2.30 mmol, 1.00 equiv) and 2-(1-phenyl-1*H*-pyrazol-4-yl)ethan-1-amine (516 mg, 2.76 mmol, 1.20 equiv) to yield **Acridin3** as a yellow solid in 64% yield (200 mg). The crude material was used without further purification.

^{1}H NMR (500 MHz, $CDCl_3$): δ 8.46 (d, *J* = 7.9 Hz, 2H), 7.78 – 7.71 (m, 2H), 7.67 (t, *J* = 7.6 Hz, 2H), 7.60 – 7.50 (m, 5H), 7.48 – 7.41 (m, 4H), 7.38 (d, *J* = 7.6 Hz, 2H), 7.32 (t, *J* = 7.4 Hz, 1H), 6.95 (s, 1H), 5.72 (t, *J* = 6.3 Hz, 1H), 2.78 – 2.71 (m, 4H), 2.62 – 2.53 (m, 4H), 2.37 – 2.26 (m, 2H).

^{13}C NMR (126 MHz, $CDCl_3$): δ 153.7, 153.4, 140.9, 140.4, 139.6, 138.7, 137.1, 133.3, 131.79 (q, *J* = 33.1 Hz), 130.1, 129.9, 128.9, 128.8, 128.2, 128.1, 127.0, 126.7 (br), 126.2, 123.7 (q, *J* = 272.8 Hz), 121.1 (q, *J* = 320.7 Hz), 118.7, 117.0, 65.8, 27.8, 26.4, 25.6.

^{19}F NMR (282 MHz, $CDCl_3$): δ -62.88, -78.17.

HRMS (ESI): *m/z* calculated for $C_{39}H_{31}F_3N_3^+$ ([M - OTf]$^+$): 598.2465; found 598.2485.

14-(2-(1-Phenyl-1H-pyrazol-4-yl)ethyl)-7-(4-(trifluoromethyl)phenyl)-5,6,8,9-tetrahydrodibenzo[c,h]acridin-14-ium trifluoromethanesulfonate **Acridin4:**

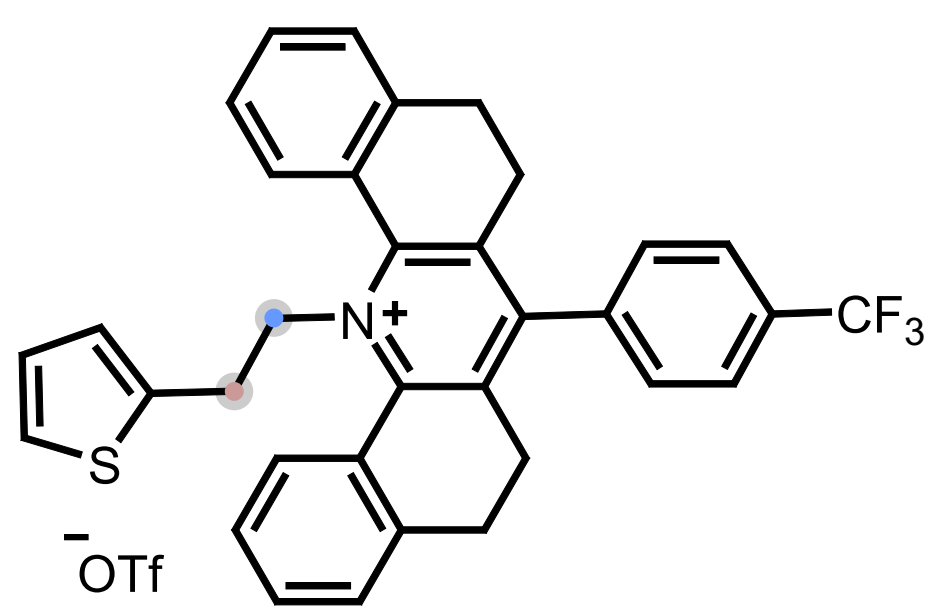

Acridin4

Synthesized according to General procedure B described in section 4.1 using 7-(4-(trifluoromethyl)phenyl)-5,6,8,9-tetrahydrodibenzo[*c*,*h*]xanthen-14-ium trifluoromethanesulfonate (318 mg, 0.55 mmol, 1.00 equiv) and 2-(thiophen-2-yl)ethan-1-amine (83.8 mg, 0.66 mmol, 1.20 equiv) to yield **Acridin4**. The crude material was used without further purification.

7-(4-((Difluoro-l3-methyl)-l2-fluoraneyl)phenyl)-14-(4-hydroxy-3-methoxyphenethyl)-5,6,8,9-tetrahydrodibenzo[c,h]acridin-14-ium trifluoromethanesulfonate **Acridin5:**

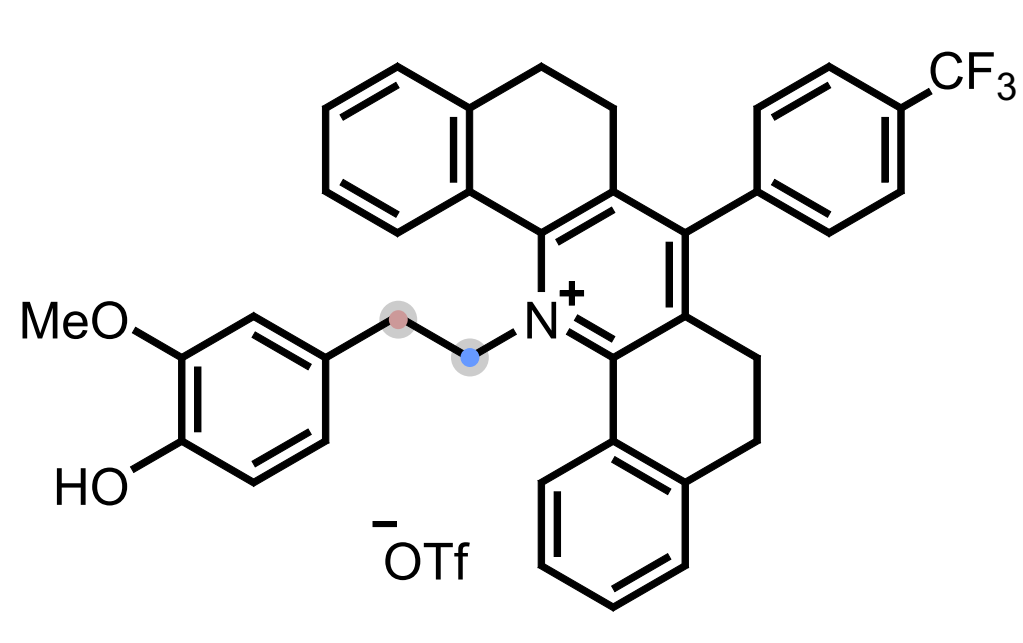

Acridin5

Synthesized according to General procedure B described in section 4.1 using 7-(4-(trifluoromethyl)phenyl)-5,6,8,9-tetrahydrodibenzo[*c*,*h*]xanthen-14-ium trifluoromethanesulfonate (809 mg, 1.40 mmol, 1.00 equiv) and 4-(2-aminoethyl)-2-methoxyphenol (281 mg, 1.68 mmol, 1.20 equiv) to yield **Acridin5** as a yellow solid in 78% yield (800 mg). The crude material was used without further purification.

^{1}H NMR (500 MHz, $CDCl_3$): δ 8.51 (d, *J* = 8.0 Hz, 2H), 7.86 (dd, *J* = 97.0, 8.0 Hz, 2H), 7.67 (t, *J* = 7.7 Hz, 2H), 7.53 (t, *J* = 7.0 Hz, 2H), 7.38 (d, *J* = 7.4 Hz, 2H), 7.31 (d, *J* = 8.0 Hz, 2H), 7.23 (d, *J* = 8.1 Hz, 1H), 6.72 (d, *J* = 7.9 Hz, 1H), 6.12 (d, *J* = 2.2 Hz, 1H), 6.12 (d, *J* = 2.2 Hz, 1H), 5.96 (dd, *J* = 7.9, 2.1 Hz, 1H), 5.82 (t, *J* = 5.9 Hz, 2H), 3.51 (s, 3H), 2.74 (dd, *J* = 15.1, 5.4 Hz, 2H), 2.62 – 2.48 (m, 8H), 2.28 (td, *J* = 16.3, 5.1 Hz, 1H).

^{13}C NMR (126 MHz, $CDCl_3$): δ 153.5, 153.1, 147.1, 145.7, 140.7, 138.8, 136.9, 133.1, 131.83 (q, *J* = 33.1 Hz), 130.3, 128.9, 128.6, 128.2, 127.0, 126.8, 126.5, 123.8 (q, *J* = 264.6 Hz), 121.1 (q, *J* = 321.3 Hz), 120.9, 114.2, 110.9, 66.1, 55.9, 36.6, 27.7, 26.4.

^{19}F NMR (282 MHz, $CDCl_3$): δ -62.85, -78.14.

HRMS (ESI): *m/z* calculated for $C_{37}H_{31}F_3NO_2^+$ ([M - OTf]$^+$): 578.2302; found 578.2326.

4.3 Synthesis and Characterization of Aryl Bromides

The following aryl bromides have been previously reported[7–9] and were prepared accordingly:

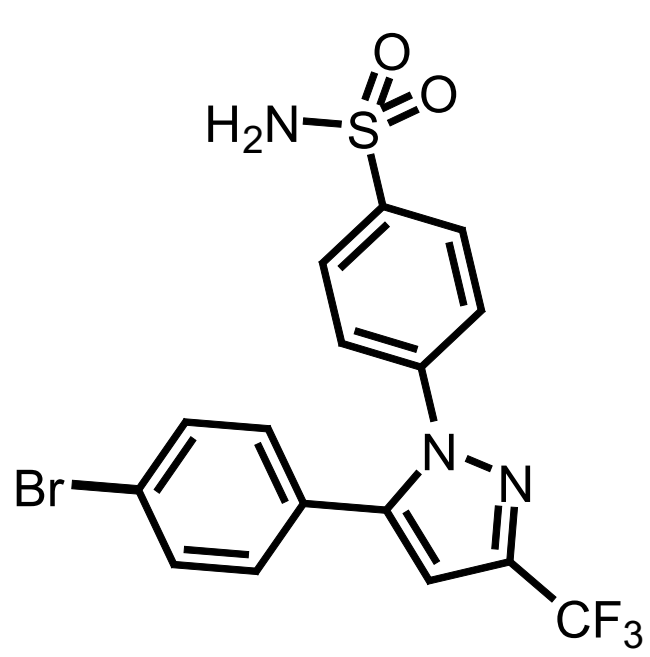

2ao

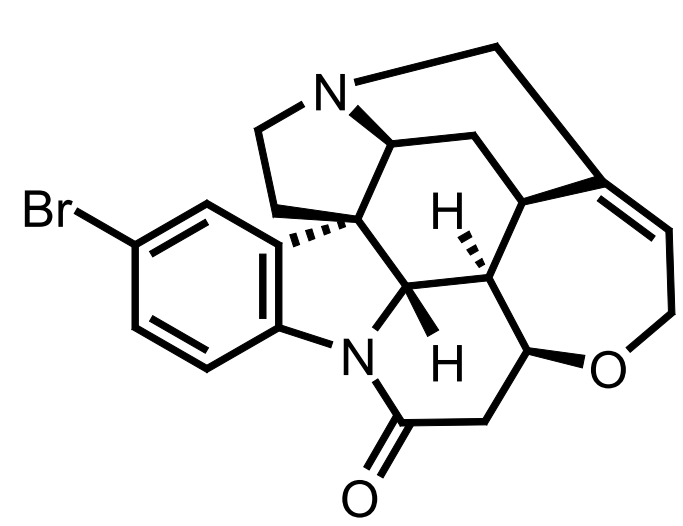

2aq

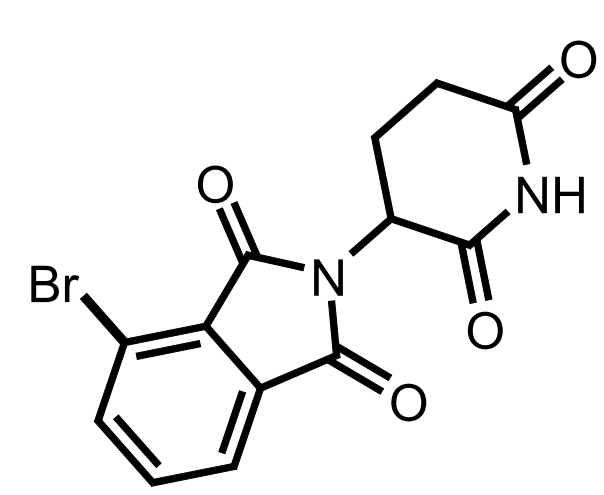

2ar

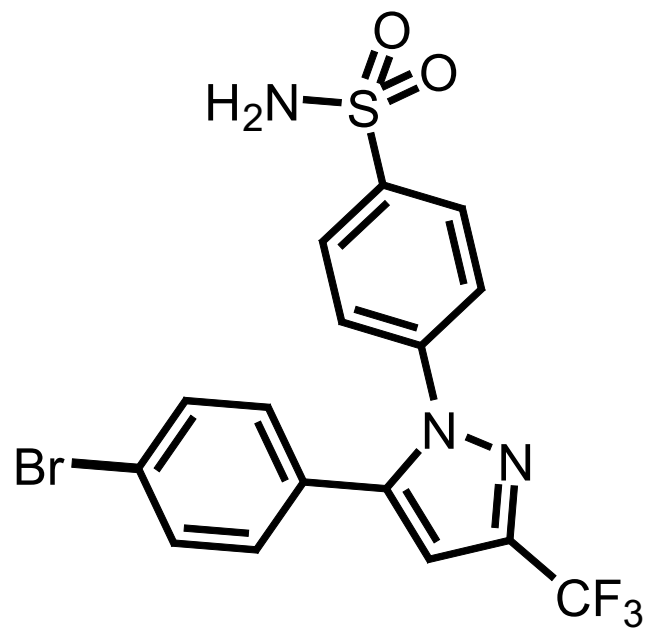

2ao

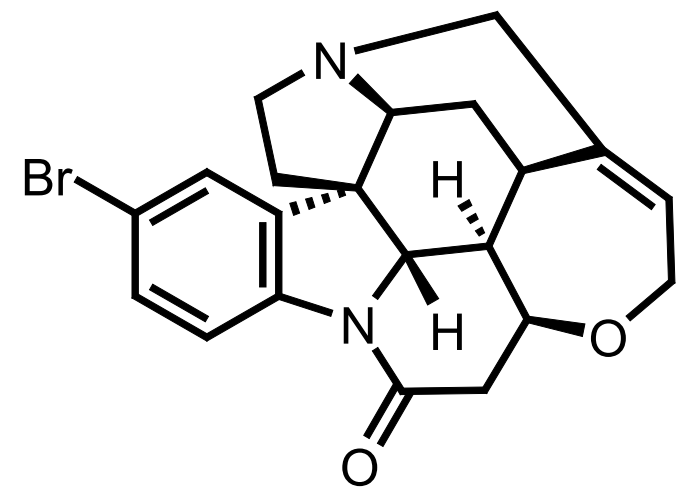

2aq

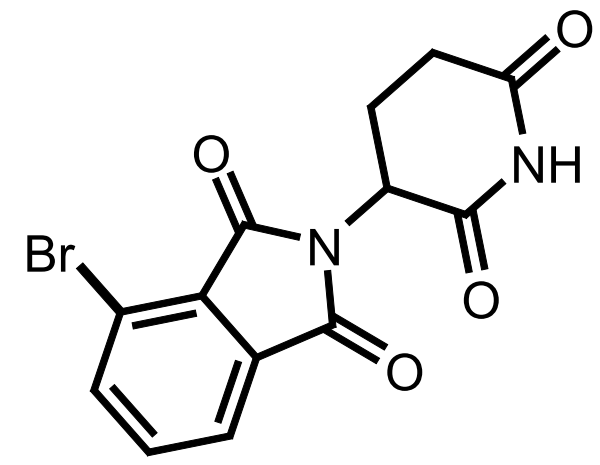

2ar

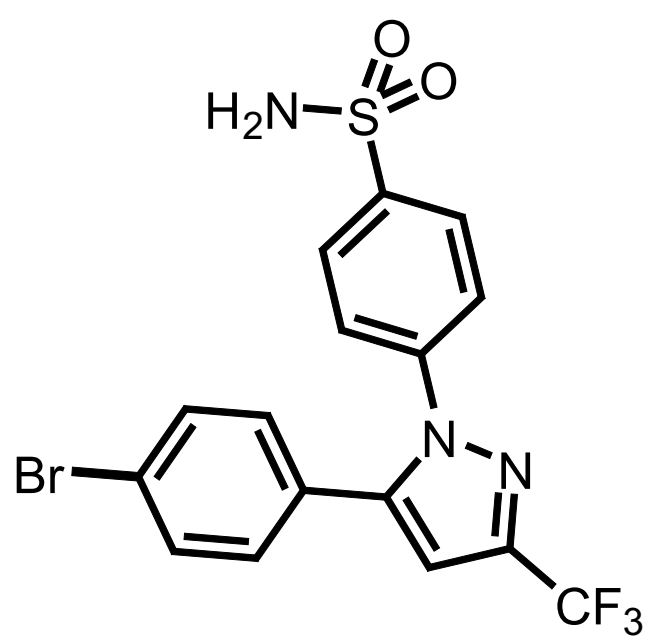

2ao

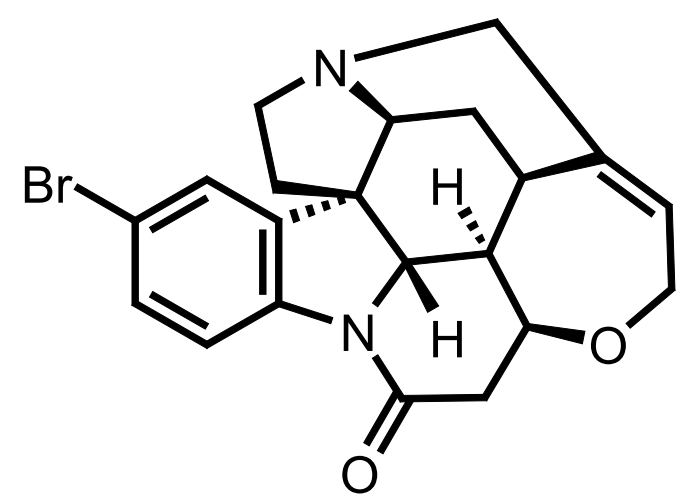

2aq

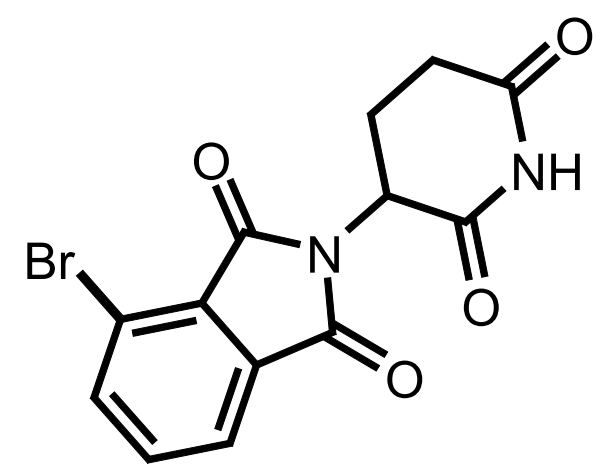

2ar

5-Benzoyl-N-(3-bromophenyl)-2,3-dihydro-1H-pyrrolizine-1-carboxamide **2ap**:

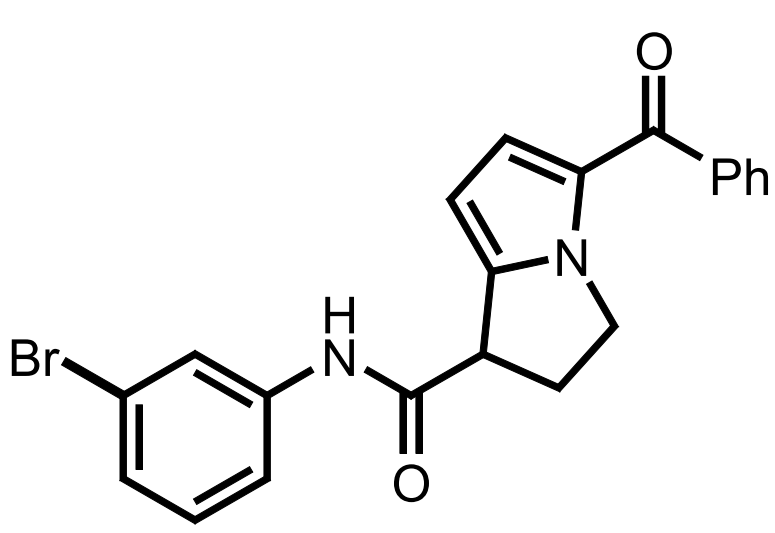

2ap

The titled compound **2ap** was prepared according to a modified procedure[10]: A flame-dried 250 mL round bottom flask was charged with 5-benzoyl-2,3-dihydro-1*H*-pyrrolizine-1-carboxylic acid (1.02 g, 4.00 mmol, 1.00 equiv), 3-bromoaniline (0.825 g , 4.80 mmol, 1.20 equiv) and diisopropylethylamine (0.836 mL, 4.80 mmol, 1.20 equiv) and cooled to 0 °C. Then, *N*,*N'*-dicyclohexylcarbodiimide (0.990 g, 4.80 mmol, 1.20 equiv) was added portion-wise over a period of 5 minutes and allowed to stir at 0 °C for another 5 minutes. Then, the ice bath was removed, and the reaction mixture was allowed to stir for an additional 18 h. The crude mixture was filtered through Celite® and the Celite®

was washed further with DCM. The reaction mixture was then washed with HCl (1M aq.) and sat. $NaHCO_3$ (aq.), dried with Na_2SO_4, filtered, and concentrated under reduced pressure. The crude reaction mixture was purified by flash column chromatography using 100:0 → 97:3 DCM/ethyl acetate as an eluent to yield compound **2ap** as a white solid in 50% yield (0.816 g; 2.00 mmol).

^{1}H NMR (500 MHz, $CDCl_3$): δ 7.84 (br, 1H), 7.82 (dt, J = 7.0, 1.4 Hz, 2H), 7.74 (t, J = 1.9 Hz, 1H), 7.55 (tt, J = 7.4, 1.9 Hz, 1H), 7.46 (tt, J = 7.4, 1.6 Hz, 2H), 7.43 (dq, J = 8.1, 1.1 Hz, 1H), 7.25 (dq, J = 8.1, 1.1, 1H), 7.17 (t, J = 8.1 Hz, 1H), 6.88 (d, J = 4 Hz, 1H), 6.16 (d, J = 4 Hz, 1H), 4.56 (dt, J = 12.2, 7.2, 1H), 4.46 (dt, J = 12.2, 7.2, 1H), 4.06 (t, J = 7.02, 1H), 2.95 (q, J = 7.4 Hz, 2H).

^{13}C NMR (126 MHz, $CDCl_3$): δ 185.4, 169.3, 142.3, 139.1, 138.8, 131.8, 130.5, 129.0, 128.4, 128.0, 127.9, 125.3, 123.0, 122.8, 118.5, 103.0, 47.8, 45.4, 32.7.

HRMS (ESI): m/z calculated for $C_{21}H_{18}BrN_2O_2^+$ ($[M + H]^+$): 409.0547; found: 409.0547.

5. Synthesis and Characterization of Dihydropyridines 6 and 7

2,4,6-Triphenyl-1,4-bis(4-phenylbutan-2-yl)-1,4-dihydropyridine **6**:

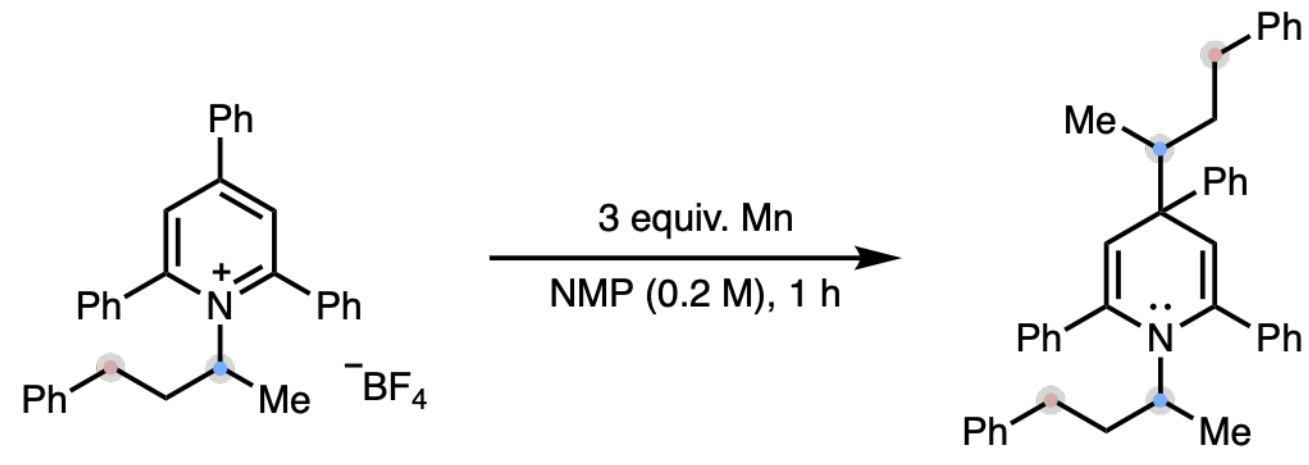

Inside a nitrogen-filled glovebox, a 1-dram vial equipped with a PTFE-coated magnetic stir bar was charged with 2,4,6-triphenyl-1-(4-phenylbutan-2-yl)pyridin-1-ium tetrafluoroborate **1a** (52.7 mg, 0.100 mmol, 1.00 equiv), manganese powder (16.5 mg, 0.300 mmol, 3.00 equiv), and 0.500 mL NMP. The vial was then hermetically sealed with a syringeable cap (PP/PTFE/silicone liner septum) and stirred at room temperature for 1 hour at 1500 RPM. The vial was then removed from the glovebox, diluted with ethyl acetate (approximately 2 mL), and then washed with 10% LiCl (aq.) solution (approximately 1 mL) and brine (approximately 1 mL). The organic layer was dried over Na_2SO_4, filtered, and concentrated *in vacuo* to obtain crude reaction mixture. The crude rection mixture was purified by flash column chromatography using 100:0 → 90:10 hexane/diethyl ether as an eluent followed by preparative thin-layer chromatography (95:5 pentane/diethyl ether) to yield 2,4,6-triphenyl-1,4-bis(4-phenylbutan-2-yl)-1,4-dihydropyridine **6** (white solid; 48% isolated yield) as a mixture of diastereomers (1:1 dr).

^{1}H NMR (500 MHz, $CDCl_3$): δ 7.55 – 7.51 (m, 8H), 7.40 – 7.38 (m, 4H), 7.35 – 7.29 (m, 16H), 7.18 – 7.03 (m, 14H), 7.00 – 6.99 (m, 4H), 6.64 – 6.62 (m, 4H), 5.17 – 5.16 (m, 2H), 5.14 – 5.12 (m, 2H), 3.13 – 3.06 (m, 2H), 2.66 – 2.60 (m, 2H), 2.35 – 2.29 (m, 2H), 2.16 – 2.06 (m, 4H), 1.80 – 1.71 (m, 4H), 1.58 – 1.49 (m, 2H, overlaps with water), 1.29 – 1.20 (m, 2H, overlaps with grease), 1.13 – 1.05 (m, 2H), 0.85 – 0.83 (m, 6H), 0.64 (d, J = 7.0 Hz, 6H).

^{13}C NMR (126 MHz, $CDCl_3$): δ 151.4, 146.0, 145.6, 145.3, 144.9, 142.9, 141.8, 140.98, 140.96, 140.71, 140.69, 128.78, 128.75, 128.6, 128.27, 128.26, 128.21, 128.15, 128.13,

128.10, 128.08, 127.9, 127.8, 127.0, 125.6, 125.5, 125.3, 115.6, 115.5, 114.7, 114.6, 57.7, 47.6, 44.6, 37.80, 37.76, 34.6, 34.3, 34.2, 33.3, 20.60, 20.58, 15.44, 15.38.

HRMS (ESI): *m/z* calculated for $C_{43}H_{44}N^+$ ($[M+H]^+$): 573.3468, found: 573.3461.

2,4,6-Triphenyl-1-(4-phenylbutan-2-yl)-4-(1-phenylbutyl)-1,4-dihydropyridine **7**:

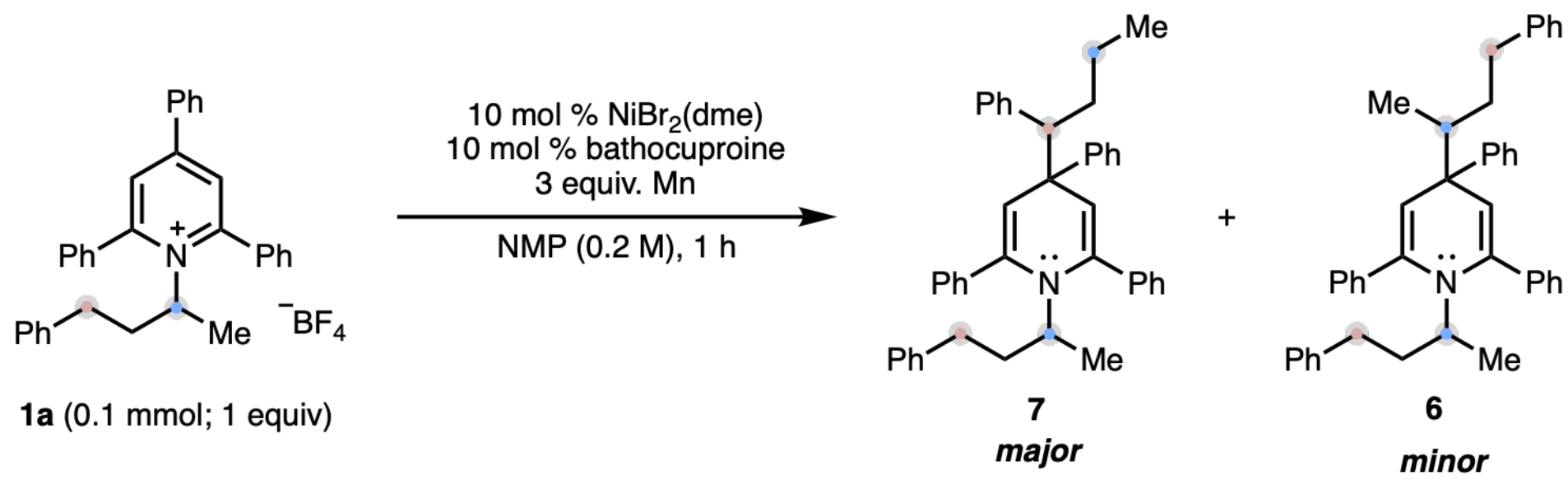

Inside a nitrogen-filled glovebox, a 1-dram vial equipped with a PTFE-coated magnetic stir bar was charged with 2,4,6-triphenyl-1-(4-phenylbutan-2-yl)pyridin-1-ium tetrafluoroborate **1a** (52.7 mg, 0.100 mmol, 1.00 equiv), manganese powder (16.5 mg, 0.300 mmol, 3.00 equiv), and 0.500 mL NMP. The vial was then hermetically sealed with a syringeable cap (PP/PTFE/silicone liner septum) and stirred at room temperature for 1 hour at 1500 RPM. The vial was then removed from the glovebox, diluted with ethyl acetate (approximately 2 mL), and then washed with 10% LiCl (aq.) solution (approximately 1 mL) and brine (approximately 1 mL). The organic layer was dried over Na_2SO_4, filtered, and concentrated *in vacuo* to obtain crude reaction mixture. The crude rection mixture was purified by flash column chromatography using 100:0 → 90:10 hexane/diethyl ether as an eluent followed by preparative thin-layer chromatography (95:5 pentane/diethyl ether) to yield 2,4,6-triphenyl-1-(4-phenylbutan-2-yl)-4-(1-phenylbutyl)-1,4-dihydropyridine **7** (white solid, 42% isolated yield) as a mixture of diastereomers (1:1 dr) in a 16:1 regioisomeric ratio of **7**/**6**.

^{1}**H NMR** (500 MHz, $CDCl_3$): δ 7.62 – 7.52 (m, 4H), 7.52 – 7.46 (m, 4H), 7.43 – 7.31 (m, 14H), 7.28 – 7.19 (m, 8H, overlaps with $CHCl_3$), 7.14 – 7.05 (m, 12H), 6.85 – 6.82 (m, 4H), 6.61 – 6.59 (m, , 4H), 5.42 (d, *J* = 2.1 Hz, 2H), 5.39 (d, *J* = 2.1 Hz, 1H), 5.37 (d, *J* =

2.1 Hz, 1H), 3.12 – 3.05 (m, 2H), 2.96 – 2.92 (m, 2H), 2.16 – 2.06 (m, 4H), 1.83 – 1.63 (m, 4H), 1.50 – 1.41 (m, 2H), 1.15 – 0.93 (m, 6H), 0.76 – 0.73 (m, 6H), 0.61 (d, J = 7.0 Hz, 3H), 0.58 (d, J = 7.0 Hz, 3H).

^{13}C NMR (126 MHz, $CDCl_3$): δ 151.1, 151.0, 145.5, 145.3, 144.6, 144.3, 130.1, 130.0, 128.8, 128.18, 128.15, 128.11, 128.03, 127.98, 127.93, 127.91, 127.86, 127.76, 127.72, 127.69, 127.67, 127.62, 127.61, 127.18, 127.17, 126.94, 126.91, 125.69, 125.68, 125.3, 125.2, 116.9, 115.6, 114.6, 113.2, 58.60, 58.58, 57.43, 57.42, 47.75, 47.71, 37.6, 37.4, 33.11, 33.08, 32.9, 32.8, 21.2, 20.1, 20.3, 14.09, 14.08.

HRMS (ESI): *m/z* calculated for $C_{43}H_{44}N^+$ ($[M+H]^+$): 573.3468, found: 573.3458.

6. Evaluation of Substrate Scope for Nickel-Catalyzed Migratory Arylation of Aliphatic Amines

6.1 General Procedure 1 for Evaluation of (Hetero)Aryl Bromides for Nickel-Catalyzed Migratory Arylation of Aliphatic Amines

6.1.1 *Method A* (for electron-rich and electron-neutral aryl bromides):

6.1.1.1 Preparation of a stock solution of the precatalyst (0.02 M):

Inside a nitrogen-filled glovebox, a 1-dram vial equipped with a PTFE-coated magnetic stir bar was charged with $NiBr_2$(dme) (10 mol %), bathocuproine (10 mol %), and NMP (0.02 M). The vial was hermetically sealed with a syringeable cap (PP/PTFE/silicone liner septum), and the resulting solution was stirred vigorously for 15 min. The formation of a homogeneous "process red" (PC 994) solution indicated the generation of the bathocuproine-ligated nickel precatalyst. The precatalyst vial was then removed from the glovebox for use in the reaction setup described below.

6.1.1.2 Reaction setup:

Inside a nitrogen-filled glovebox, a 1-dram vial equipped with a PTFE-coated magnetic stir bar was charged with $[Ir(ppy)_2(dtbbpy)]PF_6$ (2.70 mg, 0.003 mmol, 3.00 mol %), followed by 2,4,6-triphenyl-1-(4-phenylbutan-2-yl)pyridin-1-ium tetrafluoroborate **1a** (52.7 mg, 0.100 mmol, 1.00 equiv) and manganese powder (16.5 mg, 0.300 mmol, 3.00 equiv). (Note: If aryl bromide or heteroaryl bromide was solid, it was added to the vial at this point.) The vial was then hermetically sealed with a syringeable cap (PP/PTFE/silicone liner septum) and removed from the glovebox. Under a positive nitrogen pressure, the reaction vial was charged with aryl bromide or heteroaryl bromide (0.200 mmol, 2.00 equiv) and *n*-propyl bromide (9.00 µL, 0.100 mmol, 1.00 equiv), followed by 0.500 mL of the precatalyst solution (described in 6.1.1.1). The punctured cap was then sealed with M grease, secured with electrical tape, and irradiated for 4 hours with tuna blue Kessil lamps with maximum intensity, fan speed, and stir rate. After completion of the reaction, the mixture was diluted with ~2 mL ethyl acetate followed by ~1 mL of 10% aq. LiCl solution, gently shaken, and allowed to settle. The biphasic solution was then passed through a short pad of Celite® into a scintillation vial, rinsing further with

ethyl acetate to minimize the loss of material. The aqueous layer was decanted, and the organic layer was washed with ~1 mL of brine, shaken, and allowed to settle. After decanting the aqueous layer, the organic layer was dried over Na_2SO_4, filtered through a short pad of Celite® topped with Na_2SO_4, and concentrated under reduced pressure to obtain crude reaction mixture. The crude reaction mixture was further purified by flash column chromatography and/or preparative thin layer chromatography.

6.1.2 *Method B* (for electron-deficient aryl bromides):

6.1.2.1 <u>*Preparation of a precatalyst stock solution (0.02 M)*</u>:

Inside a nitrogen-filled glovebox, a 1-dram vial equipped with a PTFE-coated magnetic stir bar was charged with $NiBr_2$(dme) (10 mol %), 6,6′-Me_2bpy (10 mol %), and NMP (0.02 M). The vial was hermetically sealed with a syringeable cap (PP/PTFE/silicone liner septum), and the resulting solution was stirred vigorously for 15 min. The formation of a homogeneous “process red” (PC 994) solution indicated the generation of the 6,6′-Me_2bpy-ligated nickel precatalyst. The precatalyst solution was then used inside the glovebox for reaction setup described below.

6.1.2.2 <u>*Reaction setup*</u>:

Inside a nitrogen-filled glovebox, a 1-dram vial equipped with a PTFE-coated magnetic stir bar was charged with 2,4,6-triphenyl-1-(4-phenylbutan-2-yl)pyridin-1-ium tetrafluoroborate **1a** (52.7 mg, 0.100 mmol, 1.00 equiv) and manganese powder (16.5 mg, 0.300 mmol, 3.00 equiv). To this vial, 0.500 mL of the precatalyst solution, described in 6.1.2.1, was added. The vial was then hermetically sealed with a syringeable cap (PP/PTFE/silicone liner septum) and stirred at 1500 RPM for 1 h inside the glovebox. The reaction vial was charged with $[Ir(ppy)_2(dtbbpy)]PF_6$ (2.70 mg, 0.003 mmol, 3.00 mol %) and aryl bromide or heteroaryl bromide (0.200 mmol, 2.00 equiv). The vial was then hermetically sealed with a syringeable cap and removed from the glovebox. Under a positive nitrogen pressure, the reaction vial was charged with *n*-propyl bromide (9.00 µL, 0.100 mmol,1.00 equiv). The punctured cap was then sealed with M grease, secured with electrical tape, and irradiated for 4 h with tuna blue Kessil lamps with maximum intensity, fan speed, and stir rate. After completion of the reaction, the mixture was diluted with ~2

mL ethyl acetate followed by ~1 mL of 10% aq. LiCl solution, gently shaken, and allowed to settle. The biphasic solution was then passed through a short pad of Celite® into a scintillation vial, rinsing further with ethyl acetate to minimize the loss of material. The aqueous layer was decanted, and the organic layer was washed with ~1 mL of brine, shaken, and allowed to settle. After decanting the aqueous layer, the organic layer was dried over Na_2SO_4, filtered through a short pad of Celite® topped with Na_2SO_4, and concentrated under reduced pressure to obtain crude reaction mixture. The crude reaction mixture was further purified by flash column chromatography and/or preparative thin layer chromatography.

6.2 General Procedure 2 for Evaluation of Katritzky Salts for Nickel-Catalyzed Migratory Arylation of Aliphatic Amines

6.2.1 *Method A*

6.2.1.1 <u>*Preparation of a stock solution of the precatalyst (0.02 M)*</u>:

Inside a nitrogen-filled glovebox, a 1-dram vial equipped with a PTFE-coated magnetic stir bar was charged with $NiBr_2$(dme) (10 mol %), bathocuproine (10 mol %), and NMP (0.02 M). The vial was hermetically sealed with a syringeable cap (PP/PTFE/silicone liner septum), and the resulting solution was stirred vigorously for 15 min. The formation of a homogeneous "process red" (PC 994) solution indicated the generation of the bathocuproine-ligated nickel precatalyst. The precatalyst vial was then removed from the glovebox for use in the reaction setup described below.

6.2.1.2 <u>*Reaction setup*</u>:

Inside a nitrogen-filled glovebox, a 1-dram vial equipped with a PTFE-coated magnetic stir bar was charged with $[Ir(ppy)_2(dtbbpy)]PF_6$ (2.70 mg, 0.003 mmol, 3.00 mol %), followed by Katritzky salt (0.100 mmol, 1.00 equiv) and manganese powder (16.5 mg, 0.300 mmol, 3.00 equiv). The vial was then hermetically sealed with a syringeable cap (PP/PTFE/silicone liner septum) and removed from the glovebox. Under a positive nitrogen pressure, the reaction vial was charged with 1-bromo-4-fluorobenzene **2a** (22.0 µL, 0.200 mmol, 2.00 equiv) and *n*-propyl bromide (9.00 µL, 0.100 mmol, 1.00

equiv), followed by 0.500 mL of the precatalyst solution (described in 6.2.1). The punctured cap was then sealed with M grease, secured with electrical tape, and irradiated for 4 h with tuna blue Kessil lamps with maximum intensity, fan speed, and stir rate. After completion of the reaction, the mixture was diluted with ~2 mL ethyl acetate followed by ~1 mL of 10% aq. LiCl solution, gently shaken, and allowed to settle. The biphasic solution was then passed through a short pad of Celite® into a scintillation vial, rinsing further with ethyl acetate to minimize the loss of material. The aqueous layer was decanted, and the organic layer was washed with ~1 mL of brine, shaken, and allowed to settle. After decanting the aqueous layer, the organic layer was dried over Na_2SO_4, filtered through a short pad of Celite® topped with Na_2SO_4, and concentrated under reduced pressure to obtain crude reaction mixture. The crude reaction mixture was further purified by flash column chromatography and/or preparative thin layer chromatography.

6.2.2 *Method C (For Katritzky Salts derived from α-primary amines)*

6.2.2.1 *Preparation of a stock solution of the precatalyst (0.02 M)*:

Inside a nitrogen-filled glovebox, a 1-dram vial equipped with a PTFE-coated magnetic stir bar was charged with $NiBr_2$(dme) (10 mol %), bathocuproine (10 mol %), and NMP (0.02 M). The vial was hermetically sealed with a syringeable cap (PP/PTFE/silicone liner septum), and the resulting solution was stirred vigorously for 15 min. The formation of a homogeneous "process red" (PC 994) solution indicated the generation of the bathocuproine-ligated nickel precatalyst. The precatalyst vial was then removed from the glovebox for use in the reaction setup described below.

6.2.2.2 *Reaction setup*:

Inside a nitrogen-filled glovebox, a 1-dram vial equipped with a PTFE-coated magnetic stir bar was charged with **Acridin** (0.050 mmol, 1.00 equiv) and manganese powder (8.25 mg, 0.150 mmol, 3.00 equiv). To this vial, 0.500 mL of the precatalyst solution, described in 6.2.2.1, was added. The vial was then hermetically sealed with a syringeable cap (PP/PTFE/silicone liner septum), removed from the glovebox and stirred vigorously at 50

ºC for 1 h. The reaction vial was brought back into the glovebox and charged with $[Ir(ppy)_2(dtbbpy)]PF_6$ (1.35 mg, 0.0015 mmol, 3.00 mol %). (Note: If aryl bromide or heteroaryl bromide was solid, it was added to the vial at this point.) The vial was then hermetically sealed with a syringeable cap and removed from the glovebox. Under a positive nitrogen pressure, the reaction vial was charged with aryl bromide or heteroaryl bromide (0.150 mmol, 3.00 equiv), *n*-propyl bromide (4.50 µL, 0.050 mmol, 1.00 equiv) and diisopropylamine (7.00 µL, 0.050 mmol, 1.00 equiv). The punctured cap was then sealed with M grease, secured with electrical tape, and irradiated for 12 h in a Hepatochem Lucent 360 photoreactor (425 nm) at 1100 RPM. A temperature of 60°C was maintained throughout the reaction with the use of an external Julabo chiller connected to the photoreactor water bath. After completion of the reaction, the mixture was diluted with ~2 mL ethyl acetate followed by ~1 mL of 10% aq. LiCl solution, gently shaken, and allowed to settle. The biphasic solution was then passed through a short pad of Celite® into a scintillation vial, rinsing further with ethyl acetate to minimize the loss of material. The aqueous layer was decanted, and the organic layer was washed with ~1 mL of brine, shaken, and allowed to settle. After decanting the aqueous layer, the organic layer was dried over Na_2SO_4, filtered through a short pad of Celite® topped with Na_2SO_4, and concentrated under reduced pressure to obtain crude reaction mixture. The crude reaction mixture was further purified by flash column chromatography and/or preparative thin layer chromatography.

6.3 Characterization *of Nickel-Catalyzed Migratory Arylation of Aliphatic Amines Products*

1-Fluoro-4-(1-phenylbutyl)benzene **3a**:

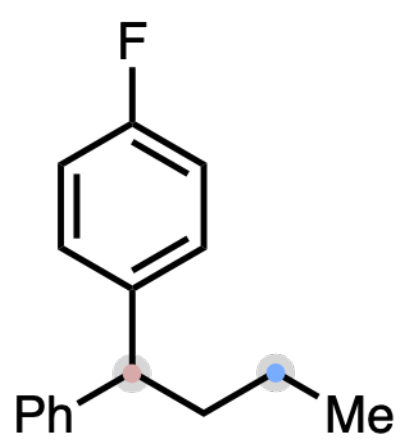

3a

The titled compound **3a** was prepared according to *Method A* described in *General Procedure 1* from 2,4,6-triphenyl-1-(4-phenylbutan-2-yl)pyridin-1-ium tetrafluoroborate **1a** (52.7 mg, 0.100 mmol, 1.00 equiv) and 1-bromo-4-fluorobenzene (22.0 µL, 0.200 mmol, 2.00 equiv). The regioselectivity (>20:1 rr) of the reaction was determined by ^{19}F spectroscopic analysis of the crude reaction mixture. The crude reaction mixture was purified by flash column chromatography using 100:0 → 97:3 hexane/diethyl ether as an eluent followed by preparative thin layer chromatography (99:1 pentane/diethyl ether) to yield compound **3a** as a white solid in 79% yield (18.0 mg, 0.079 mmol). NMR spectra are in accordance with those reported in the literature.[11]

^{1}H NMR (300 MHz, $CDCl_3$): δ 7.32 – 7.25 (m, 2H), 7.24 – 7.14 (m, 5H), 7.00 – 6.91 (m, 2H), 3.90 (t, *J* = 7.7 Hz, 1H), 2.08 – 1.91 (m, 2H), 1.28 (h, *J* = 7.7 Hz, 2H), 0.93 (t, *J* = 7.7 Hz, 3H).

^{13}C NMR (126 MHz, $CDCl_3$): δ 161.4 (d, *J* = 243.9 Hz), 145.3, 141.2 (d, *J* = 3.2 Hz), 129.3 (d, *J* = 7.8 Hz), 128.6, 127.9, 126.3, 115.2 (d, *J* = 21.0 Hz), 50.4, 38.2, 21.2, 14.2.

^{19}F NMR (282 MHz, $CDCl_3$): δ -117.6.

HRMS (EI): *m/z* calculated for $C_{16}H_{17}F^+$ ($[M]^+$): 228.1314, found: 228.1318.

N,*N*-Dimethyl-4-(1-phenylbutyl)aniline **3b**:

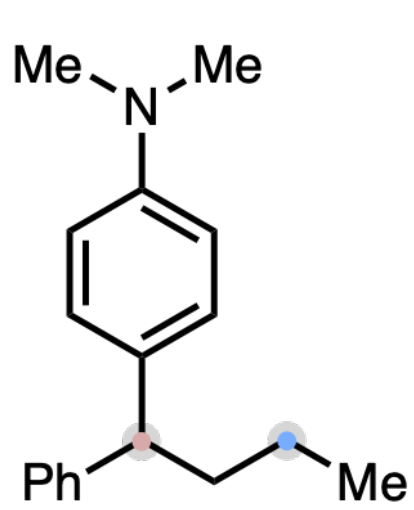

3b

The titled compound **3b** was prepared according to *Method A* described in *General Procedure 1* from 2,4,6-triphenyl-1-(4-phenylbutan-2-yl)pyridin-1-ium tetrafluoroborate **1a** (52.7 mg, 0.100 mmol, 1.00 equiv) and 4-bromo-*N*,*N*-dimethylaniline (40.0 mg, 0.200 mmol, 2.00 equiv). (*Note*: The regioselectivity (20:1 rr) of the reaction was determined by

^{1}H spectroscopic analysis of the crude reaction mixture of a duplicate run, which passed through a short silica plug, eluted with 7:3 hexane/ethyl acetate, and concentrated *in vacuo*.) The crude reaction mixture was purified by flash column chromatography using 100:0 → 90:10 hexane/diethyl ether as an eluent followed by preparative thin layer chromatography (97:3 pentane/diethyl ether) to yield compound **3b** as a colorless oil in 52% yield (13.2 mg, 0.052 mmol). NMR spectra are in accordance with those reported in the literature.[12]

^{1}H NMR (400 MHz, $CDCl_3$): δ 7.30 – 7.24 (m, 4H, overlaps with $CHCl_3$), 7.18 – 7.15 (m, 1H), 7.13 (d, J = 8.9 Hz, 2H), 6.70 (d, J = 8.9 Hz, 2H), 3.85 (t, J = 7.8 Hz, 1H), 2.93 (s, 6H), 2.06 – 1.96 (m, 2H), 1.37 – 1.25 (m, 2H), 0.94 (t, J = 7.3 Hz, 3H).

^{13}C NMR (126 MHz, $CDCl_3$): δ 149.1, 146.4, 133.7, 128.6, 128.4, 127.9, 125.8, 112.9, 50.2, 40.9, 38.3, 21.4, 14.2.

HRMS (EI): *m/z* calculated for $C_{18}H_{23}N^+$ ($[M]^+$): 253.1830, found: 253.1828.

1-Methoxy-4-(1-phenylbutyl)benzene **3c**:

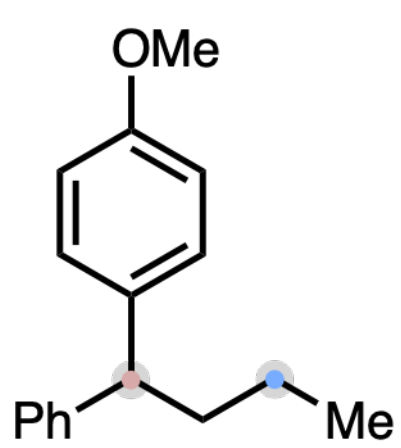

3c

The titled compound **3c** was prepared according to *Method A* described in *General Procedure 1* from 2,4,6-triphenyl-1-(4-phenylbutan-2-yl)pyridin-1-ium tetrafluoroborate **1a** (52.7 mg, 0.100 mmol, 1.00 equiv) and 1-bromo-4-methoxybenzene (37.0 mg, 0.200 mmol, 2.00 equiv). (*Note*: The regioselectivity (>20:1 rr) of the reaction was determined by ^{1}H spectroscopic analysis of the crude reaction mixture of a duplicate run, which passed through a short silica plug, eluted with 7:3 hexane/ethyl acetate, and concentrated *in vacuo*.) The crude reaction mixture was purified by flash column chromatography using 100:0 → 98:2 hexane/ethyl acetate as an eluent to yield compound **3c** as a colorless oil in 86% yield (20.6 mg, 0.086 mmol). NMR spectra are in accordance with those reported in the literature.[12]

^{1}**H NMR** (500 MHz, $CDCl_3$): δ 7.29 – 7.25 (m, 2H), 7.21 – 7.19 (m, 2H), 7.15 – 7.12 (m, 3H), 6.82 (dt, *J* = 8.7, 3.0 Hz, 2H), 3.87 (t, *J* = 7.8 Hz, 1H), 3.78 (s, 3H), 2.00 (q, *J* = 7.8 Hz, 2H), 1.34 – 1.22(m, 2H), 0.93 (t, *J* = 7.3 Hz, 3H).

^{13}**C NMR** (126 MHz, $CDCl_3$): δ 157.9, 145.9, 137.7, 128.9, 128.5, 127.9, 126.0, 113.9, 55.3, 50.3, 38.2, 21.3, 14.2.

HRMS (EI): *m/z* calculated for $C_{17}H_{20}O^+$ ($[M]^+$): 240.1514, found: 240.1518.

Methyl(4-(1-phenylbutyl)phenyl)sulfane **3d**:

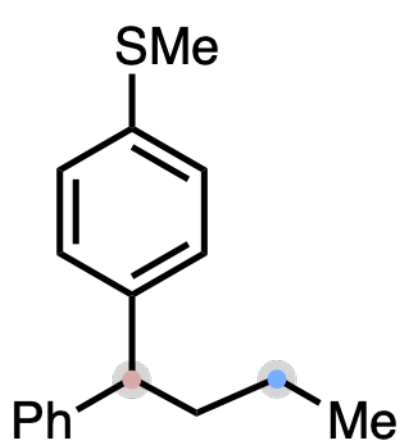

3d

The titled compound **3d** was prepared according to *Method A* described in *General Procedure 1* from 2,4,6-triphenyl-1-(4-phenylbutan-2-yl)pyridin-1-ium tetrafluoroborate **1a** (52.7 mg, 0.100 mmol, 1.00 equiv) and 4-bromothioanisole (40.6 mg, 0.200 mmol, 2.00 equiv). (*Note*: The regioselectivity (>20:1 rr) of the reaction was determined by ^{1}H spectroscopic analysis of the crude reaction mixture of a duplicate run, which passed through a short silica plug, eluted with 7:3 hexane/ethyl acetate, and concentrated *in vacuo*.) The crude reaction mixture was purified by flash column chromatography using 99:1 hexane/ethyl acetate as an eluent followed by preparative thin layer chromatography (100% pentane) to yield compound **3d** as a colorless oil in 62% yield (15.9 mg, 0.062 mmol).

^{1}**H NMR** (500 MHz, $CDCl_3$): δ 7.27 (t, *J* = 7.4 Hz, 2H, overlaps with $CHCl_3$), 7.23 – 7.21 (m, 2H), 7.19 – 7.15 (m, 5H), 3.87 (t, *J* = 7.8 Hz, 1H), 2.45 (s, 3H), 2.00 (q, *J* = 7.8 Hz, 2H), 1.28 (h, *J* = 7.8 Hz, 2H), 0.92 (t, *J* = 7.8 Hz, 3H).

^{13}**C NMR** (126 MHz, $CDCl_3$): δ 145.3, 141.6, 135.7, 128.6, 128.5, 127.9, 127.2, 126.2, 50.7, 38.0, 21.3, 16.3, 14.2.

HRMS (EI): *m/z* calculated for $C_{17}H_{20}S^+$ ($[M]^+$): 256.1286, found: 256.1291.

1-(1-Phenylbutyl)-4-(trifluoromethyl)benzene **3e**:

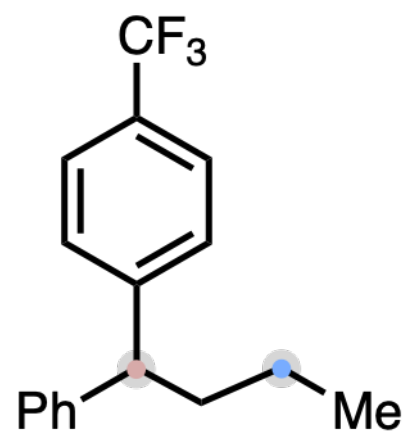

3e

The titled compound **3e** was prepared according to *Method B* described in *General Procedure 1* from 2,4,6-triphenyl-1-(4-phenylbutan-2-yl)pyridin-1-ium tetrafluoroborate **1a** (52.7 mg, 0.100 mmol, 1.00 equiv) and 1-bromo-4-(trifluoromethyl)benzene (45.0 mg, 0.200 mmol, 2.00). (*Note*: The regioselectivity (20:1 rr) of the reaction was determined by ^{1}H spectroscopic analysis of the crude reaction mixture of a duplicate run, which passed through a short silica plug, eluted with hexane, and concentrated *in vacuo*.) The crude reaction mixture was purified by flash column chromatography using 100% hexane as an eluent to yield compound **3e** as a white solid in 61% yield (17.0 mg, 0.061 mmol). NMR spectra are in accordance with those reported in the literature.[13]

^{1}H NMR (300 MHz, CDCl$_3$): δ 7.52 (d, *J* = 8.1 Hz, 2H), 7.37 – 7.27 (m, 4H), 7.24 – 7.16 (m, 3H), 3.97 (t, *J* = 7.8 Hz, 1H), 2.03 (qd, *J* = 7.3, 2.1 Hz, 2H), 1.37 – 1.16 (m, 2H), 0.93 (t, *J* = 7.3 Hz, 3H).

^{13}C NMR (126 MHz, CDCl$_3$): δ 149.6 (q, *J* = 1.4 Hz), 144.4, 128.7, 128.4 (q, *J* = 32.5 Hz), 128.3, 128.0, 126.6, 125.5 (q, *J* = 3.8 Hz), 124.4 (q, *J* = 271.8 Hz), 51.1, 37.8, 21.2, 14.2.

^{19}F NMR (282 MHz, CDCl$_3$): δ -62.34.

HRMS (EI): *m/z* calculated for $C_{17}H_{17}F_3^+$ ([M]$^+$): 278.1282, found: 278.1278.

1-Chloro-4-(1-phenylbutyl)benzene **3f**:

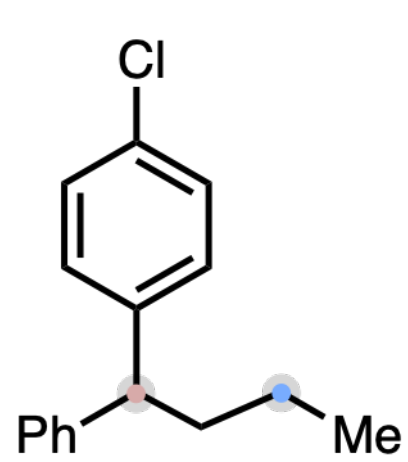

3f

The titled compound **3f** was prepared according to *Method B* described in *General Procedure 1* from 2,4,6-triphenyl-1-(4-phenylbutan-2-yl)pyridin-1-ium tetrafluoroborate **1a** (52.7 mg, 0.100 mmol, 1.00 equiv) and 1-bromo-4-chlorobenzene (38.2 mg, 0.200 mmol, 2.00 equiv). (*Note*: The regioselectivity (>20:1 rr) of the reaction was determined by ^{1}H spectroscopic analysis of the crude reaction mixture of a duplicate run, which passed through a short silica plug, eluted with 9:1 hexane/ethyl acetate, and concentrated *in*

vacuo.) The crude reaction mixture was purified by flash column chromatography using 100:0 → 97:3 hexane/ethyl acetate as an eluent followed by preparative HPLC (XBridge BEH C18 OBD column, 75% to 95% MeCN in H_2O with 0.1% NH_4OH) to yield compound **3f** as a colorless oil in 50% yield (12.2 mg, 0.050 mmol). NMR spectra are in accordance with those reported in the literature.[13]

^{1}H NMR (500 MHz, $CDCl_3$): δ 7.30 – 7.24 (m, 3H, overlaps with $CHCl_3$), 7.23 – 7.14 (m, 6H), 3.88 (t, *J* = 7.8 Hz, 1H), 1.99 (qd, *J* = 7.5, 2.1 Hz, 2H), 1.28 (h, *J* = 7.3 Hz, 2H, overlaps with grease), 0.92 (t, *J* = 7.3 Hz, 3H).

^{13}C NMR (126 MHz, $CDCl_3$): δ 144.9, 144.0, 131.8, 129.4, 128.61, 128.62 127.9, 126.4, 50.6, 37.9, 21.2, 14.2.

HRMS (EI): *m/z* calculated for $C_{16}H_{17}Cl^+$ ($[M]^+$): 244.1019, found: 244.1014.

4-(1-Phenylbutyl)benzaldehyde **3g**:

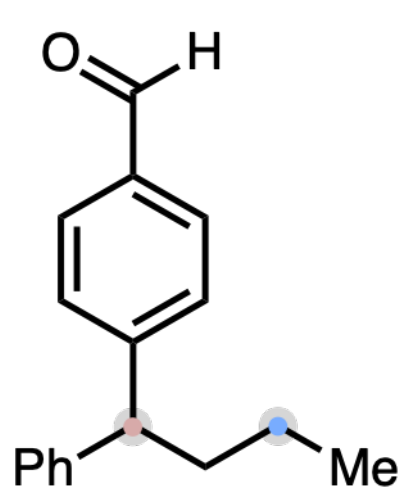

3g

The titled compound **3g** was prepared according to *Method B* described in *General Procedure 1* from 2,4,6-triphenyl-1-(4-phenylbutan-2-yl)pyridin-1-ium tetrafluoroborate **1a** (52.7 mg, 0.100 mmol, 1.00 equiv) and 4-bromobenzaldehyde (37.0 mg, 0.200 mmol, 2.00 equiv). (*Note*: The regioselectivity (5:1 rr) of the reaction was determined by ^{1}H spectroscopic analysis of the crude reaction mixture of a duplicate run, which passed through a short silica plug, eluted with 7:3 hexane/ethyl acetate, and concentrated *in vacuo*.) The crude reaction mixture was purified by flash column chromatography using 100:0 → 90:10 hexane/ethyl acetate as an eluent followed by preparative thin-layer chromatography (90:10 pentane/diethyl ether) to yield compound **3g** as a colorless oil in 59% yield (14.0 mg, 0.059 mmol) in 16:1 regioisomeric ratio. NMR spectra are in accordance with those reported in the literature.[14]

^{1}H NMR (500 MHz, $CDCl_3$): δ 9.96 (s, 1H), 7.79 (d, *J* = 8.2 Hz, 2H), 7.41 (d, *J* = 8.2 Hz, 2H), 7.29 (t, *J* = 7.6 Hz, 2H), 7.24 – 7.17 (m, 3H), 3.99 (t, *J* = 7.8 Hz, 1H), 2.11 – 2.00 (qd, *J* = 7.9, 4.6 Hz, 2H), 1.34 – 1.22 (m, 2H), 0.94 (t, *J* = 7.4 Hz, 3H).

^{13}C NMR (126 MHz, $CDCl_3$): δ 192.1, 152.8, 144.2, 134.8, 130.1, 128.74, 128.70, 128.0, 126.6, 51.4, 37.7, 21.2, 14.2.

HRMS (ESI): *m/z* calculated for $C_{17}H_{19}O^+$ ($[M + H]^+$): 239.1431, found: 239.1430.

1-(4-(1-Phenylbutyl) phenyl)ethan-1-one **3h**:

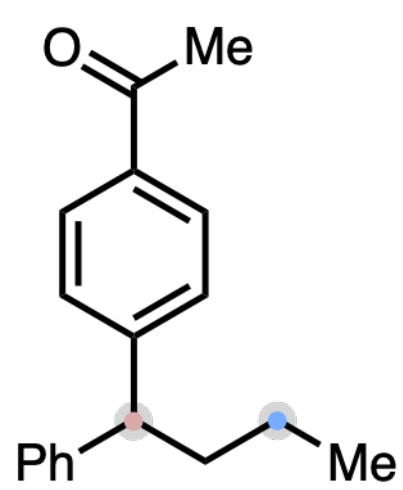

3h

The titled compound **3h** was prepared according to *Method B* described in *General Procedure 1* from 2,4,6-triphenyl-1-(4-phenylbutan-2-yl)pyridin-1-ium tetrafluoroborate **1a** (52.7 mg, 0.100 mmol, 1.00 equiv) and 1-(4-bromophenyl)ethan-1-one (39.8 mg, 0.200 mmol, 2.00 equiv). (*Note*: The regioselectivity (>20:1 rr) of the reaction was determined by ^{1}H spectroscopic analysis of the crude reaction mixture of a duplicate run, which passed through a short silica plug, eluted with 7:3 hexane/ethyl acetate, and concentrated *in vacuo*.) The crude reaction mixture was purified by flash column chromatography using 95:5 hexane/ethyl acetate as an eluent followed by preparative HPLC (XBridge BEH C18 OBD column, 75% to 95% MeCN in H_2O with 0.1% NH_4OH) to yield compound **3h** as a colorless oil in 63% yield (15.9 mg, 0.063 mmol) as a mixture of regioisomers (15:1 regioisomeric ratio of **3h**/**4h**). NMR spectra are in accordance with those reported in the literature.[14]

^{1}H NMR (500 MHz, $CDCl_3$): δ 7.87 (dt, *J* = 8.4, 1.7 Hz, 2H), 7.33 (d, *J* = 8.4 Hz, 2H), 7.28 (tt, *J* = 7.7, 3.0 Hz, 2H), 7.24 – 7.20 (m, 2H), 7.18, (tt, 7.2, 1.3 Hz, 1H), 3.97 (t, *J* = 7.8 Hz, 1H), 2.56 (s, 3H), 2.04 (qd, *J* = 7.2, 2.3 Hz, 2H), 1.30 (hd, *J* = 7.3, 1.8 Hz, 2H), 0.93 (t, *J* = 7.3 Hz, 3H).

^{13}C NMR (126 MHz, $CDCl_3$): δ 197.9, 151.2, 144.4, 135.3, 128.74, 128.68, 128.2, 128.0, 126.5, 51.2, 37.7, 26.7, 21.2, 14.2.

HRMS (ESI): *m/z* calculated for $C_{18}H_{21}O^+$ ([M + H]$^+$): 253.1587, found: 253.1596.

Methyl 4-(1-phenylbutyl)benzoate **3i**:

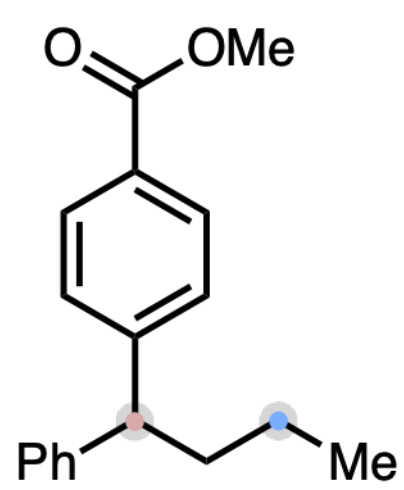

3i

The titled compound **3i** was prepared according to *Method B* described in *General Procedure 1* from 2,4,6-triphenyl-1-(4-phenylbutan-2-yl)pyridin-1-ium tetrafluoroborate **1a** (52.7 mg, 0.100 mmol, 1.00 equiv) and methyl 4-bromobenzoate **2i** (43.0 mg, 0.200 mmol, 2.00 equiv). (*Note*: The regioselectivity (15:1 rr) of the reaction was determined by ^{1}H spectroscopic analysis of the crude reaction mixture of a duplicate run, which passed through a short silica plug, eluted with 7:3 hexane/ethyl acetate, and concentrated *in vacuo*.) The crude reaction mixture was purified by flash column chromatography using 100:0 → 97:3 hexane/diethyl ether as an eluent followed by preparative thin layer chromatography (98:2 pentane/diethyl ether) to yield compound **3i** as a colorless oil in 74% yield (19.8 mg, 0.074 mmol). NMR spectra are in accordance with those reported in the literature.[14]

^{1}H NMR (500 MHz, $CDCl_3$): δ 7.95 (d, J = 8.4 Hz, 2H), 7.31 (d, J = 8.4 Hz, 2H), 7.28 (d, J = 7.6 Hz, 2H), 7.23 (d, J = 7.0 Hz, 2H), 7.19 (td, J = 7.0, 1.5 Hz, 1H), 3.97 (t, J = 7.8 Hz, 1H), 3.89 (s, 3H), 2.04 (qd, J = 7.1, 1.8 Hz, 2H), 1.35 – 1.23 (m, 2H, overlaps with grease), 0.93 (t, J = 7.4 Hz, 3H).

^{13}C NMR (126 MHz, $CDCl_3$): δ 167.2, 150.9, 144.5, 129.9, 128.6, 128.5, 128.1, 128.0, 126.4, 52.1, 51.2, 37.8, 21.2, 14.2.

HRMS (EI): *m/z* calculated for $C_{18}H_{20}O_2^+$ ([M]$^+$): 268.1463, found: 268.1469.

N,*N*-Dimethyl-4-(1-phenylbutyl)benzenesulfonamide **3j**:

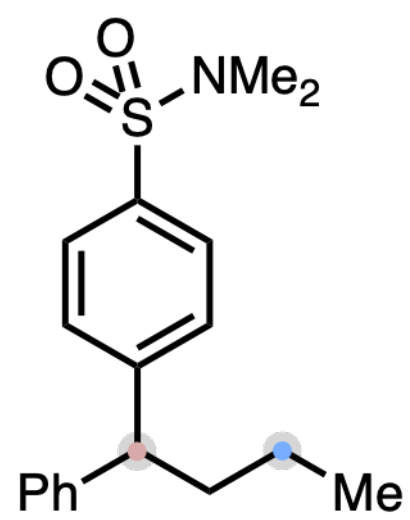

3j

The titled compound **3j** was prepared according to *Method B* described in *General Procedure 1* from 2,4,6-triphenyl-1-(4-phenylbutan-2-yl)pyridin-1-ium tetrafluoroborate **1a** (52.7 mg, 0.100 mmol, 1.00 equiv) and methyl 4-bromo-*N*,*N*-dimethylbenzenesulfonamide (52.8 mg, 0.200 mmol, 2.00 equiv). (*Note*: The regioselectivity (11:1 rr) of the reaction was determined by ^{1}H spectroscopic analysis of the crude reaction mixture of a duplicate run, which passed through a short silica plug, eluted with 7:3 hexane/ethyl acetate, and concentrated *in vacuo*.) The crude reaction mixture was purified by flash column chromatography using 100:0 → 75:25 hexane/diethyl ether as an eluent followed by preparative thin-layer chromatography (80:20 pentane/diethyl ether) to yield compound **3j** as a white solid in 63% yield (20.0 mg, 0.063 mmol) as a mixture of regioisomers (8:1 regioisomeric ratio of **3j**/**4j**).

^{1}H NMR (500 MHz, $CDCl_3$): δ 7.68 (d, *J* = 8.4 Hz, 2H), 7.40 (d, *J* = 8.4 Hz, 2H), 7.30 (t, *J* = 7.5 Hz, 2H), 7.25 – 7.17 (m, 3H), 3.98 (t, *J* = 7.8 Hz, 1H), 2.69 (s, 6H), 2.11 – 1.98 (m, 2H), 1.34 – 1.20 (m, 2H), 0.93 (t, *J* = 7.3 Hz, 3H).

^{13}C NMR (126 MHz, $CDCl_3$): δ 150.8, 144.0, 133.3, 128.8, 128.6, 128.1, 128.0, 126.7, 51.2, 38.1, 37.8, 21.2, 14.1.

HRMS (EI): *m/z* calculated for $C_{18}H_{23}NO_2S^+$ ($[M]^+$): 317.1449, found: 317.1437.

(4-(1-Phenylbutyl)phenyl)methanol **3k**:

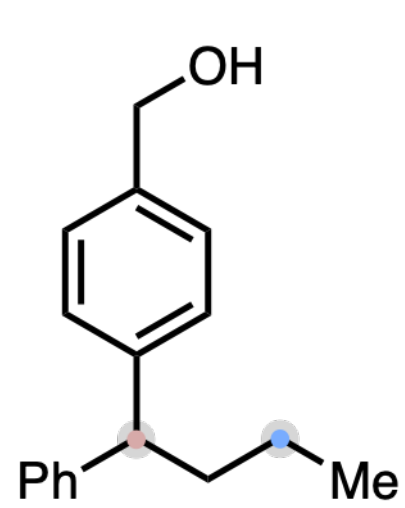

3k

The titled compound **3k** was prepared according to *Method A* described in *General Procedure 1* from 2,4,6-triphenyl-1-(4-phenylbutan-2-yl)pyridin-1-ium tetrafluoroborate **1a** (52.7 mg, 0.100 mmol, 1.00 equiv) and (4-bromophenyl)methanol **2k** (37.4 mg, 0.200 mmol, 2.00 equiv). (*Note*: The regioselectivity (>20:1 rr) of the reaction was determined by ^{1}H spectroscopic analysis of the crude reaction mixture of a duplicate run, which passed through a short silica plug, eluted with 7:3 hexane/ethyl acetate, and concentrated *in vacuo*.) The crude reaction mixture was purified by flash column

chromatography using 100:0 → 90:10 hexane/diethyl ether as an eluent followed by preparative thin-layer chromatography (80:20 pentane/diethyl ether) to yield compound **3k** as a colorless oil in 67% yield (16.0 mg, 0.067 mmol). NMR spectra are in accordance with those reported in the literature.[15]

^{1}H NMR (500 MHz, $CDCl_3$): δ 7.30 – 7.22 (m, 8H), 7.17 (tt, *J* = 7.1, 1.6 Hz, 1H), 4.64 (s, 2H), 3.92 (t, *J* = 7.8 Hz, 1H), 2.03 (qt, *J* = 7.7, 2.4 Hz, 2H), 1.65 (br s, 1H), 1.29 (h, *J* = 7.7 Hz, 2H), 0.93 (t, *J* = 7.4 Hz, 3H).

^{13}C NMR (126 MHz, $CDCl_3$): δ 145.4, 145.1, 138.7, 128.5, 128.2, 128.0, 127.3, 126.2, 65.3, 50.9, 38.0, 21.3, 14.2.

HRMS (EI): *m/z* calculated for $C_{17}H_{20}O^+$ ($[M]^+$): 240.1514, found: 240.1507.

4-(1-Phenylbutyl)phenol **3l**:

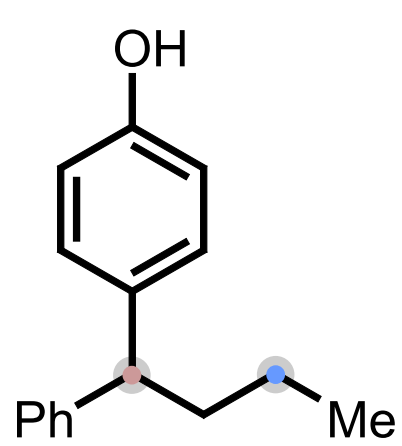

3l

The titled compound **3l** was prepared according to *Method A* described in *General Procedure 1* from 2,4,6-triphenyl-1-(4-phenylbutan-2-yl)pyridin-1-ium tetrafluoroborate **1a** (52.7 mg, 0.100 mmol, 1.00 equiv) and 4-bromophenol (34.6 mg, 0.200 mmol, 2.00 equiv). (*Note*: The regioselectivity (10:1 rr) of the reaction was determined by ^{1}H spectroscopic analysis of the crude reaction mixture of a duplicate run, which passed through a short silica plug, eluted with 7:3 hexane/ethyl acetate, and concentrated *in vacuo*.) The crude reaction mixture was purified by flash column chromatography using 100:0 → 80:20 hexane/ethyl acetate as an eluent followed by preparative HPLC (XBridge BEH C18 OBD column, 75% to 95% MeCN in H_2O with 0.1% NH_4OH) to yield compound **3l** as a colorless oil in 30% yield (6.8 mg, 0.03 mmol).

^{1}H NMR (500 MHz, $CDCl_3$): δ 7.27 (t, *J* = 7.5 Hz, 2H, overlaps with $CHCl_3$), 7.23 – 7.21 (m, 2H), 7.16 (tt, *J* = 7.2, 1.4 Hz, 1H), 7.10 (dt, *J* = 8.5, 3.0 Hz, 2H), 6.74 (dt, *J* = 8.5, 3.0 Hz, 2H), 4.70 (s, 1H), 3.85 (t, *J* = 7.8 Hz, 1H), 1.97 (qt, *J* = 7.8, 2.7 Hz, 2H), 1.33 – 1.22 (m, 2H, overlaps with grease), 0.92 (t, *J* = 7.3, 3H).

^{13}C NMR (126 MHz, $CDCl_3$): δ 153.8, 145.8, 137.8, 129.1, 128.5, 127.9, 126.1, 115.3, 50.3, 38.2, 21.3, 14.2.

HRMS (EI): *m/z* calculated for $C_{16}H_{18}O^+$ ($[M]^+$): 227.1436, found: 227.1438.

4-(1-Phenylbutyl)aniline **3m**:

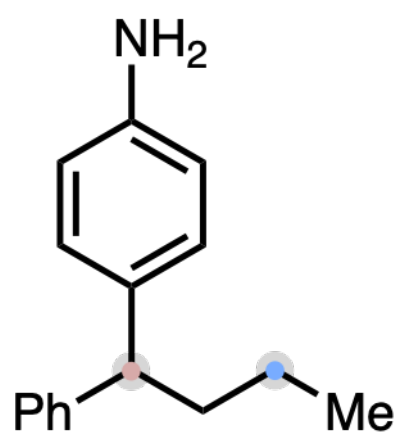

3m

The titled compound **3m** was prepared according to *Method A* described in *General Procedure 1* from 2,4,6-triphenyl-1-(4-phenylbutan-2-yl)pyridin-1-ium tetrafluoroborate **1a** (52.7 mg, 0.100 mmol, 1.00 equiv) and 4-bromoaniline (34.4 mg, 0.200 mmol, 2.00 equiv). (*Note*: The regioselectivity (20:1 rr) of the reaction was determined by ^{1}H spectroscopic analysis of the crude reaction mixture of a duplicate run, which passed through a short silica plug, eluted with 7:3 hexane/ethyl acetate, and concentrated *in vacuo*.) The crude reaction mixture was purified by flash column chromatography using 100:0 → 75:25 hexane/ethyl acetate as an eluent to yield compound **3m** as a brown oil in 68% yield (15.3 mg, 0.068 mmol). NMR spectra are in accordance with those reported in the literature.[14]

^{1}H NMR (500 MHz, $CDCl_3$): δ 7.29 – 7.24 (m, 2H, overlaps with $CHCl_3$), 7.23 – 7.20 (m, 2H), 7.15 (tt, 7.1, 1.5 Hz, 1H) 7.06 (dt, J = 8.3, 2.8 Hz, 2H), 6.61 (dt, J = 8.3, 2.8 Hz, 2H), 3.81 (t, J = 7.8 Hz, 1H), 3.55 (br s, 2H) 1.98 (q, J = 7.8 Hz, 2H), 1.34 – 1.21 (m, 2H), 0.92 (t, J = 7.3 Hz, 3H).

^{13}C NMR (126 MHz, $CDCl_3$): δ 146.2, 144.5, 135.7, 128.8, 128.4, 127.9, 125.9, 115.4, 50.3, 38.2, 21.3, 14.2.

HRMS (ESI): *m/z* calculated for $C_{16}H_{20}N^+$ ($[M + H]^+$): 226.1591, found: 226.1599.

1-Methoxy-3-(1-phenylbutyl)benzene **3n**:

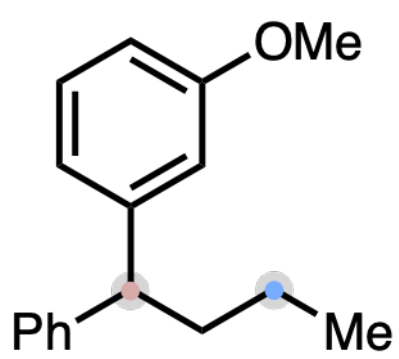

3n

The titled compound **3n** was prepared according to *Method A* described in *General Procedure 1* from 2,4,6-triphenyl-1-(4-phenylbutan-2-yl)pyridin-1-ium tetrafluoroborate **1a** (52.7 mg, 0.100 mmol, 1.00 equiv) and 1-bromo-3-methoxybenzene (25.3 mL, 0.200 mmol, 2.00 equiv). (*Note*: The regioselectivity (>20:1 rr) of the reaction was determined by ^{1}H spectroscopic analysis of the crude reaction mixture of a duplicate run, which passed through a short silica plug, eluted with 7:3 hexane/ethyl acetate, and concentrated *in vacuo*.) The crude reaction mixture was purified by flash column chromatography using 100:0 → 97:3 hexane/diethyl ether as an eluent followed by preparative thin layer chromatography (98:2 pentane/diethyl ether) to yield compound **3n** as a colorless oil in 91% yield (21.8 mg, 0.091 mmol). NMR spectra are in accordance with those reported in the literature.[14]

^{1}H NMR (300 MHz, $CDCl_3$): δ 7.33 – 7.22 (m, 4H, overlaps with $CHCl_3$), 7.22 – 7.13 (m, 2H), 6.85 (d, *J* = 7.7 Hz, 1H), 6.82 (t, *J* = 2.1 Hz, 1H), 6.73 (dd, *J* = 8.2, 2.1 Hz, 1H), 3.91 (t, *J* = 7.8 Hz, 1H), 3.80 (s, 3H), 2.02 (qt, *J* = 7.7, 2.4 Hz, 2H), 1.38 – 1.23 (m, 2H, overlaps with grease), 0.93 (t, *J* = 7.3 Hz, 3H).

^{13}C NMR (126 MHz, $CDCl_3$): δ 159.7, 147.2, 145.3, 129.4, 128.5, 128.0, 126.2, 120.5, 114.2, 111.0, 55.3, 51.2, 38.0, 21.3, 14.2.

HRMS (EI): *m*/*z* calculated for $C_{17}H_{20}O^+$ ($[M]^+$): 240.1514, found: 240.1510.

1-Methoxy-2-(1-phenylbutyl)benzene **3o**:

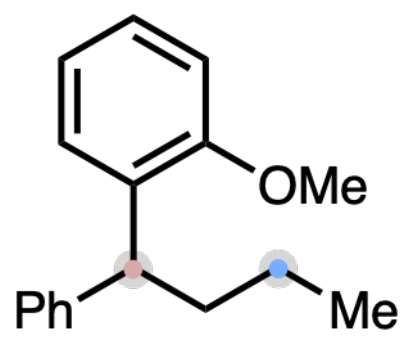

3o

The titled compound **3o** was prepared according to *Method A* described in *General Procedure 1* from 2,4,6-triphenyl-1-(4-phenylbutan-2-yl)pyridin-1-ium tetrafluoroborate **1a** (52.7 mg, 0.100 mmol, 1.00 equiv) and 1-bromo-2-methoxybenzene (24.9 mL, 0.200 mmol, 2.00 equiv). (*Note*: The regioselectivity (3:1 rr) of the reaction was determined by ^{1}H spectroscopic analysis of the crude reaction mixture of a duplicate run, which passed through a short silica plug, eluted with 7:3 hexane/ethyl acetate, and concentrated *in vacuo*.) The crude reaction mixture was purified by flash column chromatography using 100:0 → 97:3 hexane/diethyl ether as an eluent followed by preparative thin layer chromatography (98:2 pentane/diethyl ether) to yield compound **3o** as a colorless oil in 54% yield (21.8 mg, 0.091 mmol) as a mixture of regioisomers (3:1 regioisomeric ratio of **3o**/**4o**). NMR spectra are in accordance with those reported in the literature.[16]

^{1}H NMR (500 MHz, $CDCl_3$): δ 7.28 – 7.22 (m, 5H, overlaps with $CHCl_3$), 7.19 – 7.11 (m, 2H), 6.92 (td, *J* = 7.5, 1.2 Hz, 1H), 6.83 (d, *J* = 8.2 Hz, 1H), 4.41 (t, *J* = 7.8 Hz, 1H), 3.77 (s, 3H), 1.98 (qt, *J* = 7.6, 2.0 Hz, 2H), 1.33 – 1.25 (m, 2H), 0.92 (t, *J* = 7.4 Hz, 3H).

^{13}C NMR (126 MHz, $CDCl_3$): δ 157.2, 145.5, 134.0, 128.3, 128.2, 127.8, 127.0, 125.8, 120.6, 110.8, 55.6, 42.9, 37.4, 21.3, 14.3.

HRMS (EI): *m/z* calculated for $C_{17}H_{20}O^{+}$ ($[M]^{+}$): 240.1514, found: 240.1511.

tert-Butyl 5-(1-phenylbutyl)-1*H*-indole-1-carboxylate **3p**:

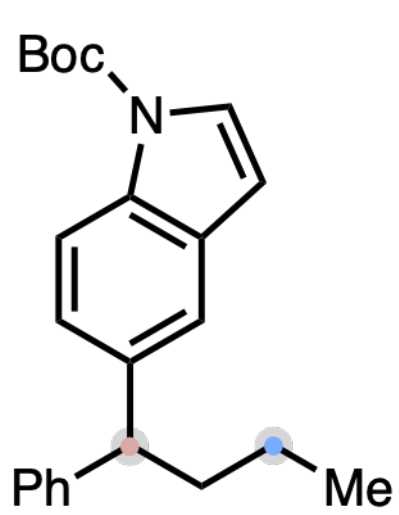

3p

The titled compound **3p** was prepared according to *Method A* described in *General Procedure 1* from 2,4,6-triphenyl-1-(4-phenylbutan-2-yl)pyridin-1-ium tetrafluoroborate **1a** (52.7 mg, 0.100 mmol, 1.00 equiv) and *tert*-butyl 5-bromo-1*H*-indole-1-carboxylate (59.2 mg, 0.200 mmol, 2.00 equiv). (*Note*: The regioselectivity (12:1 rr) of the reaction was determined by ^{1}H spectroscopic analysis of the crude reaction mixture of

a duplicate run, which passed through a short silica plug, eluted with 9:1 hexane/ethyl acetate, and concentrated *in vacuo*.) The crude reaction mixture was purified by flash column chromatography using 100:0 → 97:3 hexane/diethyl ether as an eluent followed by preparative thin layer chromatography to yield compound **3p** as a white solid in 68% yield (23.8 mg, 0.068 mmol). NMR spectra are in accordance with those reported in the literature.[17]

^{1}H NMR (500 MHz, $CDCl_3$): δ 8.03 (d, *J* = 8.0 Hz, 1H), 7.57 (d, *J* = 3.7 Hz, 1H), 7.44 (s, 1H), 7.27 (d, *J* = 4.3 Hz, 4H), 7.21 (dd, *J* = 8.0, 1.8 Hz, 1H), 7.18 – 7.13 (m, 1H), 6.52 (d, *J* = 3.7 Hz, 1H), 4.02 (t, *J* = 7.8 Hz, 1H), 2.08 (qd, *J* = 7.4, 1.7 Hz, 2H), 1.66 (s, 9H), 1.32 (h, *J* = 7.4 Hz, 2H), 0.95 (t, *J* = 7.4 Hz, 3H).

^{13}C NMR (126 MHz, $CDCl_3$): δ 150.0, 146.0, 139.9, 133.8, 130.9, 128.5, 128.0, 126.2, 126.0, 124.7, 120.0, 115.1, 107.5, 83.6, 51.0, 38.3, 28.3, 21.4, 14.2.

HRMS (ESI): *m/z* calculated for $C_{23}H_{27}NNaO_2^+$ ([M + Na]$^+$): 372.1934, found: 372.1937.

5-(1-phenylbutyl)-1H-indole **3q**:

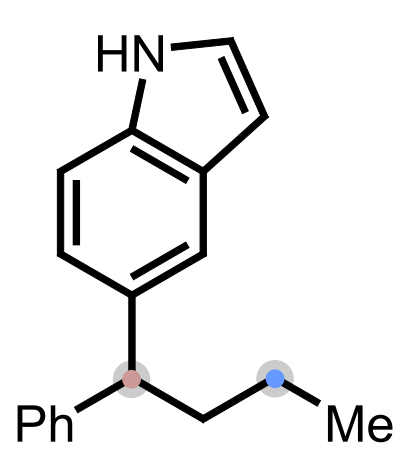

3q

The titled compound **3q** was prepared according to *Method A* described in *General Procedure 1* from 2,4,6-triphenyl-1-(4-phenylbutan-2-yl)pyridin-1-ium tetrafluoroborate **1a** (52.7 mg, 0.100 mmol, 1.00 equiv) and 5-bromo-1*H*-indole (38.8 mg, 0.200 mmol, 2.00 equiv). (*Note*: The regioselectivity (7:1 rr) of the reaction was determined by ^{1}H spectroscopic analysis of the crude reaction mixture of a duplicate run, which passed through a short silica plug, eluted with 9:1 hexane/ethyl acetate, and concentrated *in vacuo*.) The crude reaction mixture was purified by flash column chromatography using 100:0 → 92:8 hexane/ethyl acetate as an eluent followed by preparative HPLC (XBridge BEH C18 OBD column, 60% to 95% MeCN in H_2O with 0.1% NH_4OH) to yield compound **3q** as a white solid in 62% yield (15.4 mg, 0.062 mmol). NMR spectra are in accordance with those reported in the literature.[14]

^{1}H NMR (500 MHz, $CDCl_3$): δ 8.05 (br s, 1H), 7.52 (d, *J* = 1.1 Hz, 1H), 7.29 – 7.25 (m, 6H, overlaps with $CHCl_3$), 7.17 (t, *J* = 2.8 Hz, 1H), 7.13 (tt, *J* = 7.0, 1.6 Hz, 1H), 7.06 (dd, *J* = 8.4, 1.7 Hz, 1H), 6.49 (tt, *J* = 2.2, 0.7 Hz, 1H), 4.01 (t, *J* = 7.8 Hz, 1H), 2.08 (qd, *J* = 7.6, 3.9 Hz, 2H), 1.38 – 1.23 (m, 2H, overlaps with grease), 0.93 (t, *J* = 7.4 Hz, 3H).

^{13}C NMR (126 MHz, $CDCl_3$): δ 146.7, 137.0, 134.7, 128.4, 128.10, 128.06, 125.8, 124.4, 122.9, 119.5, 111.0, 102.7, 51.3, 38.5, 21.4, 14.3.

HRMS (ESI): *m/z* calculated for $C_{18}H_{20}N^+$ ($[M + H]^+$): 250.1591, found: 250.1587.

N,*N*-Dimethyl-5-(1-phenylbutyl)pyridin-2-amine **3r**:

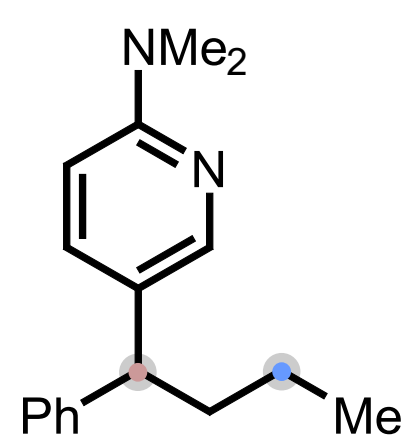

3r

The titled compound **3r** was prepared according to *Method A* described in *General Procedure 1* from 2,4,6-triphenyl-1-(4-phenylbutan-2-yl)pyridin-1-ium tetrafluoroborate **1a** (52.7 mg, 0.100 mmol, 1.00 equiv) and 5-bromo-N,N-dimethylpyridin-2-amine (40.2 mL, 0.200 mmol, 2.00 equiv). (*Note*: The regioselectivity (>20:1 rr) of the reaction was determined by ^{1}H spectroscopic analysis of the crude reaction mixture of a duplicate run, which passed through a short silica plug, eluted with 7:3 hexane/ethyl acetate, and concentrated *in vacuo*.) The crude reaction mixture was purified by flash column chromatography using 100:0 → 85:15 hexane/ethyl acetate as an eluent followed by preparative HPLC (XBridge BEH C18 OBD column, 75% to 95% MeCN in H_2O with 0.1% NH_4OH) to yield compound **3r** as a light yellow oil in 66% yield (16.8 mg, 0.066 mmol).

^{1}H NMR (500 MHz, $CDCl_3$): δ 8.08 (d, *J* = 2.6 Hz, 1H), 7.30 – 7.24 (m, 3H, overlaps with $CHCl_3$), 7.22 – 7.20 (m, 2H), 7.15 (tt, *J* = 7.2, 1.1 Hz, 1H), 6.45 (d, *J* = 9.6 Hz, 1H), 3.78 (t, *J* = 7.9 Hz, 1H), 3.04 (s, 6H), 1.97 (qd, *J* = 7.7, 2.7 Hz, 2H), 1.34 – 1.21 (m, 2H, overlaps with grease), 0.91 (t, *J* = 7.4 Hz, 3H).

^{13}C NMR (126 MHz, $CDCl_3$): δ 158.3, 147.1, 145.7, 137.0, 128.0, 128.5, 127.8, 126.1, 106.0, 47.8, 38.3, 37.9, 21.2, 14.2.

HRMS (ESI): *m/z* calculated for $C_{17}H_{23}N_2^+$ ($[M + H]^+$): 255.1856, found: 255.1891.

Methyl 5-(1-phenylbutyl)nicotinate **3s**:

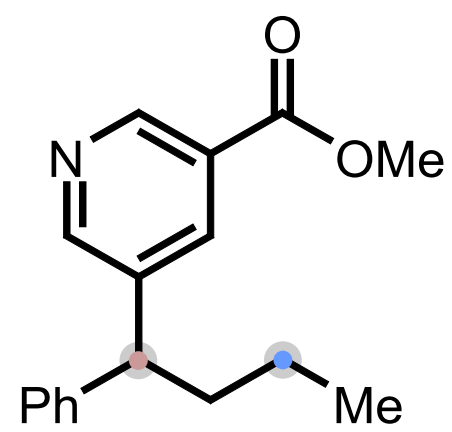

3s

The titled compound **3s** was prepared according to *Method B* described in *General Procedure 1* from 2,4,6-triphenyl-1-(4-phenylbutan-2-yl)pyridin-1-ium tetrafluoroborate **1a** (52.7 mg, 0.100 mmol, 1.00 equiv) and methyl 5-bromonicotinate (43.2 mg, 0.200 mmol, 2.00 equiv). (*Note*: *Note*: The regioselectivity (5:1 rr) of the reaction was determined by ^{1}H spectroscopic analysis of the crude reaction mixture of a duplicate run, which passed through a short silica plug, eluted with 7:3 hexane/ethyl acetate, and concentrated *in vacuo*.) The crude reaction mixture was purified by flash column chromatography using 100:0 → 80:20 hexane/ethyl acetate as an eluent followed by preparative HPLC (XBridge BEH C18 OBD column, 75% to 95% MeCN in H_2O with 0.1% NH_4OH) to yield compound **3s** as a colorless oil in 40% yield (12.0 mg, 0.04 mmol) as a mixture of regioisomers (6:1 regioisomeric ratio of **3s**/**4s**).

^{1}H NMR (500 MHz, $CDCl_3$): δ 9.03 (d, *J* = 2.0 Hz, 1H), 8.66 (d, *J* = 2.0, 1H), 8.15 (t, *J* = 2.0 Hz, 1H), 7.99 (tt, *J* = 7.5, 1.6 Hz, 2H), 7.22 (tt, *J* = 7.5, 1.6 Hz, 3H), 3.99 (t, *J* = 7.8 Hz, 1H), 3.93 (s, 3H), 2.14 – 2.00 (m, 2H), 1.31–1.23 (m, 2H), 0.94 (t, *J* = 7.3 Hz, 3H).

^{13}C NMR (126 MHz, $CDCl_3$): δ 166.1, 153.4, 148.8, 143.5, 140.8, 136.1, 128.9, 127.9, 126.8, 126.0, 52.5, 48.6, 37.5, 21.1, 14.1.

HRMS (ESI): *m/z* calculated for $C_{17}H_{20}NO_2^+$ ($[M + H]^+$): 270.1489, found: 270.1507.

4-(5-(1-Phenylbutyl)pyrimidin-2-yl)morpholine **3t**:

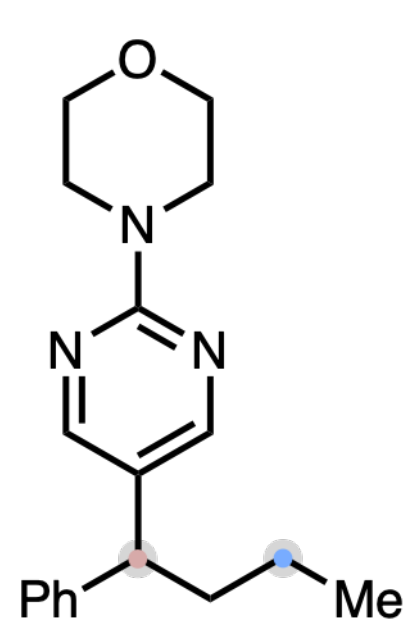

3t

The titled compound **3t** was prepared according to *Method A* described in *General Procedure 1* from 2,4,6-triphenyl-1-(4-phenylbutan-2-yl)pyridin-1-ium tetrafluoroborate **1a** (52.7 mg, 0.100 mmol, 1.00 equiv) and 4-(5-bromopyrimidin-2-yl)morpholine (48.8 mg, 0.200 mmol, 2.00 equiv). (*Note*: The regioselectivity (20:1 rr) of the reaction was determined by ^{1}H spectroscopic analysis of the crude reaction mixture of

a duplicate run, which passed through a short silica plug, eluted with 7:3 hexane/ethyl acetate, and concentrated *in vacuo*.) The crude reaction mixture was purified by flash column chromatography using 100:0 → 80:20 hexane:ethyl acetate as an eluent followed by preparative HPLC (XBridge BEH C18 OBD column, 75% to 95% MeCN in H_2O with 0.1% NH_4OH) to yield compound **3t** as a colorless oil in 46% yield (13.7 mg, 0.046 mmol).

^{1}H NMR (500 MHz, $CDCl_3$): δ 8.21 (br s, 2H), 7.28 (tt, J = 7.8, 1.9 Hz, 2H, overlaps with $CHCl_3$), 7.21 – 7.16 (m, 3H), 3.76 – 3.73 (m, 9H, overlapping singlet and triplet signals), 2.04 – 1.91 (m, 2H), 1.34 – 1.24 (m, 2H), 0.93 (t, J = 7.4 Hz, 3H).

^{13}C NMR (126 MHz, $CDCl_3$): δ 161.0, 157.3, 144.4, 128.8, 127.8, 126.7, 126.5, 67.0, 45.9, 44.5, 37.6, 21.1, 14.1.

HRMS (ESI): *m/z* calculated for $C_{18}H_{24}N_3O^+$ ([M + H]$^+$): 298.1914, found: 298.1929.

1-Fluoro-4-(1-(4-methoxyphenyl)propyl)benzene **3u**:

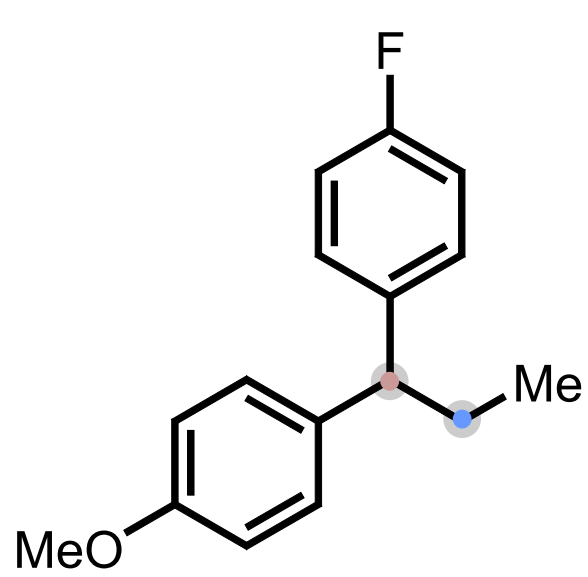

3u

The titled compound **3u** was prepared according to *Method A* described in *General Procedure 2* from 1-(1-(4-methoxyphenyl)propan-2-yl)-2,4,6-triphenylpyridin-1-ium tetrafluoroborate **1u** (54.3 mg, 0.100 mmol, 1.00 equiv) and 1-bromo-4-fluorobenzene (22 µL, 0.200mmol, 2.00 equiv). (*Note*: The regioselectivity (>20:1 rr) of the reaction was determined by ^{19}F spectroscopic analysis of the crude reaction mixture.) The crude reaction mixture was purified by flash column chromatography using hexane as an eluent to yield compound **3u** as a yellow oil in 86% yield (21 mg, 0.086 mmol). NMR spectra are in accordance with those reported in the literature.[18]

^{1}H NMR (500 MHz, $CDCl_3$): δ 7.18 – 7.14 (m, 2H), 7.12 (dt, J = 8.6, 1.8 Hz, 2H), 6.95 (tt, J = 8.7, 2.1 Hz, 2H), 6.82 (dt, J = 8.6, 1.8 Hz, 2H), 3.78 (s, 3H), 3.72 (t, J = 7.8 Hz, 1H), 2.01 (pd, J = 7.4, 2.0 Hz, 2H), 0.88 (t, J = 7.3 Hz, 3H).

^{13}C NMR (126 MHz, $CDCl_3$): δ 161.3 (d, *J* = 243.7 Hz), 158.0, 141.4 (d, *J* = 3.2 Hz), 137.3, 129.3 (d, *J* = 7.8 Hz), 128.8, 115.2 (d, *J* = 21.1 Hz), 113.9, 55.4, 51.7, 29.0, 12.9.

^{19}F{^{1}H} NMR (376 MHz, $CDCl_3$): δ -117.70.

HRMS (EI): *m/z* calculated for $C_{16}H_{17}FO^+$ ($[M]^+$): 244.1263, found: 244.1263.

1-Fluoro-4-(1-(4-methoxyphenyl)butyl)benzene **3v**:

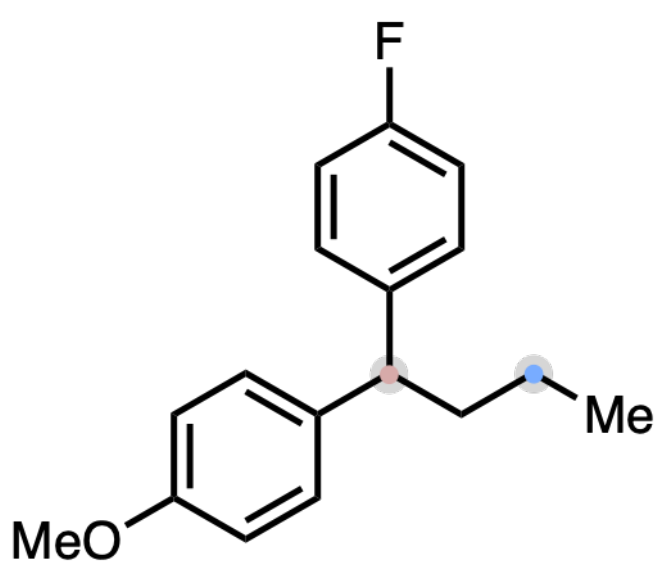

3v

The titled compound **3v** was prepared according to *Method A* described in *General Procedure 2* from 1-(4-(4-methoxyphenyl)butan-2-yl)-2,4,6-triphenylpyridin-1-ium tetrafluoroborate **1v** (55.7 mg, 0.100 mmol, 1.00 equiv) and 1-bromo-4-fluorobenzene **2a** (22 µL, 0.200mmol, 2.00 equiv). (*Note*: The regioselectivity (>20:1 rr) of the reaction was determined by ^{19}F spectroscopic analysis of the crude reaction mixture.) The crude reaction mixture was purified by flash column chromatography using 100:0 → 97:3 hexane/diethyl ether as an eluent followed by preparative thin layer chromatography (98:2 pentane/diethyl ether) to yield compound **3v** as a white solid in 85% yield (22.0 mg, 0.085 mmol). NMR spectra are in accordance with those reported in the literature.[17]

^{1}H NMR (500 MHz, $CDCl_3$): δ 7.20 – 7.15 (m, 2H), 7.13 (dt, *J* = 8.7, 2.2 Hz, 2H), 6.95 (tt, *J* = 8.7, 2.2 Hz, 2H), 6.83 (dt, *J* = 8.7, 2.2 Hz, 2H), 3.85 (t, *J* = 7.8 Hz, 1H), 3.78 (s, 3H), 1.96 (qd, *J* = 7.3, 2.7 Hz, 2H), 1.27 (h, *J* = 7.3 Hz, 2H), 0.92 (t, *J* = 7.3 Hz, 3H).

^{13}C NMR (126 MHz, $CDCl_3$) δ 161.3 (d, *J* = 243.8 Hz), 158.0, 141.6 (d, *J* = 3.3 Hz), 137.4, 129.2 (d, *J* = 7.8 Hz), 128.8, 115.2 (d, *J* = 21.0 Hz), 113.9, 55.4, 49.5, 38.4, 21.2, 14.2.

^{19}F{^{1}H} NMR (282 MHz, $CDCl_3$): δ -117.7.

HRMS (EI): *m/z* calculated for $C_{17}H_{19}FO^+$ ($[M]^+$): 258.1420, found: 258.1416.

1-Fluoro-4-(1-phenylheptyl)benzene **3w:**

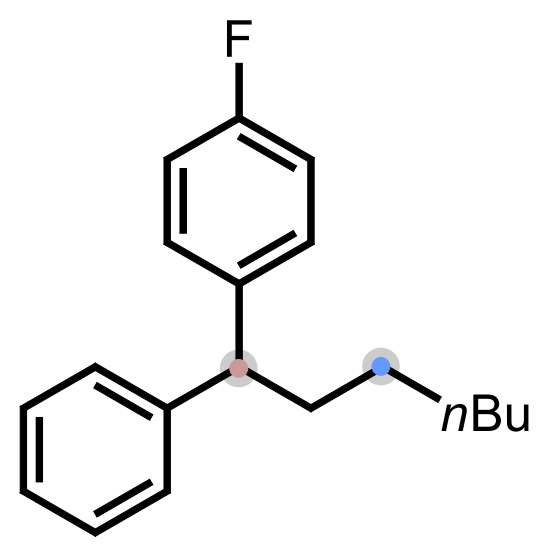

3w

The titled compound **3w** was prepared according to *Method A* described in *General Procedure 2* from 2,4,6-triphenyl-1-(1-phenylheptan-3-yl)pyridin-1-ium tetrafluoroborate **1w** (60.2 mg, 0.100 mmol, 1.00 equiv) and 1-bromo-4-fluorobenzene (22.0 µL, 0.200 mmol, 2.00 equiv). The regioselectivity (>20:1 rr) of the reaction was determined by ^{19}F spectroscopic analysis of the crude reaction mixture. The crude reaction mixture was purified by flash column chromatography using 100:0 → 95:5 pentane/diethyl ether as an eluent followed by preparative thin layer chromatography (99:1 pentane/diethyl ether) to yield compound **3w** as a colorless oil in 52% yield (14.0 mg, 0.052 mmol).

^{1}H NMR (300 MHz, $CDCl_3$): δ 7.32 – 7.24 (m, 2H, overlaps with $CDCl_3$), 7.23 – 7.14 (m, 5H), 6.96 (tt, *J* = 9.0, 3.5 Hz, 2H), 3.87 (t, *J* = 7.8 Hz, 1H), 2.01 (qd, *J* = 8.3, 1.9 Hz, 2H), 1.39 – 1.13 (m, 9H), 0.86 (t, *J* = 6.6 Hz, 3H).

^{13}C NMR (126 MHz, $CDCl_3$): δ 161.38 (d, *J* = 243.9 Hz), 145.3, 141.19 (d, *J* = 3.3 Hz), 129.3 (d, *J* = 7.8 Hz), 128.6, 127.9, 126.3, 115.23 (d, *J* = 21.0 Hz), 50.7, 36.0, 31.9, 29.4, 28.1, 22.8, 14.2.

^{19}F NMR (282 MHz, $CDCl_3$): δ -117.57.

HRMS (EI): *m/z* calculated for $C_{19}H_{23}F^+$ ($[M]^+$): 270.1784, found: 270.1787.

1-Fluoro-4-(1-phenylpentyl)benzene **3x**:

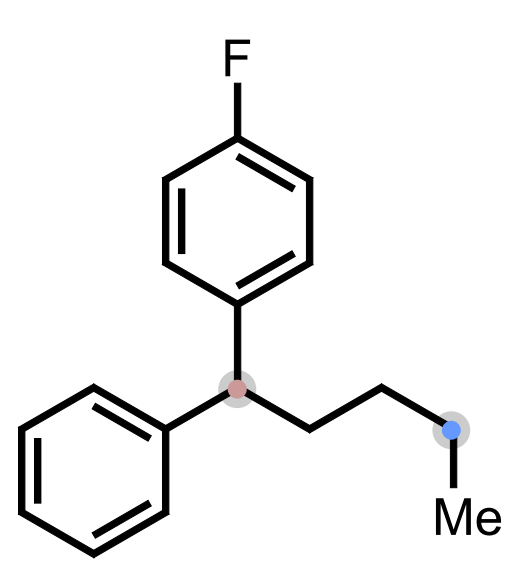

3x

The titled compound **3x** was prepared according to *Method A* described in *General Procedure 2* from 2,4,6-triphenyl-1-(5-phenylpentan-2-yl)pyridin-1-ium tetrafluoroborate **1x** (54.1 mg, 0.100 mmol, 1.00 equiv) and 1-bromo-4-fluorobenzene **2a** (22 µL, 0.200mmol, 2.00 equiv). (*Note*: The regioselectivity (>20:1 rr) of the reaction was determined by ^{19}F spectroscopic analysis of the crude

reaction mixture.) The crude reaction mixture was purified by flash column chromatography using 100:0 → 99:1 hexane/diethyl ether as an eluent to yield compound **3x** as a colorless oil in 50% yield (12.0 mg, 0.050 mmol).

^{1}H NMR (300 MHz, $CDCl_3$): δ 7.32 – 7.24 (m, 2H), 7.15 – 7.22 (m, 5H), 6.96 (t, *J* = 8.7 Hz, 2H), 3.87 (t, *J* = 7.8 Hz, 1H), 2.01 (qd, *J* = 7.8, 1.7 Hz, 2H), 1.46 – 1.14 (m, 4H), 0.87 (t, *J* = 7.1 Hz, 3H).

^{13}C NMR (126 MHz, $CDCl_3$): δ 161.4 (d, *J* = 243.9 Hz), 145.3, 141.2 (d, *J* = 3.2 Hz), 129.3 (d, *J* = 7.7 Hz), 128.6, 127.9, 126.3, 115.2 (d, *J* = 21.0 Hz), 50.7, 35.7, 30.3, 22.8, 14.1.

^{19}F NMR (282 MHz, $CDCl_3$): δ -117.56.

HRMS (EI): *m/z* calculated for $C_{17}H_{19}F^{+}$ ($[M]^{+}$): 242.1471, found: 242.1475.

4-(1-(4-Fluorophenyl)butyl)phenol, **3y**:

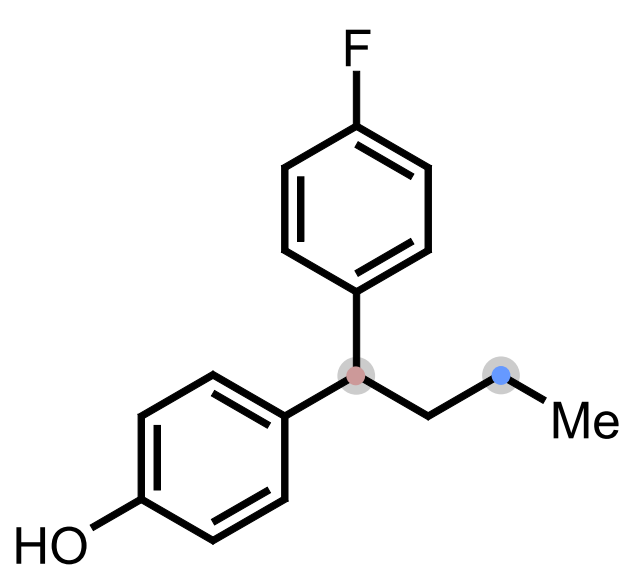

3y

The titled compound **3y** was prepared according to *Method A* described in *General Procedure 2* from 1-(4-(4-hydroxyphenyl)butan-2-yl)-2,4,6-triphenylpyridin-1-ium tetrafluoroborate **1y** (54.3 mg, 0.100 mmol, 1.00 equiv) and 1-bromo-4-fluorobenzene **2a** (22 µL, 0.200mmol, 2.00 equiv). (*Note*: The regioselectivity (>20:1 rr) of the reaction was determined by ^{19}F spectroscopic analysis of the crude reaction mixture.) The crude reaction mixture was purified by flash column chromatography using hexane as an eluent followed by preparative HPLC (XBridge BEH C18 OBD column, 75% to 95% MeCN in H_2O with 0.1% NH_4OH) to yield compound **3y** as a colorless oil in 72% yield (17.6 mg, 0.072 mmol).

^{1}H NMR (300 MHz, $CDCl_3$): δ 7.16 (dd, *J* = 8.7, 5.5 Hz, 2H), 7.07 (d, *J* = 8.5 Hz, 2H), 6.95 (t, *J* = 8.7 Hz, 2H), 6.75 (d, *J* = 8.5 Hz, 2H), 4.72 (br s, 1H), 3.83 (t, *J* = 7.9 Hz, 1H), 1.95 (qd, *J* = 7.0, 1.8 Hz, 2H), 1.34 – 1.18 (m, 2H, overlaps with grease), 0.92 (t, *J* = 7.4 Hz, 3H).

^{13}C NMR (126 MHz, $CDCl_3$): δ 161.3 (d, *J* = 243.6 Hz), 153.9, 141.5, 137.6, 129.2 (d, *J* = 7.4 Hz), 129.0, 115.4, 115.2 (d, *J* = 21.1 Hz), 49.5, 38.4, 21.2, 14.2.

^{19}F NMR (282 MHz, $CDCl_3$): δ -117.64.

HRMS (EI): m/z calculated for $C_{16}H_{17}FO^+$ ($[M]^+$): 244.1263, found: 244.1257.

1-Chloro-4-(1-(4-fluorophenyl)propyl)benzene **3z**:

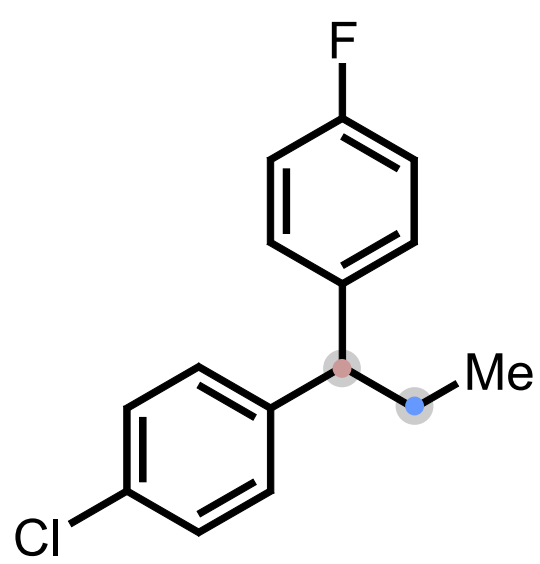

3z

The titled compound **3z** was prepared according to *Method A* described in *General Procedure 2* from 1-(1-(4-chlorophenyl)propan-2-yl)-2,4,6-triphenylpyridin-1-ium tetrafluoroborate **1z** (54.8 mg, 0.100 mmol, 1.00 equiv) and 1-bromo-4-fluorobenzene **2a** (22 µL, 0.200mmol, 2.00 equiv). (*Note*: The regioselectivity (>20:1 rr) of the reaction was determined by ^{19}F spectroscopic analysis of the crude reaction mixture.) The crude reaction mixture was purified by flash column chromatography using hexane as an eluent followed by preparative HPLC (XBridge BEH C18 OBD column, 75% to 95% MeCN in H_2O with 0.1% NH_4OH) to yield compound **3z** as a colorless oil in 63% yield (15.7 mg, 0.063 mmol).

^{1}H NMR (300 MHz, $CDCl_3$): δ 7.26 – 7.22 (m, 2H, overlaps with $CHCl_3$), 7.14 – 7.11 (m, 4H), 6.96 (tt, *J* = 8.7, 2.1 Hz, 2H), 3.74 (t, *J* = 7.3 Hz, 1H), 2.01 (p, *J* = 7.3 Hz, 2H), 0.88 (t, *J* = 7.3 Hz, 3H).

^{13}C NMR (126 MHz, $CDCl_3$): δ 161.5 (d, *J* = 244.4 Hz), 143.9, 140.4 (d, *J* = 3.3 Hz), 132.0, 129.3 (d, *J* = 7.7 Hz), 129.3, 128.7, 115.4 (d, *J* = 21.1 Hz), 51.9, 28.8, 12.8.

^{19}F NMR (282 MHz, $CDCl_3$): δ -117.1.

HRMS (ESI): *m/z* calculated for $C_{15}H_{14}ClF^+$ ($[M]^+$): 248.0768, found: 248.0772.

4,4'-(Propane-1,1-diyl)bis(fluorobenzene) **3aa**:

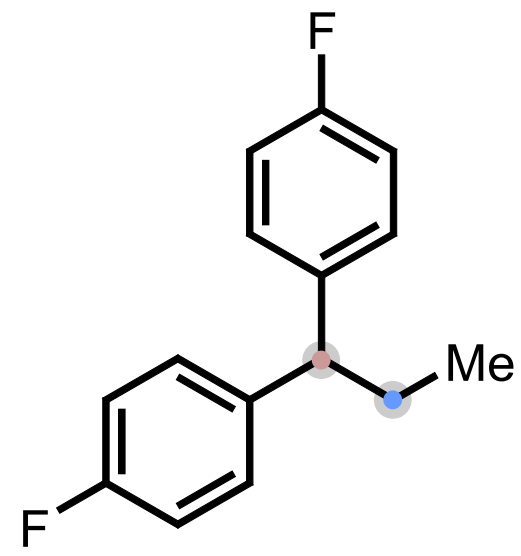

3aa

The titled compound **3aa** was prepared according to *Method A* described in *General Procedure 2* from 1-(1-(4-fluorophenyl)propan-2-yl)-2,4,6-triphenylpyridin-1-ium tetrafluoroborate **1aa** (53.1 mg, 0.100 mmol, 1.00 equiv) and 1-bromo-4-fluorobenzene **2a** (22 µL, 0.200mmol, 2.00 equiv). (*Note*: The regioselectivity (>20:1 rr) of the reaction was determined by ^{19}F spectroscopic analysis of the crude reaction mixture.) The crude reaction mixture was purified by flash column chromatography using hexane as an eluent followed by preparative HPLC (XBridge BEH C18 OBD column, 75% to 95% MeCN in H_2O with 0.1% NH_4OH) to yield compound **3aa** as a yellow oil in 80% yield (18.6 mg, 0.080 mmol). NMR spectra are in accordance with those reported in the literature.[19]

^{1}H NMR (300 MHz, $CDCl_3$): δ 7.19 – 7.12 (m, 4H), 6.96 (tt, *J* = 8.7, 2.1 Hz, 4H), 3.76 (t, *J* = 7.8 Hz, 1H), 2.01 (p, *J* = 7.4 Hz, 2H), 0.88 (t, *J* = 7.4 Hz, 3H).

^{13}C NMR (126 MHz, $CDCl_3$): δ 161.5 (d, *J* = 244.2 Hz), 140.8 (d, *J* = 2.3 Hz), 129.3 (d, *J* = 7.8 Hz), 115.3 (d, *J* = 21.2 Hz), 51.8, 29.0, 12.8.

^{19}F NMR (282 MHz, $CDCl_3$): δ -117.25.

HRMS (ESI): *m/z* calculated for $C_{15}H_{14}F_2$ ($[M]^+$): 232.1064, found: 232.1067.

1-Fluoro-4-(1-(4-(trifluoromethyl)phenyl)propyl)benzene **3ab**:

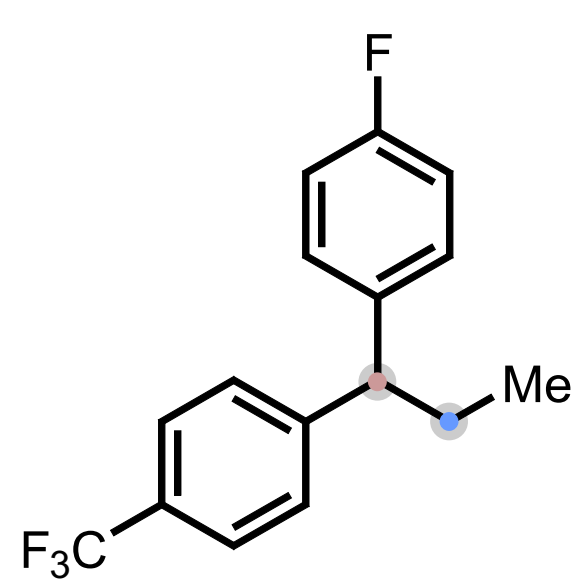

3ab

The titled compound **3ab** was prepared according to *Method A* described in *General Procedure 2* from 2,4,6-triphenyl-1-(1-(4-(trifluoromethyl)phenyl)propan-2-yl)pyridin-1-ium tetrafluoroborate **1ab** (58.1 mg, 0.100 mmol, 1.00 equiv) and 1-bromo-4-fluorobenzene **2a** (22 µL, 0.200mmol, 2.00 equiv). (*Note*: The regioselectivity (>20:1 rr) of the reaction was determined by ^{19}F spectroscopic analysis of the crude reaction mixture.) The crude reaction mixture was

purified by flash column chromatography using hexane as an eluent followed by preparative HPLC (XBridge BEH C18 OBD column, 75% to 95% MeCN in H_2O with 0.1% NH_4OH) to yield compound **3ab** as a white solid in 58% yield (16.4 mg, 0.060 mmol).

^{1}H NMR (300 MHz, $CDCl_3$): δ 7.53 (d, *J* = 8.1 Hz, 2H), 7.31 (d, *J* = 8.1 Hz, 2H), 7.20 – 7.14 (m, 2H), 6.98 (tt, *J* = 8.7, 2.1 Hz, 2H), 3.84 (t, *J* = 7.4 Hz, 1H), 2.06 (p, *J* = 7.4 Hz, 2H), 0.90 (t, *J* = 7.4 Hz, 3H).

^{13}C NMR (126 MHz, $CDCl_3$): δ 161.6 (d, *J* = 244.7 Hz), 149.2, 139.9 (d, *J* = 3.2 Hz), 129.4 (d, *J* = 7.8 Hz), 128.6 (q, *J* = 32.3 Hz), 128.3, 125.6 (q, *J* = 3.8 Hz), 124.4 (q, *J* = 271.8 Hz), 115.5 (d, *J* = 21.2 Hz), 52.4, 28.7, 12.7.

^{19}F NMR (282 MHz, $CDCl_3$): δ -62.4, -116.7.

HRMS (EI): *m/z* calculated for $C_{16}H_{14}F_4^+$ ($[M]^+$): 282.1032, found: 282.1037.

1-(1-(4-Fluorophenyl)propyl)-3-methoxybenzene **3ac**:

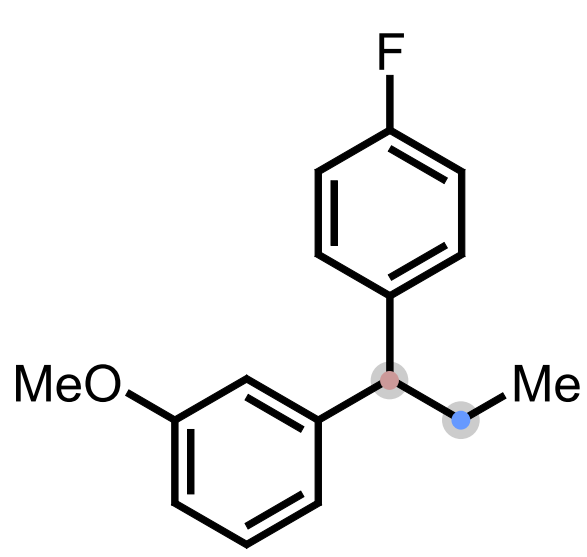

3ac

The titled compound **3ac** was prepared according to *Method A* described in *General Procedure 2* from 1-(1-(3-methoxyphenyl)propan-2-yl)-2,4,6-triphenylpyridin-1-ium tetrafluoroborate **1ac** (54.3 mg, 0.100 mmol, 1.00 equiv) and 1-bromo-4-fluorobenzene **2a** (22 µL, 0.200mmol, 2.00 equiv). (*Note*: The regioselectivity (>20:1 rr) of the reaction was determined by ^{19}F spectroscopic analysis of the crude reaction mixture.) The crude reaction mixture was purified by flash column chromatography using 100:0 → 97:3 hexane/diethyl ether as an eluent followed by preparative thin layer chromatography (98:3 pentane/diethyl ether) to yield compound **3ac** as a colorless oil in 74% yield (18.0 mg, 0.074 mmol).

^{1}H NMR (500 MHz, $CDCl_3$): δ 7.22 – 7.17 (m, 3H), 6.96 (tt, *J* = 8.7, 3.0 Hz, 2H), 6.81 (d, *J* = 7.7 Hz, 1H), 6.76 (t, *J* = 2.1 Hz, 1H), 6.72 (dd, *J* = 8.2, 2.1 Hz, 1H), 3.78 (s, 3H), 3.74 (t, *J* = 7.8 Hz, 1H), 2.04 (pd, *J* = 7.4, 1.2 Hz, 2H), 0.89 (t, *J* = 7.3 Hz, 3H).

^{13}**C NMR** (126 MHz, $CDCl_3$): δ 161.4 (d, *J* = 243.9 Hz), 159.8, 146.8, 140.8 (d, *J* = 3.3 Hz), 129.5, 129.3 (d, *J* = 7.8 Hz), 120.4, 115.2 (d, *J* = 21.1 Hz), 114.2, 111.1, 55.3, 52.6, 28.8, 12.9.

^{19}**F{^{1}H} NMR** (282 MHz, $CDCl_3$): δ -117.49.

HRMS (EI): *m/z* calculated for $C_{16}H_{17}FO^+$ ($[M]^+$): 244.1263, found: 244.1265.

1-(1-(4-Fluorophenyl)propyl)-3-(trifluoromethyl)benzene **3ad**:

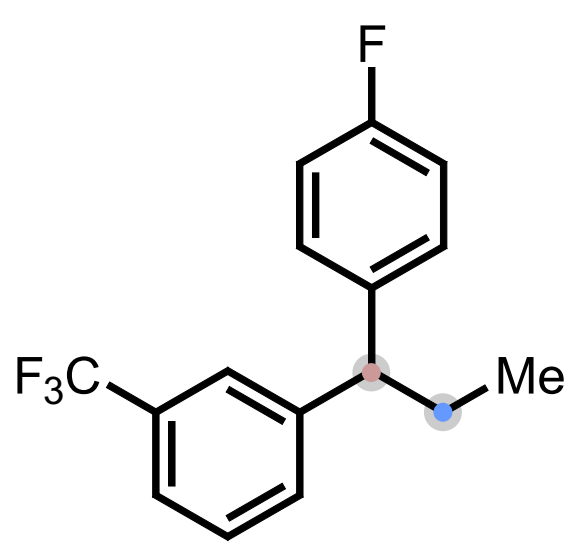

3ad

The titled compound **3ad** was prepared according to *Method A* described in *General Procedure 2* from 2,4,6-triphenyl-1-(1-(3-(trifluoromethyl)phenyl)propan-2-yl)pyridin-1-ium tetrafluoroborate **1ad** (58.1 mg, 0.100 mmol, 1.00 equiv) and 1-bromo-4-fluorobenzene **2a** (22 µL, 0.200mmol, 2.00 equiv). (*Note*: The regioselectivity (>20:1 rr) of the reaction was determined by ^{19}F spectroscopic analysis of the crude reaction mixture.) The crude reaction mixture was purified by flash column chromatography using hexane as an eluent followed by preparative HPLC (XBridge BEH C18 OBD column, 75% to 95% MeCN in H_2O with 0.1% NH_4OH) to yield compound **3ad** as a white solid in 60% yield (16.9 mg, 0.060 mmol).

^{1}**H NMR** (500 MHz, $CDCl_3$): δ 7.46 – 7.44 (m, 2H), 7.42 – 7.35 (m, 2H), 7.17 (m, 2H), 6.98 (tt, *J* = 8.7, 3.0 Hz, 2H), 3.84 (t, *J* = 7.3 Hz, 1H), 2.06 (p, *J* = 7.3 Hz, 2H), 0.90 (t, *J* = 7.3 Hz, 3H).

^{13}**C NMR** (126 MHz, $CDCl_3$): δ 161.5 (d, *J* = 244.7 Hz), 146.0, 139.8 (d, *J* = 3.2 Hz), 131.2 (q, *J* = 1.4 Hz), 130.7 (q, *J* = 32.0 Hz), 129.3 (d, *J* = 7.9 Hz), 128.9, 124.5 (q, *J* = 3.8 Hz), 123.1 (q, *J* = 3.8 Hz), 123.1 (q, *J* = 272.3 Hz), 115.4 (d, *J* = 21.2 Hz), 52.2, 28.6, 12.6.

^{19}**F{^{1}H} NMR** (282 MHz, $CDCl_3$): δ -62.5, -116.8.

HRMS (EI): *m/z* calculated for $C_{16}H_{14}F_4^+$ ($[M]^+$): 282.1032, found: 282.1034.

1-(1-(4-Fluorophenyl)propyl)-2-methoxybenzene **3ae**:

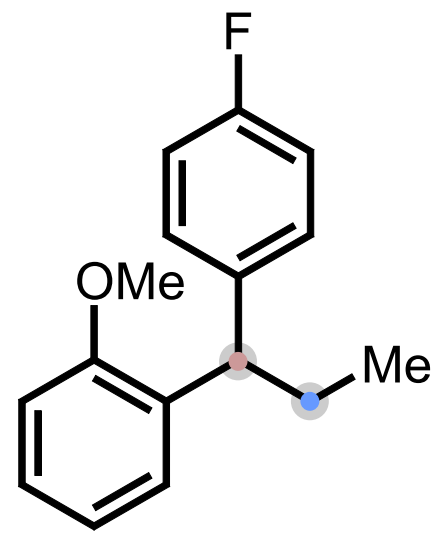

3ae

The titled compound **3ae** was prepared according to *Method A* described in *General Procedure 2* from 1-(1-(2-methoxyphenyl)propan-2-yl)-2,4,6-triphenylpyridin-1-ium tetrafluoroborate **1ae** (54.3 mg, 0.100 mmol, 1.00 equiv) and 1-bromo-4-fluorobenzene **2a** (22 µL, 0.200mmol, 2.00 equiv). (*Note*: The regioselectivity (12:1 rr) of the reaction was determined by ^{19}F spectroscopic analysis of the crude reaction mixture.) The crude reaction mixture was purified by flash column chromatography using 100:0 → 97:3 hexane/diethyl ether as an eluent followed by preparative thin layer chromatography (98:2 pentane/diethyl ether) to yield compound **3ae** as a colorless oil in 53% yield (12.9 mg, 0.053 mmol).

^{1}H NMR (500 MHz, $CDCl_3$): δ 7.22 – 7.14 (m, 4H), 6.92 (tt, *J* = 8.7, 1.8 Hz, 3H), 6.83 (d, *J* = 8.1 Hz, 1H), 4.24 (t, *J* = 7.8 Hz, 1H), 3.76 (s, 3H), 2.00 (pd, *J* = 7.2, 1.4 1H), 0.88 (t, *J* = 7.3 Hz, 1H).

^{13}C NMR (126 MHz, $CDCl_3$): δ 161.2 (d, *J* = 243.1 Hz), 157.2, 140.9 (d, *J* = 3.2 Hz), 133.6, 129.6 (d, *J* = 7.8 Hz), 127.5, 127.2, 120.7, 114.9 (d, *J* = 21.0 Hz), 110.9, 55.6, 44.6, 28.1, 12.8.

^{19}F{^{1}H} NMR (282 MHz, $CDCl_3$): δ -118.09.

HRMS (EI): *m/z* calculated for $C_{16}H_{17}FO^+$ ($[M]^+$): 244.1263, found: 244.1259.

1-(4-Fluorophenyl)-2,3-dihydro-1H-indene **3af**:

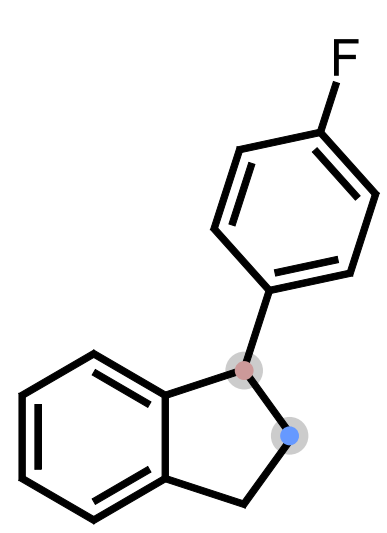

3af

The titled compound **3af** was prepared according to *Method A* described in *General Procedure 2* from 1-(2,3-dihydro-1H-inden-2-yl)-2,4,6-triphenylpyridin-1-ium tetrafluoroborate **1af** (51.1 mg, 0.100 mmol, 1.00 equiv) and 1-bromo-4-fluorobenzene **2a** (22 µL, 0.200mmol, 2.00 equiv). (*Note*: The regioselectivity (>20:1 rr) of the reaction was determined by ^{19}F spectroscopic analysis of the crude reaction mixture.)

The crude reaction mixture was purified by flash column chromatography using hexane as an eluent followed by preparative HPLC (XBridge BEH C18 OBD column, 75% to 95% MeCN in H_2O with 0.1% NH_4OH) to yield compound **3af** as a clear oil in 80% yield (17.0 mg, 0.080 mmol). NMR spectra in accordance with those reported in the literature.[20]

^{1}H NMR (300 MHz, $CDCl_3$): δ 7.30 – 7.26 (m, 1H, overlaps with $CHCl_3$), 7.22 – 7.11 (m, 4H), 6.99 (tt, *J* = 8.7, 2.2 Hz, 3H), 6.93 (d, *J* = 7.4 Hz, 1H), 4.32 (t, *J* = 8.3 Hz, 1H), 3.12 – 2.86 (m, 2H), 2.57 (dtd, *J* = 12.6, 7.8, 3.8 Hz, 1H), 2.01 (dq, *J* = 12.6, 8.7 Hz, 1H).

^{13}C NMR (126 MHz, $CDCl_3$): δ 161.7 (d, *J* = 244.0 Hz), 146.8, 144.4, 141.2 (d, *J* = 3.1 Hz), 129.6 (d, *J* = 7.8 Hz), 126.8, 126.6, 124.9, 124.6, 115.3 (d, *J* = 21.1 Hz), 51.0, 36.9, 31.9.

^{19}F{^{1}H} NMR (282 MHz, $CDCl_3$): δ -117.19.

HRMS (EI): *m/z* calculated for $C_{15}H_{13}F$ ($[M]^+$): 212.1001, found: 212.1001.

1-(4-Fluorophenyl)-1,2,3,4-tetrahydronaphthalzene **3ag**:

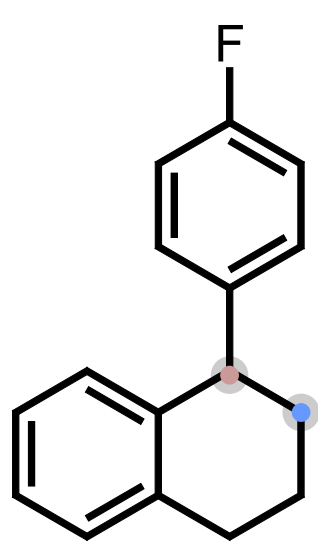

3ag

The titled compound **3ag** was prepared according to *Method A* described in *General Procedure 2* from 2,4,6-triphenyl-1-(4-phenylbutan-2-yl)pyridin-1-ium tetrafluoroborate **1a** (52.7 mg, 0.100 mmol, 1.00 equiv) and 1-bromo-4-fluorobenzene **2a** (22 µL, 0.200mmol, 2.00 equiv). (*Note*: The regioselectivity (>20:1 rr) of the reaction was determined by ^{19}F spectroscopic analysis of the crude reaction mixture.) The crude reaction mixture was purified by flash column chromatography using 100:0 → 98:2 hexane/diethyl ether to yield compound **3ag** as a white solid in 49% yield (11.0 mg, 0.049 mmol). NMR spectra in accordance with those reported in the literature.[11]

^{1}H NMR (500 MHz, $CDCl_3$): δ 7.16 – 7.10 (m, 2H), 7.07 – 7.02 (m, 3H), 6.97 (tt, *J* = 8.7, 2.9 Hz, 2H), 6.81 (d, *J* = 7.7 Hz, 1H), 4.11 (t, *J* = 6.6 Hz, 1H), 2.98 – 2.77 (m, 2H), 2.19 – 2.08 (m, 1H), 1.92 – 1.71 (m, 3H).

^{13}**C NMR** (126 MHz, $CDCl_3$): δ 161.4 (d, *J* = 243.7 Hz), 143.3 (d, *J* = 3.2 Hz), 139.3, 137.7, 130.3, 130.2 (d, *J* = 4.6 Hz), 129.2, 126.2, 125.8, 115.10 (d, *J* = 21.1 Hz), 45.0, 33.5 (d, *J* = 1.1 Hz), 29.9, 21.0.

^{19}**F NMR** (282 MHz, $CDCl_3$): δ -117.62.

HRMS (EI): *m/z* calculated for $C_{16}H_{15}F^+$ ($[M]^+$): 226.1158, found: 226.1155.

1-(4-Fluorophenyl)-5-methoxy-1,2,3,4-tetrahydronaphthalene **3ah**:

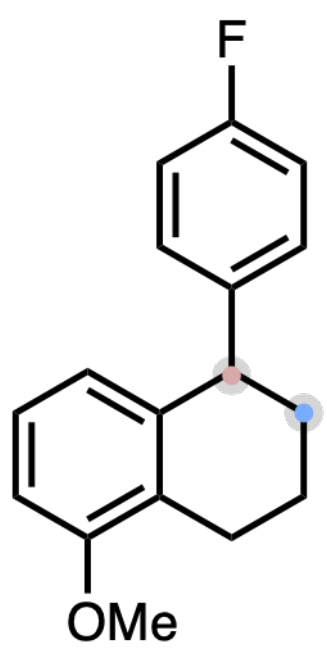

3ah

The titled compound **3ah** was prepared according to *Method A* described in *General Procedure 2* from 1-(5-methoxy-1,2,3,4-tetrahydronaphthalen-2-yl)-2,4,6-triphenylpyridin-1-ium tetrafluoroborate **1ah** (55.5 mg, 0.100 mmol, 1.00 equiv) and 1-bromo-4-fluorobenzene **2a** (22 µL, 0.200mmol, 2.00 equiv). (*Note*: The regioselectivity (>20:1 rr) of the reaction was determined by ^{19}F spectroscopic analysis of the crude reaction mixture.) The crude reaction mixture was purified by flash column chromatography using 100:0 → 97:3 hexane/diethyl ether as an eluent followed by preparative thin layer chromatography (98:3 pentane/diethyl ether) to yield compound **3ah** as a colorless oil in 47% yield (12.0 mg, 0.047 mmol). NMR spectra in accordance with those reported in the literature.[7]

^{1}**H NMR** (500 MHz, $CDCl_3$): δ 7.07 – 6.99 (m, 3H), 6.95 (tt, *J* = 8.7, 2.2 Hz, 2H), 6.69 (d, *J* = 8.1 Hz, 1H), 6.45 (d, *J* = 7.8 Hz, 1H), 4.10 (t, *J* = 6.4 Hz, 1H), 3.85 (s, 3H), 2.75 (td, *J* = 6.4, 2.4 Hz, 2H), 2.12 – 2.06 (m, 1H), 1.87 – 1.78 (m, 2H), 1.76 – 1.68 (m, 1H).

^{13}**C NMR** (126 MHz, $CDCl_3$): δ 161.4 (d, *J* = 243.7 Hz), 157.3, 143.2 (d, *J* = 3.3 Hz), 140.5, 130.3 (d, *J* = 7.8 Hz), 126.8, 126.0, 122.4, 115.0 (d, *J* = 21.0 Hz), 107.2, 55.4, 44.9, 32.8 (d, *J* = 1.1 Hz), 23.4, 20.1.

^{19}**F{^{1}H} NMR** (282 MHz, $CDCl_3$): δ -117.72.

HRMS (EI): *m/z* calculated for $C_{17}H_{17}FO^+$ ($[M]^+$): 256.1263, found: 256.1259.

2-(1-(4-Fluorophenyl)propyl)furan **3ai:**

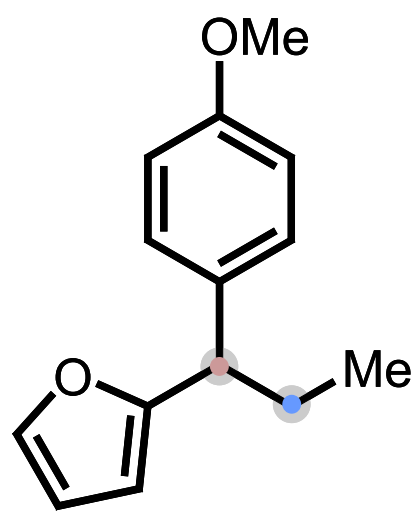

3ai

The titled compound **3ai** was prepared according to *Method A* described in *General Procedure 2* from 1-(1-(furan-2-yl)propan-2-yl)-2,4,6-triphenylpyridin-1-ium tetrafluoroborate **1ai** (25.2 mg, 0.050 mmol, 1.00 equiv) and 1-bromo-4-fluorobenzene (11.0 µL, 0.100 mmol, 2.00 equiv). The regioselectivity (>20:1 rr) of the reaction was determined by ^{19}F spectroscopic analysis of the crude reaction mixture. The crude reaction mixture was purified by flash column chromatography using 100:0 → 90:10 hexane/ethyl acetate as an eluent followed by reverse phase column chromatography using 40% to 100% MeCN in H_2O to yield compound **3ai** as a white solid in 73% yield (7.9 mg, 0.037 mmol).

^{1}H NMR (500 MHz, $CDCl_3$) δ 7.30 (d, *J* = 2.5 Hz, 1H), 7.14 (dt, *J* = 8.6, 3.3 Hz, 2H), 6.84 (t, *J* = 2.8 Hz, 2H), 6.28 (dd, *J* = 3.2, 1.9 Hz, 1H), 6.03 (d, *J* = 3.2 Hz, 1H), 3.83 – 3.73 (m, 4H), 2.11 (hept, *J* = 7.4 Hz, 1H), 1.86 (hept, *J* = 7.4 Hz, 1H), 0.88 (t, *J* = 7.3 Hz, 3H).

^{13}C NMR (126 MHz, CDCl3) δ 158.65, 158.32, 141.30, 134.97, 128.95, 113.94, 110.03, 105.11, 55.37, 46.41, 28.03, 12.47.

HRMS (EI): *m/z* calculated for $C_{14}H_{16}O_2^+$ ([M]$^+$): 216.1150, found: 228.1142.

3-(1-(4-Fluorophenyl)propyl)thiophene **3aj:**

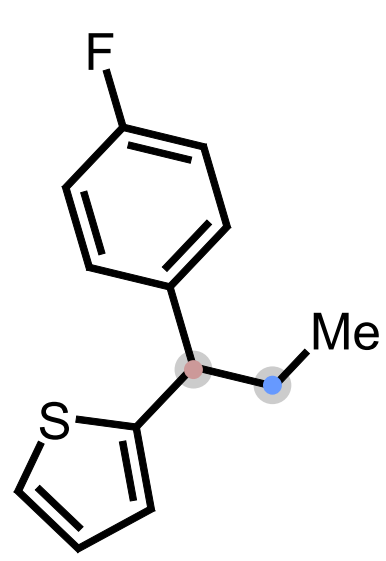

3aj

The titled compound **3aj** was prepared according to *Method A* described in *General Procedure 2* from 2,4,6-triphenyl-1-(1-(thiophen-3-yl)propan-2-yl)pyridin-1-ium tetrafluoroborate **1aj** (27.8 mg, 0.050 mmol, 1.00 equiv) and 1-bromo-4-fluorobenzene (11.0 µL, 0.100 mmol, 2.00 equiv). The regioselectivity (>20:1 rr) of the reaction was determined by ^{19}F spectroscopic analysis of the crude reaction mixture. The crude reaction mixture was purified by flash column chromatography using 100:0 → 95:5 pentane/diethyl ether as an eluent followed by preparative thin layer chromatography (98:2 pentane/diethyl ether) to yield compound **3aj** as a colorless oil in 56% yield (6.2 mg, 0.028 mmol).

^{1}**H NMR** (500 MHz, $CDCl_3$): δ 7.24 – 7.19 (m, 2H), 7.14 (dd, *J* = 5.2, 1.2 Hz, 1H), 6.98 (tt, *J* = 8.7, 3.2 Hz, 2H), 6.91 (dd, *J* = 5.2, 3.5 Hz, 1H), 6.80 (d, *J* = 3.6 Hz, 1H), 4.00 (t, *J* = 7.7 Hz, 1H), 2.14 (hept, *J* = 7.5 Hz, 1H), 2.06 – 1.96 (m, 1H), 0.90 (t, *J* = 7.3 Hz, 3H).

^{13}**C NMR** (126 MHz, $CDCl_3$): δ 161.66 (d, *J* = 244.4 Hz), 149.6, 140.4 (d, *J* = 3.1 Hz), 129.30 (d, *J* = 7.9 Hz), 126.7, 123.9, 123.7, 115.37 (d, *J* = 21.2 Hz), 40.1, 29.9, 12.7.

^{19}**F NMR** (282 MHz, $CDCl_3$): δ -116.80.

HRMS (EI): *m/z* calculated for $C_{13}H_{13}FS^+$ ($[M]^+$): 220.0722, found: 220.0716.

3-(1-(4-Fluorophenyl)propyl)benzofuran **3ak:**

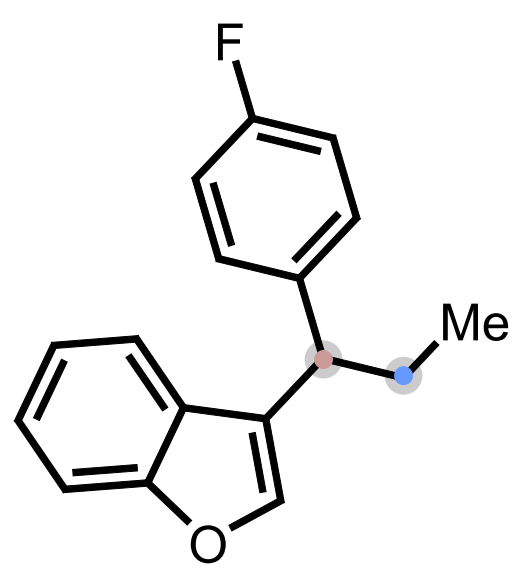

3ak

The titled compound **3ak** was prepared according to *Method A* described in *General Procedure 2* from 1-(1-(benzofuran-3-yl)propan-2-yl)-2,4,6-triphenylpyridin-1-ium tetrafluoroborate **1ak** (30.9 mg, 0.050 mmol, 1.00 equiv) and 1-bromo-4-fluorobenzene (11.0 µL, 0.100 mmol, 2.00 equiv). The regioselectivity (>20:1 rr) of the reaction was determined by ^{19}F spectroscopic analysis of the crude reaction mixture. The crude reaction mixture was purified by flash column chromatography using 100:0 → 95:5 pentane/diethyl ether as an eluent followed by preparative thin layer chromatography (98:2 pentane/diethyl ether) to yield compound **3ak** as a colorless oil in 63% yield (8.0 mg, 0.032 mmol).

^{1}**H NMR** (500 MHz, $CDCl_3$): δ 7.48 (br s, 1H), 7.44 (d, *J* = 8.2 Hz, 1H), 7.28 – 7.20 (m, 4H, overlaps with $CDCl_3$), 7.11 (t, *J* = 7.5 Hz, 1H), 6.97 (t, *J* = 8.7 Hz, 2H), 3.92 (t, *J* = 7.7 Hz, 1H), 2.21 (hept, *J* = 7.1 Hz, 1H), 1.97 (hept, *J* = 7.5 Hz, 1H), 0.95 (t, *J* = 7.4 Hz, 3H)

^{13}**C NMR** (126 MHz, $CDCl_3$): δ 161.6 (d, *J* = 244.1 Hz), 155.7, 141.2, 139.19 (d, *J* = 3.1 Hz), 129.48 (d, *J* = 7.9 Hz), 127.7, 124.3, 124.1, 122.4, 120.4, 115.35 (d, *J* = 21.2 Hz), 111.6, 43.2, 28.5, 12.6.

^{19}**F NMR** (282 MHz, $CDCl_3$): δ -116.86.

HRMS (EI): *m/z* calculated for $C_{17}H_{15}FO^+$ ($[M]^+$): 254.1107, found: 254.1108.

3-(1-(4-Fluorophenyl)propyl)-1-methyl-1H-indole **3al:**

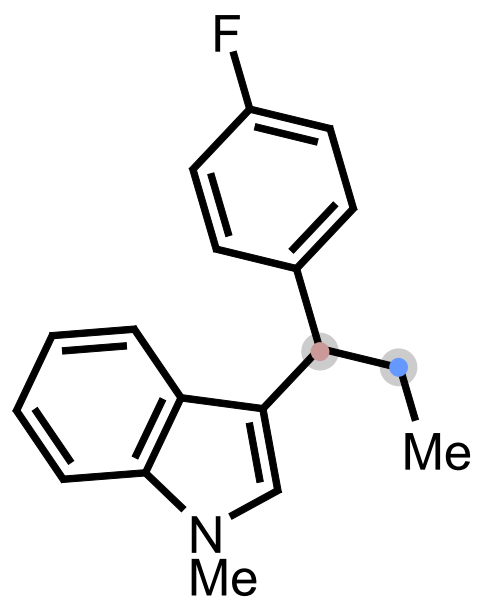

3al

The titled compound **3al** was prepared according to *Method A* described in *General Procedure 2* from 1-(1-(1-methyl-1*H*-indol-3-yl)propan-2-yl)-2,4,6-triphenylpyridin-1-ium tetrafluoroborate **1al** (28.3 mg, 0.050 mmol, 1.00 equiv) and 1-bromo-4-fluorobenzene (11.0 µL, 0.100 mmol, 2.00 equiv). The regioselectivity (>3:1 rr) of the reaction was determined by ^{19}F spectroscopic analysis of the crude reaction mixture. The crude reaction mixture was purified by flash column chromatography using 100:0 → 90:10 hexane/ethyl acetate as an eluent followed by reverse phase column chromatography using 40% to 100% MeCN in H_2O to yield compound **3al** as a white solid in 39% yield (5.3 mg, 0.020 mmol).

^{1}H NMR (400 MHz, $CDCl_3$) δ 7.39 (dt, *J* = 7.9, 1.0 Hz, 1H), 7.29 – 7.21 (m, 8H), 7.20 – 7.13 (m, 1H), 7.03 – 6.97 (m, 1H), 6.94 (tt, *J* = 8.7, 2.9 Hz, 2H), 6.85 (br s, 1H), 4.03 (t, *J* = 7.6 Hz, 1H), 3.76 (s, 3H), 2.26 – 2.14 (m, 1H), 2.04 – 1.90 (m, 1H), 0.94 (t, *J* = 7.3 Hz, 3H).

^{13}C NMR (126 MHz, CDCl3) δ 160.2 (d, *J* = 243.2 Hz), 140.11 (d, *J* = 3.1 Hz), 136.2, 128.22 (d, *J* = 7.8 Hz), 126.31, 124.70, 120.51, 118.47, 117.77, 117.61, 113.87 (d, *J* = 21.0 Hz), 108.09, 43.0, 28.7, 28.2, 11.7.

^{19}F NMR (282 MHz, $CDCl_3$): δ -117.93.

HRMS (ESI): *m/z* calculated for $C_{18}H_{19}FN^+$ ($[M + H]^+$): 268.1497, found: 268.1489.

4-(1-(4-Fluorophenyl)ethyl)-1-phenyl-1H-pyrazole **3am:**

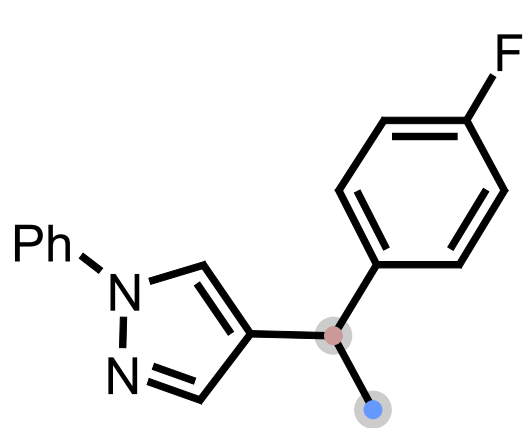

3am

The titled compound **3am** was prepared according to *Method C* described in *General Procedure 2* from 14-(2-(1-phenyl-1H-pyrazol-4-yl)ethyl)-7-(4-(trifluoromethyl)phenyl)-5,6,8,9-tetrahydrodibenzo[c,h]acridin-14-ium trifluoromethanesulfonate **Acridin3** (37.4 mg, 0.050 mmol, 1.00 equiv) and 1-bromo-4-fluorobenzene (11.0 µL, 0.100 mmol, 2.00 equiv). The

regioselectivity (>20:1 rr) of the reaction was determined by ^{19}F spectroscopic analysis of the crude reaction mixture. The crude reaction mixture was purified by flash column chromatography using 100:0 → 90:10 hexane/ethyl acetate as an eluent followed by preparative thin layer chromatography (90:10 pentane/diethyl ether) to yield compound **3am** as a colorless oil in 45% yield (6.0 mg, 0.023 mmol).

^1H NMR (500 MHz, $CDCl_3$): δ 7.68 – 7.59 (m, 3H), 7.52 (br s, 1H), 7.43 (tt, *J* = 8.0, 2.3 Hz, 2H), 7.31 – 7.16 (m, 3H, overlaps with $CDCl_3$), 7.00 (tt, *J* = 8.7, 3.2 Hz, 2H), 4.10 (q, *J* = 7.2 Hz, 1H), 1.62 (d, *J* = 7.1 Hz, 3H).

^{13}C NMR (126 MHz, $CDCl_3$): δ 161.57 (d, *J* = 244.1 Hz), 142.15 (d, *J* = 3.1 Hz), 140.3, 140.2, 129.5, 129.1, 128.83 (d, *J* = 7.8 Hz), 126.3, 124.7, 119.0, 115.41 (d, *J* = 21.2 Hz), 35.3, 23.0.

^{19}F NMR (282 MHz, $CDCl_3$): δ -117.02.

HRMS (ESI): *m/z* calculated for $C_{17}H_{16}FN_2^+$ ($[M + H]^+$): 266.1293, found: 266.1289.

2-(1-(4-Fluorophenyl)ethyl)thiophene **3an:**

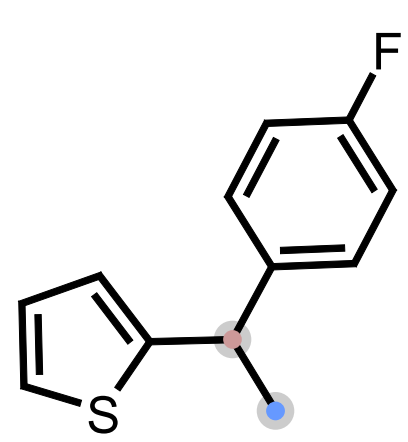

3an

The titled compound **3an** was prepared according to *Method C* described in *General Procedure 2* from 14-(2-(1-phenyl-1H-pyrazol-4-yl)ethyl)-7-(4-(trifluoromethyl)phenyl)-5,6,8,9-tetrahydrodibenzo[c,h]acridin-14-ium trifluoromethanesulfonate **Acridin4** (34.4 mg, 0.050 mmol, 1.00 equiv) and 1-bromo-4-fluorobenzene **2a** (11 µL, 0.100mmol, 2.00 equiv). The (NMR) yield and regioselectivity (>20:1 rr) of the reaction were obtained by analysis of the crude reaction mixture by ^{19}F NMR spectroscopy using 1-bromonaphthalene as an internal standard.

4-(5-(4-(1-Phenylbutyl)3-(trifluoromethyl)-*1H*-pyrazol-1-yl)benzenesulfonamide **3ao**:

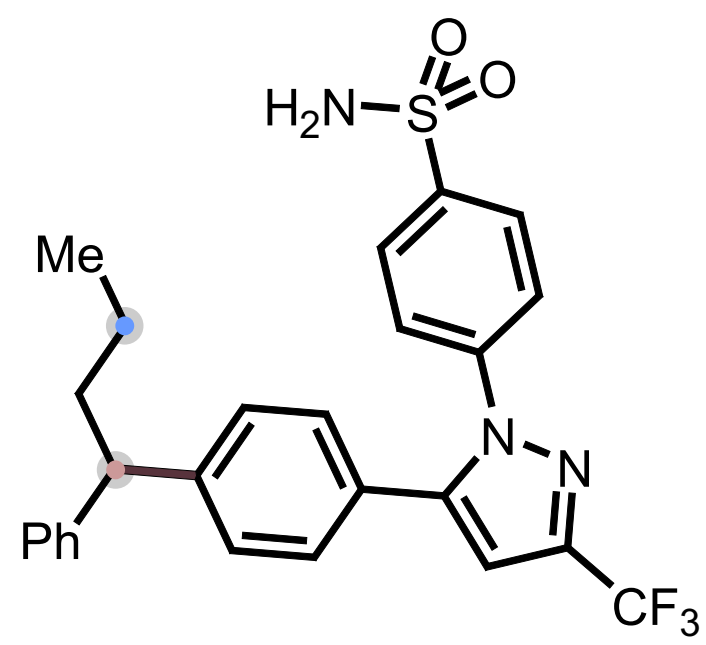

3ao

The titled compound **3ao** was prepared according to *Method B* described in *General Procedure 1* from 2,4,6-triphenyl-1-(4-phenylbutan-2-yl)pyridin-1-ium tetrafluoroborate **1a** (52.7 mg, 0.100 mmol, 1.00 equiv) and 4-(5-(4-bromophenyl)-3-(trifluoromethyl)-1*H*-pyrazol-1-yl)benzenesulfonamide **2ao** (89.2 mg, 0.200 mmol, 2.00 equiv). (*Note*: The regioselectivity (13:1 rr) of the reaction was determined by ^{1}H spectroscopic analysis of the crude reaction mixture of a duplicate run, which passed through a short silica plug, eluted with 7:3 hexane/ethyl acetate, and concentrated *in vacuo*.) The crude reaction mixture was purified by flash column chromatography using 100:0 → 75:25 hexane/ethyl acetate as an eluent followed by preparative HPLC (XBridge BEH C18 OBD column, 60% to 95% MeCN in H_2O with 0.1% NH_4OH) to yield compound **3ao** as a white solid in 62% yield (30.9 mg, 0.062 mmol).

^{1}H NMR (500 MHz, $CDCl_3$): δ 7.89 (dt, *J* = 8.8, 2.5 Hz, 2H), 7.47 (dt, *J* = 8.8, 2.5 Hz, 2H), 7.30 (tt, *J* = 7.8, 1.6 Hz, 2H), 7.26 – 7.19 (m, 5H), 7.12 (dt, *J* = 8.2, 1.9 Hz, 2H), 6.72 (s, 1H), 4.82 (br s, 2H), 3.92 (t, *J* = 7.8 Hz, 1H), 2.07 – 1.96 (m, 2H), 1.33 – 1.22 (m, 2H), 0.93 (t, *J* = 7.3 Hz, 3H).

^{13}C NMR (126 MHz, $CDCl_3$): δ 149.4, 145.3, 144.3 (q, *J* = 38.6 Hz),142.8, 142.2, 141.4, 129.0, 128.53, 128.45, 127.9, 127.7, 126.4, 126.0, 125.7, 121.2 (q, *J* = 269.2 Hz), 106.6 (q, *J* = 1.8 Hz), 39.9, 39.4, 34.0, 22.2.

^{19}F NMR (282 MHz, $CDCl_3$): δ -62.5.

HRMS (ESI): *m/z* calculated for $C_{26}H_{25}F_3N_3O_2S^+$ ([M + H]$^+$): 500.1615, found: 500.1609.

5-Benzoyl-*N*-(3-((*R*)-1-phenylbutyl)phenyl)-2,3-dihydro-1*H*-pyrrolizine-1-carboxamide **3ap**:

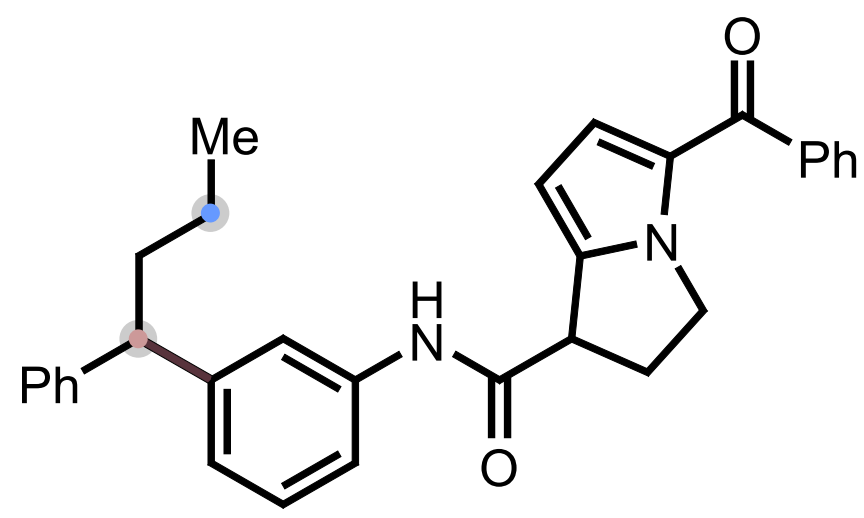

3ap

The titled compound **3ap** was prepared according to *Method B* described in *General Procedure 1* from 2,4,6-triphenyl-1-(4-phenylbutan-2-yl)pyridin-1-ium tetrafluoroborate **1a** (52.7 mg, 0.100 mmol, 1.00 equiv) and 5-benzoyl-*N*-(3-bromophenyl)-2,3-dihydro-1*H*-pyrrolizine-1-carboxamide **2ap** (81.8 mg, 0.200 mmol, 2.00 equiv). (*Note*: The regioselectivity (16:1 rr) of the reaction was determined by ^{1}H spectroscopic analysis of the crude reaction mixture of a duplicate run, which passed through a short silica plug, eluted with 6:4 hexane/ethyl acetate, and concentrated *in vacuo*.) The crude reaction mixture was purified by flash column chromatography using 100:0 → 70:30 hexane/ethyl acetate as an eluent followed by preparative HPLC (XBridge BEH C18 OBD column, 60% to 95% MeCN in H_2O with 0.1% NH_4OH) to yield compound **3ap** as a white solid in 54% yield (25 mg, 0.054 mmol).

^{1}H NMR (500 MHz, $CDCl_3$): δ 7.84 (d, *J* = 7.1 Hz, 2H), 7.55 (t, *J* = 7.4 Hz, 1H), 7.49 – 7.46 (m, 3H), 7.38 – 7.36 (m, 1H), 7.34 – 7.21 (m, 4H, overlaps with $CHCl_3$), 7.16 (t, *J* = 7.2 Hz, 1H), 7.02 (d, *J* = 7.6 Hz, 1H), 6.89 (d, *J* = 4.0 Hz, 1H), 6.17 (d, *J* = 4.0 Hz, 1H), 4.61 – 4.56 (m, 1H), 4.50 – 4.45 (m, 1H), 4.06 (dd, *J* = 8.4, 6.2 Hz, 1H), 3.88 (t, *J* = 7.8 Hz, 1H), 3.03 – 2.94 (m, 2H), 2.00 (q, *J* = 8.1 Hz, 2H), 1.30 – 1.23 (m, 2H, overlaps with grease), 0.92 (t, *J* = 7.4 Hz, 3H).

^{13}C NMR (126 MHz, $CDCl_3$): δ 185.3, 169.0, 146.74, 146.72, 145.05, 145.04, 142.4, 139.2, 137.5, 131.8, 129.2, 129.1, 128.6, 128.4, 128.01, 127.98, 126.3, 125.2, 124.48, 124.45, 119.58, 119.57, 117.9, 117.8, 103.0, 51.2, 47.8, 45.6, 37.92, 37.88, 32.9, 21.3, 14.2. Mixture of diastereomers.

HRMS (ESI): *m/z* calculated for $C_{31}H_{31}N_2O_2$ ([M + H]$^+$): 463.2386, found: 463.2388.

(4a*R*,4a^1*R*,5a*S*,8a*R*,8a^1*S*,15a*S*)-10-((*R*)-1-Phenylbutyl)-2,4a,4a^1,5,5a,7,8,8a^1,15,15a-decahydro-14*H*-4,6-methanoindolo[3,2,1-*ij*]oxepino[2,3,4-*de*]pyrrolo[2,3-*h*]quinolin-14-one **3aq**:

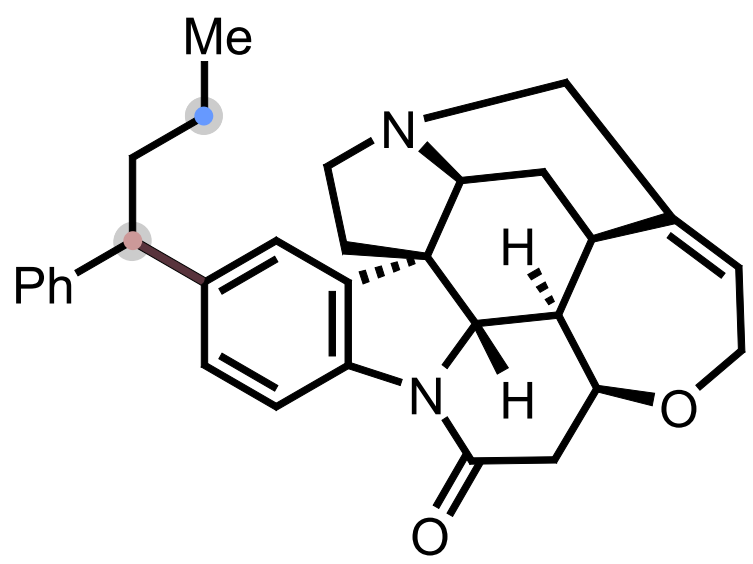

3aq

The titled compound **3aq** was prepared according to *Method B* described in *General Procedure 1* from 2,4,6-triphenyl-1-(4-phenylbutan-2-yl)pyridin-1-ium tetrafluoroborate **1a** (52.7 mg, 0.100 mmol, 1.00 equiv) and (3a^1*R*,6a*S*,6a^1*S*,12a*S*,12a^1*R*)-8-bromo-2,3a^1,5a,6,6a^1,12,12a,12a^1-octahydro-4*H*,11*H*-1-oxa-5,10b-diaza-5,6a-ethanoazuleno[1,8,7-*bcd*]fluoranthen-11-one **2aq** (82.6 mg, 0.200 mmol, 2.00 equiv). (*Note*: The regioselectivity (16:1 rr) of the reaction was determined by ^{1}H spectroscopic analysis of the crude reaction mixture of a duplicate run, which passed through a short silica plug, eluted with 8:2 dichloromethane/methanol, and concentrated *in vacuo*.) The crude reaction mixture was purified by flash column chromatography using 100:0 → 80:20 dichloromethane/methanol as an eluent followed by preparative HPLC (XBridge BEH C18 OBD column, 50% to 90% MeCN in H_2O with 0.1% NH_4OH) to yield compound **3aq** as a white solid in 48% yield (22 mg, 0.048 mmol).

^{1}H NMR (500 MHz, $CDCl_3$): 7.97 (d, *J* = 8.3 Hz, 1H), 7.29 – 7.13 (m, 6H, overlaps with $CHCl_3$), 7.01 (d, *J* = 9.7, 1H), 5.9 (br s, 1H), 4.29 – 4.25 (m, 1H), 4.17 – 4.10 (m, 2H), 4.06 (dd, *J* = 13.9, 6.1 Hz, 1H), 3.90 – 3.84 (m, 2H), 3.75 (d, *J* = 13.2 Hz, 1H), 3.35 – 3.24 (m, 1H), 3.15 – 3.08 (m, 2H), 2.89 (q, *J* = 7.1 Hz, 1H), 2.78 (d, *J* = 15.5 Hz, 1H), 2.65 (dd, *J* = 17.4, 3.3 Hz, 1H), 2.42 – 2.33 (m, 1H), 2.02 – 1.93 (m, 2H), 1.89 (d, *J* = 7.3 Hz, 2H), 1.47 (d, *J* = 14.4 Hz, 1H), 1.30 – 1.21 (m, 2H, overlaps with grease), 0.91 (t, *J* = 7.3 Hz, 3H).

^{13}C NMR (126 MHz, $CDCl_3$): 169.1, 145.4, 145.3, 141.9 (br), 140.5, 129.6, 128.6, 128.55, 128.54, 128.4 (br), 127.99, 127.97, 126.2, 121.7, 121.6, 116.2, 77.8, 64.7, 60.5, 60.40, 60.35, 60.30, 52.8, 52.1, 51.01, 50.96, 50.44, 50.43, 48.2, 42.9 (br), 42.5, 38.5, 38.3, 31.7, 29.9, 26.8, 21.30, 21.29, 14.20, 14.18. Mixture of diastereomers.

HRMS (ESI): *m*/*z* calculated for $C_{31}H_{35}N_2O_2$ ($[M + H]^+$): 467.2699, found: 467.2702.

2-(2,6-Dioxopiperidin-3-yl)-4-((*R*)-1-phenylbutyl)isoindoline-1,3-dione **3ar**:

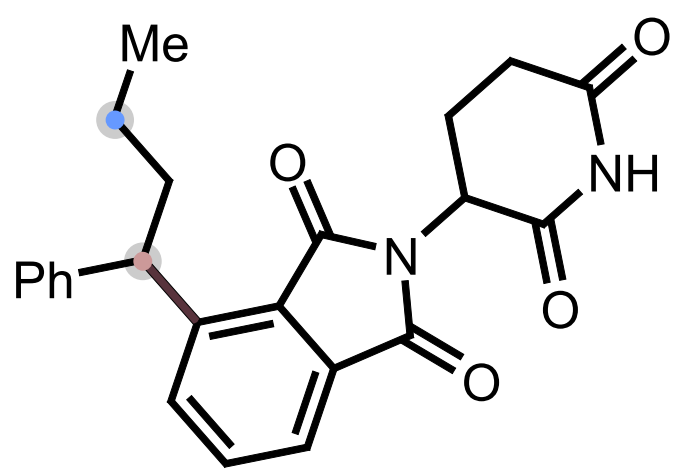

3ar

The titled compound **3ar** was prepared according to *Method B* described in *General Procedure 1* from 2,4,6-triphenyl-1-(4-phenylbutan-2-yl)pyridin-1-ium tetrafluoroborate **1a** (52.7 mg, 0.100 mmol, 1.00 equiv) and 4-bromo-2-(2,6-dioxopiperidin-3-yl)isoindoline-1,3-dione **2ar** (67.4 mg, 0.200 mmol, 2.00 equiv). (*Note*: The reaction was run at 0.1 M concentration, instead of 0.2 M, to maximize the solubility of the starting material *Note*: The regioselectivity (>20:1 rr) of the reaction was determined by ^{1}H spectroscopic analysis of the crude reaction mixture of a duplicate run, which passed through a short silica plug, eluted with 6:4 hexane/ethyl acetate, and concentrated *in vacuo*.) The crude reaction mixture was purified by flash column chromatography using 100:0 → 75:25 hexane/ethyl acetate as an eluent followed by preparative HPLC (XBridge BEH C18 OBD column, 75% to 95% MeCN in H_2O with 0.1% NH_4OH) to yield compound **3ar** as a colorless oil in 66% yield (25.8 mg, 0.066 mmol).

^{1}H NMR (500 MHz, $CDCl_3$): δ 7.98 (br, 1H), 7.77 – 7.78 (m, 2H), 7.62 (dd, *J* = 7.5, 1.6 Hz, 1H), 7.30 (t, *J* = 7.5 Hz, 2H), 7.23 – 7.19 (m, 3H), 4.96 (dd, *J* = 12.4, 5.3 Hz, 1H), 4.07 (t, *J* = 7.8 Hz, 1H), 2.95 – 2.68 (m, 3H), 2.19 – 2.00 (m, 3H), 1.35 – 1.26 (m, 2H), 0.94 (t, *J* = 7.3 Hz, 3H).

^{13}C NMR (126 MHz, $CDCl_3$): δ 170.8, 168.0, 167.6, 167.3, 153.7, 143.4, 134.4, 132.3, 129.0, 129.7, 127.9, 127.0, 124.1, 123.2, 51.5, 49.4, 37.6, 31.6, 22.8, 21.1, 14.1.

HRMS (ESI): *m/z* calculated for $C_{23}H_{23}N_2O_4^+$ ([M + H]$^+$): 391.1653, found: 391.1652.

(*Z*)-3-(2-(1-(4-Fluorophenyl)propyl)dibenzo[b,e]oxepin-11(6H)-ylidene)-N,N-dimethylpropan-1-amine **3as:**

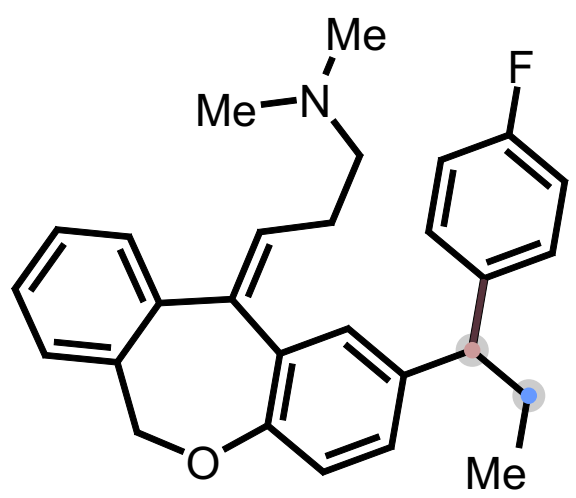

The titled compound **3as** was prepared according to *Method A* described in *General Procedure 2* from 1-(1-(1-(4-chlorobenzoyl)-5-methoxy-2-methyl-1H-indol-3-yl)propan-2-yl)-2,4,6-triphenylpyridin-1-ium tetrafluoroborate **1as** (35.7 mg, 0.050 mmol, 1.00 equiv) and 1-bromo-4-fluorobenzene (11.0 µL, 0.100 mmol, 2.00 equiv). The regioselectivity (>10:1 rr) of the reaction was determined by ^{19}F spectroscopic analysis of the crude reaction mixture. The crude reaction mixture was purified by flash column chromatography using 100:0 → 90:10 DCM/MeOH as an eluent followed by reverse phase column chromatography using 40% to 100% MeCN in H_2O to yield compound **3as** as a white solid in 43% yield (9.0 mg, 0.022 mmol).

^{1}H NMR (400 MHz, $CDCl_3$) δ 7.34 – 7.24 (m, 4H, overlaps with $CDCl_3$), 7.24 – 7.14 (m, 2H), 7.06 (dd, *J* = 8.4, 2.3 Hz, 1H), 6.97 (tt, *J* = 8.8, 3.2 Hz, 2H), 6.87 (d, *J* = 2.3 Hz, 1H), 6.82 (d, *J* = 8.4 Hz, 1H), 5.60 (t, *J* = 7.0 Hz, 1H), 5.19 (br s, 1H), 3.70 (t, *J* = 7.8 Hz, 1H), 2.79 (br s, 1H), 2.66 (q, *J* = 7.4 Hz, 2H), 2.47 (s, 6H), 2.07 – 1.93 (m, 2H), 0.87 (t, *J* = 7.3 Hz, 3H).

^{13}C NMR (126 MHz, $CDCl_3$) δ 161.2 (d, *J* = 244.2 Hz), 153.9, 144.3, 142.5, 141.2 (d, *J* = 3.3 Hz), 137.5, 133.6, 130.1 (br), 129.22 (d, *J* = 7.7 Hz), 129.1, 128.8, 127.9, 127.4, 126.3, 123.9, 119.9, 115.2 (d, *J* = 21.0 Hz), 70.6, 57.9, 51.5, 43.4, 29.7, 28.6, 12.7. (1 aromatic signal is not observed due to signal broadening).

^{19}F NMR (282 MHz, $CDCl_3$): δ -117.28.

HRMS (ESI): *m/z* calculated for $C_{28}H_{31}FNO^+$ ([M + H]$^+$): 416.2384, found: 416.2372.

2-(1-(4-Fluorophenyl)butyl)-6-methoxynaphthalene **3at:**

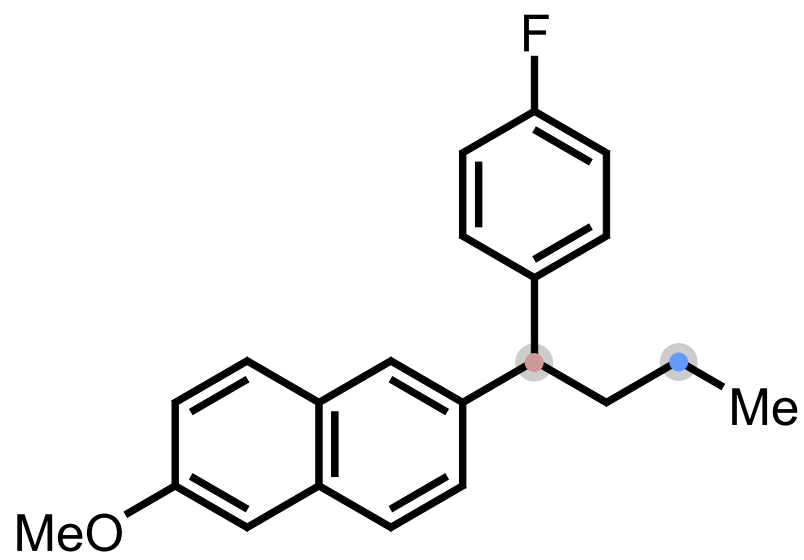

3at

The titled compound **3at** was prepared according to *Method A* described in *General Procedure 2* from 1-(4-(6-methoxynaphthalen-2-yl)butan-2-yl)-2,4,6-triphenylpyridin-1-ium tetrafluoroborate **1at** (32.0 mg, 0.050 mmol, 1.00 equiv) and 1-bromo-4-fluorobenzene (11.0 µL, 0.100 mmol, 2.00 equiv). The regioselectivity (20:1 rr) of the reaction was determined by ^{19}F spectroscopic analysis of the crude reaction mixture. The crude reaction mixture was purified by flash column chromatography using 100:0 → 98:2 pentane/diethyl ether as an eluent followed by preparative thin layer chromatography (98:2 pentane/diethyl ether) to yield compound **3at** as a colorless oil in 58% yield (9.0 mg, 0.029 mmol).

^{1}H NMR (300 MHz, $CDCl_3$): δ 7.67 (dd, J = 13.8, 8.7 Hz, 2H), 7.62 – 7.58 (m, 1H), 7.28 (d, *J* = 1.9 Hz, 1H), 7.25 – 7.19 (m, 2H), 7.16 – 7.07 (m, 2H), 6.96 (tt, *J* = 8.7, 3.1 Hz, 2H), 4.02 (t, *J* = 7.8 Hz, 1H), 3.90 (s, 3H), 2.08 (p, *J* = 7.5 Hz, 2H), 1.30 (h, *J* = 7.8 Hz, 2H), 0.94 (t, *J* = 7.3 Hz, 3H).

^{13}C NMR (126 MHz, $CDCl_3$): δ 161.39 (d, *J* = 243.9 Hz), 157.5, 141.25 (d, *J* = 3.3 Hz), 140.4, 133.3, 129.43 (d, *J* = 7.6 Hz), 129.3, 129.1, 127.3, 127.1, 125.8, 118.9, 115.22 (d, *J* = 21.0 Hz), 105.8, 55.5, 50.2, 38.0, 21.3, 14.2.

^{19}F NMR (282 MHz, $CDCl_3$): δ -117.53.

HRMS (EI): *m/z* calculated for $C_{21}H_{21}FO^+$ ($[M]^+$): 308.1576, found: 308.1570.

4-(1-(4-Fluorophenyl)ethyl)-2-methoxyphenol **3au:**

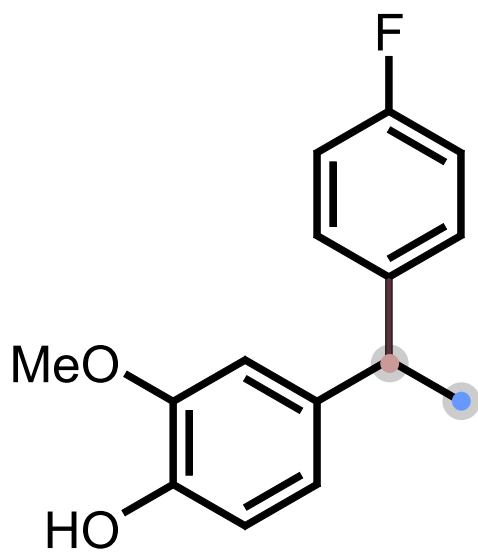

3au

The titled compound **3au** was prepared according to *Method C* described in *General Procedure 2* from 7-(4-((difluoro-l3-methyl)-l2-fluoraneyl)phenyl)-14-(4-hydroxy-3-methoxyphenethyl)-5,6,8,9-tetrahydrodibenzo[c,h]acridin-14-ium trifluoromethanesulfonate **Acridin5** (36.4 mg, 0.050 mmol, 1.00 equiv) and 1-bromo-4-fluorobenzene (11.0 µL, 0.100 mmol, 2.00 equiv). The regioselectivity (16:1 rr) of the reaction was determined by ^{19}F spectroscopic analysis of the crude reaction mixture. The crude reaction mixture was purified by flash column chromatography using 100:0 → 90:10 hexane/ethyl acetate as an eluent followed by preparative thin layer chromatography (90:10 pentane/diethyl ether) to yield compound **3au** as a colorless oil in 65% yield (8.0 mg, 0.033 mmol) as a mixture of regioisomers (10:1 regioisomeric ratio of **3au**/**4au**).

^{1}H NMR (500 MHz, $CDCl_3$): δ 7.20 – 7.12 (m, 2H), 6.96 (tt, *J* = 9.0, 3.1 Hz, 2H), 6.85 (d, *J* = 8.1 Hz, 1H), 6.72 (dd, *J* = 8.2, 2.0 Hz, 1H), 6.65 (d, *J* = 2.1 Hz, 1H), 4.07 (q, *J* = 7.2 Hz, 1H), 3.83 (s, 3H), 1.59 (d, *J* = 7.2 Hz, 3H).

^{13}C NMR (126 MHz, $CDCl_3$): δ 161.36 (d, *J* = 243.9 Hz), 146.6, 144.1, 142.52 (d, *J* = 3.3 Hz), 138.3, 128.99 (d, *J* = 7.9 Hz), 120.1, 115.17 (d, *J* = 21.0 Hz), 114.3, 110.4, 56.0, 43.8, 22.4.

^{19}F NMR (282 MHz, $CDCl_3$): δ -117.60.

HRMS (EI): *m/z* calculated for $C_{15}H_{15}FO_2^+$ ($[M]^+$): 246.1056, found: 246.1054.

3-(4-Fluorophenyl)-3-phenylpropan-1-ol **3av:**

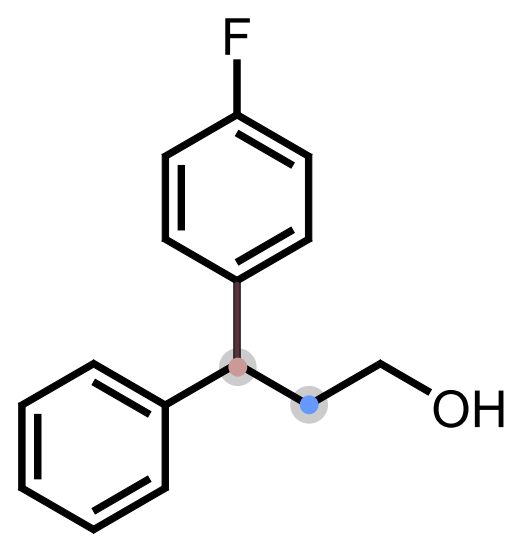

3av

The titled compound **3av** was prepared according to *Method A* described in *General Procedure 2* from 1-(1-hydroxy-3-phenylpropan-2-yl)-2,4,6-triphenylpyridin-1-ium tetrafluoroborate **1av** (52.9 mg, 0.100 mmol, 1.00 equiv) and 1-bromo-4-fluorobenzene (22.0 µL, 0.200 mmol, 2.00 equiv). The regioselectivity (>20:1 rr) of the reaction was determined by ^{19}F spectroscopic analysis of the crude reaction mixture. The crude reaction mixture was purified by flash column chromatography using 100:0 → 80:20 hexane/ethyl acetate as an eluent to yield compound **3av** as a white solid in 39% yield (9.0 mg, 0.030 mmol). NMR spectra are in accordance with those reported in the literature.[21]

^{1}H NMR (500 MHz, $CDCl_3$): δ 7.32 – 7.26 (m, 2H), 7.26 – 7.17 (m, 5H), 6.97 (tt, *J* = 8.6, 3.0 Hz, 2H), 4.14 (t, *J* = 7.9 Hz, 1H), 3.61 (t, *J* = 6.4 Hz, 2H), 2.36 – 2.23 (m, 2H), 1.26 (br s, 1H).

^{13}C NMR (126 MHz, $CDCl_3$): δ 161.52 (d, *J* = 244.4 Hz), 144.4, 140.36 (d, *J* = 3.2 Hz), 129.37 (d, *J* = 7.8 Hz), 128.8, 127.9, 126.6, 115.43 (d, *J* = 21.2 Hz), 61.1, 46.6, 38.4.

4-(1-(4-Fluorophenyl)butyl)phenyl 3-(6-(1-(2,2-difluorobenzo[*d*][1,3]dioxol-5-yl)cyclopropane-1-carboxamido)-3-methylpyridin-2-yl)benzoate **3aw**:

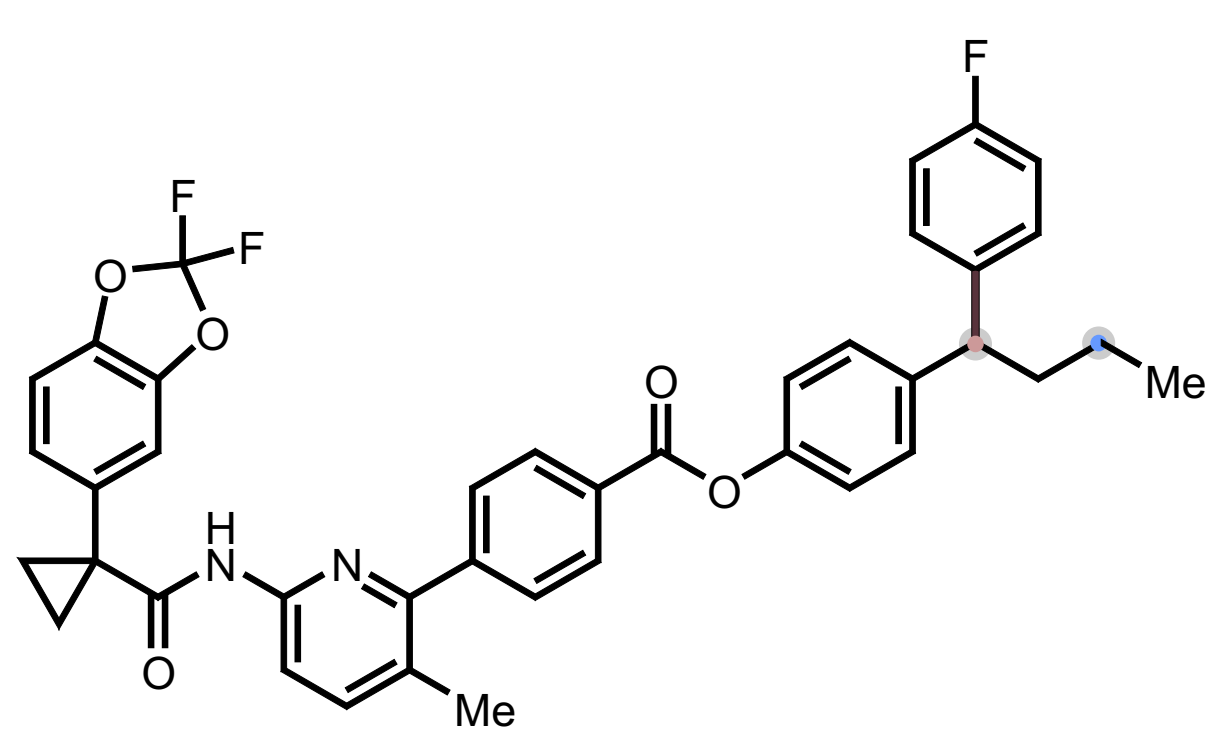

3aw

The titled compound **3aw** was prepared according to *Method B* described in *General Procedure 1* from 1-(4-(4-((4-(6-(1-(2,2-difluorobenzo[d][1,3]dioxol-5-yl)cyclopropane-1-carboxamido)-3-methylpyridin-2-yl)benzoyl)oxy)phenyl)butan-2-yl)-2,4,6-triphenylpyridin-1-ium tetrafluoroborate **1aw** (48.4 mg, 0.050 mmol, 1.00 equiv) and 1-bromo-4-fluorobenzene **2a** (11.0 µL, 0.100 mmol, 2.00 equiv). (*Note*: The regioselectivity

(20:1 rr) of the reaction was determined by ^{19}F spectroscopic analysis of the crude reaction mixture.) The crude reaction mixture was purified by flash column chromatography using 70:30 hexane/ethyl acetate as an eluent followed by preparative HPLC (XBridge BEH C18 OBD column, 75% to 95% MeCN in H_2O with 0.1% NH_4OH) to yield compound **3aw** as a colorless oil in 50% yield (17.0 mg; 0.025 mmol).

^{1}H NMR (500 MHz, $CDCl_3$): δ 8.23 (t, *J* = 1.8 Hz, 1H), 8.19 (dt, *J* = 7.7, 1.4 Hz, 1H), 8.11 (d, *J* = 8.4 Hz, 1H), 7.70 (br s, 1H), 7.68 (dt, *J* = 7.7, 1.4 Hz, 1H), 7.60 (d, *J* = 8.4 Hz, 1H), 7.56 (t, *J* = 7.7 Hz, 1H), 7.26 (dt, *J* = 8.5, 1.8 Hz, 2H, overlaps with $CHCl_3$), 7.23 (dd, *J* = 8.2, 1.8 Hz, 1H) 7.21 – 7.17 (m, 3H), 7.11 (dt, *J* = 8.7, 2.7 Hz, 2H), 7.06 (d, *J* = 8.2 Hz, 1H), 6.97 (tt, *J* = 8.8, 3.0 Hz, 2H), 3.93 (t, *J* = 7.9 Hz, 1H), 2.28 (s, 3H), 2.05 – 1.96 (m, 2H), 1.75 (q, *J* = 3.9 Hz, 2H), 1.35 – 1.27 (m, 2H, overlaps with grease), 1.16 (q, *J* = 3.9 Hz, 2H), 0.94 (t, *J* = 7.3 Hz, 3H).

^{13}C NMR (126 MHz, $CDCl_3$): δ 171.9, 165.0, 161.5 (d, *J* = 244.1 Hz), 155.2, 149.2 (d, *J* = 15.1 Hz), 144.3, 143.8, 143.0, 141.2, 140.8 (d, *J* = 3.2 Hz), 140.4, 135.0, 134.1, 131.8 (t, *J* = 256 Hz) 130.8, 129.9 (d, *J* = 11.4 Hz), 129.4 (d, *J* = 7.7 Hz), 128.9, 128.7, 127.1, 126.7, 121.6, 115.3 (d, *J* = 21.1 Hz), 113.2, 112.5, 110.3, 49.8, 38.2, 31.3, 29.9, 21.2, 19.3, 17.3, 14.1.

^{19}F NMR (282 MHz, $CDCl_3$): δ -49.62, -117.28.

HRMS (ESI): *m/z* calculated for $C_{40}H_{34}F_3N_2O_5$ ([M]+H): 679.2420, found: 679.2421.

4-(1-(4-Fluorophenyl)butyl)phenyl (2*S*)-2-(6-methoxynaphthalen-2-yl)propanoate **3ax**:

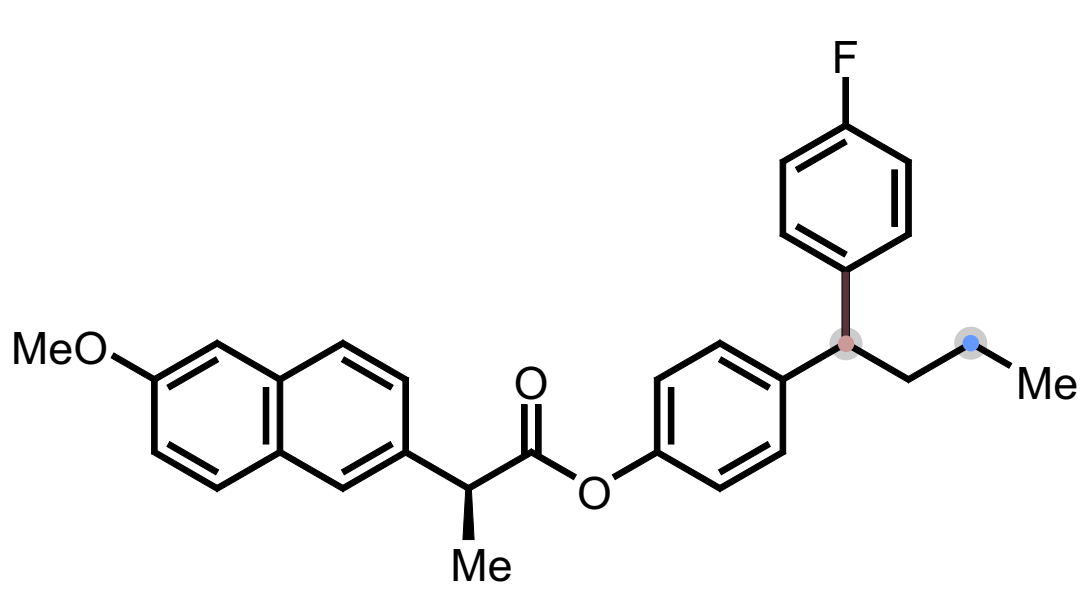

3ax

The titled compound **3ax** was prepared according to *Method A* described in *General Procedure 2* from 1-(4-(4-(((*S*)-2-(6-methoxynaphthalen-2-yl)propanoyl)oxy)phenyl)butan-2-yl)-2,4,6-triphenylpyridin-1-ium tetrafluoroborate **1ax**

(37.7 mg, 0.050 mmol, 1.00 equiv) and 1-bromo-4-fluorobenzene **2a** (11.0 μL, 0.100 mmol, 2.00 equiv). (*Note*: The regioselectivity (20:1 rr) of the reaction was determined by ^{19}F spectroscopic analysis of the crude reaction mixture.) The crude reaction mixture was purified by flash column chromatography using 100:0 → 85:15 hexane/ethyl acetate as an eluent followed by preparative HPLC (XBridge BEH C18 OBD column, 75% to 95% MeCN in H_2O with 0.1% NH_4OH) to yield **3ax** as a white solid in 82% yield (18.7 mg; 0.041 mmol).

^{1}H NMR (500 MHz, $CDCl_3$): δ 7.77 – 7.69 (m, 3H), 7.48 (dd, *J* = 8.5, 1.9 Hz, 1H), 7.18 – 7.10 (m, 6H), 6.93 (tt, *J* = 8.7, 2.2 Hz, 2H), 6.89 (dt, *J* = 8.7, 2.2 Hz, 2H), 4.07 (q, *J* = 7.1 Hz, 1H), 3.92 (s, 3H), 3.85 (t, *J* = 7.8 Hz, 1H), 1.93 (qt, *J* = 7.5, 2.1 Hz, 2H), 1.67 (d, *J* = 7.1 Hz, 3H), 1.29 – 1.18 (m, 2H), 0.89 (t, *J* = 7.3 Hz, 3H).

^{13}C NMR (126 MHz, $CDCl_3$): δ 173.4, 161.4 (d, *J* = 244.0 Hz), 157.9, 149.2, 142.7, 140.8 (d, *J* = 2.9 Hz), 135.3, 134.0, 129.5, 129.34, 129.27, 129.1, 128.7, 127.5, 126.3 (d, *J* = 2.1 Hz), 121.3, 119.2, 115.3 (d, *J* = 21.0 Hz), 105.8, 55.48, 49.75, 45.71, 38.17, 21.14, 18.64, 14.11.

^{19}F NMR (282 MHz, $CDCl_3$): δ -117.36.

HRMS (ESI): *m/z* calculated for $C_{30}H_{30}FO_3^+$ ($[M + H]^+$): 457.2174, found: 457.2171.

4-(1-(4-Fluorophenyl)butyl)phenyl 2-(3-cyano-4-isobutoxyphenyl)-4-methylthiazole-5-carboxylate **3ay**:

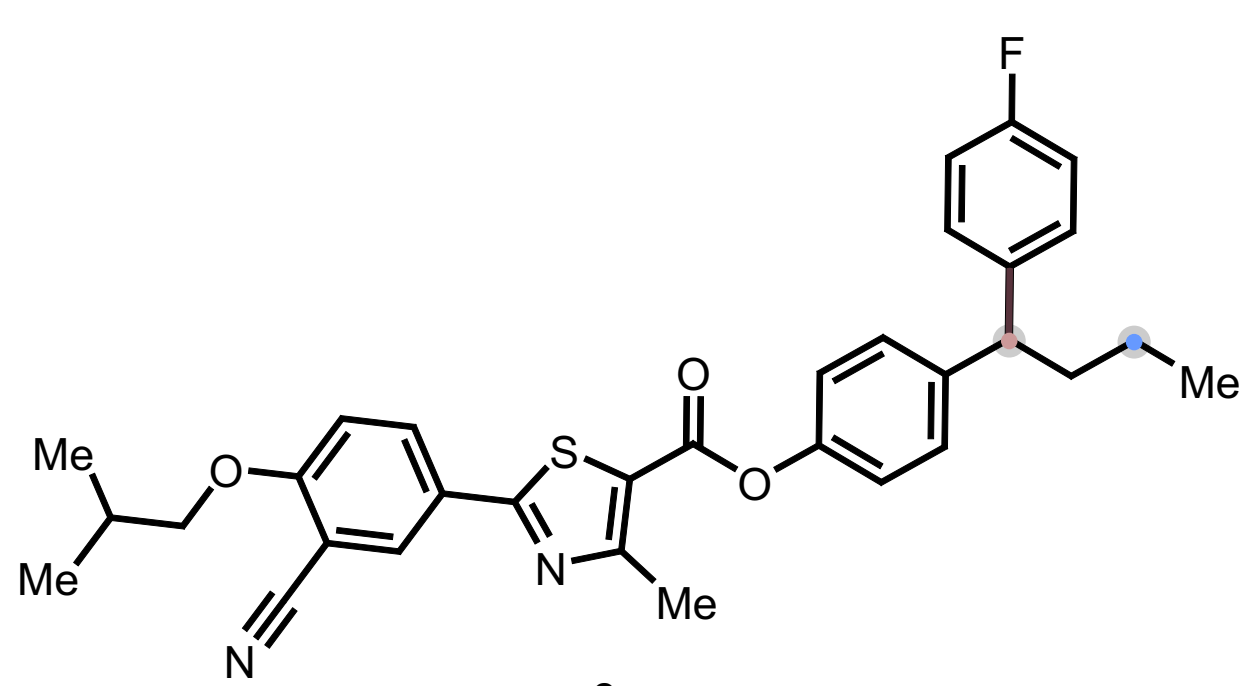

3ay

The titled compound **3ay** was prepared according to *Method A* described in *General Procedure 2* from 1-(4-(4-((2-(3-cyano-4-isobutoxyphenyl)-4-methylthiazole-5-carbonyl)oxy)phenyl)butan-2-yl)-2,4,6-triphenylpyridin-1-ium tetrafluoroborate

1ay (42.1 mg, 0.050 mmol, 1.00 equiv) and 1-bromo-4-fluorobenzene **2a** (11.0 μL, 0.100 mmol, 2.00 equiv). The regioselectivity of the reaction was determined by ^{19}F spectroscopic analysis of the crude reaction mixture. The crude reaction mixture was purified by flash column chromatography using 100:0 → 88:12 hexane/ethyl acetate as an eluent to yield compound **3ay** as a white solid in 82% yield (22.0 mg; 0.041 mmol).

^{1}H NMR (500 MHz, $CDCl_3$): δ 8.22 (d, *J* = 2.2 Hz, 1H), 8.12 (dd, *J* = 8.9, 2.2 Hz, 1H), 7.26 (dt, *J* = 8.6, 2.2 Hz, 2H, overlaps with $CHCl_3$), 7.20 – 216 (m, 2H), 7.12 (dt, *J* = 8.6, 2.2 Hz, 2H), 7.03 (d, *J* = 8.9 Hz, 1H), 6.97 (tt, *J* = 8.7, 3.0 Hz, 2H), 3.94 – 3.90 (m, 3H), 2.81 (s, 3H), 2.21 (sept, *J* = 13.6, 7.0 Hz, 1H), 2.00 (q, *J* = 7.7 Hz, 2H), 1.36 – 1.19 (m, 2H, overlaps with grease), 1.10 (d, *J* = 6.8 Hz, 6H), 0.93 (t, *J* = 7.3 Hz, 3H).

^{13}C NMR (126 MHz, $CDCl_3$): δ 168.3, 163.0 (d, *J* = 39.2 Hz), 162.4, 160.7, 160.50, 148.6, 143.3, 140.7 (d, *J* = 3.2 Hz), 132.6 (d, *J* = 52.8 Hz), 129.4 (d, *J* = 7.8 Hz), 129.0, 126.0, 121.6, 120.8, 115.5, 115.4, 115.3, 112.8, 103.3, 75.9, 49.8, 38.2, 28.3, 21.3, 19.2, 17.8, 14.2.

^{19}F NMR (471 MHz, $CDCl_3$): δ -117.22.

HRMS (ESI): *m/z* calculated for $C_{32}H_{32}FN_2O_3S^+$ ($[M + H]^+$): 543.2113, found: 543.2116.

7. Mechanistic Studies

7.1 Computational Studies

All DFT calculations were carried out with the Gaussian 16 program.[22] Geometry optimizations were performed at 298.15 K using M06-L[22] functional and def2-SVP[23] basis set in a gas-phase. Frequency calculations were performed at the same level of theory as used for geometry optimizations. Transition states all have single imaginary frequency and were verified by intrinsic reaction coordinate (IRC) calculations. With the optimized geometries of intermediates and transition states, single-point energy calculations were performed at 298.15 K using M06-L functional[22] and def2-TZVP[23] basis set with solvent correction using CPCM[24–26] model in *N,N*-dimethylacetamide (DMA) solvent.

Our initial mechanistic studies began with the evaluation of canonical mechanistic pathways (Figure S8). Nickel-catalyzed, cross-electrophile coupling reactions have been previously proposed to occur through multiple distinct mechanistic pathways: 1) the nickel(0) species reacts with the aryl bromide first by oxidative addition followed by radical capture to form a nickel(III) intermediate; 2) the nickel(0) species captures the alkyl radical first to form a nickel(I) alkyl intermediate, which then undergoes oxidative addition with the aryl bromide; 3) the nickel(I)-aryl species reduces **1a** and then traps the alkyl radical to form a cationic Ni(III) species; 4) the nickel(I)-aryl species traps the alkyl radical to form a Ni(II) species. To test whether these manifolds could lead to the formation of the migratory product, we evaluated the steps following the radical capture in each manifold using density functional theory (DFT) at the M06-L/def2-TZVP/CPCM(DMA)//M06-L/def2-SVP level of theory.

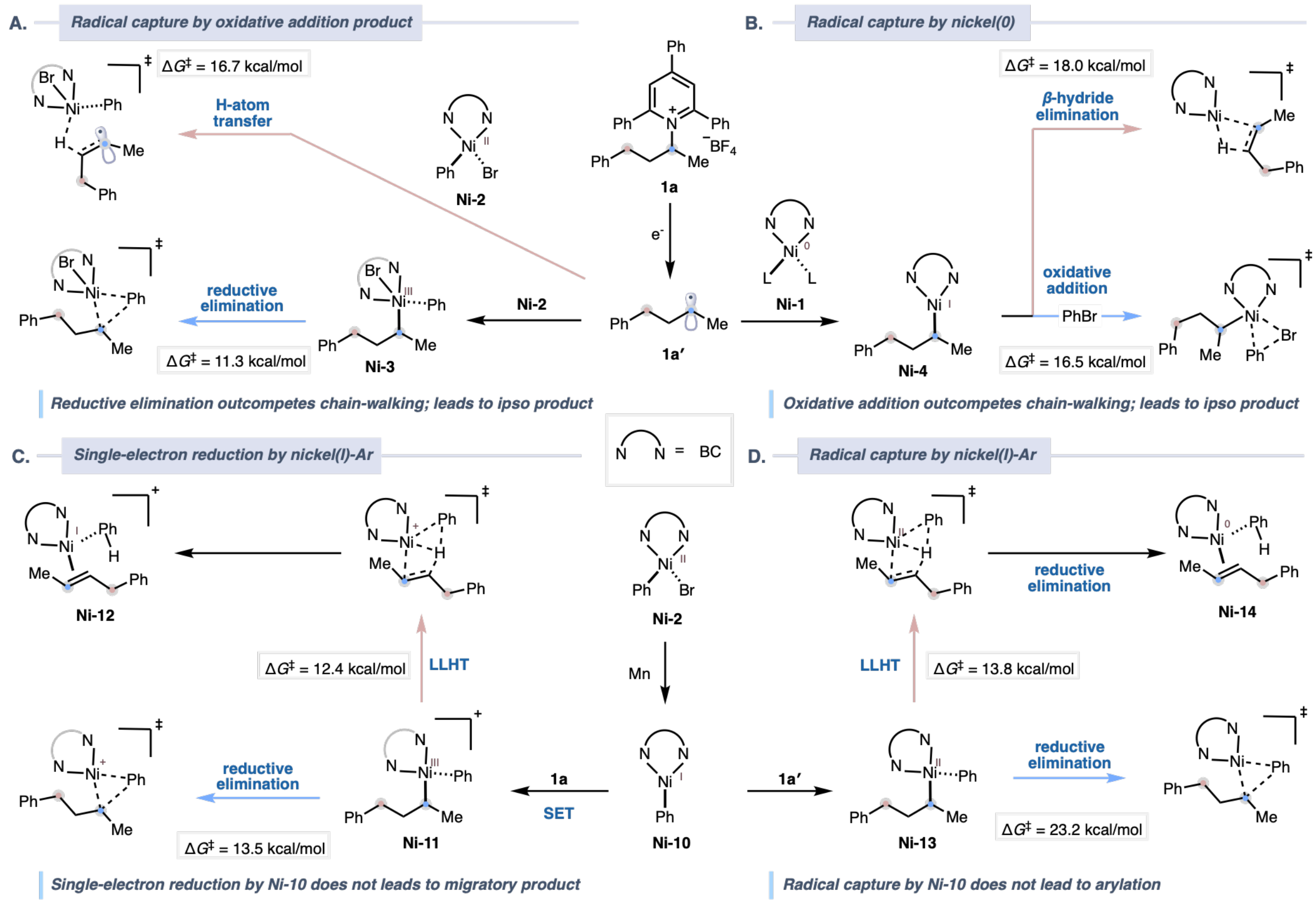

Figure S8: Summary of evaluation of canonical mechanisms.

Radical capture by oxidative addition product pathway:

The energy diagram of the radical capture of **1a′** by **Ni-2** pathway is shown below. Upon capture of alkyl radical **1a′** to form a Ni(III) intermediate **Ni-3**, a reductive elimination ($\Delta G^{\ddagger}$ = 2.0 kcal/mol) was kinetically favored to give the *ipso*-coupling product. We attempted to calculate the chain-walking pathway, which would lead to the migratory arylation product. First, we attempted to calculate the β-H elimination pathway involving **Ni-3** intermediate. Although the β-H elimination pathway from **Ni-3** was unable to be identified, **1a′** was found to undergo a hydrogen atom transfer (HAT) by **Ni-2**, which also led to the chain-walking intermediates. However, the free energy barrier for the HAT pathway ($\Delta G^{\ddagger}$ = 16.7 kcal/mol) indicated that it was less feasible. Moreover, an outer sphere reductive elimination would also give the *ipso*-coupling product with a higher energy barrier ($\Delta G^{\ddagger}$ = 10.3 kcal/mol). Overall, the radical capture of **1a′** by **Ni-2** may lead to the *ipso*-coupling product.

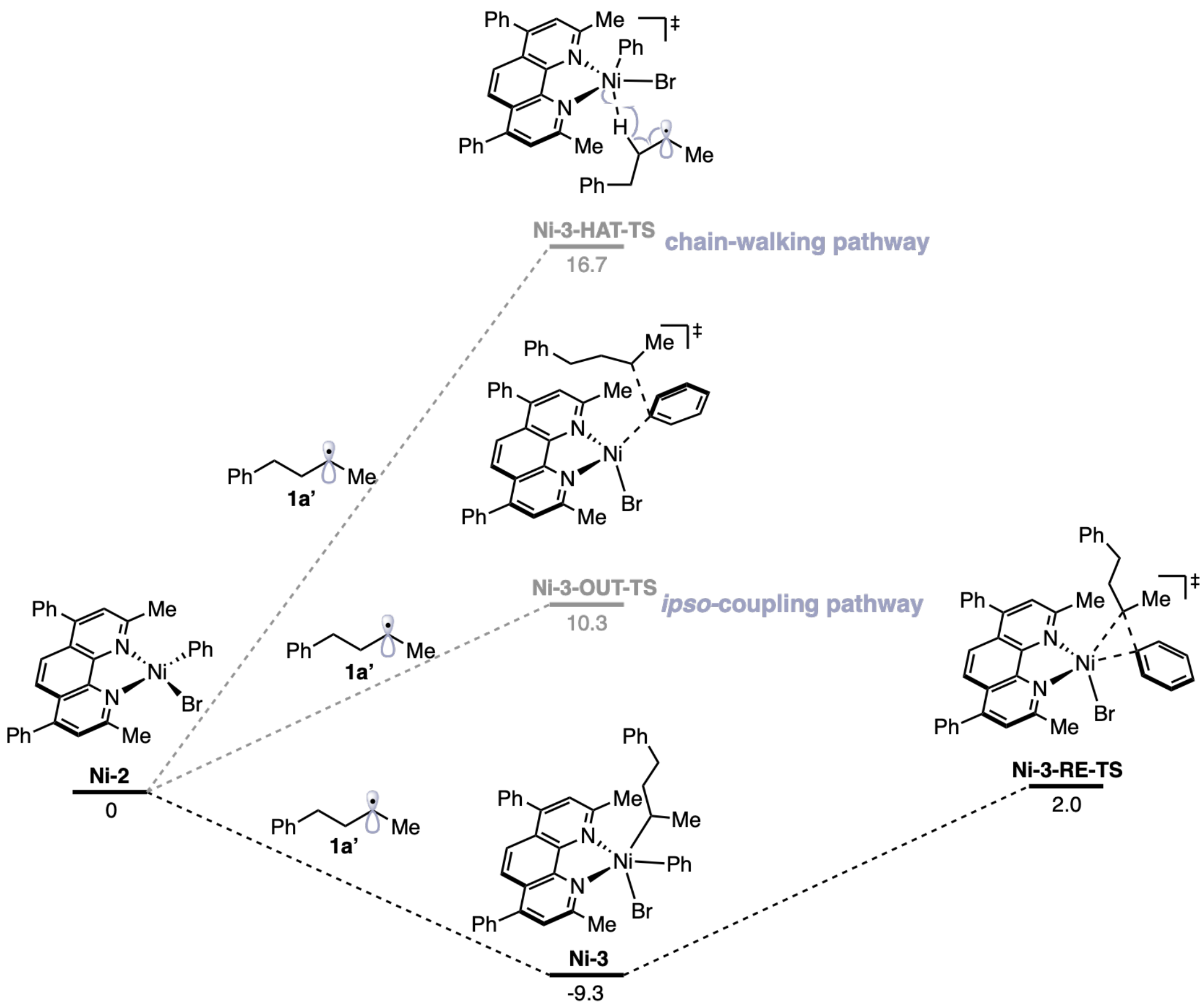

Figure S9: Energy diagram of radical capture of **1a′** by **Ni-2**. HAT: hydrogen atom transfer; OUT: outer sphere reductive elimination; RE: reductive elimination. M06-L/def2-TZVP/CPCM(DMA)//M06-L/def2-SVP. Gibbs free energies in kcal/mol.

Radical capture by nickel(0):

The energy diagram of the radical capture of **1a′** by **Ni-1** is shown below. Upon capture of alkyl radical **1a′** to form a Ni(I) intermediate **Ni-4**, the oxidative addition of PhBr has a slightly lower free energy barrier than β-H elimination leading to chain-walking ($\Delta G^{\ddagger}$: 16.5 kcal/mol vs. 18.0 kcal/mol). However, as discussed above the nickel(III) intermediate **Ni-3**, which would be formed upon oxidative addition was expected to undergo reductive elimination to form the *ipso*-coupling product. These results suggested the radical capture by nickel(0) may not explain the regioselectivity observed for the migratory product and instead may favor the *ipso* product.

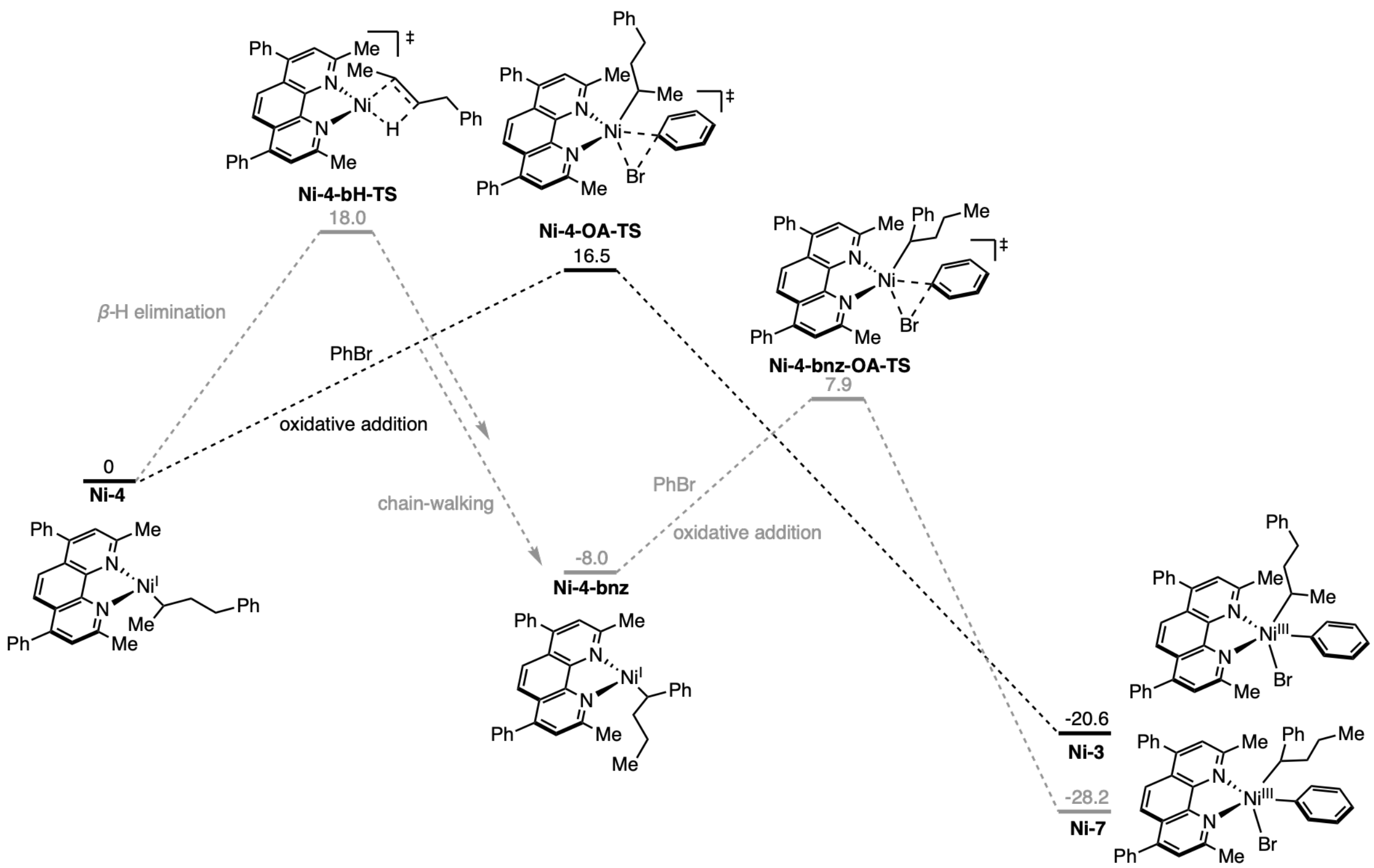

Figure S10: Energy diagram of radical capture pathway from **Ni-4**. bH: β-H elimination; OA: oxidative addition; bnz: benzylic. M06-L/def2-TZVP/CPCM(DMA)//M06-L/def2-SVP. Gibbs free energies in kcal/mol.

Single-electron reduction by nickel(I)-Ar:

Alternatively, the oxidative addition product **Ni-2** could undergo reduction to form **Ni-10**, a nickel(I)-aryl intermediate. Single-electron transfer (SET) from **Ni-10** to **1a** followed by

radical capture would result in a cationic Ni(III) intermediate, **Ni-11**, which could undergo either reductive elimination or β-hydride elimination. DFT calculation indicated that the barrier for reductive elimination was 13.5 kcal/mol. During the attempt to locate the β-hydride elimination transition state, the H-atom was found to undergo ligand-to-ligand hydride transfer (LLHT)[27] to aryl ligand to form **Ni-12**. The barrier for LLHT was found to be 12.4 kcal/mol. The results indicated that the intermediate **Ni-11** may not lead to the migratory product.

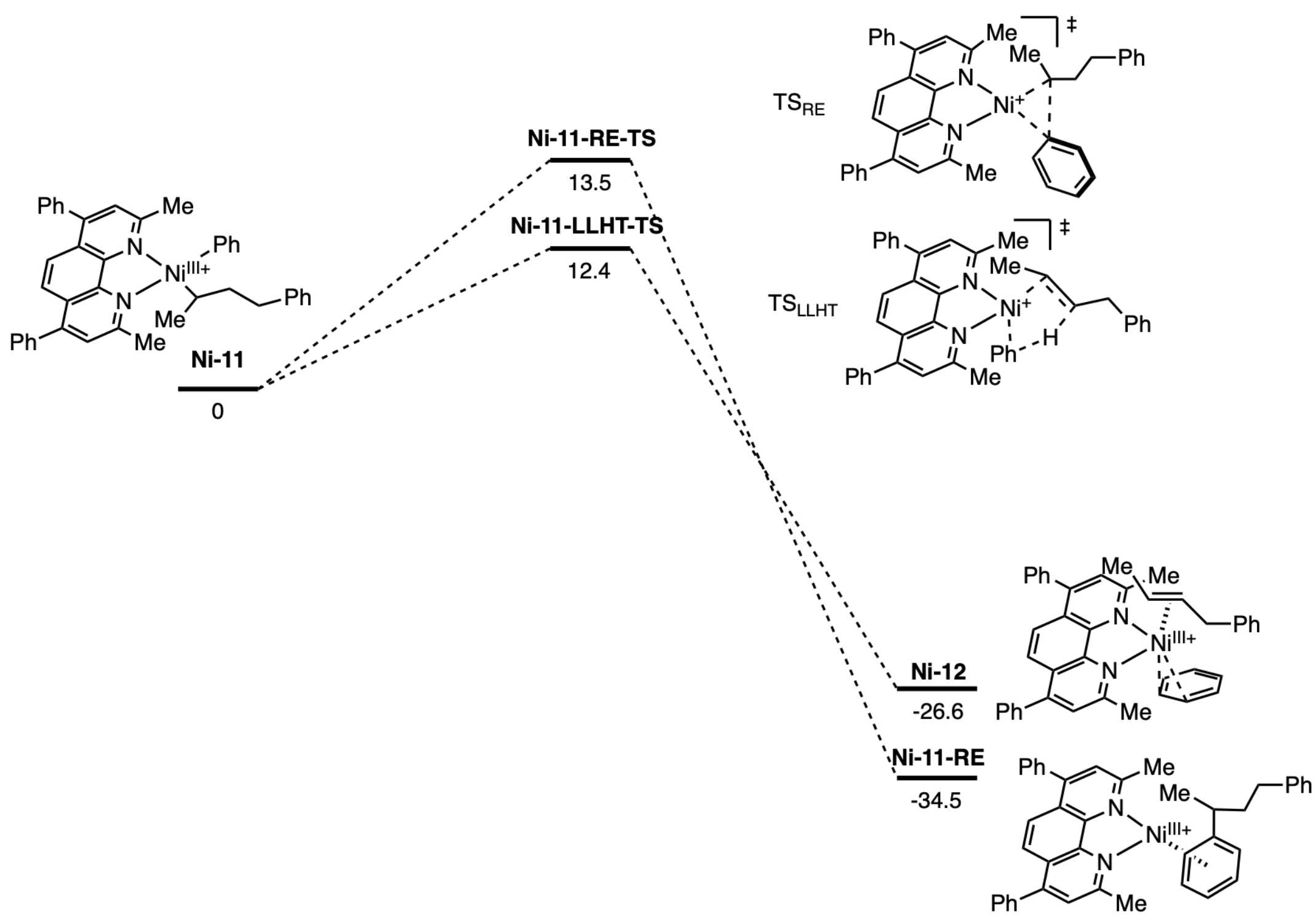

Figure S11: Energy diagram of cationic Ni(III) reductive elimination and LLHT pathways. RE: reductive elimination; LLHT: ligand-to-ligand hydride transfer. M06-L/def2-TZVP/CPCM(DMA)//M06-L/def2-SVP. Gibbs free energies in kcal/mol.

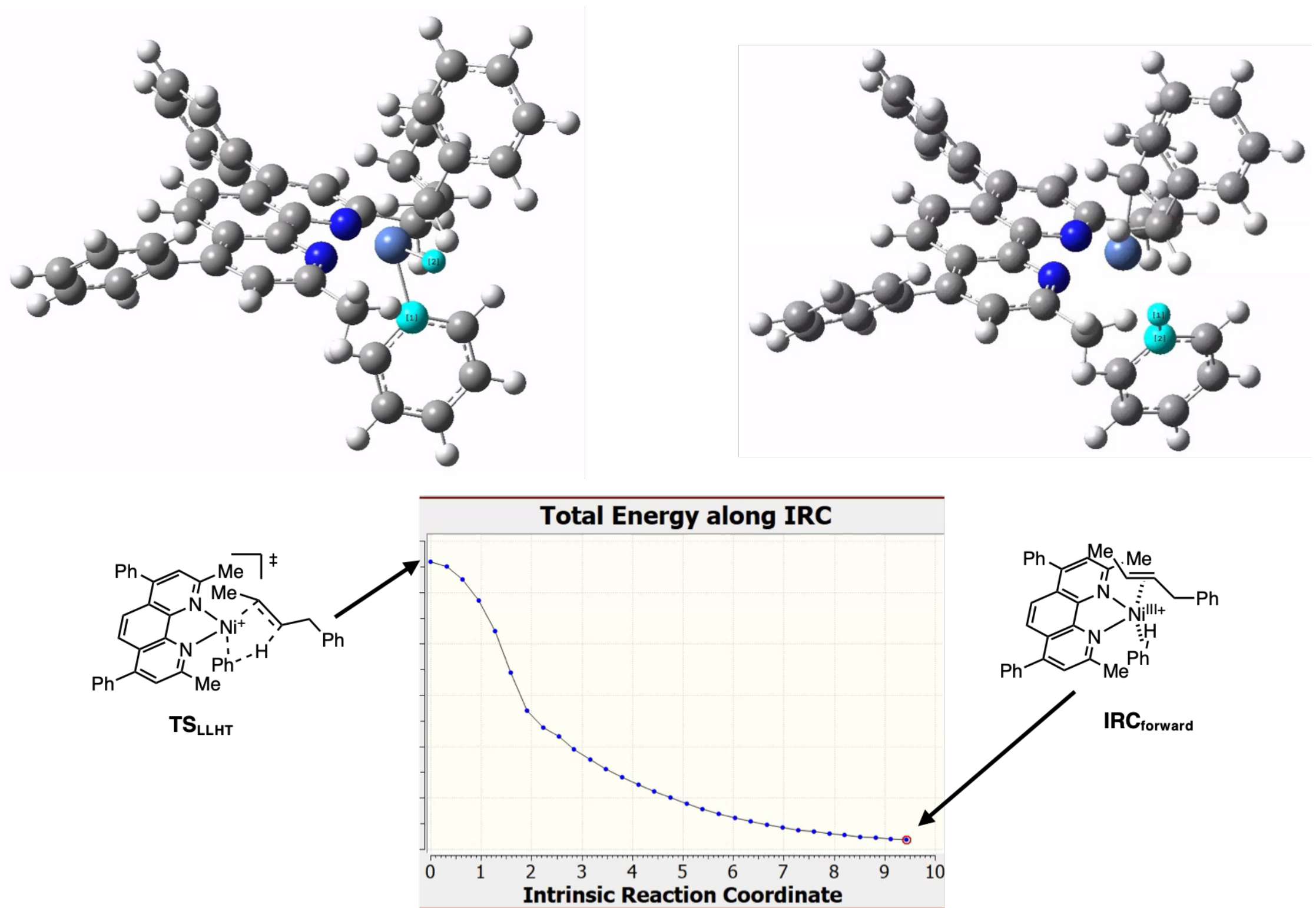

Figure S12: IRC calculation (forward) of LLHT transition state (TS_{LLHT}) from **Ni-11**.

Radical capture by nickel(I)-Ar:

In a related but distinct manifold, the radical capture of **1a′** by **Ni-10** would lead to a Ni(II) intermediate, **Ni-13.** Here, similar to the prior pathway, attempt to locate the *β*-hydride elimination transition state led to LLHT to form **Ni-14**. The barrier for LLHT was lower than that of reductive elimination from **Ni-13**. These results suggested that the migratory arylation product may not be formed through the intermediacy of **Ni-13**.

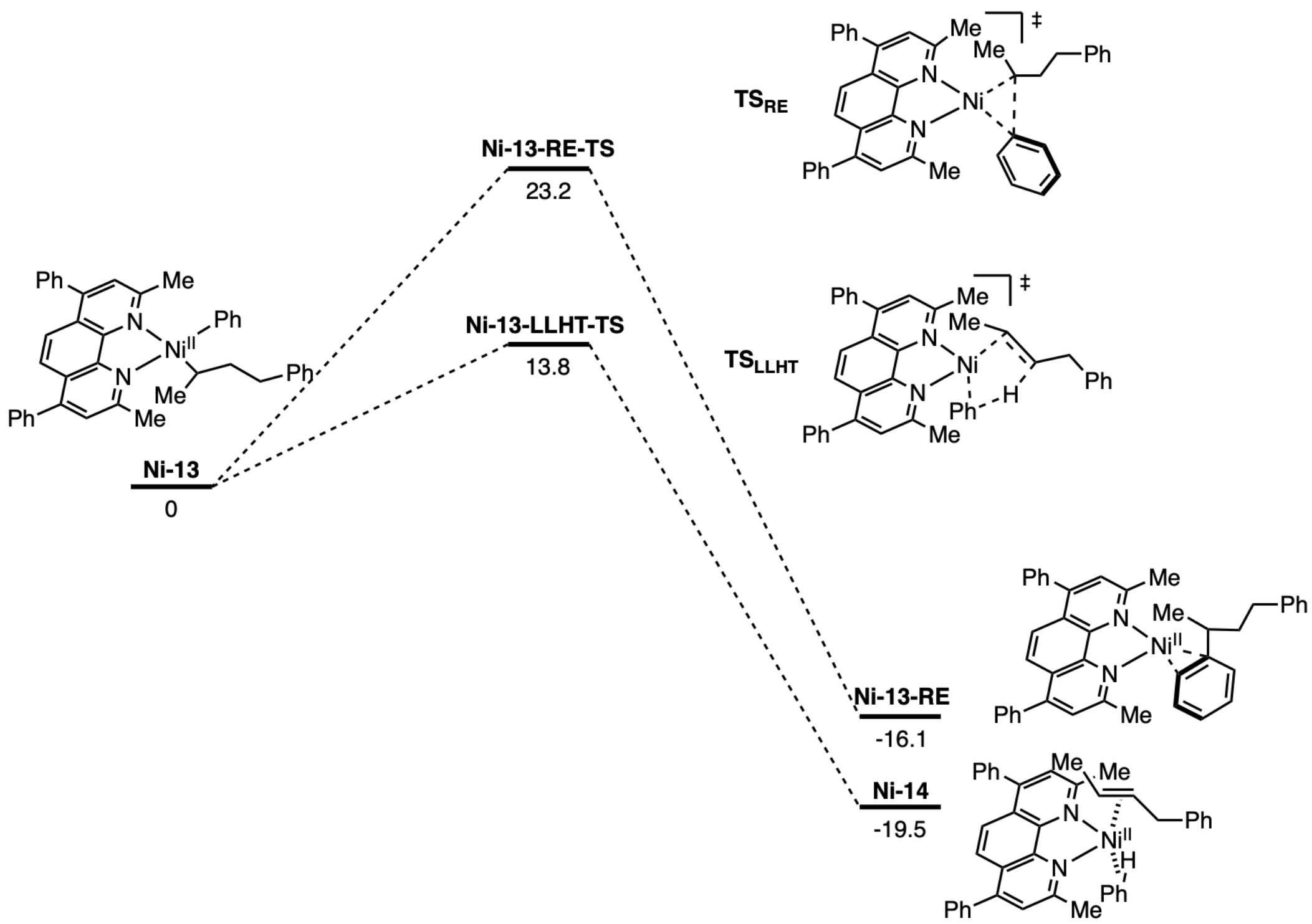

Figure S13: Energy diagram of reductive elimination and LLHT pathways from **Ni-13**. RE: reductive elimination; LLHT: ligand-to-ligand hydride transfer. M06-L/def2-TZVP/CPCM(DMA)//M06-L/def2-SVP. Gibbs free energies in kcal/mol.

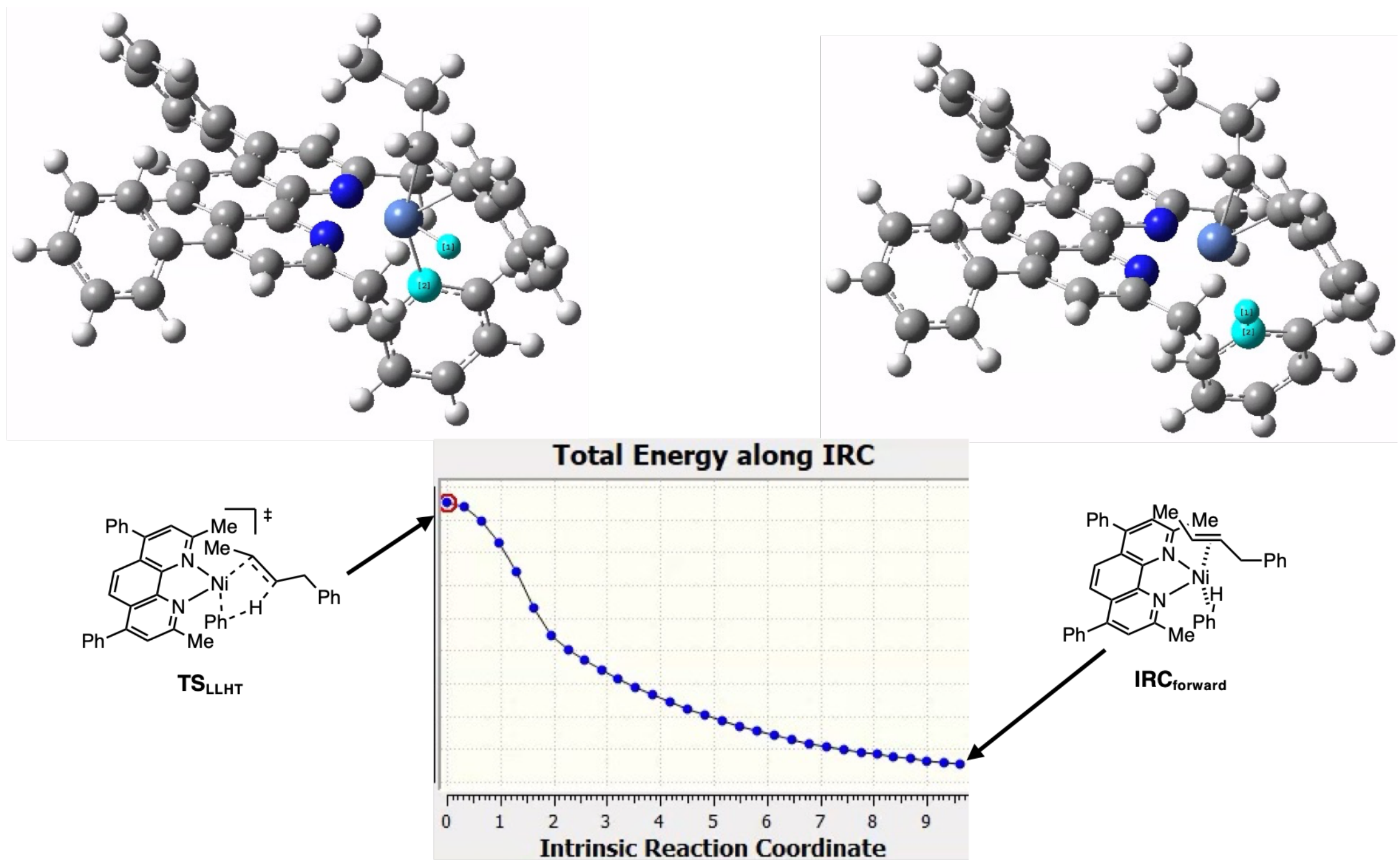

Figure S14: IRC calculation (forward) of LLHT transition state (TS$_{LLHT}$) from **Ni-13**.

Translocation of the alkyl radical upon radical capture by Ni(I):

<u>Translocation via cationic Ni(II) pathway:</u>

If the reduction of (BC)NiBr$_2$ to Ni(0) is relatively fast, Ni(0) can reduce Katrizky salt **1a**. This reduction of **1a** by Ni(0) would result in a cationic Ni(I) intermediate. Radical capture of **1′** by the cationic Ni(I) intermediate would lead to a cationic Ni(II) intermediate, **Ni-5** (where X could be BF$_4$ counterion). The energy diagram of the full chain-walking pathway of the cationic Ni(II) intermediate is shown below. Initial calculations were performed by using the cationic tricoordinate Ni(II)–alkyl intermediate, while the energy barriers became much higher, and the alkene-coordinated Ni(II)–H intermediates after β-H elimination were not found. A solvent coordination model (DMA) was then calculated, where the energy diagram is shown below. The energy difference between **Ni-5** and **Ni-6** suggests a benzylic regioisomer is thermodynamically favored by 8.5 kcal/mol.

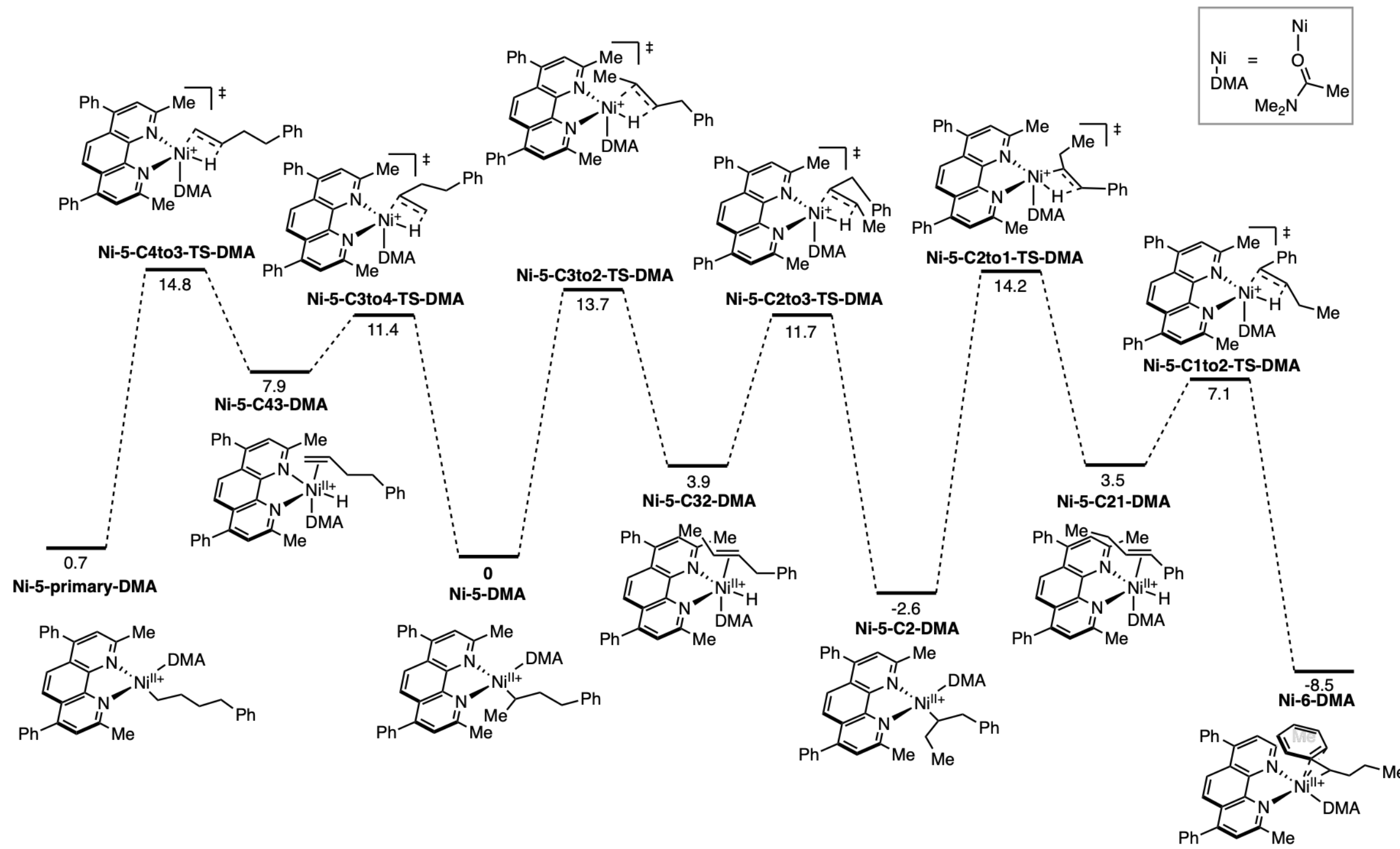

Figure S15A. Energy diagram of DMA-coordinated cationic Ni(II)–BC chain-walking pathway. M06-L/def2-TZVP/CPCM(DMA)//M06-L/def2-SVP. Gibbs free energies in kcal/mol.

Translocation via neutral Ni(II) pathway:

In an alternative scenario, if the reduction of (BC)Ni(II)Br_2 to Ni(0) by Mn were relatively slow and resulted in accumulation of (BC)Ni(I)Br, the radical capture of the transient radical **1a′** by (BC)Ni(I)Br would result in a corresponding neutral Ni(II) intermediate (**Ni-5**, where X = Br). The energy diagram of the full chain-walking pathway of the neutral Ni(II) intermediate is shown below. A sequence of β-H elimination and reinsertion enables the chain-walking process, ultimately forming the benzylic-substituted alkyl-Ni(II)-bromide intermediate. The energy difference between **Ni-5** and **Ni-6** suggests a benzylic regioisomer is thermodynamically favored by 9.0 kcal/mol.

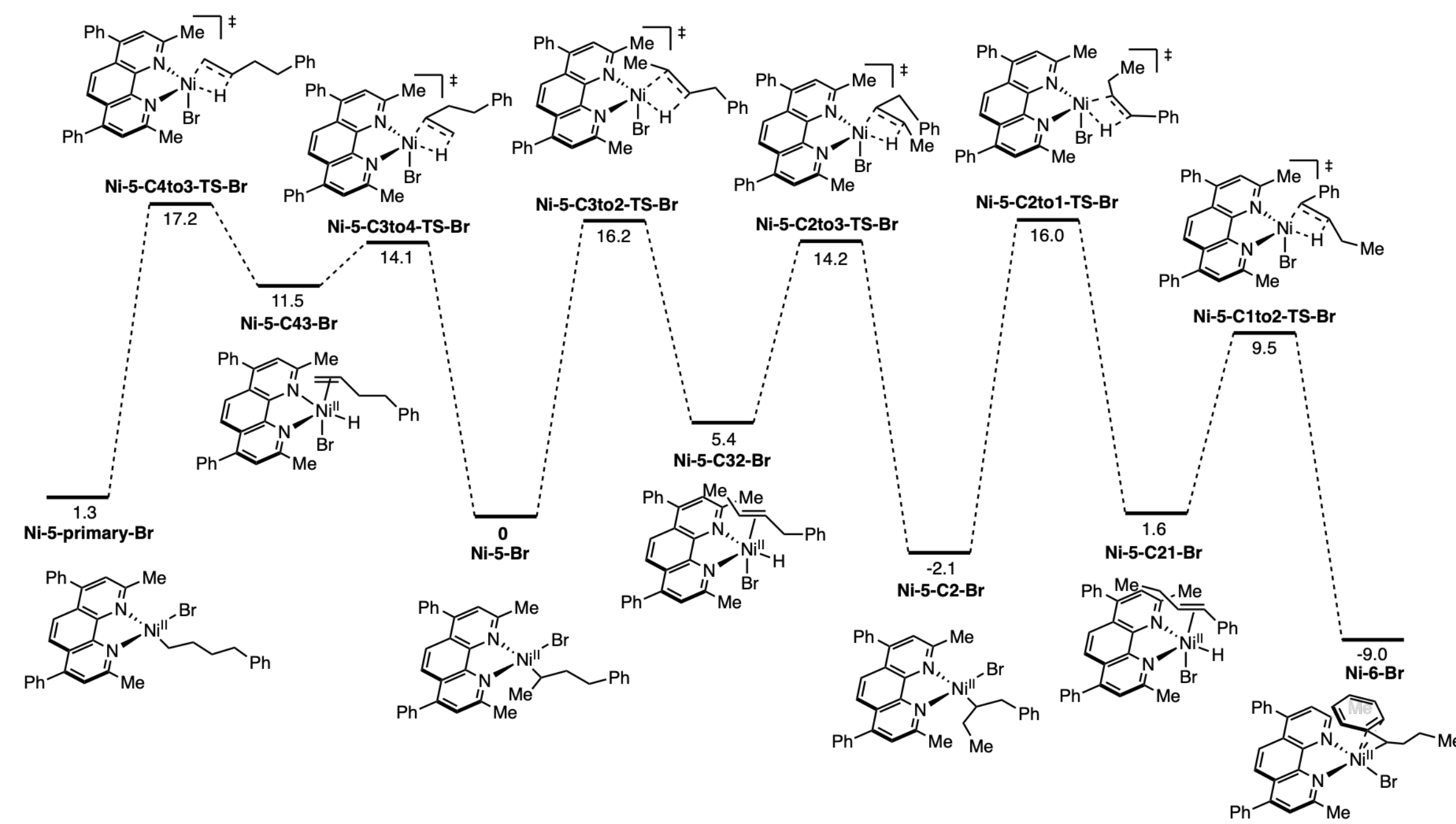

Figure S15B: Energy diagram of a neutral Ni(II) chain-walking pathway. M06-L/def2-TZVP/CPCM(DMA)//M06-L/def2-SVP. Gibbs free energies in kcal/mol.

BDFE of the newly formed C–C bonds in dihydropyridines:

The bond dissociation free energies (BDFEs) of the newly formed C–C bonds in dihydropyridines **6** and **7** are shown below.

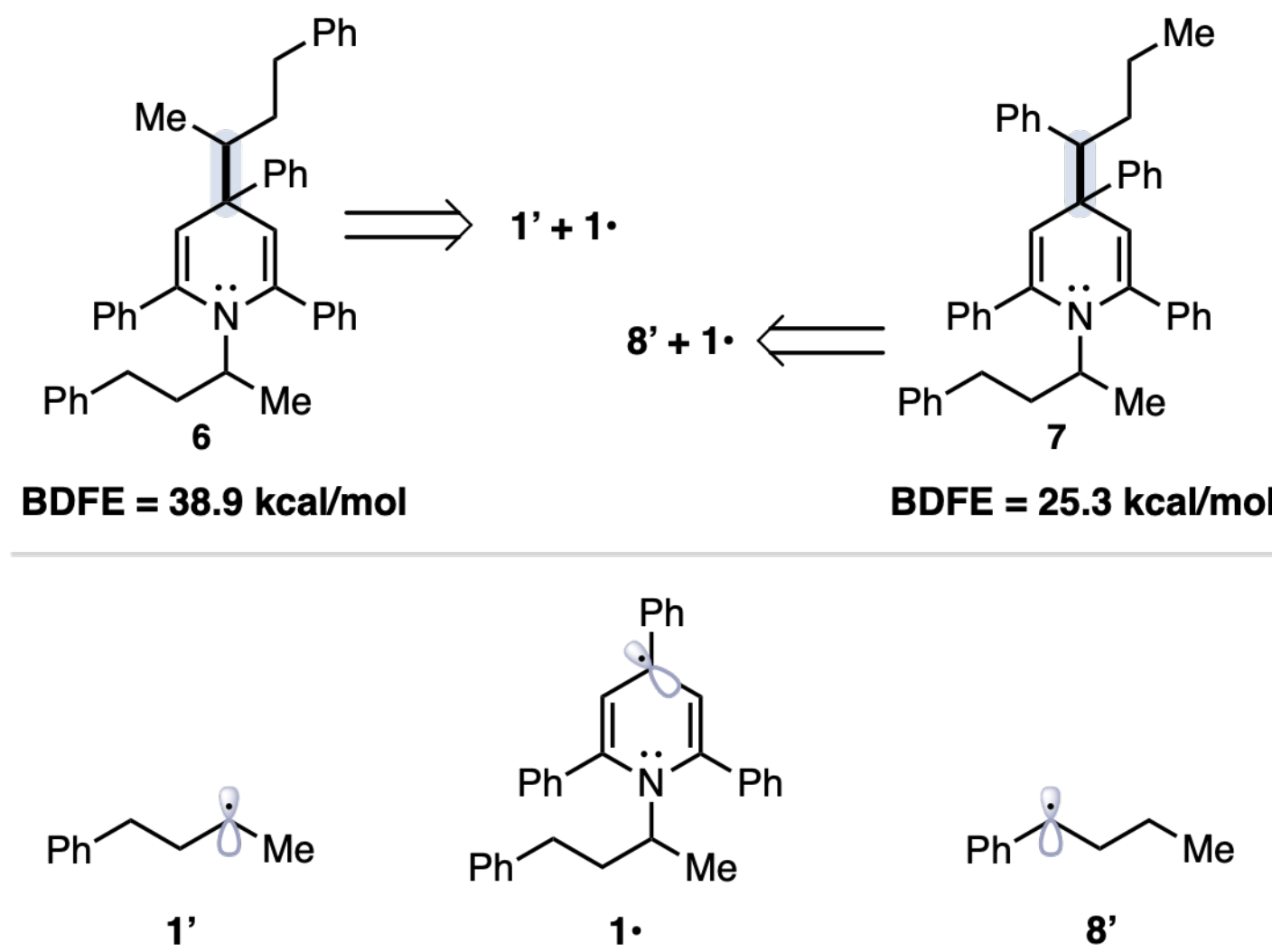

Figure S16: Calculated BDFEs of the newly formed C–C bonds. M06-L/def2-TZVP/CPCM(DMA)//M06-L/def2-SVP. Gibbs free energy in kcal/mol.

7.2 Cyclic Voltammetry

7.2.1 General Information:

Cyclic voltammetry experiments were performed on a CHI660D CH Instruments Electrochemical Workstation. Experiments were run at 100 mV/s in a 25 mL three-neck round-bottom flask equipped with three electrodes: a glassy carbon working electrode, a platinum mesh counter electrode, and an Ag^{+}/Ag pseudo-reference electrode. All measurements were referenced to the internal standard redox couple Fc^{+}/Fc. The second run was used for analysis.

7.2.2 Sample preparation:

A 10 mL volumetric flask was charged with 384 mg of tetrabutylammonium hexafluorophosphate (1 mmol; 0.1 M) and an analyte (0.01 mmol for **6** and **7**, and 0.1 mmol for **1a** were used) and dry MeCN was added to make up the volume to 10 mL.

7.2.3 Experimental setup:

The flame-dried round-bottom flask was charged with the sample (10 mL) described above. The solution was then sparged with argon for 10 minutes and a voltammogram was collected. To the flask, ferrocene (0.1 mmol) was added and another voltammogram was collected for reference. Between each measurement, the Ag^{+}/Ag and platinum mesh electrodes were rinsed, and the glassy electrode was polished on Spec-cloth.

*7.2.4 Cyclic voltammogram of 2,4,6-triphenyl-1-(4-phenylbutan-2-yl)pyridin-1-ium tetrafluoroborate **1a***:

The experiment was run starting from 0 V and in the negative direction. The second scan of the experiment was used to plot the voltammogram. The voltammogram plotted below indicated that the dihydropyridine **1a** underwent an irreversible reduction event.

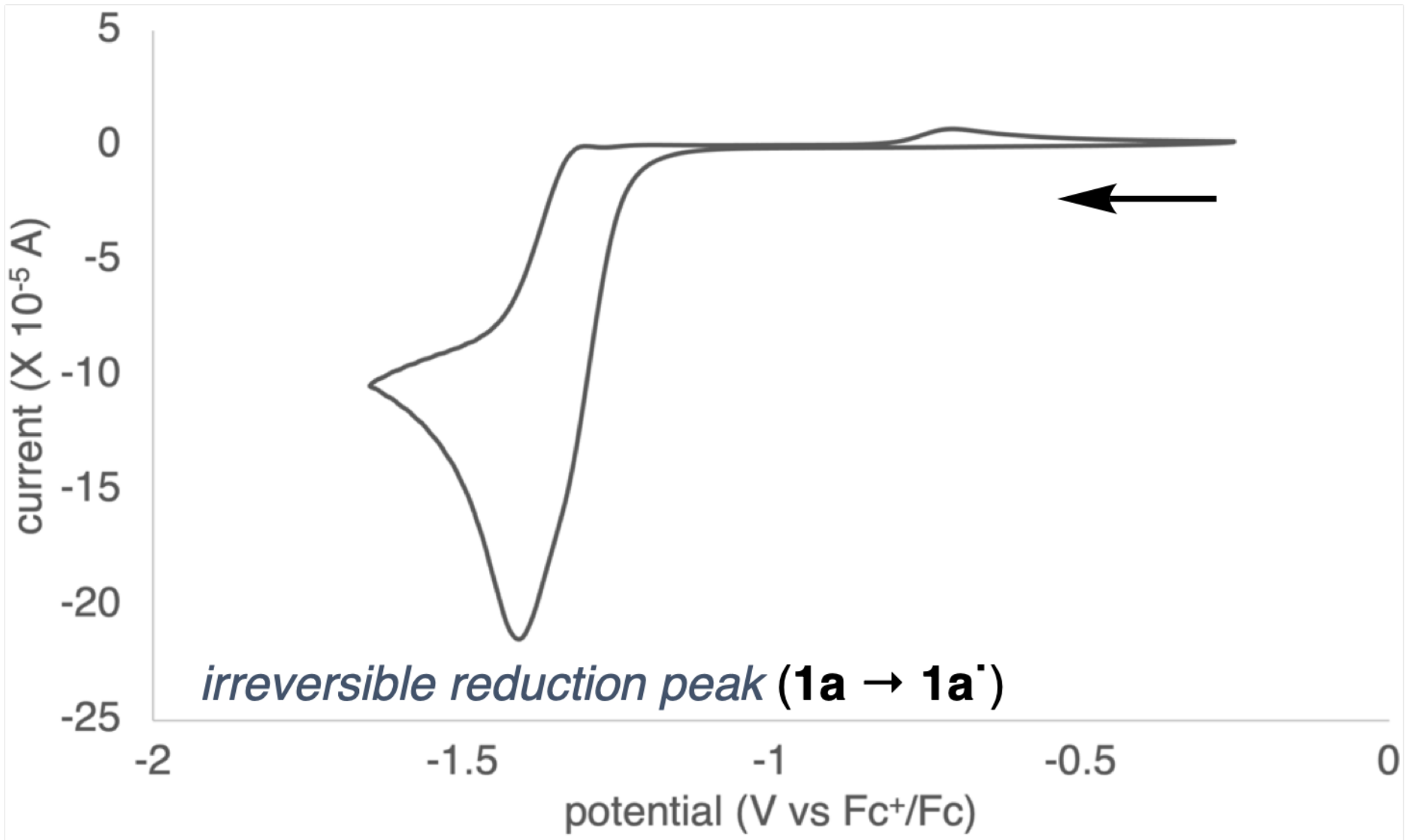

Figure S17: *Cyclic voltammogram of **1a**: $E_{p/2}$* = -1.33 V vs Fc+/Fc in MeCN.

*7.2.5 Cyclic voltammogram of 2,4,6-triphenyl-1,4-bis(4-phenylbutan-2-yl)-1,4-dihydropyridine **6***:

The experiment was run starting from 0 V and in the positive direction first followed by the negative direction. The second scan of the experiment was used to plot the voltammogram. The voltammogram plotted below indicated that the dihydropyridine **6** underwent an irreversible oxidation event followed by an irreversible reduction event. The oxidation event was attributed to a single-electron oxidation of **6** to the corresponding radical cation **[6]**$^{+\cdot}$. The reduction event matched that of Katritzky salt **1a** (see 7.2.4), indicating upon single-electron oxidation, the dihydropyridine **6** generated **1a** (as depicted below).

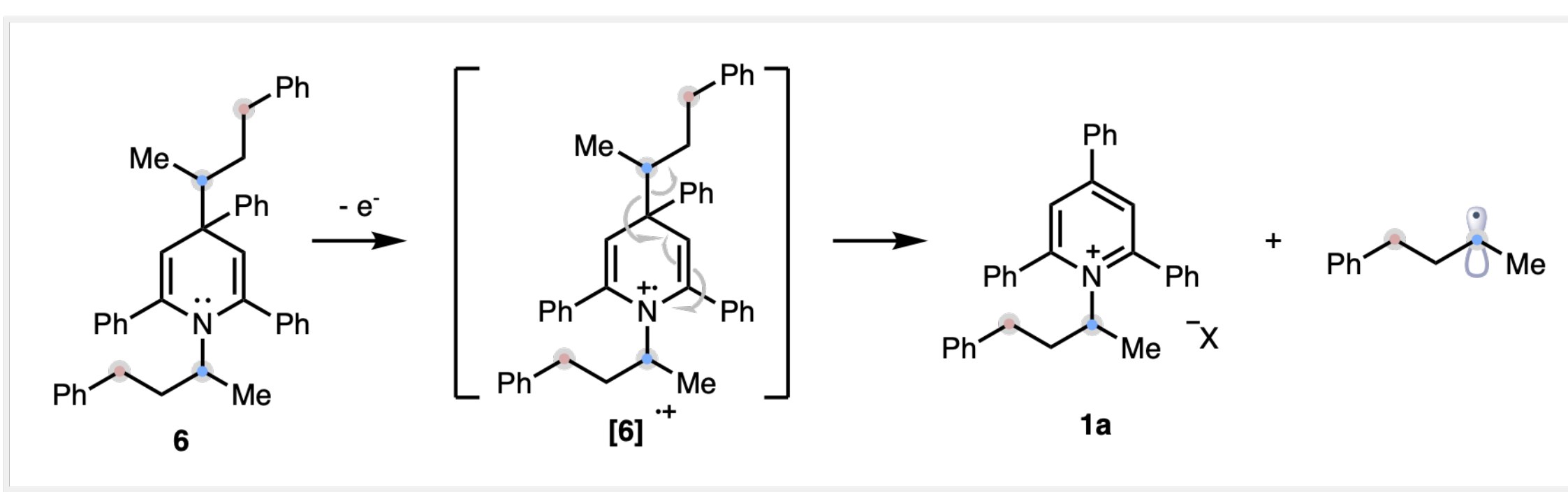

Figure S18: Schematic representation of oxidative fragmentation of dihydropyridine **6**.

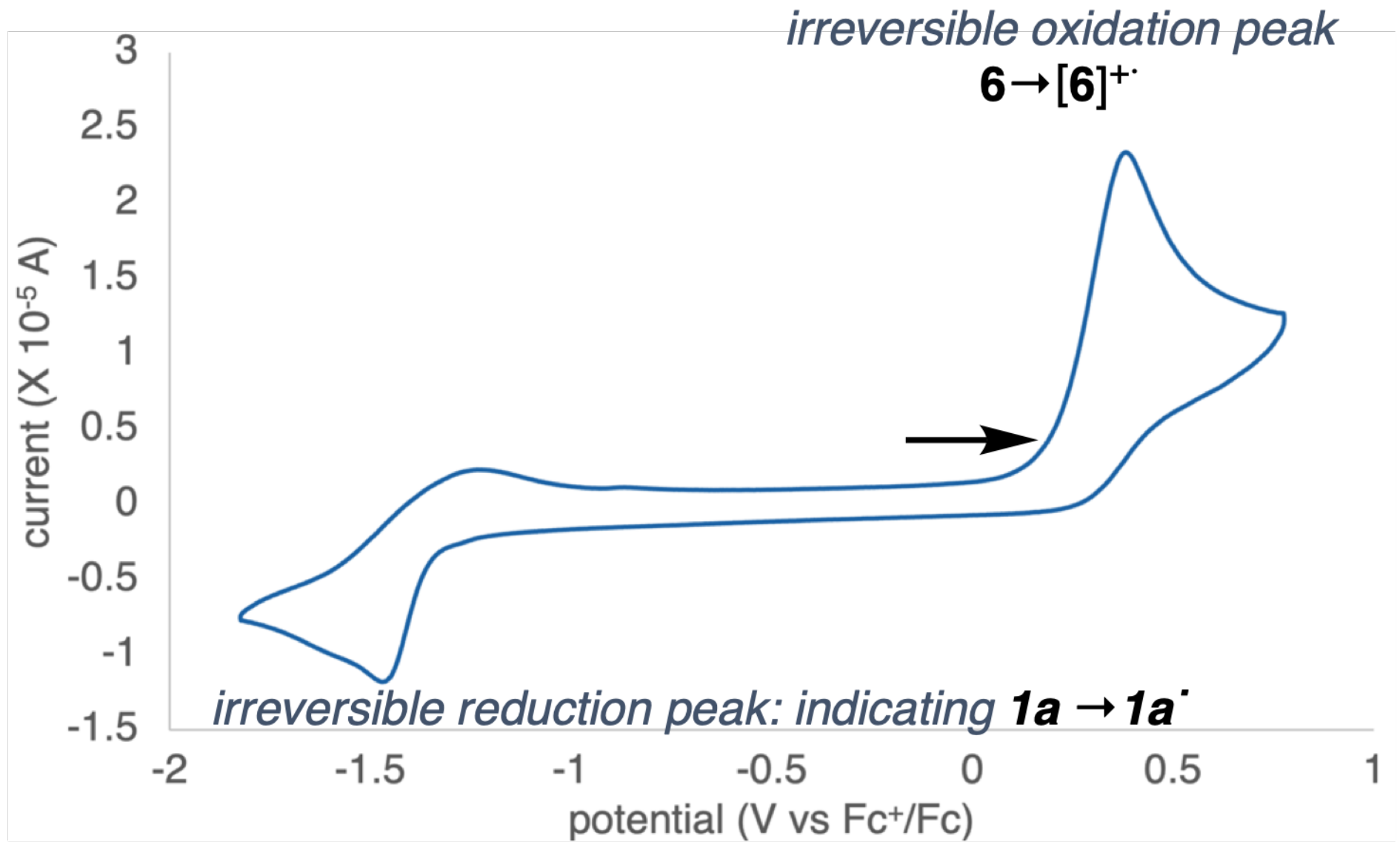

Figure S19: *Cyclic voltammogram of **6**:* $E_{p/2}$ = +0.28 V vs Fc+/Fc in MeCN.

*7.2.6 Cyclic voltammogram of 2,4,6-triphenyl-1-(4-phenylbutan-2-yl)-4-(1-phenylbutyl)-1,4-dihydropyridine **7***:

The experiment was run starting from 0 V and in the positive direction first followed by the negative direction. The second scan of the experiment was used to plot the voltammogram. The voltammogram plotted below indicated that the dihydropyridine **7** underwent an irreversible oxidation event followed by an irreversible reduction event. The oxidation event was attributed to a single-electron oxidation of **7** to the corresponding radical cation **[7]+·**. The reduction event matched with that of Katritzky salt **1a** (see 7.2.4), indicating upon single-electron oxidation, the dihydropyridine **7** generated **1a** (as depicted below).

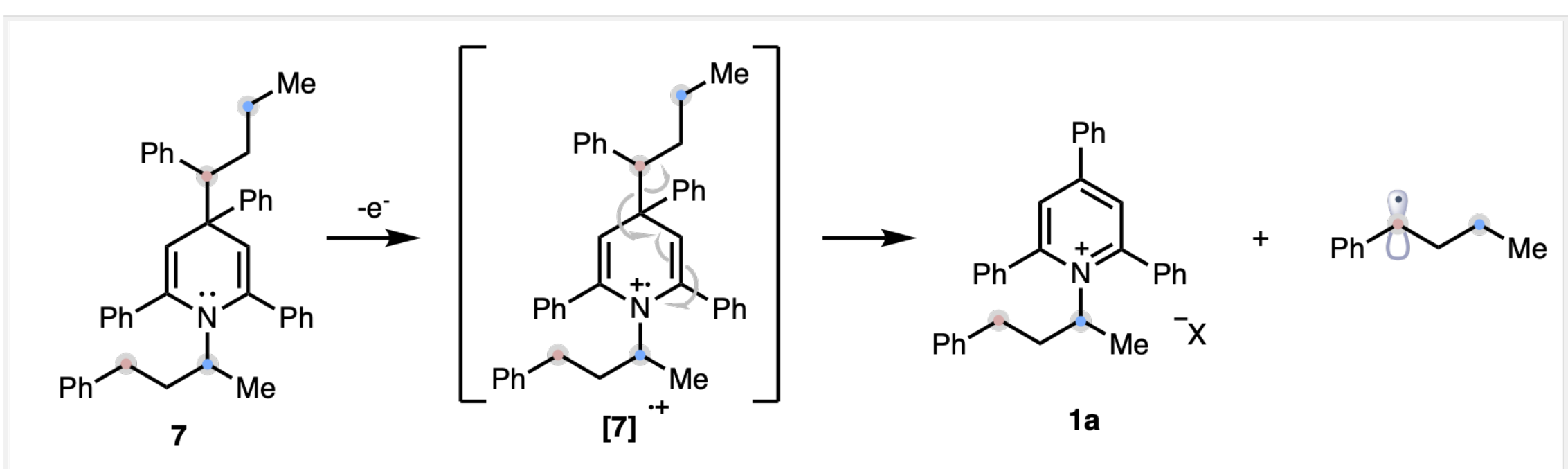

Figure S20: Schematic representation of oxidative fragmentation of dihydropyridine **7**.

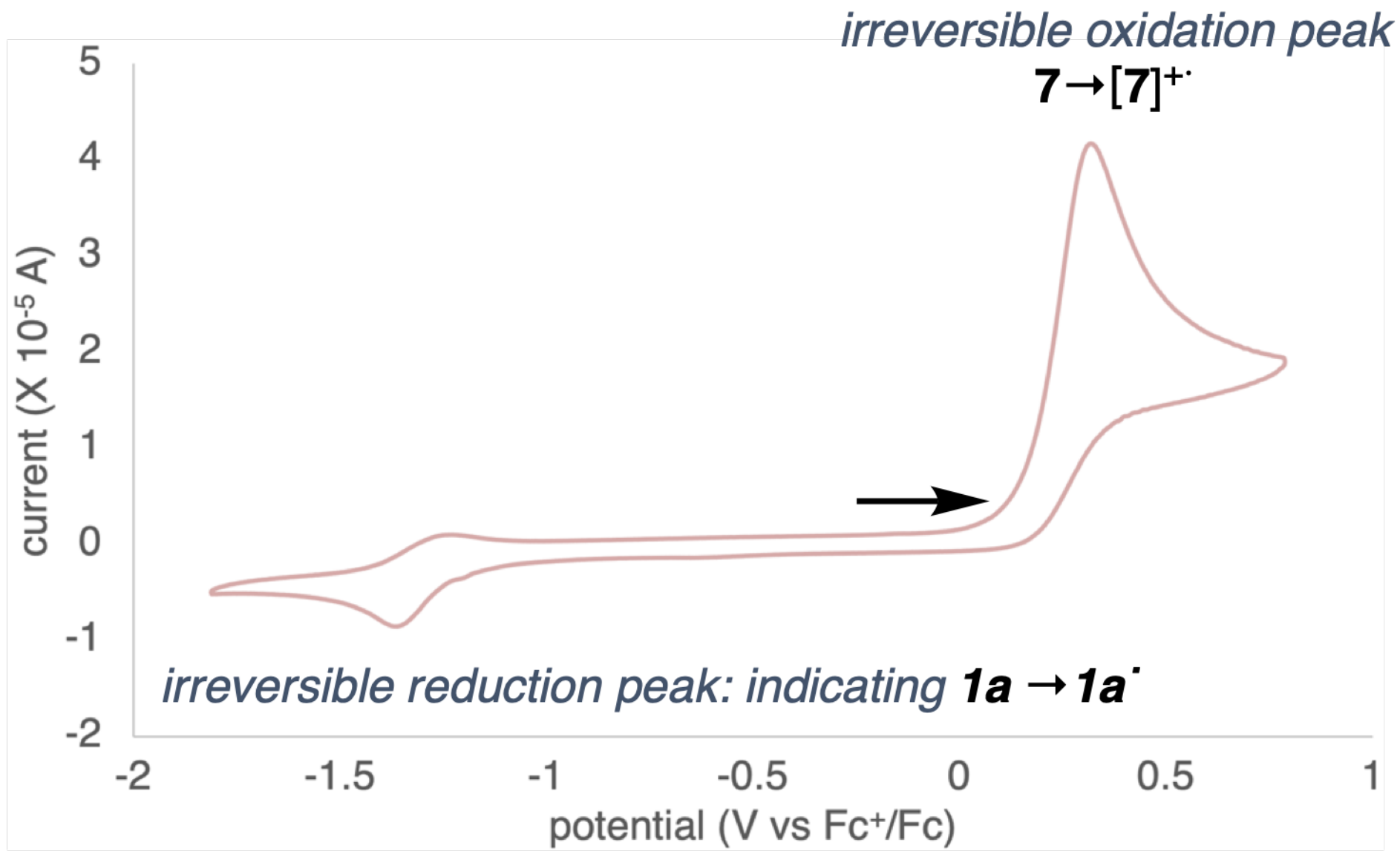

Figure S21: *Cyclic voltammogram of **7**:* $E_{p/2}$ = +0.23 V vs Fc^{+}/Fc in MeCN.

7.3 Stern-Volmer Luminescence Quenching Studies

A stock solution of $[Ir(ppy)_2(dtbbpy)]PF_6$ (0.10 mM) was prepared by dissolving 10 mg in 10 mL of anhydrous MeCN, followed by 10-fold dilution (1 mL → 10 mL). Stock solutions of quencher dihydropyridine **7** (16.1 mg in 3 mL anhydrous MeCN, 9.35 mM) were prepared in a 3 mL volumetric flask. For each luminescence measurement, a 3 mL cuvette was charged with 250 μL of the Ir stock solution and the appropriate volume of the DHP stock solution and anhydrous MeCN to give a total volume of 3.00 mL with a final $[Ir(ppy)_2(dtbbpy)]PF_6$ = 8.33 μM and dihydropyridine **7** concentrations of 0, 1, 3, and 5 mM. Steady-state emission spectra were recorded at room temperature using a fluorescence spectrometer (excitation: 420 nm, excitation slit: 3.00 nm, emission scan: 550–650 nm, emission slit: 4.50 nm, step: 2 nm, dwell time: 0.20 s). Triplicate measurements were performed at each quencher concentration. Emission intensities at λ_{em} = 588 nm were used for data analysis. The Stern-Volmer quenching constant (K_{sv}) was determined from a linear fit of I_0/I vs [**7**]: $I_0/I = 1 + K_{SV}[Q]$. The bimolecular quenching rate constant k_q was calculated using $k_q = K_{sv}/\tau_0$, where τ_0 = 557 ns as the reported excited-state lifetime of $[Ir(ppy)_2(dtbbpy)]PF_6$ in MeCN.[28]

**Entry**	**[7] = 0 mM**	**[7] = 1 mM**	**[7] = 3 mM**	**[7] = 5 mM**
Trial 1	1,507,849	1,038,219	597,430	456,163
Trial 2	1,510,807	1,035,315	594,854	455,533
Trial 3	1,499,962	1,036,806	595,257	450,259
Average	1,506,206	1,036,780	595,847	453,985
I_0/I	1.000	1.453	2.528	3.318

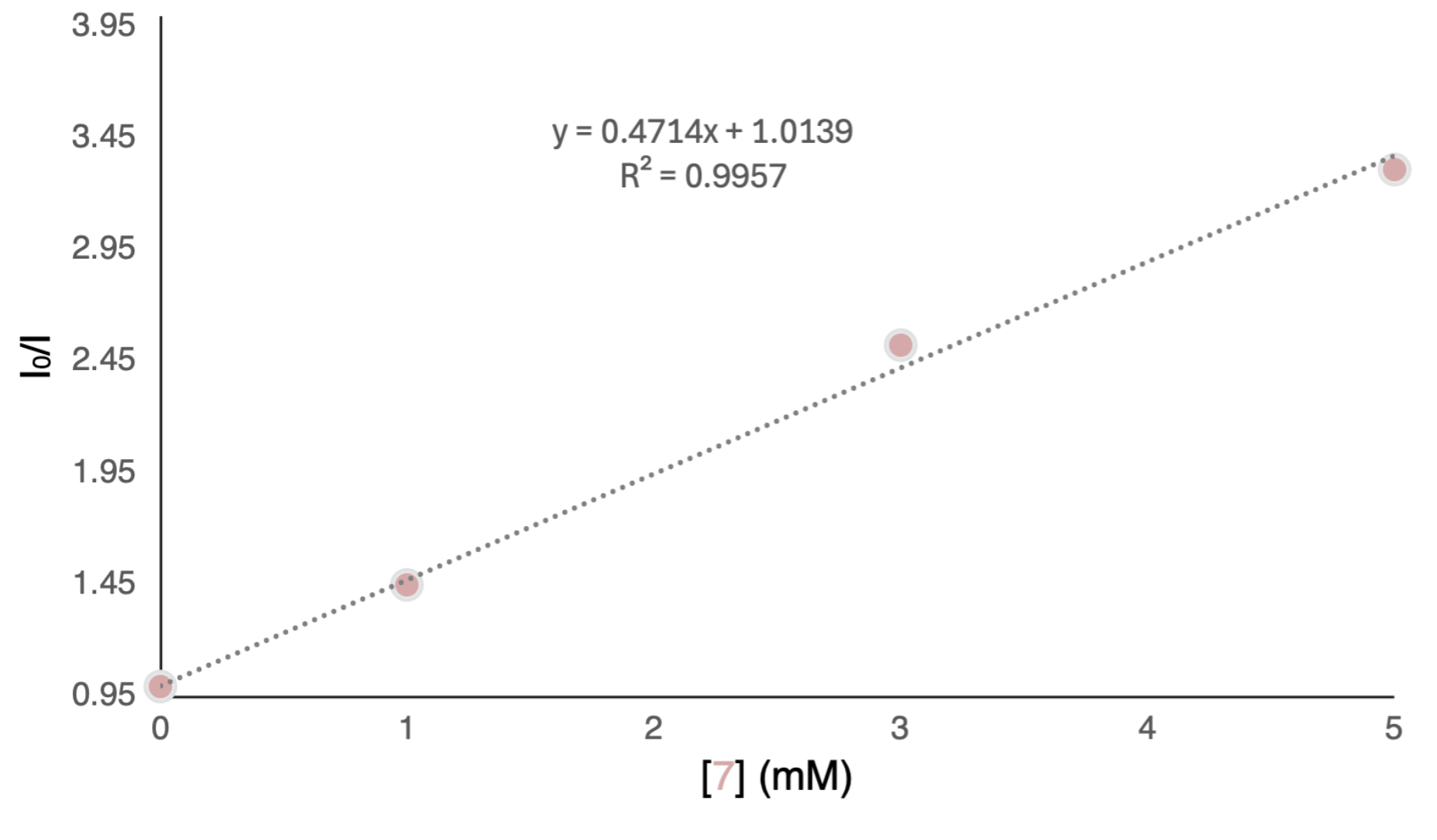

Figure S22. Stern-Volmer luminescence quenching plot of $[Ir(ppy)_2(dtbbpy)]PF_6$ by dihydropyridine **7** in anhydrous MeCN at room temperature. Emission intensity λ_{em} = 588 nm (excitation at 420 nm). K_{sv} = 471 M^{-1} (R^2 = 0.9957); $k_q = 8.46 \times 10^8$ M^{-1} s^{-1} (τ_0 = 557)

7.4 Dihydropyridine Formation

A) Dihydropyridine formation: in the absence of Ni/BC

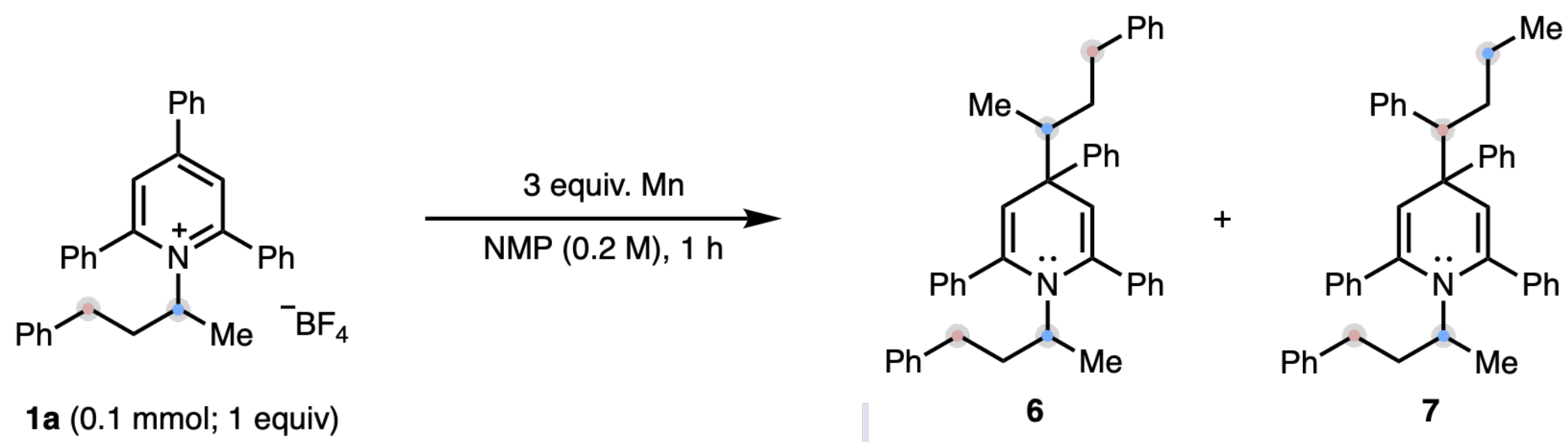

^{1}H NMR yield: [a]Based on conv. of **1a**	6	7
	40% (80%)[a]	0%

B) Dihydropyridine formation: in the presence of Ni/BC

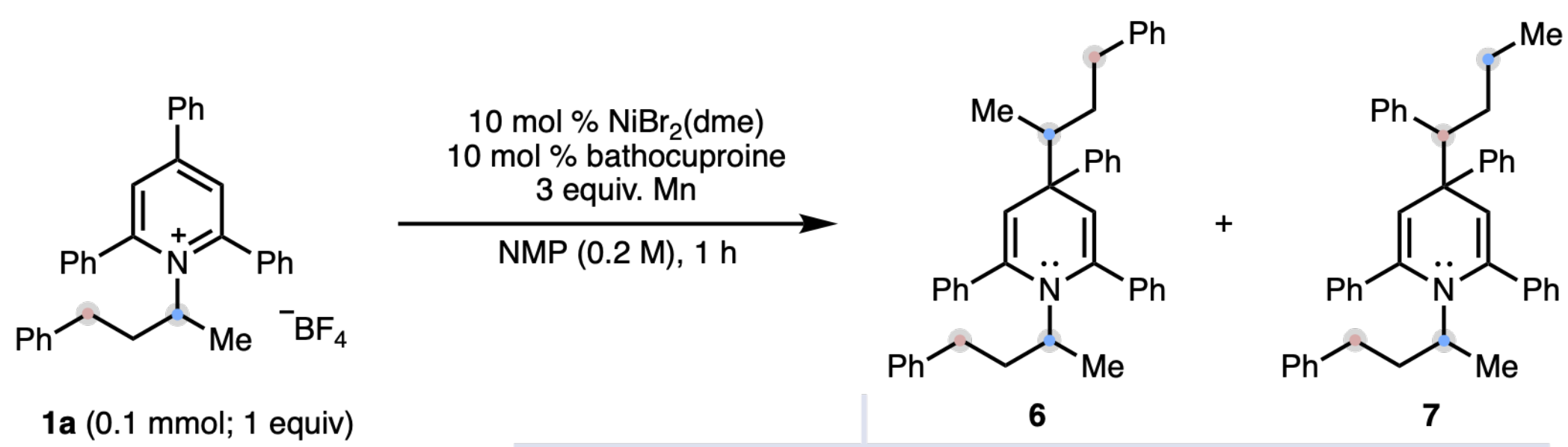

^{1}H NMR yield: [a]Based on conv. of **1a**	6	7
	1% (2%)[a]	43% (86%)[a]

C) Dihydropyridine formation: in the presence of Ni/6,6′-Me_2bpy

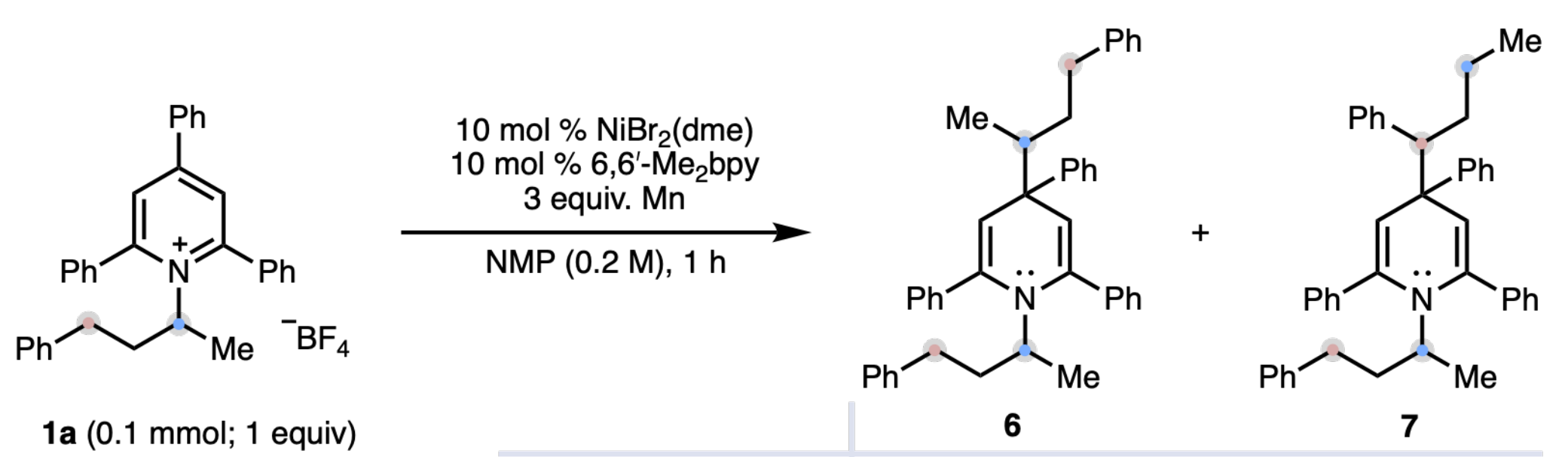

^{1}H NMR yield: [a]Based on conv. of **1a**	6	7
	2% (4%)[a]	44% (88%)[a]

Figure S23: Dihydropyridine formation in the presence and the absence of the nickel precatalyst.

Reaction setup:

Inside a nitrogen-filled glovebox, a 1-dram vial equipped with a PTFE-coated magnetic stir bar was charged with 2,4,6-triphenyl-1-(4-phenylbutan-2-yl)pyridin-1-ium

tetrafluoroborate (52.7 mg, 0.100 mmol, 1.00 equiv) and manganese powder (16.5 mg, 0.300 mmol, 3.00 equiv). The vial was then hermetically sealed with a syringeable cap (PP/PTFE/silicone liner septum) and removed from the glovebox. Under a positive nitrogen pressure, the reaction vial was charged with by 0.500 mL of the precatalyst solution (described in 3.1.1 for Ni/BC and 6.1.2.1 for Ni/6,6′-Me_2bpy). The vial was then sealed with M grease, secured with electrical tape, covered with aluminum foil, and stirred at 1500 RPM at room temperature for 1 h.

Note: For reaction run in the absence of the nickel catalyst, after the addition of **1a** and Mn, 0.500 mL NMP was added instead of the precatalyst solution.

After completion of the reaction, 1.00 mL of a 1 M solution of trimethoxybenzene in ethyl acetate was added to the reaction mixture, diluted with ~0.5 mL of 10% aqueous LiCl solution, gently shaken, and then allowed to settle. The aqueous layer was decanted, and the organic layer was washed with ~1 mL of brine, shaken, and allowed to settle. After decanting the aqueous layer, the organic phase was dried over Na_2SO_4, filtered through a short pad of Celite® topped with Na_2SO_4, concentrated under reduced pressure, and analyzed by ^1H NMR spectroscopy.

7.5 Reduction of Katritzky salt 1a by a nickel(0) complex of bathocuproine

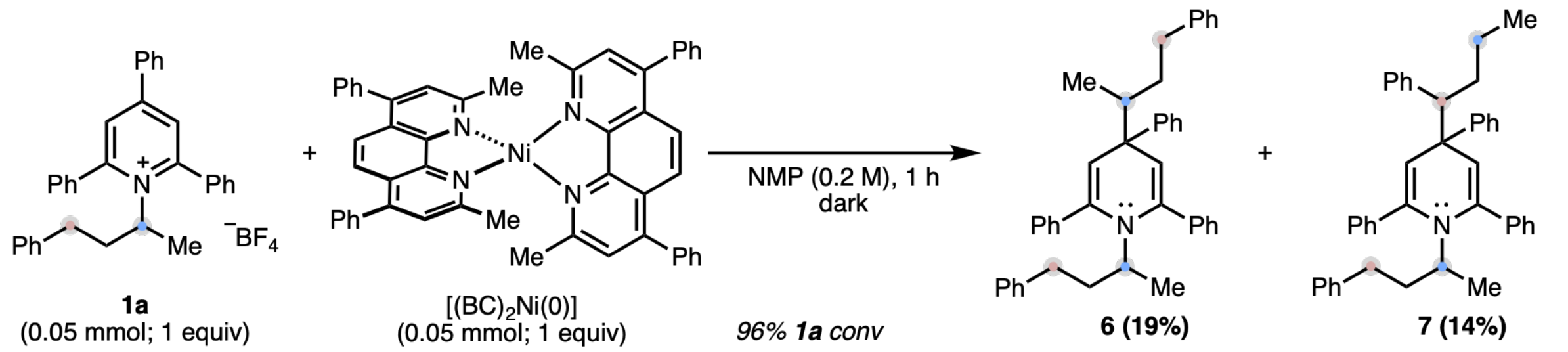

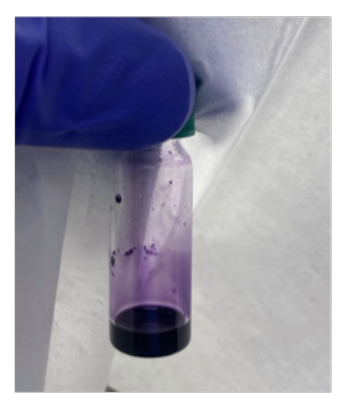

Solution of [(BC)$_2$Ni(0)]
in NMP (t = 0 min)

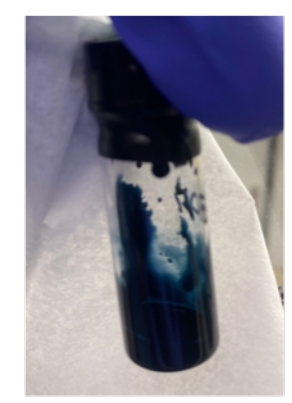

Reaction mixture
After addition of 1a to [(BC)$_2$Ni(0)]
(t = 2 min)

Figure S24: Schematic representation of reduction of Katritzky salt **1a** by (BC)$_2$nickel(0).

Experimental setup:

To a 1-dram vial, [(BC)$_2$Ni(0)] (39.0 mg; 0.05 mmol; 1.00 equiv) was added followed by 0.500 mL NMP. To the resulting dark purple solution, 2,4,6-triphenyl-1-(4-phenylbutan-2-yl)pyridin-1-ium tetrafluoroborate **1a** (26.3 mg; 0.05 mmol; 1.00 equiv) was added. The reaction vial was covered with aluminum foil and stirred at room temperature. Upon couple of minutes of stirring, the initial dark purple color solution changed immediately to dark indigo blue, which persisted for 1 h. After 1 h, the reaction was opened to air, diluted with ethyl acetate and 10% LiCl (aq.) solution, shaken gently, and allowed to settle. The aqueous layer was decanted. The ethyl acetate solution was then washed with brine, passed through a short Celite® plug, dried over Na_2SO_4, and filtered. To this solution, 0.05 mmol of 1,3,5-trimethoxybenzene was added. The solution was then concentrated and analyzed by ^1H NMR spectroscopy.

Result:

The crude reaction mixture showed 96% consumption of **1a**, of which 33% converted to the dihydropyridines **6** and **7**, and 3% alkenes. The color of the reaction mixture after

reaction was blue, which indicated a nickel(I) complex of bathocuproine ligand as reported by Martin and coworkers.[29]

Conclusions:

These results support the hypothesis that a nickel(0) complex of bathocuproine can reduce the Katritzky salt.

7.6 Time-Course Studies

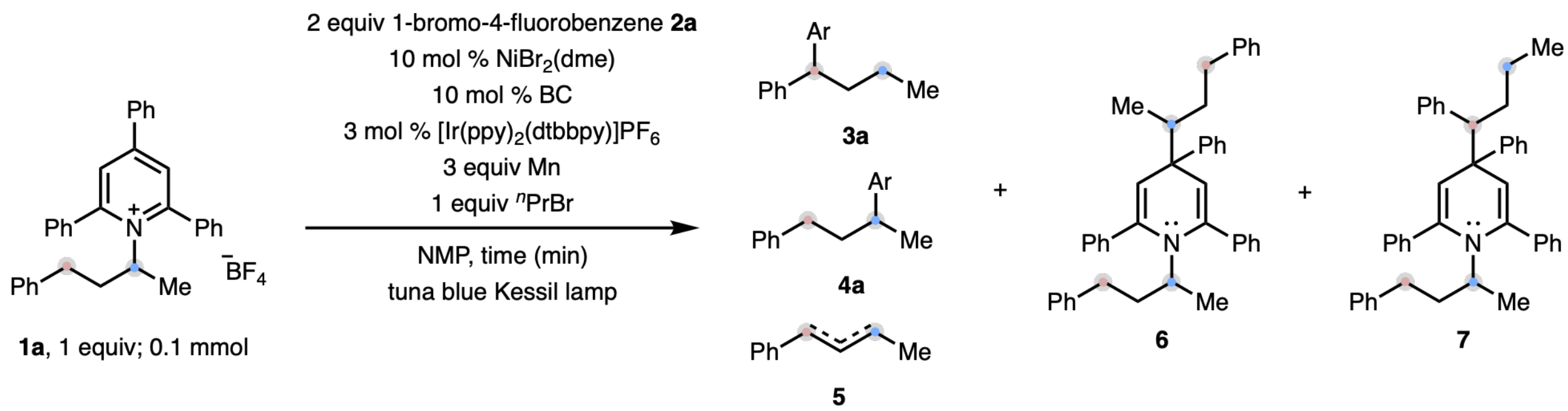

time (min)	1a	3a	4a	7	6	7+6	5
0	100	0	0	0	0	0	0
5	72	0	0	5	2	14	8
15	60	4	<1	16	2	18	10
30	14	18	<1	24	2	26	16
45	9	38	<1	24	4	28	21
120	0	64	1	2	<1	2	16
180	0	73	1	0	0	0	14
240	0	84	2	0	0	0	0

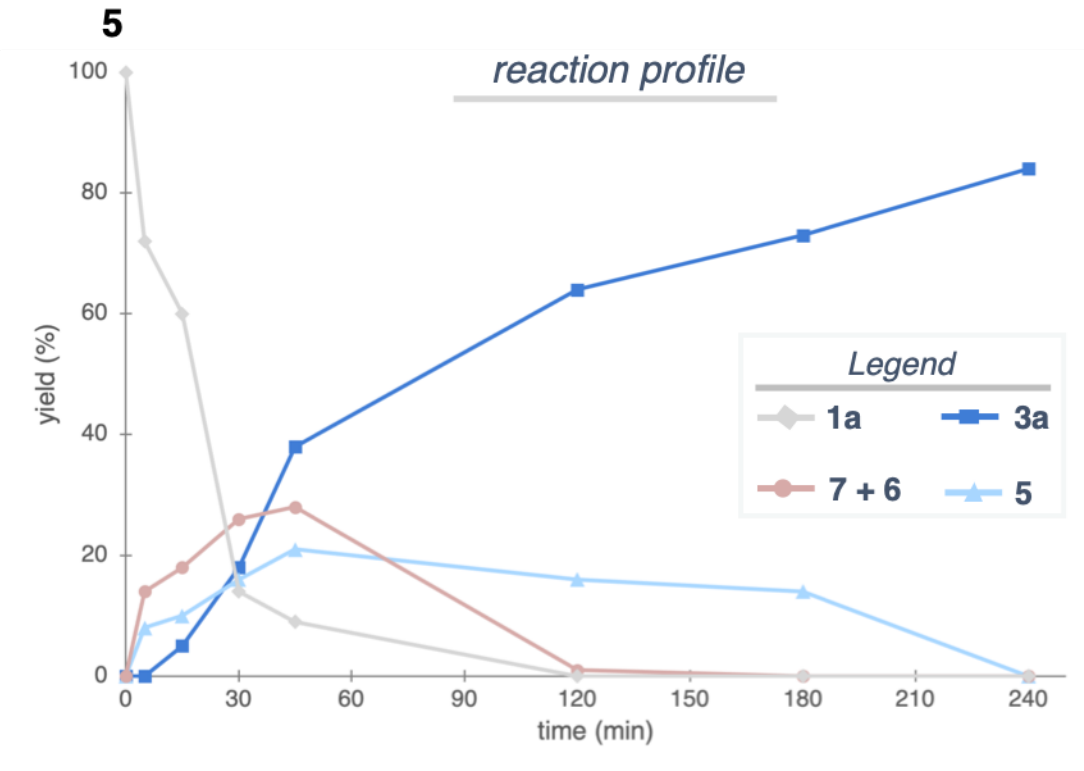

Figure S25: Time-course studies on the model reaction.

Note: These reactions were conducted according to General Procedure A described in Reaction Optimization and Control Studies in section 3. Each entry in the table represents a separate reaction and the time at which the corresponding reaction was stopped. The yield was calculated by ^{1}H or ^{19}F NMR analysis of the crude reaction mixture as described in General Procedure A.

Conclusion:

The time-course studies with **1a** revealed that at early time points there was an accumulation of the dihydropyridine **7** under the model reaction conditions, supporting the storage of the translocated alkyl radical. Furthermore, these experiments also demonstrated that under the model reaction conditions, the mixture of alkenes 5 was funneled into the product during the reaction.

7.7 Reactivity of dihydropyridines

7.7.1 Reactivity of dihydropyridines under the model reaction conditions:

A) Reaction of **6** with 1-bromo-4-fluorobenzene **2a**

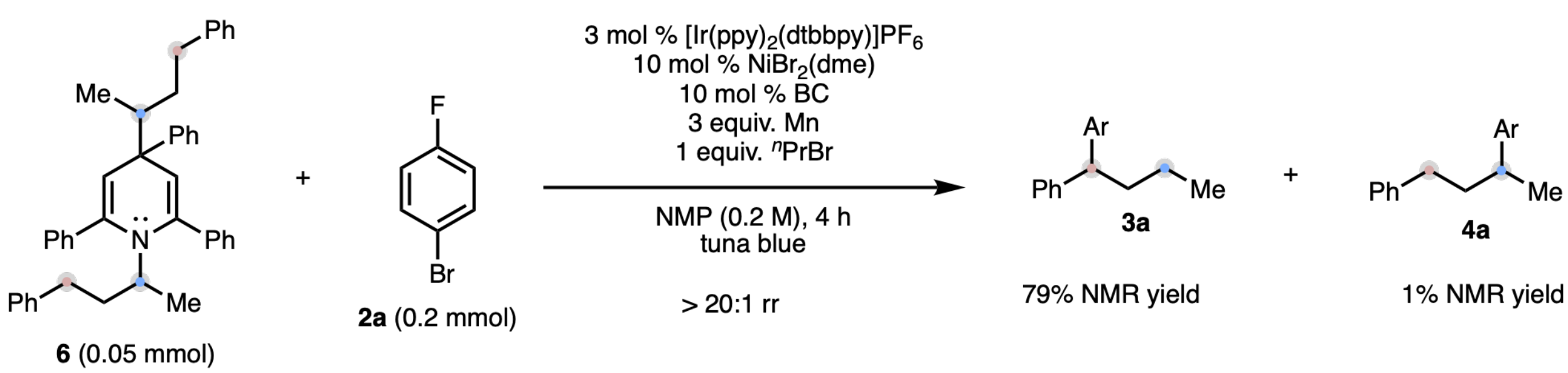

B) Reaction of **7** with 1-bromo-4-fluorobenzene **2a**

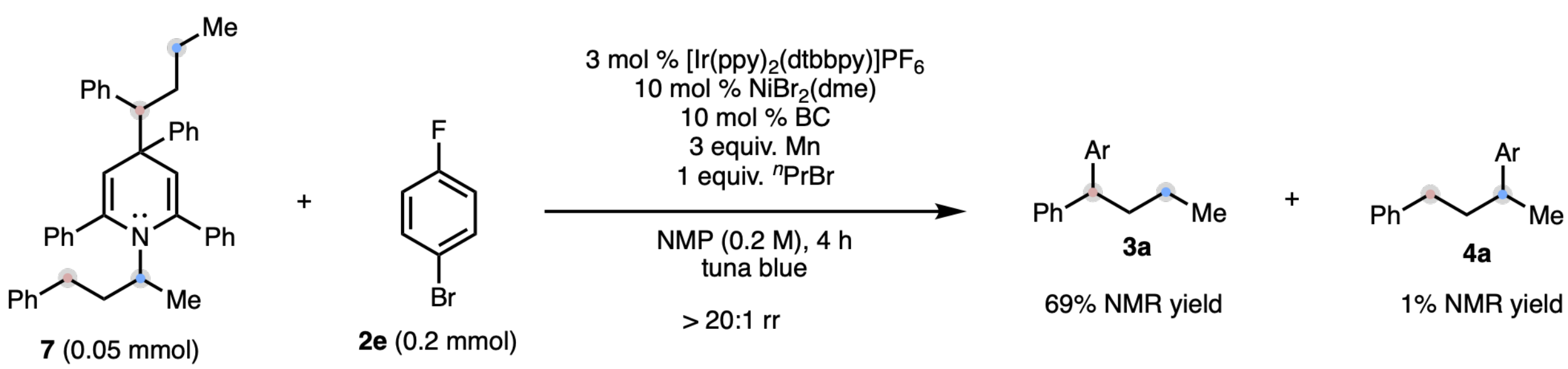

Figure S26: Reactivity of dihydropyridines **6** and **7** under model reaction conditions.

Note: These reactions were conducted according to General Procedure A described in Reaction Optimization and Control Studies in section 3. In place of 0.1 mmol of **1a**, 0.05 mmol of **6** or **7** were used, which is equivalent to 0.1 mmol of the alkyl fragment. The NMR yields are calculated based on 0.1 mmol of the alkyl fragment.

Conclusion:

The results of the reactions above indicate that the dihydropyridines are competent substrates under model reaction conditions. The high selectivity of the reaction of **6** with **2a** can be explained by the conversion of **6** to **7** prior to the release of the translocated alkyl radical for the coupling reaction (see section 7.7 below).

7.7.2 Reactivity of **6** in the presence of nickel precatalyst:

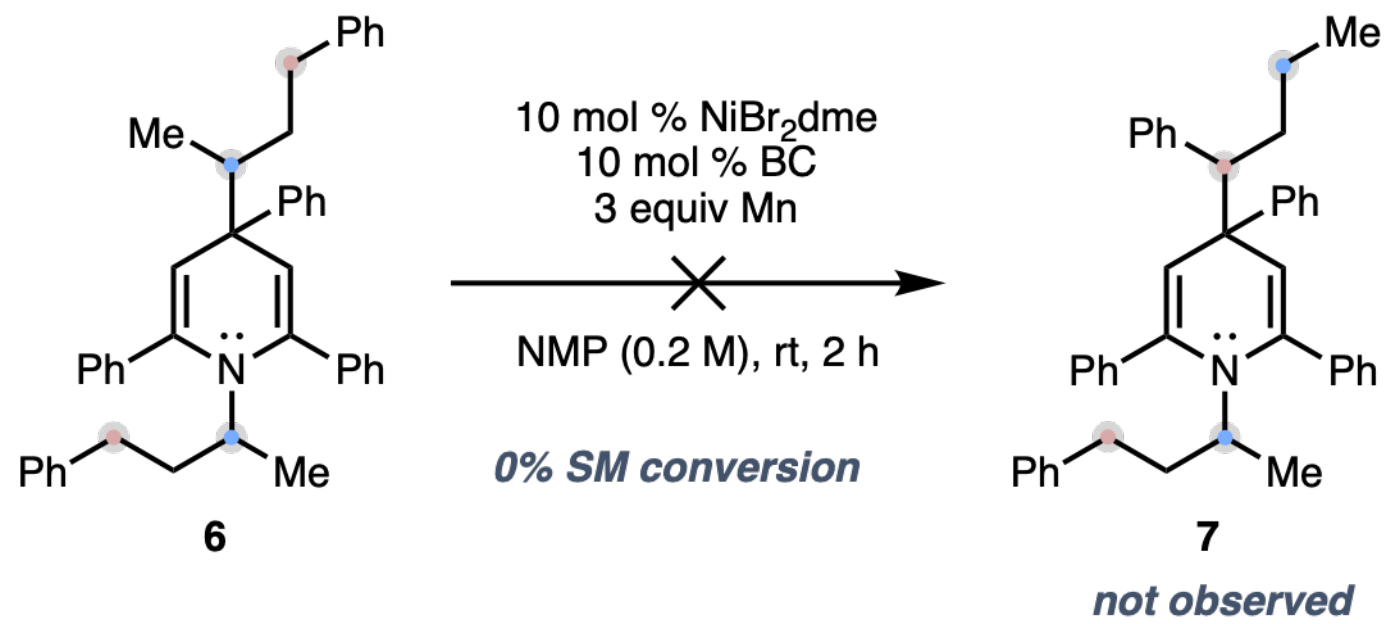

Figure S27: Reactivity of dihydropyridines **6** under reductive conditions.

Experimental setup:

To a 1-dram vial, 2,4,6-triphenyl-1,4-bis(4-phenylbutan-2-yl)-1,4-dihydropyridine **6** (29 mg; 0.05 mmol; 1.0 equiv) and Mn (8.3 mg, 0.15 mmol, 3.0 equiv) were added. The vial was then charged with 0.25 mL of the precatalyst solution (described in 3.1.1 for Ni/BC, accounting for 10 mol % Ni/BC). The resulting mixture was stirred for 2 h. The reaction was then opened to air, diluted with 0.50 mL of a 1 M solution of trimethoxybenzene in ethyl acetate ethyl acetate, followed by 2.0 mL ethyl acetate and 10% LiCl (aq.) solution, shaken gently, and allowed to settle. The aqueous layer was decanted. The ethyl acetate solution was then washed with brine, passed through a short celite plug, dried over Na_2SO_4, and filtered. The solution was then concentrated and analyzed by ^1H NMR spectroscopy.

Result:

No conversion of 2,4,6-triphenyl-1,4-bis(4-phenylbutan-2-yl)-1,4-dihydropyridine **6** was observed.

Conclusions:

Dihydropyridine **6** is persistent under reductive conditions and in the presence of nickel(0) complex of bathocuproine. Therefore, **6** does not lead to the formation of **7** under such (reductive) conditions.

7.7.3 Persistency of the dihydropyridines **6** and **7** when irradiated with tuna blue Kessil lamps:

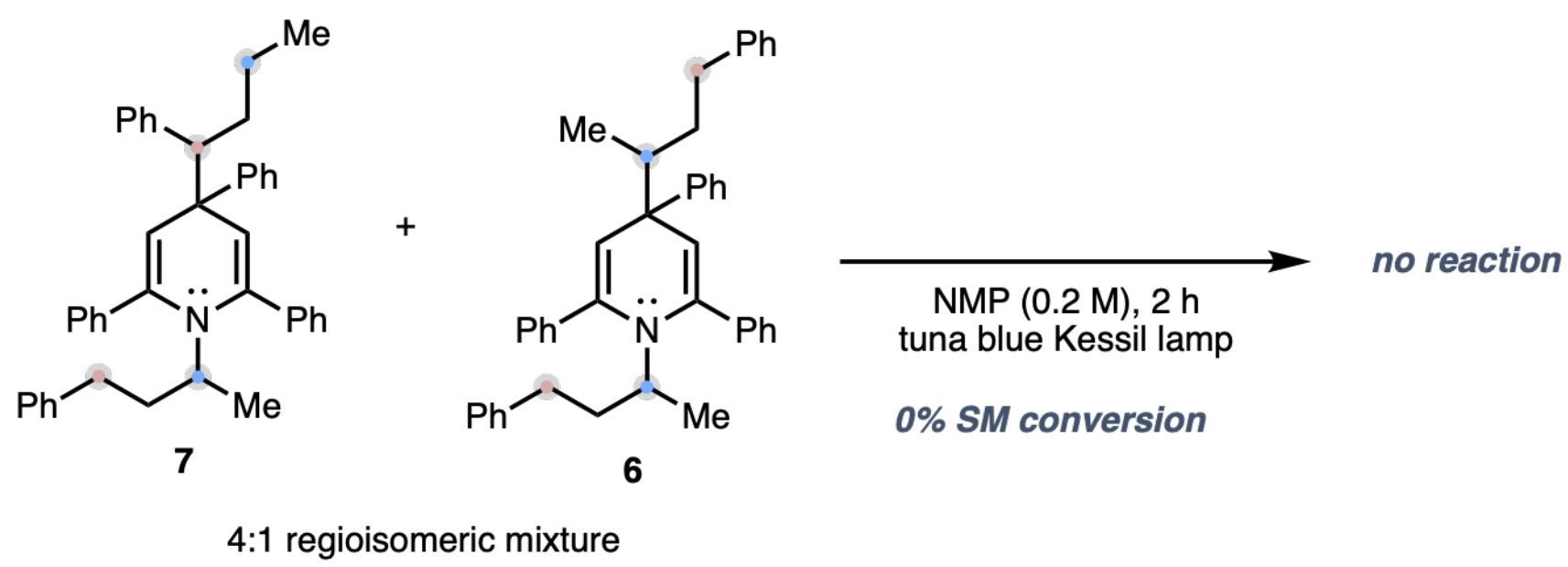

Figure S28: Persistency of dihydropyridines **6** and **7**.

Experimental setup:

To a 1-dram vial, a 4:1 regioisomeric mixture of 2,4,6-triphenyl-1-(4-phenylbutan-2-yl)-4-(1-phenylbutyl)-1,4-dihydropyridine **7** and 2,4,6-triphenyl-1,4-bis(4-phenylbutan-2-yl)-1,4-dihydropyridine **6** (29 mg; 0.05 mmol; 1.0 equiv) was added followed by 0.25 mL NMP. The resulting mixture was stirred and irradiated with tuna blue Kessil lamps for 2 h. The reaction was then opened to air, diluted with 0.50 mL of a 1 M solution of trimethoxybenzene in ethyl acetate ethyl acetate, followed by 2.0 mL ethyl acetate and 10% LiCl (aq.) solution, shaken gently, and allowed to settle. The aqueous layer was decanted. The ethyl acetate solution was then washed with brine, passed through a short Celite® plug, dried over Na_2SO_4, and filtered. The solution was then concentrated and analyzed by ^1H NMR spectroscopy.

Result:

No conversion of 2,4,6-triphenyl-1-(4-phenylbutan-2-yl)-4-(1-phenylbutyl)-1,4-dihydropyridine **7** and 2,4,6-triphenyl-1,4-bis(4-phenylbutan-2-yl)-1,4-dihydropyridine **6** was observed. The regiosiomeric ratio remained unchanged.

Conclusions:

Dihydropyridines **7** and **6** are persistent when irradiated with tuna blue Kessil lamps.

7.8 Time-course study of the reaction of dihydropyridine 6 under model reaction conditions

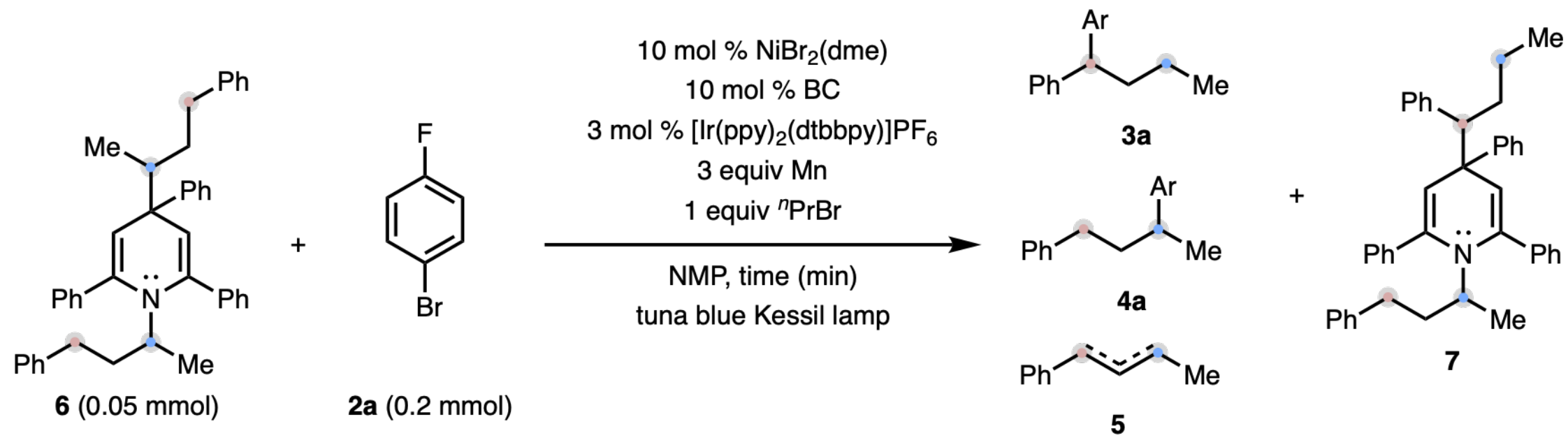

time (min)	6	3a	4a	7	5
0	100	0	0	0	0
5	56	1	0	27	10
15	23	8	0	39	17
30	10	15	<1	34	15
60	7	29	1	14	21
120	0	69	1	0	3
180	0	75	1	0	0

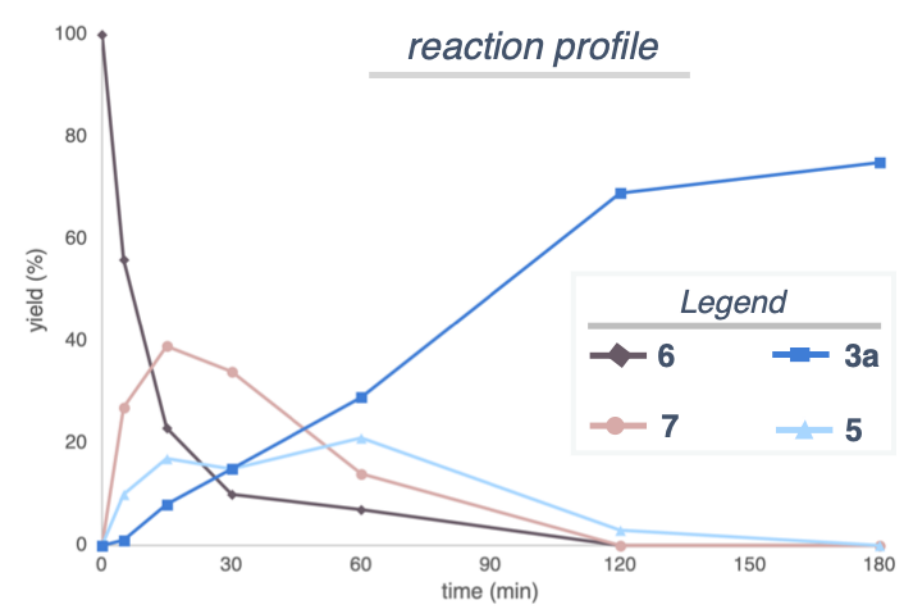

Figure S29: Time-course studies of the reaction with dihydropyridines **6** and **2a**.

Note: These reactions were conducted according to General Procedure A described in Reaction Optimization and Control Studies in section 3, except in place of **1a**, **6** was used as a limiting reagent. Each entry in the table represents a separate reaction and the time at which the corresponding reaction was stopped. The yield was calculated by ^{1}H or ^{19}F NMR analysis of the crude reaction mixture as described in General Procedure A.

Conclusion:

These time-course studies demonstrated that under the model reaction conditions, the dihydropyridine **6** was first converted to dihydropyridine **7**. These results support the high regioselectivity observed when **6** was used in place of **1a** in the model reaction (see section 7.6.1).

7.9 Other pathways considered

7.9.1 Reduction of Ni(II)-alkyl species:

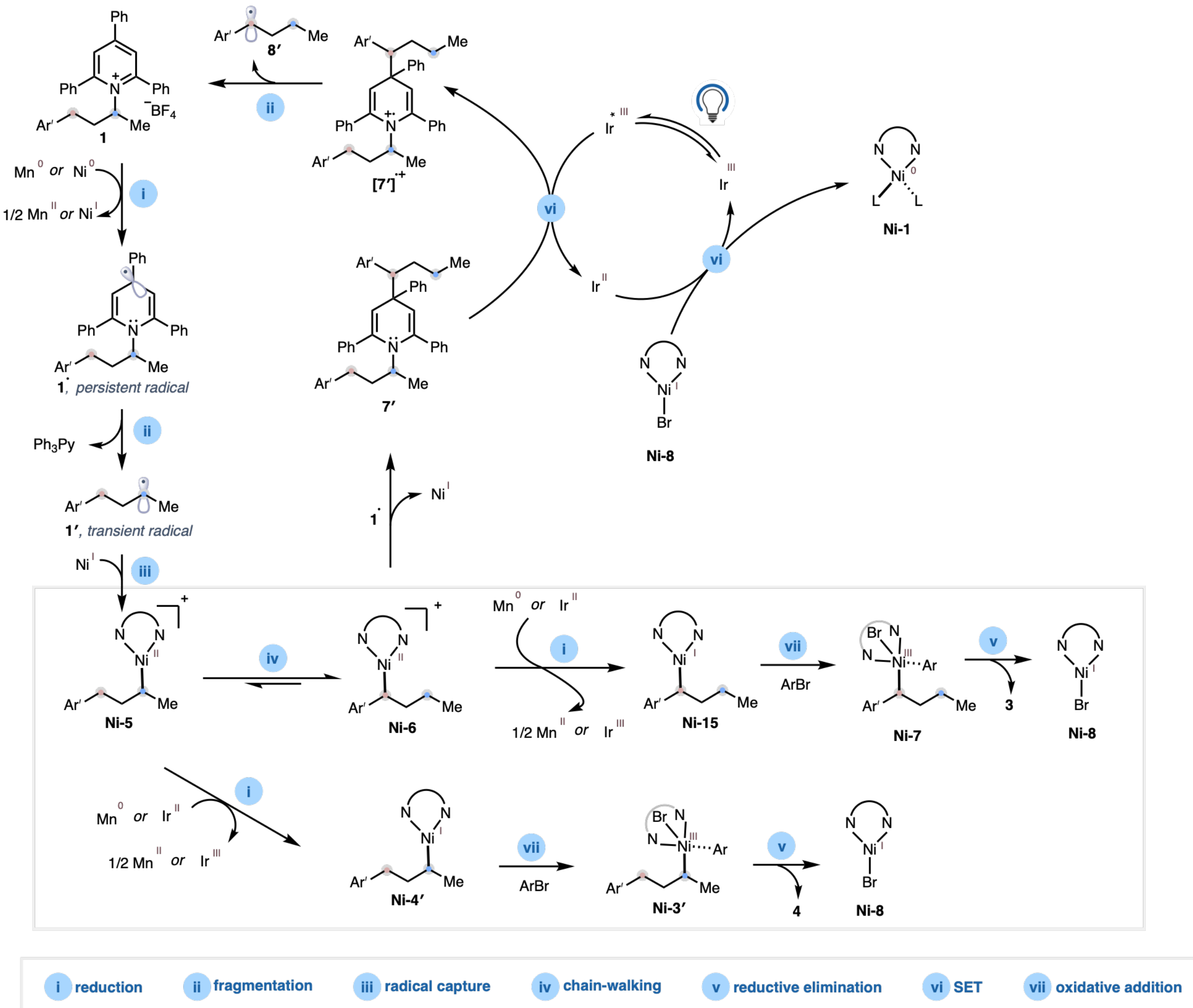

Figure S30: Proposed mechanism for the reduction of Ni(II)-alkyl species**.**

In this pathway, we considered the reduction of the Ni(II)-alkyl intermediate **Ni-5** by either Mn(0) or Ir(II). The reduction of **Ni-5** would generate a nickel(I) alkyl intermediate **Ni-4′**, which was established using DFT studies to be unlikely to lead to the observed migratory arylation (see Figure 4B). The oxidative addition of aryl bromide into **Ni-4′** followed by reductive elimination would generate the *ipso* product instead. Alternatively, **Ni-15** could be formed by reduction of the nickel(II) benzylic intermediate, **Ni-6**. Similar reduction potentials are anticipated for the regioisomeric nickel alkyl intermediates (**Ni-5 & Ni-6**).

The oxidative addition of aryl bromide into **Ni-15** followed by reductive elimination would then generate the migratory product. *In this pathway, the regioselectivity determining steps would be the reduction or the chain-walking step from **Ni-5**. However, neither of these steps would account for the different regioselectivities observed with electronically differentiated aryl bromides, such as 1-bromo-4-(trifluoromethyl)benzene **2e (**29% NMR yield; 3:1 rr under model reaction conditions) and 1-bromo-4-methoxybenzene **2c** (86% isolated yield; >20:1 rr under model reaction conditions)*.

8. Library Synthesis

General Procedure for 48-Compound Library Experiment:

Library experiments were carried out in a nitrogen-filled glovebox. Stock solutions of Ni catalysts (0.02 M in NMP) were prepared by adding nickel(II) bromide dimethoxyethane adduct (48 μmol, 10 mol %) and ligand (48 μmol, 10 mol %) to a 4 mL (1-dram) vial. Then, NMP (2398 μL) was added. The mixtures were allowed to stir for 15 min with shaking (1000 RPM) on an Eppendorf ThermoMixer at room temperature. Stock solutions of Katritzky salts (0.6 M in NMP) were prepared by adding the appropriate Katritzky salt (240 μmol, 1 equiv) to a 4 mL (1-dram) vial. Then, NMP (360 μL) was added and the mixtures were allowed to shake (1000 RPM) on an Eppendorf ThermoMixer for 15 min at room temperature. Stock solutions of (hetero)aryl bromides (0.6 M in NMP) were prepared by adding the appropriate aryl bromide (320 μmol, 2 equiv) to a 4 mL (1-dram) vial. Then, NMP (240 μL) was added followed by 1-bromopropane (160 μmol, 1 equiv) and the mixtures were allowed to shake (1000 RPM) on an Eppendorf ThermoMixer for 15 min at room temperature. A stock solution of $[Ir(dtbbpy)(ppy)_2]PF_6$ (28.8 μmol, 3 mol %) was prepared in NMP (1440 μL). To a Para-dox 96-well plate (Analytical Sales & Services, Cat No. 96973) containing 1 mL vials (borosilicate glass, 8 x 30 mm OD) with stir bars (V&P Scientific Inc., VP 711D-1, Stir Element, Dowels, Stainless Steel, Parylene Coated, 1.98 mm diameter, 4.80 mm long) was added manganese (powder, 99+% purity, 160-200 μmol, 8-10 equiv), 30 μL of the Katritzky salt stock solutions (20 μmol, 1 equiv), and 100 μL of the Ni catalyst stock solutions (2 μmol, 10 mol %) to the appropriate wells. The plate was then sealed under N_2 with a sheet of perfluoroalkoxy (PFA) film, two rubber mats and a metal lid, and allowed to shake (1000 RPM) on an Eppendorf ThermoMixer for 1 h at room temperature. After Katritzky salt activation, 30 μL of the $[Ir(dtbbpy)(ppy)_2]PF_6$ stock solution (0.6 μmol, 3 mol %) was added to all wells followed by 30 μL of the (hetero)aryl bromide/1-bromopropane stock solutions (40 μmol, 2 equiv) to the appropriate wells. The plate was then re-sealed under N_2 with a sheet of perfluoroalkoxy (PFA) film, two rubber mats and a metal lid, and the contents were stirred (500 RPM) on a tumble stirrer (V&P Scientific, Cat No. VP 710P5X). The reactions were

irradiated with a 420 nm LED array (Analytical Sales & Services, Lumidox® II 96-Well LED Array, 420-Violet, Diffuse Mat/Solid Base, Cat No. LUM296DS420) controlled by Lumidox® II Controller (Cat No. LUM2CON) for 4 h. The entire assembly was cooled with a fan and the maximum temperature of the 96-well plate was measured to be 49.8 °C with a thermocouple. After reaction completion, 533 μL of a 2 mg/mL solution of 4,4'-di-tert-butylbiphenyl in DMAc was added to the wells as internal standard (IS). The reaction contents were thoroughly mixed and an aliquot (2 μL) was added to a 384-well filter plate (Agilent Technologies Inc., 201027-100, Filter Microplate, 384-well, Polypropylene, with 0.7 μm glass fiber membrane, 140 μL/well, long drip) with collection plate underneath (Analytical Sales & Services, Cat No. 384280, 384-well Collection Plate, Polyproplyene, V-bottom, 12-130 μL Working Volume) and further diluted with DMAc (28 μL). The plate containing the filtered samples was then subject to UHPLC-MS analysis with detection by UV at 254 nm and low resolution mass spectrometry detection (positive ion mode, Injection conditions: Column: Waters Acquity BEH C18 1.7 um 2.1 x 50 mm; Injection Volume = 0.5 μL; Mobile Phase A: 5:95 MeCN:H_2O with 0.05% TFA; Mobile Phase B: 95:5 MeCN:H_2O with 0.05% TFA; Temp. = 50 °C; Gradient: 0% B to 100% B over 3 min, then a 0.5 min hold at 100% B; Flow: 1 mL/min) with results displayed as P/IS.

Katritzky salts used for library experiments:

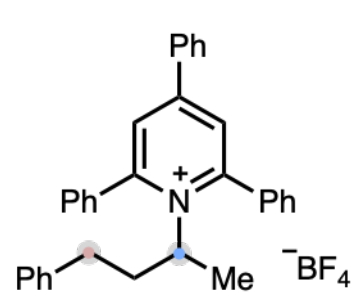

1a

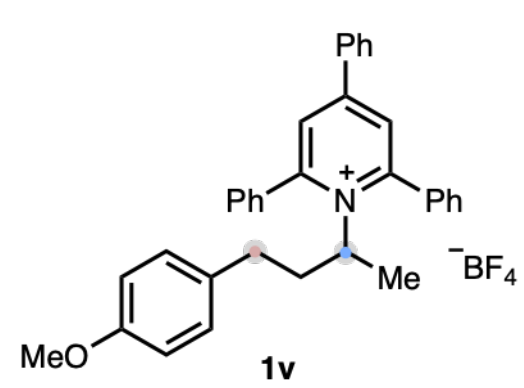

1v

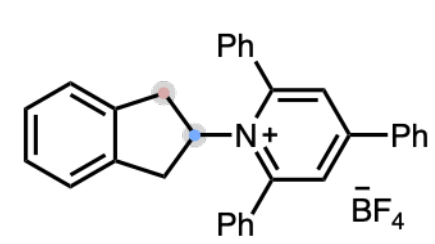

1af

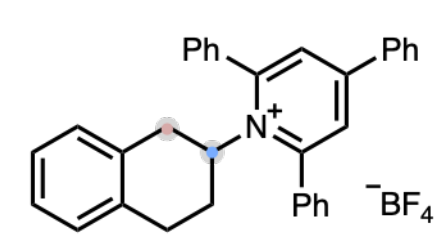

1ag

Aryl bromides used for library experiments:

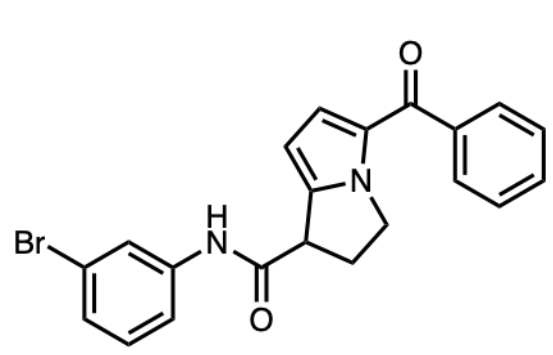

2aj, *from* Keterolac

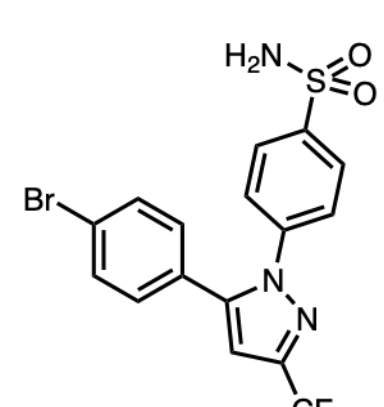

2ai, *from* Celecoxib

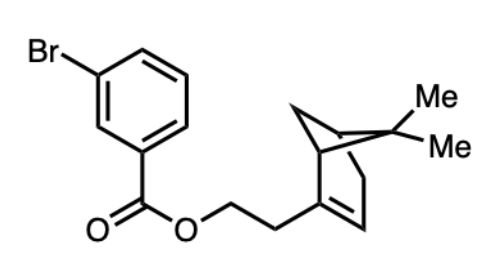

2am, *from* Nopol

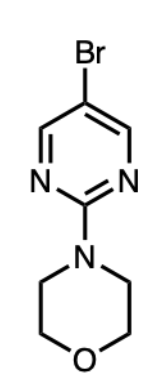

2t

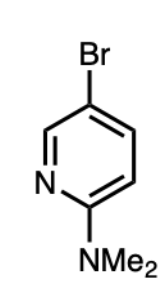

2r

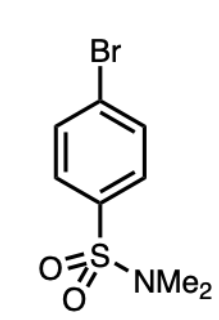

2j

Result:

The data obtained from the library experiment is tabulated below:

Note: The products from nopol were found to be nonpolar and did not absorb well at 254 nm. The product in entry 9 in the “ipso arylation reactions” was detected at 220 nm instead of 254 nm.

Migratory arylation reactions:

Entry	Katritzky salt	(Het)ArBr	Product	P/IS	Classification of P/IS
1	**1a**	**2ap**	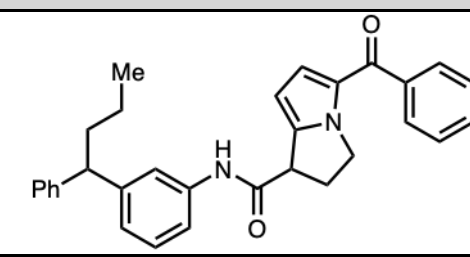	trace	< 0.1
2	**1a**	**2ao**	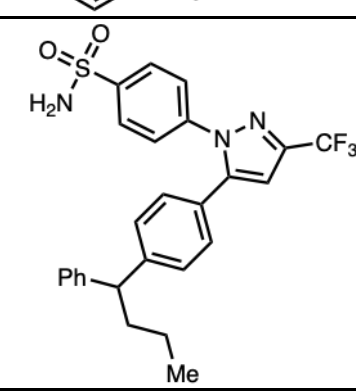	0.000	not detected
3	**1a**	**2az**	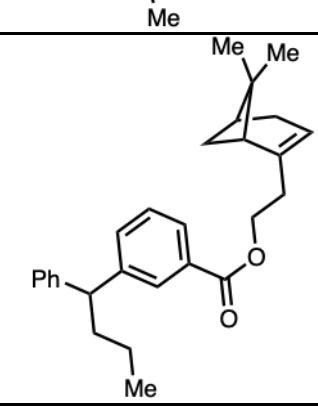	trace	<0.1
4	**1a**	**2t**	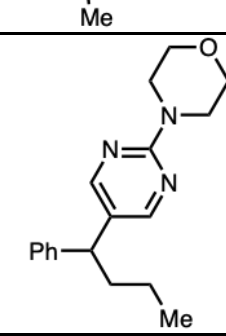	0.680	>0.1
5	**1a**	**2r**	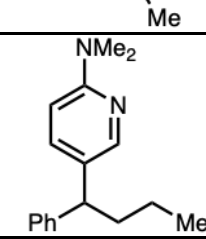	0.663	>0.1
6	**1a**	**2j**	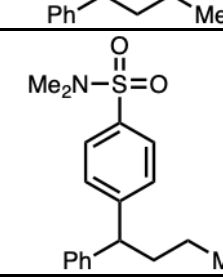	0.631	>0.1
7	**1v**	**2ap**	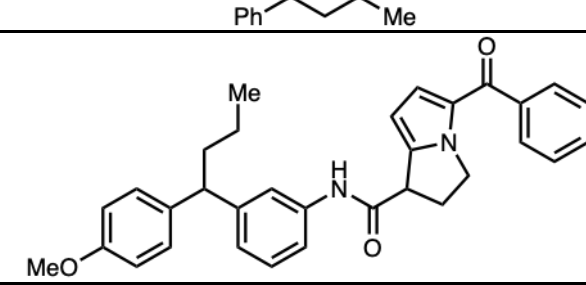	0.000	not detected
8	**1v**	**2ao**	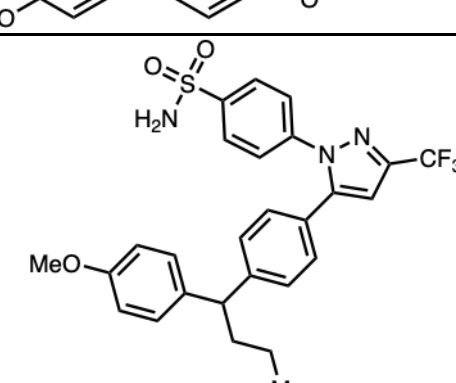	1.807	>0.1

9	**1v**	**2az**	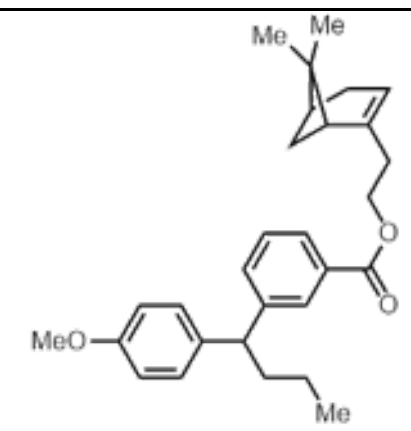	0.240	>0.1
10	**1v**	**2t**	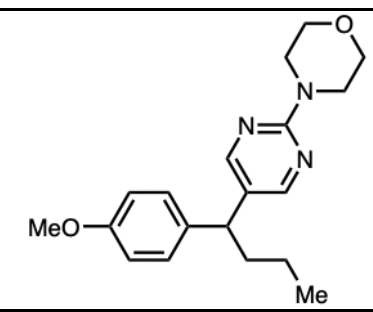	0.476	>0.1
11	**1v**	**2r**	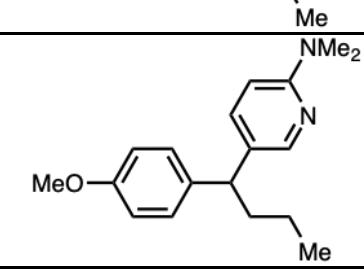	0.852	>0.1
12	**1v**	**2j**	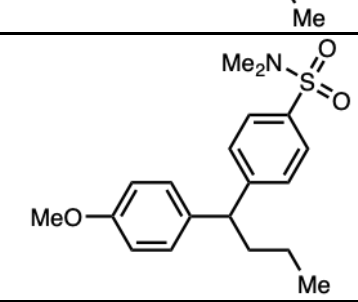	0.000	not detected
13	**1af**	**2ap**	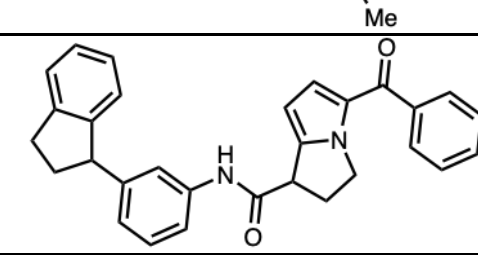	0.000	not detected
14	**1af**	**2ao**	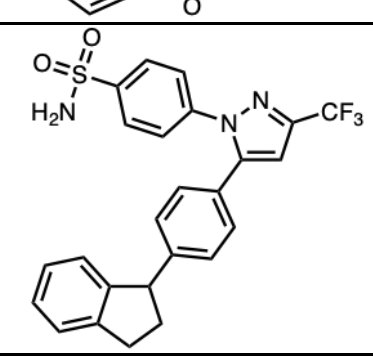	0.000	not detected
15	**1af**	**2az**	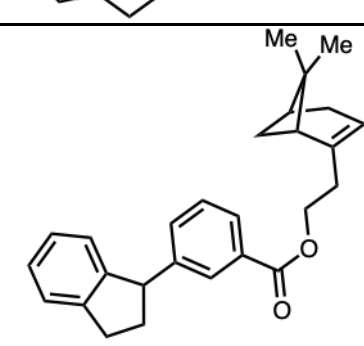	0.240	>0.1
16	**1af**	**2t**	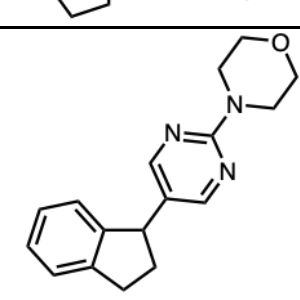	1.987	>0.1
17	**1af**	**2r**	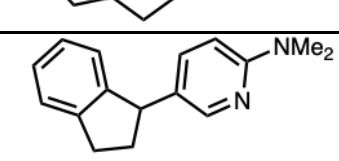	0.924	>0.1
18	**1af**	**2j**	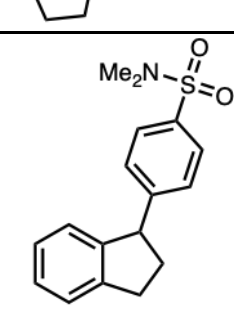	0.592	>0.1
19	**1ag**	**2ap**	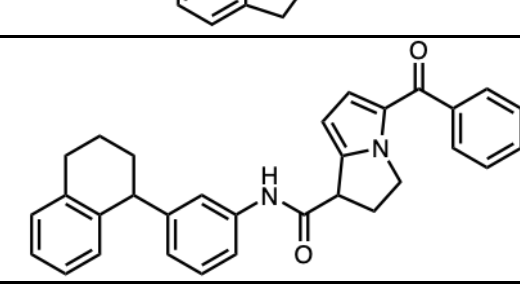	trace	<0.1
20	**1ag**	**2ao**	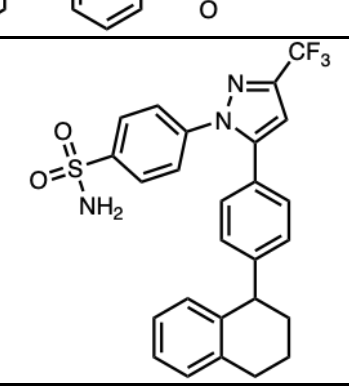	0.000	not detected

21	**1ag**	**2az**	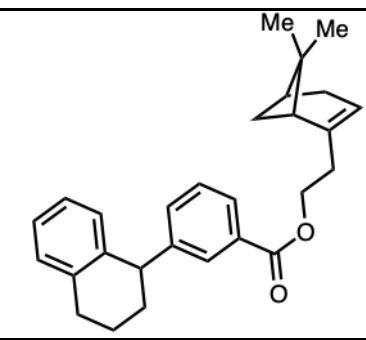	0.000	not detected
22	**1ag**	**2t**	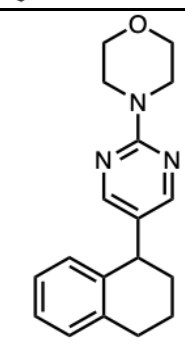	trace	<0.1
23	**1ag**	**2r**	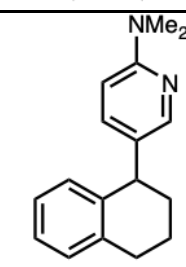	trace	<0.1
24	**1ag**	**2j**	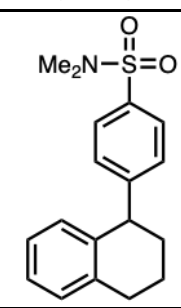	0.124	>0.1

Ipso arylation reactions:

Entry	Katritzky salt	(Het)ArBr	Product	P/IS	Classification of P/IS
1	**1a**	**2ap**	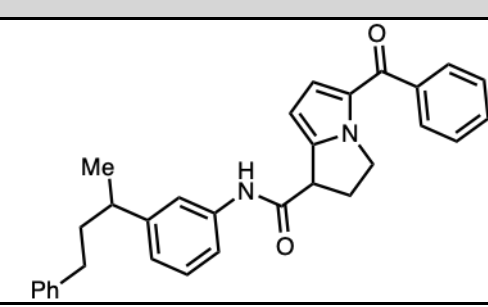	4.335	> 0.1
2	**1a**	**2ao**	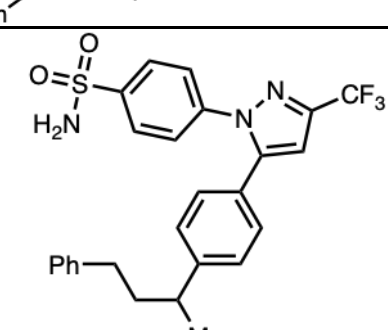	4.543	> 0.1
3	**1a**	**2az**	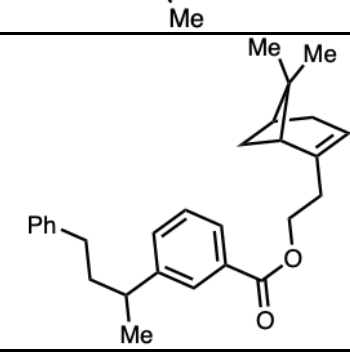	0.000	not detected
4	**1a**	**2t**	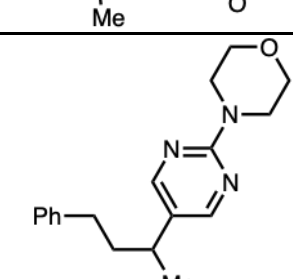	0.313	> 0.1
5	**1a**	**2r**	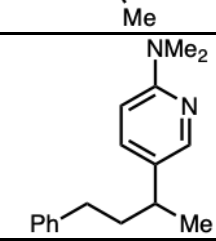	0.366	> 0.1
6	**1a**	**2j**	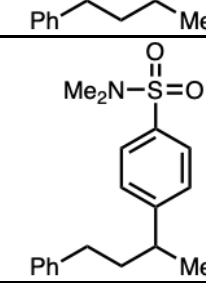	0.649	> 0.1

7	**1v**	**2ap**	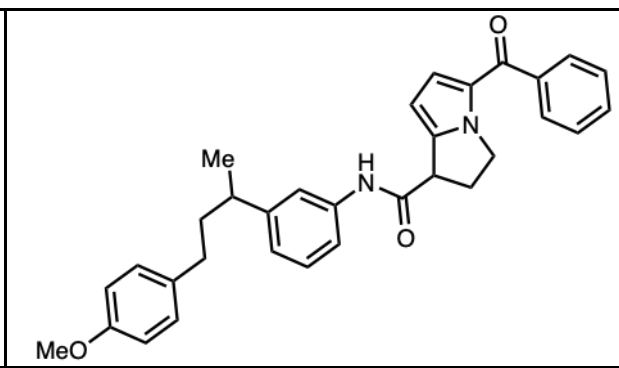	1.474	> 0.1
8	**1v**	**2ao**	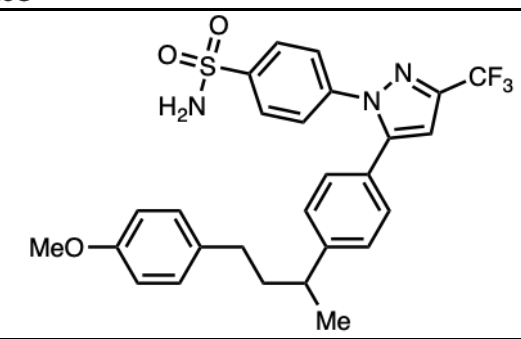	2.472	> 0.1
9	**1v**	**2az**	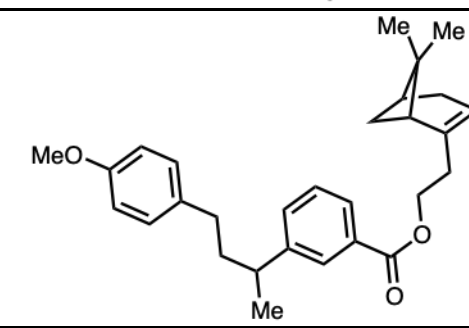	0.145	> 0.1
10	**1v**	**2t**	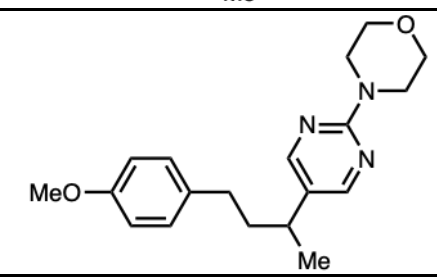	2.150	> 0.1
11	**1v**	**2r**	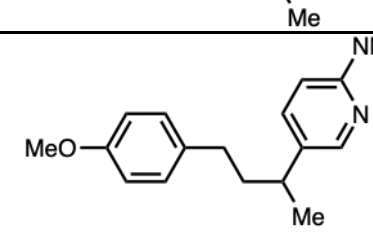	0.577	> 0.1
12	**1v**	**2j**	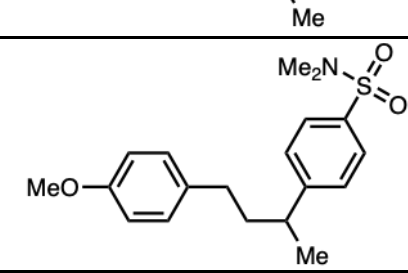	0.299	> 0.1
13	**1af**	**2ap**	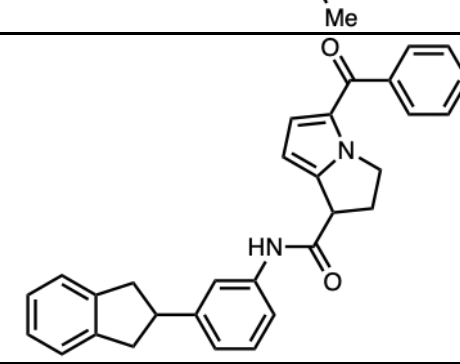	2.111	> 0.1
14	**1af**	**2ao**	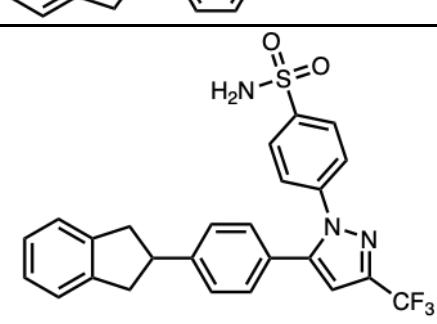	2.071	> 0.1
15	**1af**	**2az**	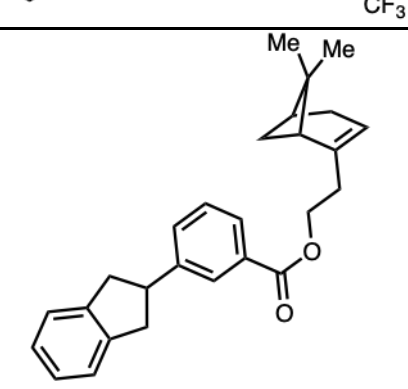	0.243	> 0.1
16	**1af**	**2t**	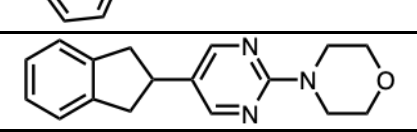	1.129	> 0.1
17	**1af**	**2r**	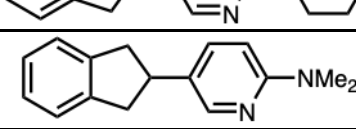	trace	< 0.1
18	**1af**	**2j**	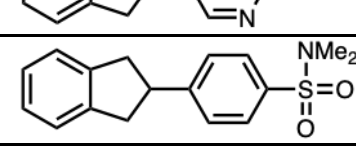	0.188	> 0.1
19	**1ag**	**2ap**	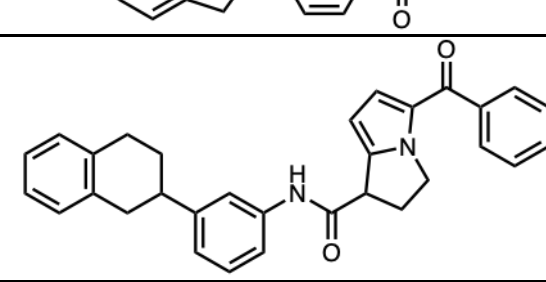	trace	< 0.1

20	**1ag**	**2ao**	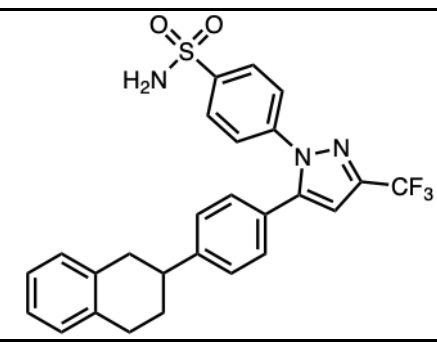	0.800	> 0.1
21	**1ag**	**2az**	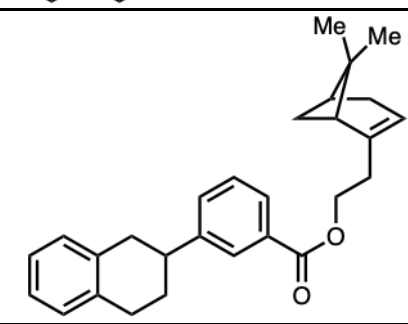	0.000	not detected
22	**1ag**	**2t**	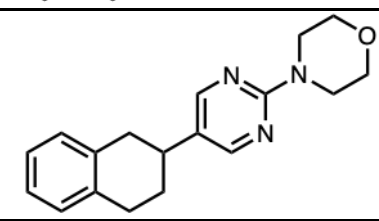	1.156	> 0.1
23	**1ag**	**2r**	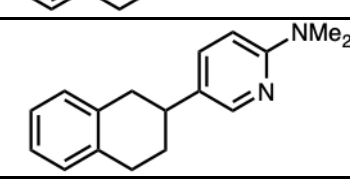	trace	< 0.1
24	**1ag**	**2j**	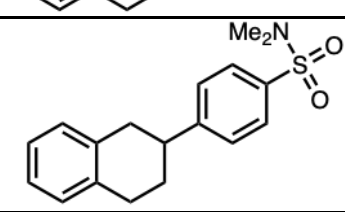	0.267	> 0.1

*Illustrative example comparing LC traces of migratory and ipso reactions between **1v** and **2t**:*

Migratory coupling

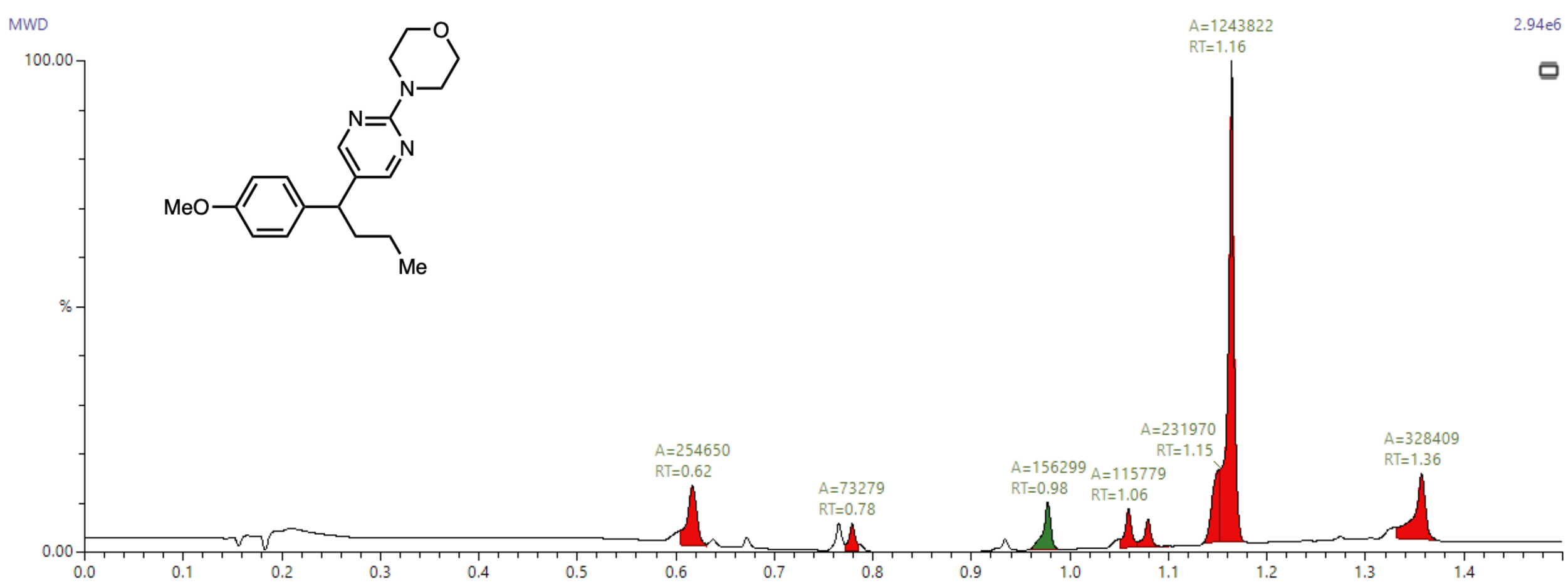

Figure S31A: Migratory coupling LC-MS ~6:1 rr by LC Area at UV 254 nm.

Ipso coupling LC-MS

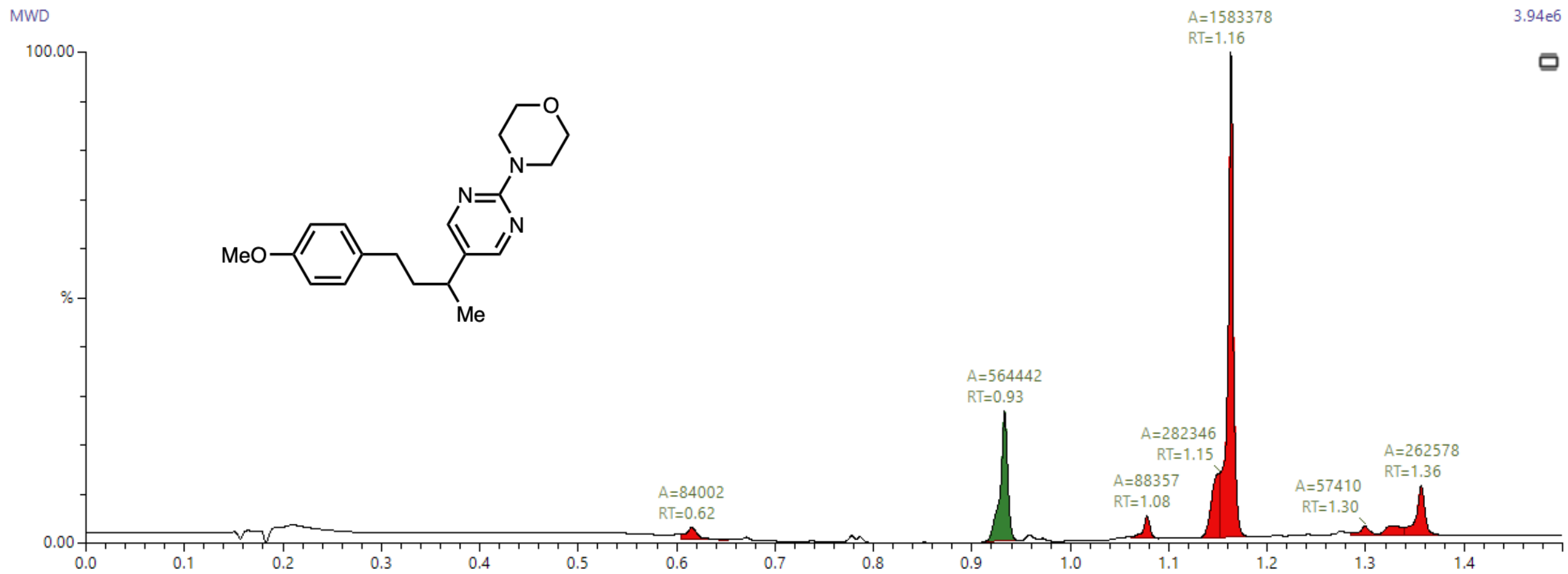

Figure S31B: Ipso coupling LC-MS.

General analytical Methods

From the 48-compound library, 22 representative reactions (10 regioisomeric pairs) were purified via preparative Reverse Phase chromatography with the following conditions: Column: XBridge C18, 19 mm x 200 mm, 5 µm particles; Flow Rate: 20 mL/min.; Column Temp. = 25 °C; Mobile Phase A: 5:95 MeCN:H_2O with 10 mM ammonium acetate or 0.05% TFA; Mobile Phase B: 95:5 MeCN:H_2O with 10 mM ammonium acetate or 0.05% TFA; Variable Gradient; Fraction collection was triggered by MS (ESI +); Fractions containing the desired product were combined and dried using centrifugal evaporation. Analytical Reverse Phase chromatography was used to determine the final purity of the library compounds.

Characterization of Migratory arylation reactions:

Entry	KS	(Het)ArBr	Product	MF	MW	MIMW	Obs. MS Ion	Purity	Retention Time for purified compound[a]
1	**1a**	**2t**	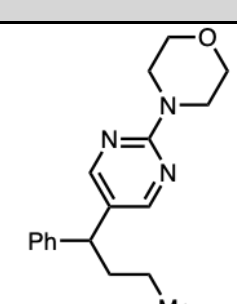	$C_{18}H_{23}N_3O$	297.4	297.18411	298.1	100%	2.05 min
2	**1a**	**2j**	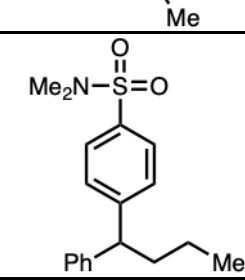	$C_{18}H_{23}NO_2S$	317.45	317.14495	318.1	100%	2.38 min

3	**1v**	**2ao**	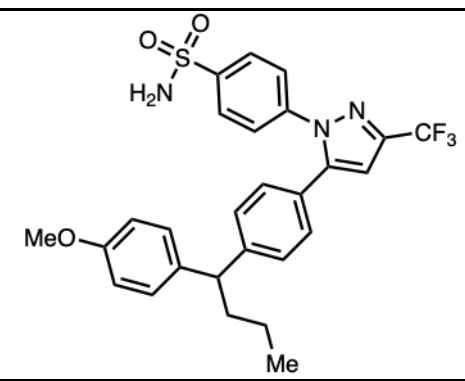	$C_{27}H_{26}F_3N_3O_3S$	529.58	529.1647	530.1	100%	2.58 min
4	**1v**	**2az**	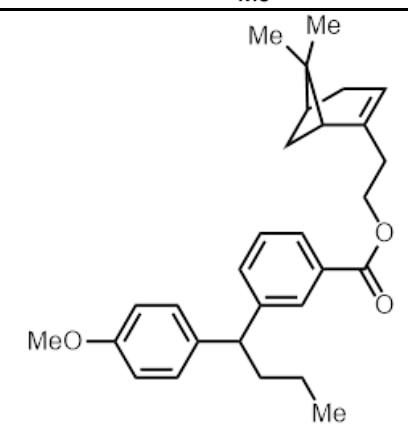	$C_{29}H_{36}O_3$	432.6	432.26645	433.2	100%	3.30 min
5	**1v**	**2t**	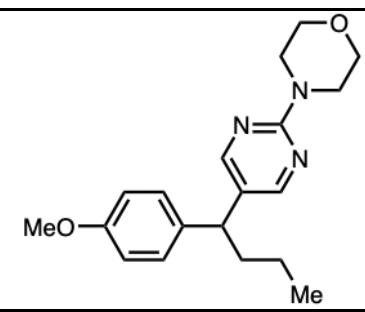	$C_{19}H_{25}N_3O_2$	327.43	327.19468	328.2	97.9%	2.04 min
6	**1v**	**2r**	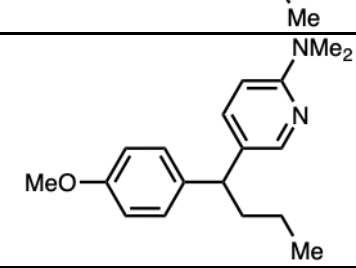	$C_{18}H_{24}N_2O$	284.4	284.18886	285.0	90.6%	1.59 min
7	**1af**	**2t**	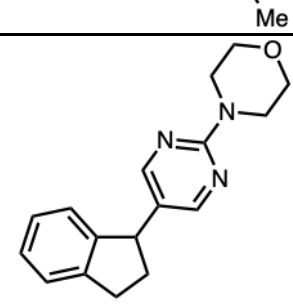	$C_{17}H_{19}N_3O$	281.36	281.15281	282.1	93.8%	1.86 min
8	**1af**	**2j**	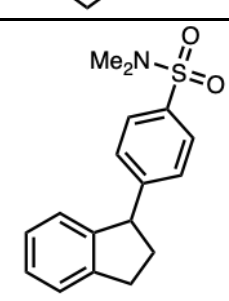	$C_{17}H_{19}NO_2S$	301.4	301.11365	302.1	100%	2.26 min
9	**1ag**	**2j**	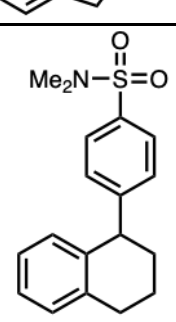	$C_{18}H_{21}NO_2S$	315.43	315.1293	316.1	100%	2.32 min

[a]Injection conditions: Column: Waters XBridge C18, 2.1 mm x 50 mm, 1.7 µm particles; Mobile Phase A: 5:95 MeCN:H_2O with 0.1% TFA; Mobile Phase B: 95:5 MeCN:H_2O with 0.1% TFA; Temp. = 50 °C; Gradient: 0% B to 100% B over 3 min., then a 0.5 min. hold at 100% B; Flow: 1 mL/min.; Detection: MS (ESI +/-) and UV (220 nm).

Characterization of Ipso arylation reactions:

Entry	KS	(Het)ArBr	Product	MF	MW	MIMW	Obs. MS Ion	Purity	Retention Time for purified compound[a]
1	**1a**	**2t**	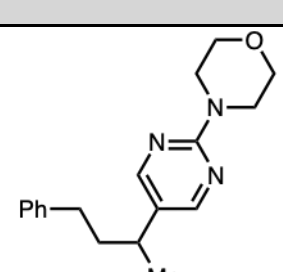	$C_{18}H_{23}N_3O$	297.4	297.18411	298.2	100%	2.01 min

2	**1a**	**2r**	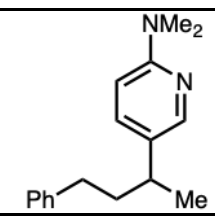	$C_{17}H_{22}N_2$	254.38	254.1783	255.0	95.3%	1.57 min
3	**1a**	**2j**	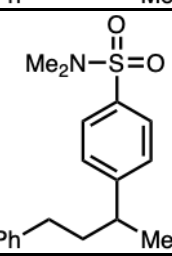	$C_{18}H_{23}NO_2S$	317.45	317.14495	318.1	96.5%	2.38 min
4	**1v**	**2ao**	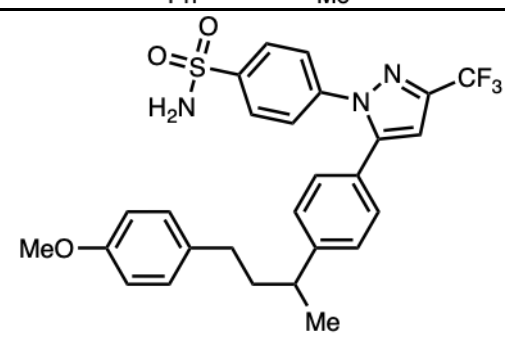	$C_{27}H_{26}F_3N_3O_3S$	529.58	529.1647	530.1	100%	2.57 min
5	**1v**	**2az**	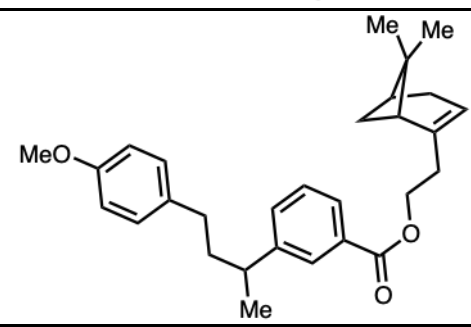	$C_{29}H_{36}O_3$	432.6	432.26645	433.2	100%	3.33 min
6	**1v**	**2t**	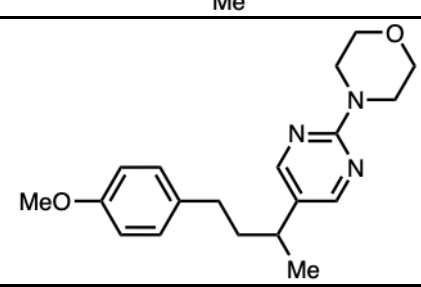	$C_{19}H_{25}N_3O_2$	327.43	327.19468	328.1	100%	1.86 min
7	**1v**	**2r**	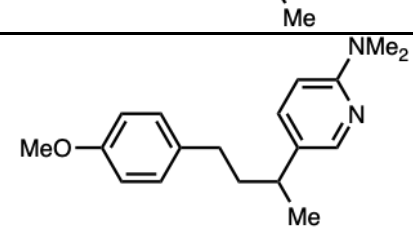	$C_{18}H_{24}N_2O$	284.4	284.18886	285.1	98.0%	1.58 min
8	**1af**	**2t**	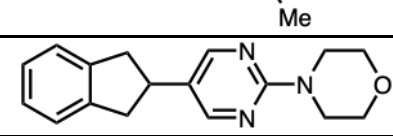	$C_{17}H_{19}N_3O$	281.36	281.15281	282.0	100%	1.77 min
9	**1af**	**2j**	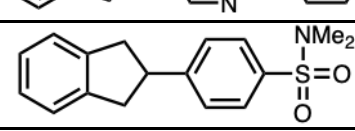	$C_{17}H_{19}NO_2S$	301.4	301.11365	302.0	97.5%	2.17 min
10	**1ag**	**2j**	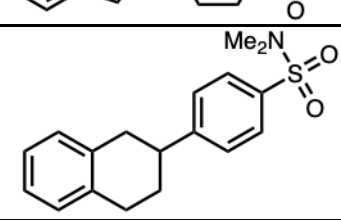	$C_{18}H_{21}NO_2S$	315.43	315.1293	316.1	100%	2.34 min

[a]Injection conditions: Column: Waters XBridge C18, 2.1 mm x 50 mm, 1.7 µm particles; Mobile Phase A: 5:95 MeCN:H_2O with 0.1% TFA; Mobile Phase B: 95:5 MeCN:H_2O with 0.1% TFA; Temp. = 50 °C; Gradient: 0% B to 100% B over 3 min., then a 0.5 min. hold at 100% B; Flow: 1 mL/min.; Detection: MS (ESI +/-) and UV (220 nm).

9. Limitations of the method

Protected, cyclic amines as terminating group

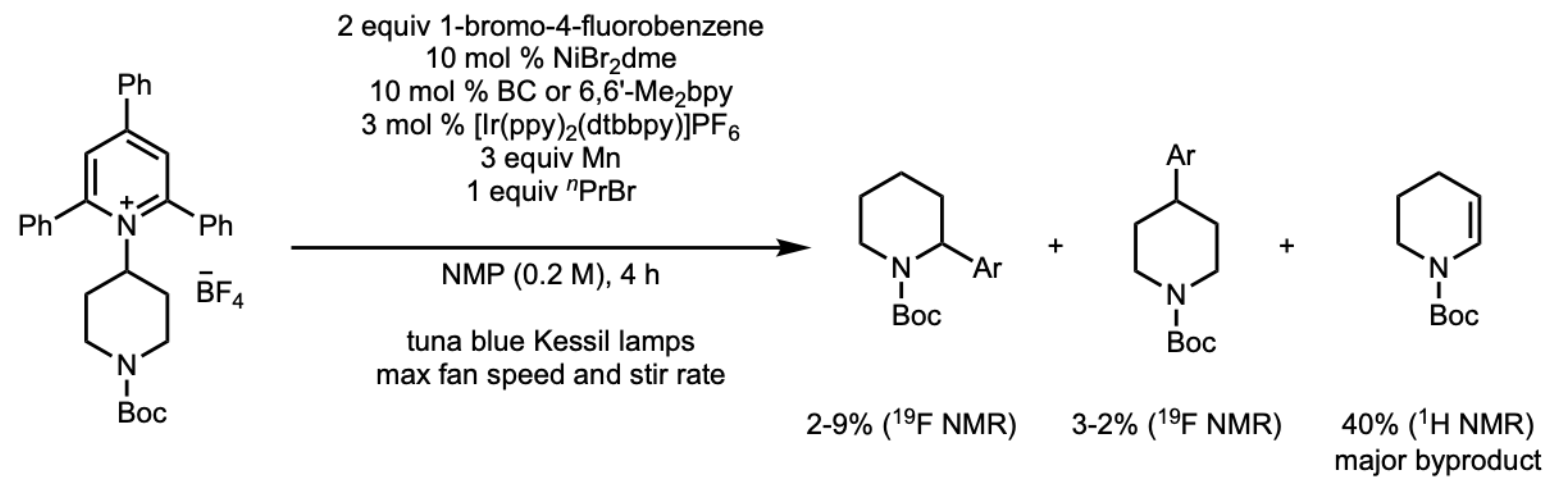

Amides as terminating group

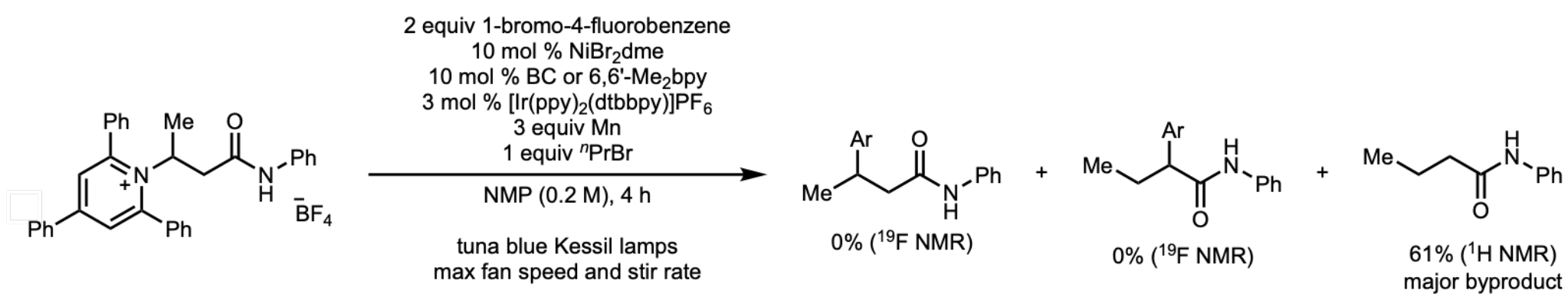

Esters as terminating group

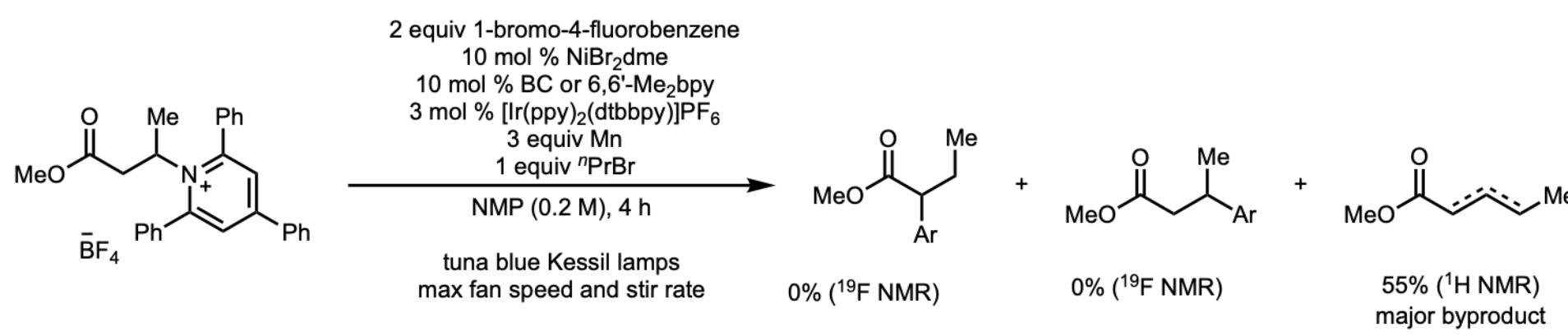

Ethers as terminating group

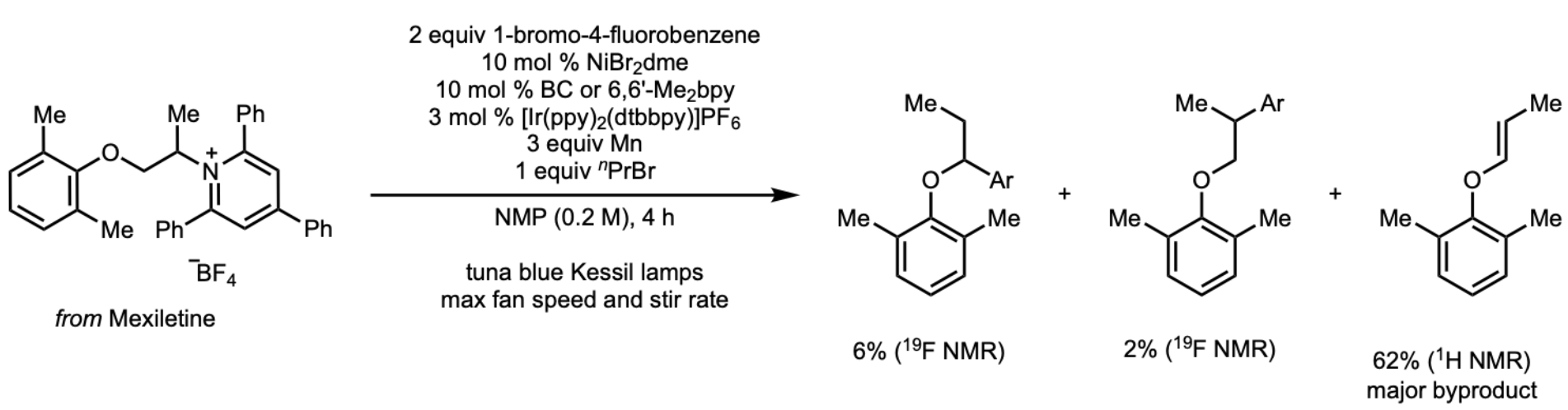

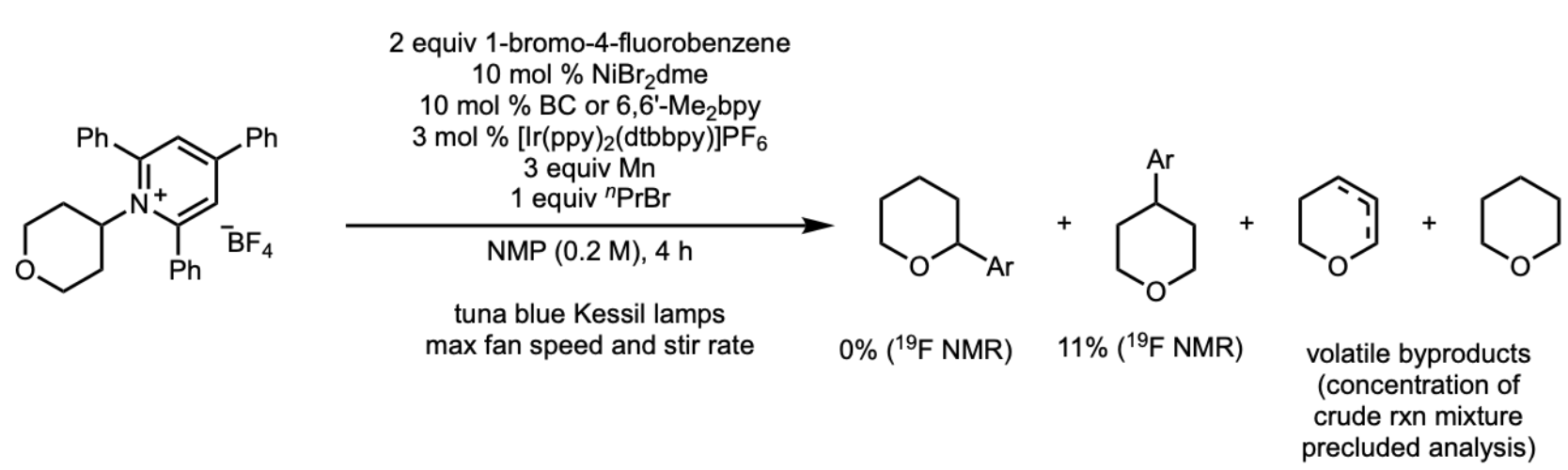

Figure S32A: Limitations to the scope.

Chain-walking through a tertiary carbon

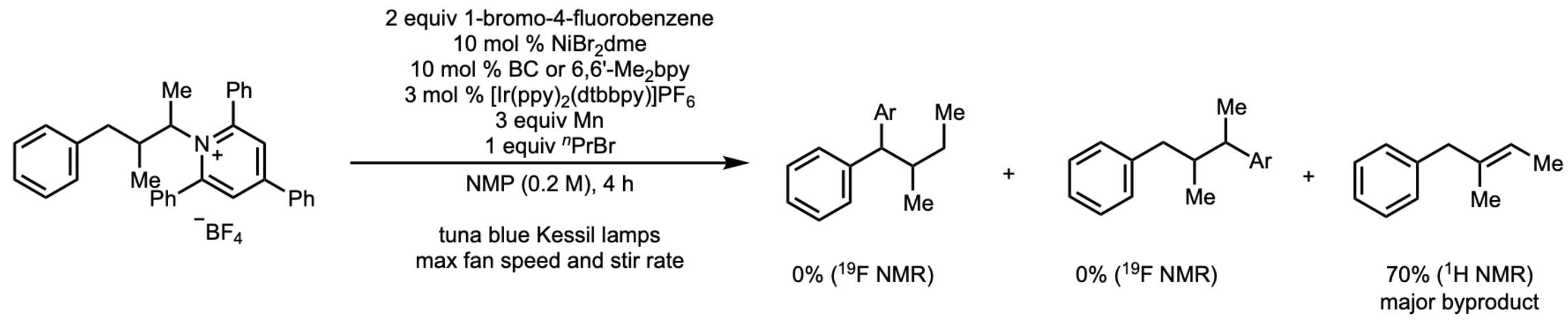

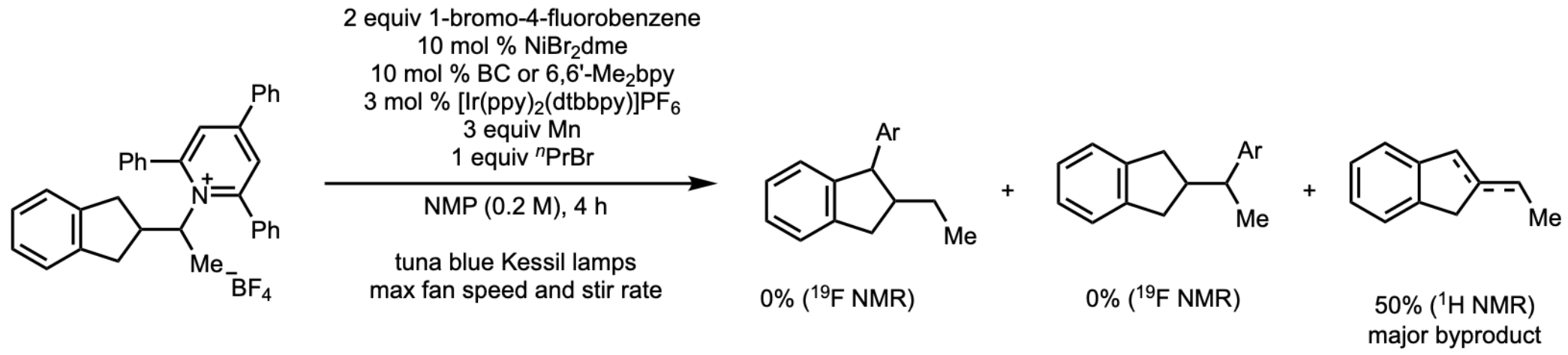

Chain-walking without any terminating group

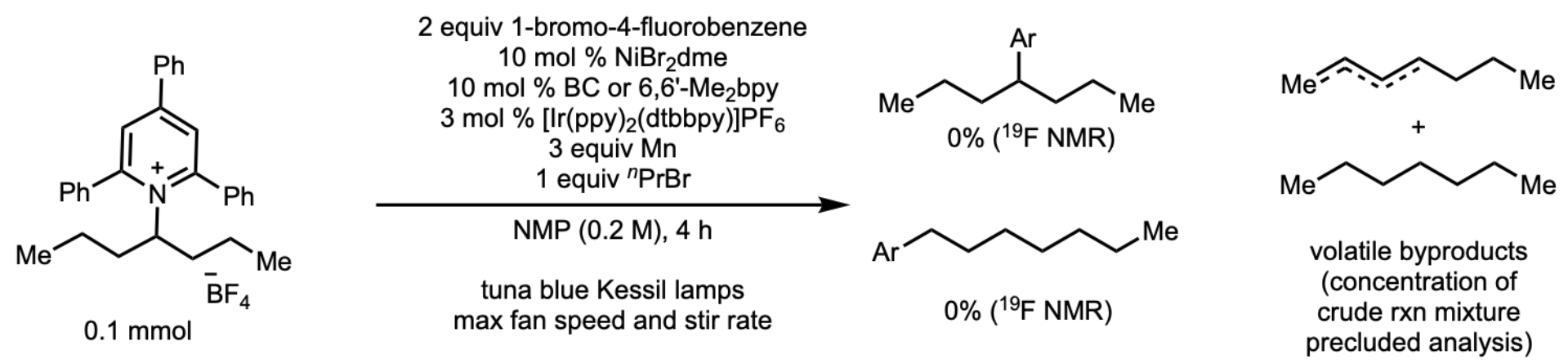

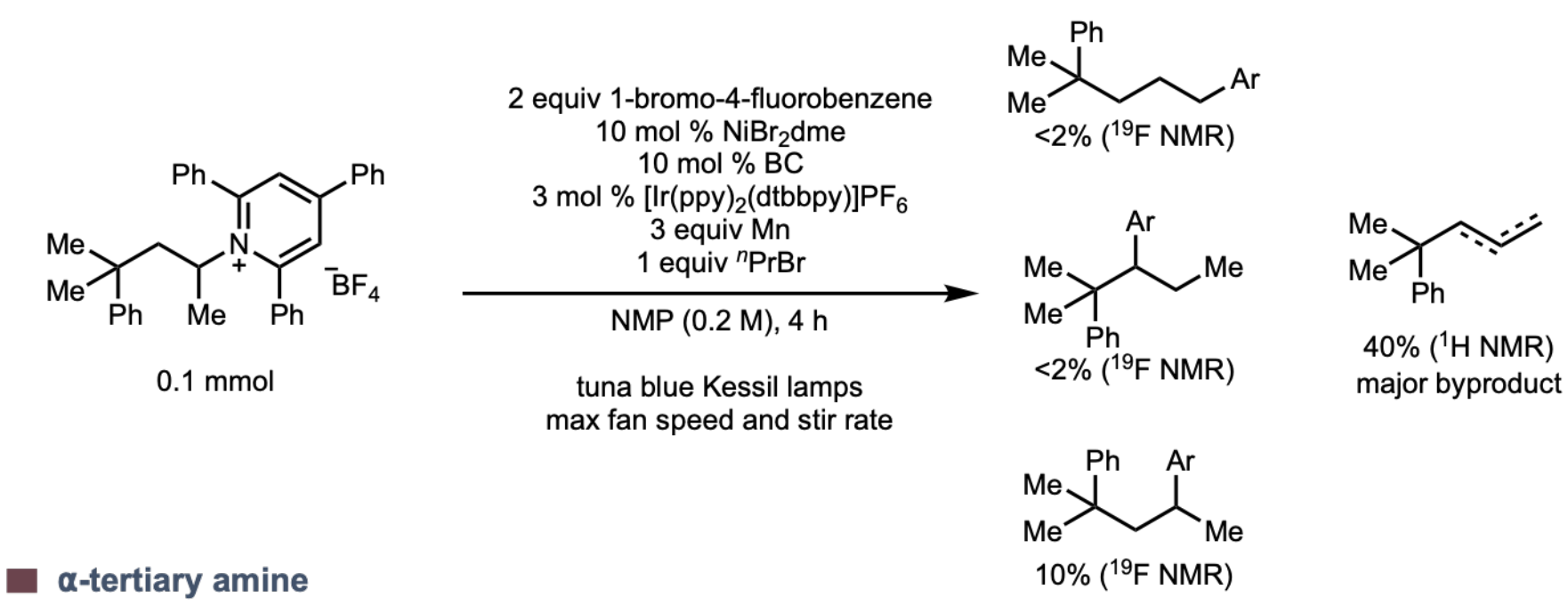

α-tertiary amine

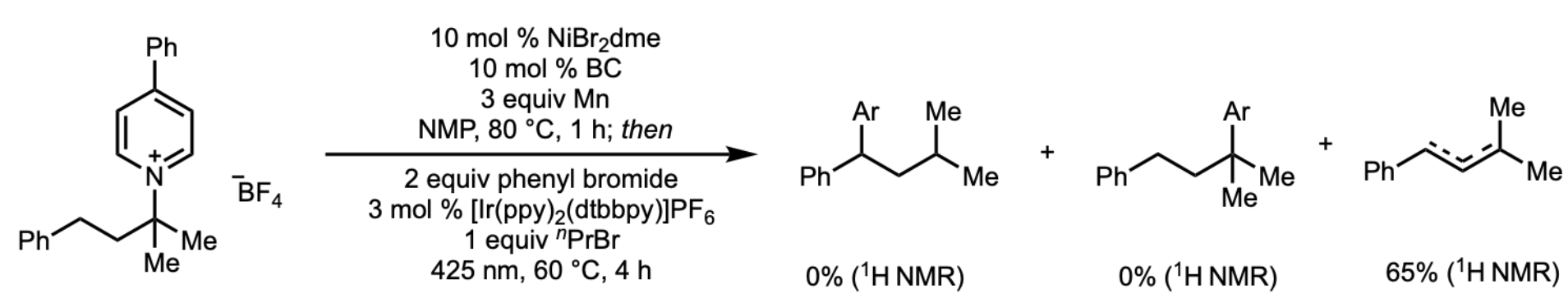

Pyridine-containing amine

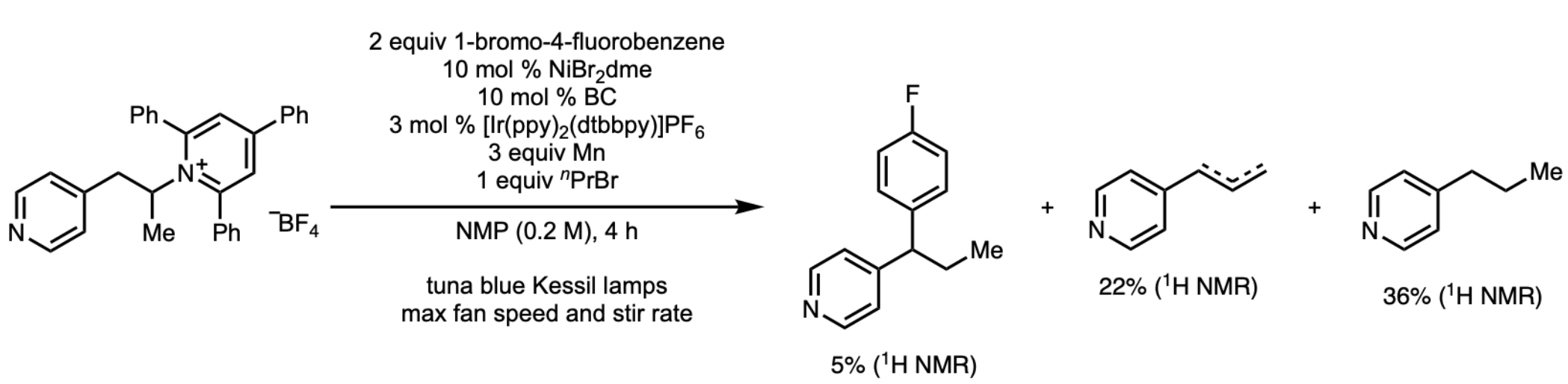

Figure S32B: Limitations to the scope.

10. Preliminary Attempts at Enantioselective Reaction

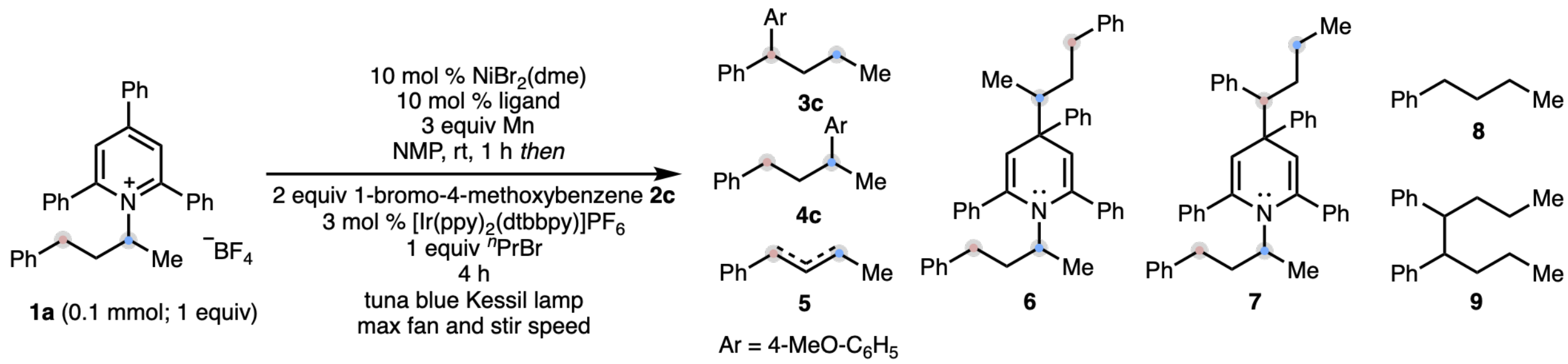

Entry	ligand	1a conv.[a] (%)	3a[a] (%)	3a (% ee)	4a[a] (%)	5[a] (%)	6[a] (%)	7[a] (%)	8[a] (%)	9[a] (%)
1	L1	99	60	~2%	12	0	0	0	0	0
2	L2	99	0	-	0	0	0	0	70	0
3	L3	99	0	-	0	11	0	0	62	0
4	L4	99	0	-	0	23	0	0	43	0
5	L5	38	0	-	7	24	0	0	34	3
6	L6	93	0	-	2	8	0	5	33	7 (14)
7[d]	L7	99	0	0	0	0	0	0	90	0

[a]Obtained by ^{1}H NMR spectroscopy using 1,3,5-trimethoxybenzene as an internal standard
[b]Obtained by ^{19}F NMR spectroscopy using 1-fluoronaphthalene as an internal standard
[c]Numbers based on conversion of **1a**

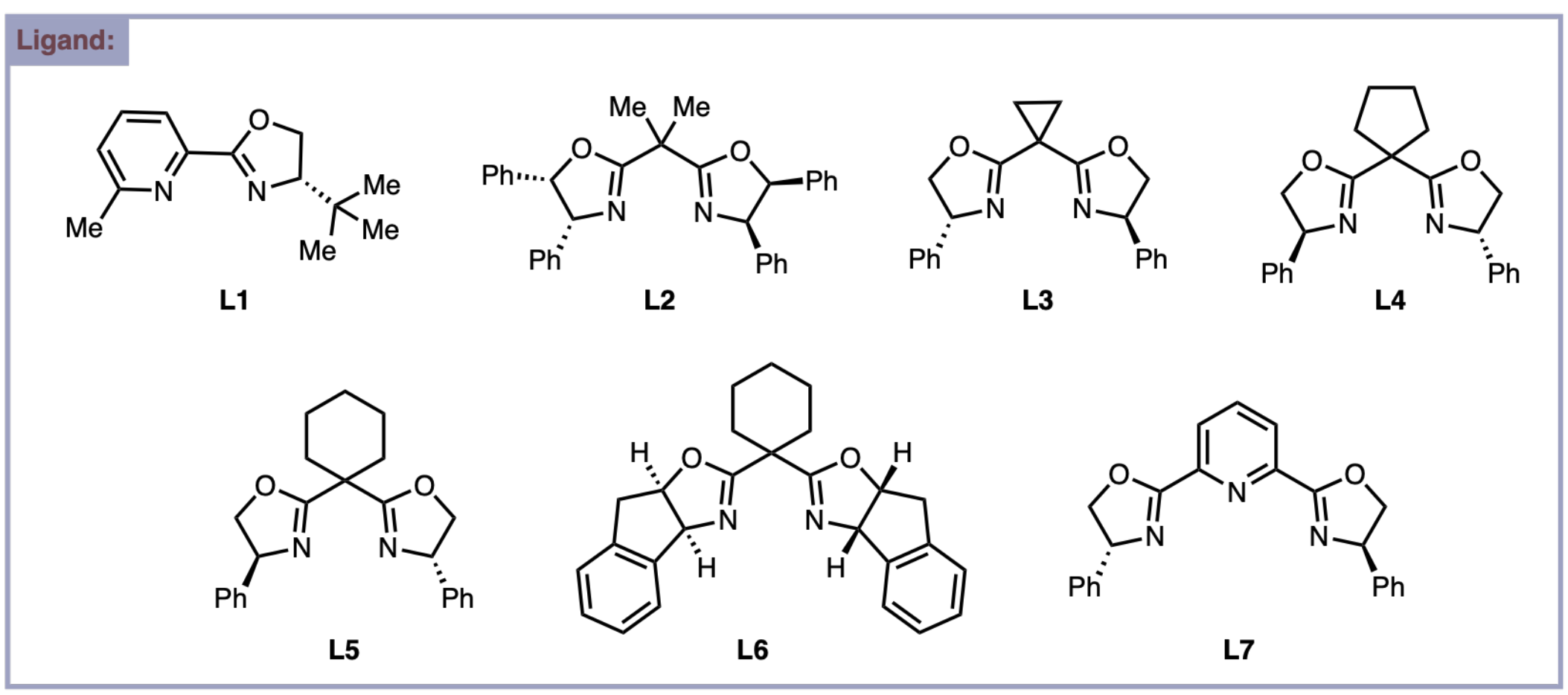

The above reactions were conducted according to *Method B* described below in *General Procedure 1* with 1-bromo-4-methoxybenzene **2c**. Product 3c from entry 1 was isolated in 5:1 rr and its enantiomeric excess was determined to be approximately 2% using chiral HPLC analysis.

HPLC:

HPLC conditions: CHIRACEL OJ-H column, 0.7% EtOH in hexane, 0.5 mL/min, 210 nm UV detector.

*HPLC trace for Racemic **3c***

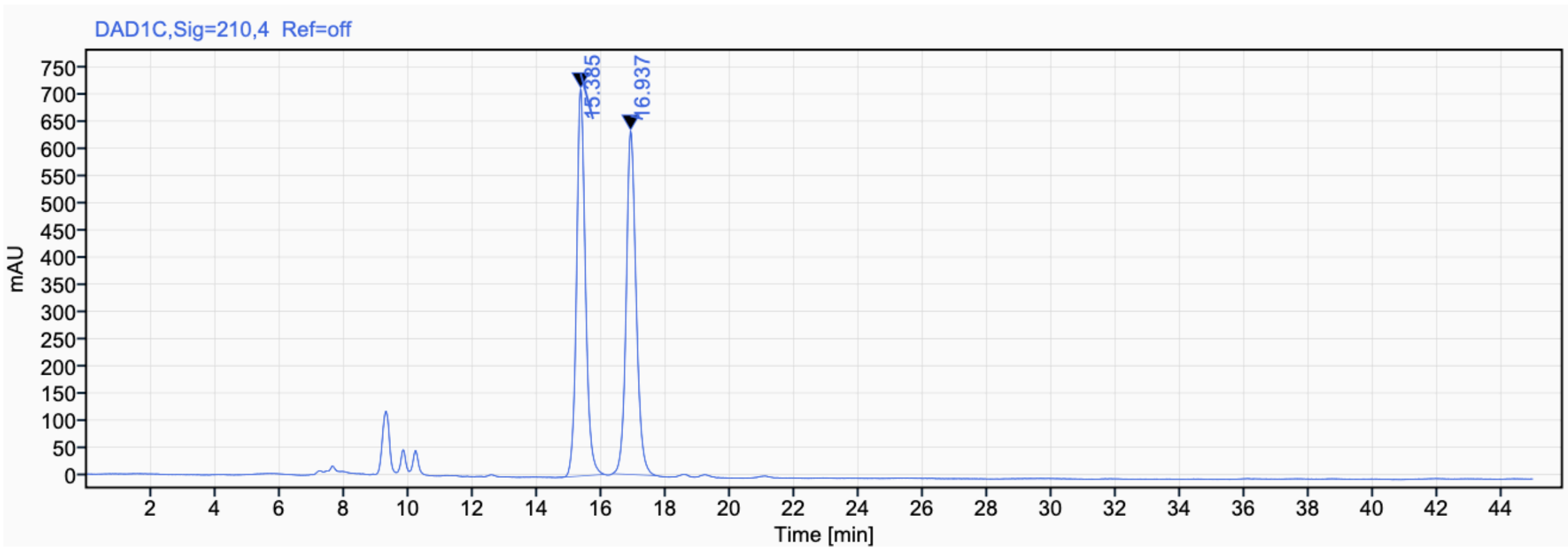

Signal: DAD1C,Sig=210,4 Ref=off

RT [min]	Type	Width [min]	Area	Height	Area%
15.385	MM m	1.37	13637.33	712.32	50.11
16.937	MM m	1.39	13578.07	631.83	49.89
		**Sum**	**27215.40**		

*HPLC trace for **3c** from Entry 1*

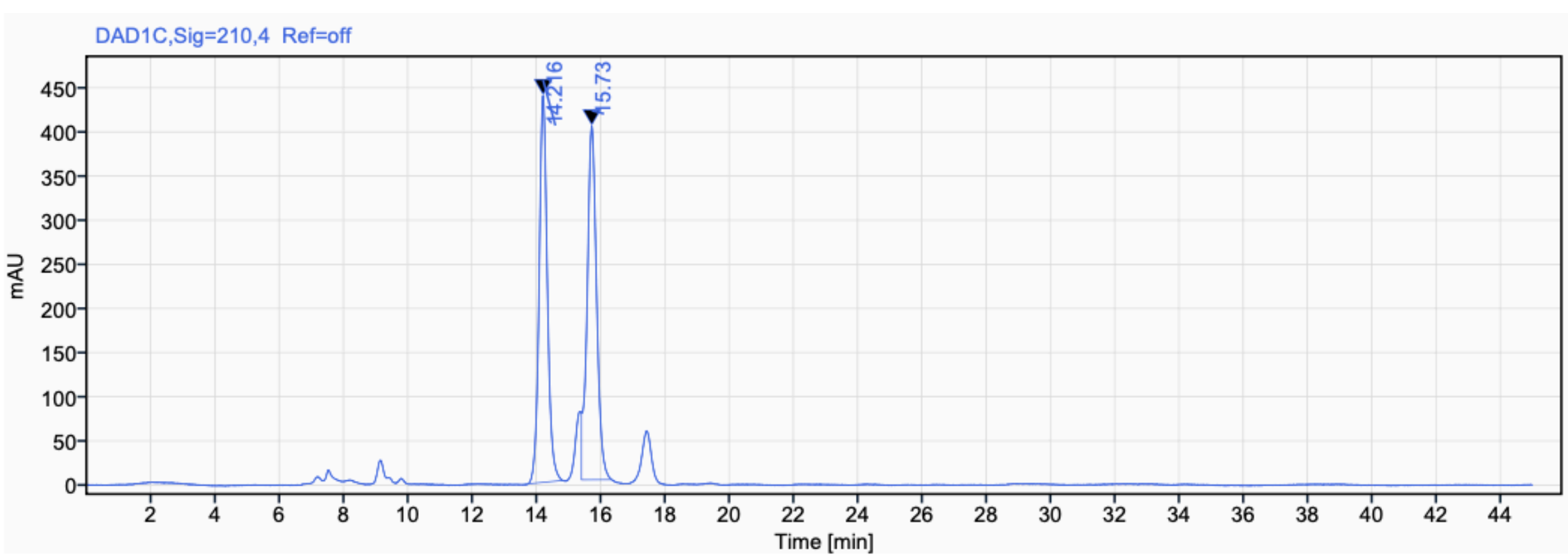

Signal: DAD1C,Sig=210,4 Ref=off

RT [min]	Type	Width [min]	Area	Height	Area%
14.216	MM m	1.06	7811.53	438.40	48.65
15.730	MM m	0.94	8244.00	400.98	51.35
		**Sum**	**16055.53**		

11. NMR Spectra

1-Fluoro-4-(1-phenylbutyl)benzene **3a**:

^{1}H NMR (300 MHz; $CDCl_3$):

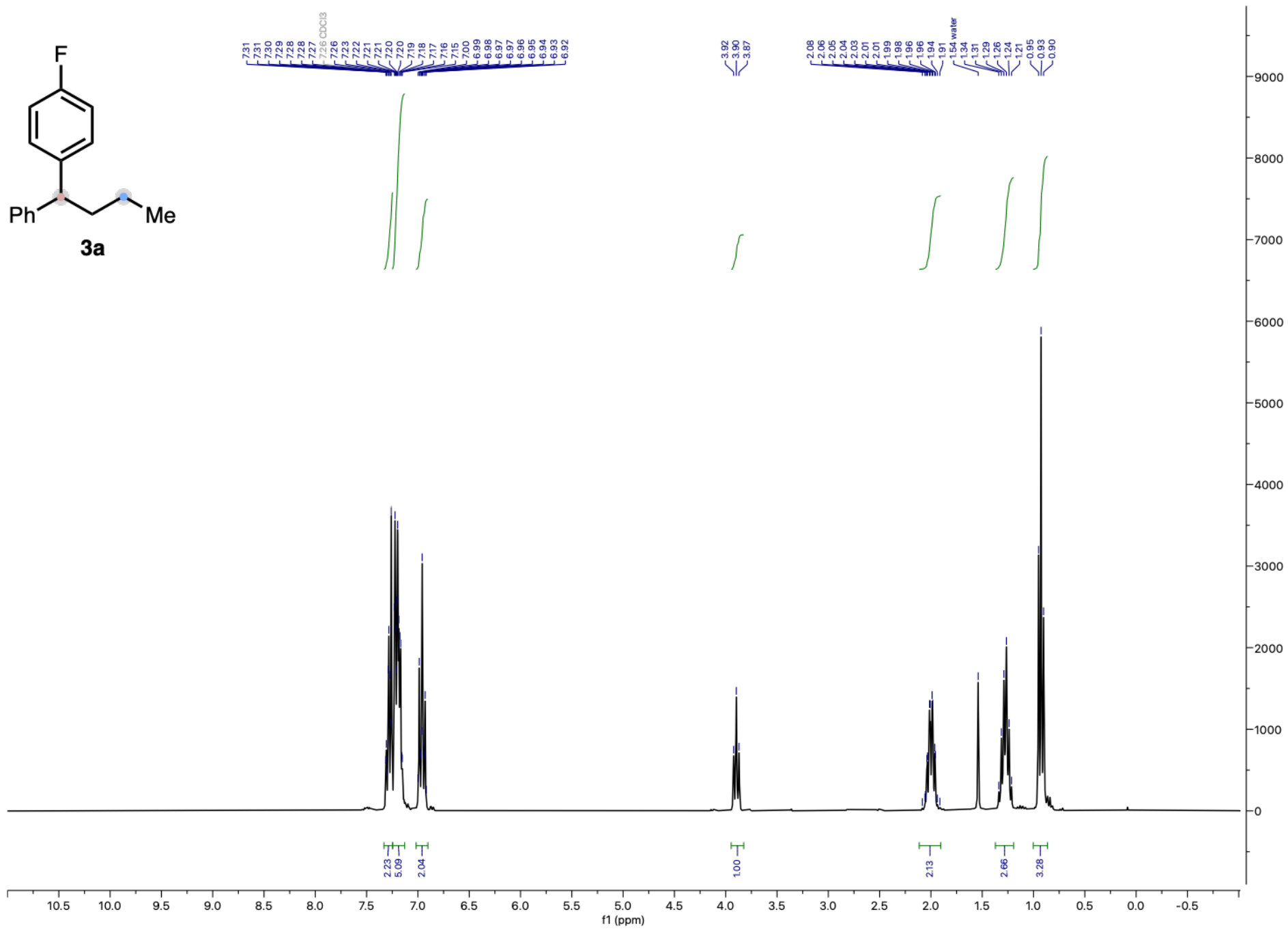

^{13}C NMR (126 MHz; $CDCl_3$):

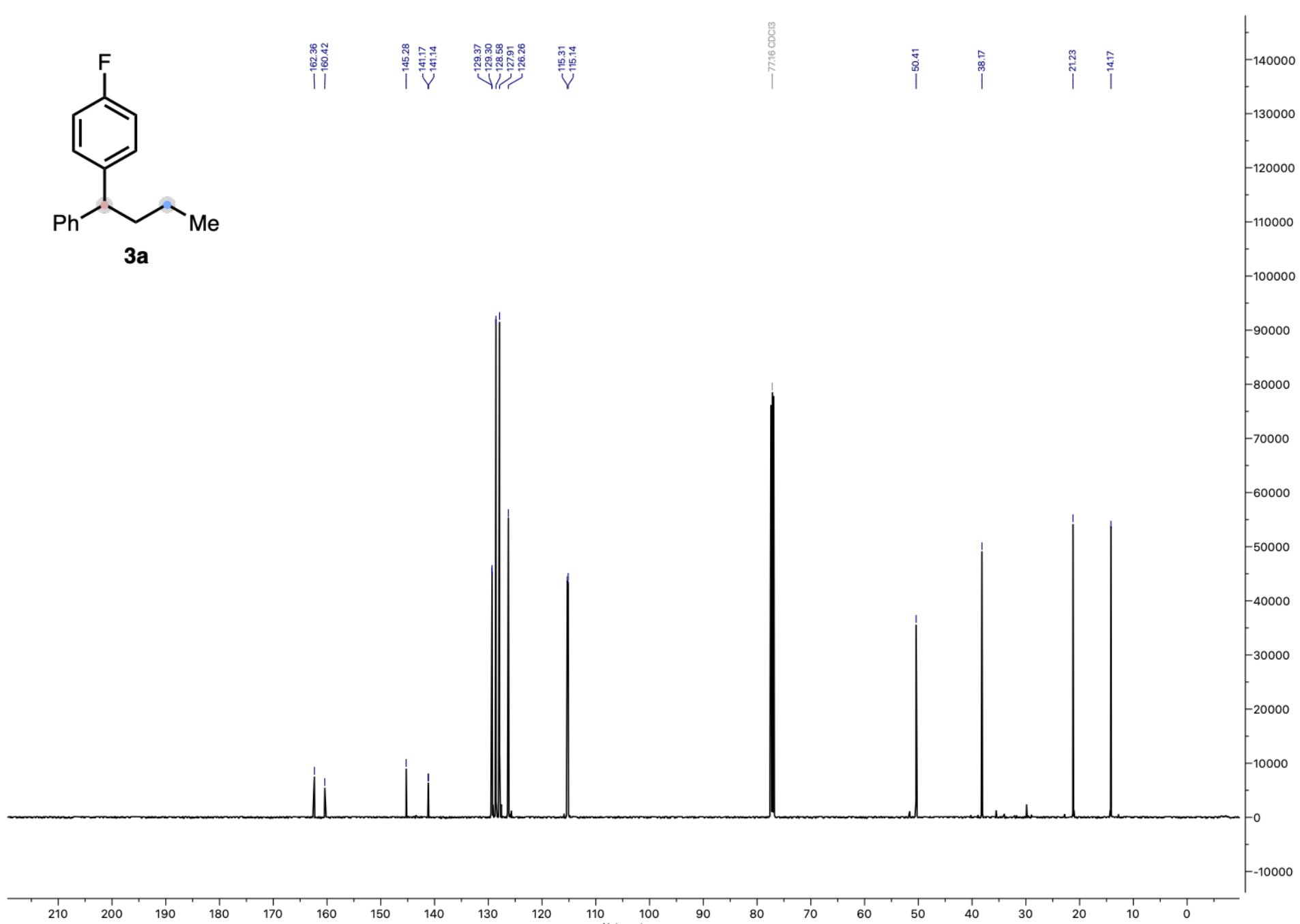

^{19}F NMR (282 MHz; $CDCl_3$):

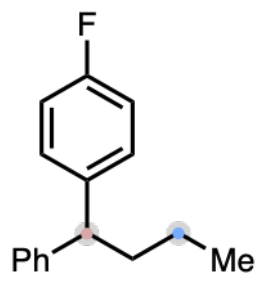

3a

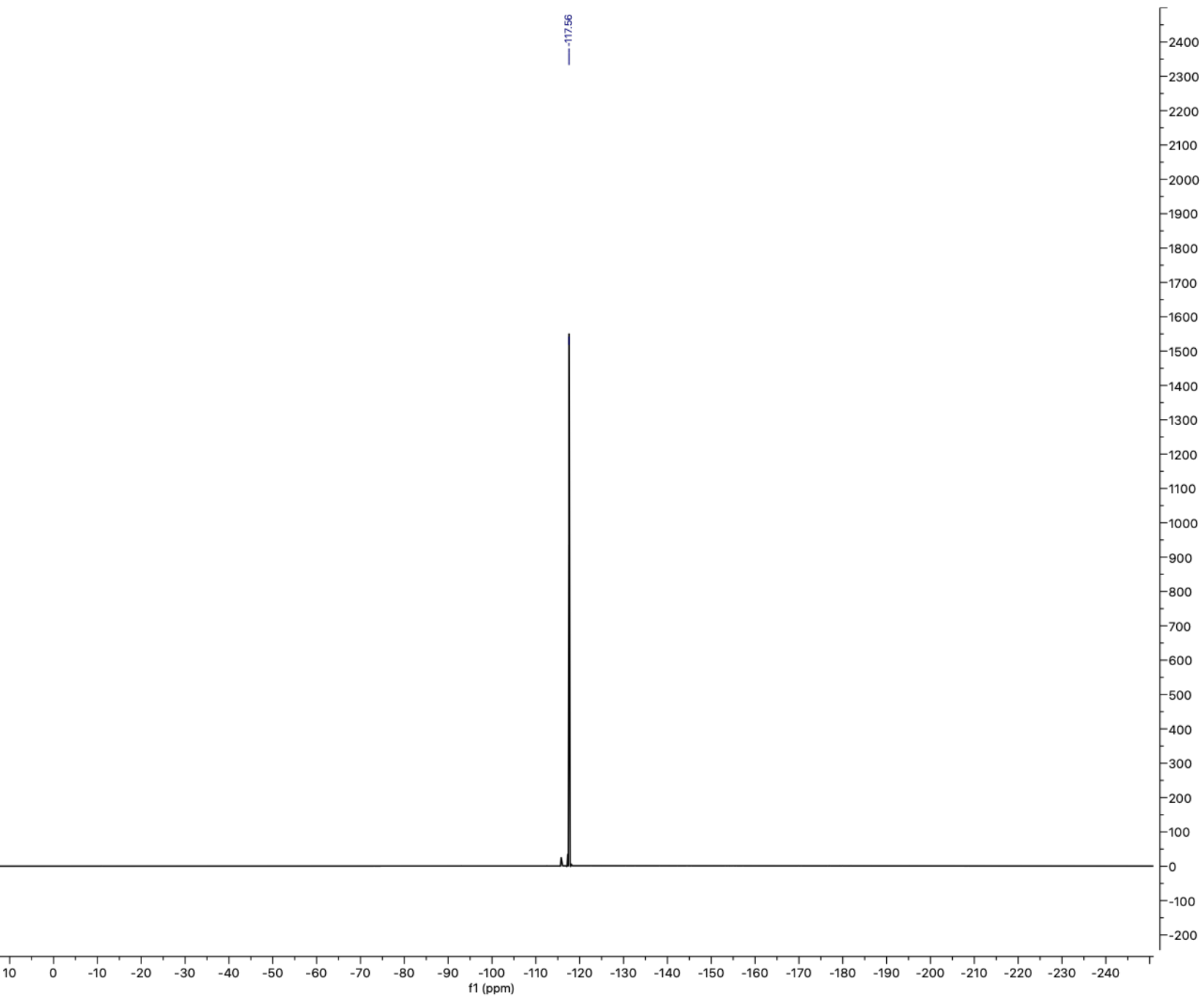

N,*N*-Dimethyl-4-(1-phenylbutyl)aniline **3b:**

^{1}H NMR (400 MHz; $CDCl_3$):

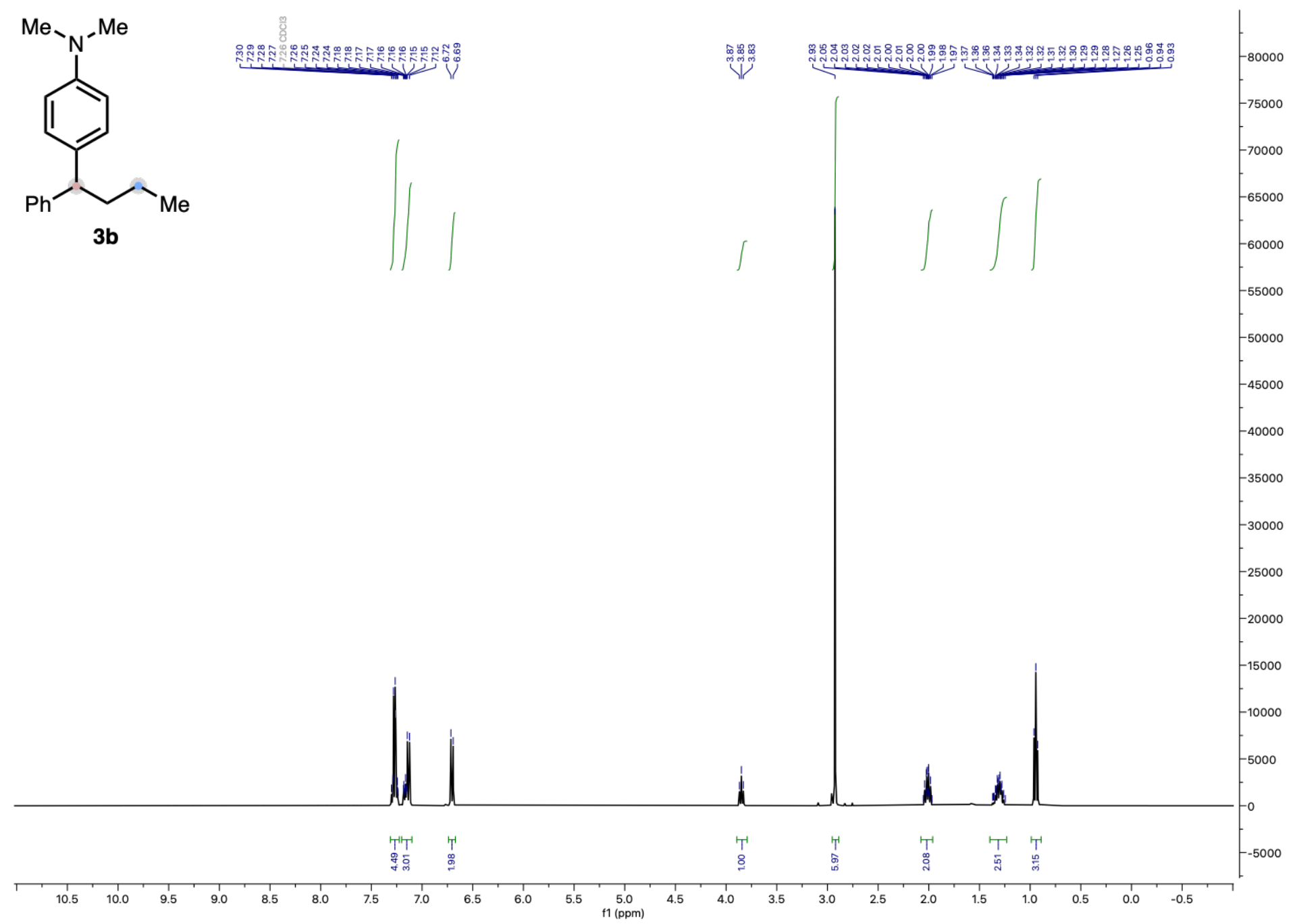

^{13}C NMR (126 MHz; $CDCl_3$):

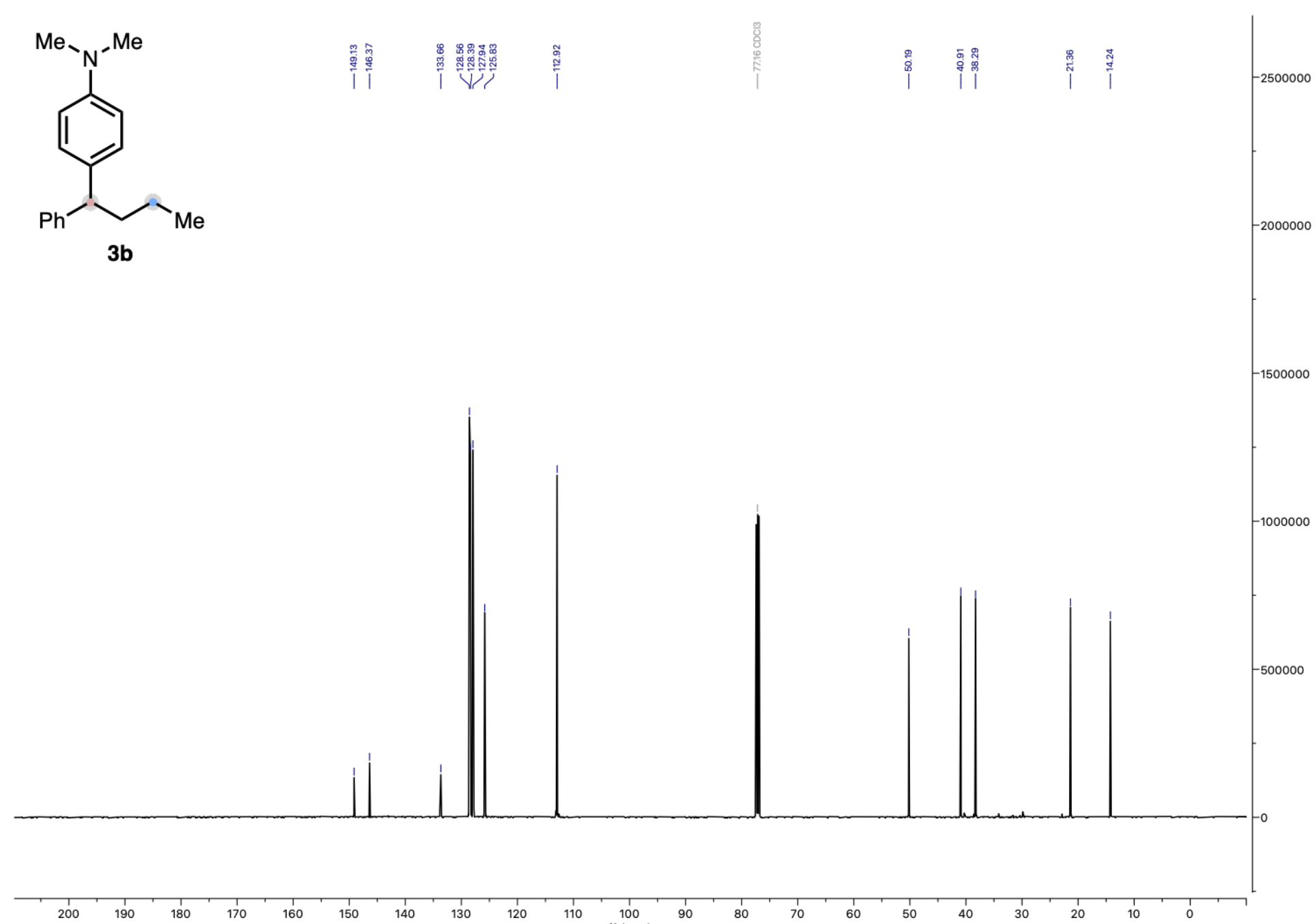

1-Methoxy-4-(1-phenylbutyl)benzene **3c**:

^{1}H NMR (500 MHz, $CDCl_3$):

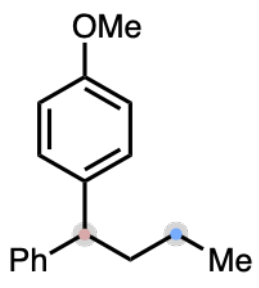

3c

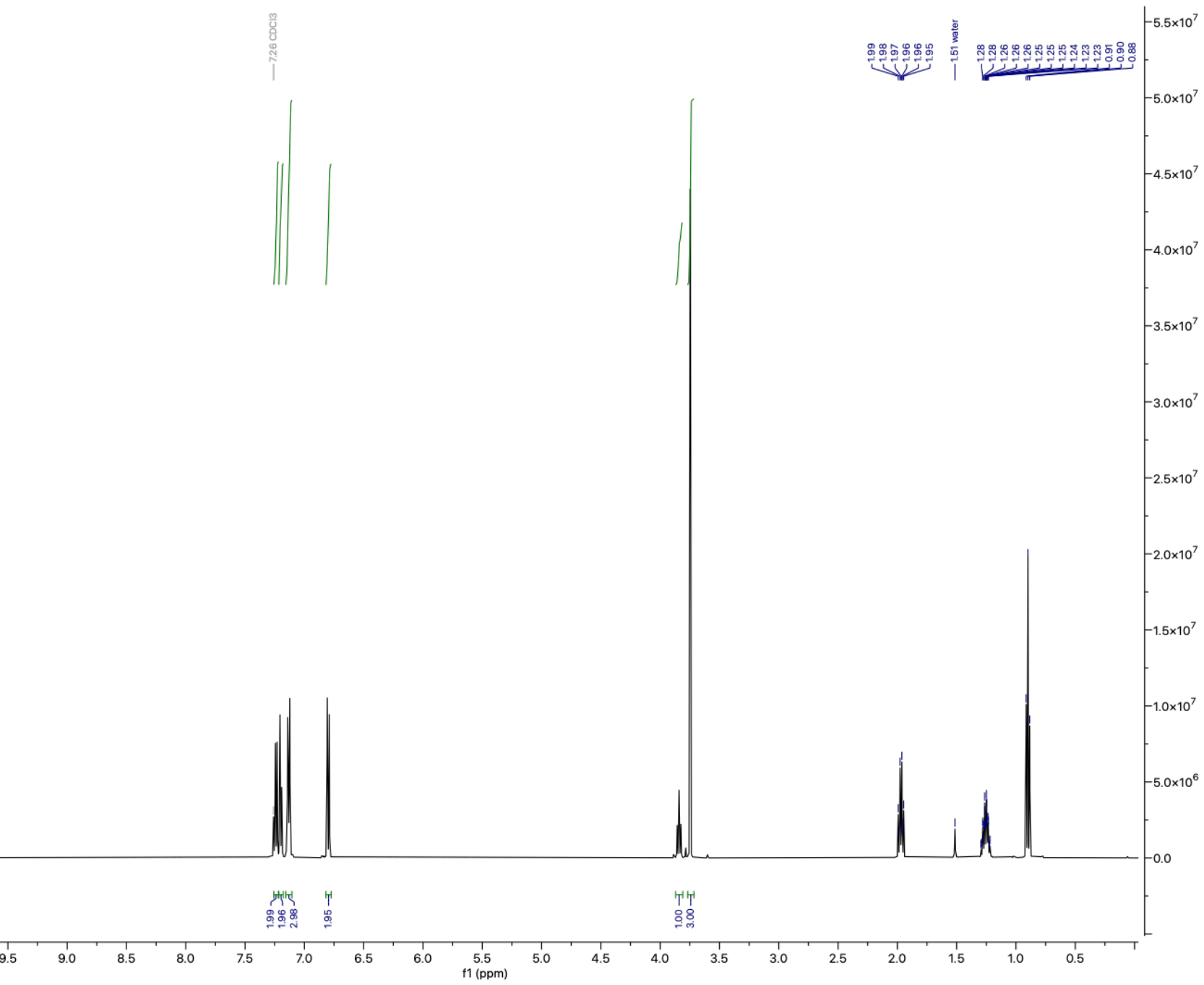

^{13}C NMR (126 MHz, $CDCl_3$):

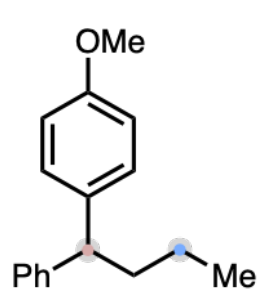

3c

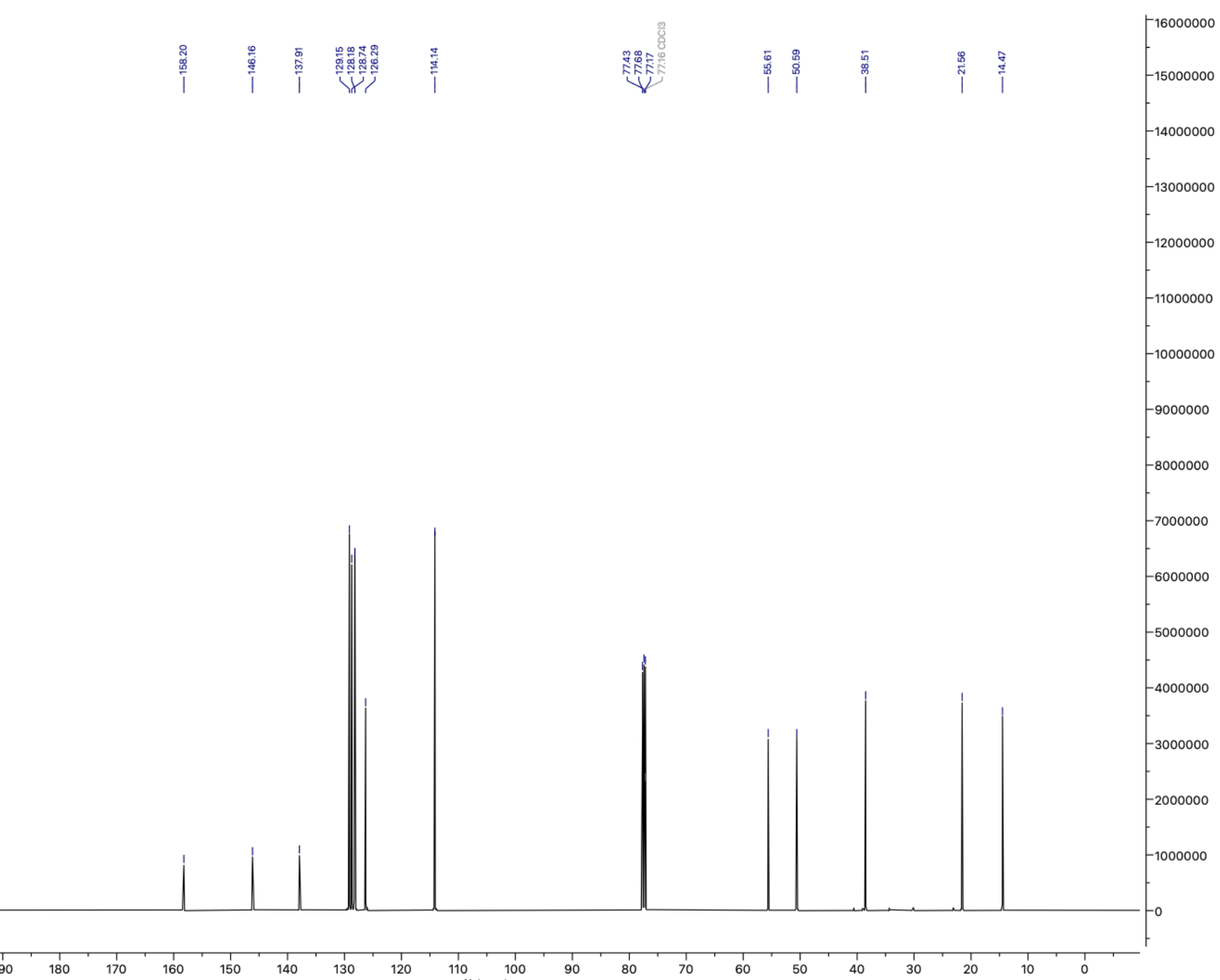

Methyl(4-(1-phenylbutyl)phenyl)sulfane **3d**:

^{1}H NMR (500 MHz, $CDCl_3$):

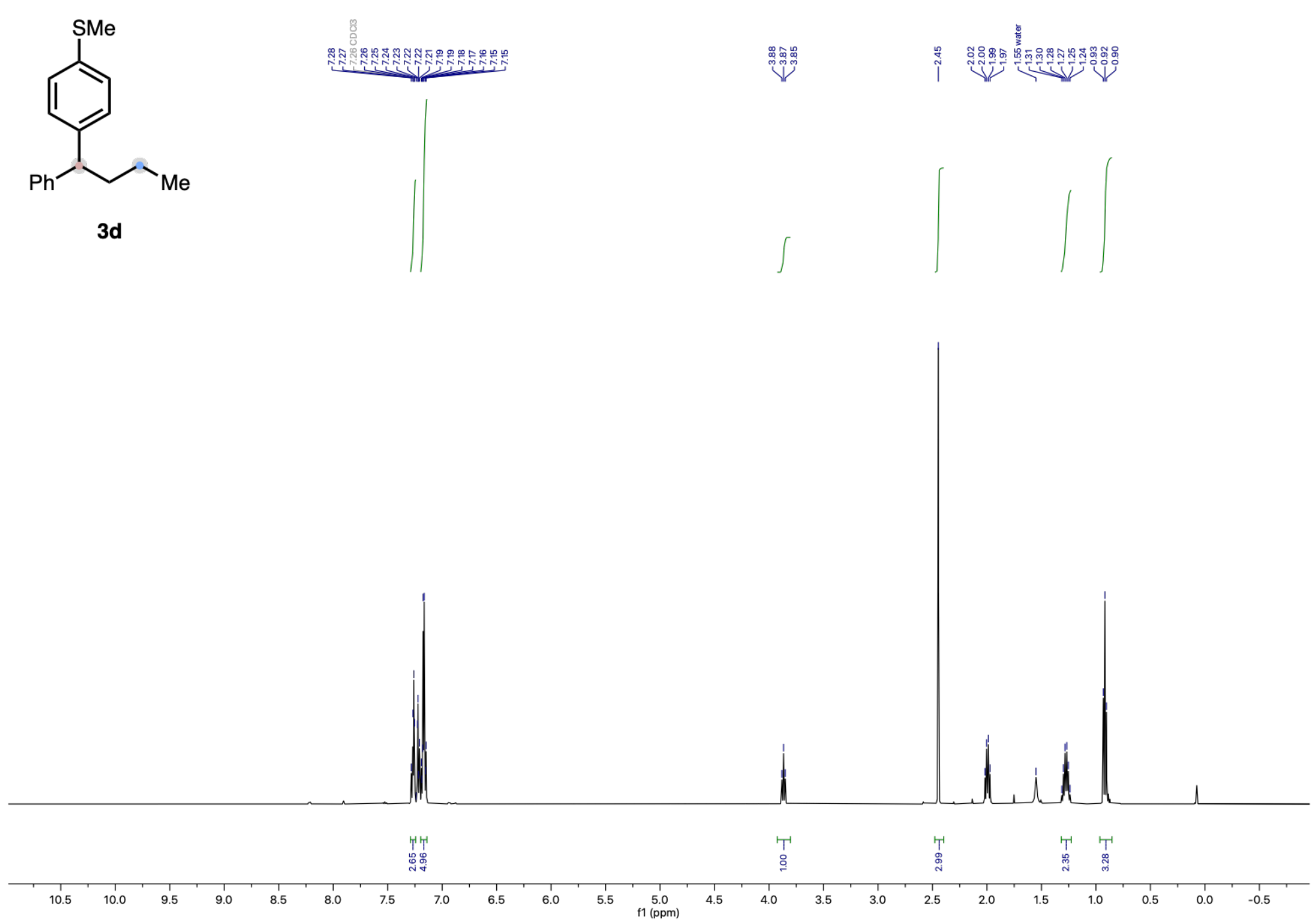

^{13}C NMR (126 MHz, $CDCl_3$):

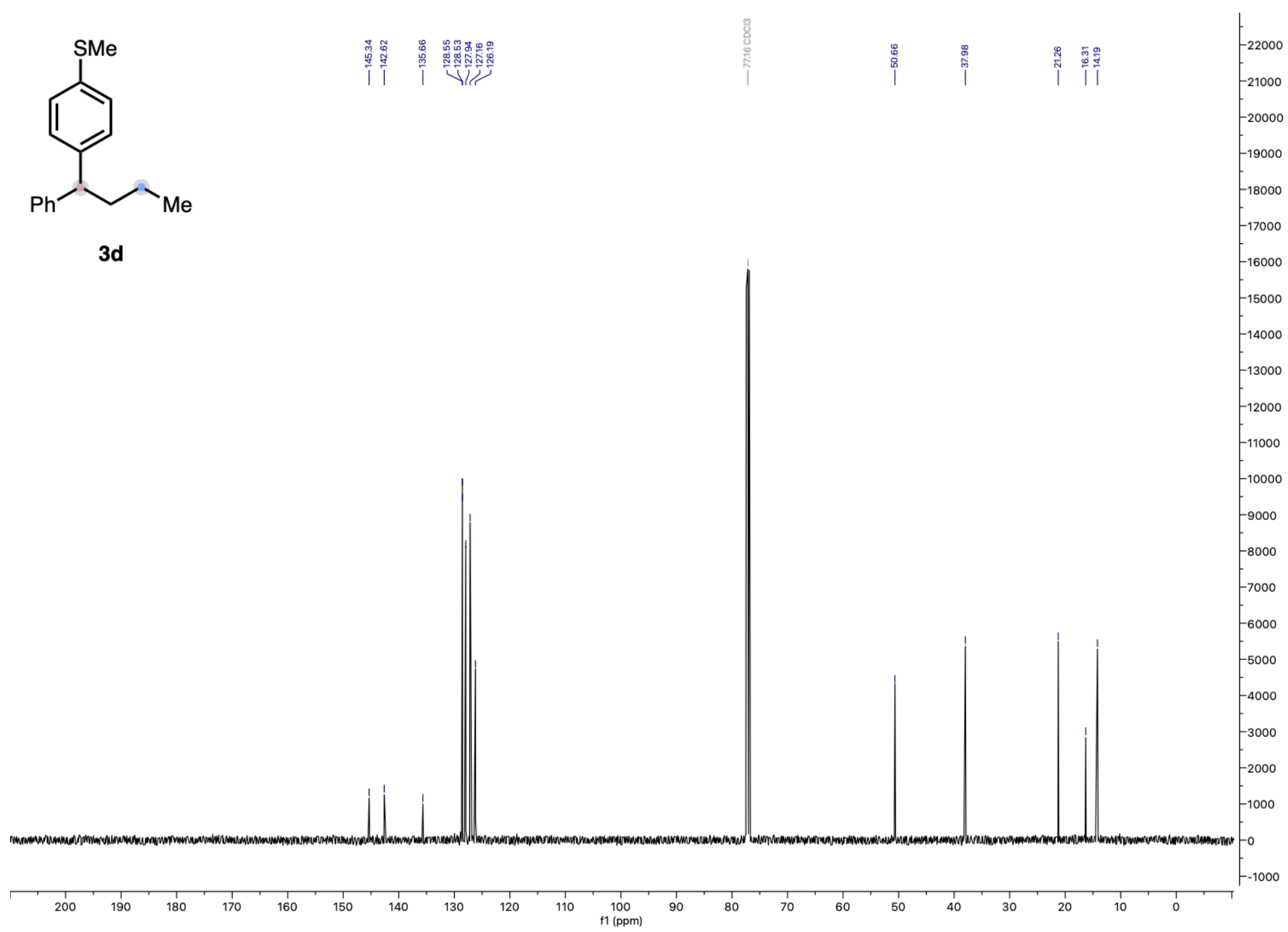

1-(1-Phenylbutyl)-4-(trifluoromethyl)benzene **3e**:

^{1}H NMR (300 MHz, $CDCl_3$):

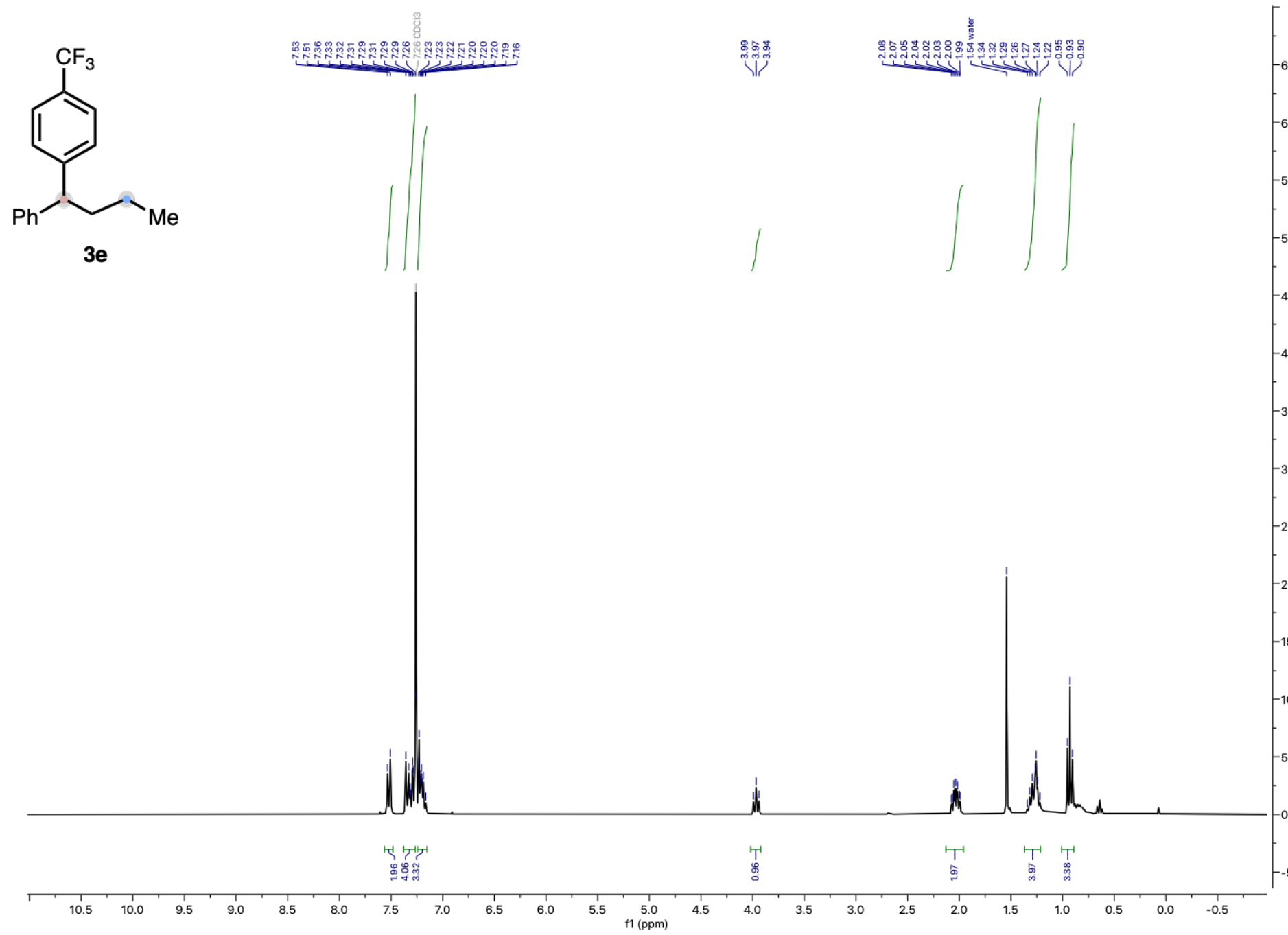

^{13}C NMR (126 MHz, $CDCl_3$):

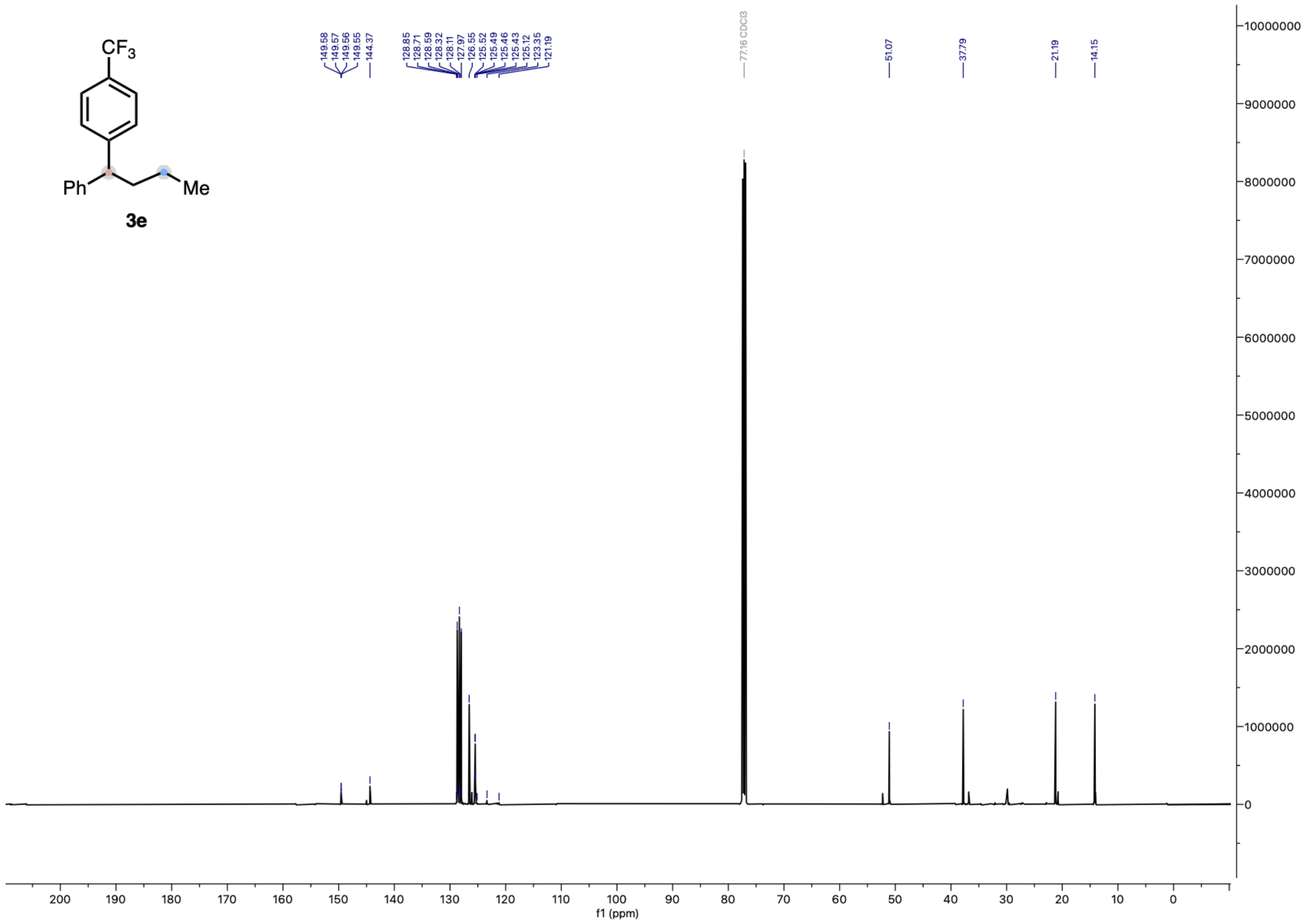

^{19}F NMR (282 MHz, $CDCl_3$):

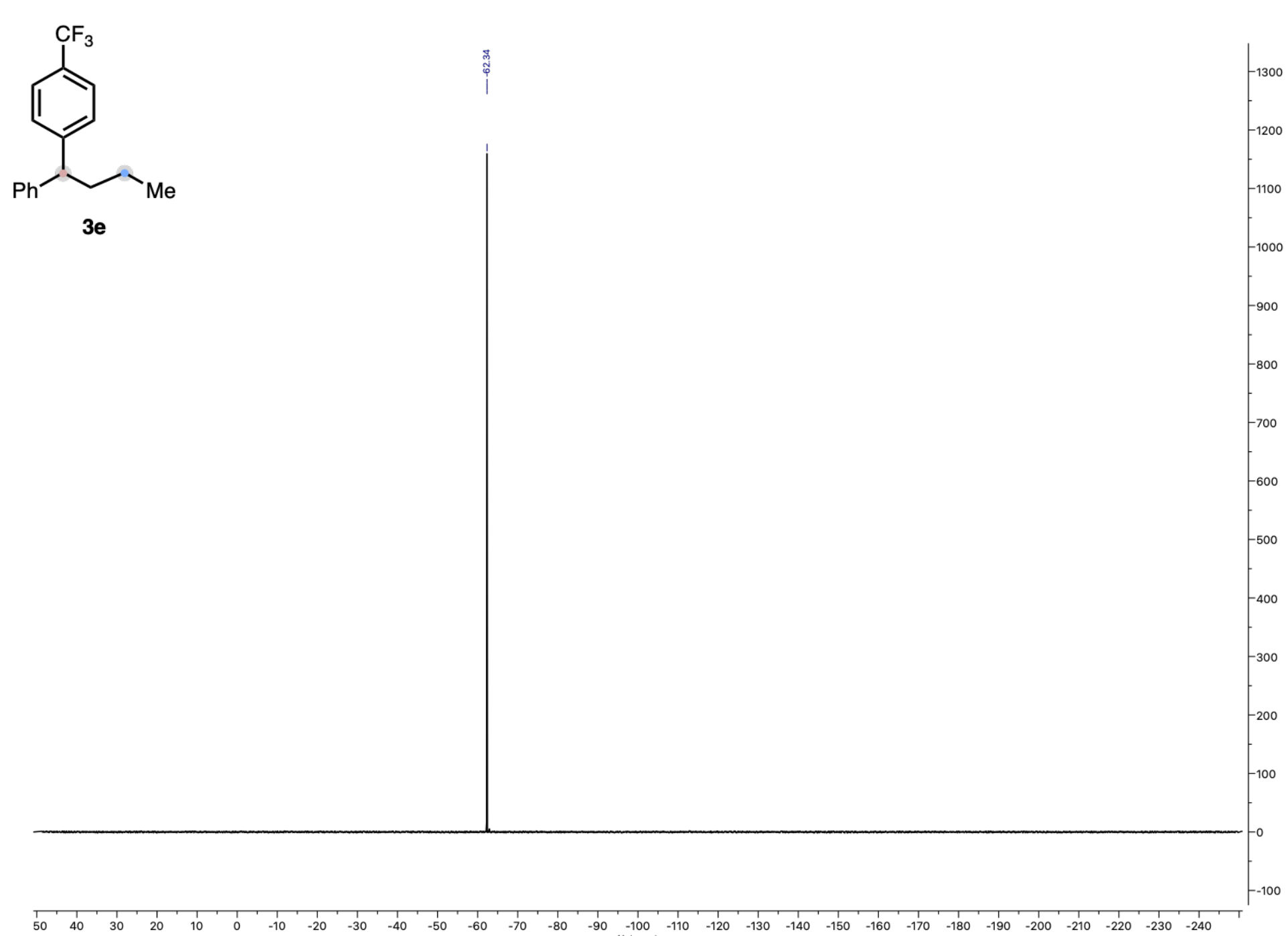

1-Chloro-4-(1-phenylbutyl)benzene **3f**:

^{1}H NMR (500 MHz, $CDCl_3$):

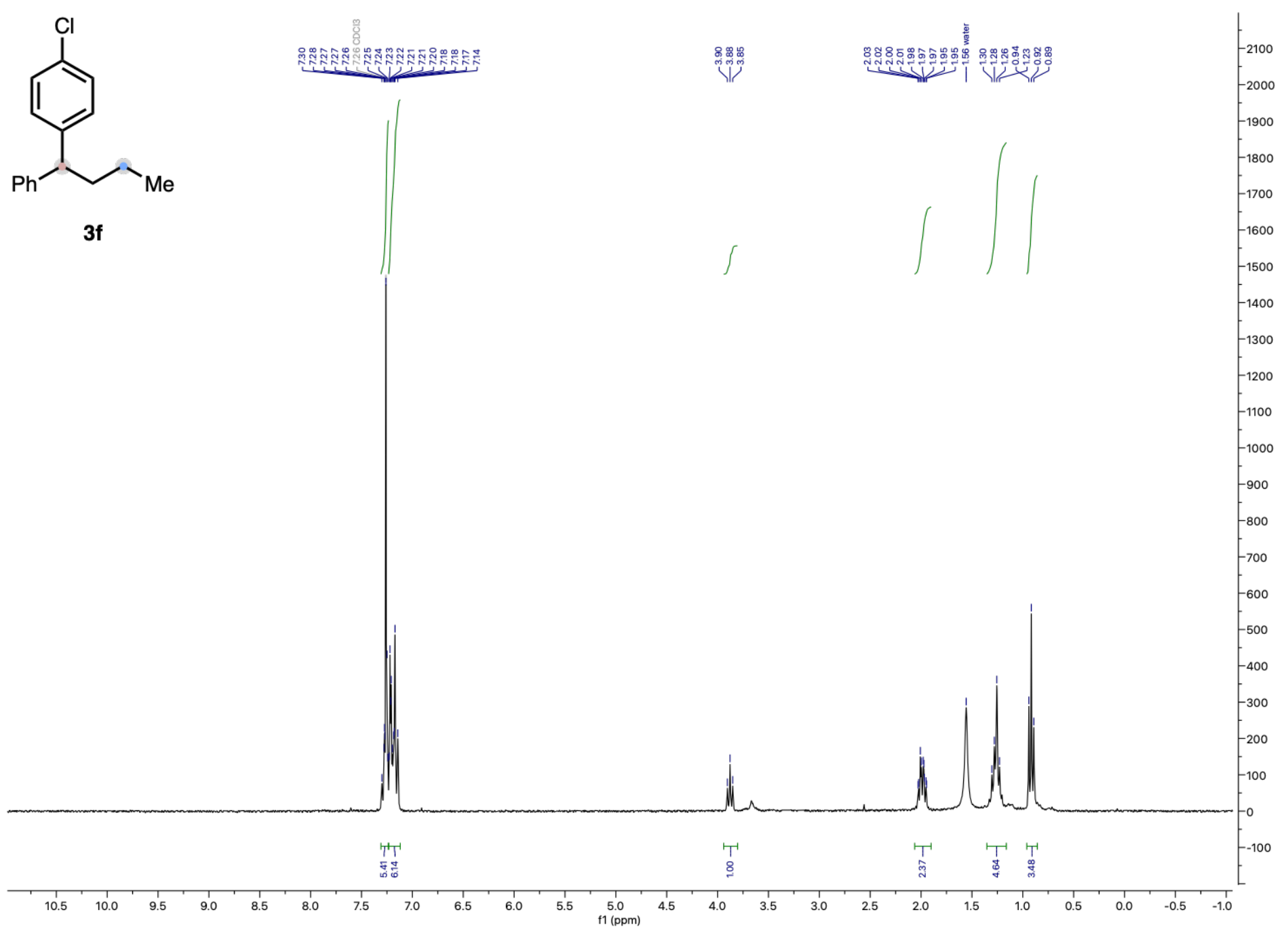

^{13}C NMR (126 MHz, $CDCl_3$):

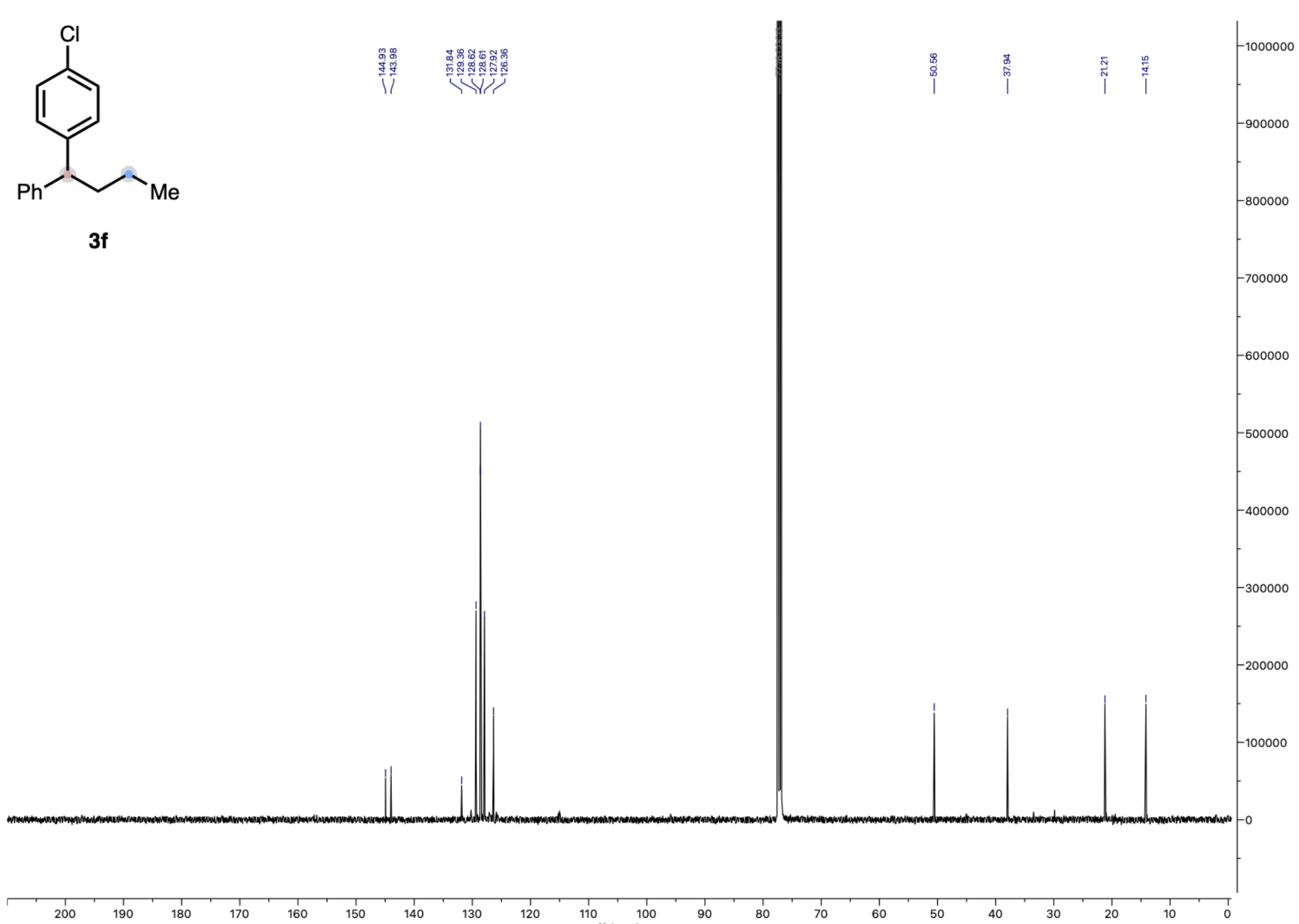

4-(1-Phenylbutyl)benzaldehyde **3g**:

^{1}H NMR (500 MHz, $CDCl_3$):

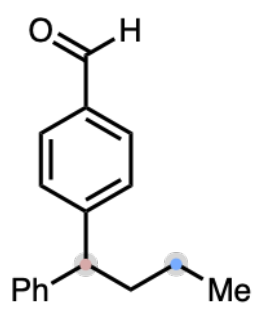

3g

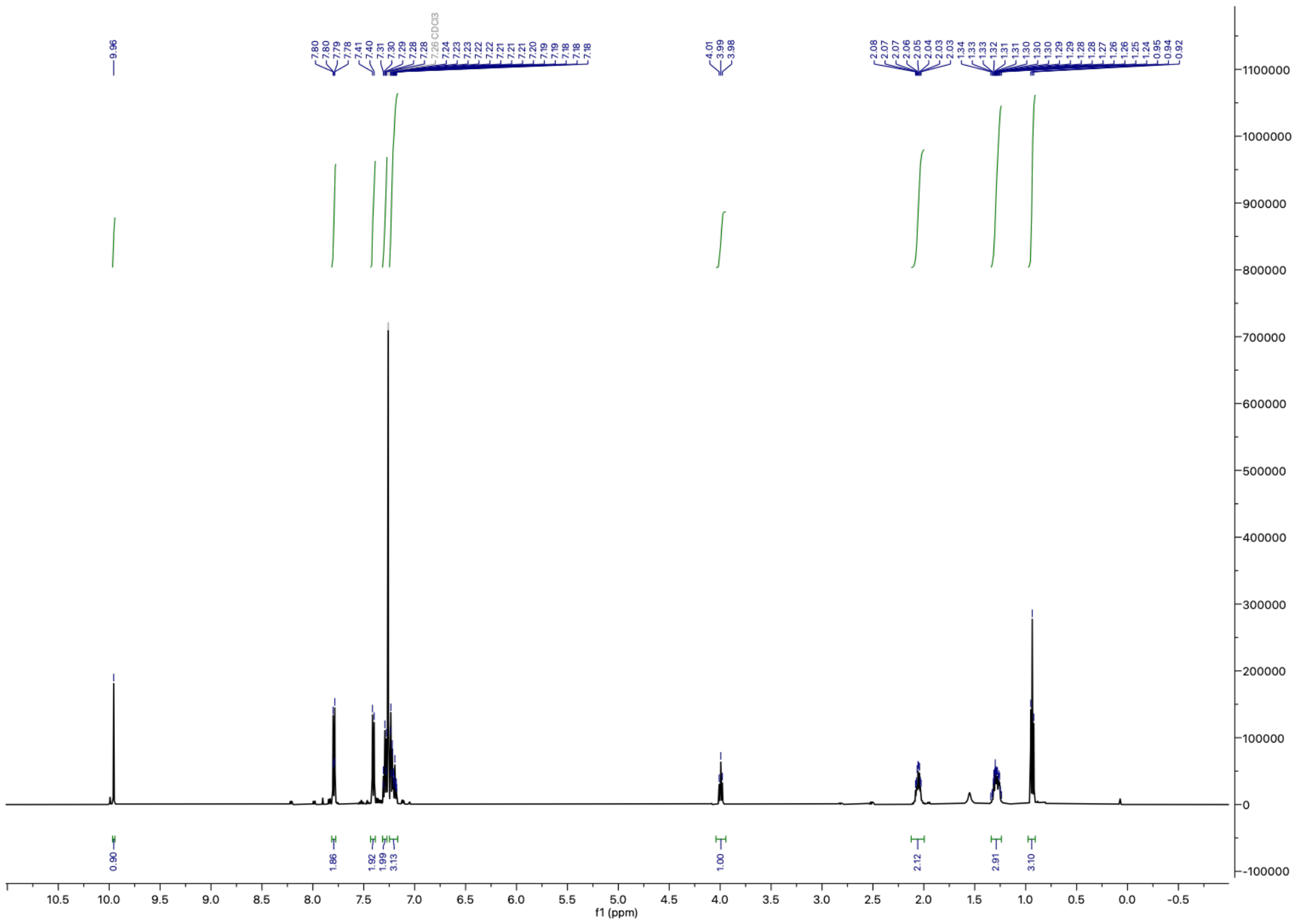

^{13}C NMR (126 MHz, $CDCl_3$):

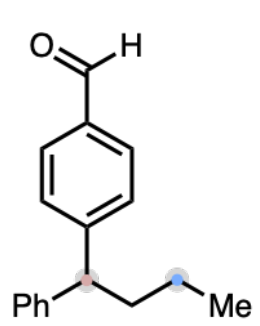

3g

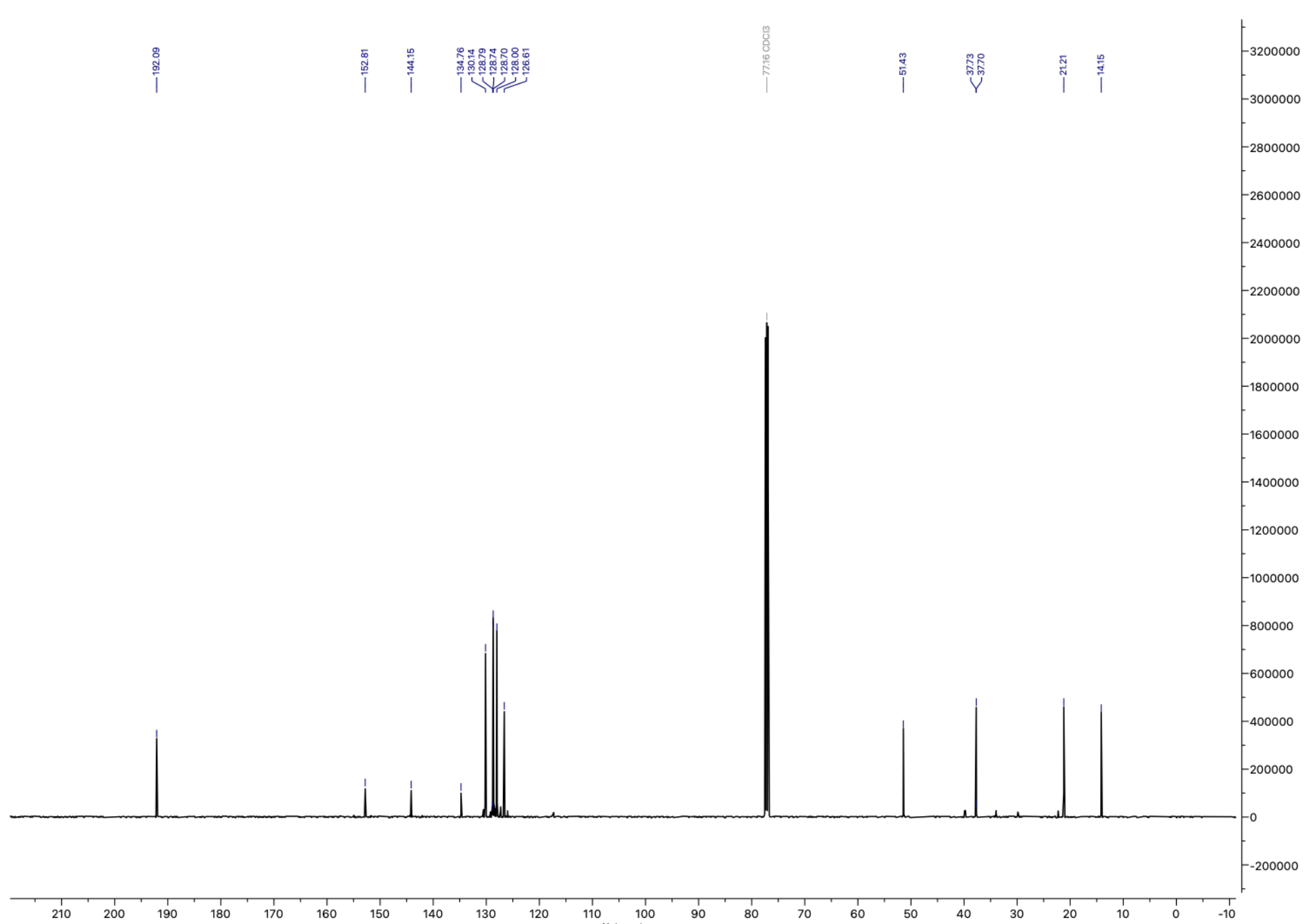

1-(4-(1-Phenylbutyl) phenyl)ethan-1-one **3h**:

^{1}H NMR (500 MHz, $CDCl_3$):

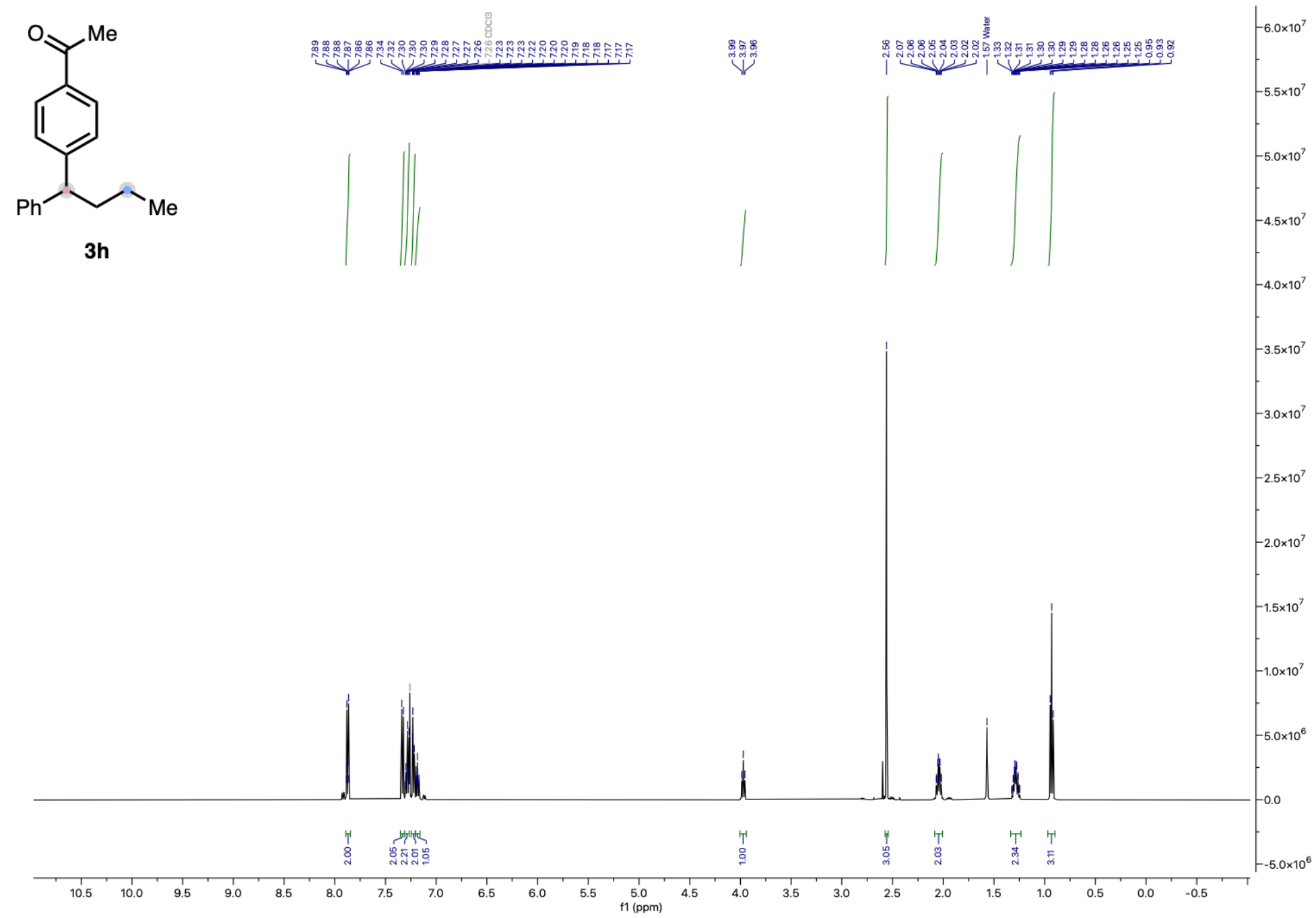

^{13}C NMR (126 MHz, $CDCl_3$):

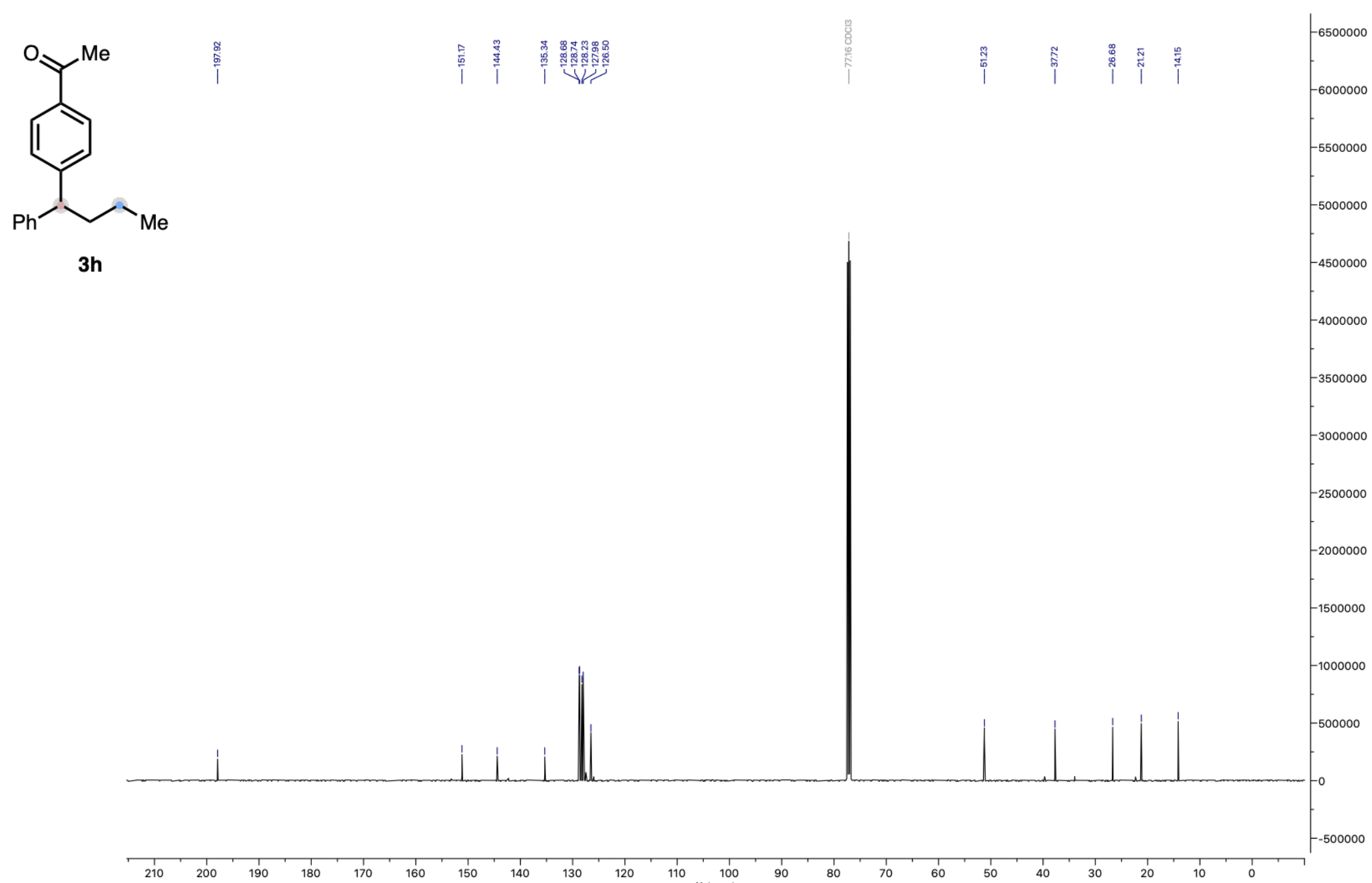

Methyl 4-(1-phenylbutyl)benzoate **3i**:

^{1}H NMR (500 MHz; $CDCl_3$):

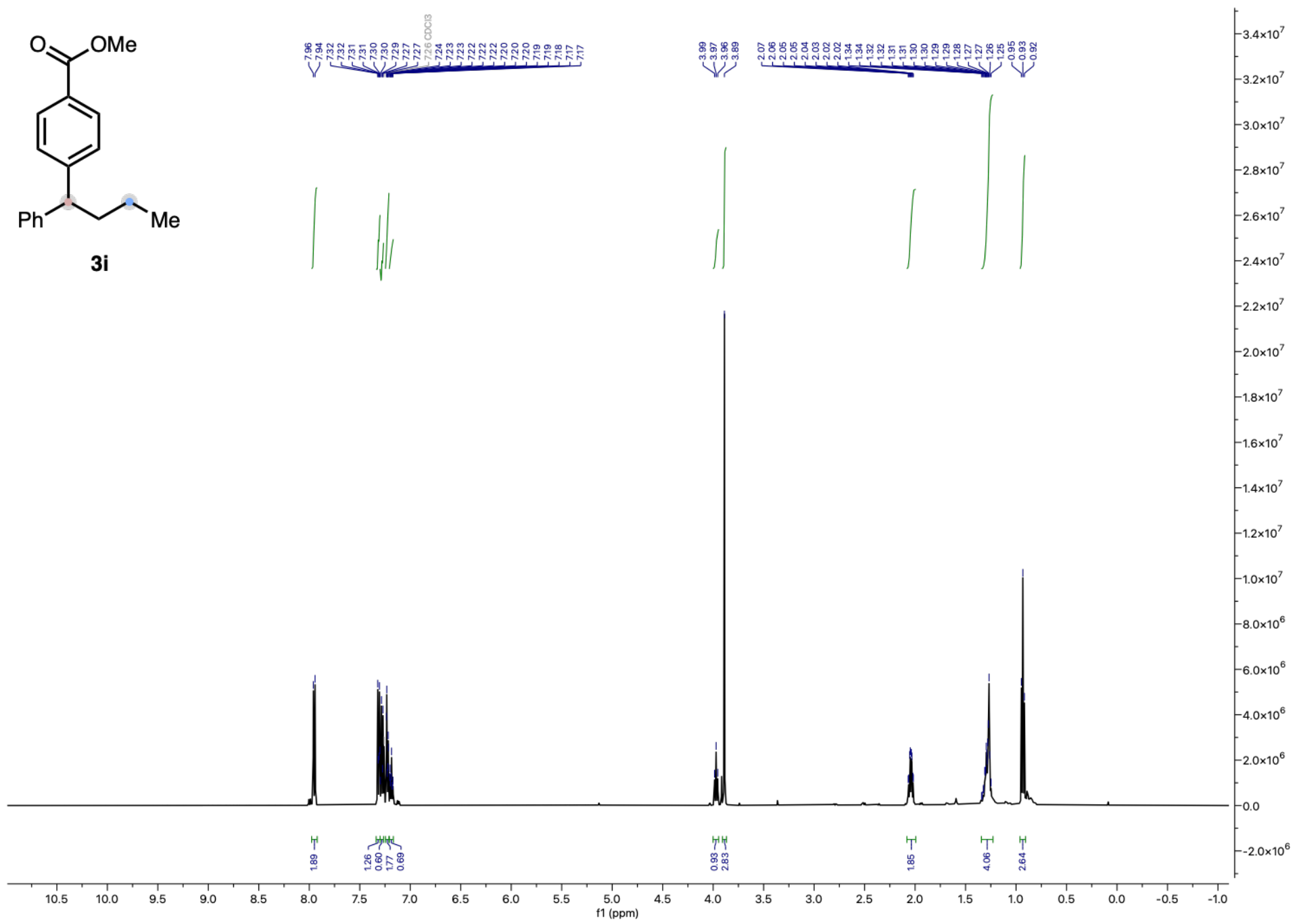

^{13}C NMR (126 MHz; $CDCl_3$):

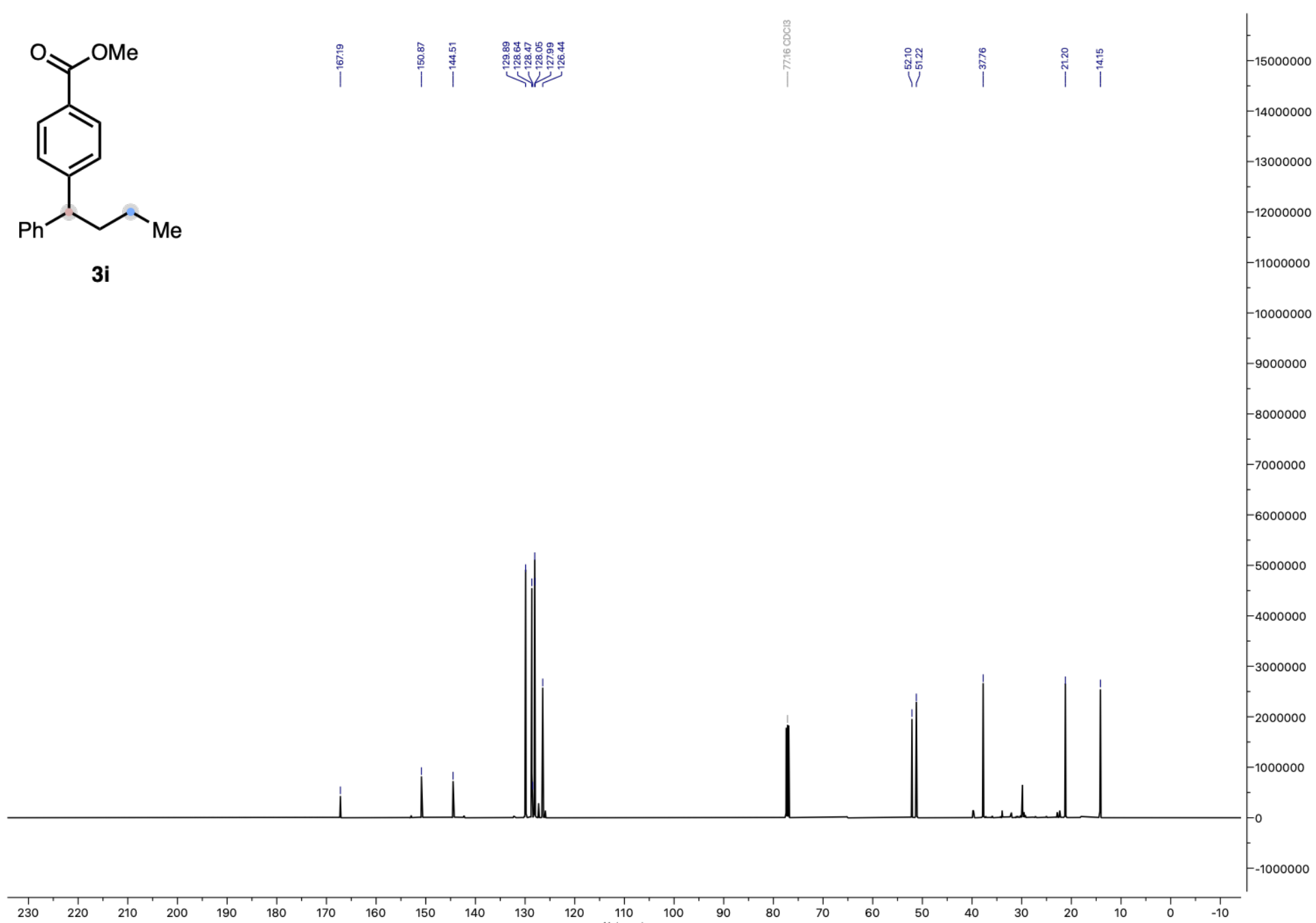

N,*N*-Dimethyl-4-(1-phenylbutyl)benzenesulfonamide **3j**:

^{1}H NMR (500 MHz; $CDCl_3$):

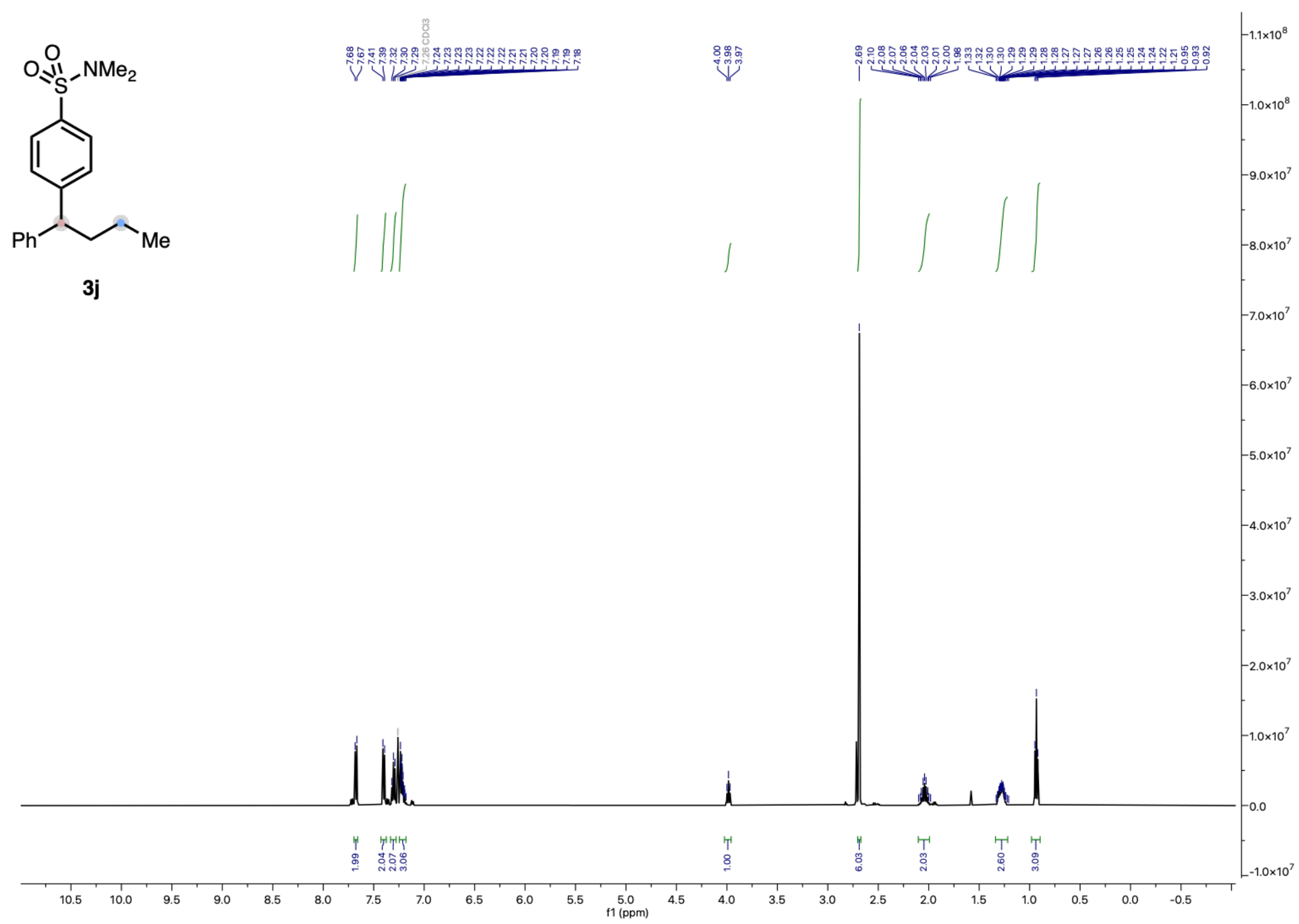

^{13}C NMR (126 MHz; $CDCl_3$):

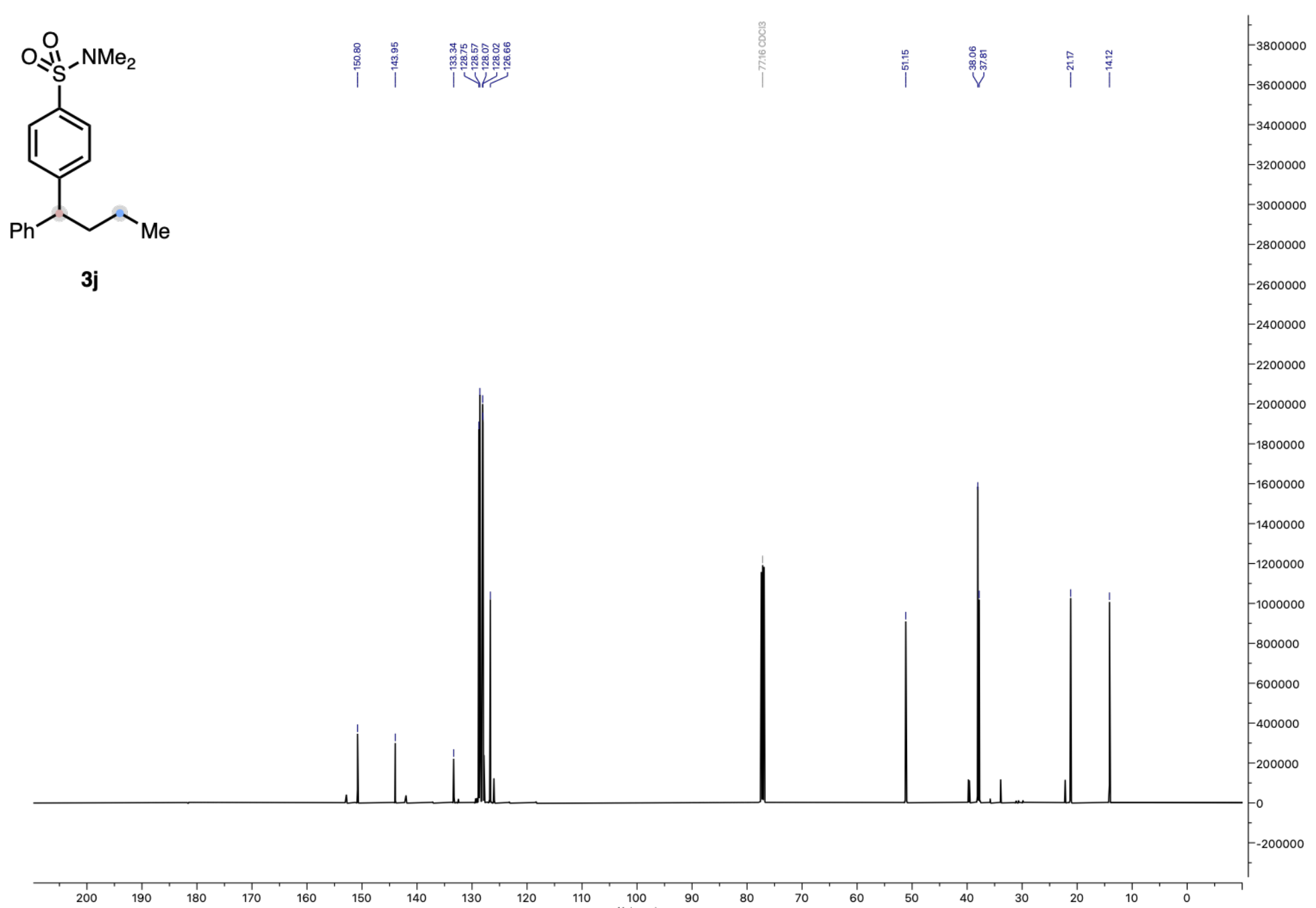

(4-(1-Phenylbutyl)phenyl)methanol **3k**:

^{1}H NMR (500 MHz, $CDCl_3$):

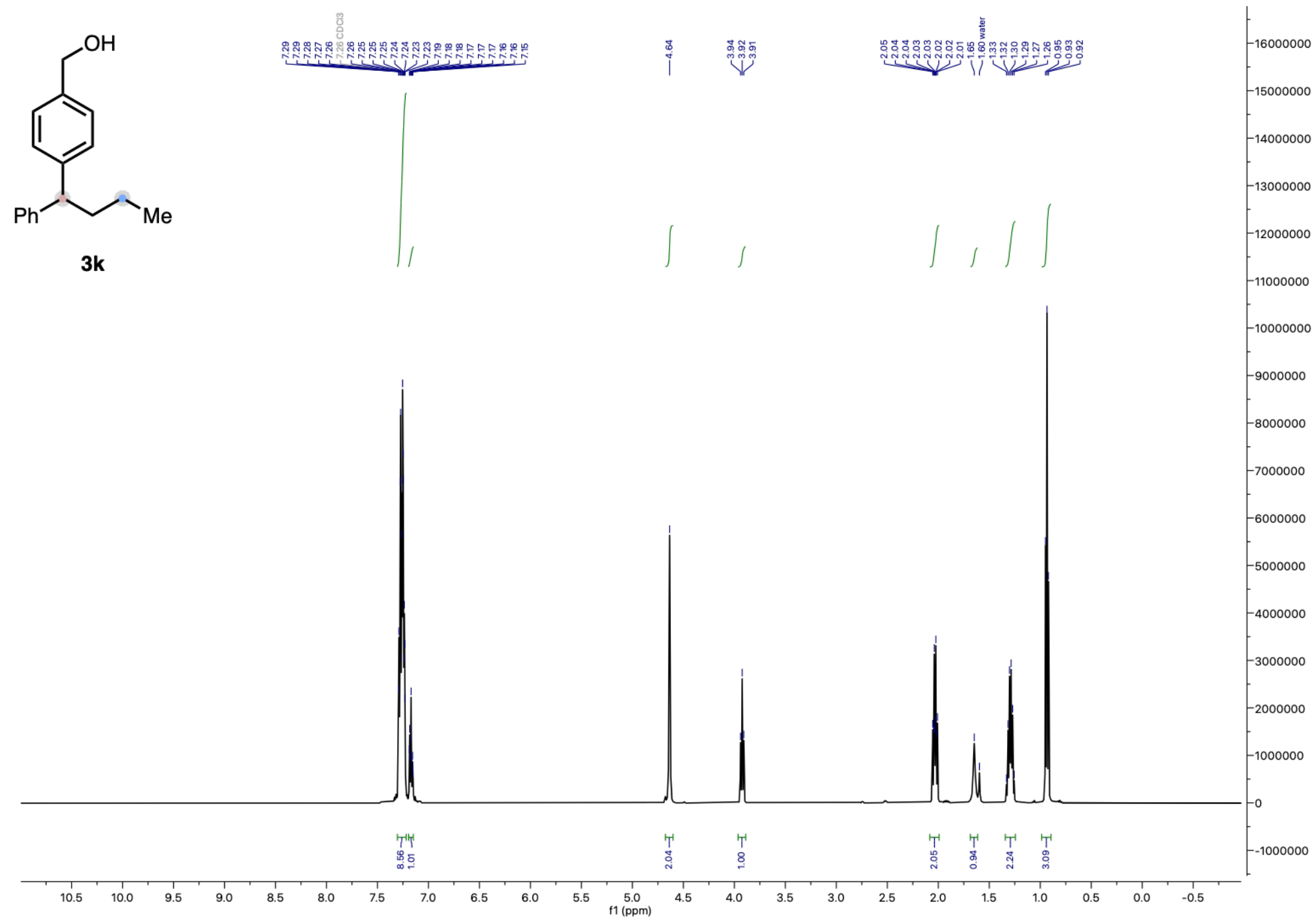

^{13}C NMR (126 MHz, $CDCl_3$):

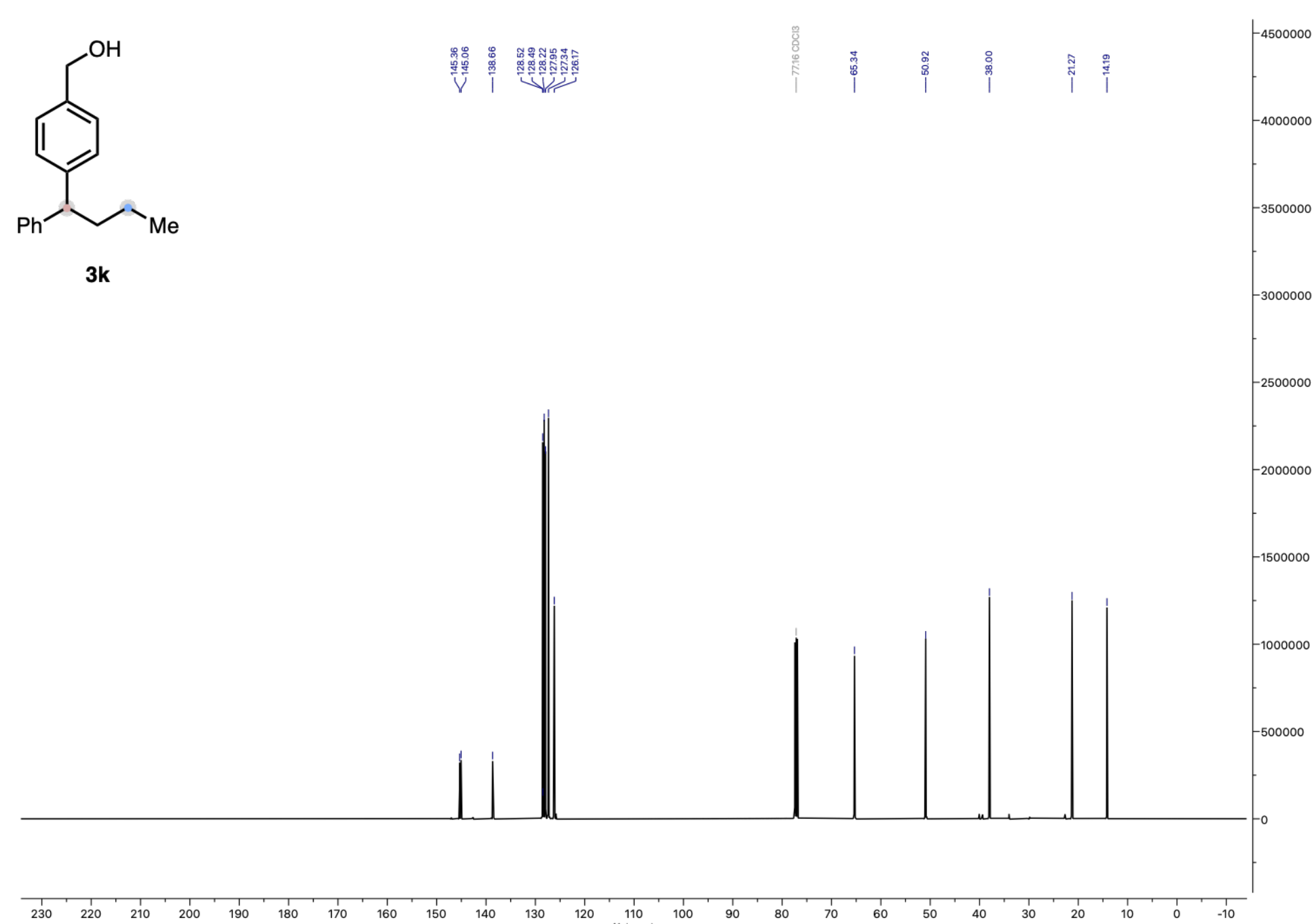

4-(1-Phenylbutyl)phenol **3l**:

^{1}H NMR (500 MHz; CDCl$_3$):

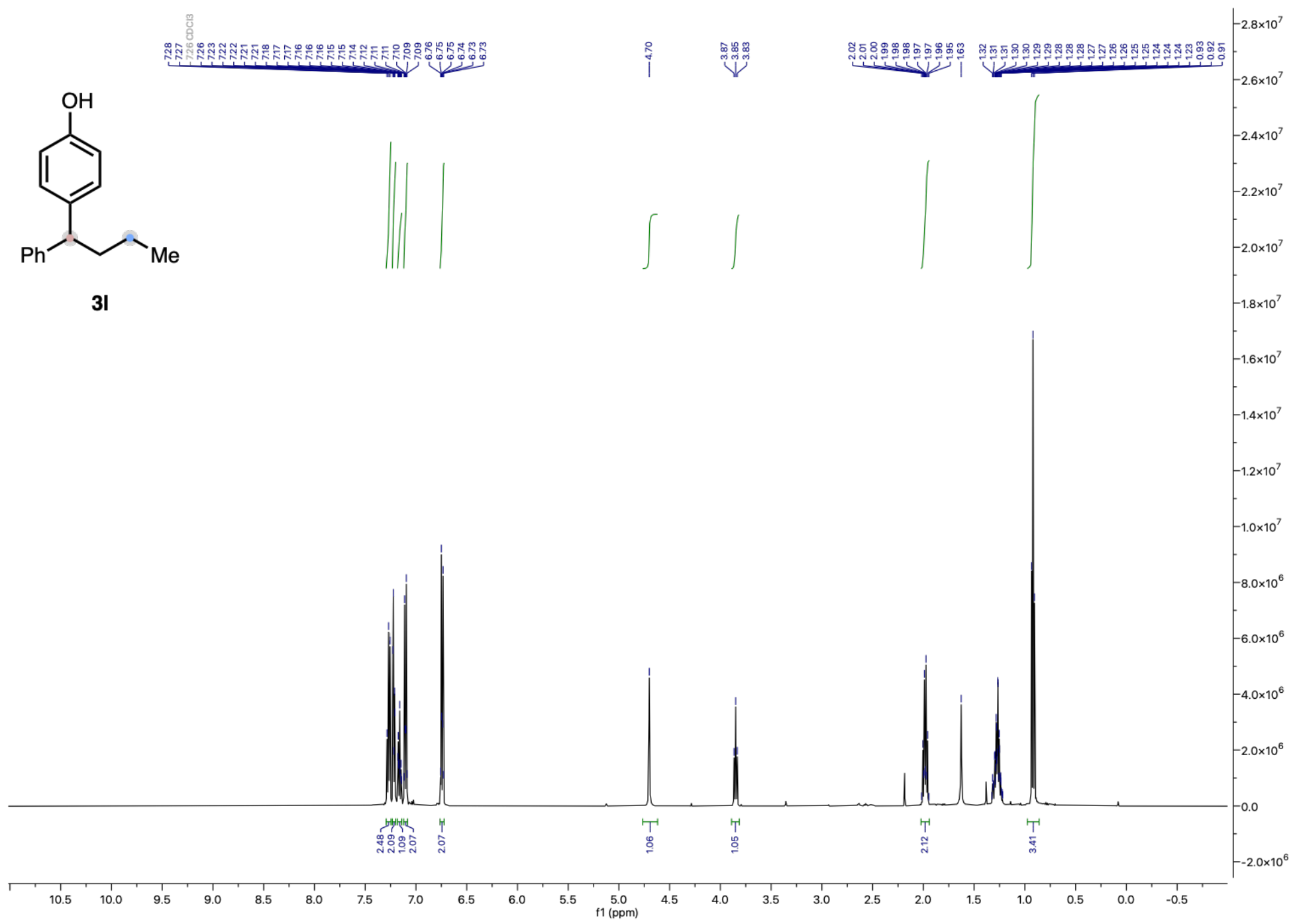

^{13}C NMR (126 MHz, CDCl$_3$):

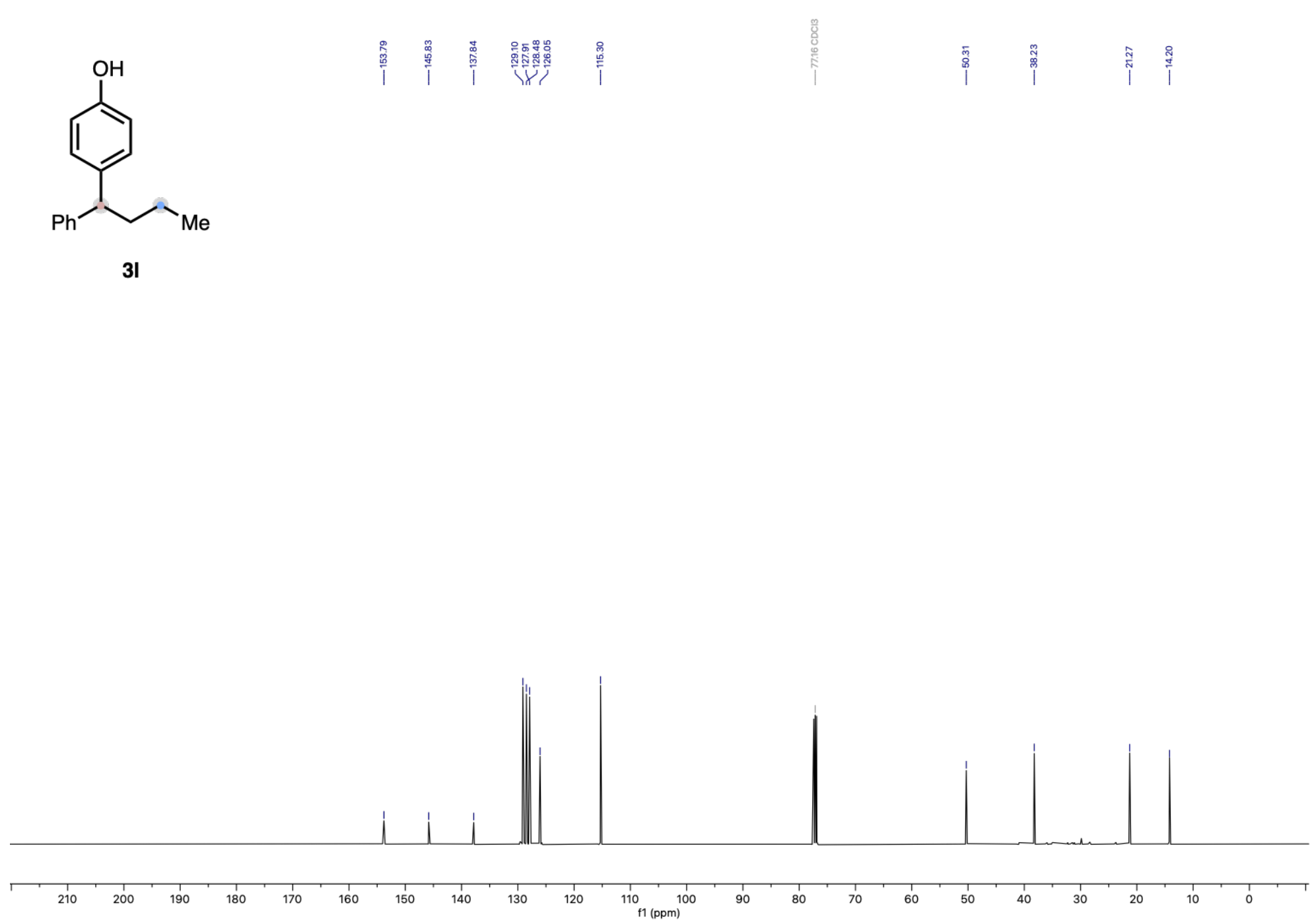

4-(1-Phenylbutyl)aniline **3m**:

^1H NMR (500 MHz; $CDCl_3$):

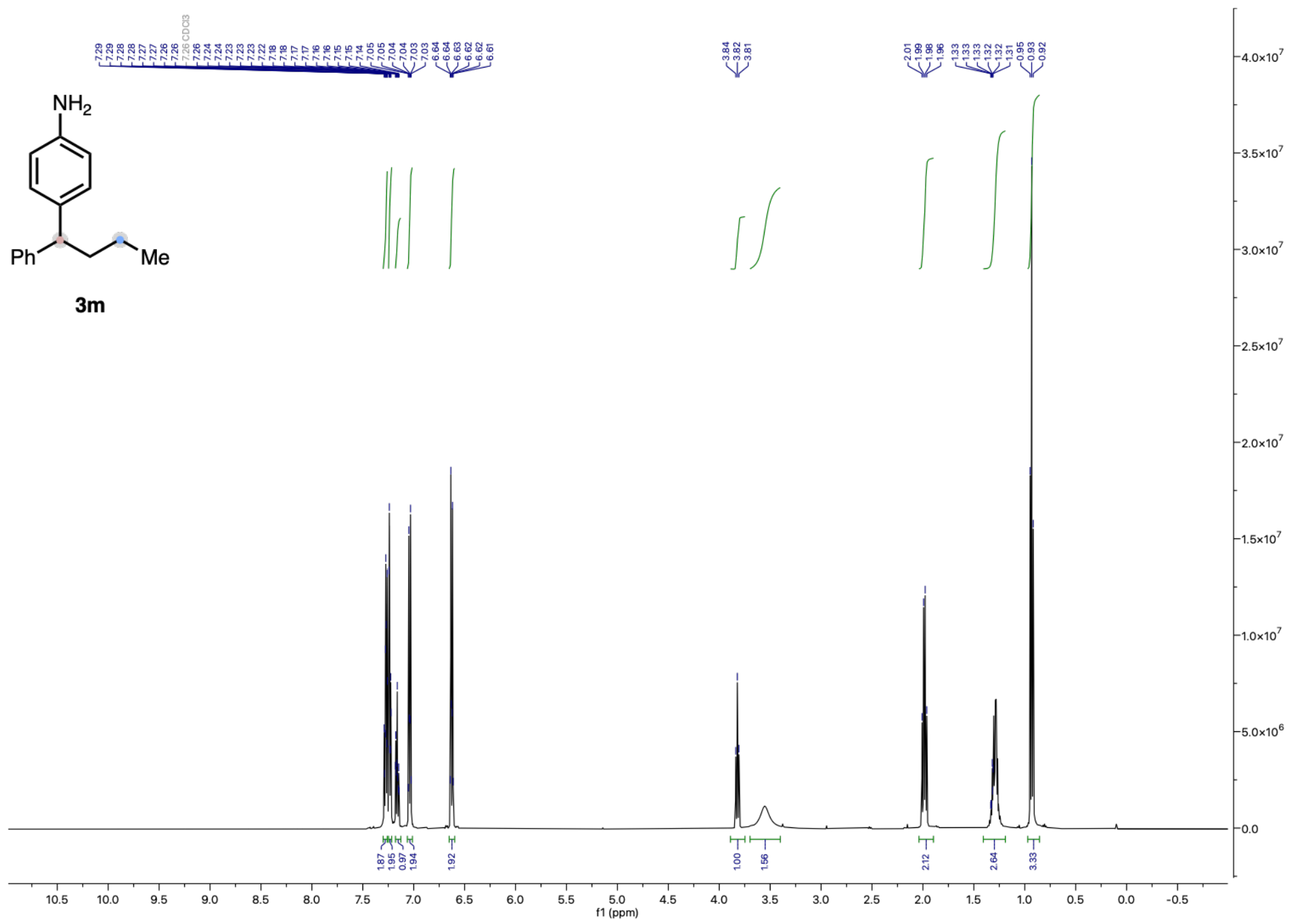

^{13}C NMR (126 MHz, $CDCl_3$):

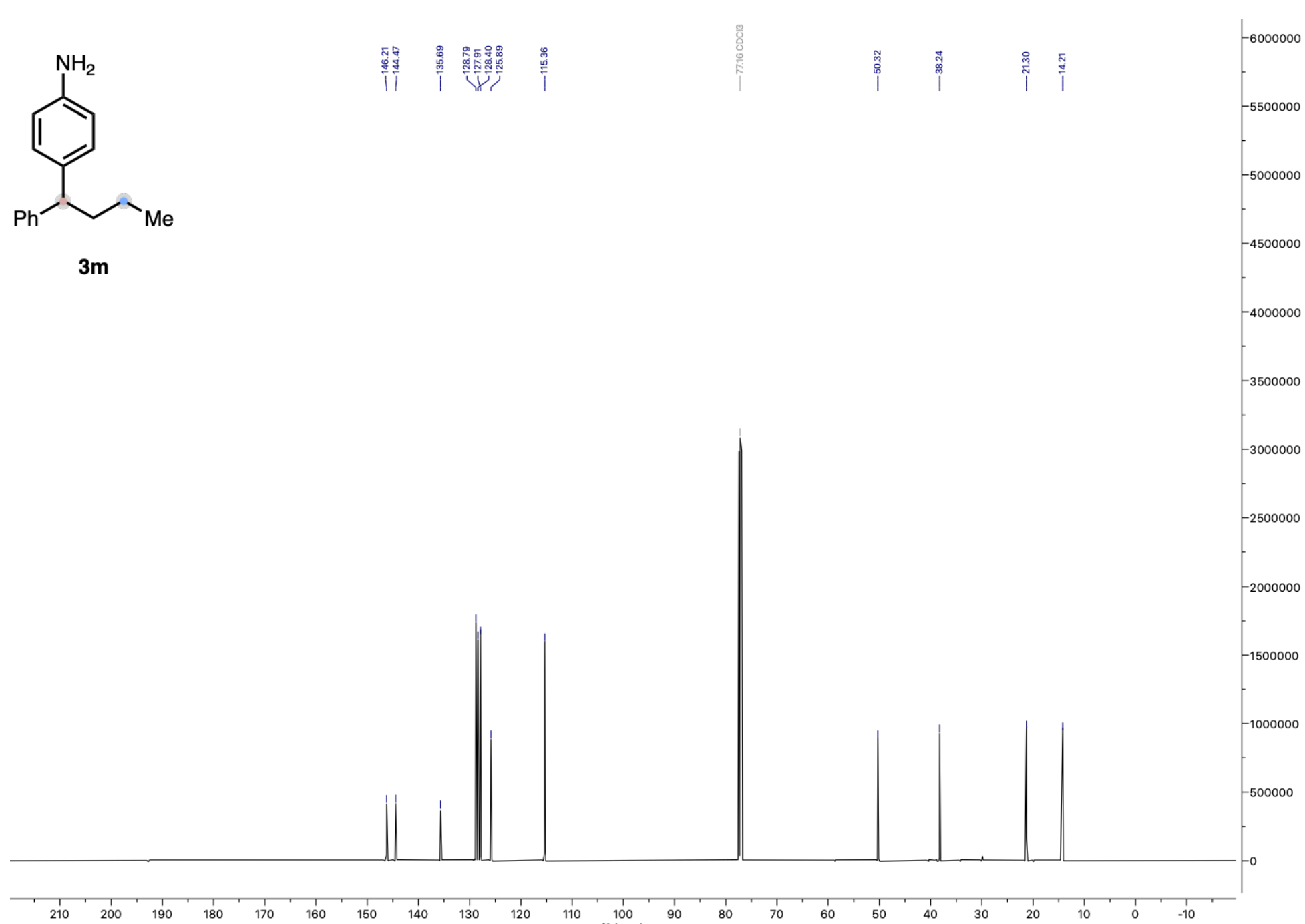

1-Methoxy-3-(1-phenylbutyl)benzene **3n**:

^{1}H NMR (300 MHz; $CDCl_3$):

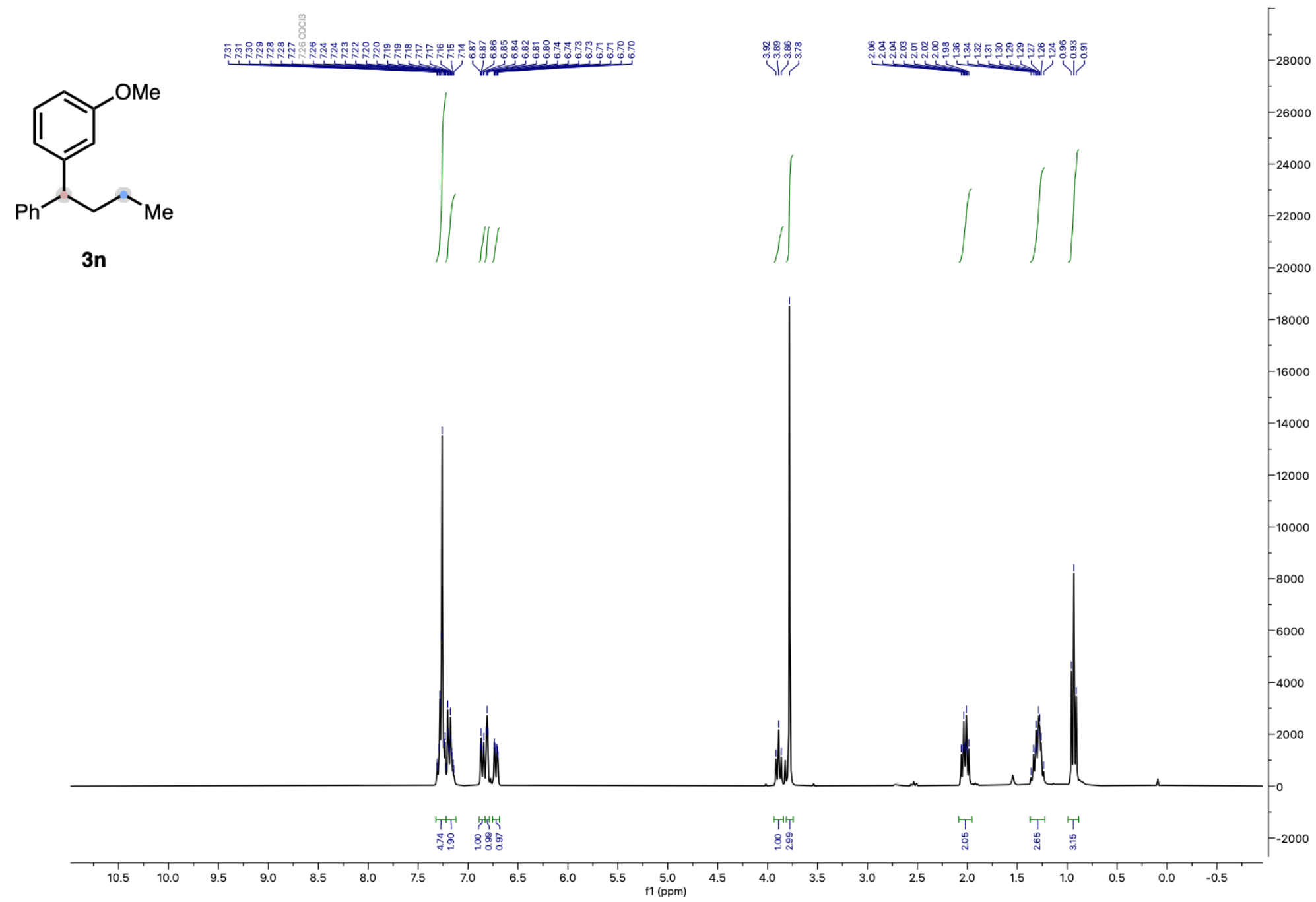

^{13}C NMR (126 MHz, $CDCl_3$):

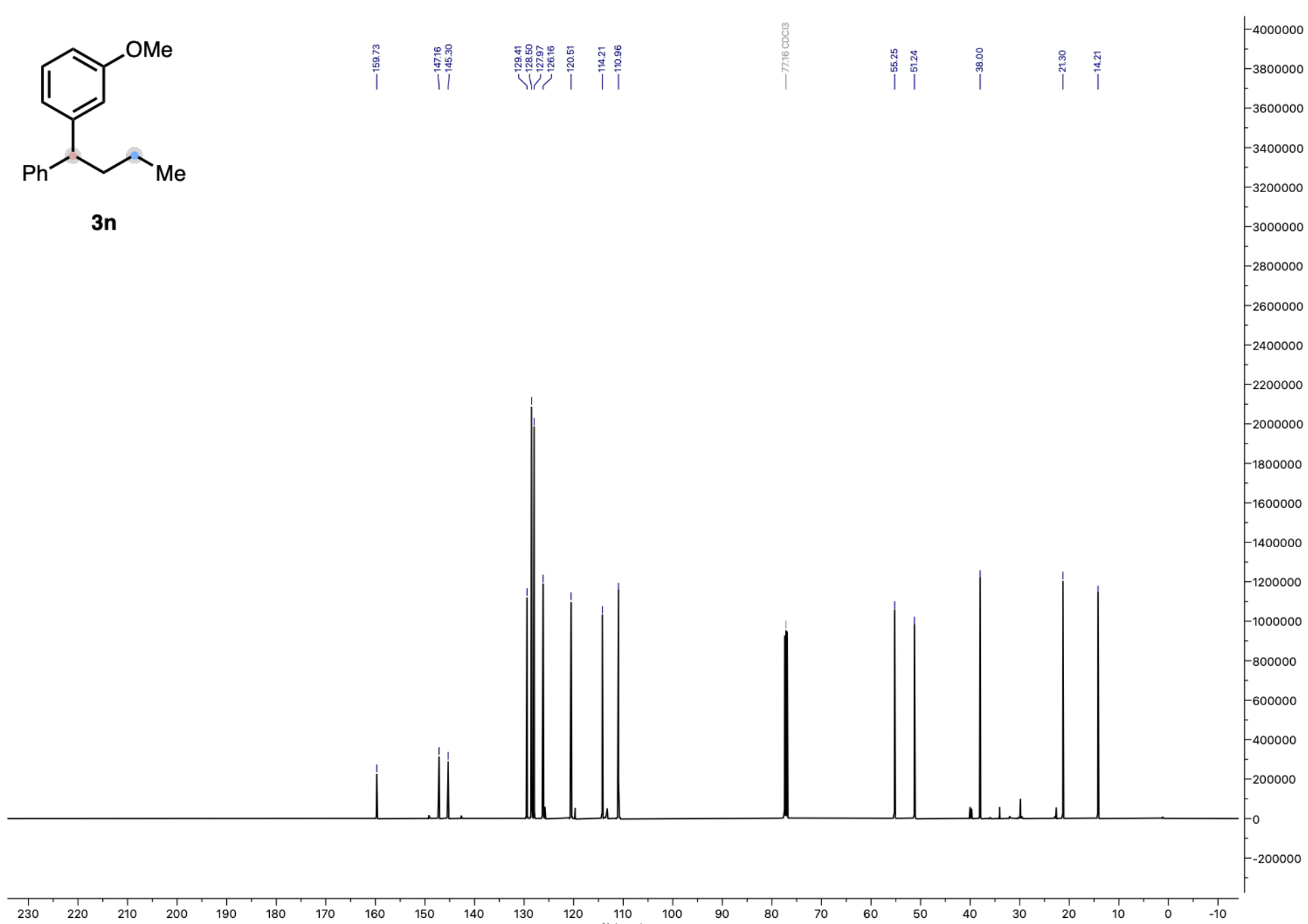

1-Methoxy-2-(1-phenylbutyl)benzene **3o**:

^{1}H NMR (300 MHz; $CDCl_3$):

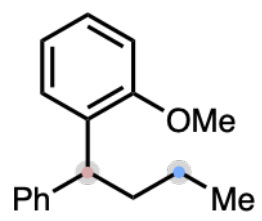

3o

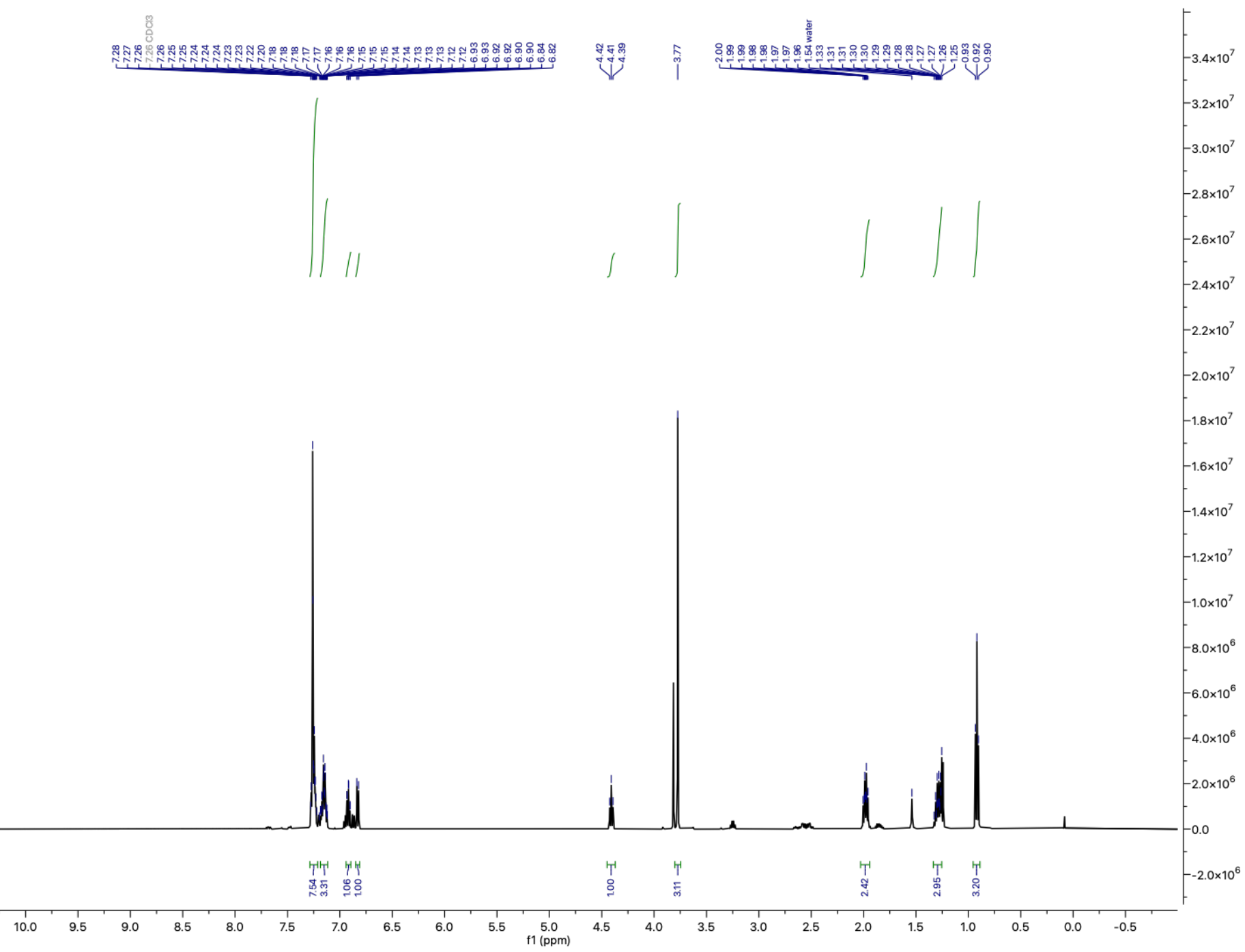

^{13}C NMR (126 MHz, $CDCl_3$):

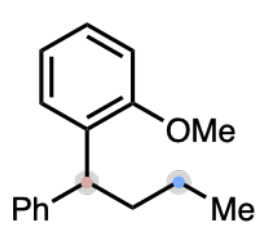

3o

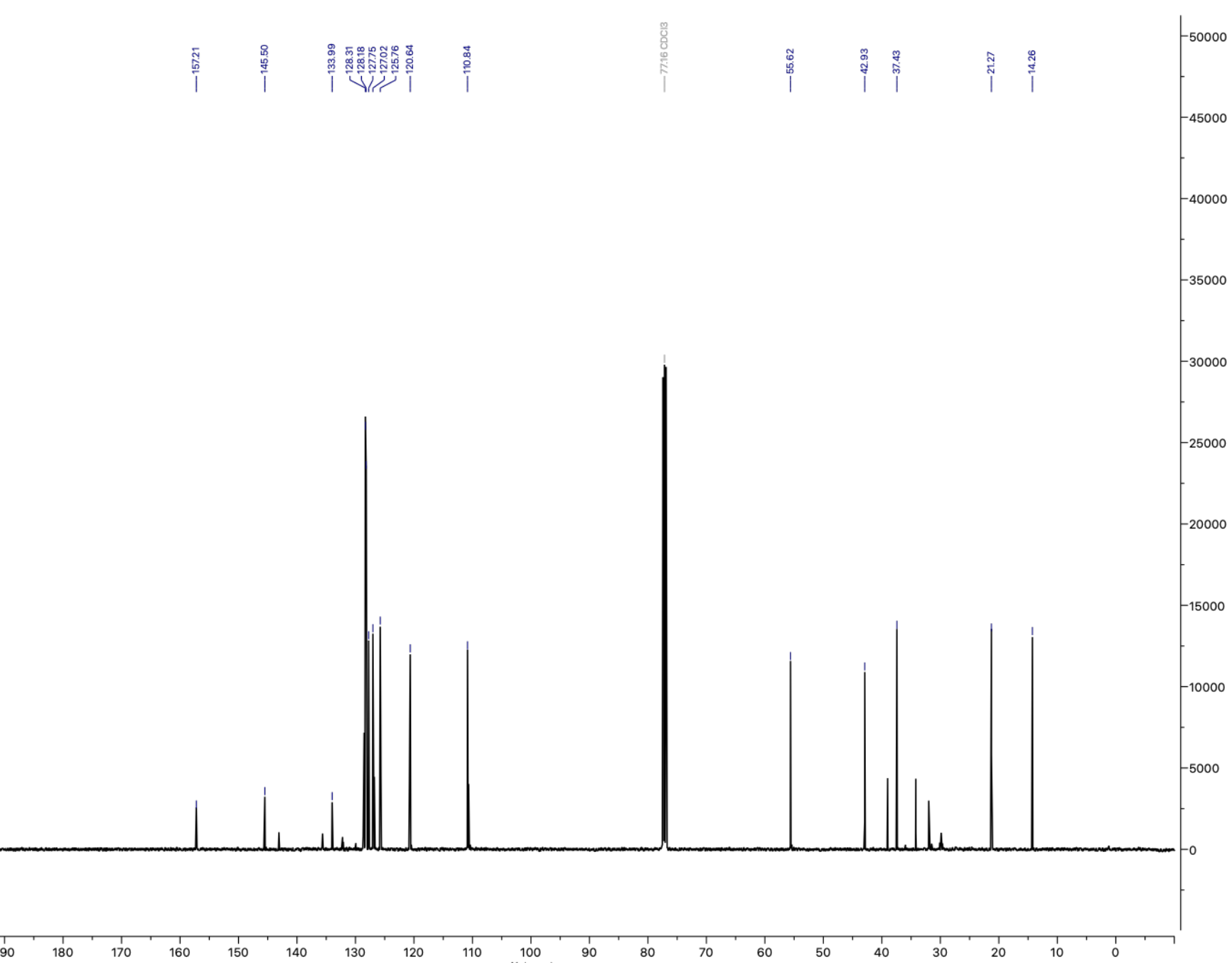

tert-Butyl 5-(1-phenylbutyl)-1*H*-indole-1-carboxylate **3p**:

^{1}H NMR (400 MHz; CDCl$_3$):

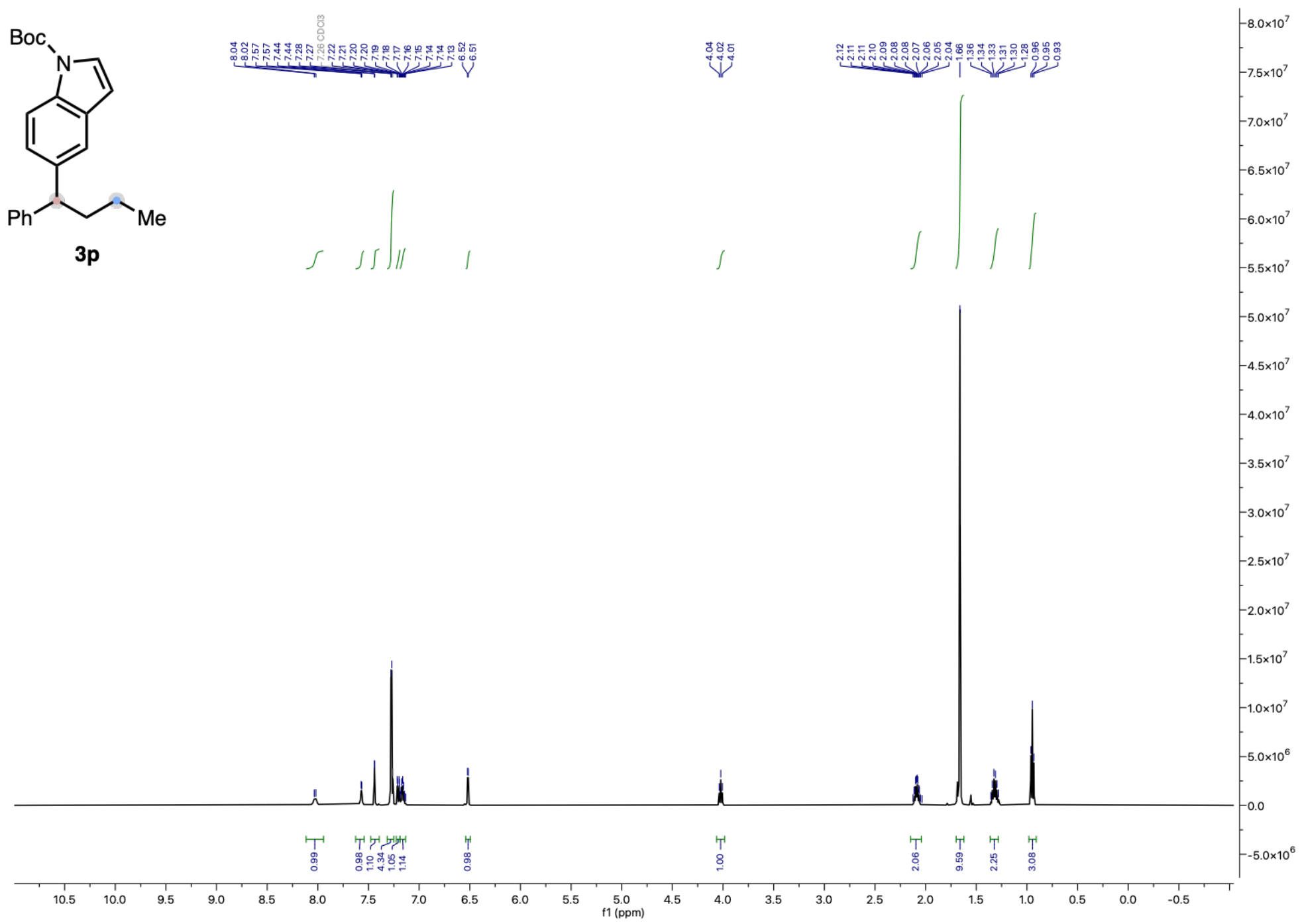

^{13}C NMR (126 MHz; CDCl$_3$):

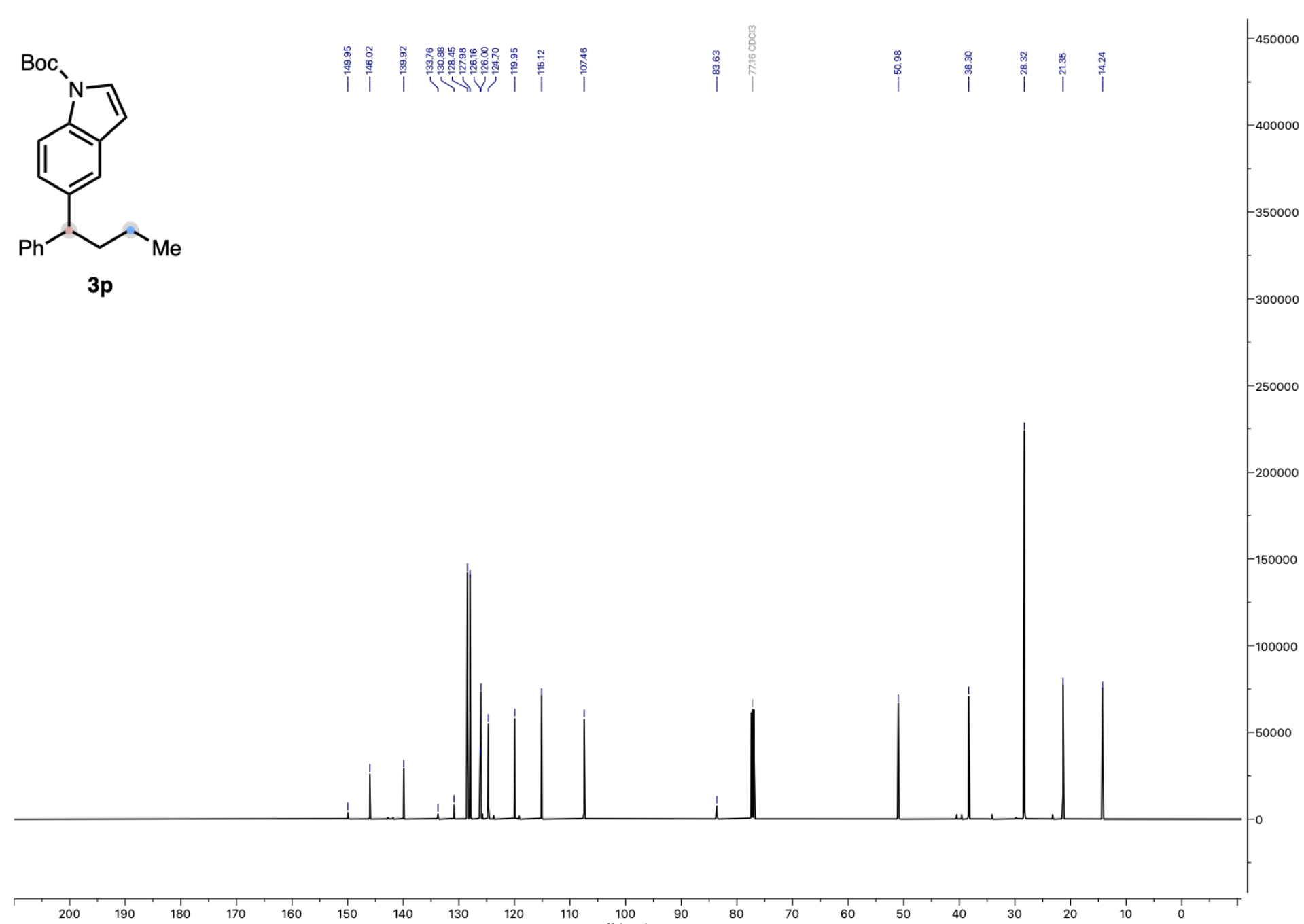

5-(1-Phenylbutyl)-1*H*-indole **3q**:

^{1}H NMR (500 MHz; $CDCl_3$):

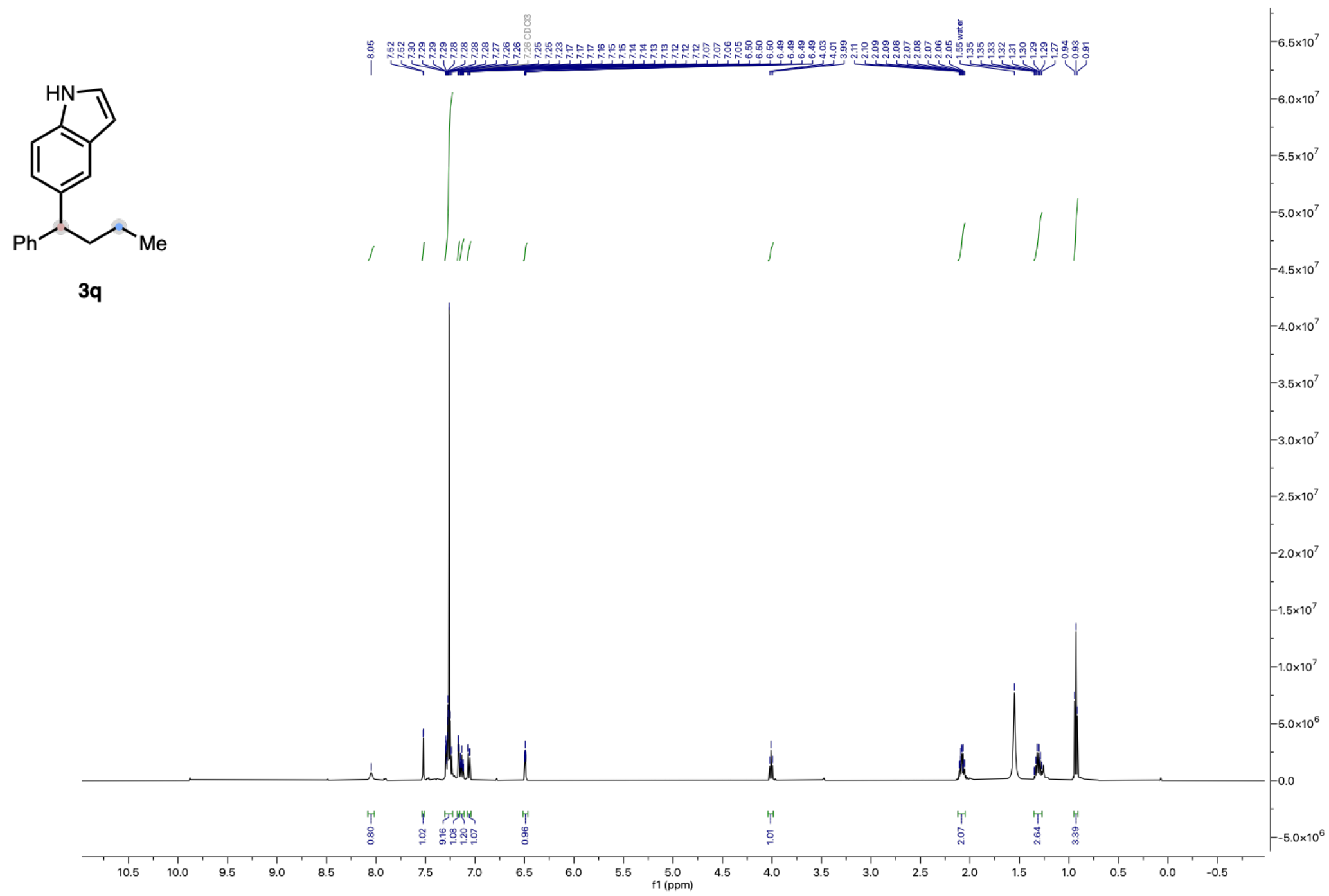

^{13}C NMR (126 MHz; $CDCl_3$):

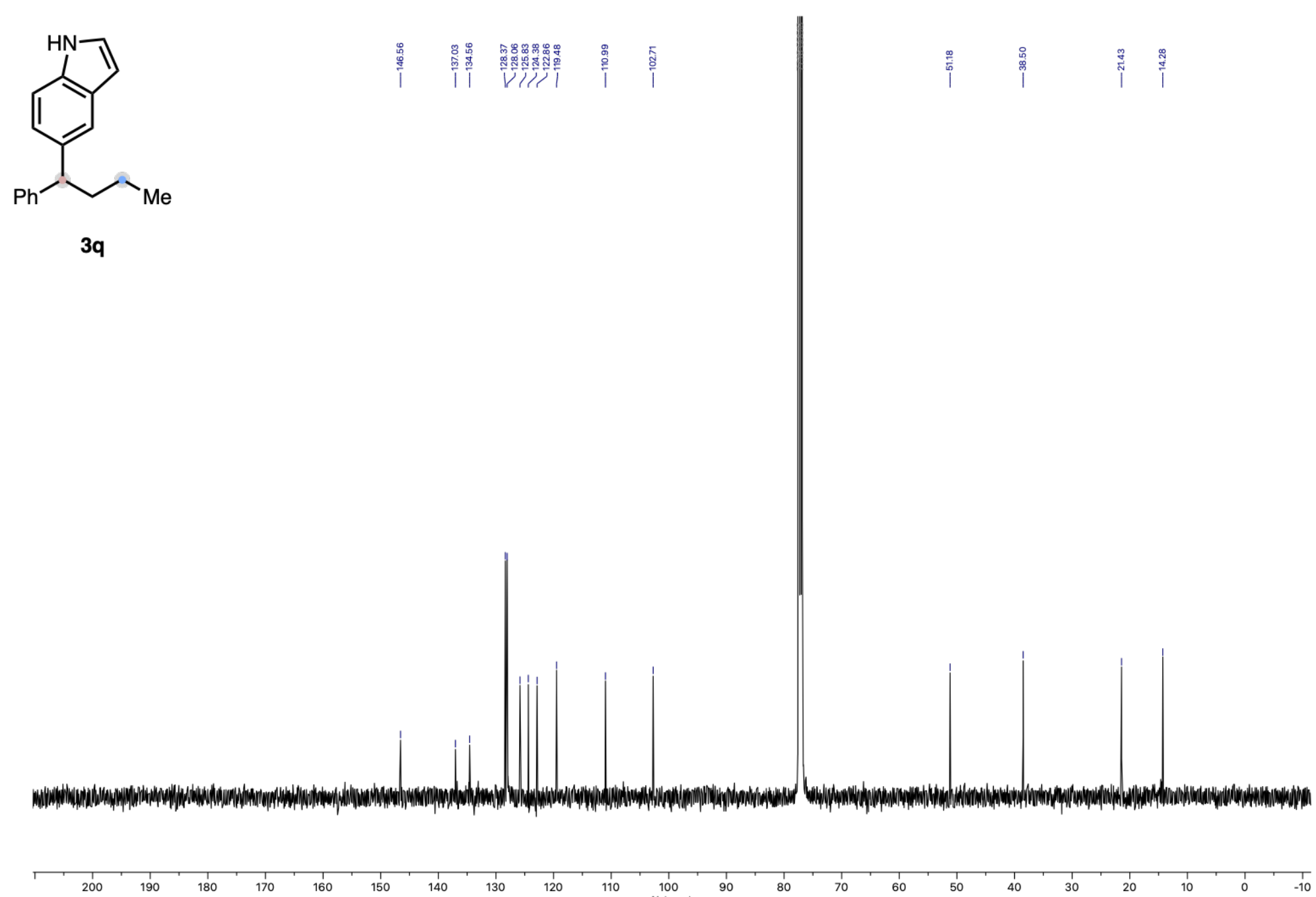

N,*N*-Dimethyl-5-(1-phenylbutyl)pyridin-2-amine **3r**:

^{1}H NMR (500 MHz; $CDCl_3$):

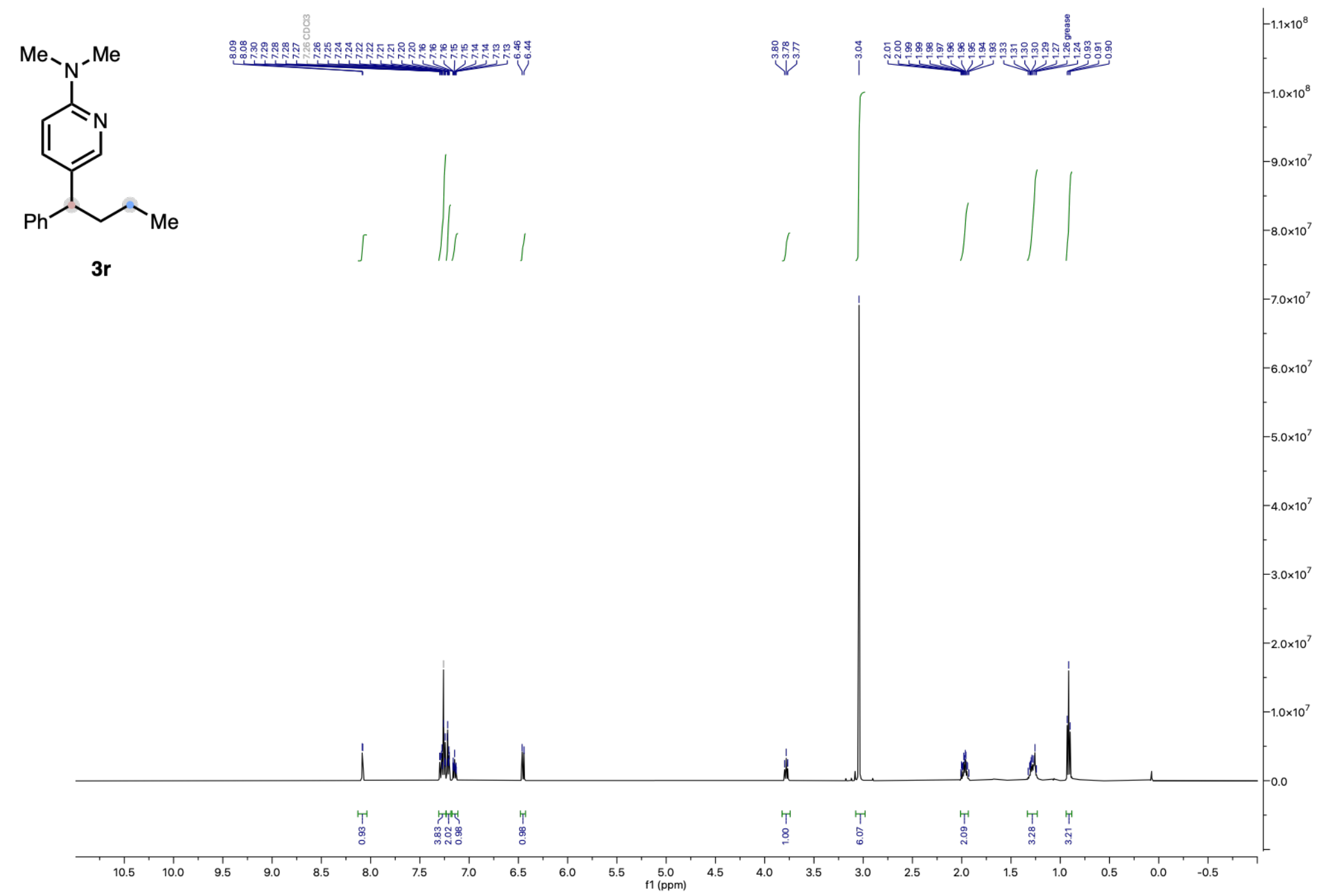

^{13}C NMR (126 MHz; $CDCl_3$):

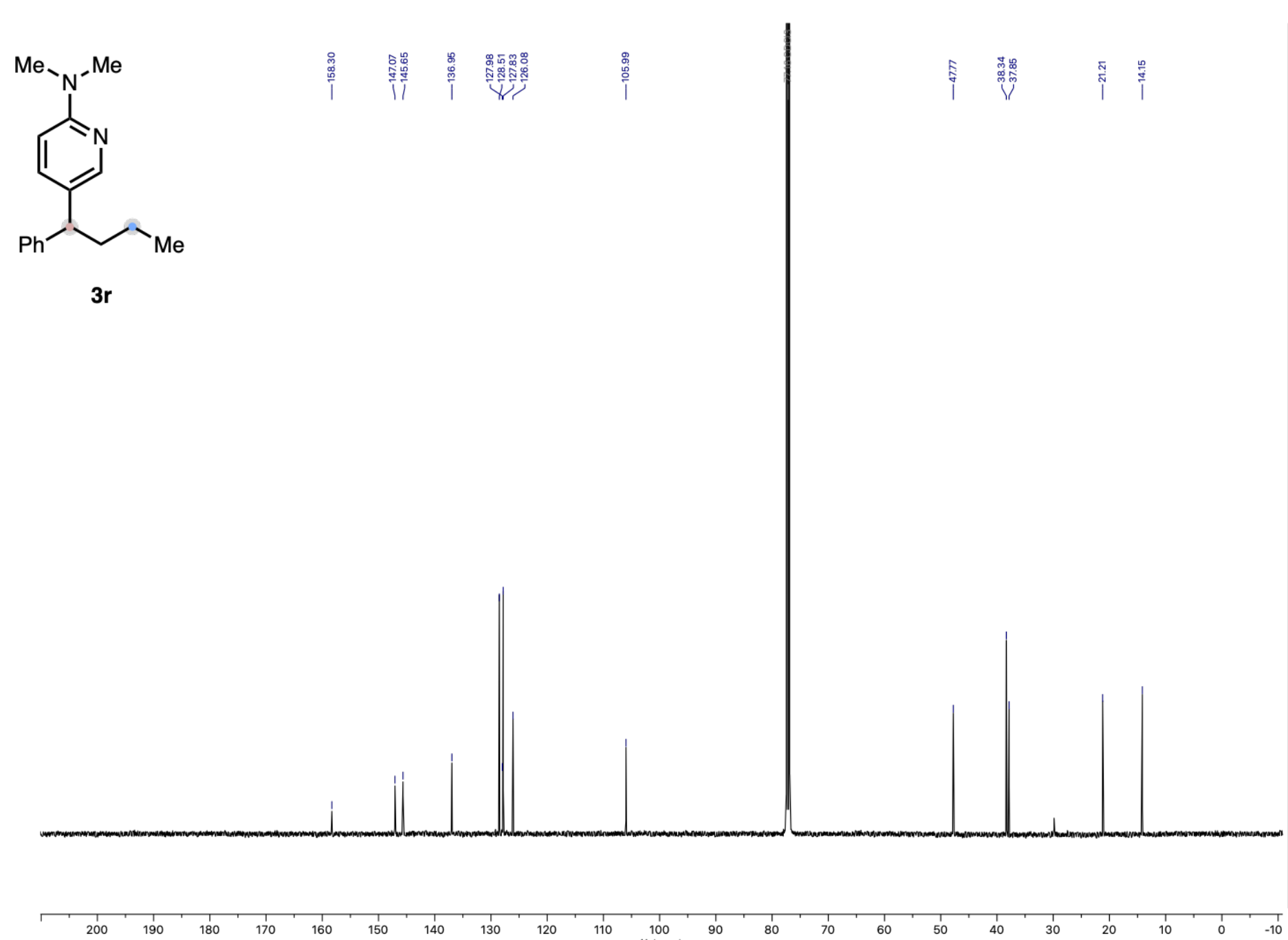

Methyl 5-(1-phenylbutyl)nicotinate **3s**:

^{1}H NMR (500 MHz; CDCl$_3$):

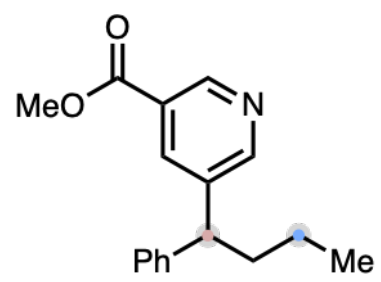

3s

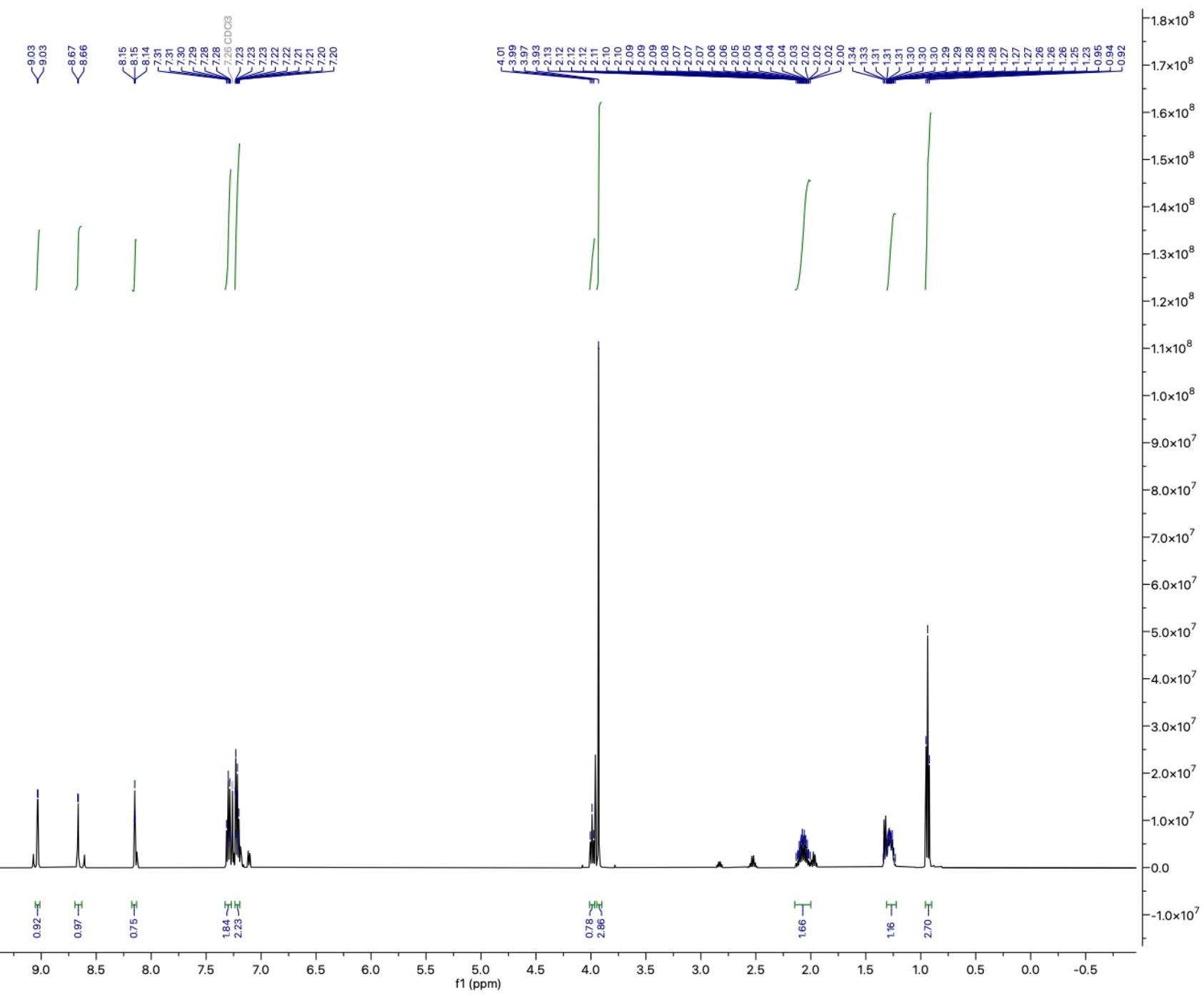

^{13}C NMR (126 MHz; CDCl$_3$):

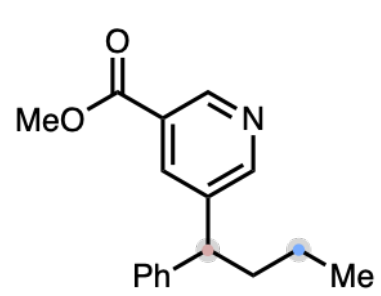

3s

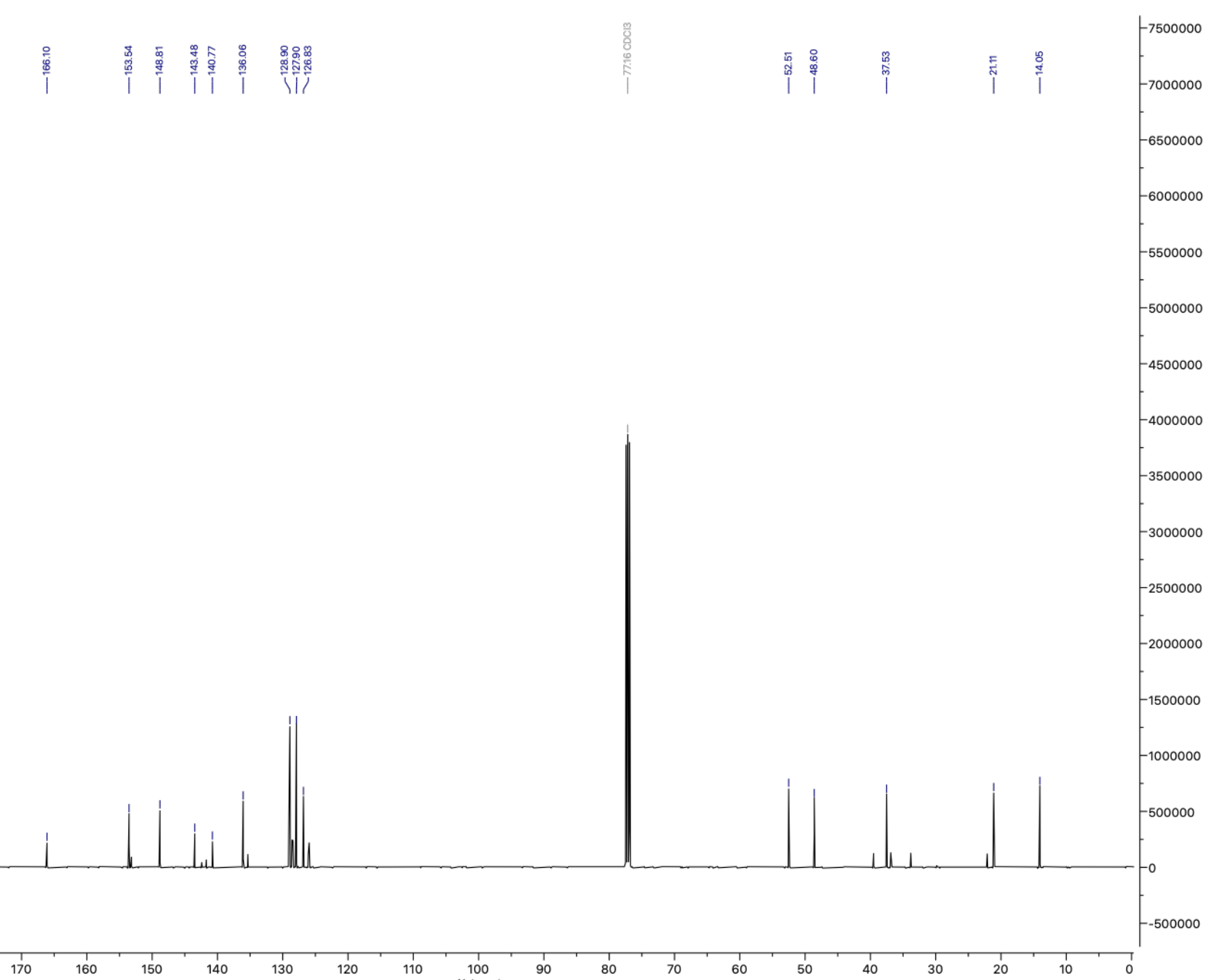

4-(5-(1-Phenylbutyl)pyrimidin-2-yl)morpholine **3t**:

^1H NMR (500 MHz; $CDCl_3$):

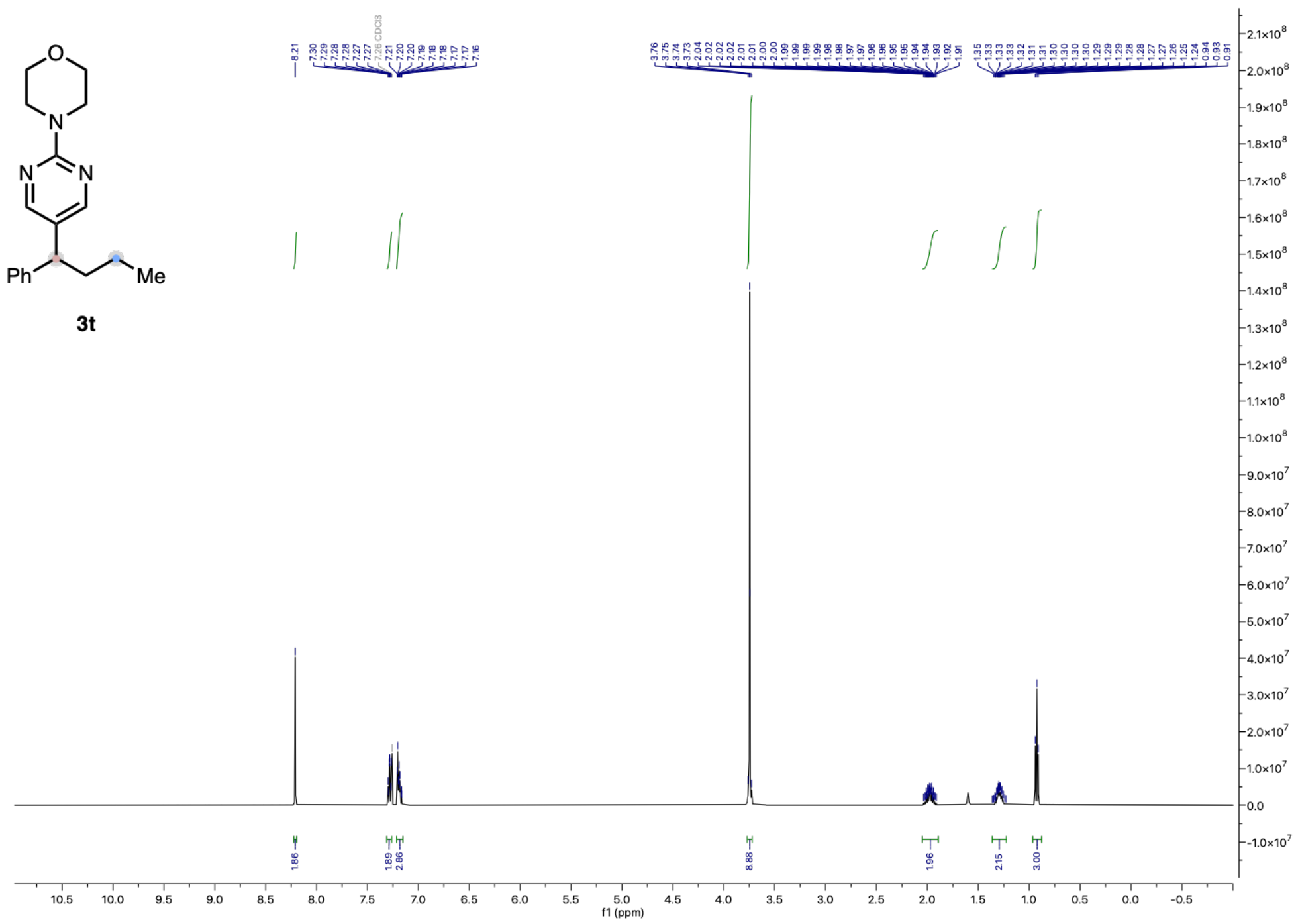

^{13}C NMR (126 MHz; $CDCl_3$):

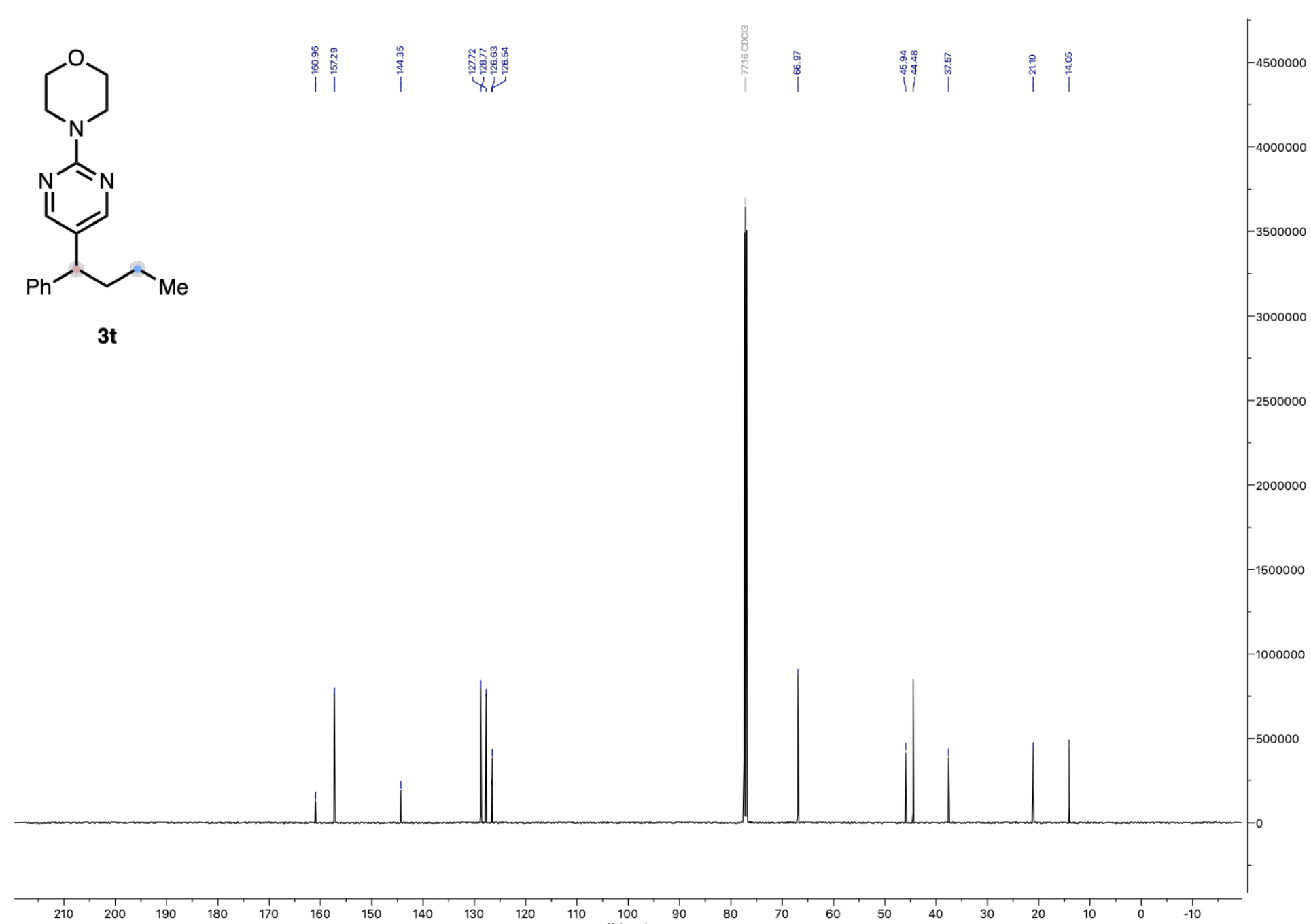

1-Fluoro-4-(1-(4-methoxyphenyl)propyl)benzene **3u**:

^{1}H NMR (500 MHz, $CDCl_3$):

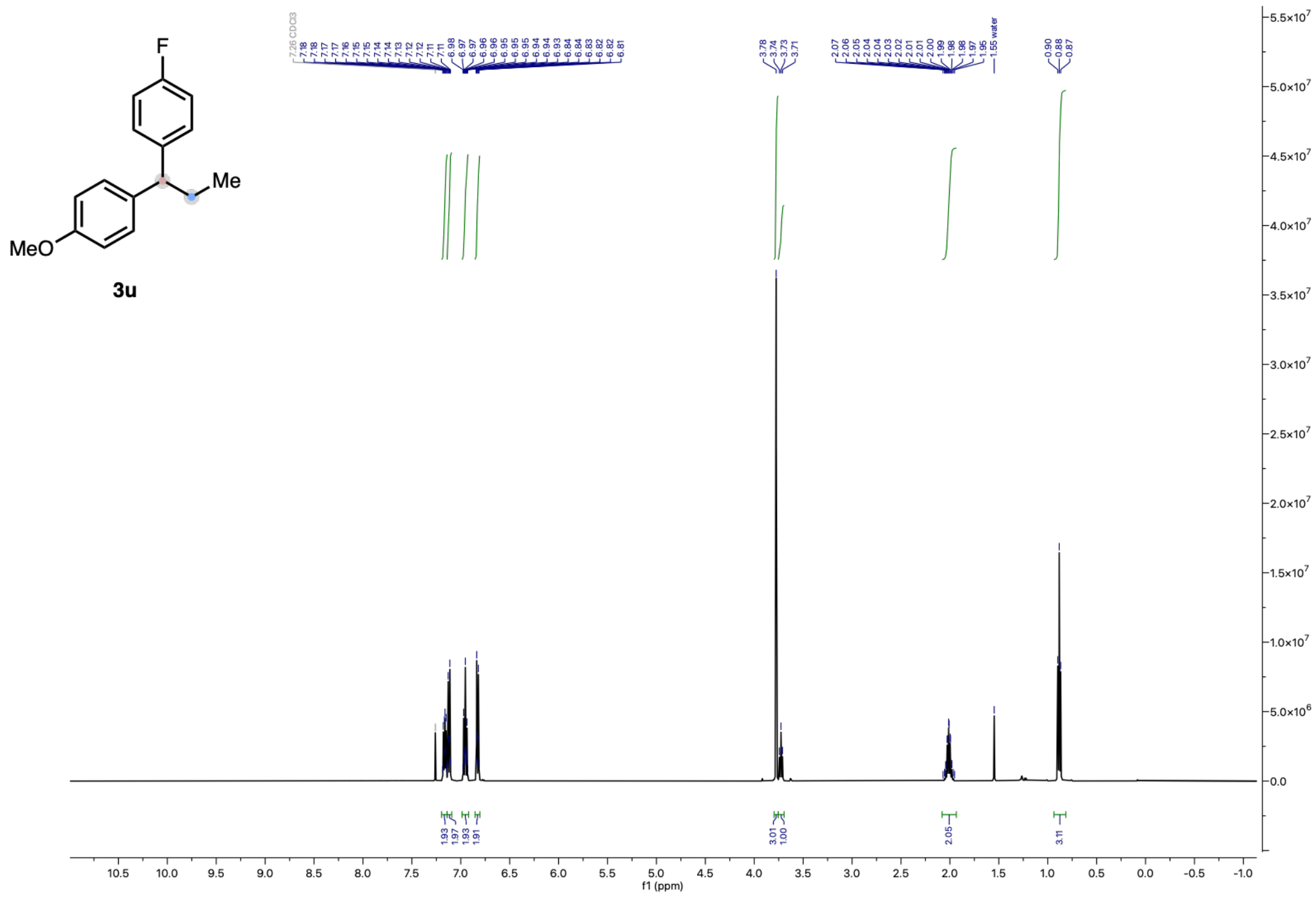

^{13}C NMR (126 MHz, $CDCl_3$):

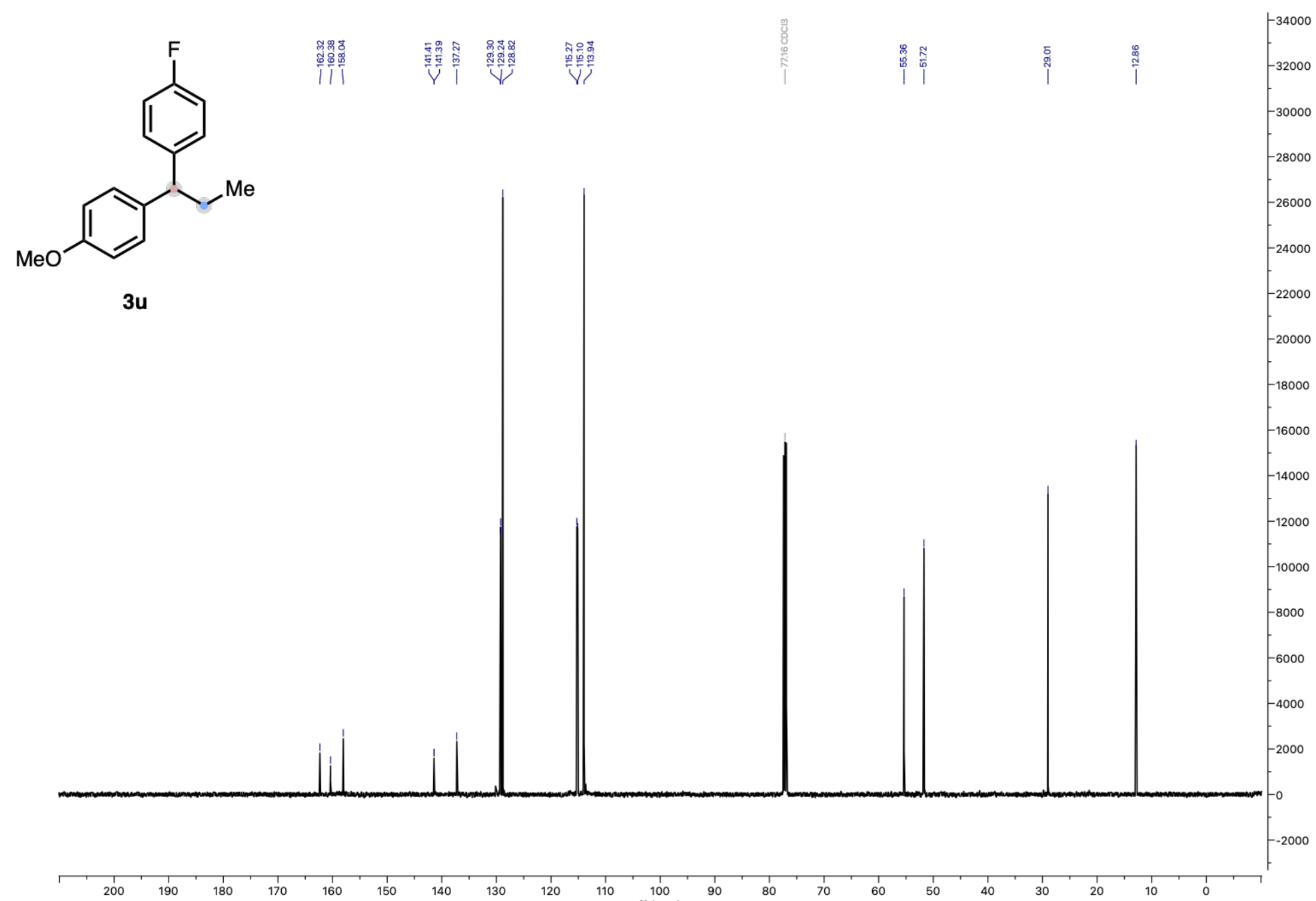

^{19}F NMR (376 MHz, CDCl$_3$):

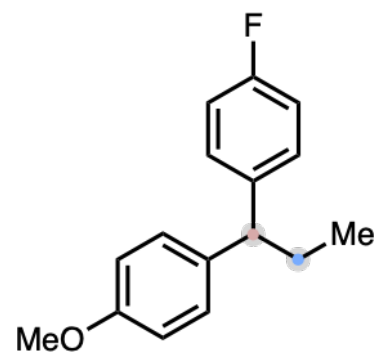

3u

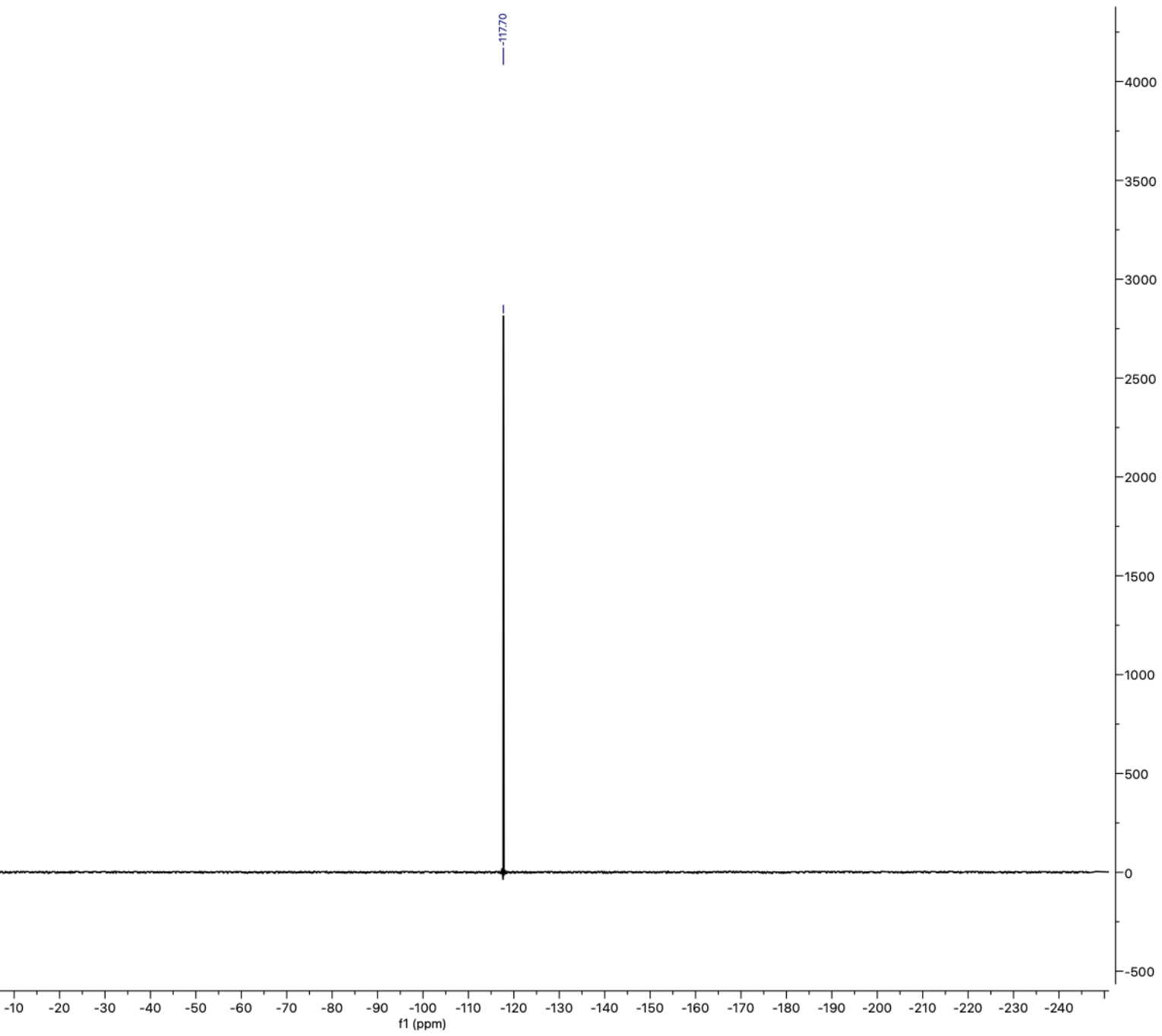

1-Fluoro-4-(1-(4-methoxyphenyl)butyl)benzene **3v**:

^{1}H NMR (500 MHz; $CDCl_3$):

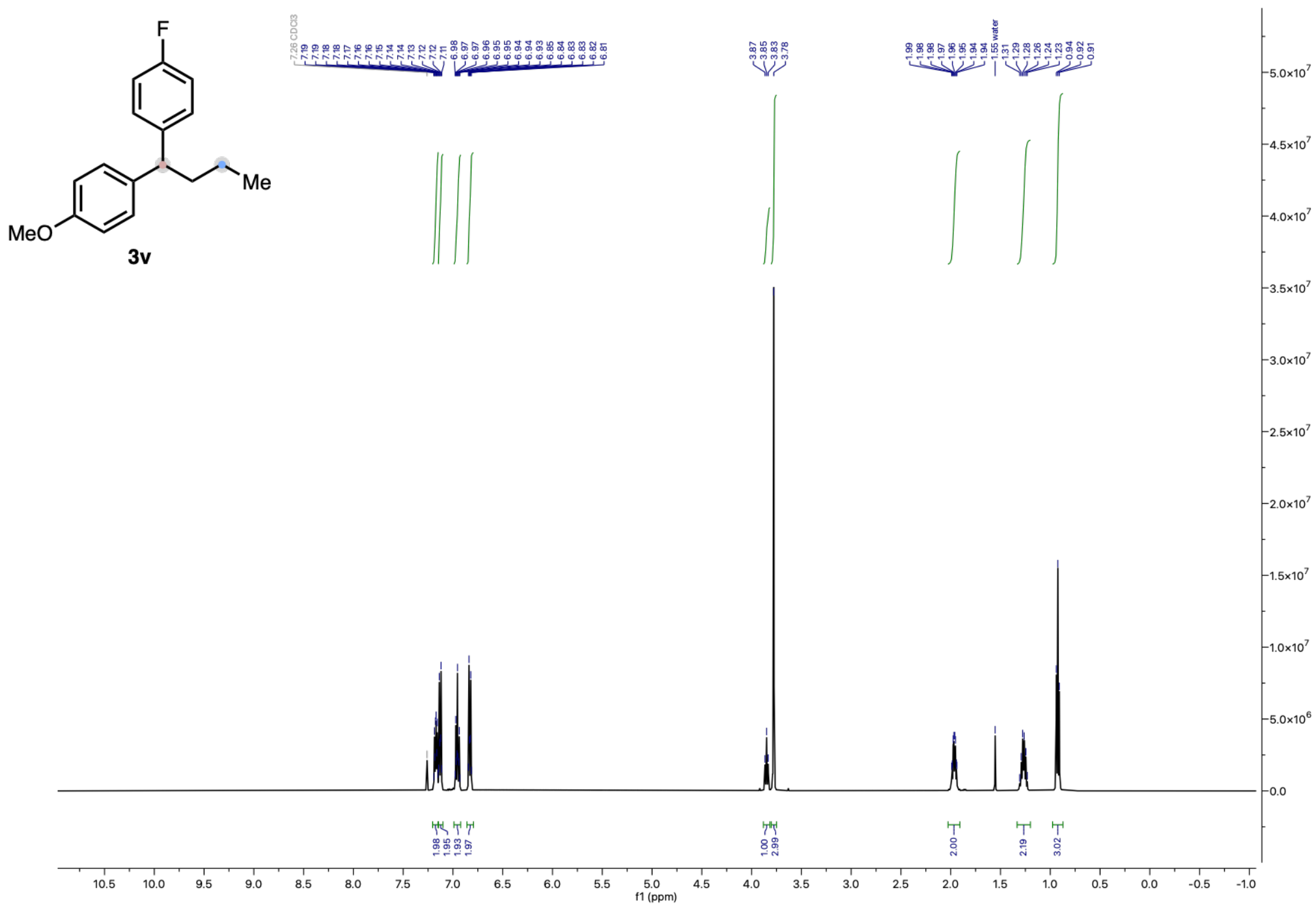

^{13}C NMR (126 MHz; $CDCl_3$):

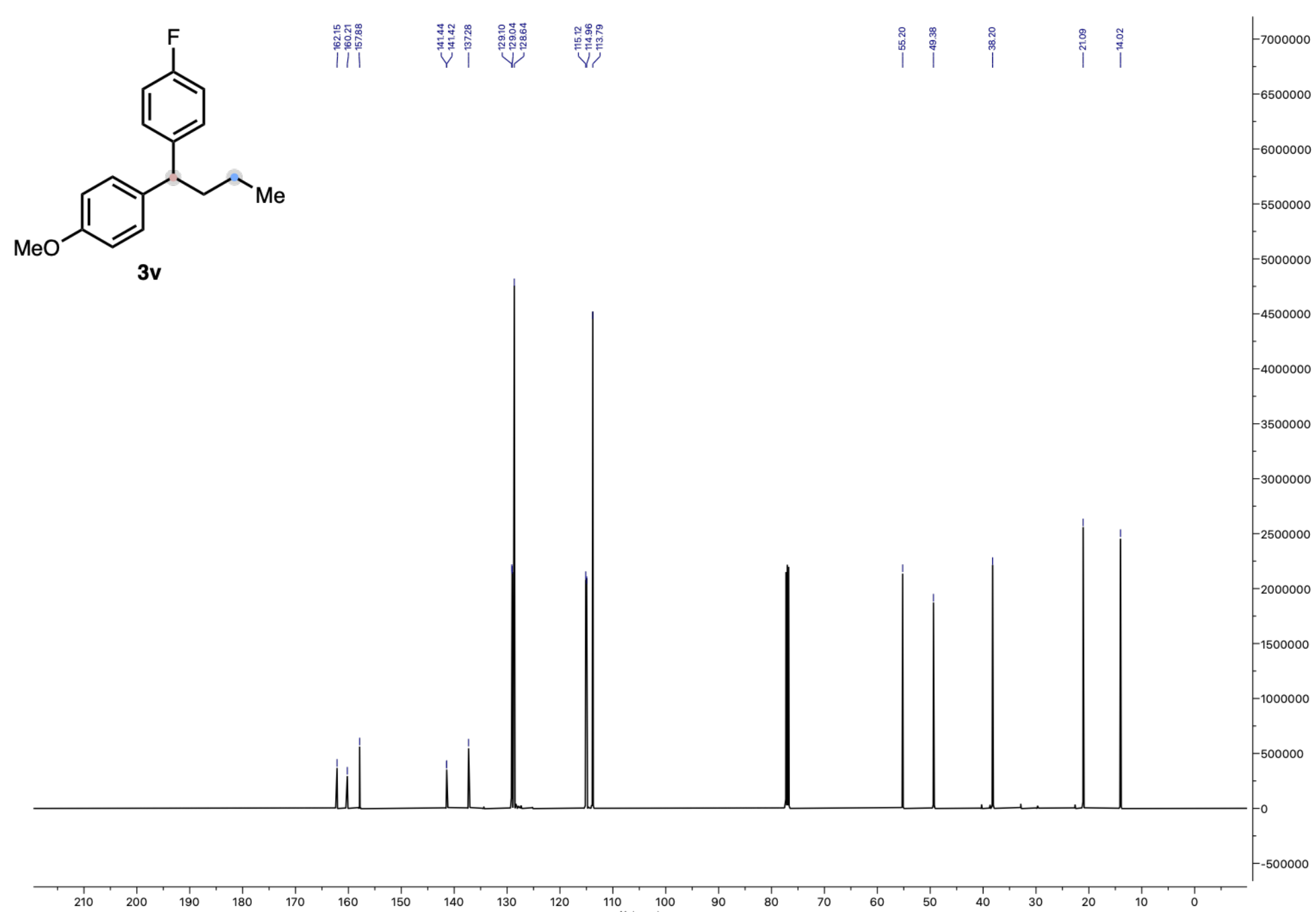

^{19}F NMR (282 MHz, $CDCl_3$):

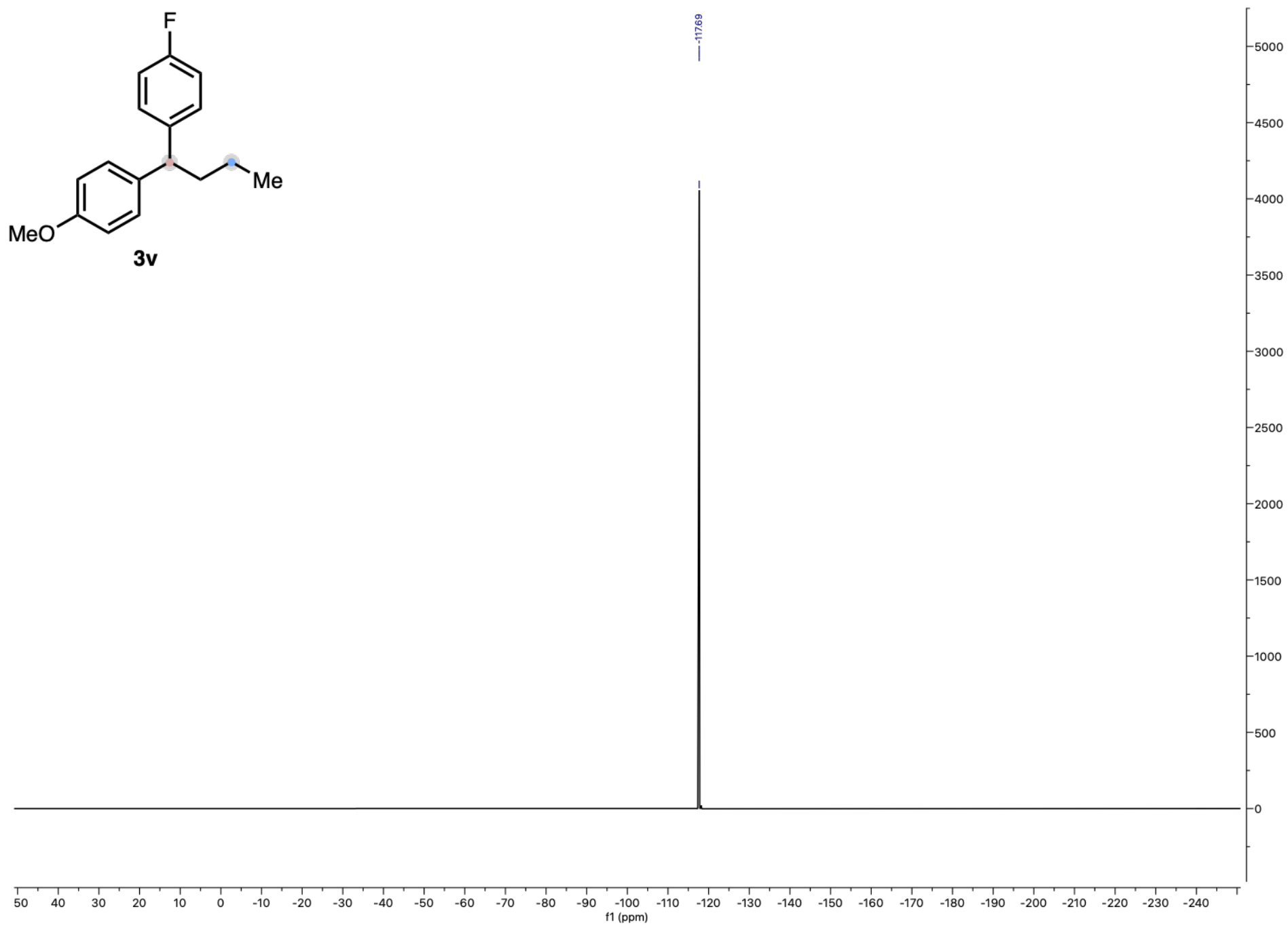

1-fluoro-4-(1-phenylheptyl)benzene **3w**:

^{1}H NMR (500 MHz; $CDCl_3$):

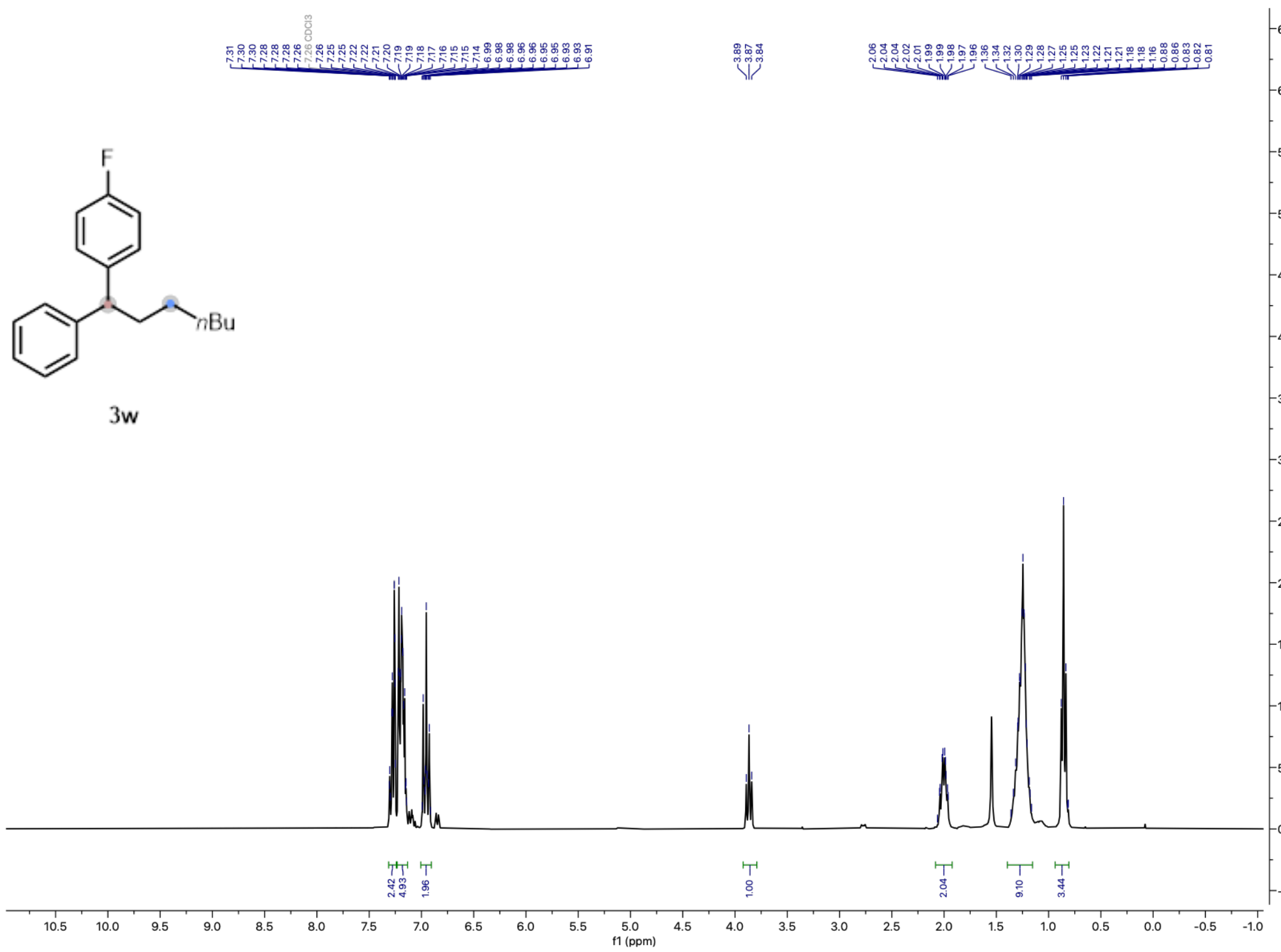

^{13}C NMR (126 MHz, $CDCl_3$):

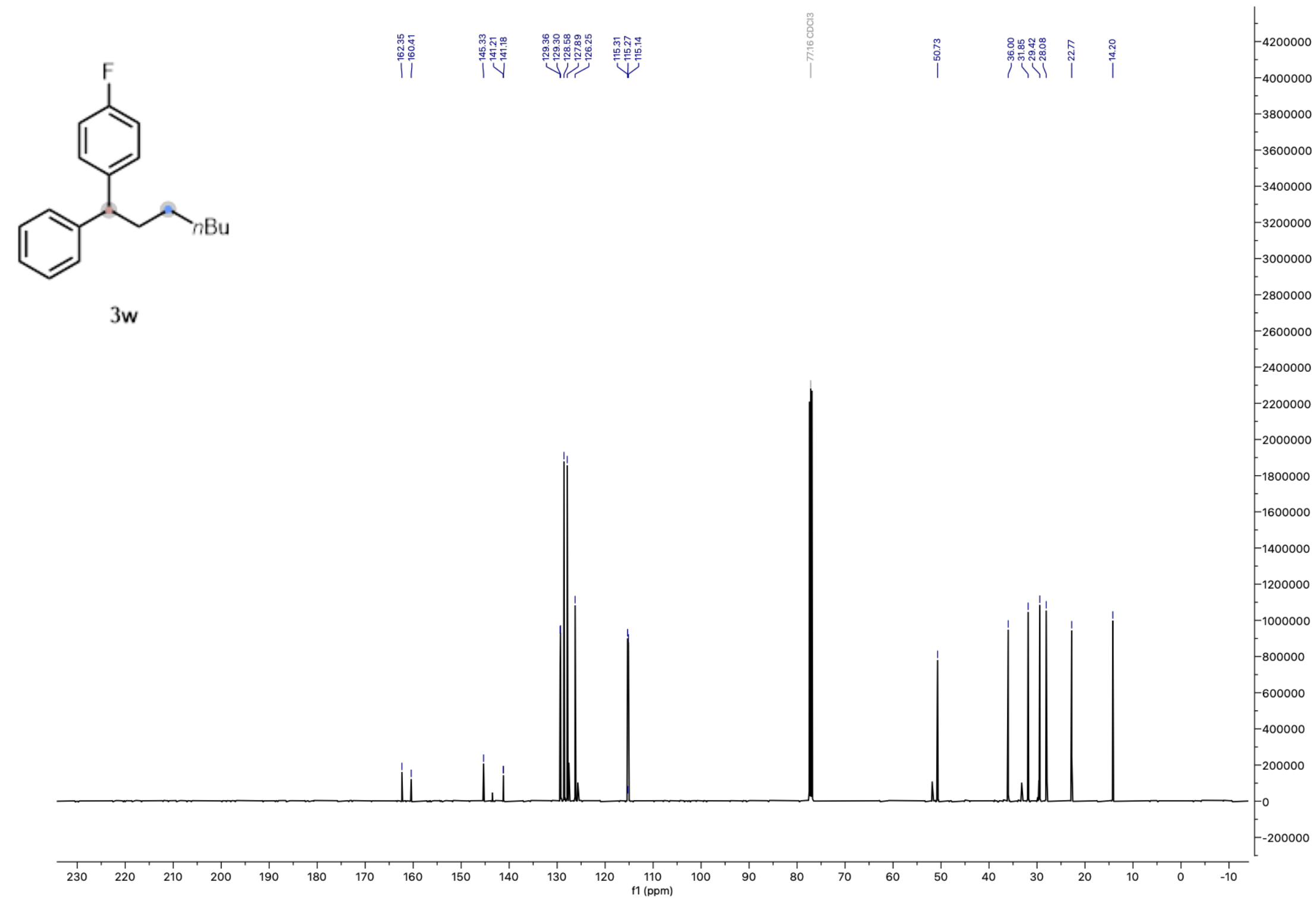

^{19}F NMR (282 MHz, $CDCl_3$):

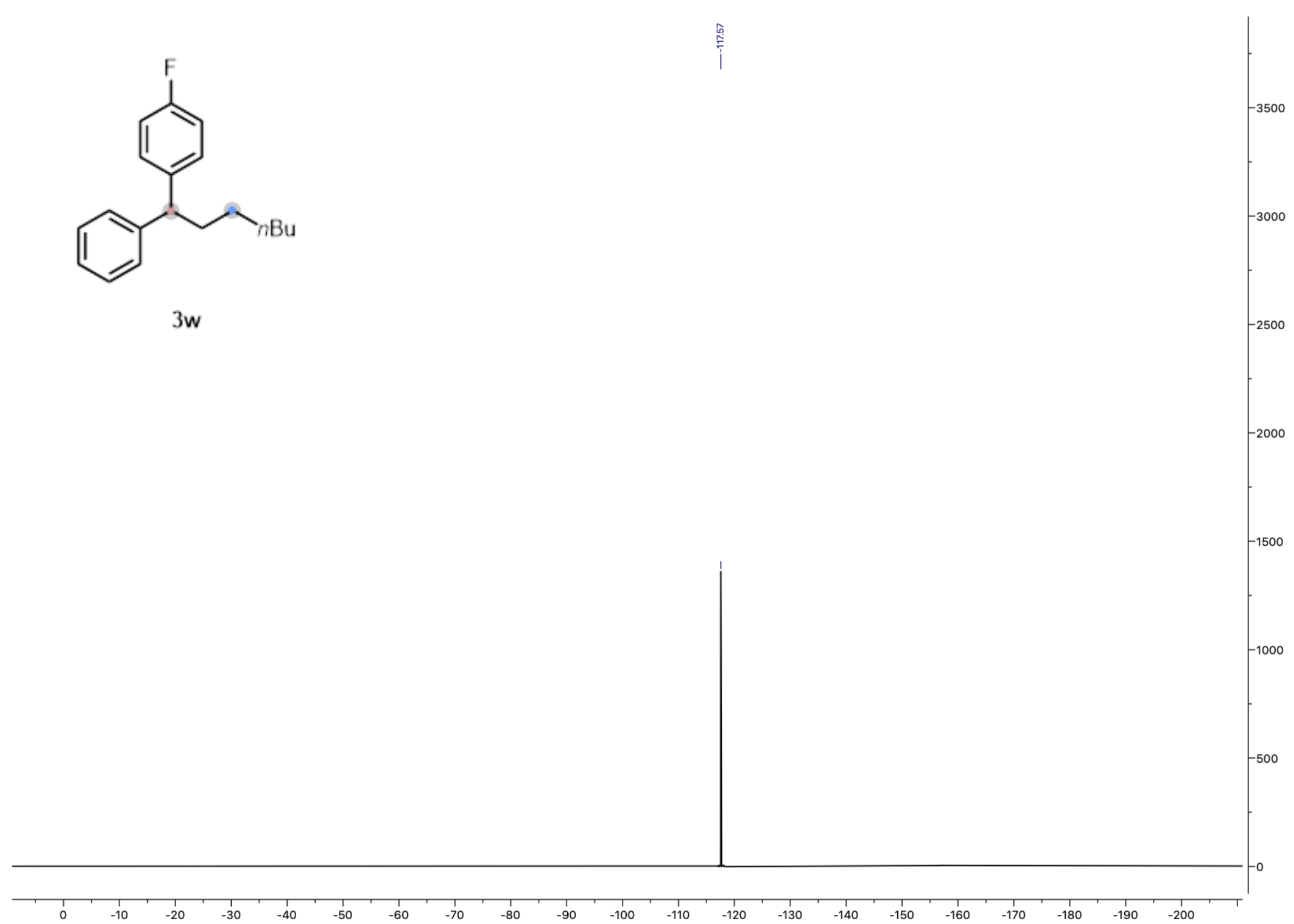

1-Fluoro-4-(1-phenylpentyl)benzene **3x**:

^{1}H NMR (300 MHz; $CDCl_3$):

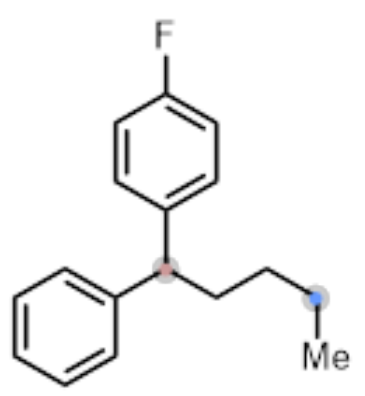

3x

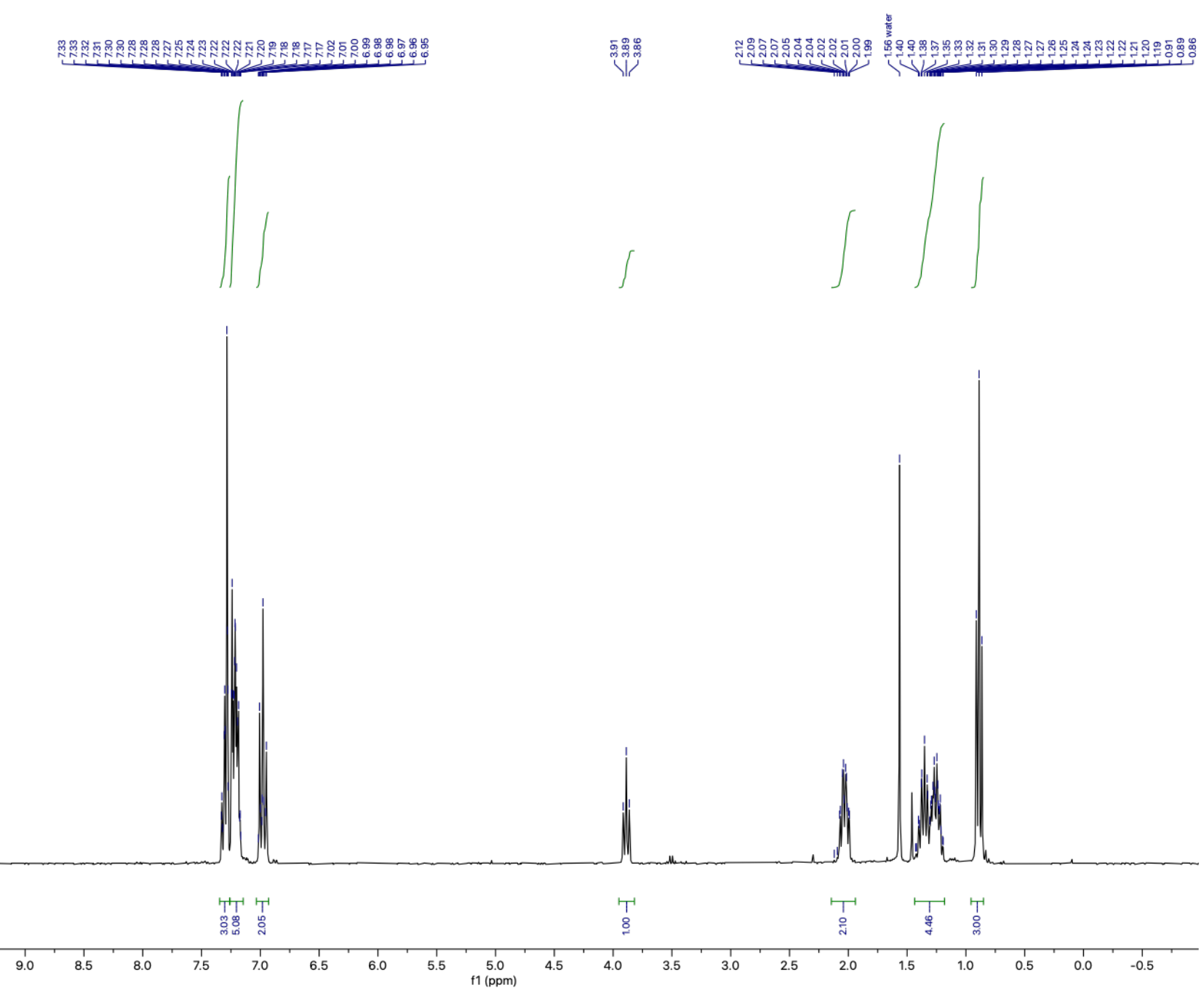

^{13}C NMR (126 MHz, $CDCl_3$):

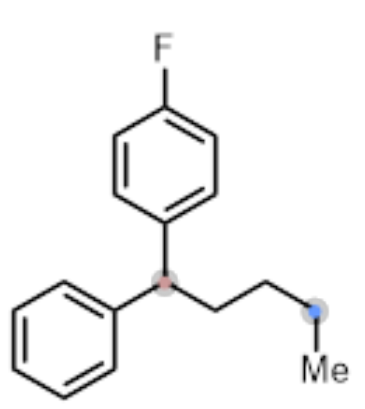

3x

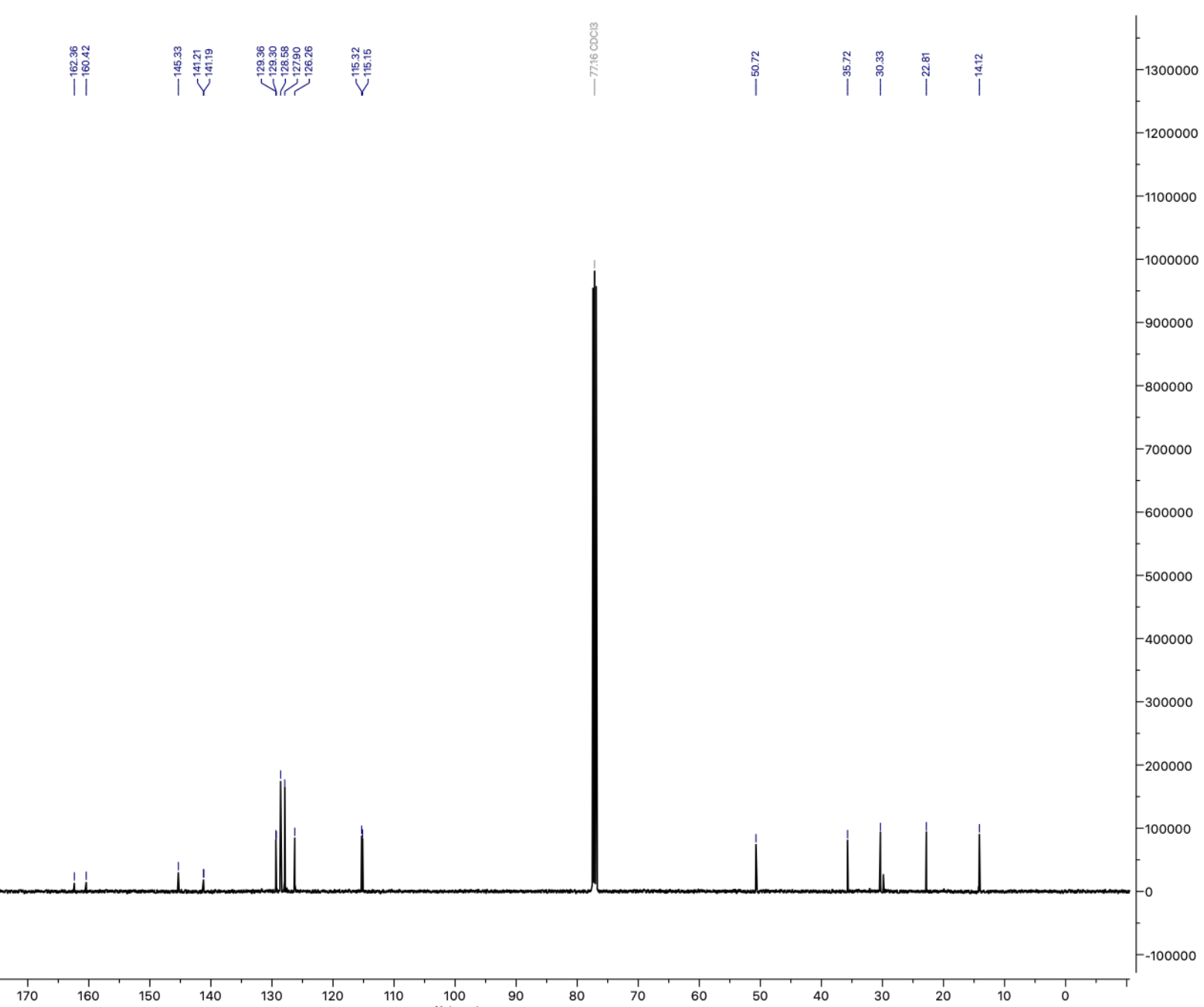

^{19}F NMR (282 MHz, $CDCl_3$):

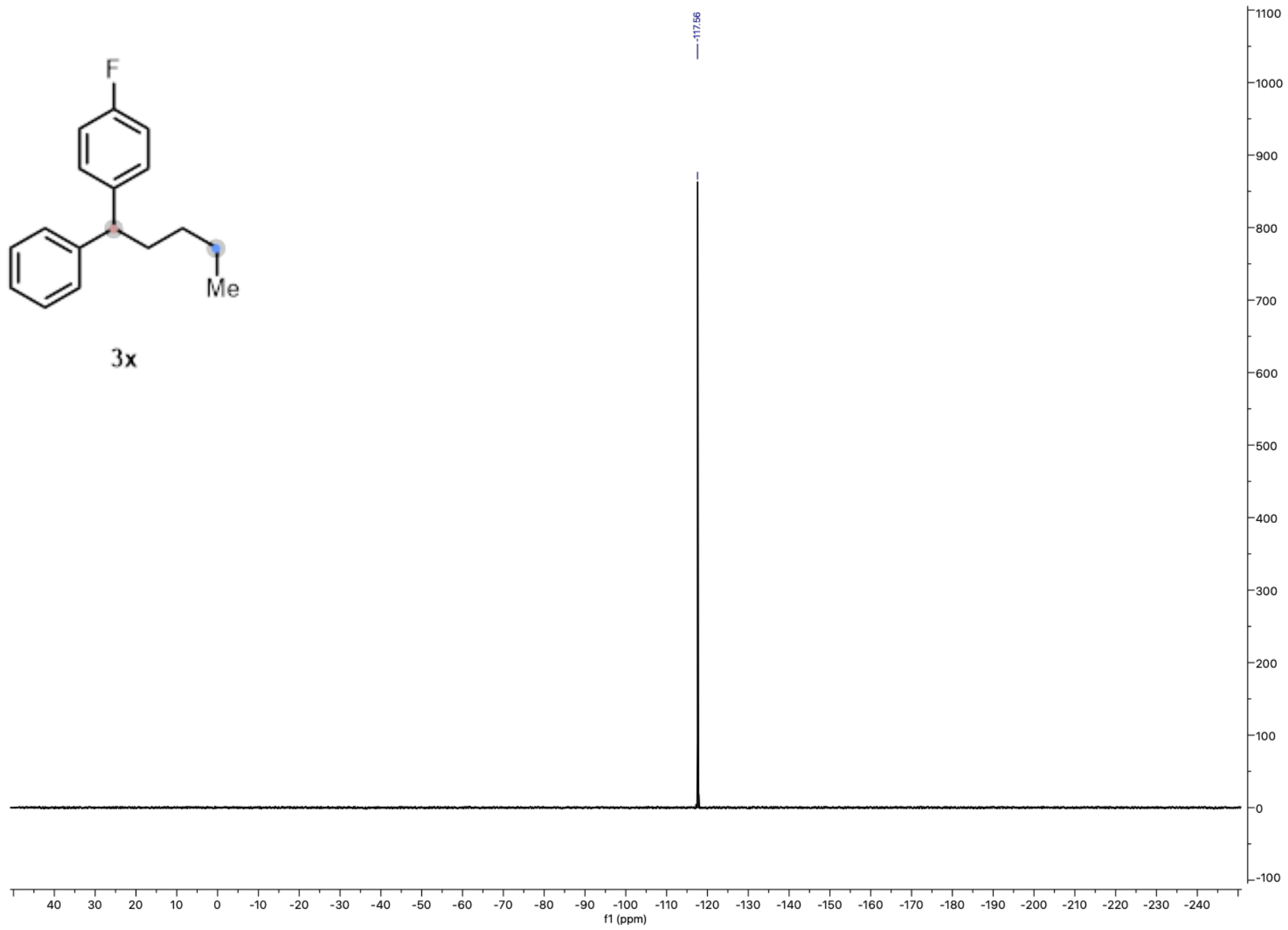

3x

4-(1-(4-Fluorophenyl)butyl)phenol, **3y**:

^{1}H NMR (300 MHz, $CDCl_3$):

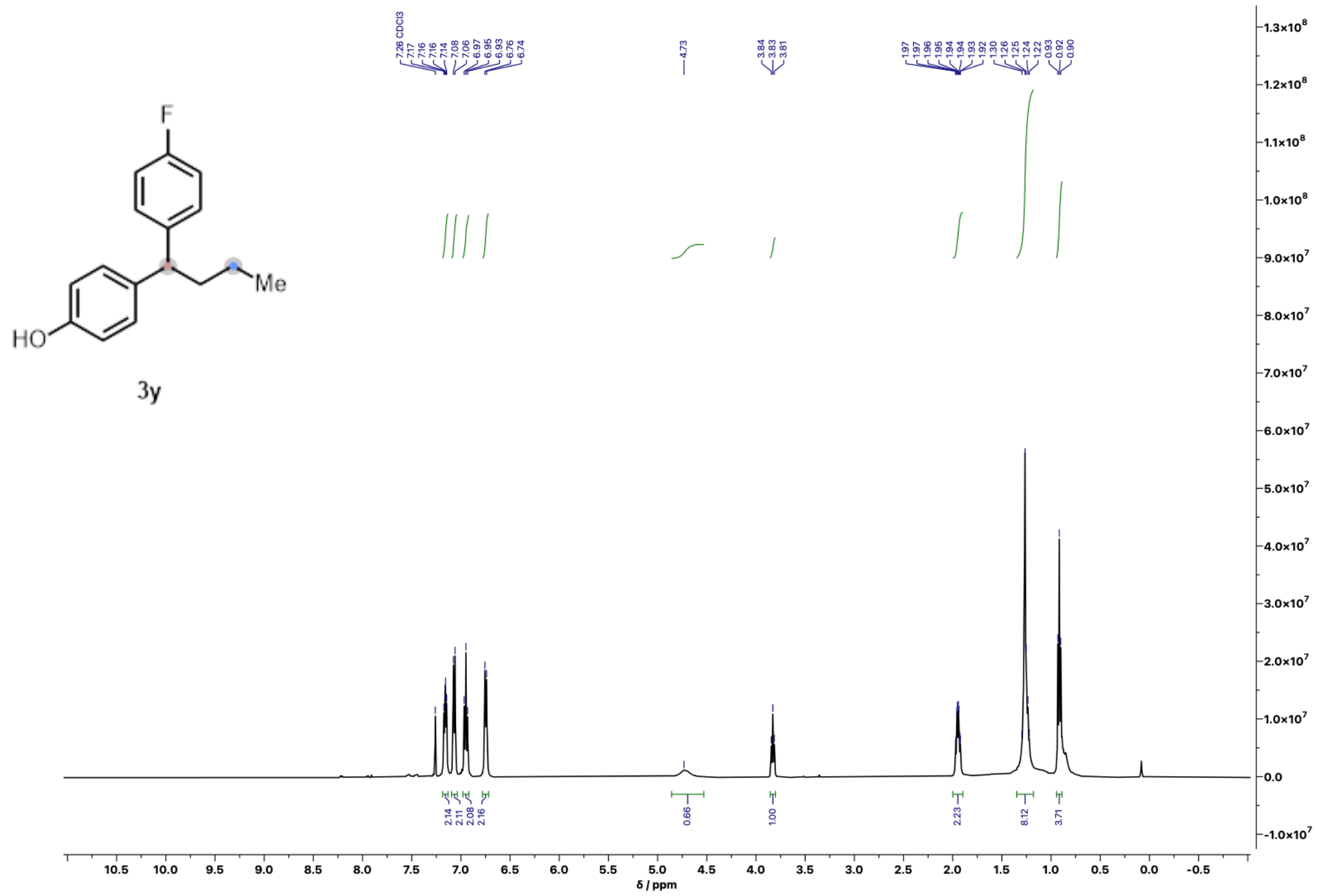

^{13}C NMR (126 MHz, $CDCl_3$):

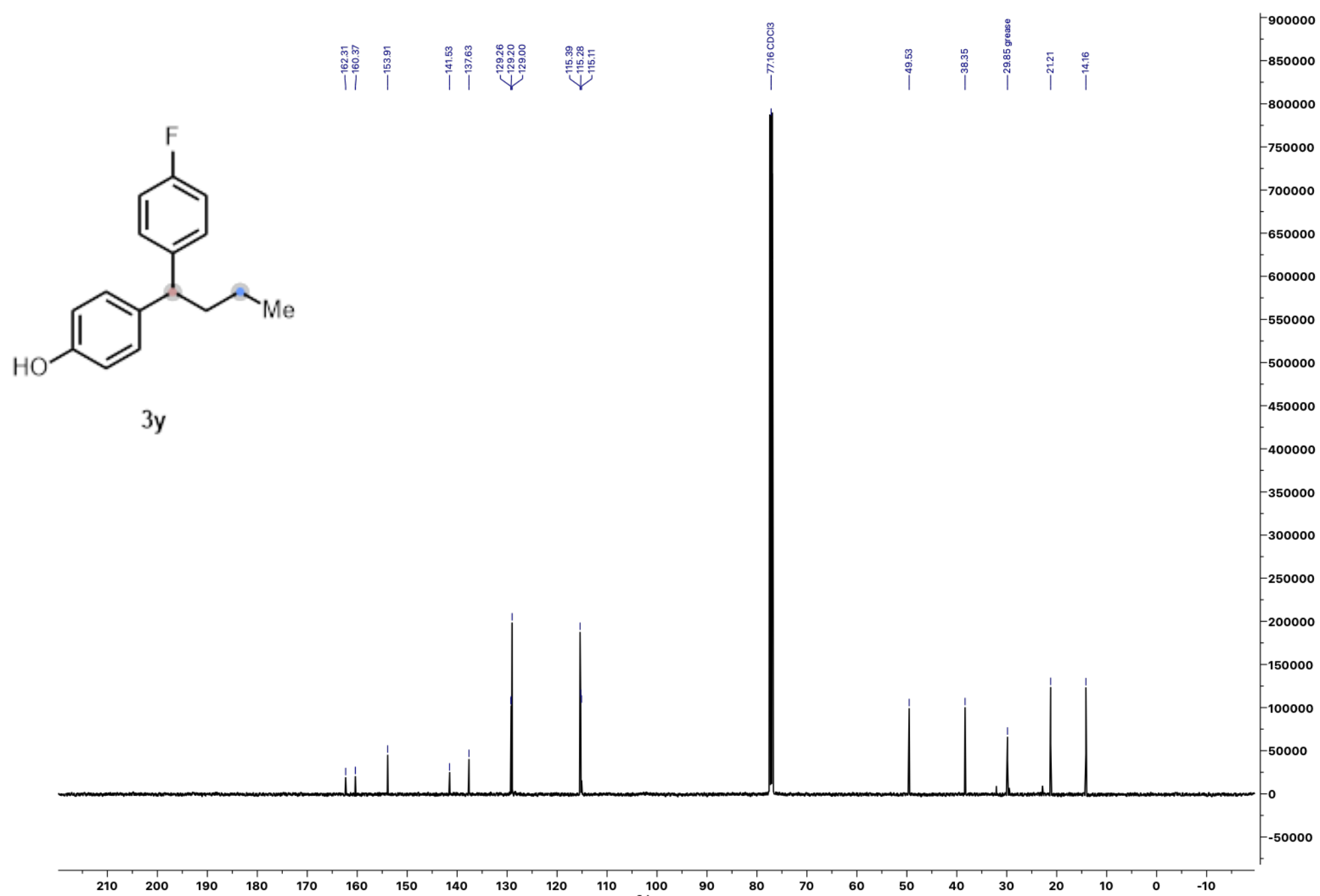

19F NMR (282 MHz, $CDCl_3$):

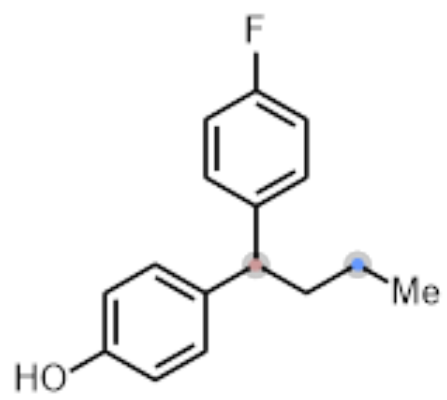

3y

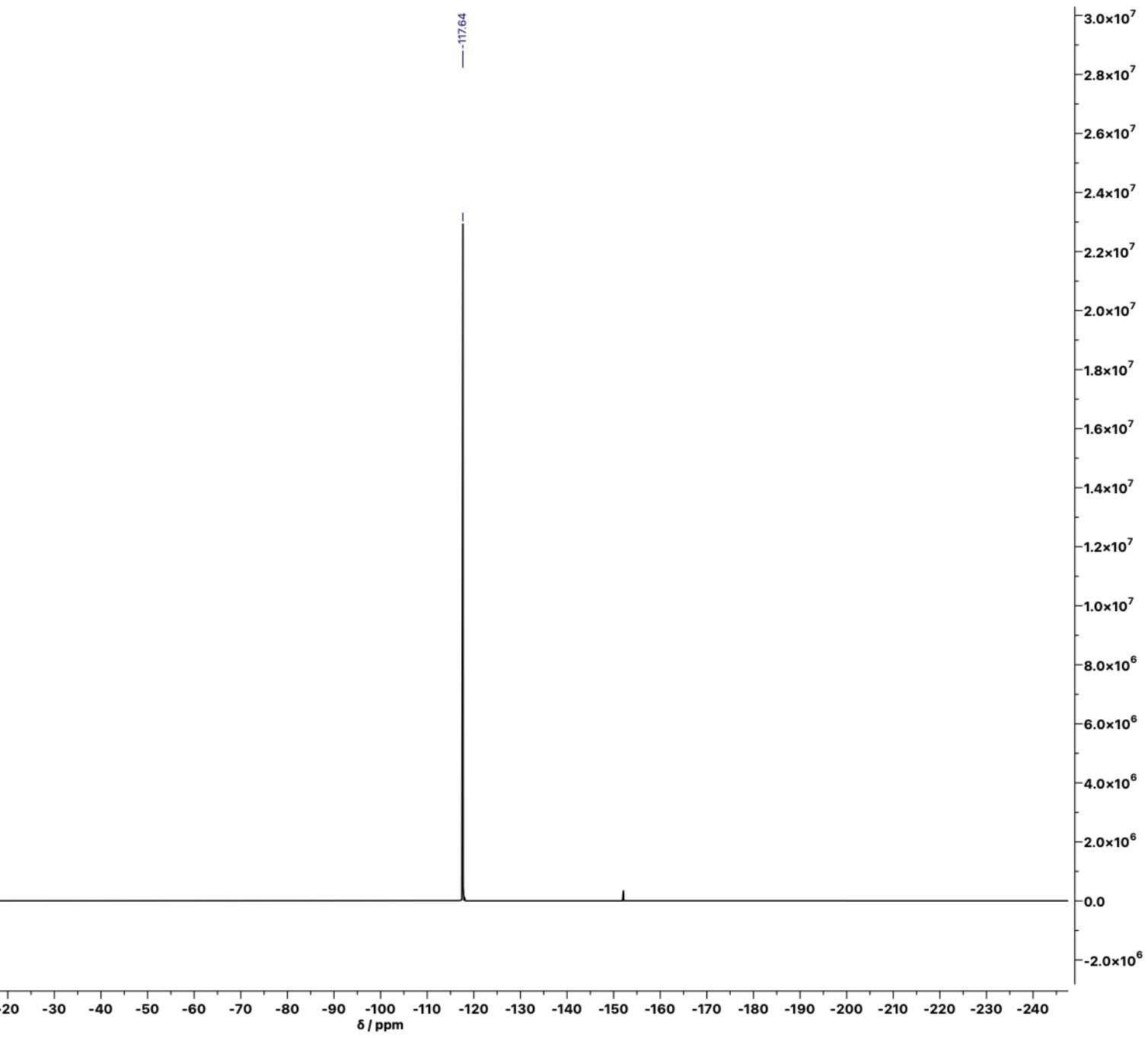

1-Chloro-4-(1-(4-fluorophenyl)propyl)benzene **3z**:

^{1}H NMR (300 MHz; $CDCl_3$):

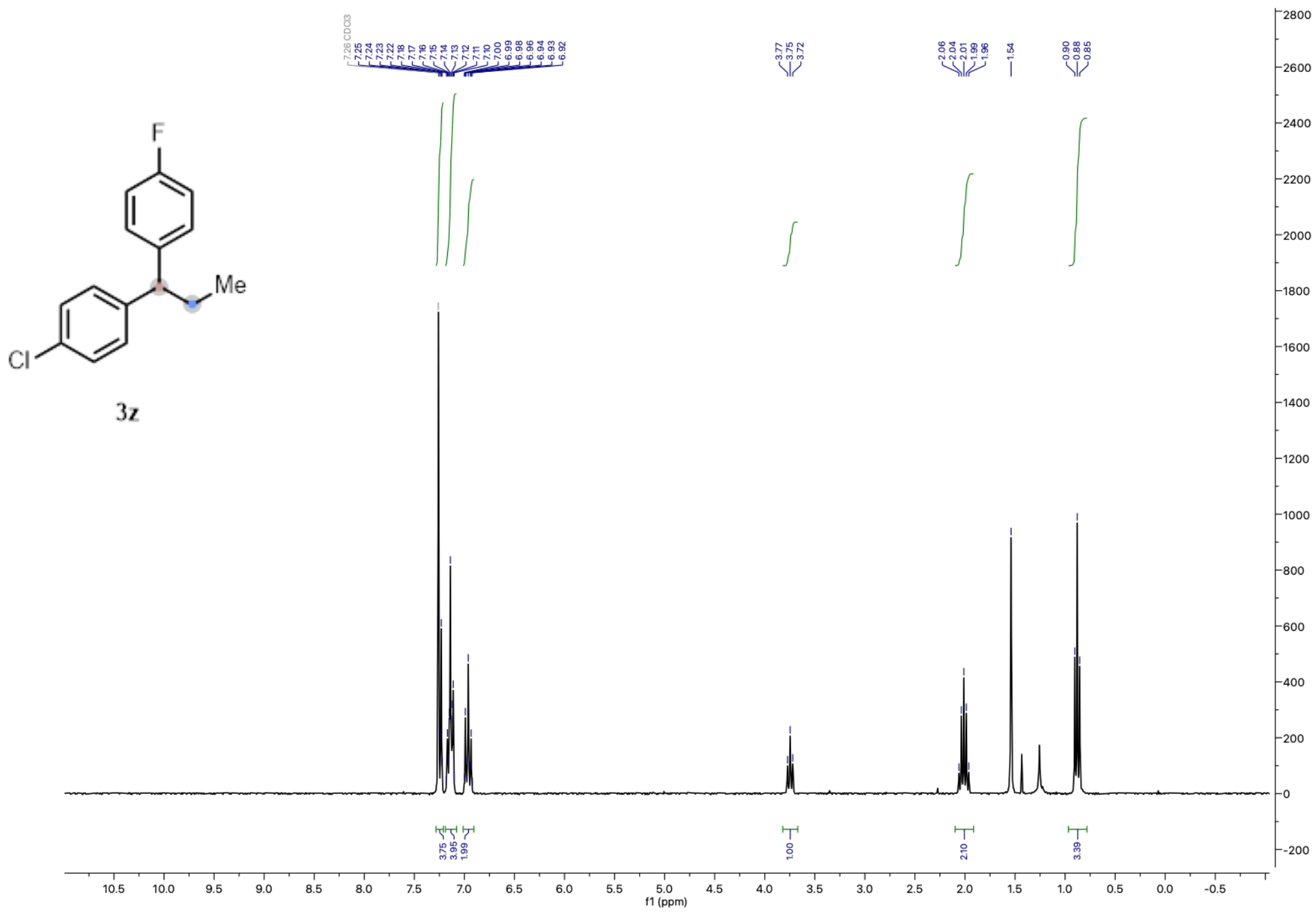

^{13}C NMR (126 MHz; $CDCl_3$):

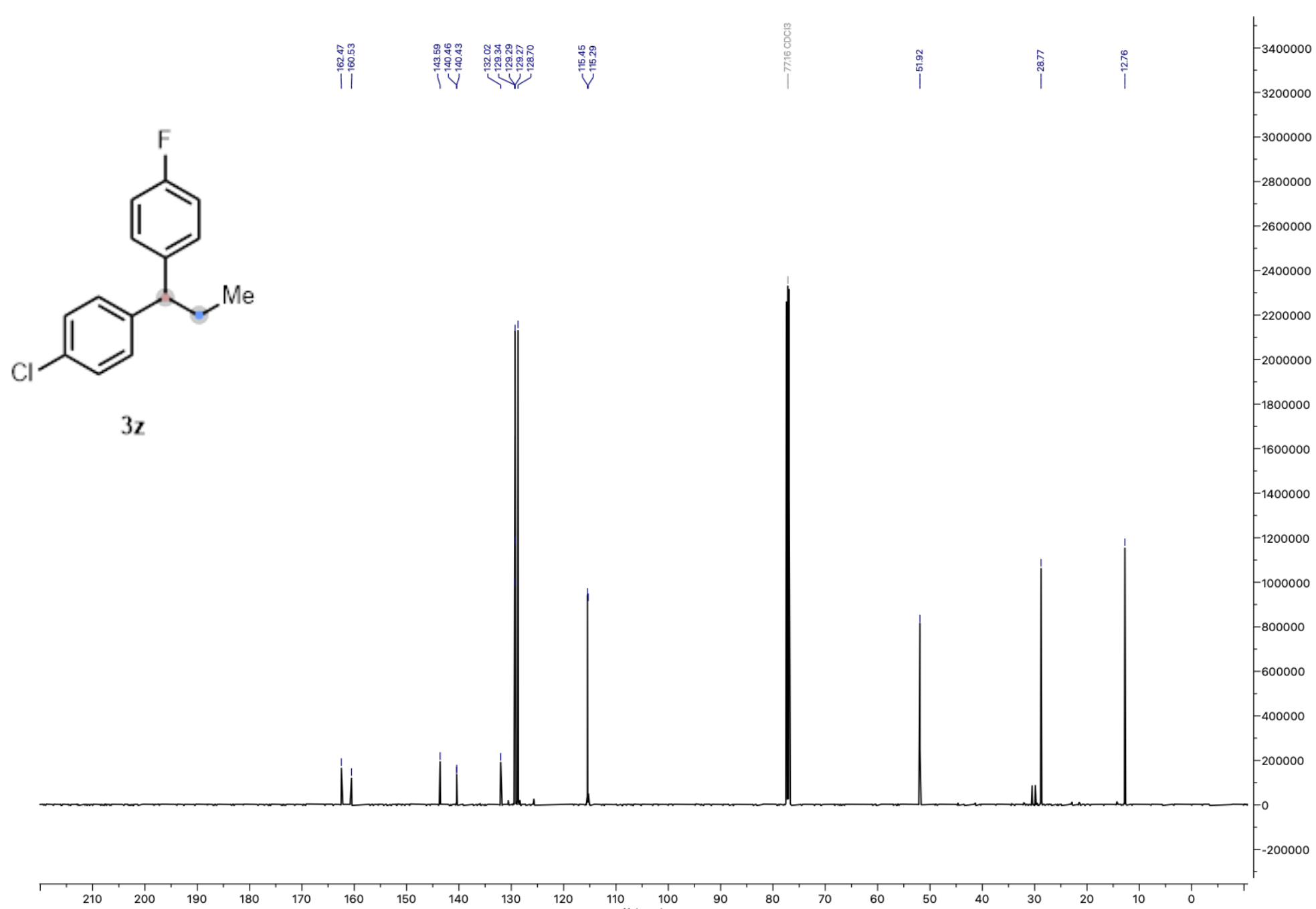

^{19}F NMR (282 MHz; $CDCl_3$):

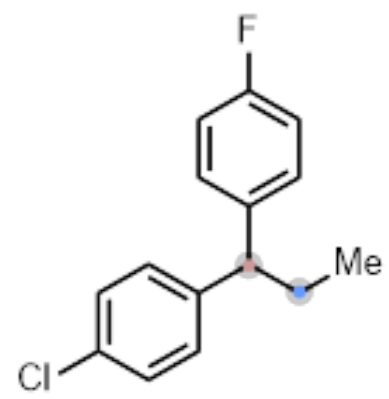

3z

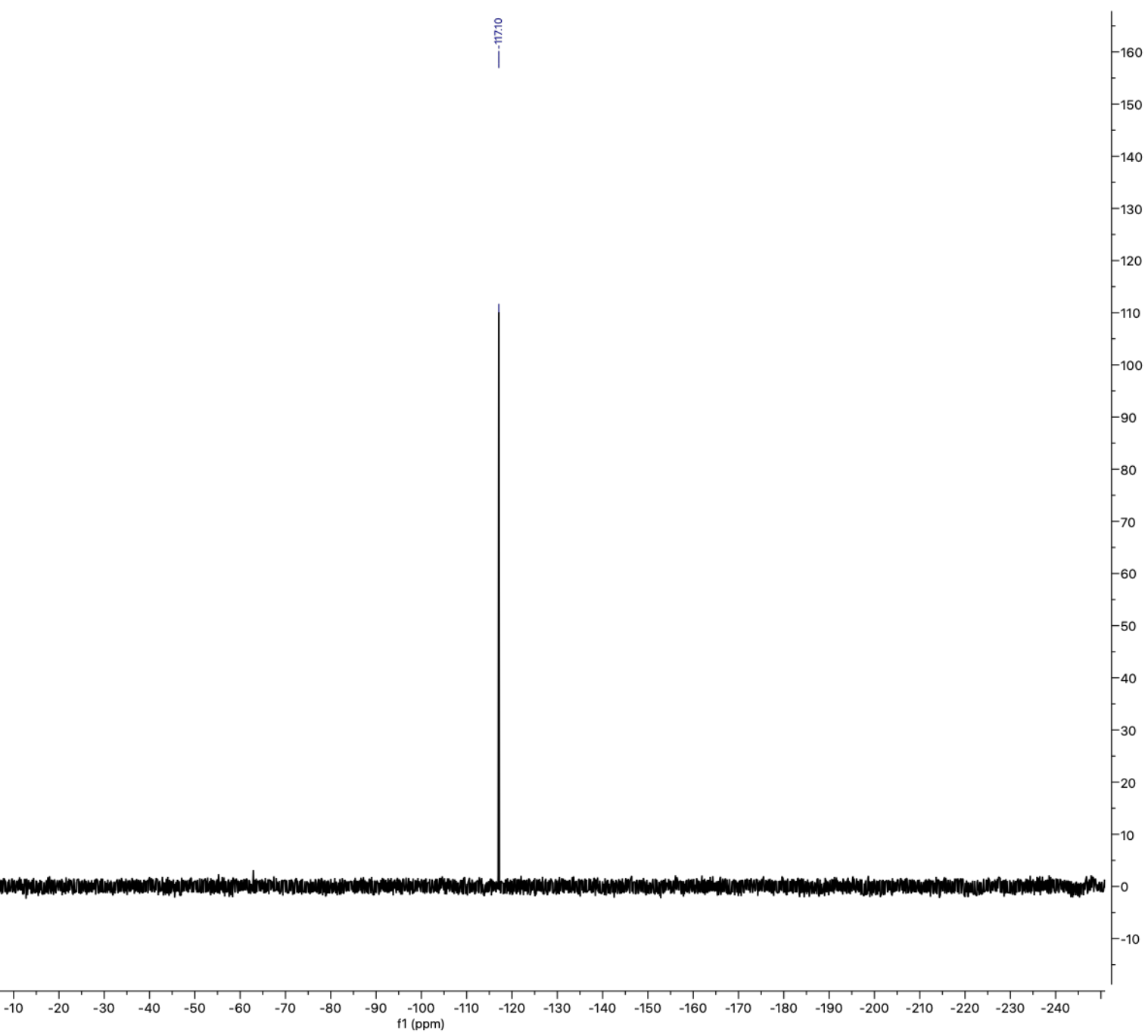

4,4'-(Propane-1,1-diyl)bis(fluorobenzene) **3aa**:

^{1}H NMR (300 MHz; $CDCl_3$):

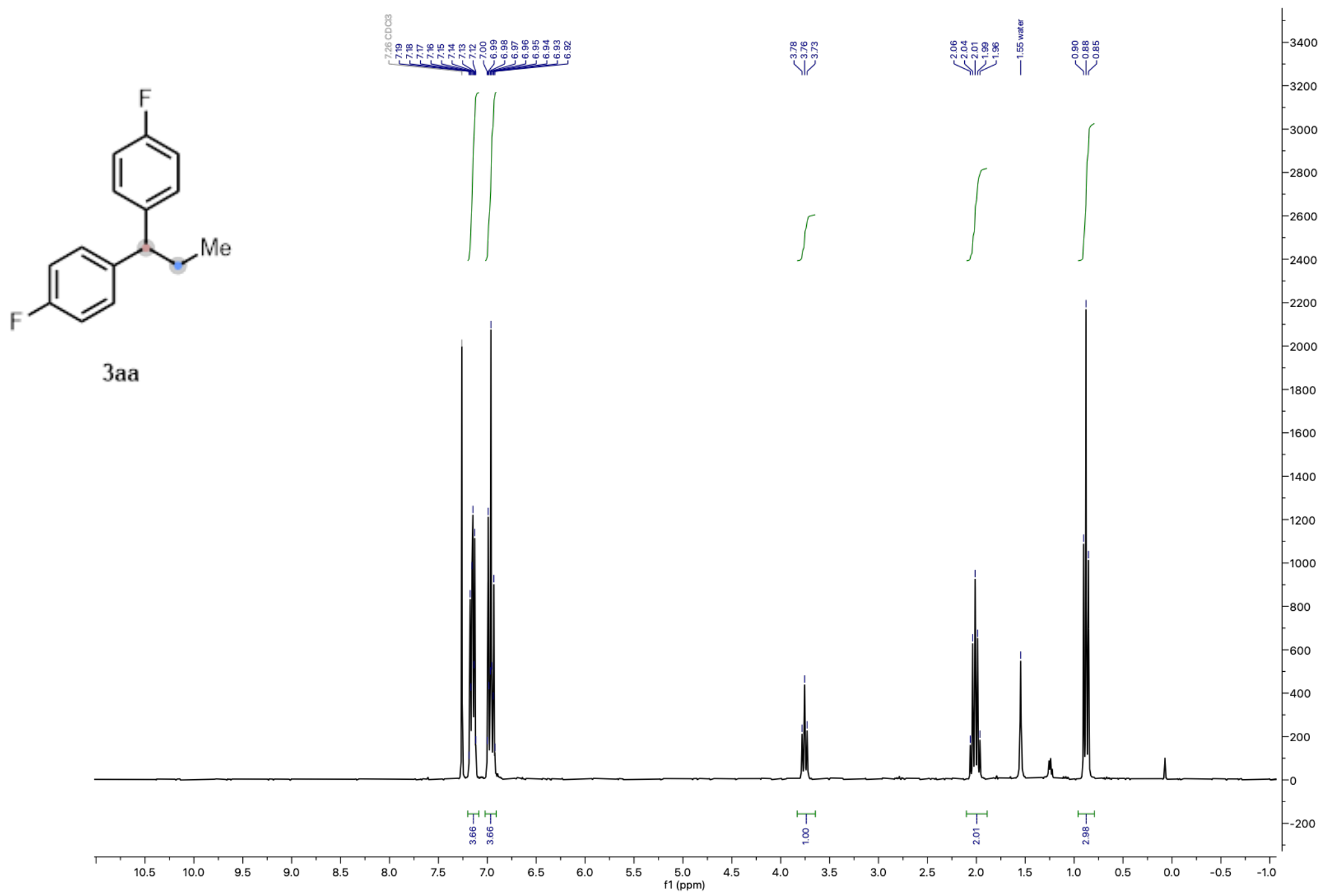

^{13}C NMR (126 MHz; $CDCl_3$):

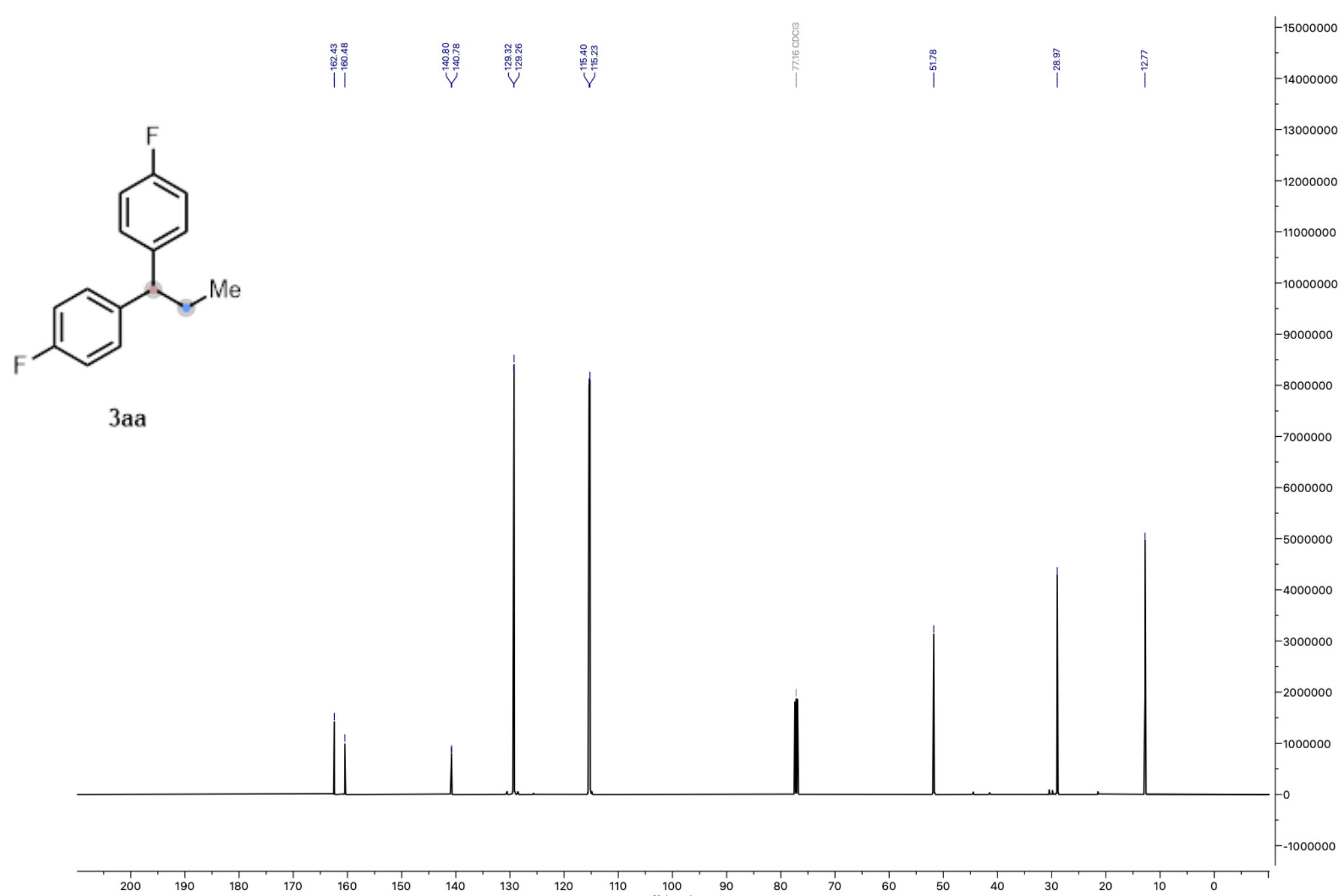

^{19}F NMR (282 MHz; $CDCl_3$):

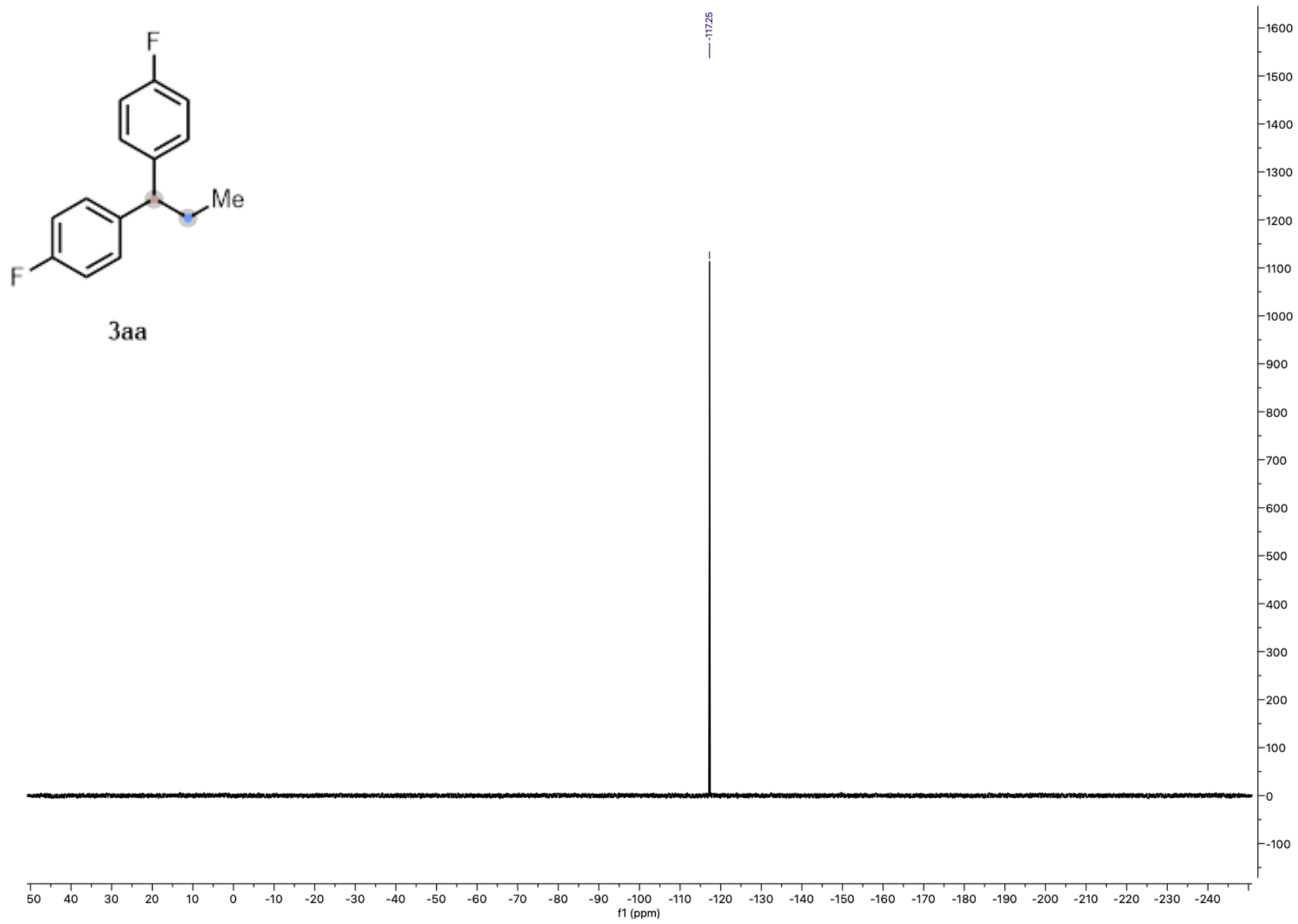

3aa

1-Fluoro-4-(1-(4-(trifluoromethyl)phenyl)propyl)benzene **3ab**:

^{1}H NMR (300 MHz; $CDCl_3$):

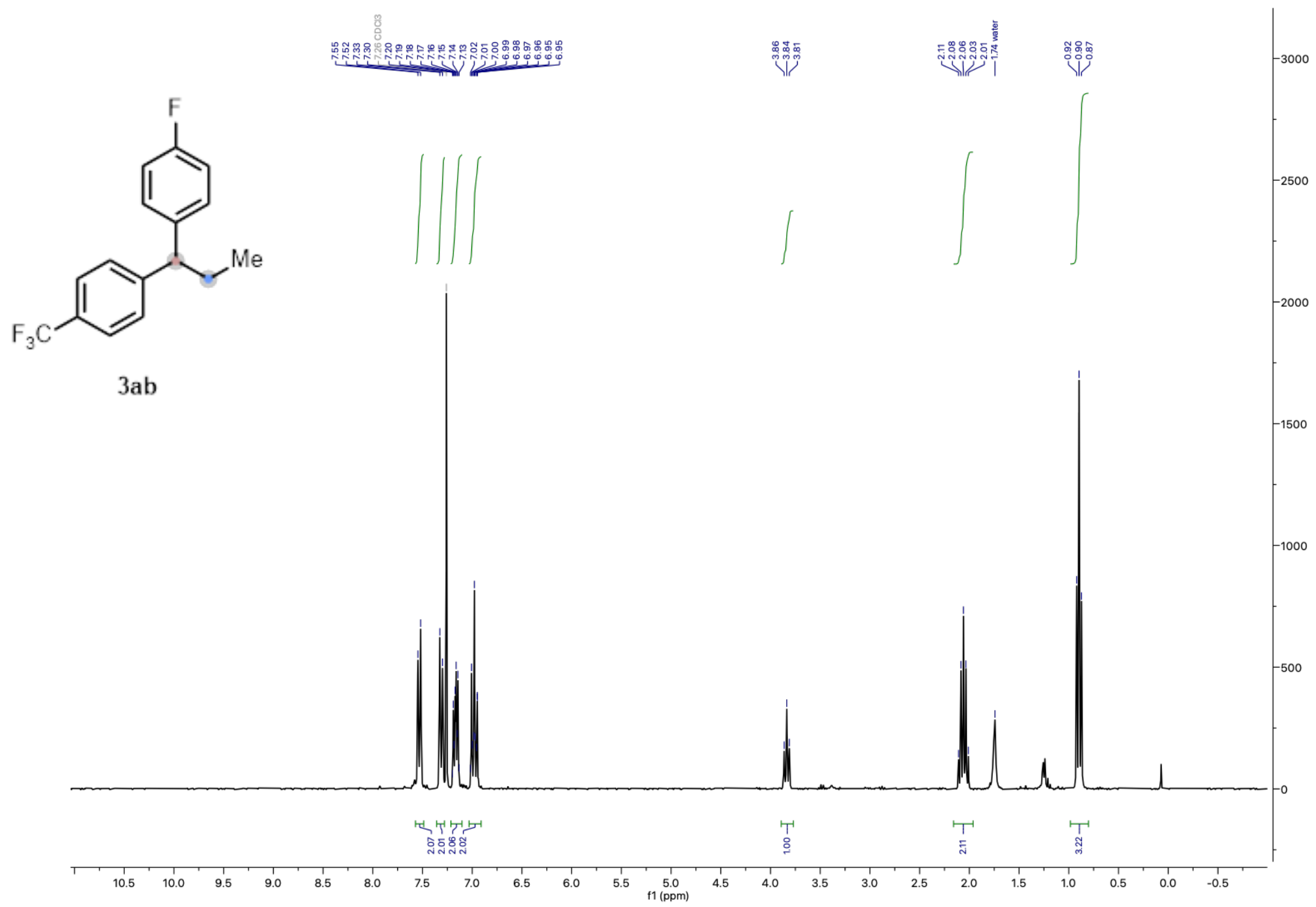

^{13}C NMR (126 MHz; $CDCl_3$):

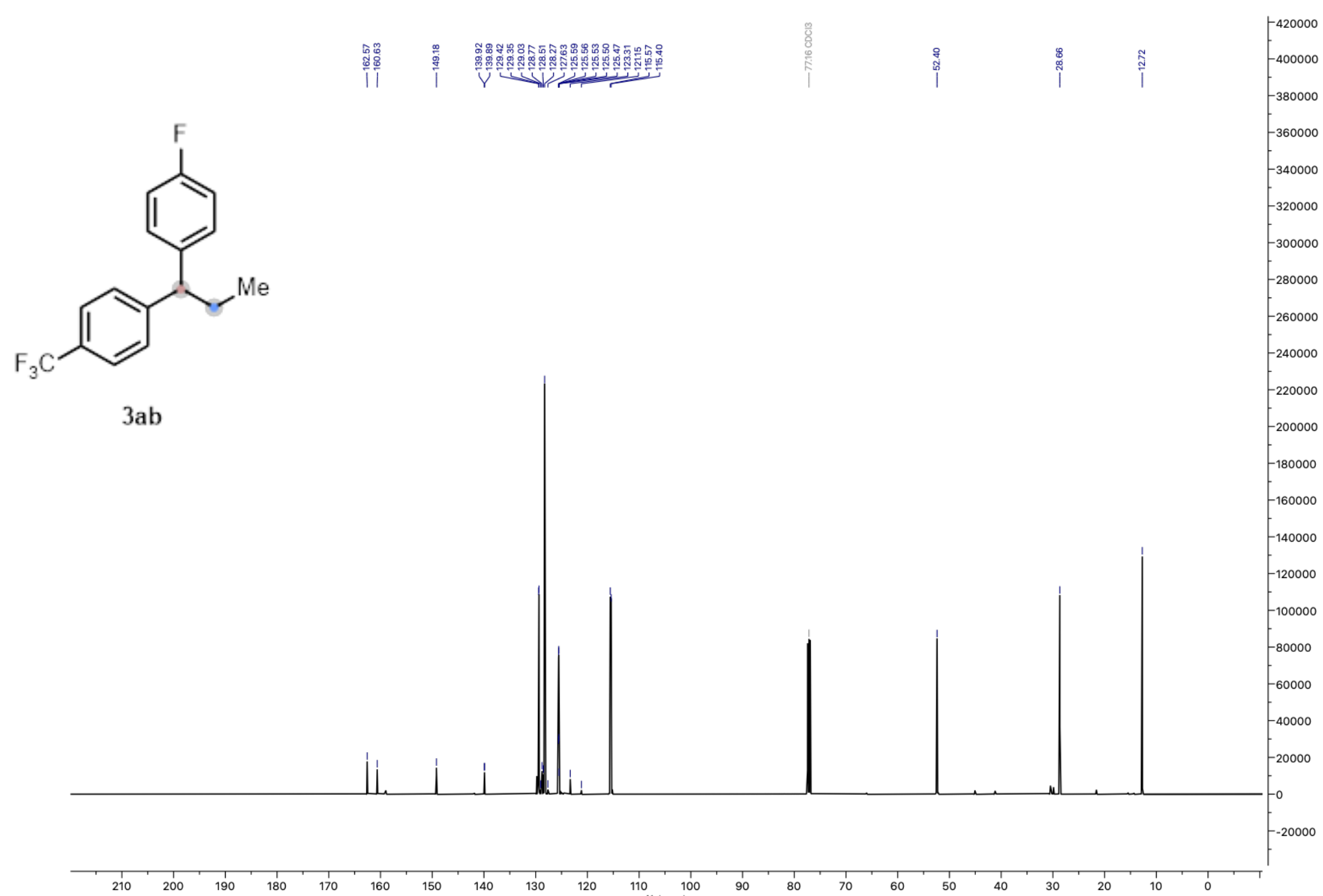

^{19}F NMR (282 MHz; $CDCl_3$):

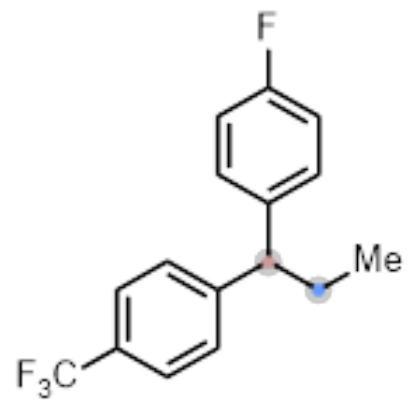

3ab

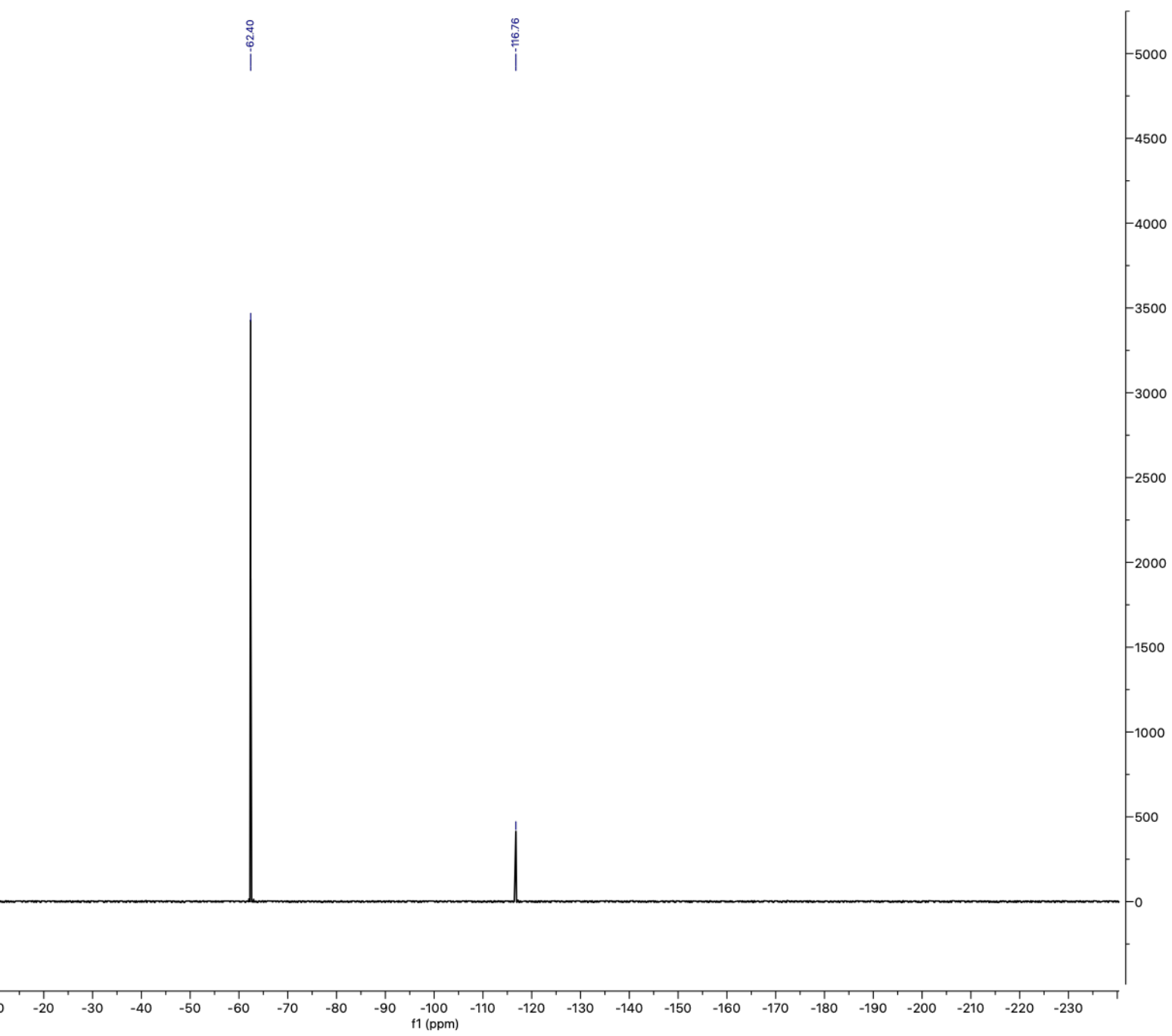

1-(1-(4-Fluorophenyl)propyl)-3-methoxybenzene **3ac**:

^{1}H NMR (500 MHz, $CDCl_3$):

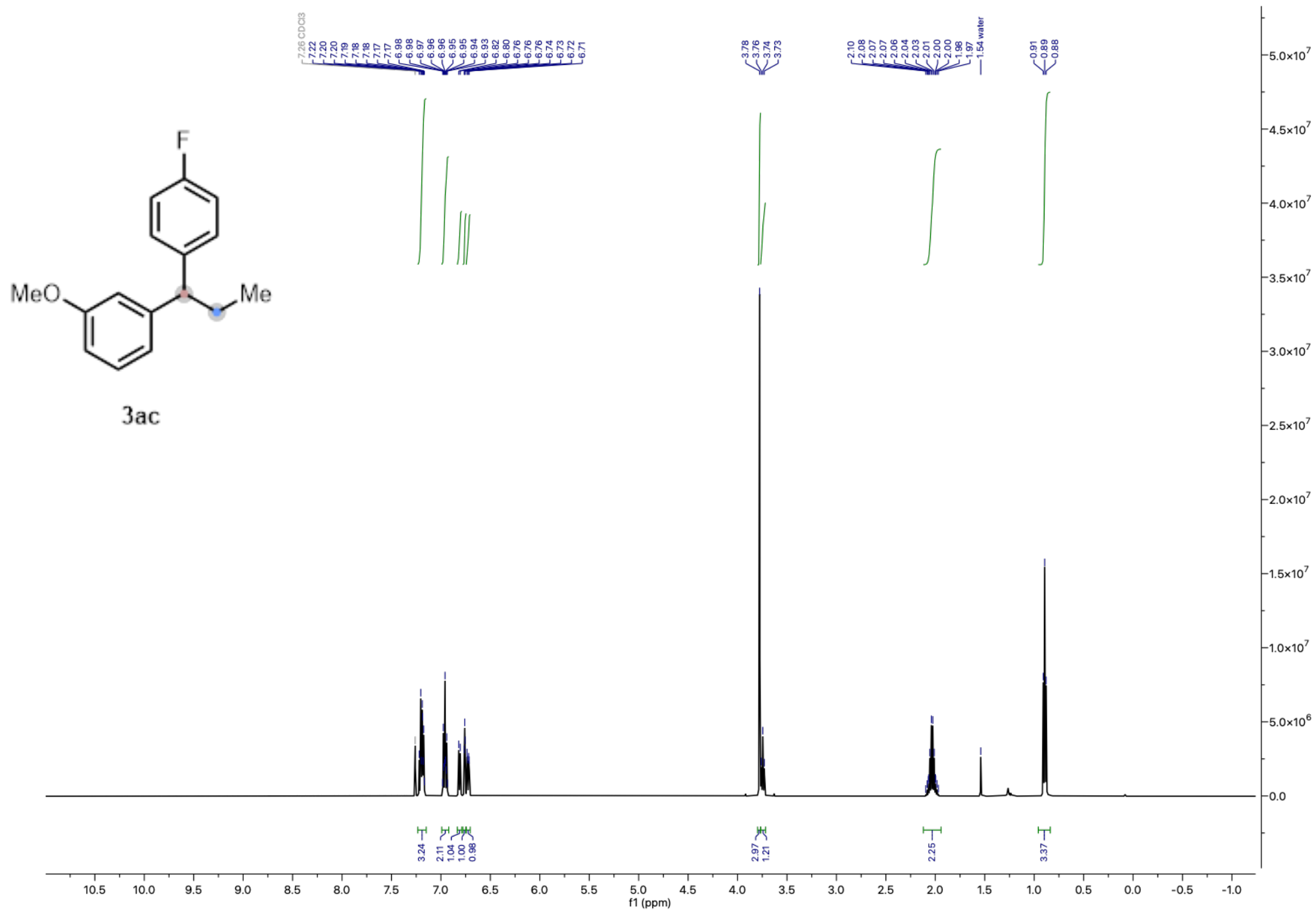

^{13}C NMR (126 MHz, $CDCl_3$):

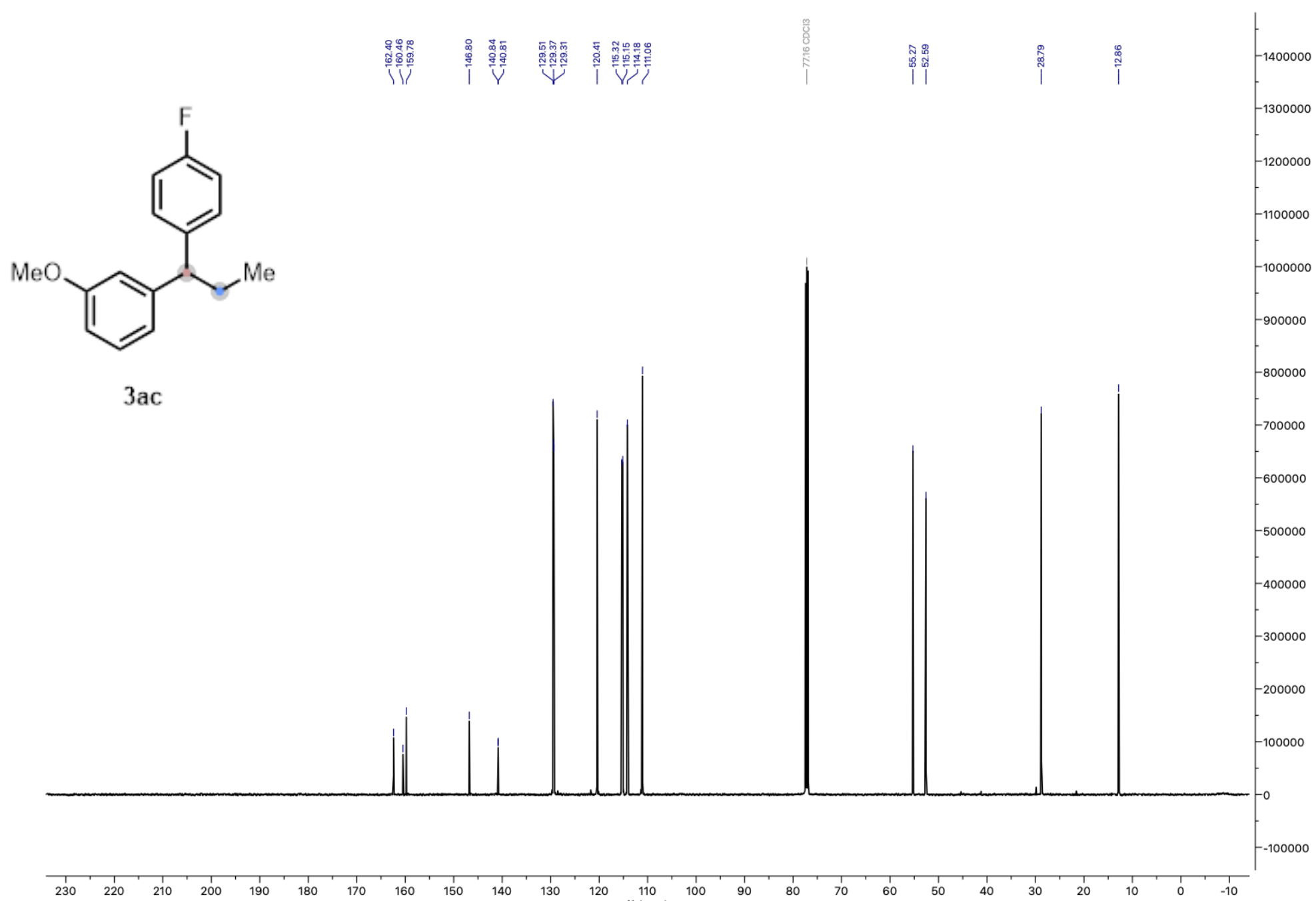

^{19}F NMR (282 MHz, $CDCl_3$)

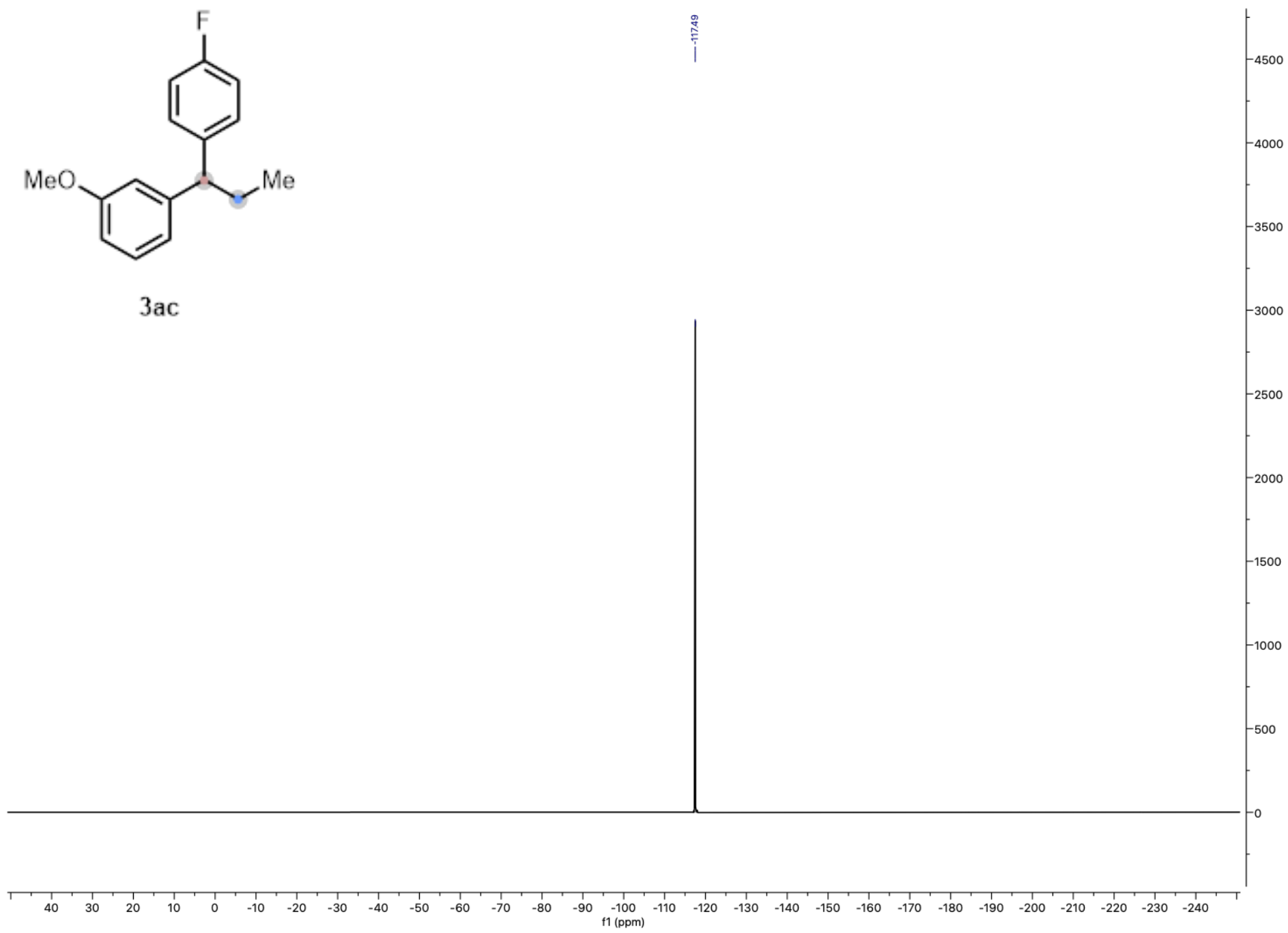

1-(1-(4-Fluorophenyl)propyl)-3-(trifluoromethyl)benzene **3ad**:

^{1}H NMR (500 MHz; $CDCl_3$):

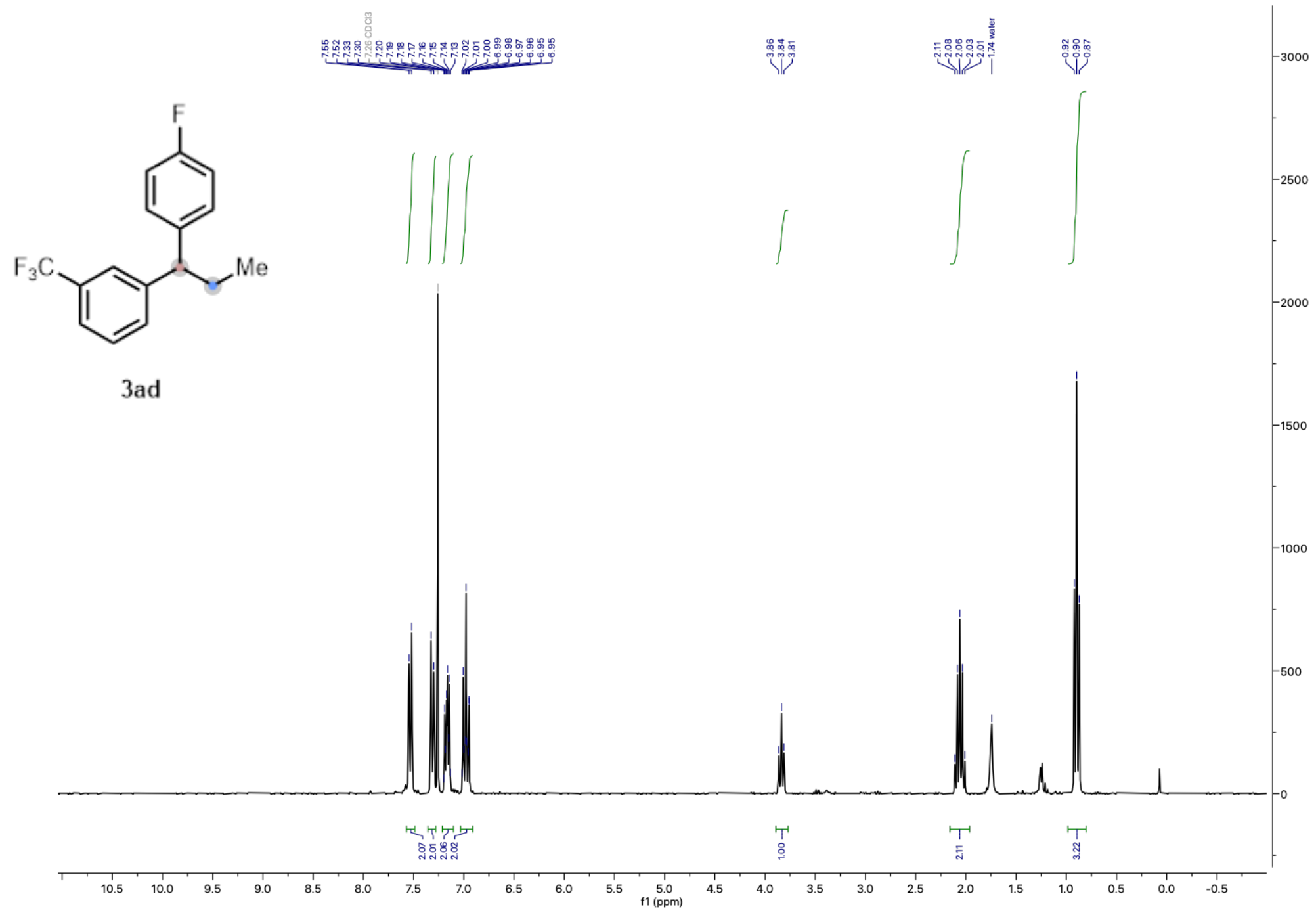

^{13}C NMR (126 MHz; $CDCl_3$):

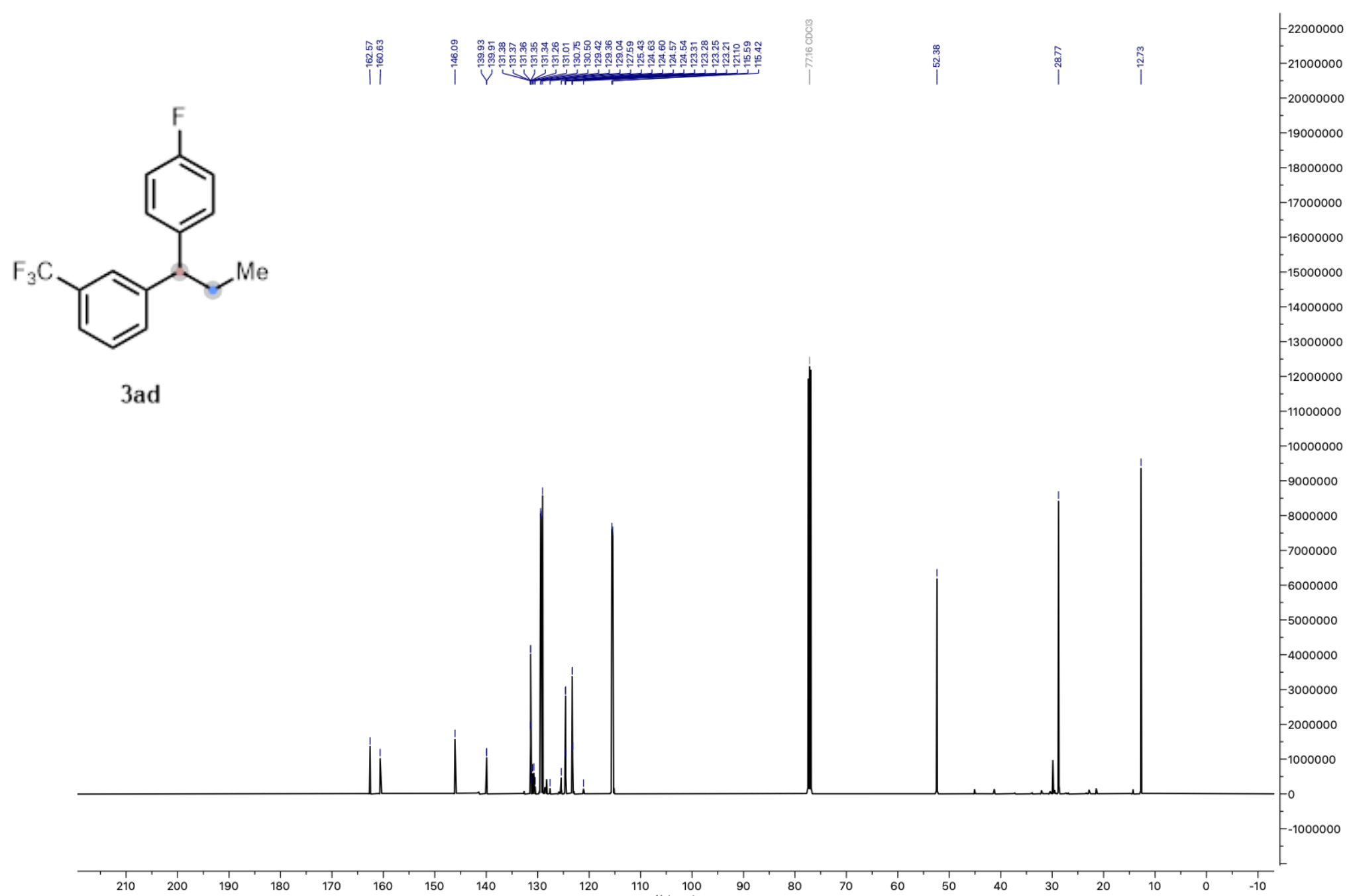

^{19}F NMR (282 MHz; $CDCl_3$):

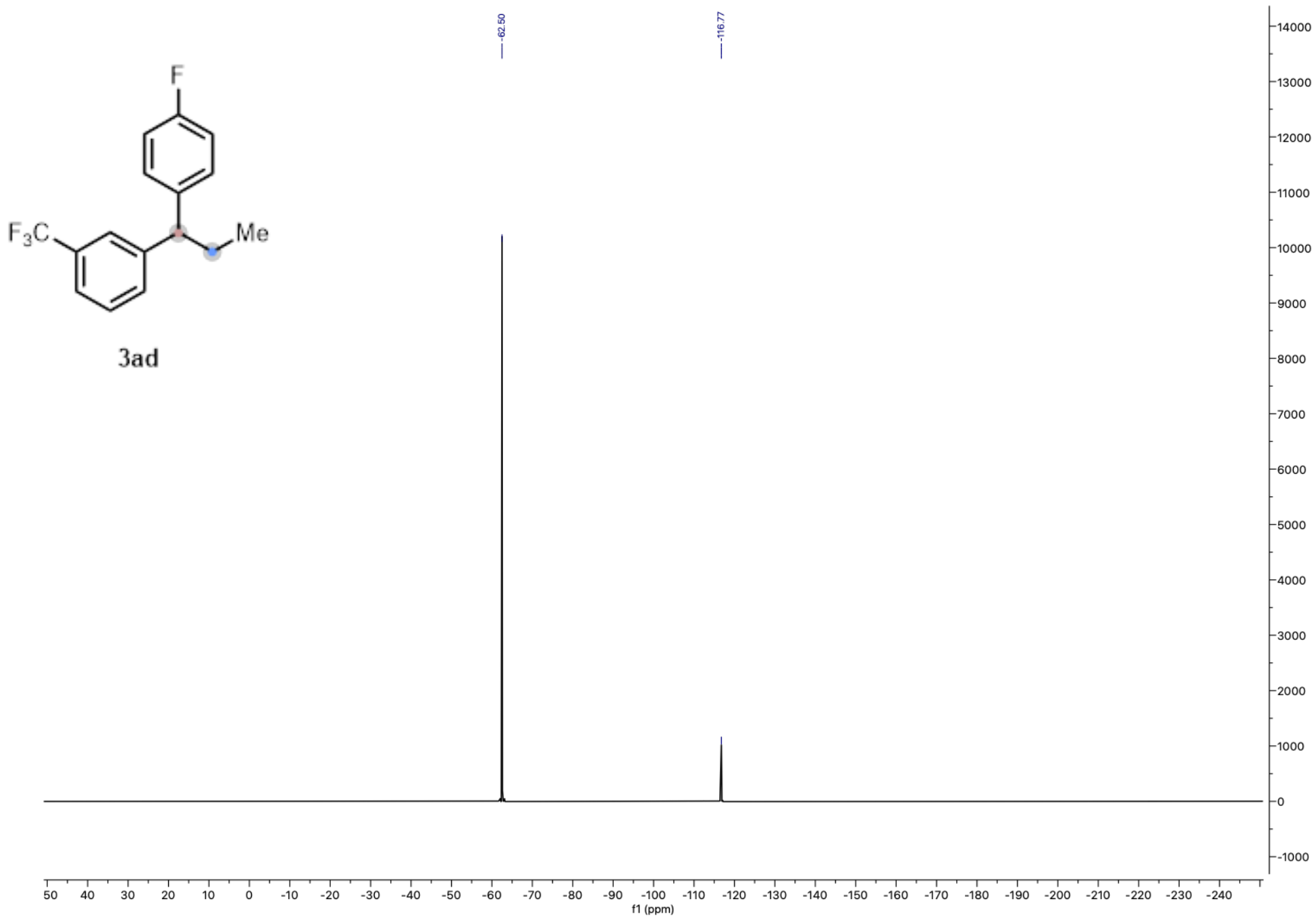

3ad

1-(1-(4-Fluorophenyl)propyl)-2-methoxybenzene **3ae**:

^{1}H NMR (500 MHz, $CDCl_3$):

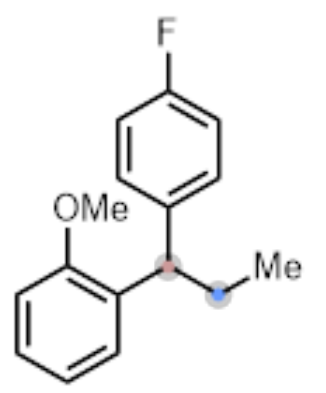

3ae

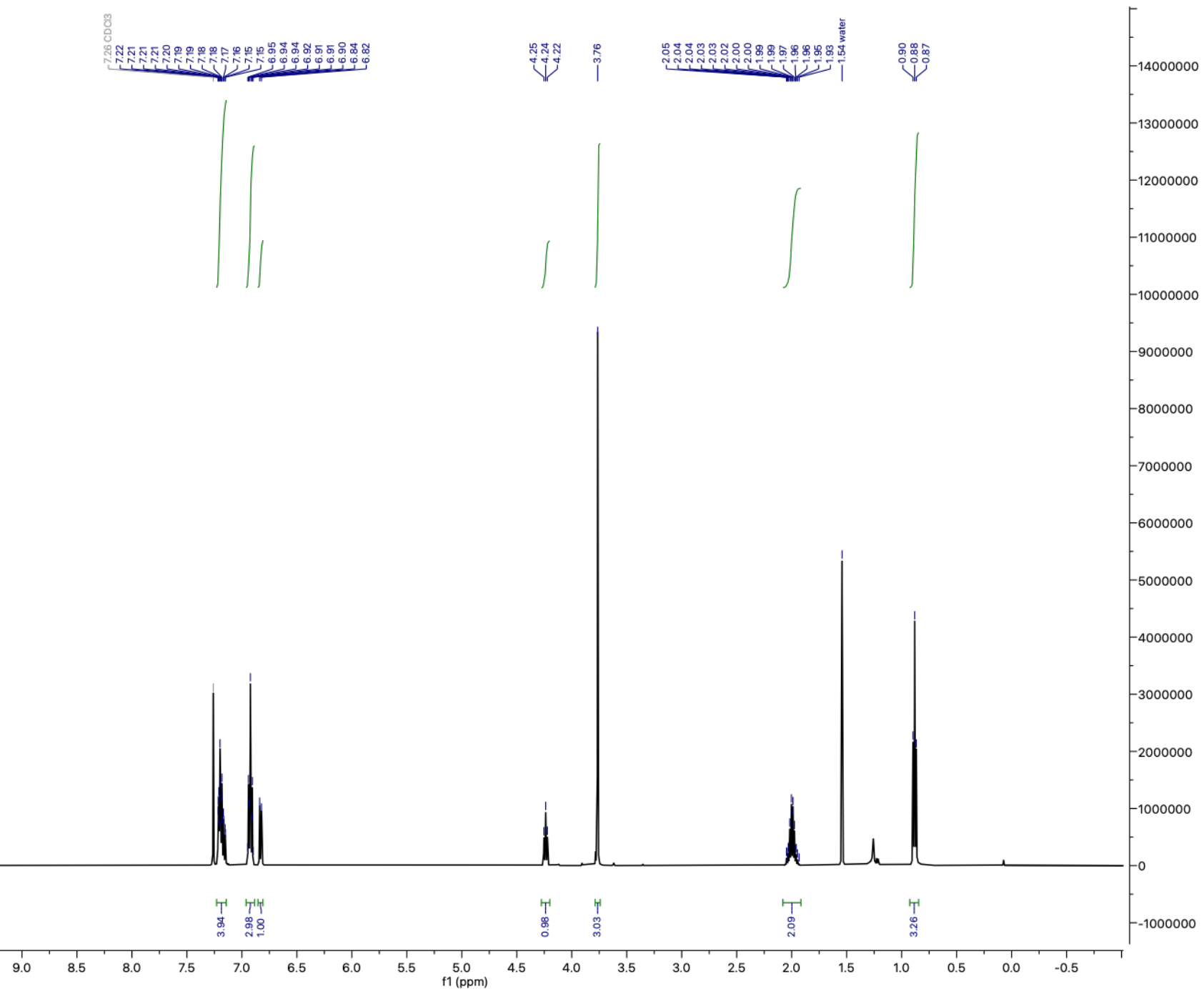

^{13}C NMR (126 MHz, $CDCl_3$):

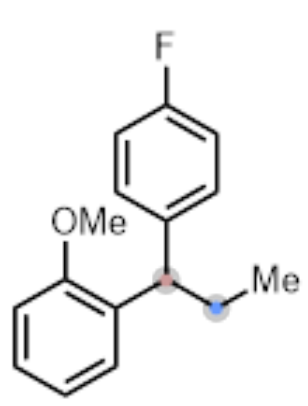

3ae

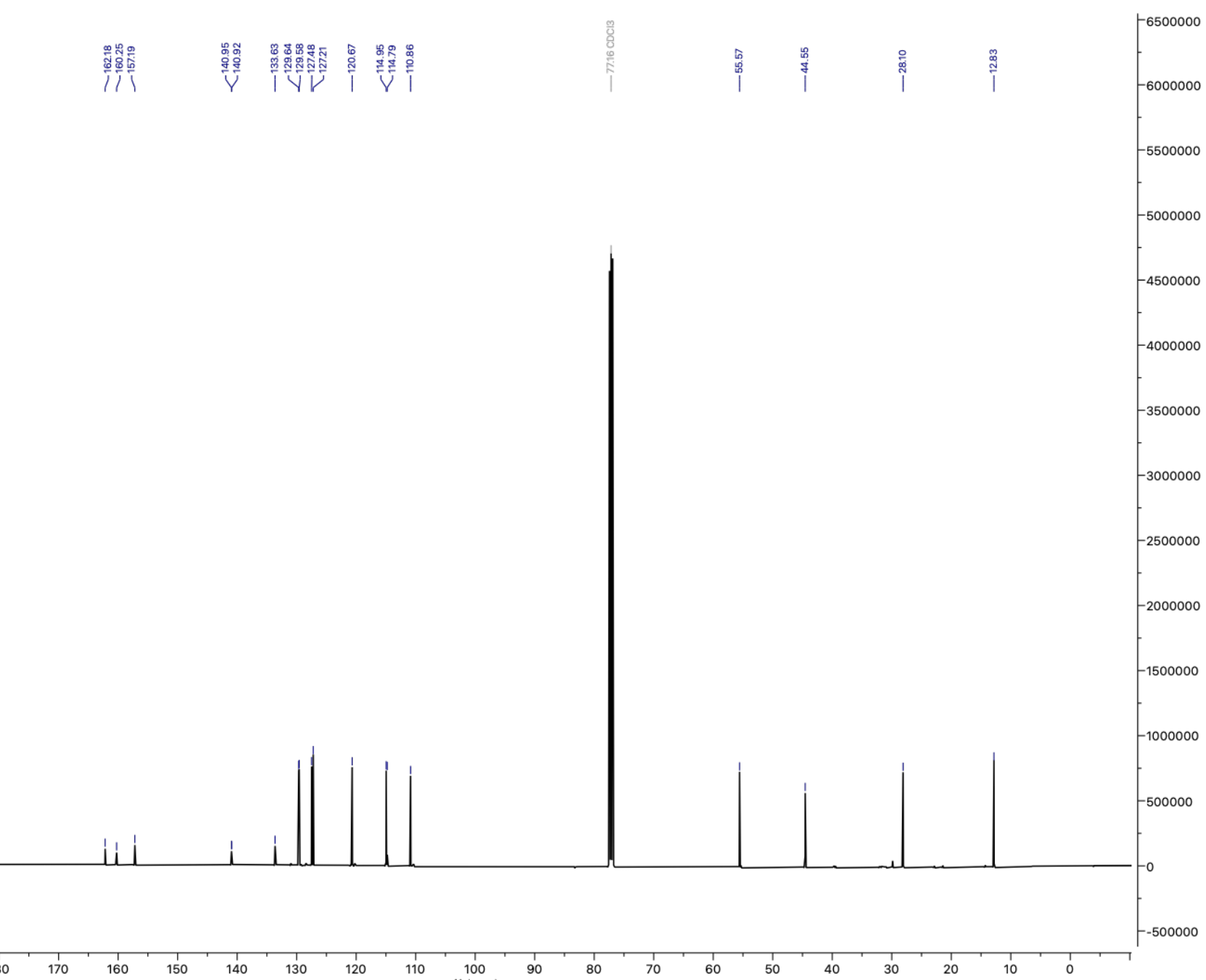

^{19}F NMR (282 MHz, $CDCl_3$):

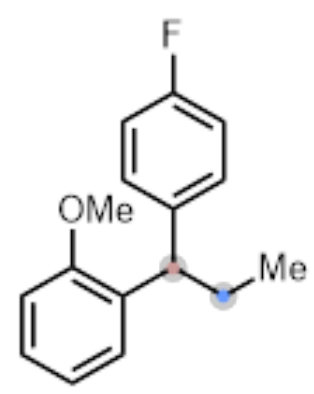

3ae

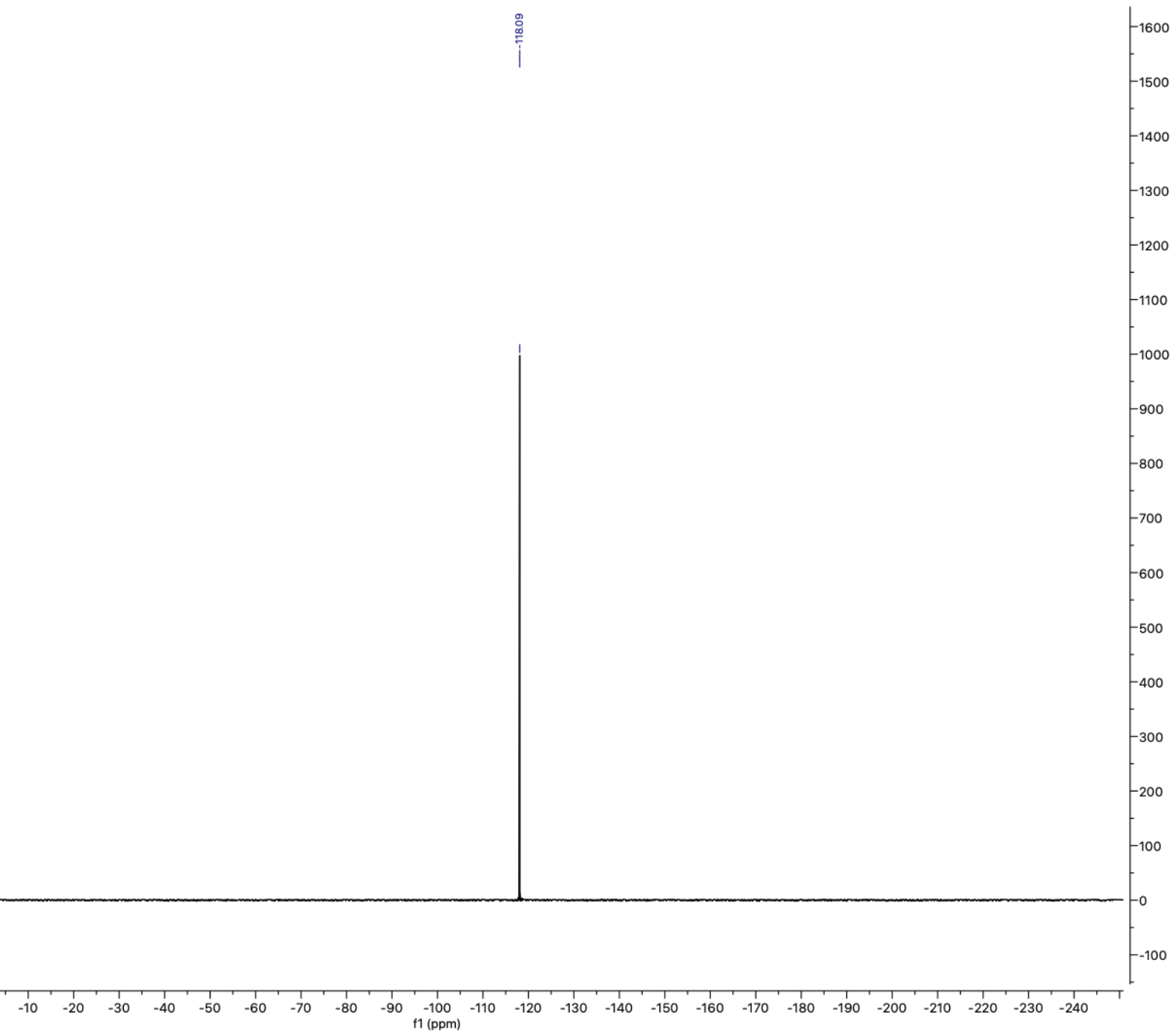

1-(4-Fluorophenyl)-2,3-dihydro-1*H*-indene **3af**:

^{1}H NMR (300 MHz; $CDCl_3$):

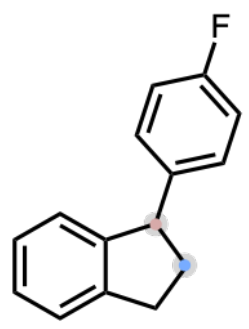

3af

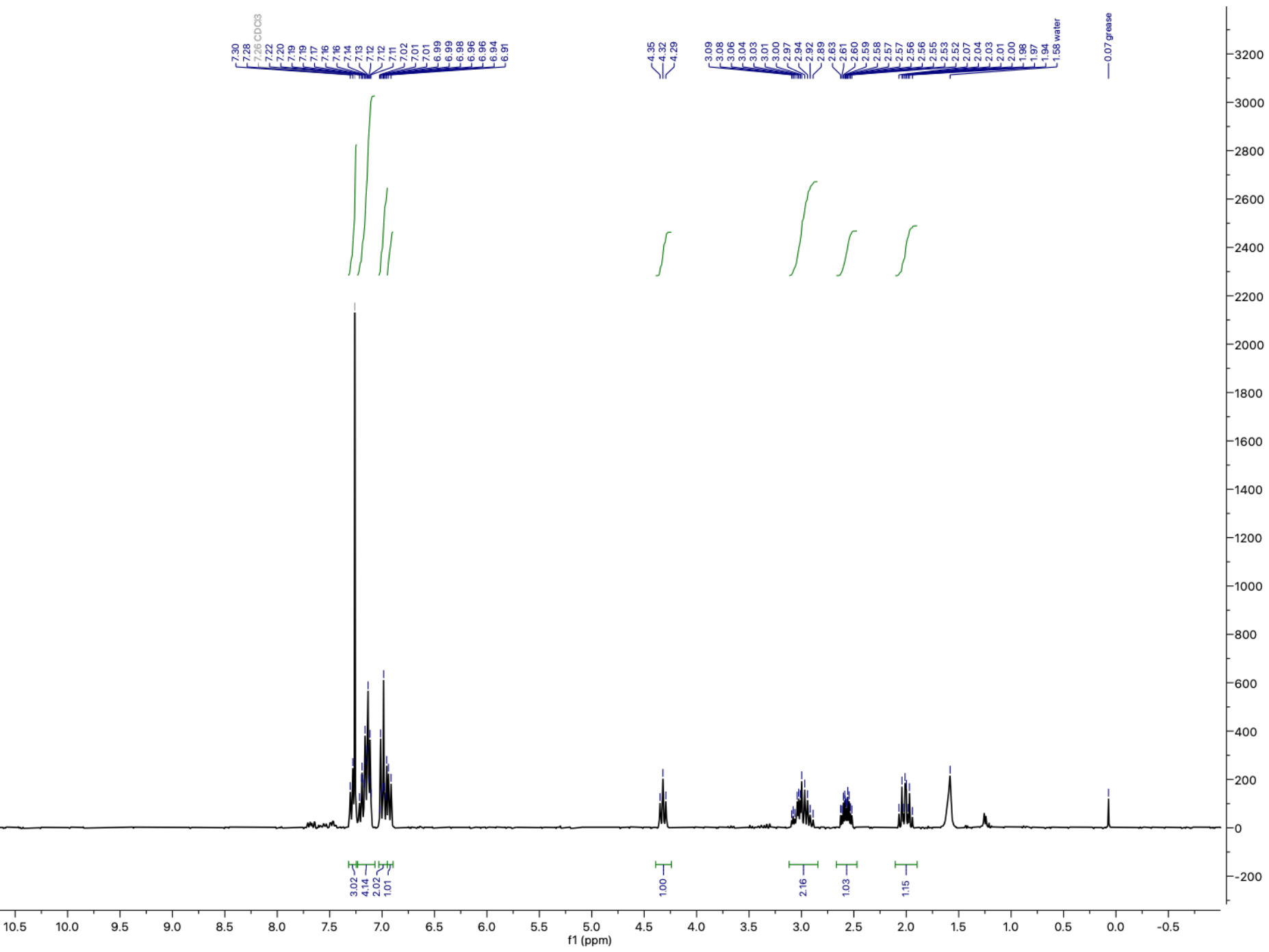

^{13}C NMR (126 MHz; $CDCl_3$):

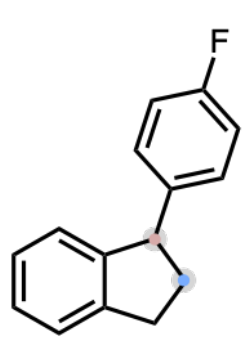

3af

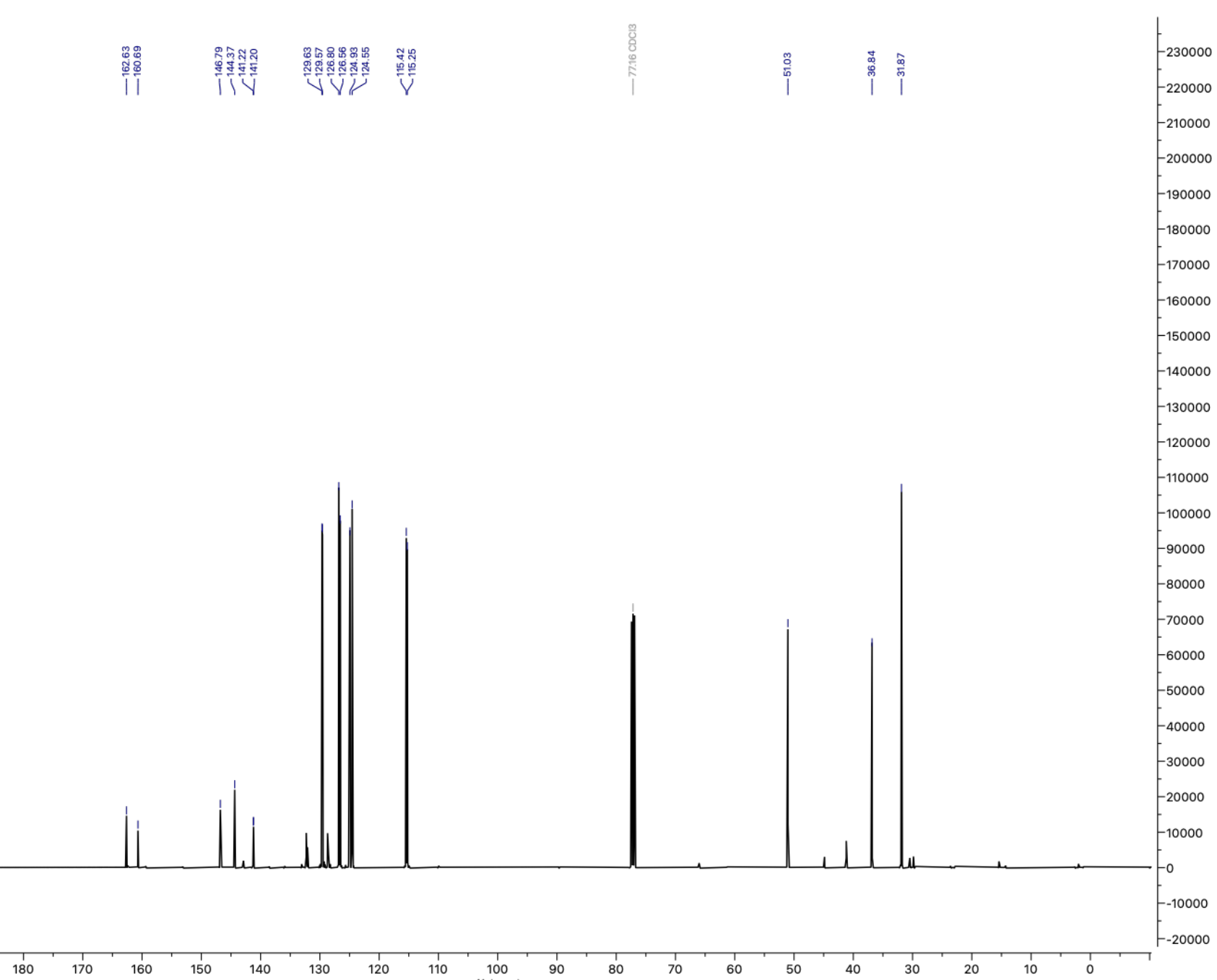

^{19}F NMR (282 MHz; $CDCl_3$):

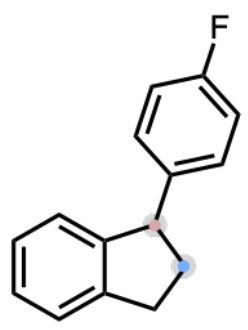

3af

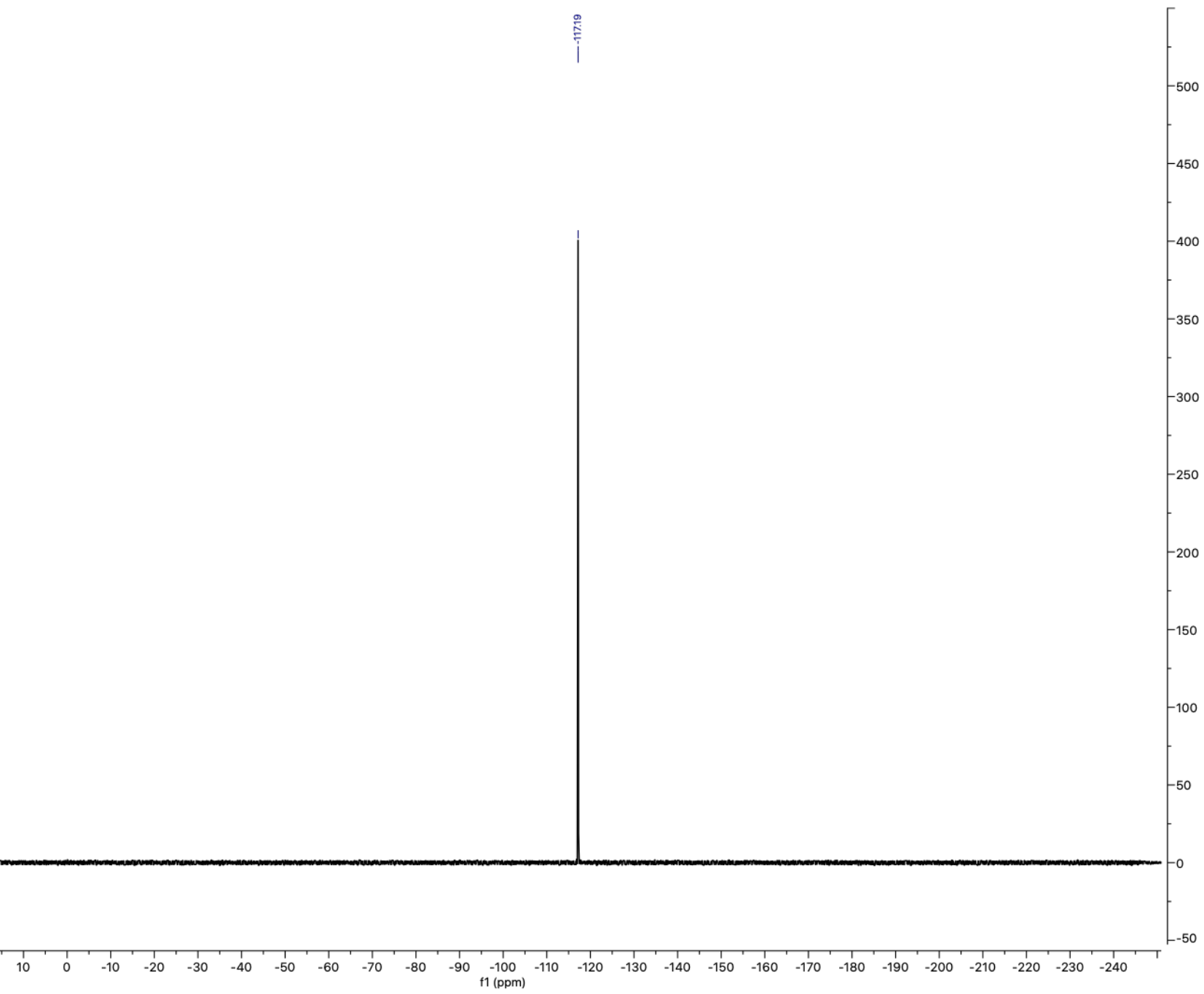

1-(4-Fluorophenyl)-1,2,3,4-tetrahydronaphthalene **3ag**:

^{1}H NMR (300 MHz, $CDCl_3$):

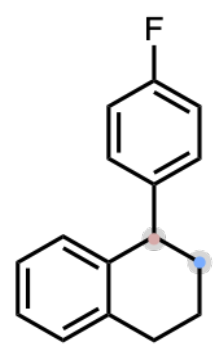

3ag

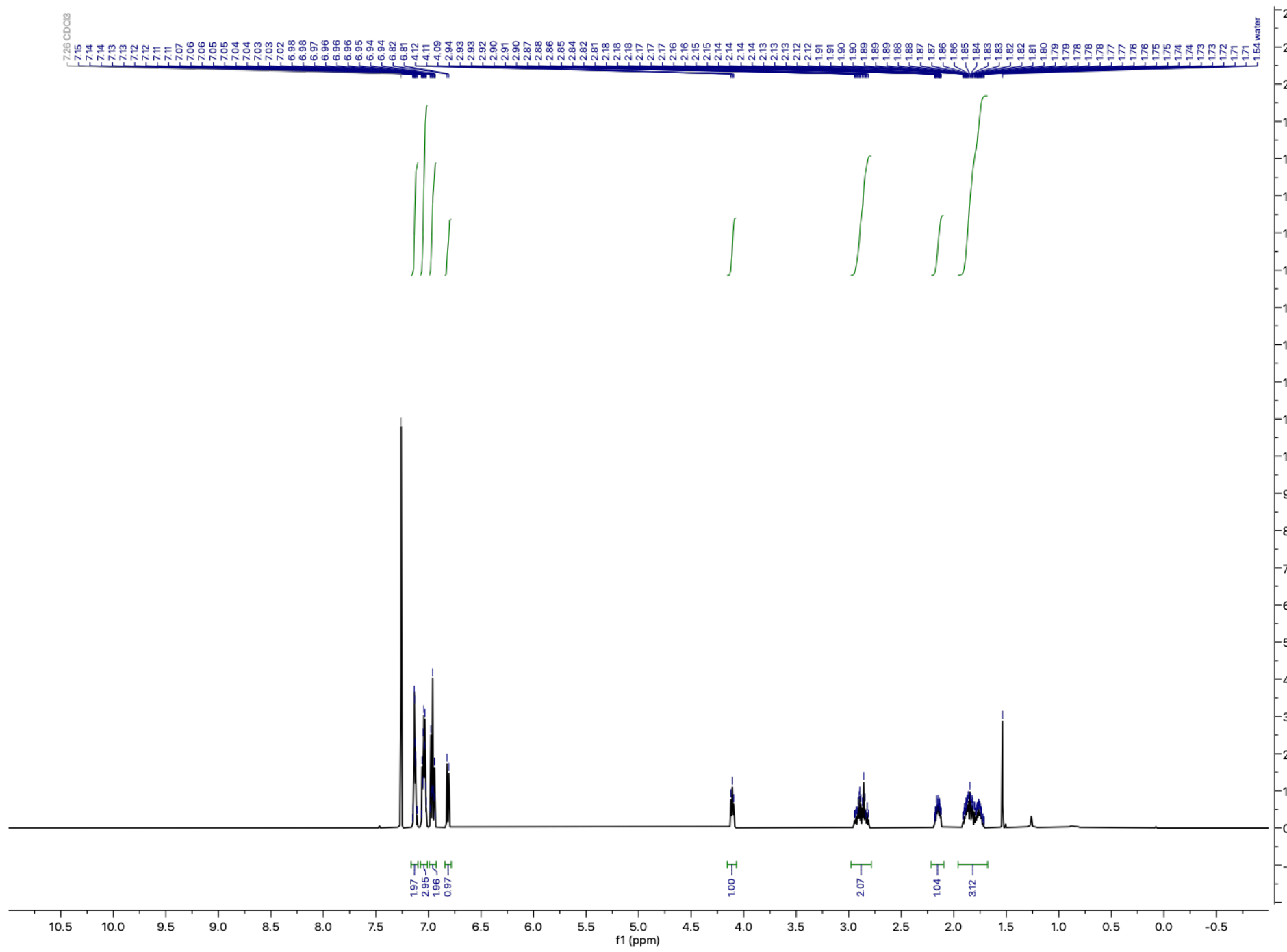

^{13}C NMR (126 MHz, $CDCl_3$):

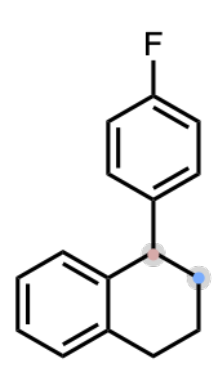

3ag

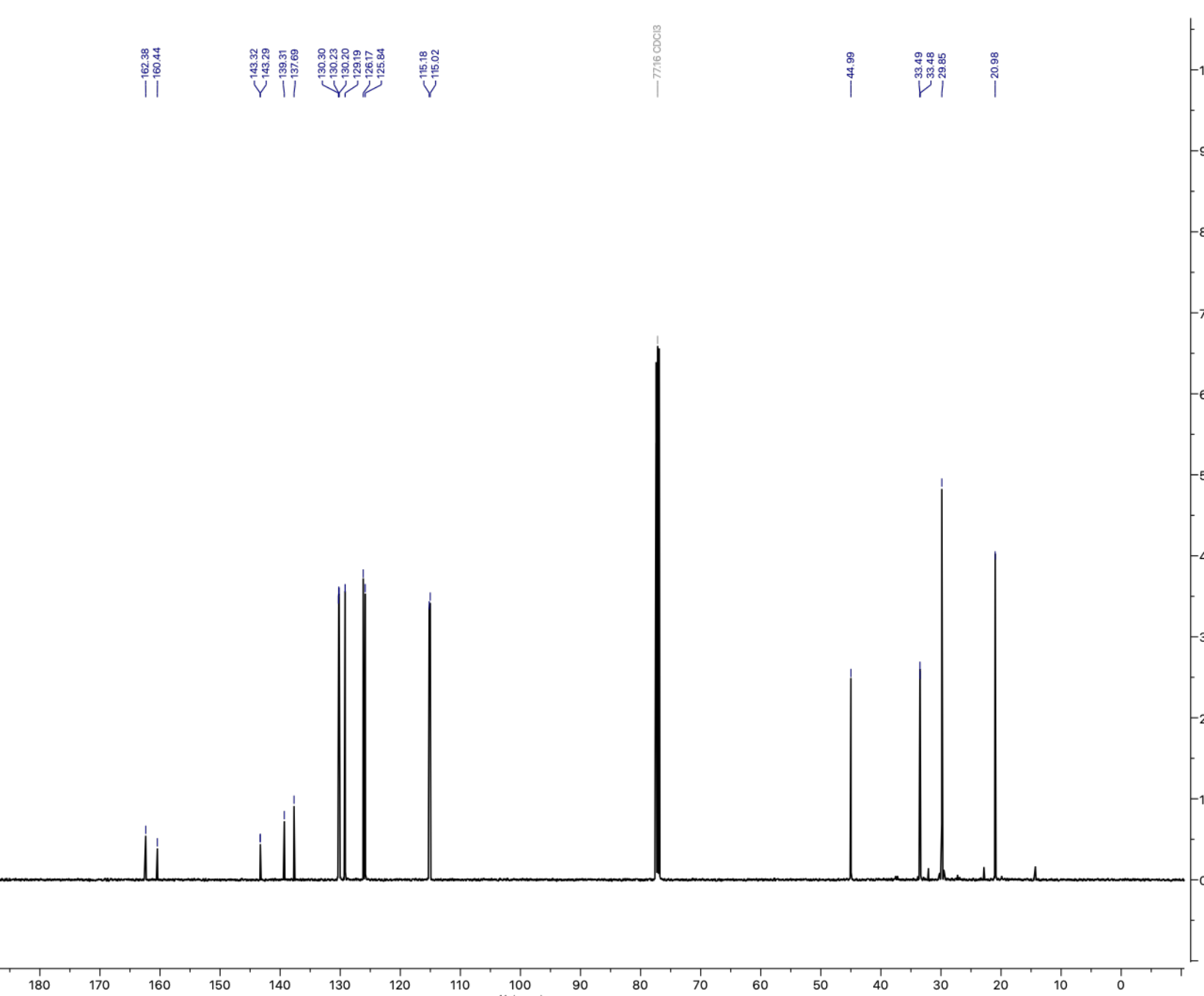

^{19}F NMR (282 MHz, CDCl$_3$):

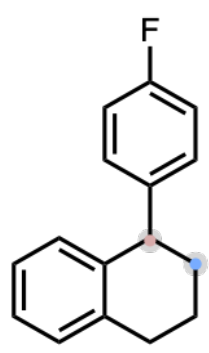

3ag

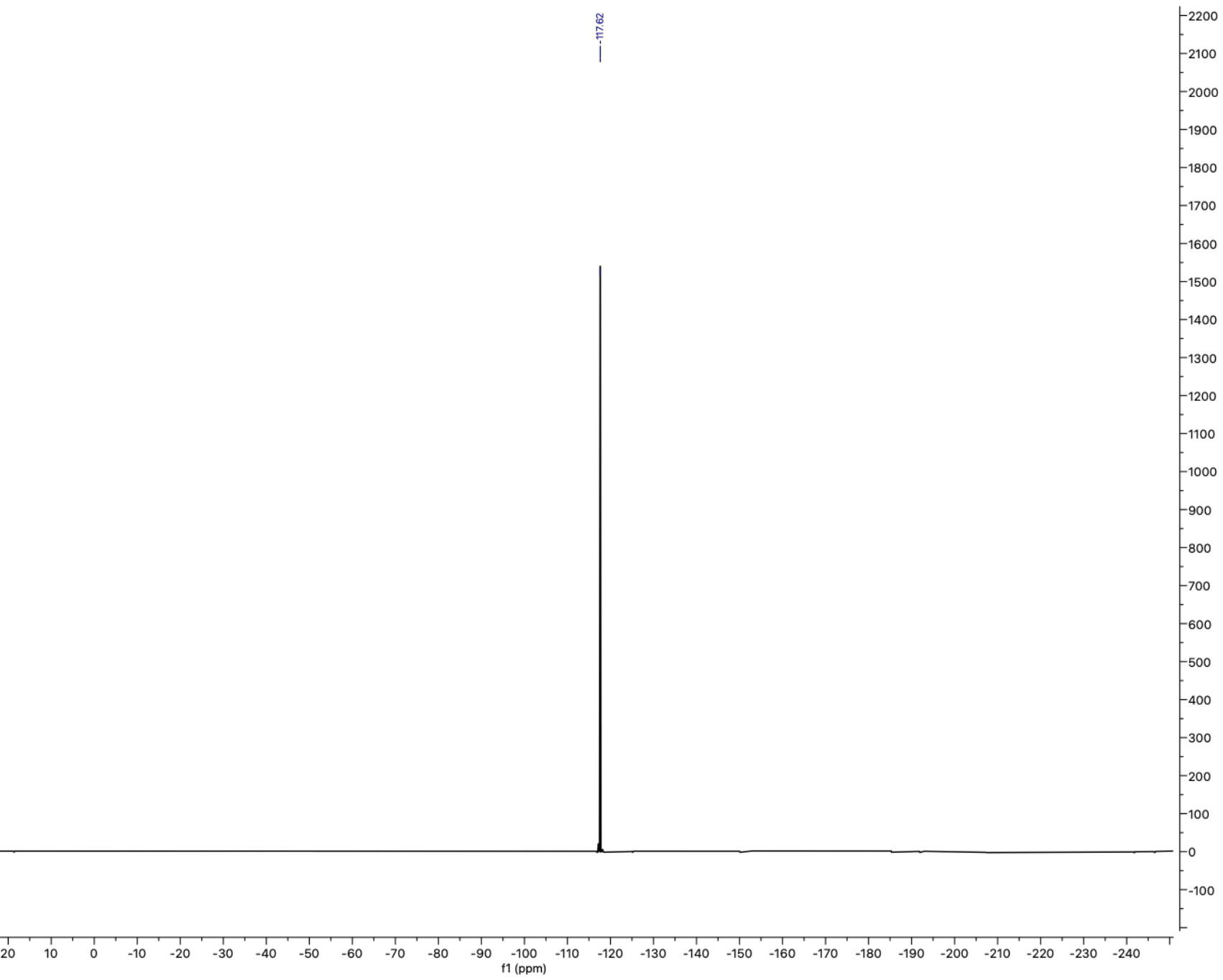

1-(4-Fluorophenyl)-5-methoxy-1,2,3,4-tetrahydronaphthalene **3ah**:

^{1}H NMR (500 MHz, $CDCl_3$):

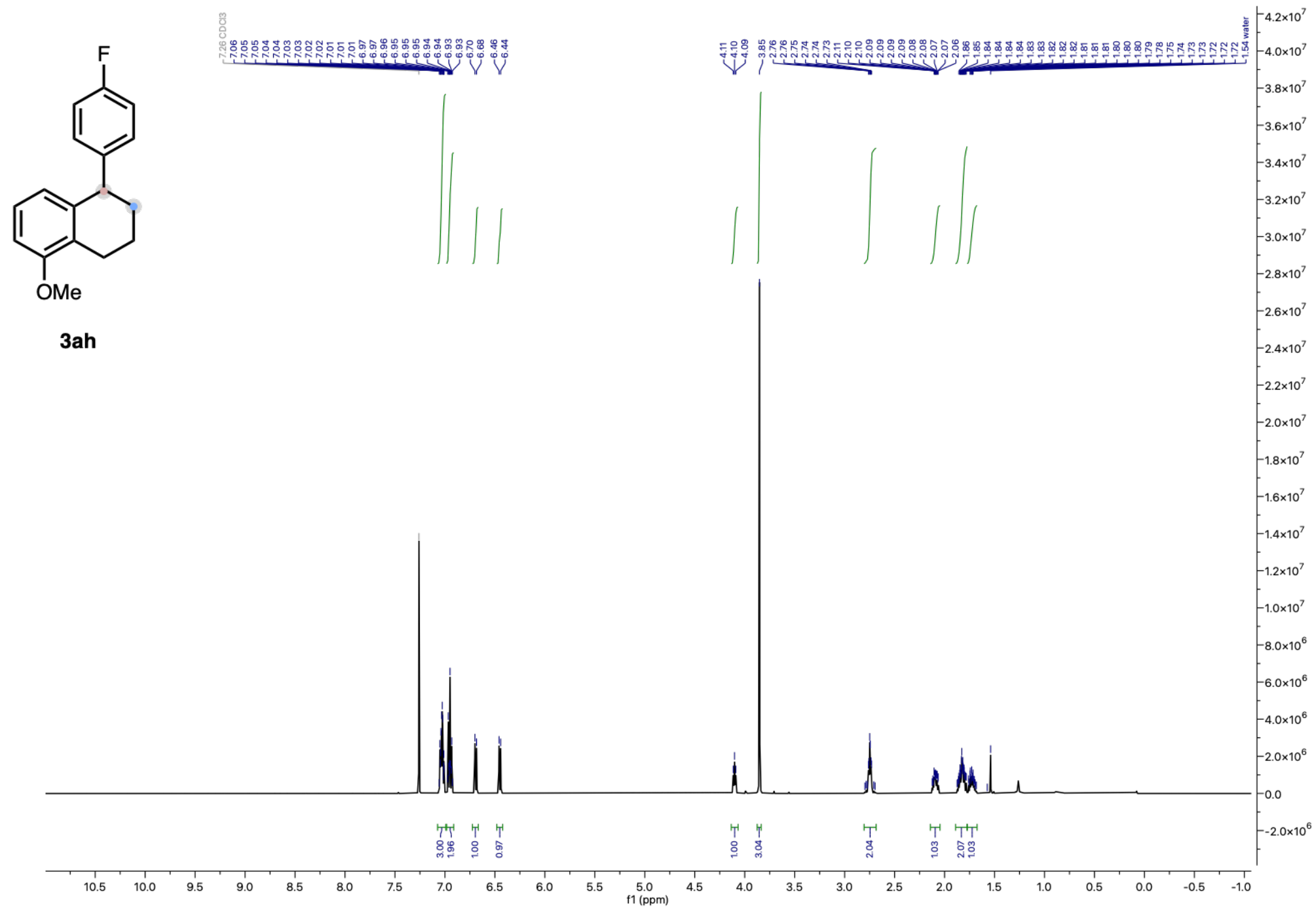

^{13}C NMR (126 MHz, $CDCl_3$):

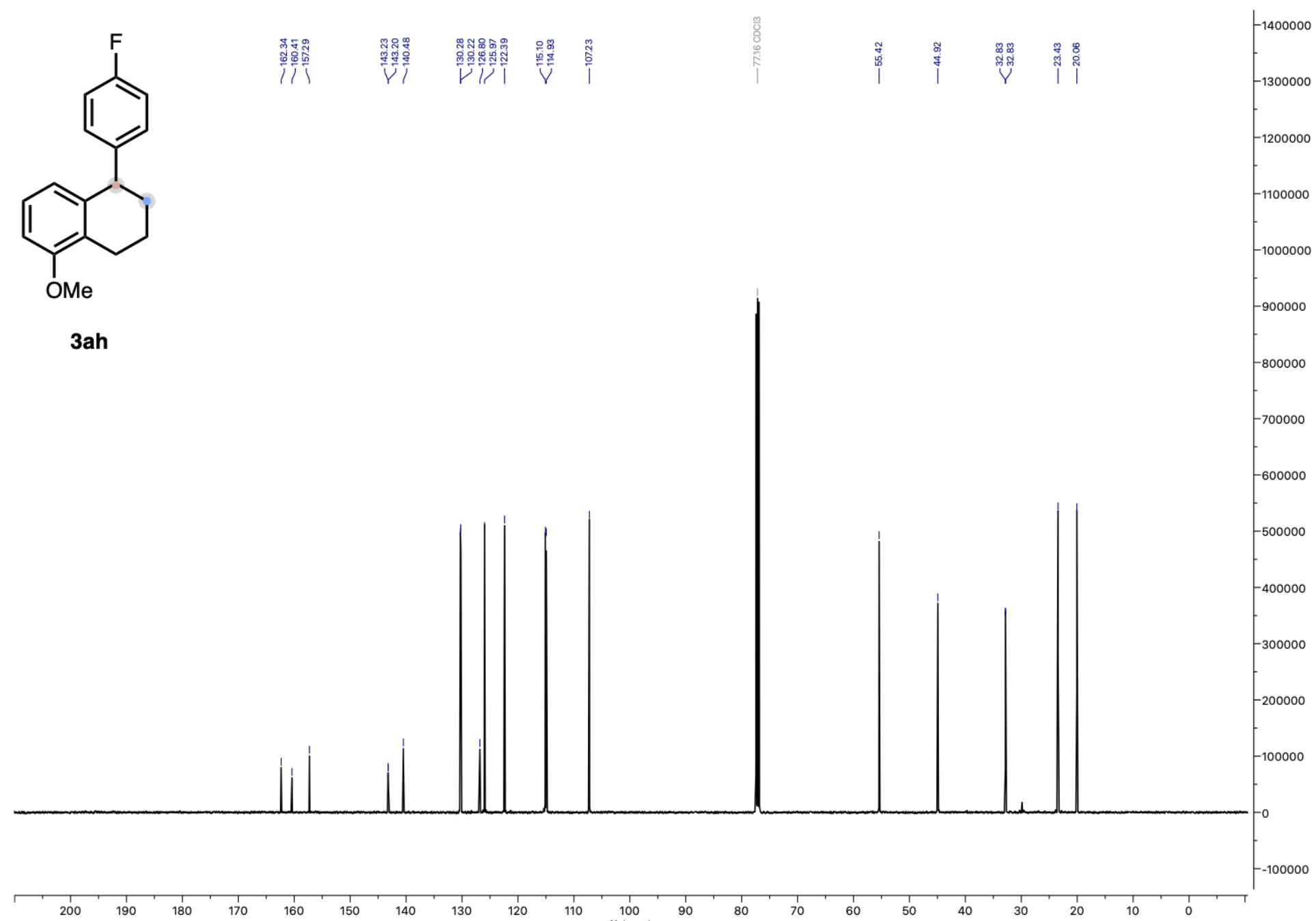

^{19}F NMR (282 MHz, $CDCl_3$):

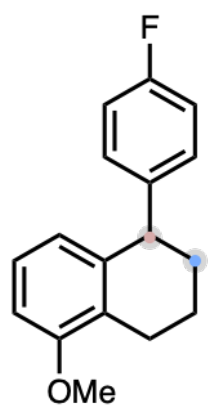

3ah

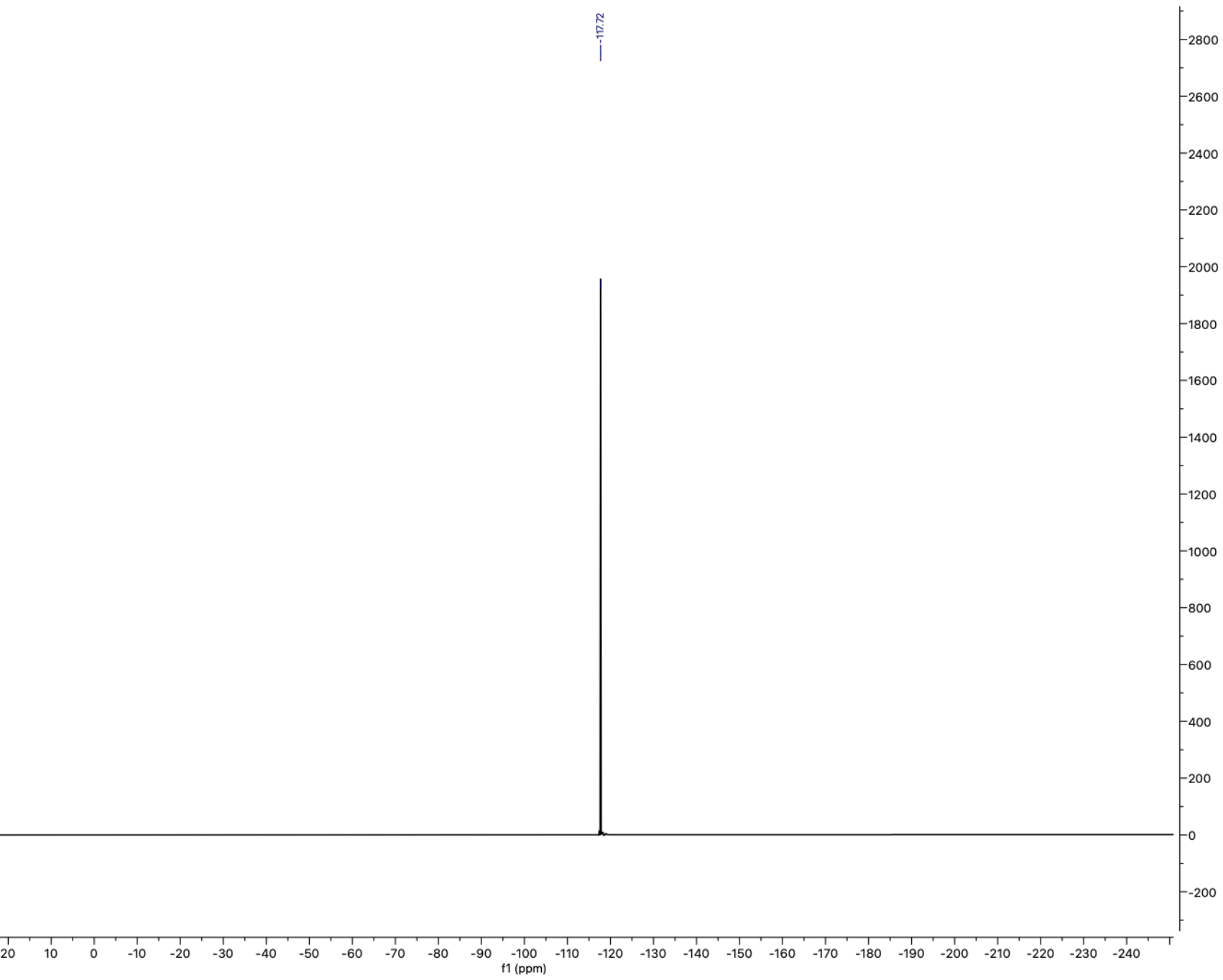

2-(1-(4-methoxyphenyl)propyl)furan **3ai**:

^{1}H NMR (500 MHz, $CDCl_3$):

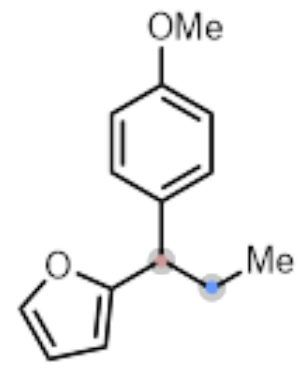

3ai

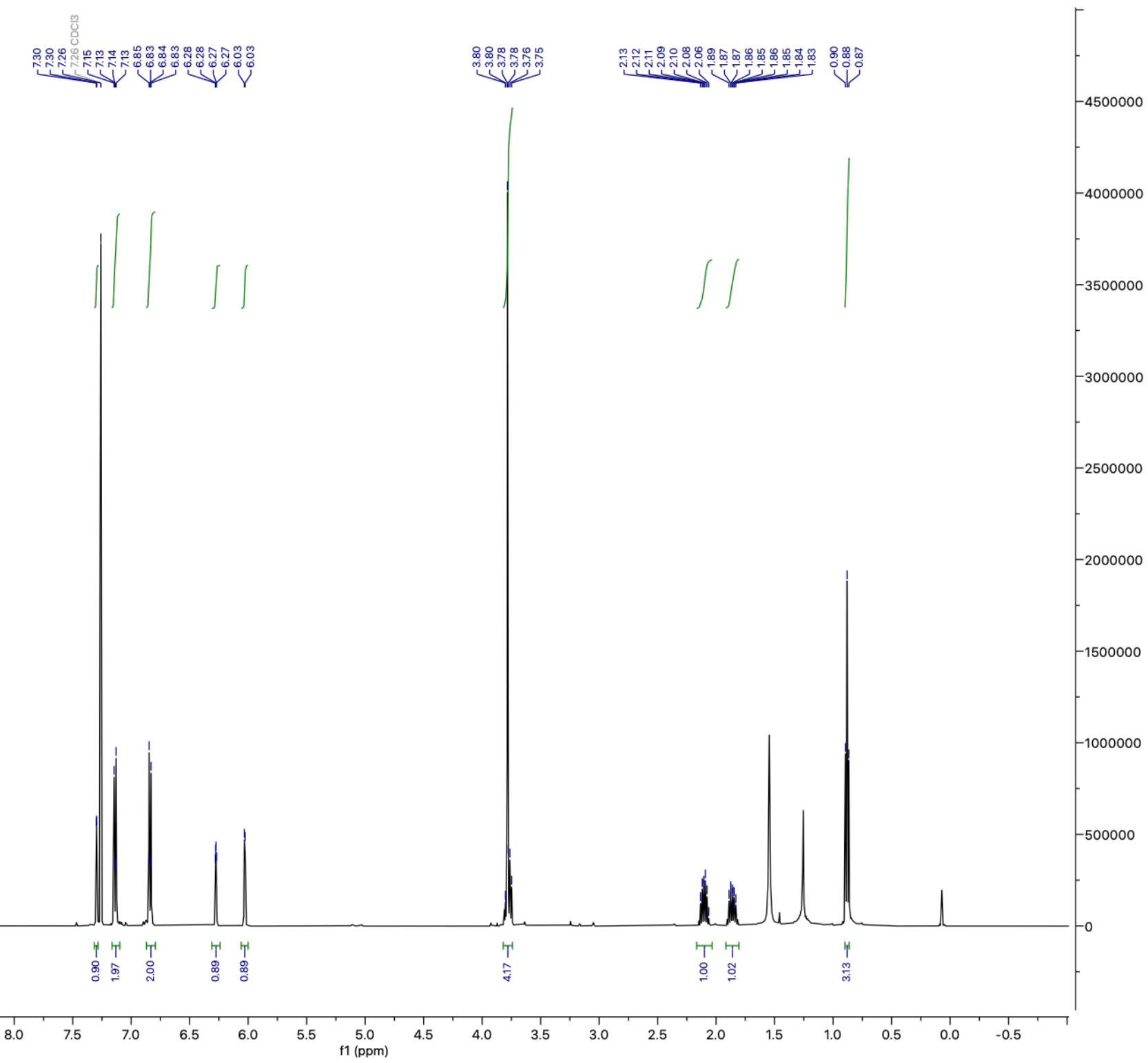

^{13}C NMR (126 MHz, $CDCl_3$):

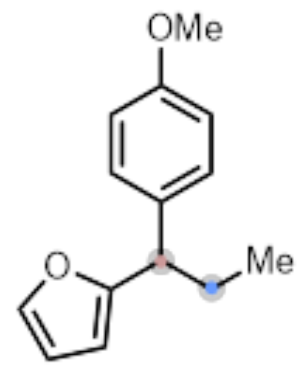

3ai

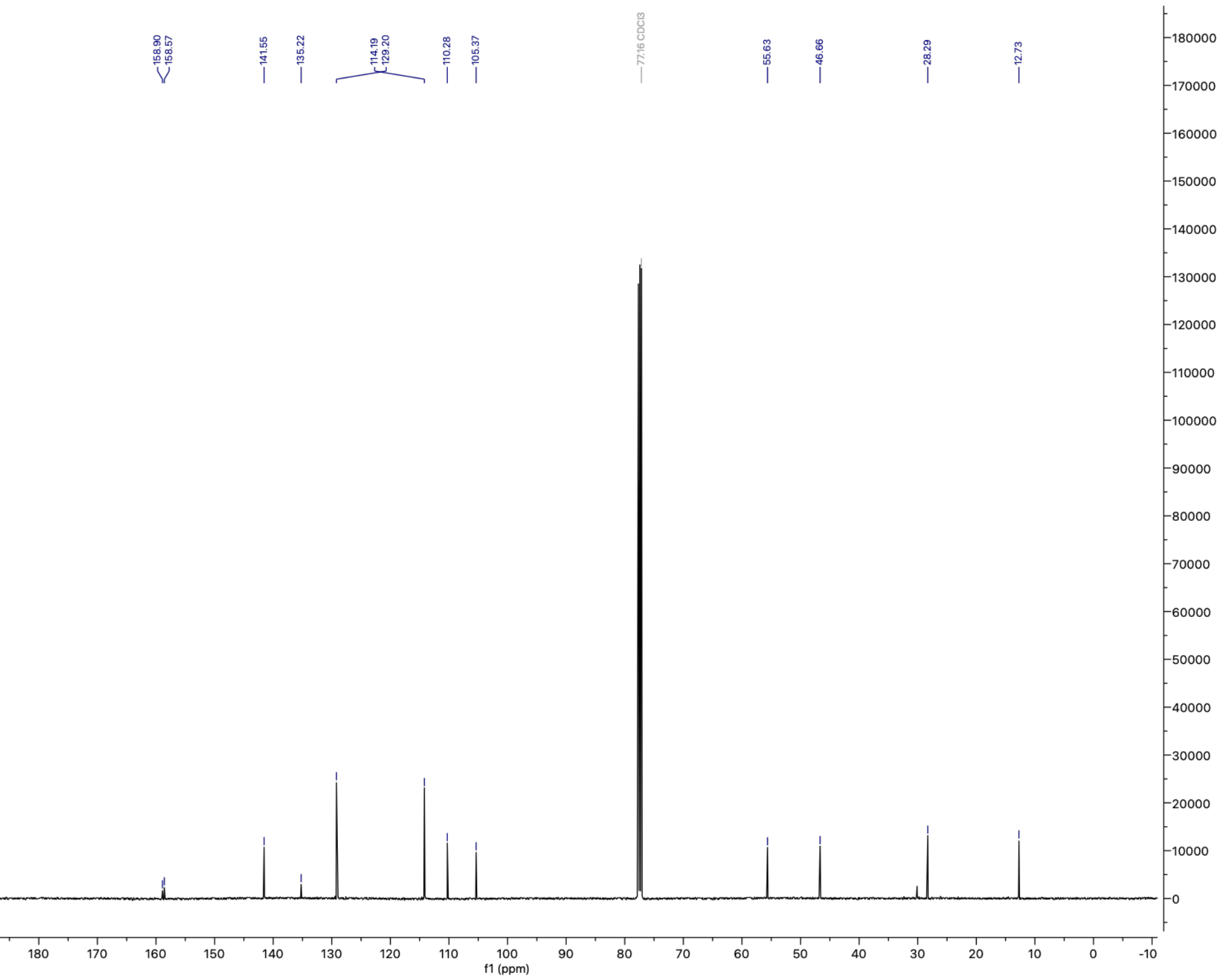

2-(1-(4-fluorophenyl)propyl)thiophene **3aj**:

^{1}H NMR (500 MHz, $CDCl_3$):

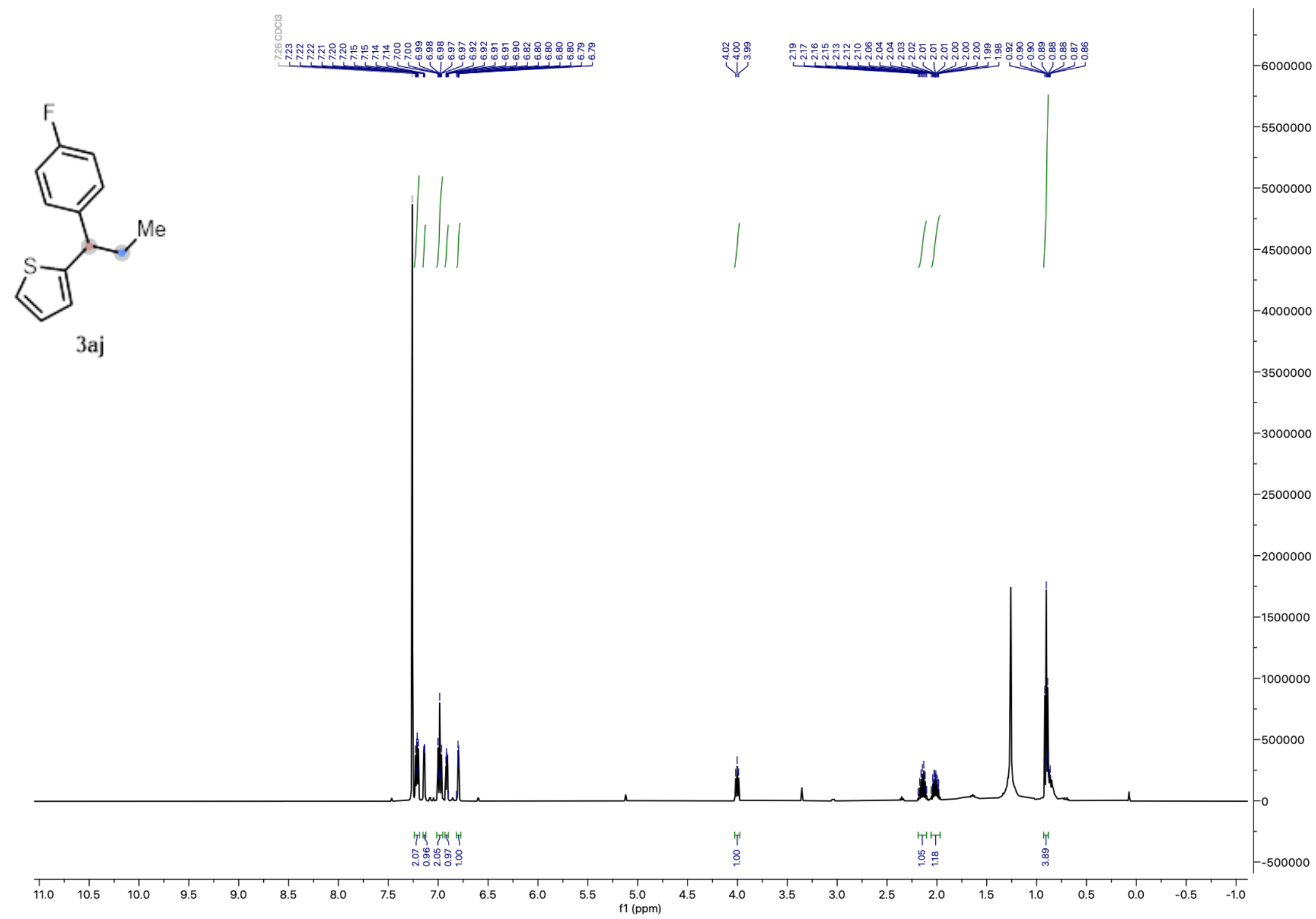

^{13}C NMR (126 MHz, $CDCl_3$):

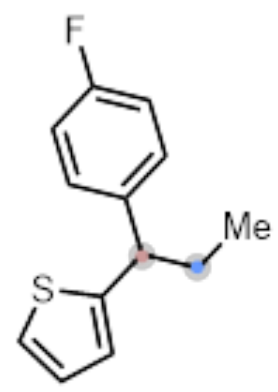

3aj

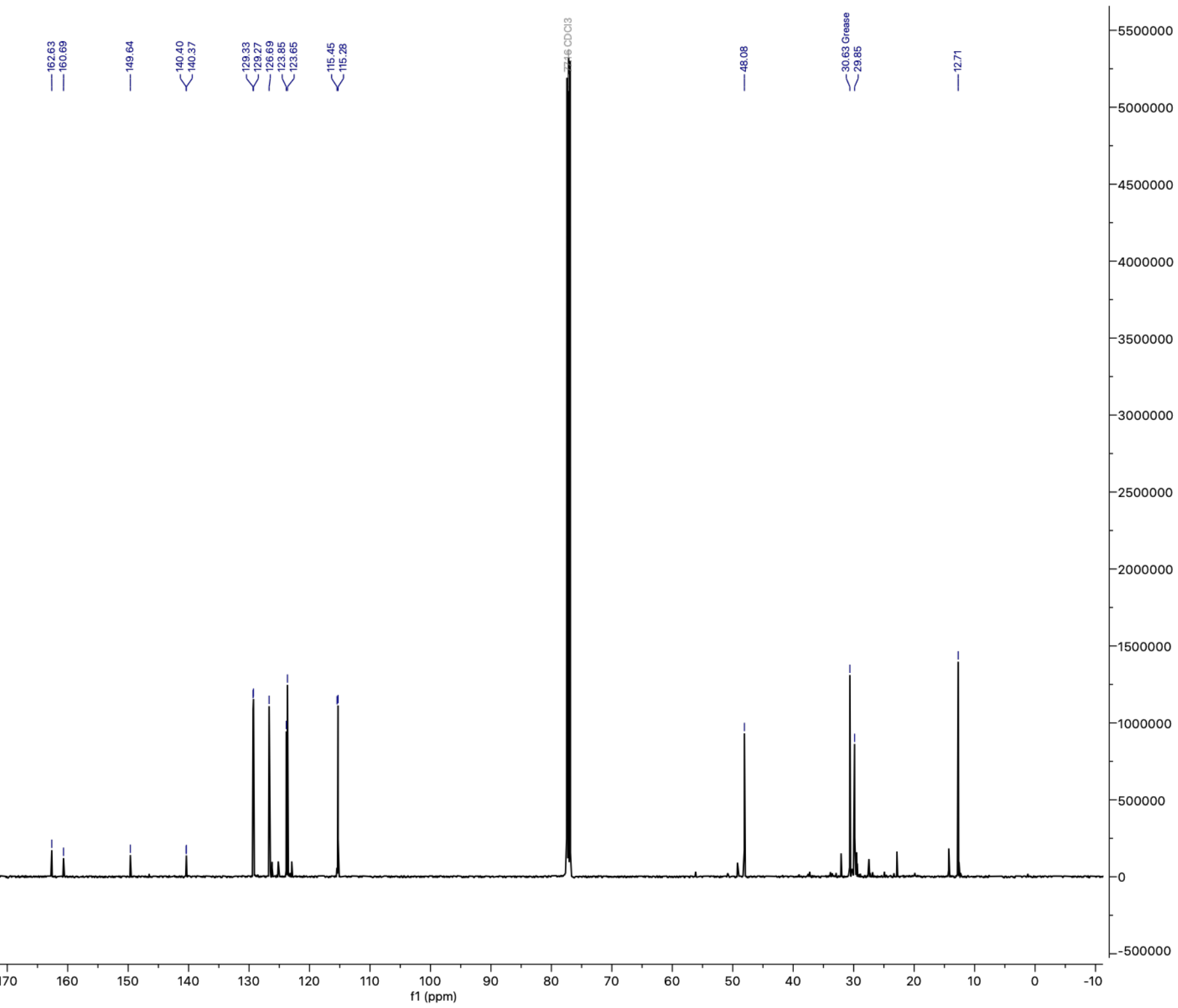

3-(1-(4-fluorophenyl)propyl)benzofuran **3ak**:

^{1}H NMR (500 MHz, $CDCl_3$):

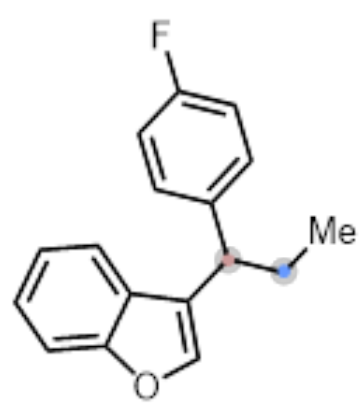

3ak

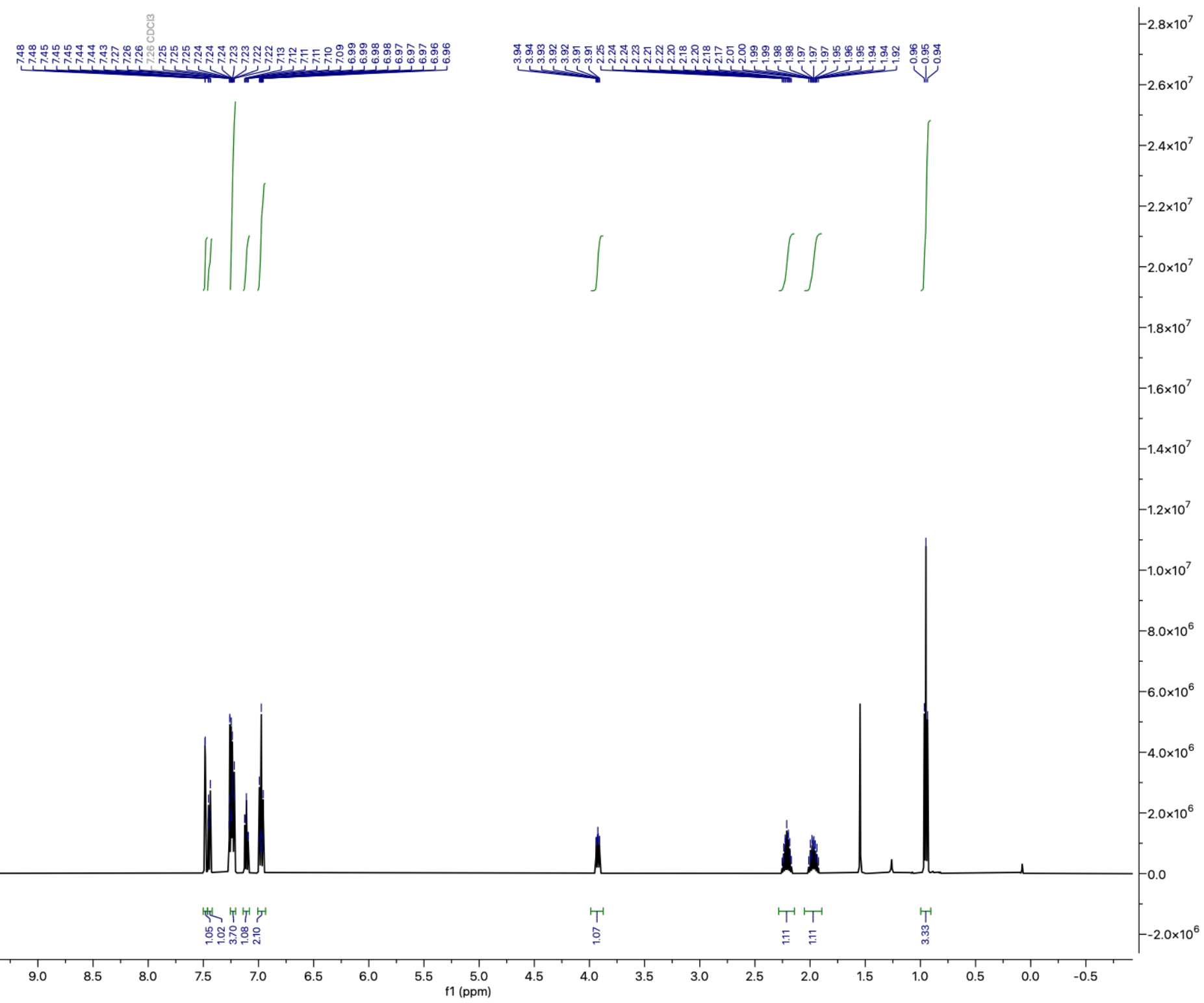

^{13}C NMR (126 MHz, $CDCl_3$):

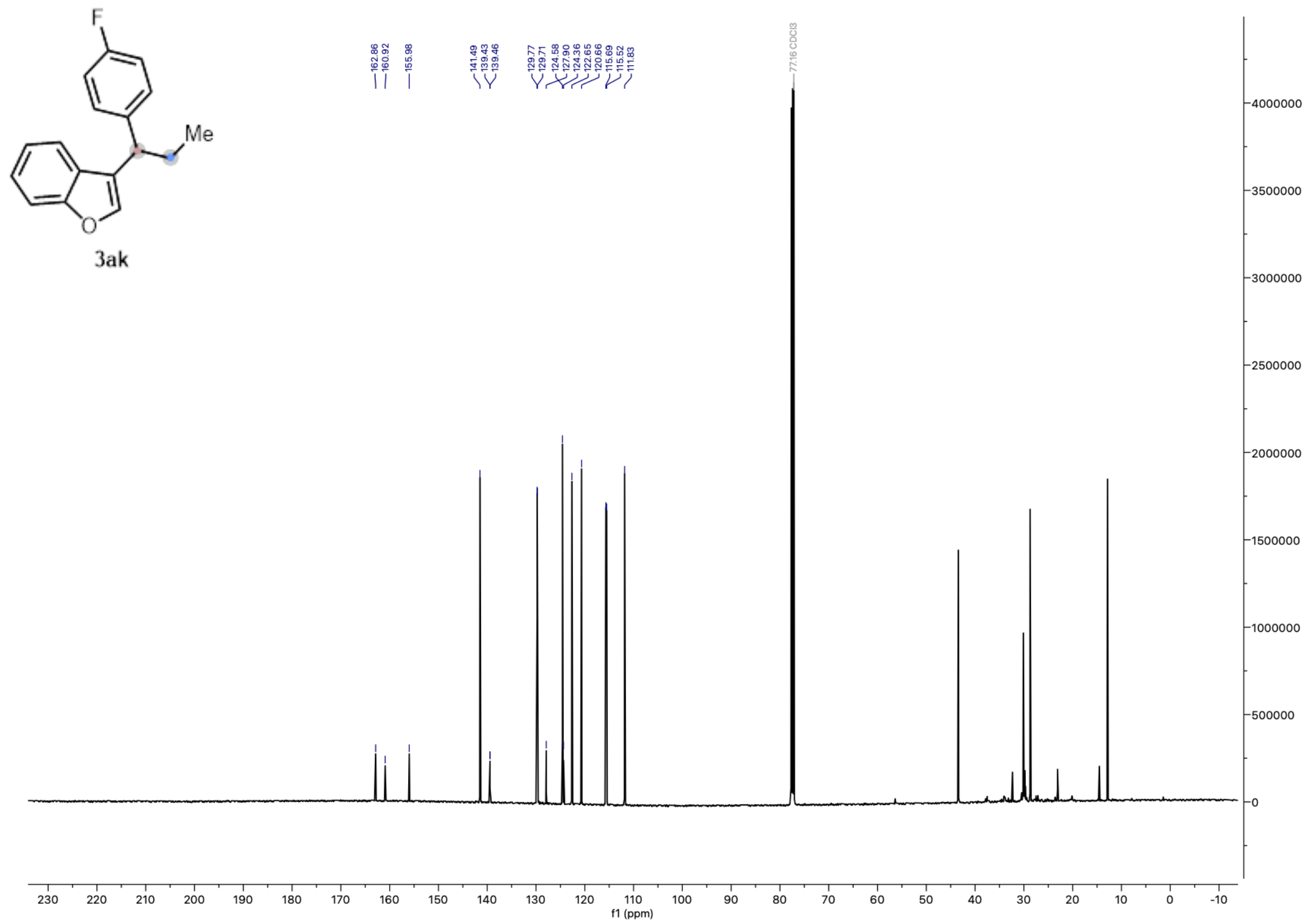

3ak

^{19}F NMR (282 MHz, $CDCl_3$):

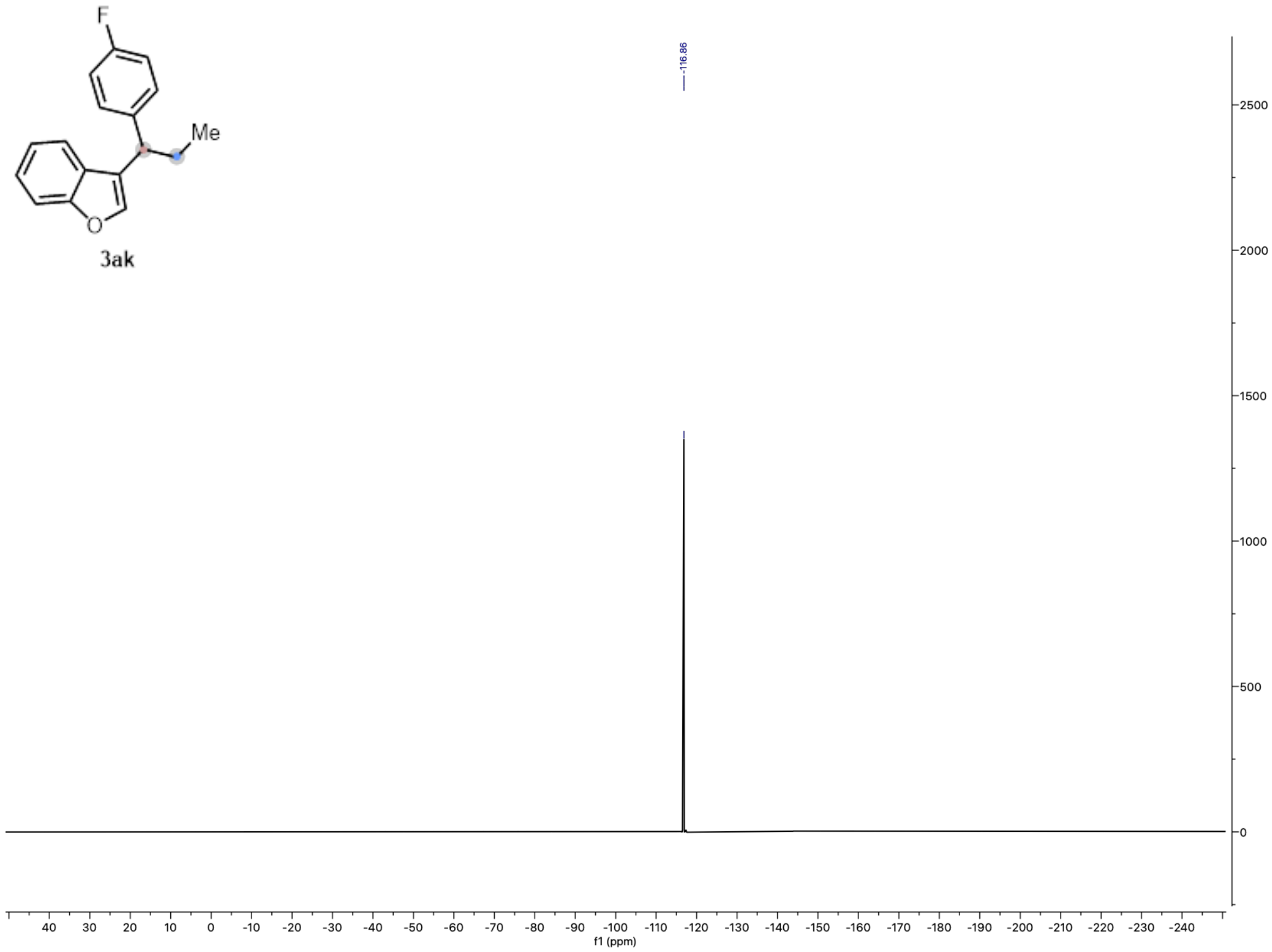

3-(1-(4-fluorophenyl)propyl)-1-methyl-1*H*-indole **3al**

^{1}H NMR (500 MHz, $CDCl_3$):

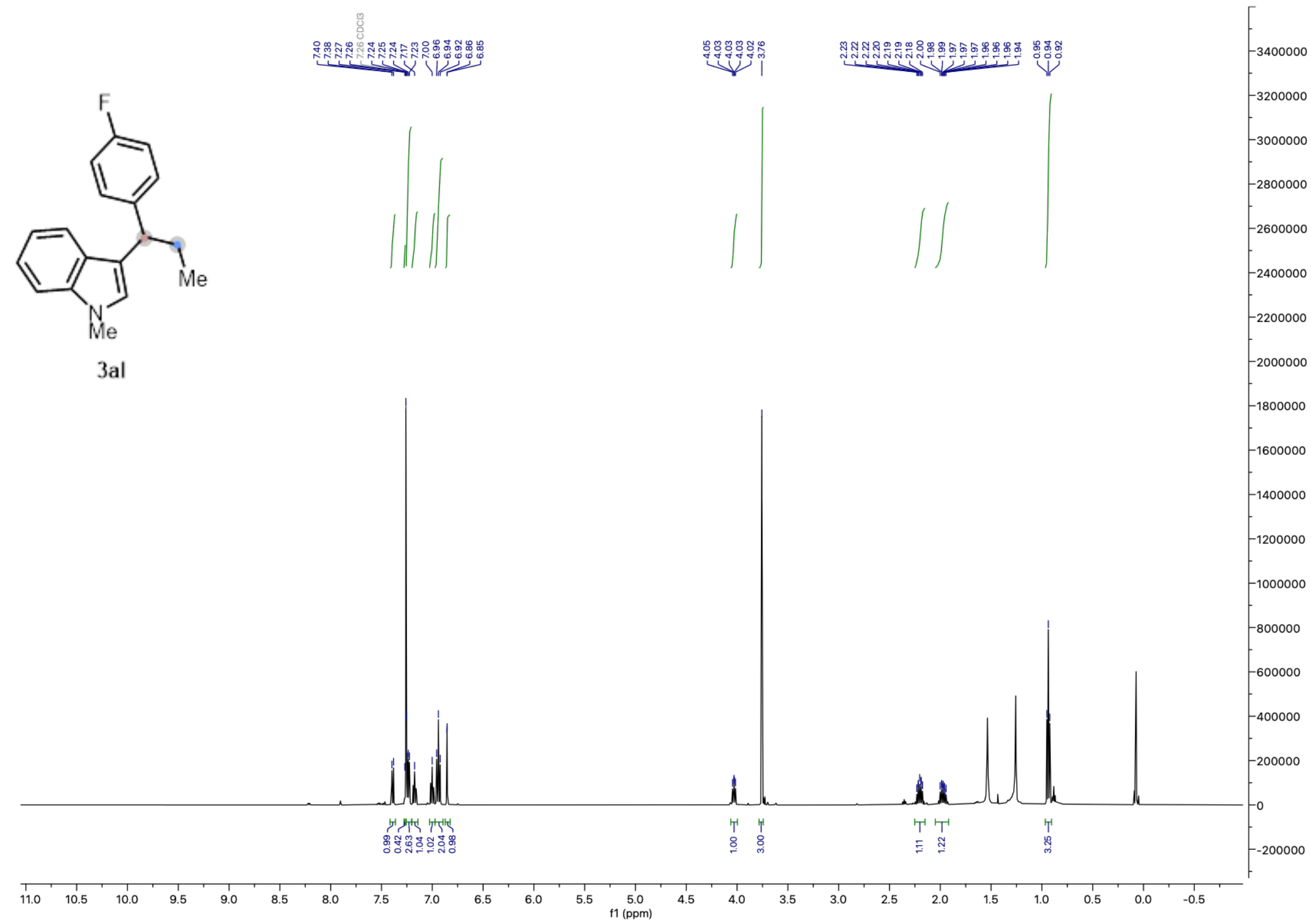

^{13}C NMR (126 MHz, $CDCl_3$):

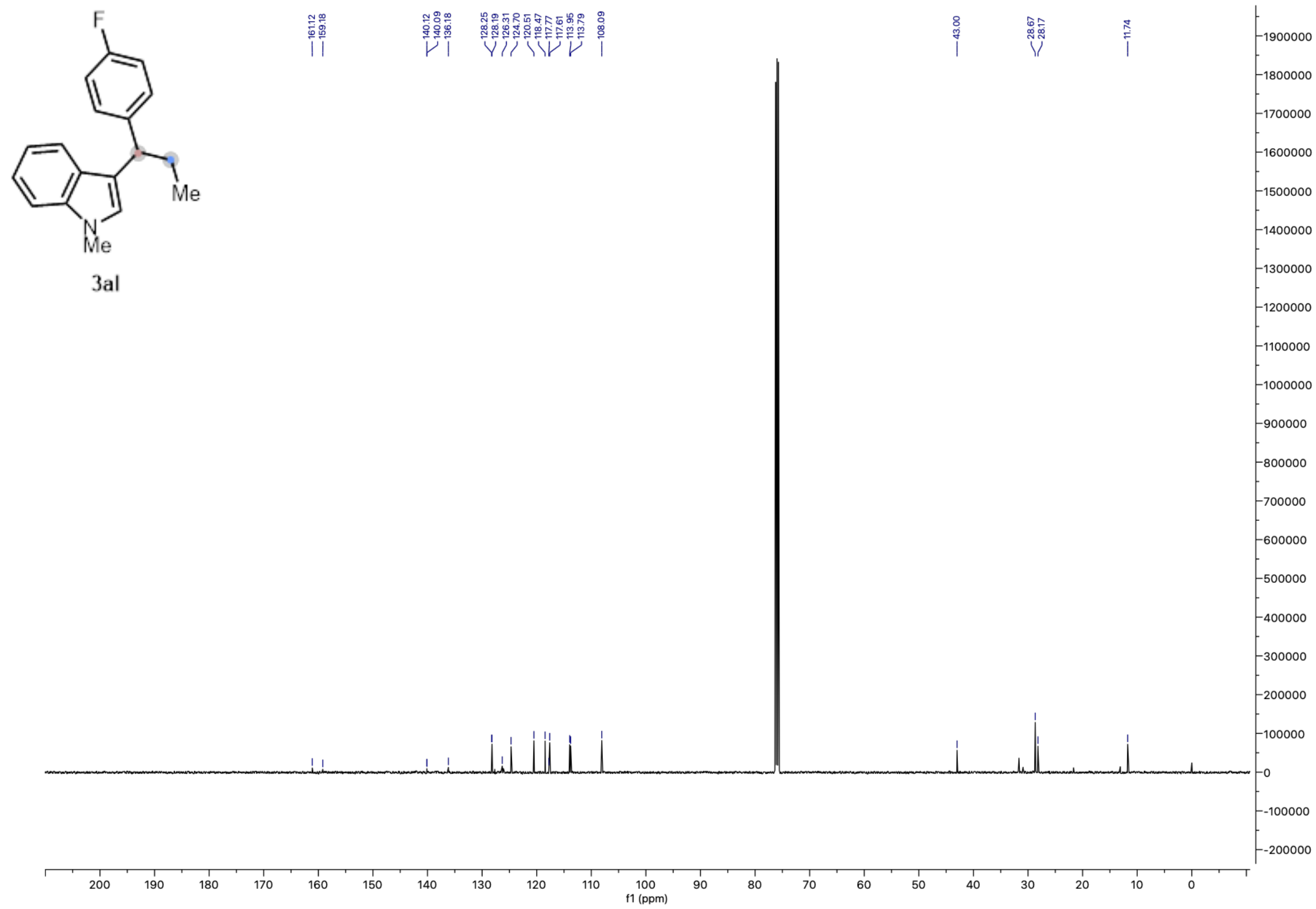

^{19}F NMR (282 MHz, $CDCl_3$):

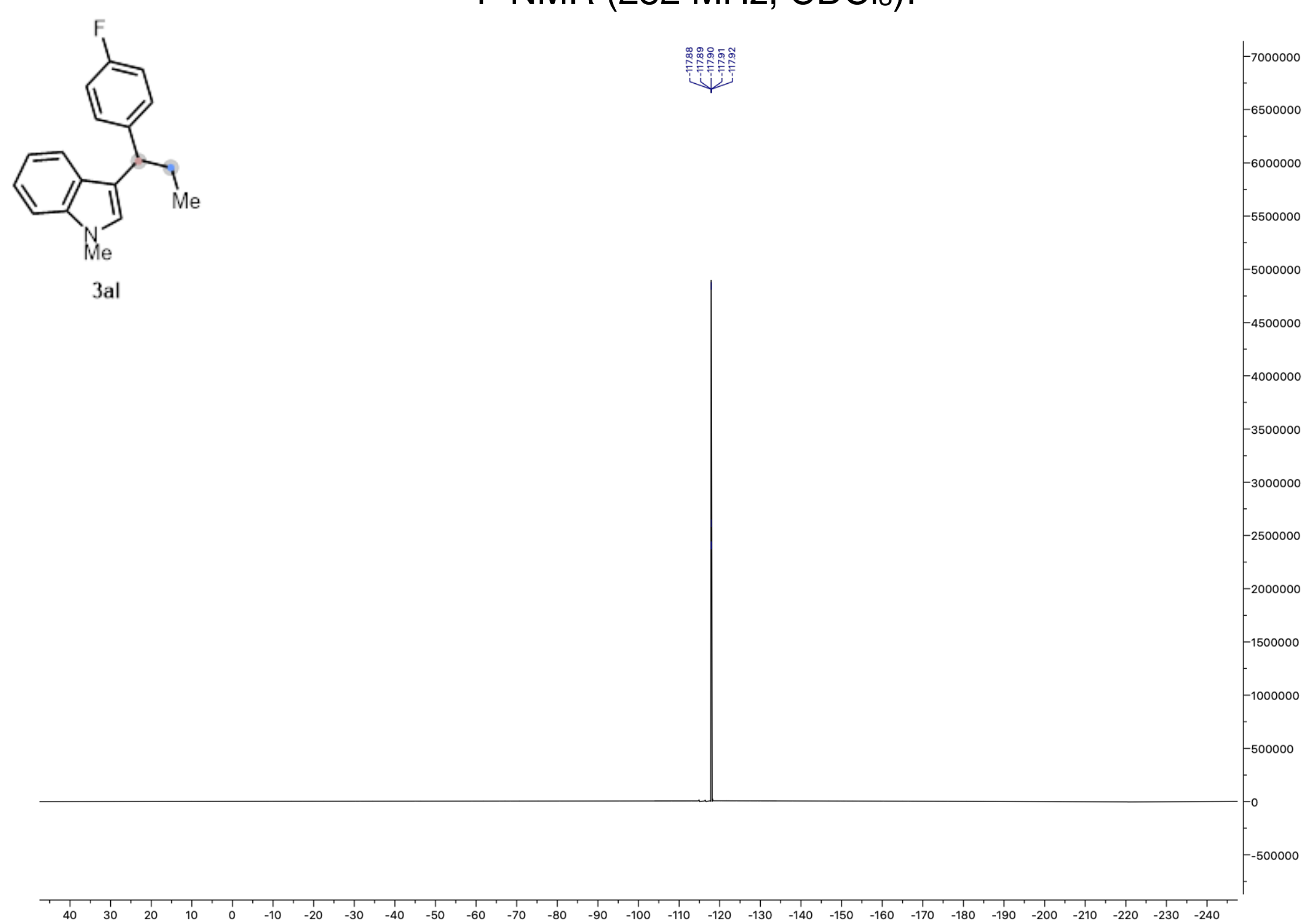

4-(1-(4-fluorophenyl)ethyl)-1-phenyl-1*H*-pyrazole **3am**:

^{1}H NMR (500 MHz, $CDCl_3$):

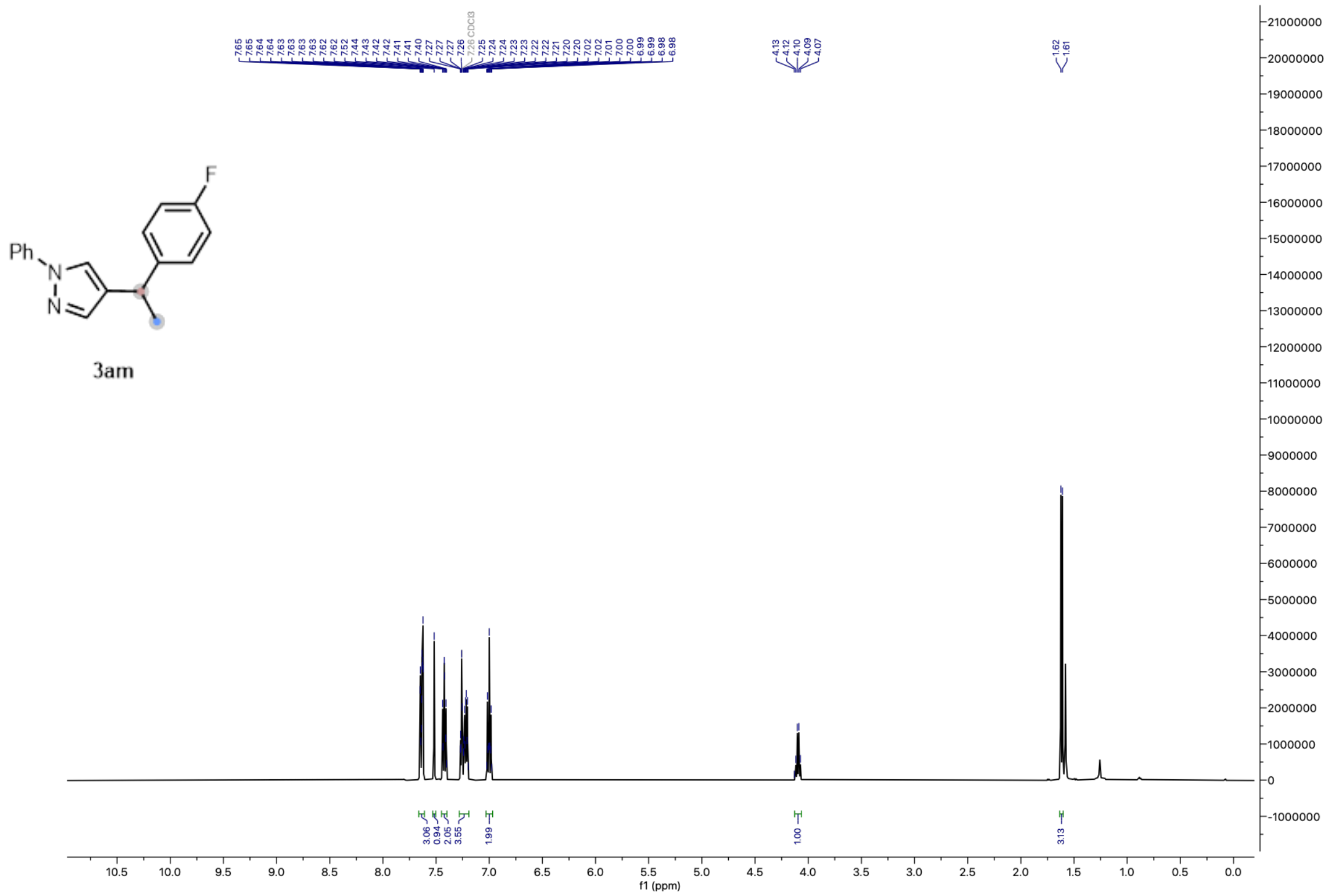

^{13}C NMR (126 MHz, $CDCl_3$):

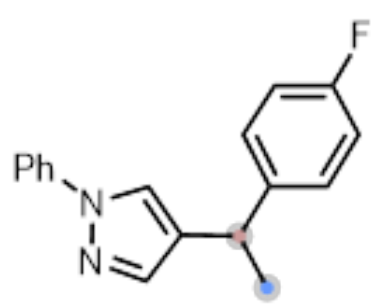

3am

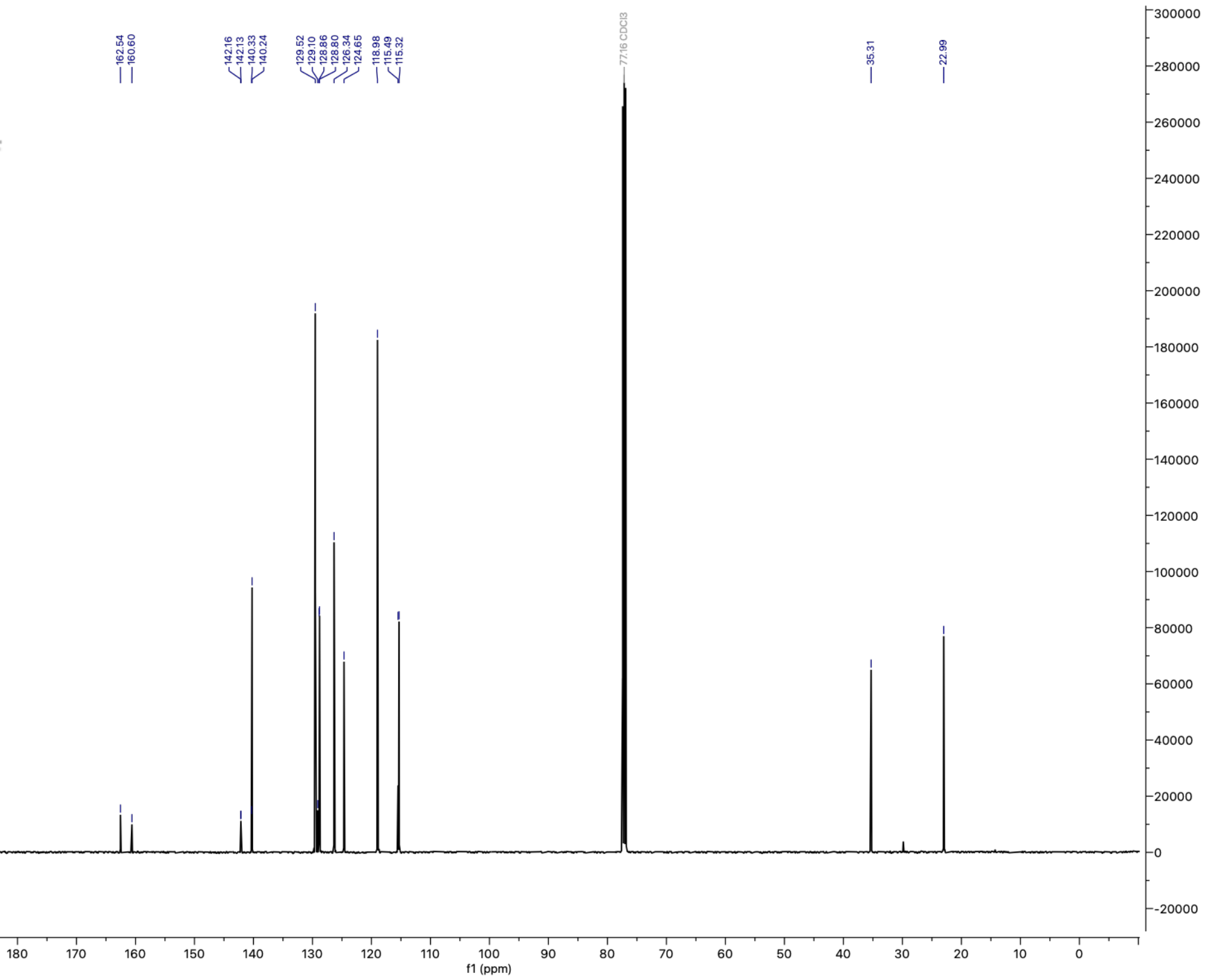

¹⁹F NMR (282 MHz, $CDCl_3$):

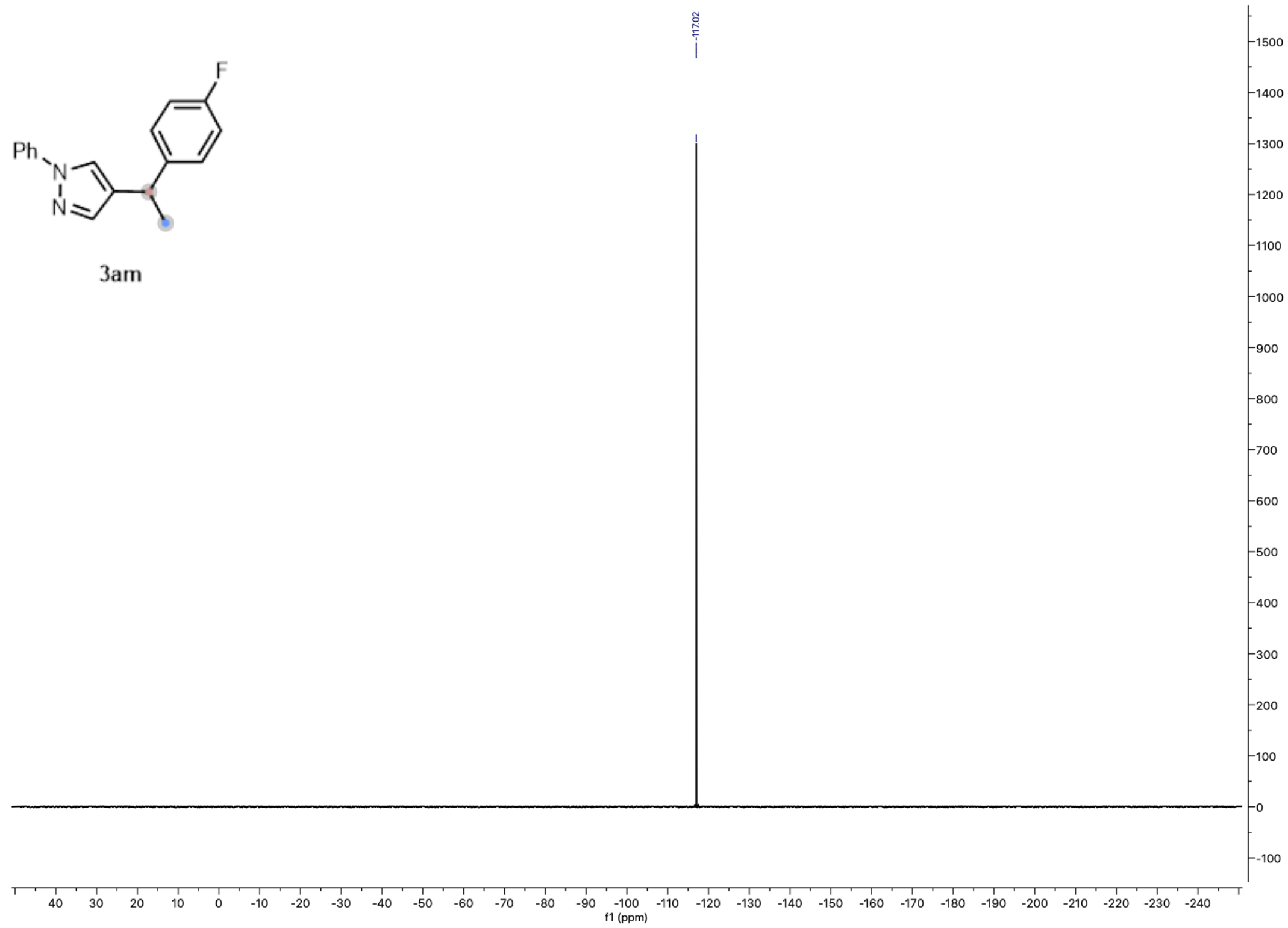

4-(5-(4-(1-Phenylbutyl)3-(trifluoromethyl)-*1H*-pyrazol-1-yl)benzenesulfonamide **3ao**:

^{1}H NMR (500 MHz, CDCl$_3$):

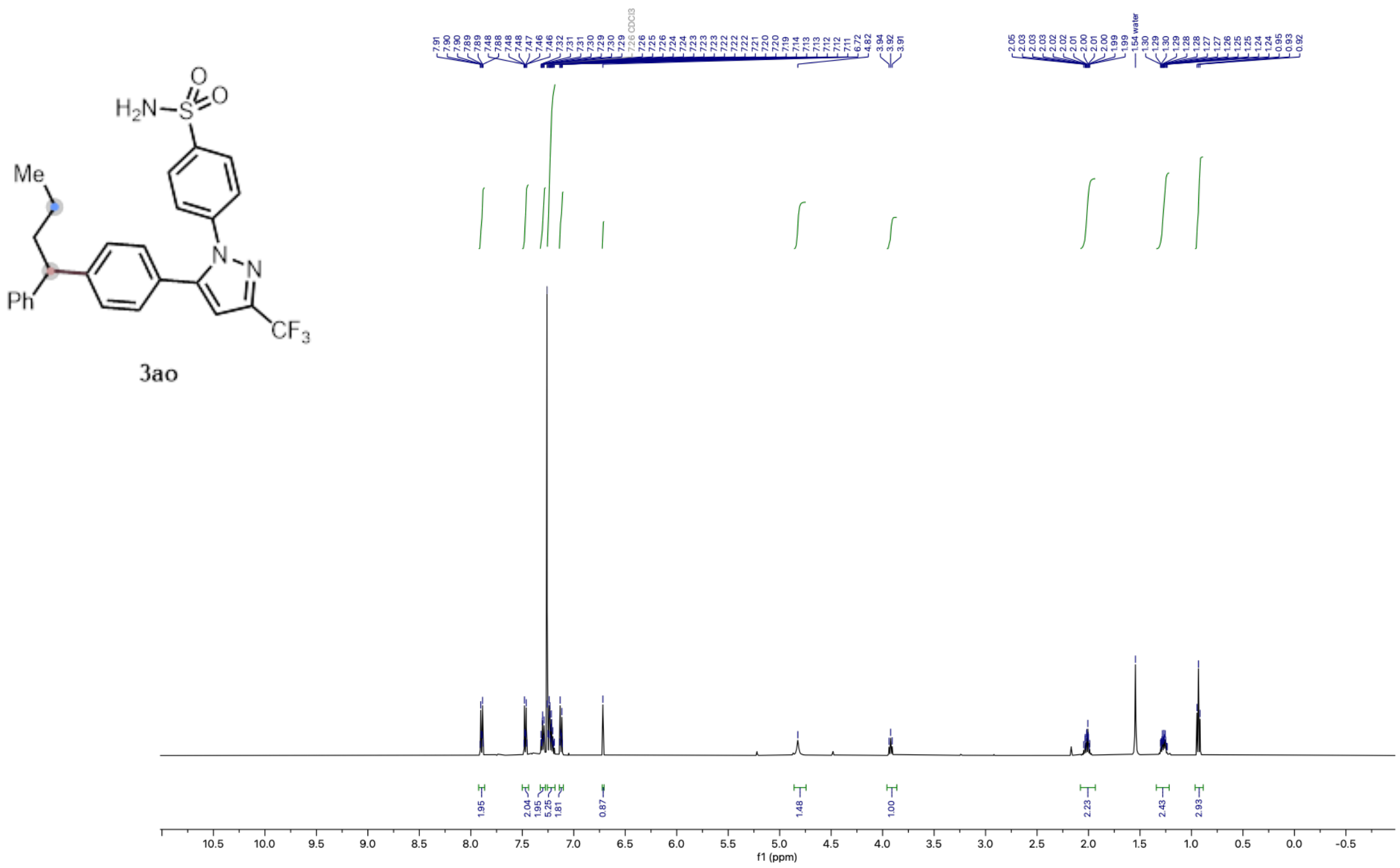

^{13}C NMR (126 MHz, CDCl$_3$):

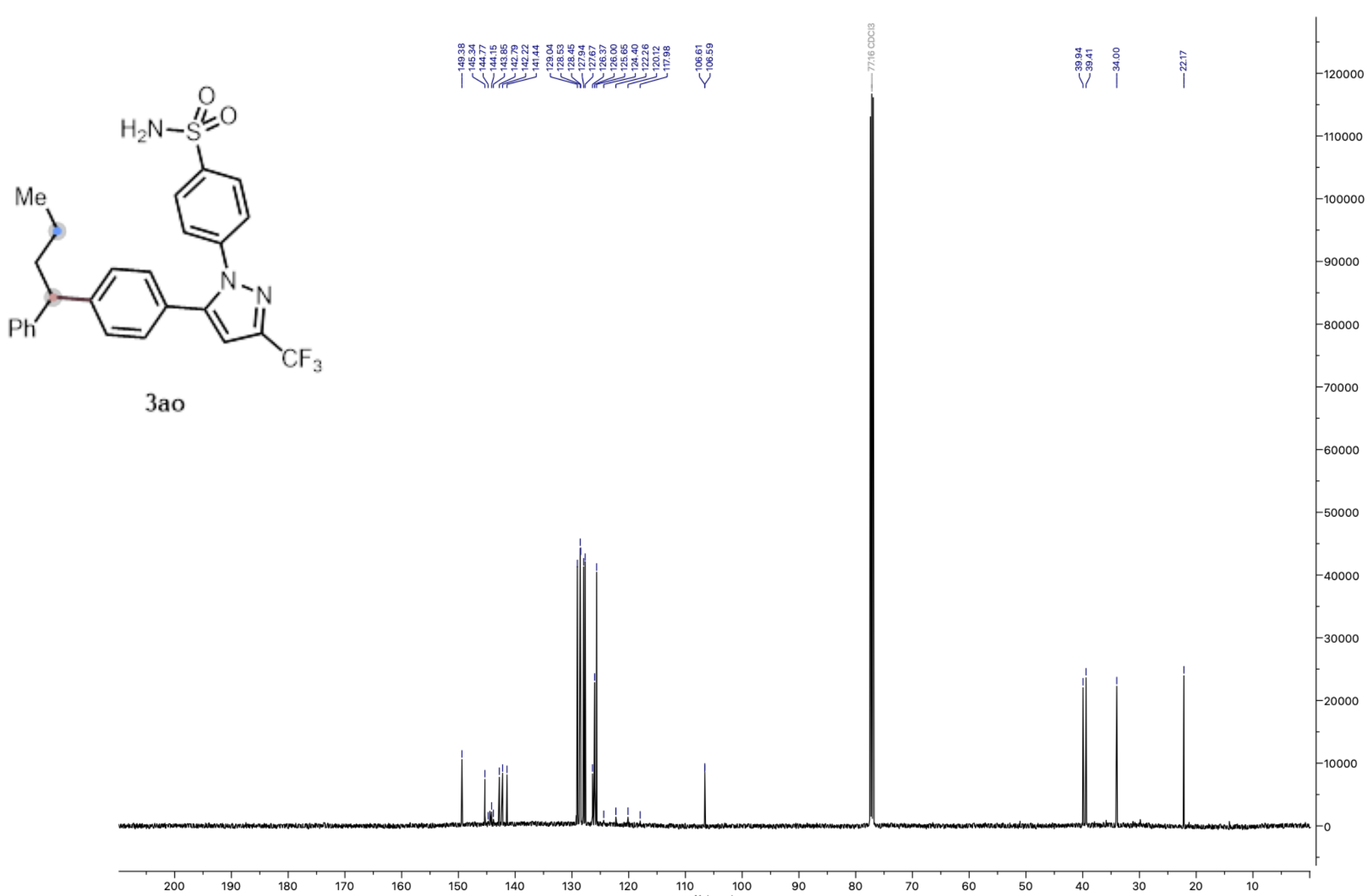

^{19}F NMR (282 MHz, $CDCl_3$):

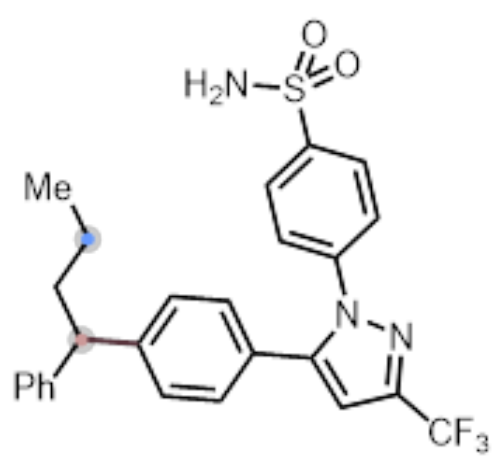

3ao

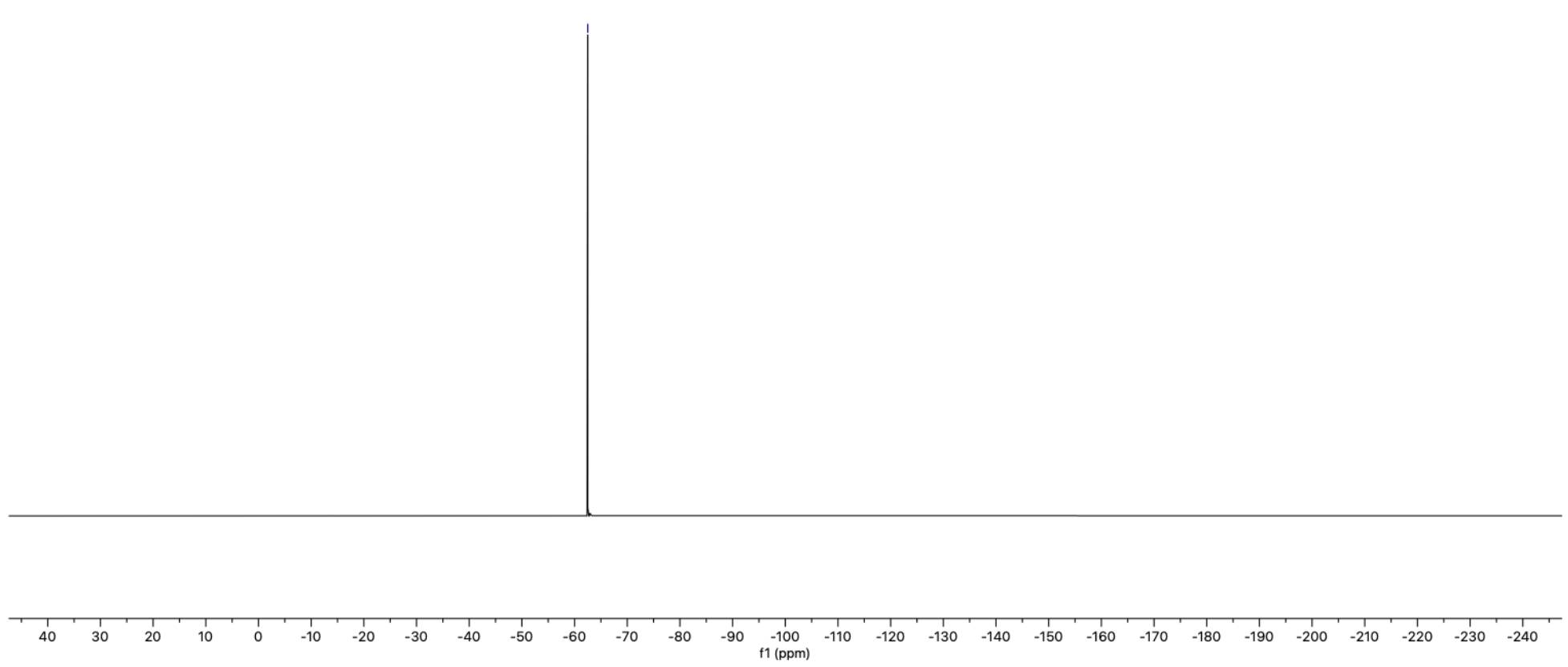

5-Benzoyl-*N*-(3-((*R*)-1-phenylbutyl)phenyl)-2,3-dihydro-1*H*-pyrrolizine-1-carboxamide **3ap**:

^{1}H NMR (500 MHz, $CDCl_3$):

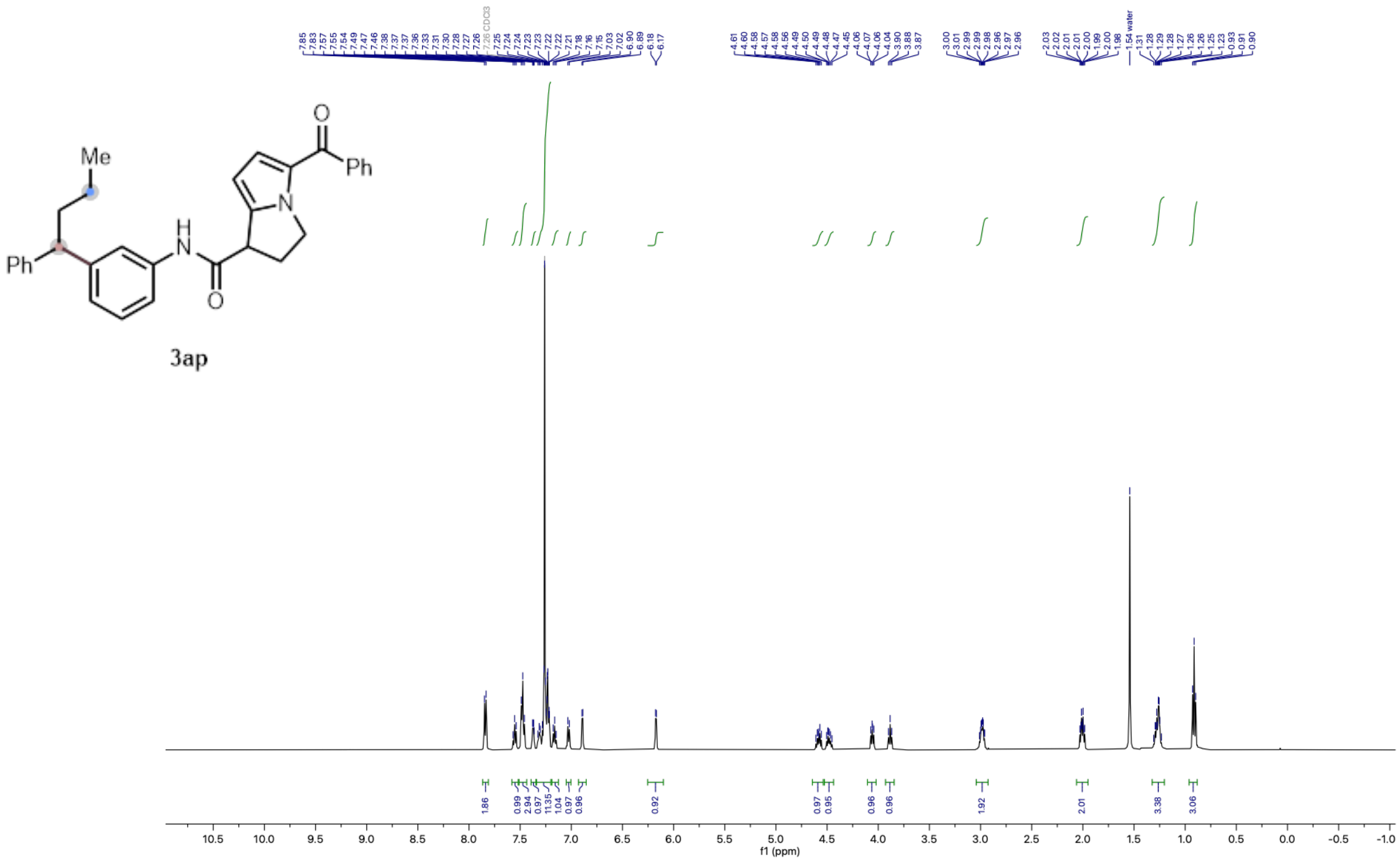

3ap

^{13}C NMR (126 MHz, $CDCl_3$):

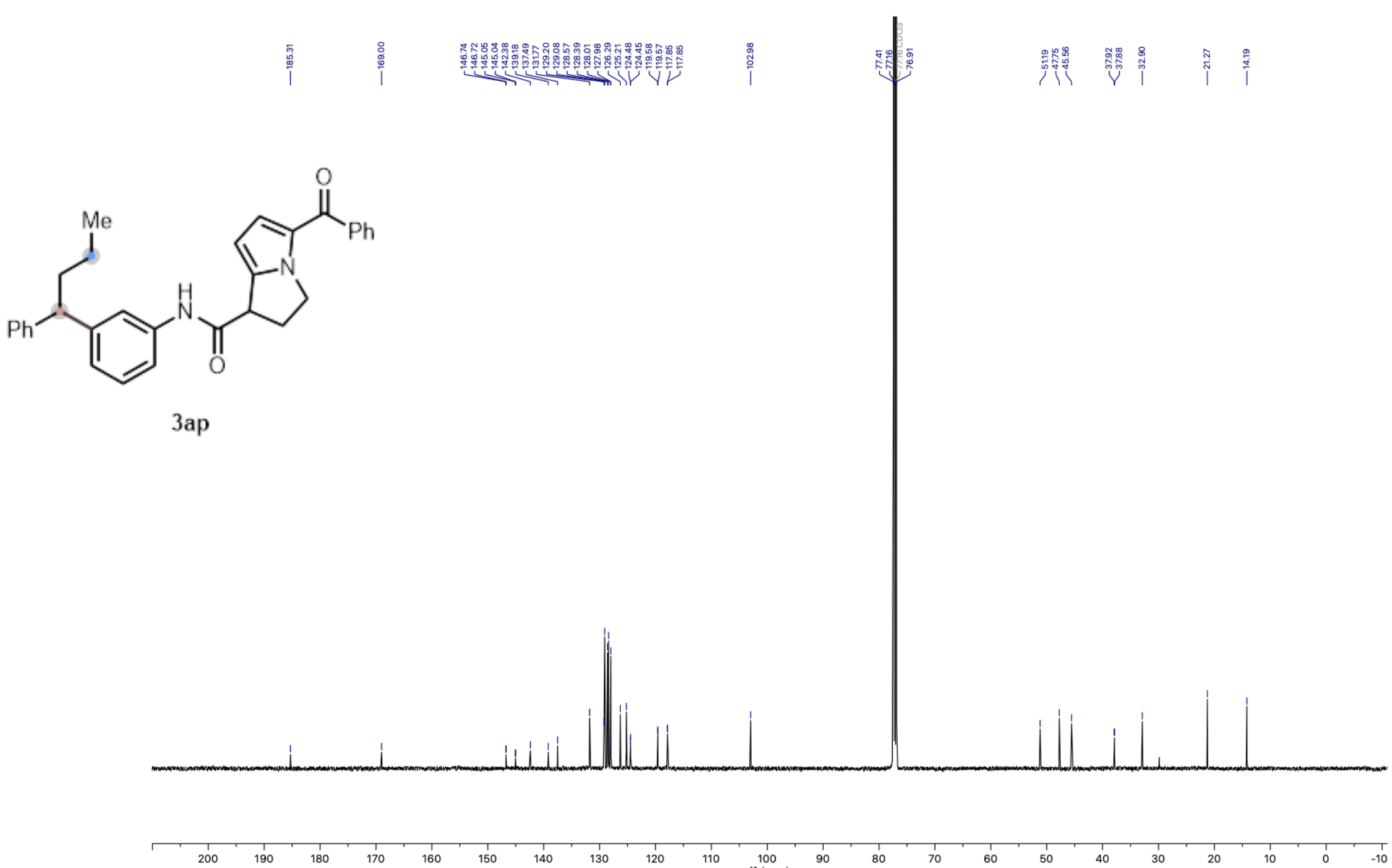

3ap

(4a*R*,4a¹*R*,5a*S*,8a*R*,8a¹*S*,15a*S*)-10-((*R*)-1-Phenylbutyl)-2,4a,4a¹,5,5a,7,8,8a¹,15,15a-decahydro-14*H*-4,6-methanoindolo[3,2,1-*ij*]oxepino[2,3,4-*de*]pyrrolo[2,3-*h*]quinolin-14-one **3aq**:

^{1}H NMR (500 MHz, $CDCl_3$):

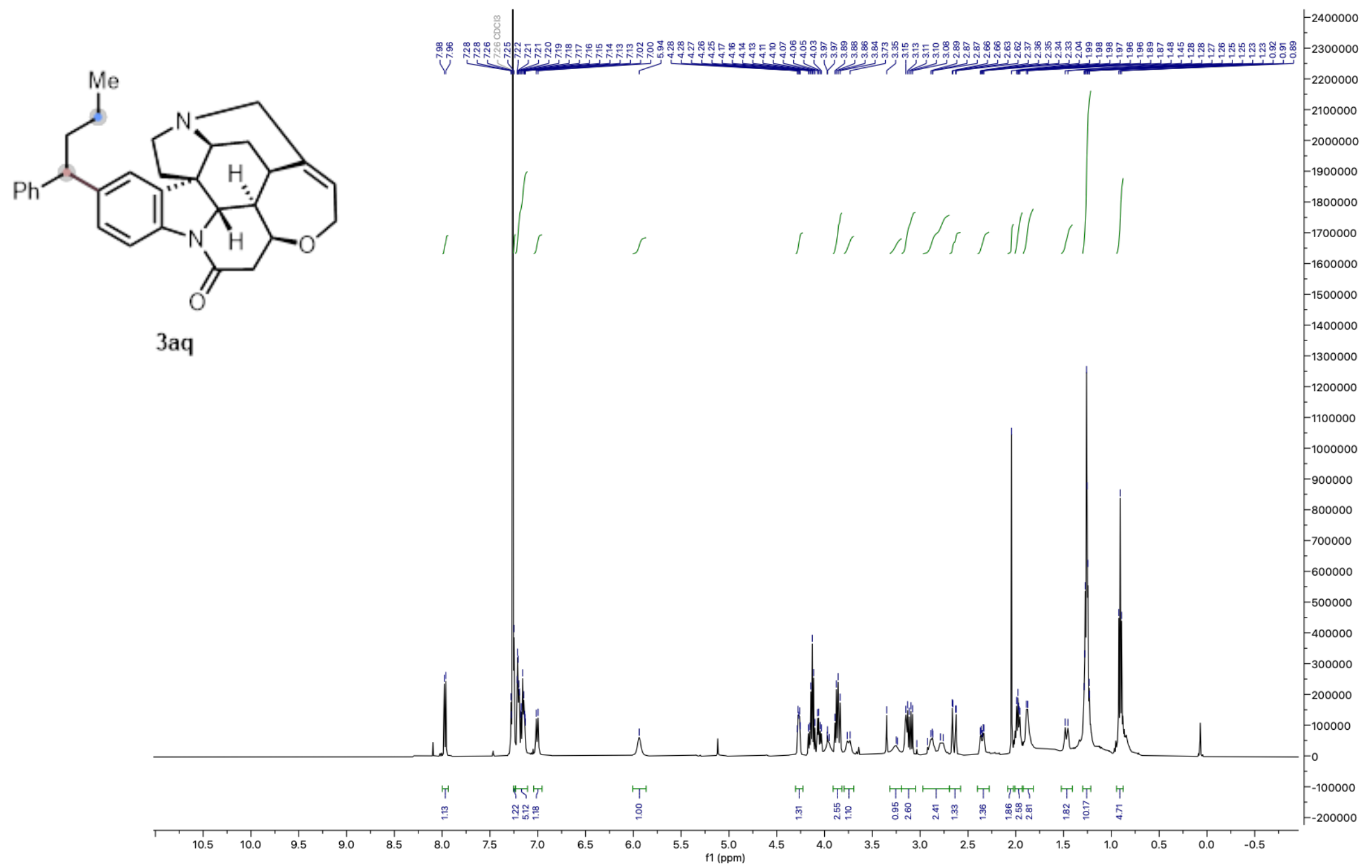

3aq

^{13}C NMR (126 MHz, $CDCl_3$):

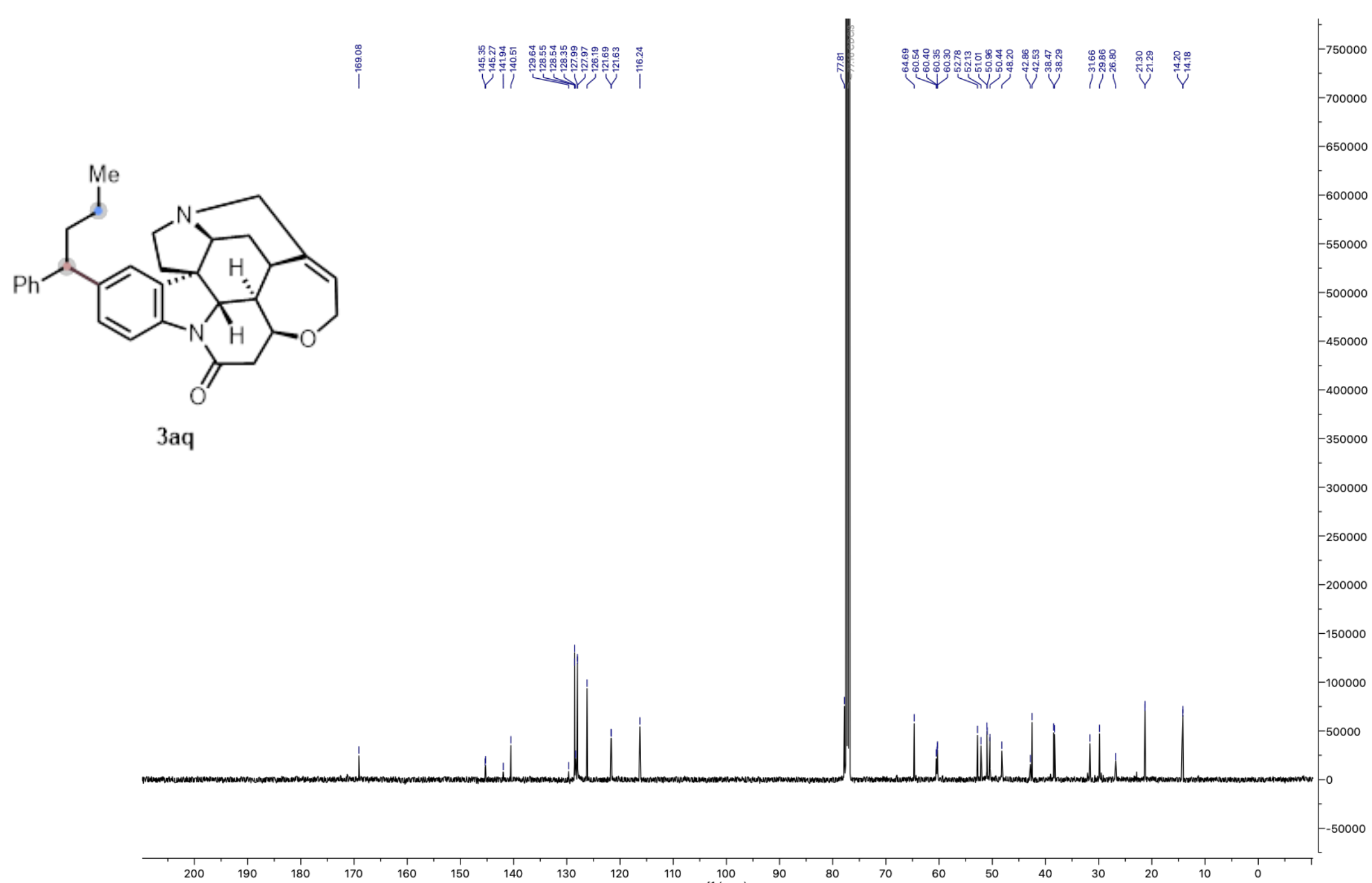

3aq

2-(2,6-Dioxopiperidin-3-yl)-4-((*R*)-1-phenylbutyl)isoindoline-1,3-dione **3ar**:

^{1}H NMR (500 MHz, $CDCl_3$):

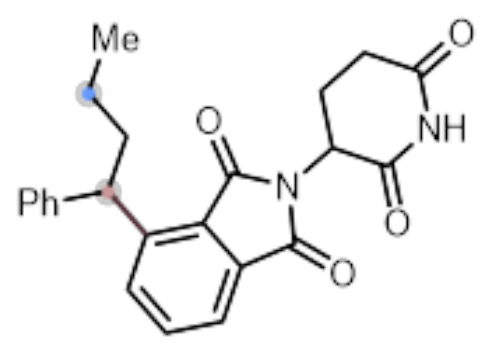

3ar

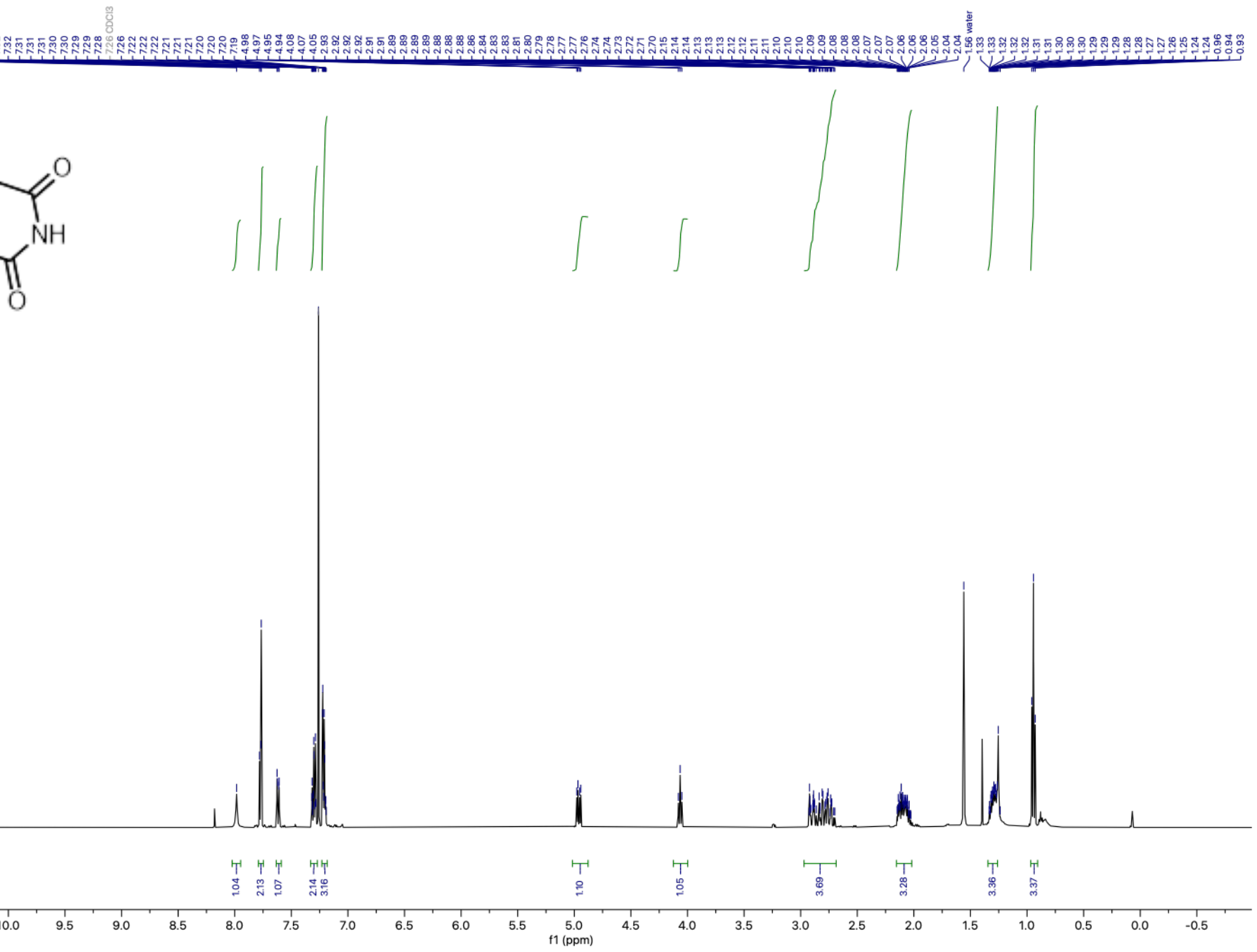

^{13}C NMR (126 MHz, $CDCl_3$):

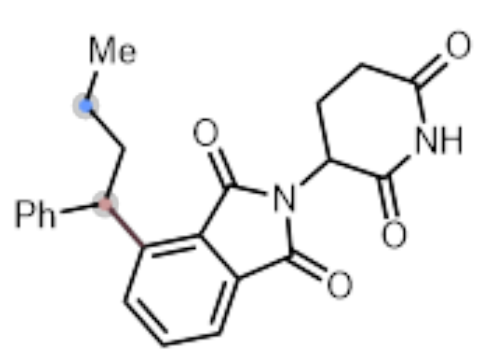

3ar

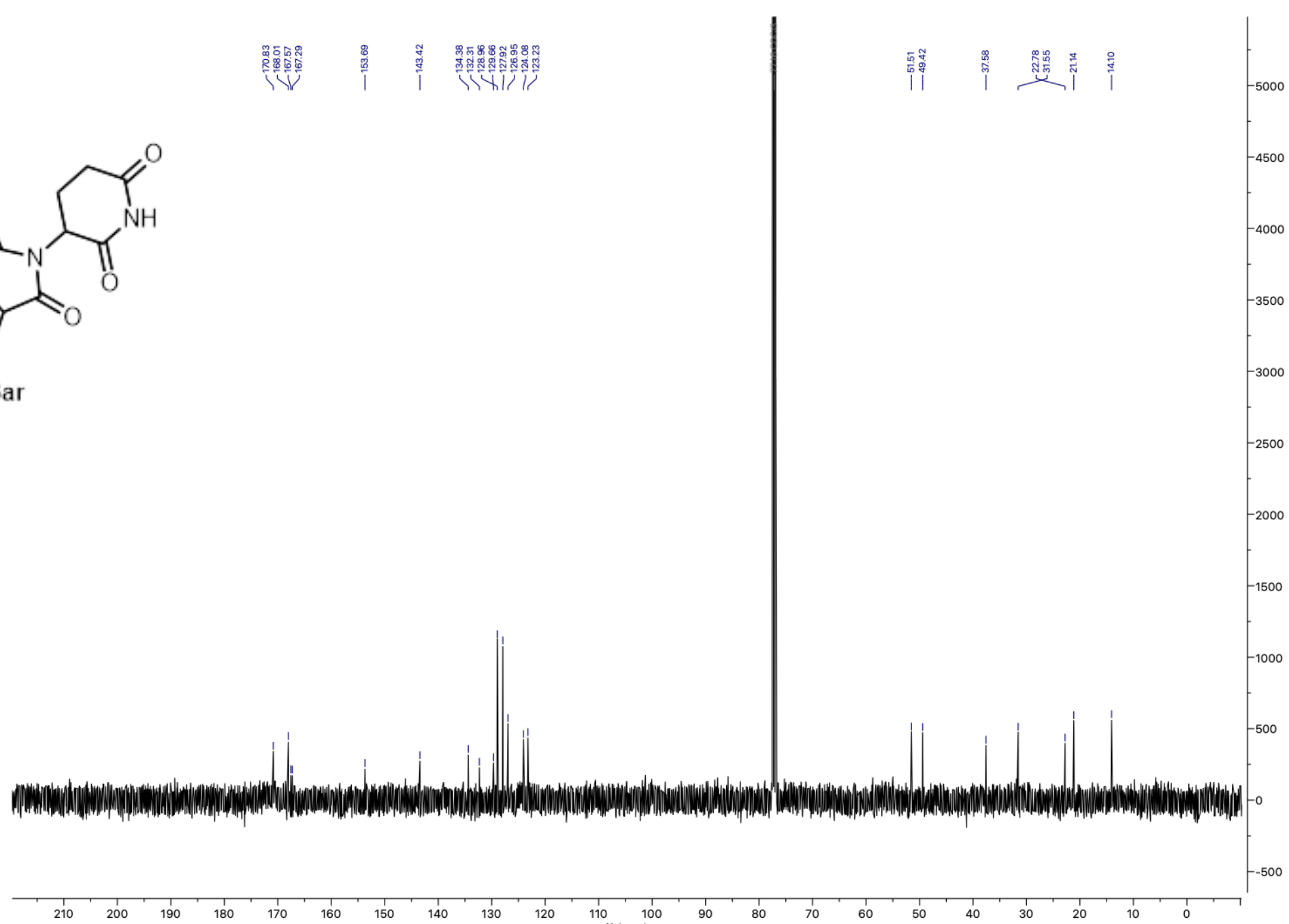

(*Z*)-3-(2-(1-(4-fluorophenyl)propyl)dibenzo[*b*,*e*]oxepin-11(6*H*)-ylidene)-*N*,*N*-dimethylpropan-1-amine **3as**:

^{1}H NMR (500 MHz, $CDCl_3$):

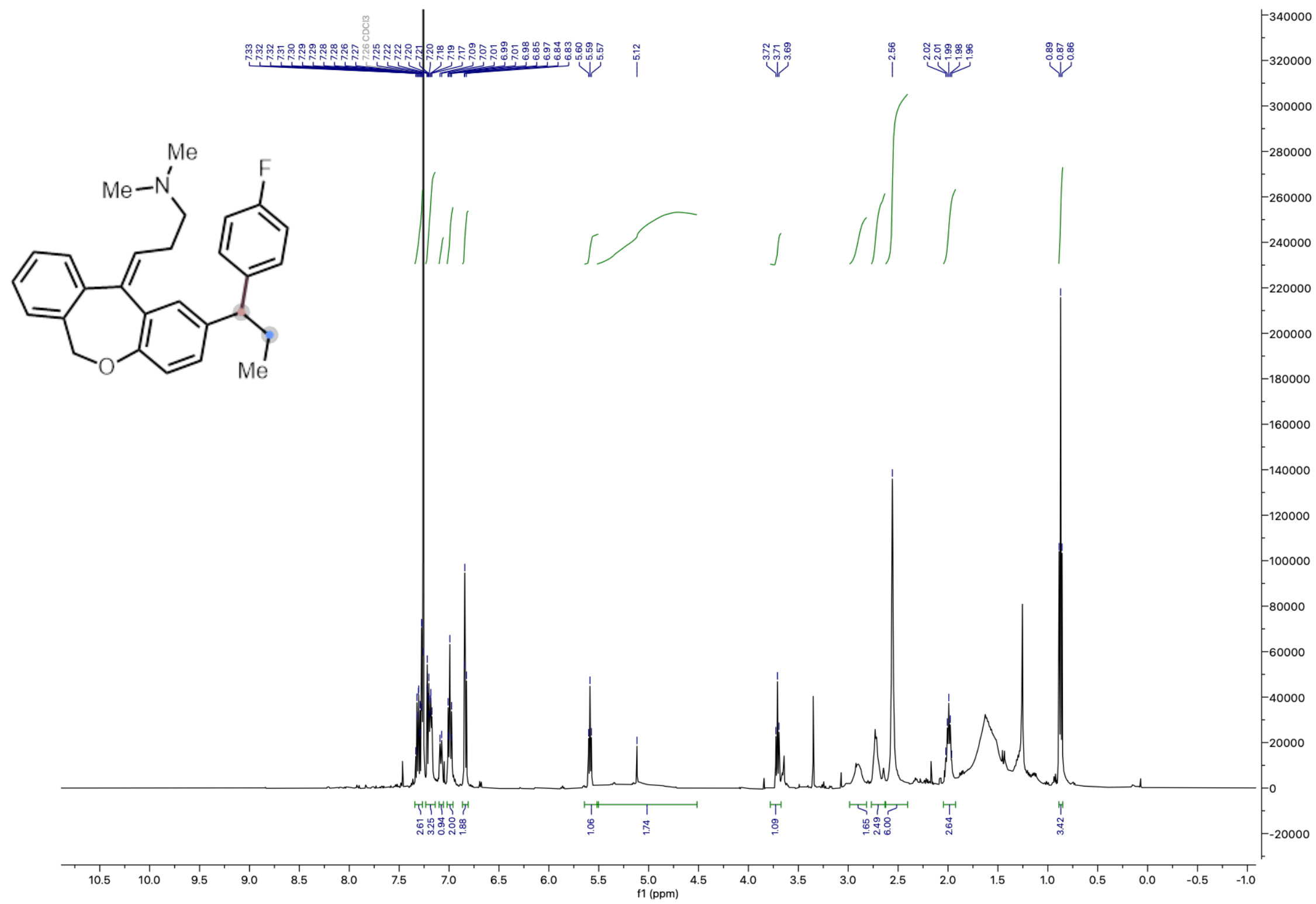

^{13}C NMR (126 MHz, $CDCl_3$):

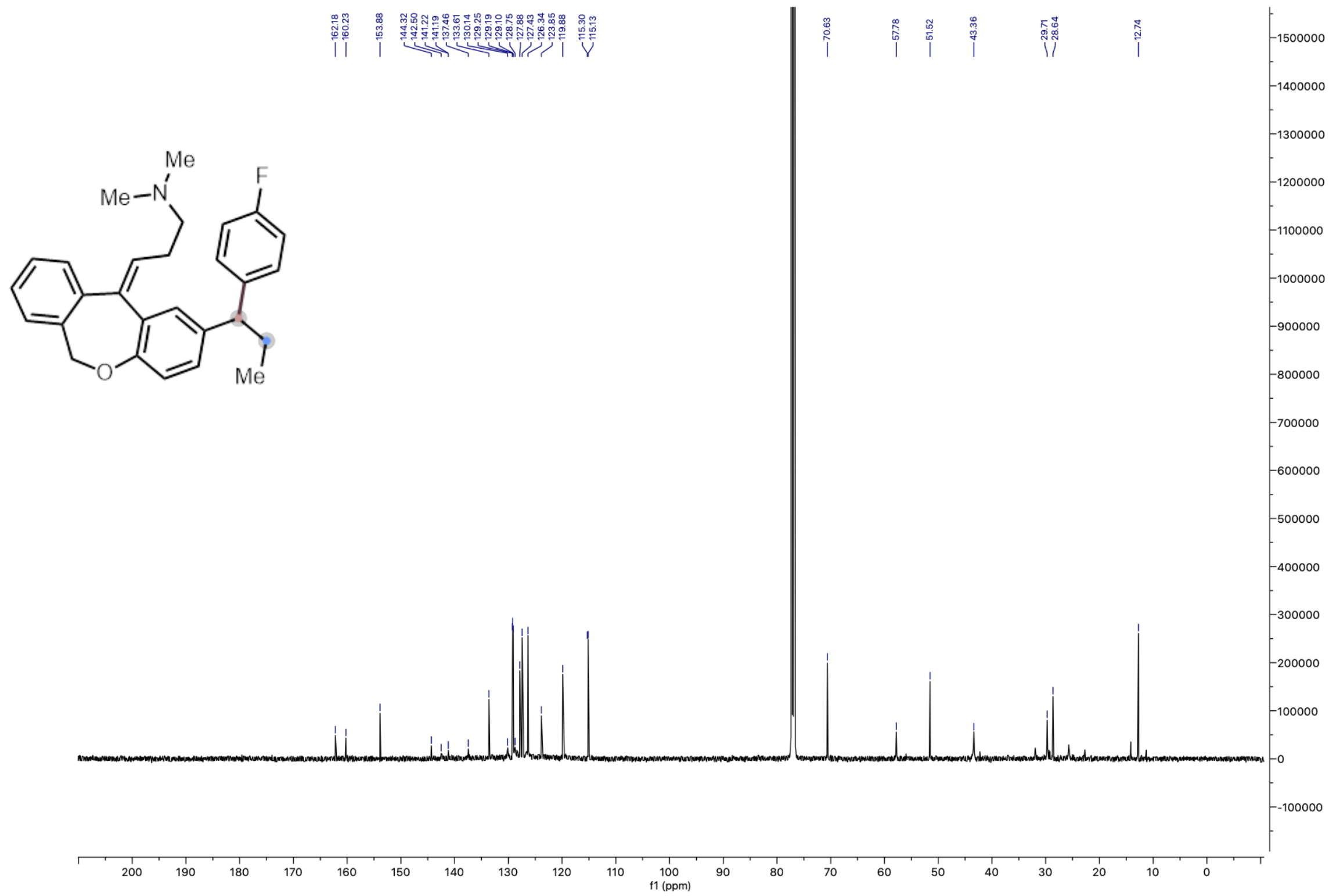

^{19}F NMR (282 MHz, $CDCl_3$):

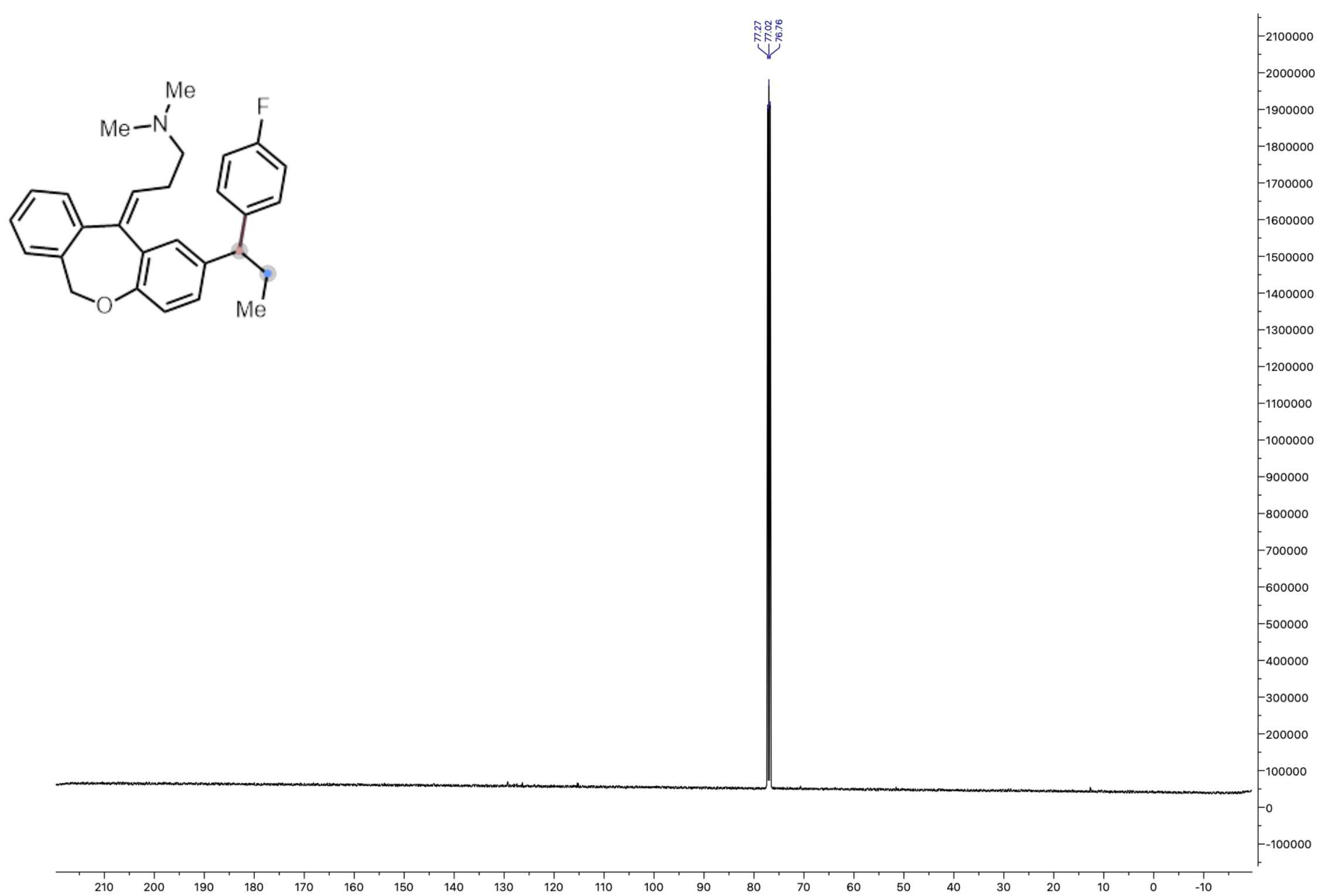

2-(1-(4-fluorophenyl)butyl)-6-methoxynaphthalene: **3at**

^{1}H NMR (500 MHz, $CDCl_3$):

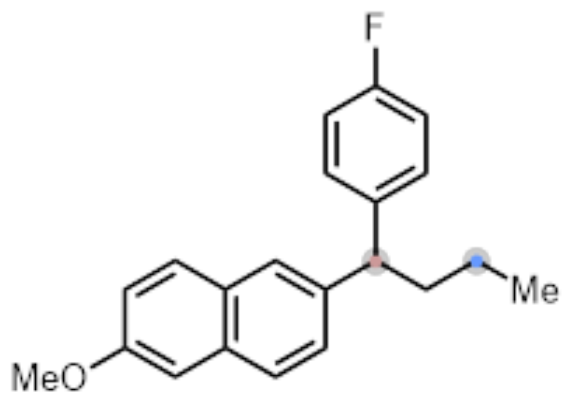

3at

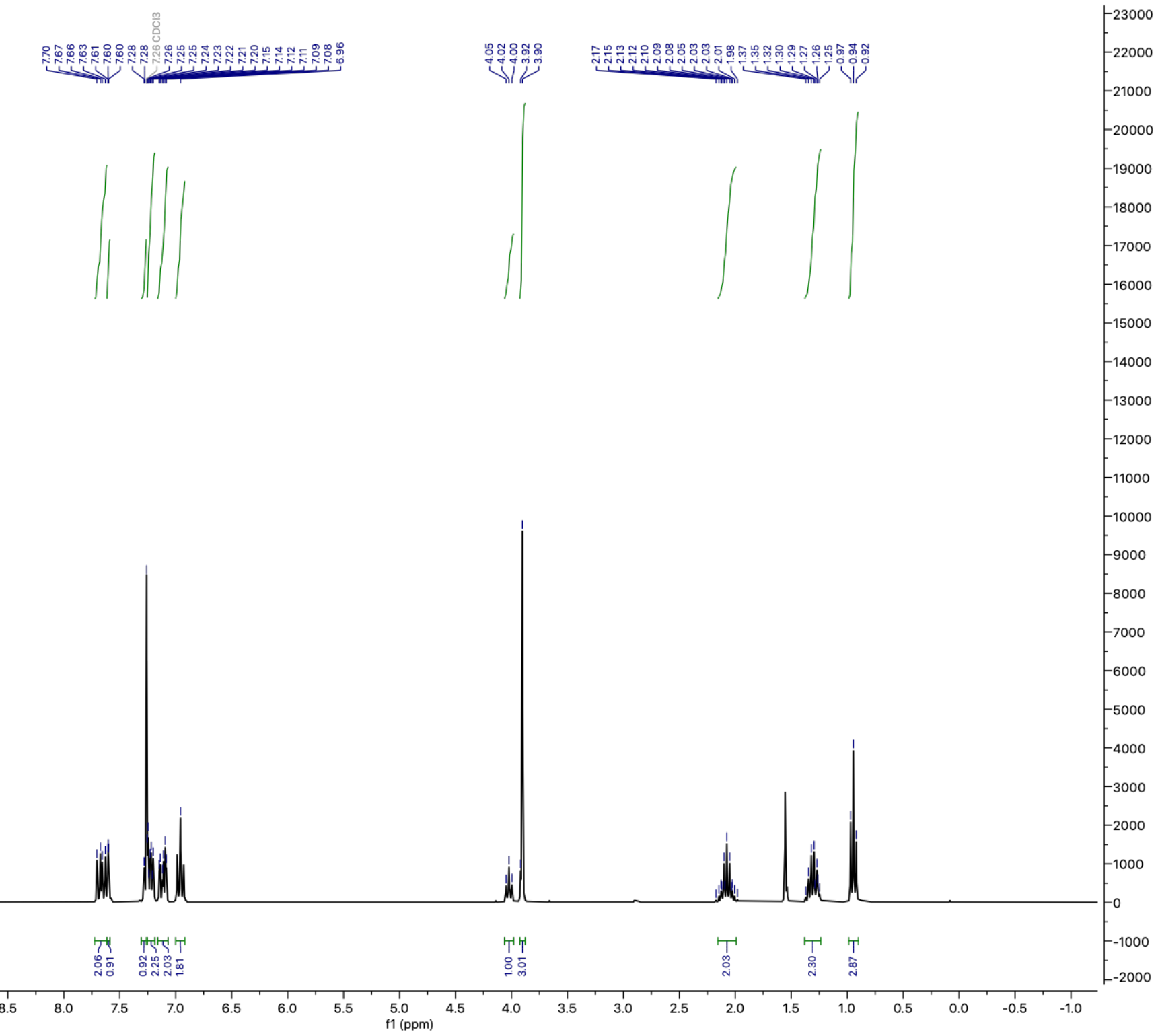

^{13}C NMR (126 MHz, $CDCl_3$):

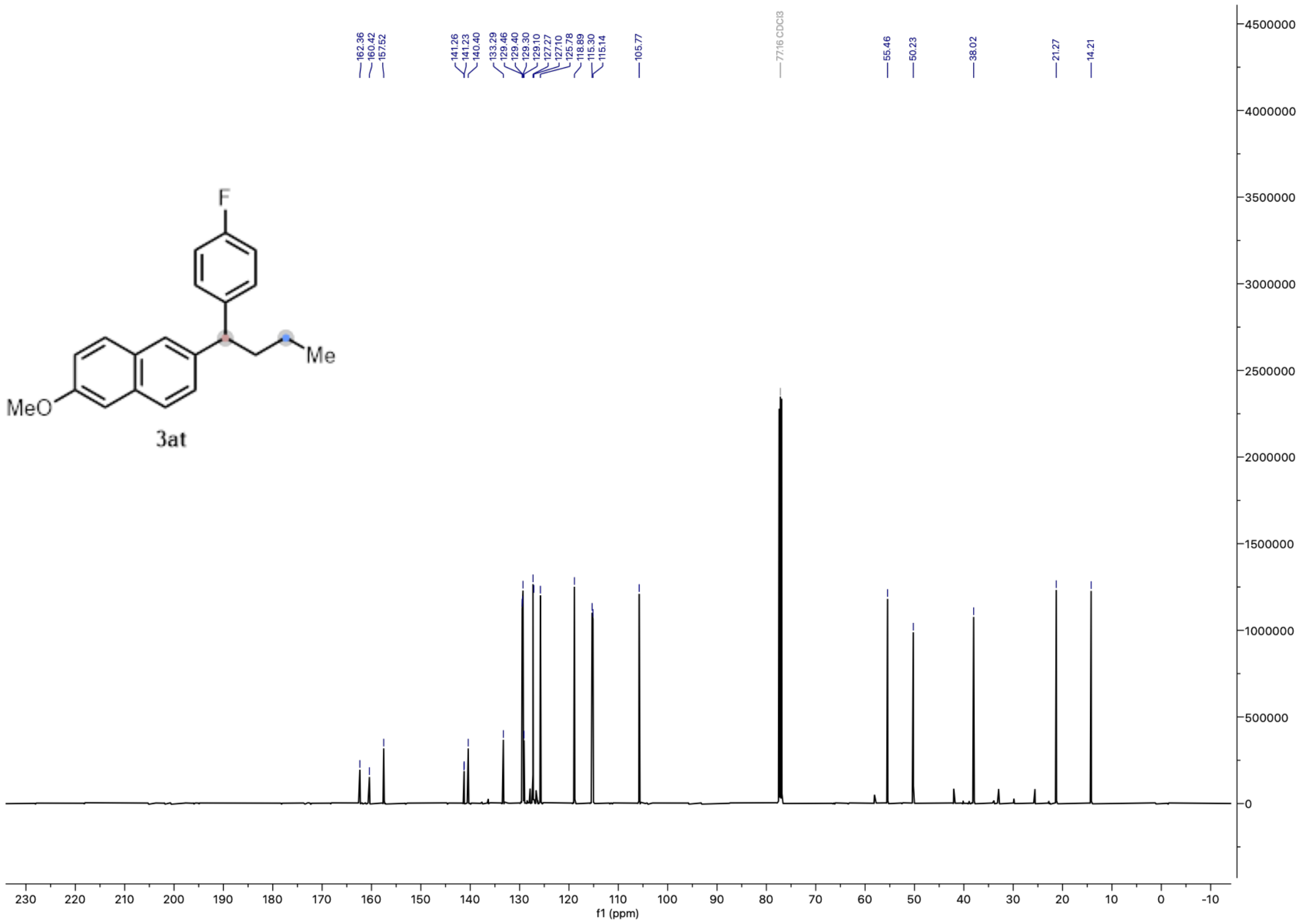

^{19}F NMR (282 MHz, $CDCl_3$):

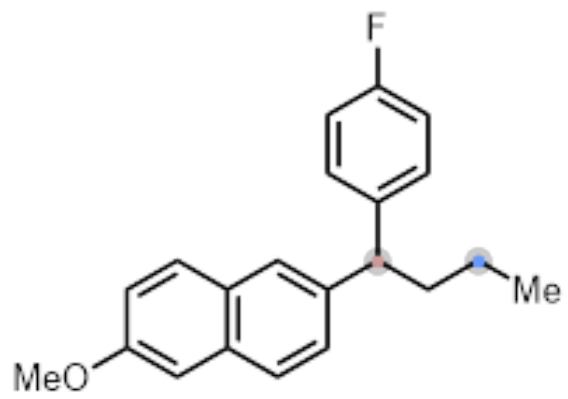

3at

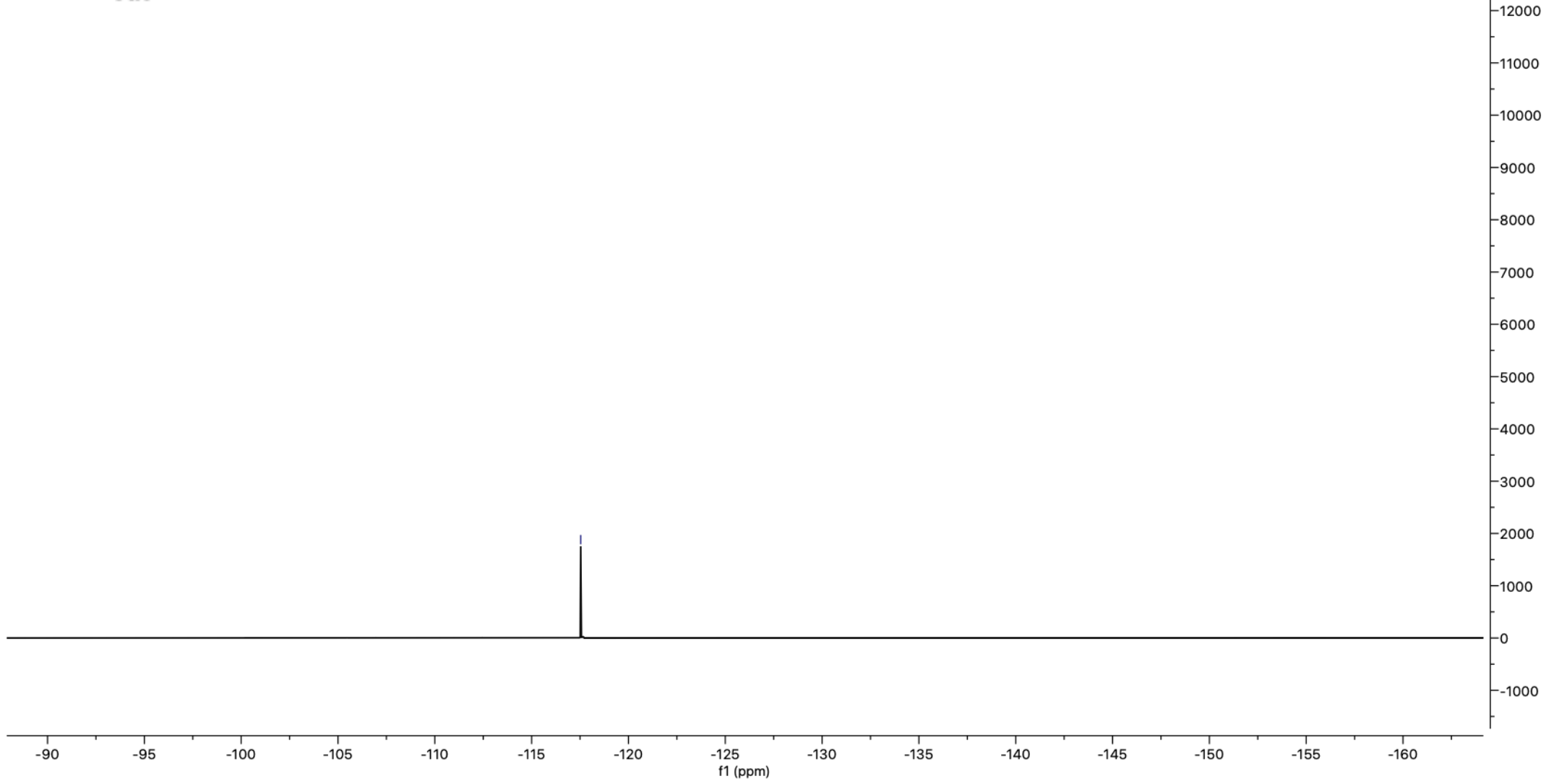

4-(1-(4-fluorophenyl)ethyl)-2-methoxyphenol **3au**:

^{1}H NMR (500 MHz, $CDCl_3$):

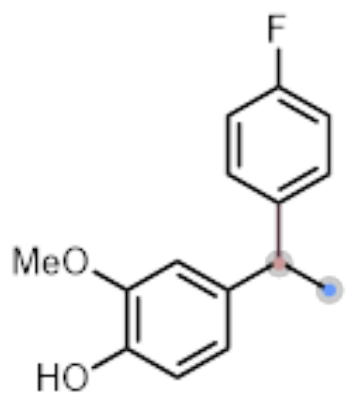

3au

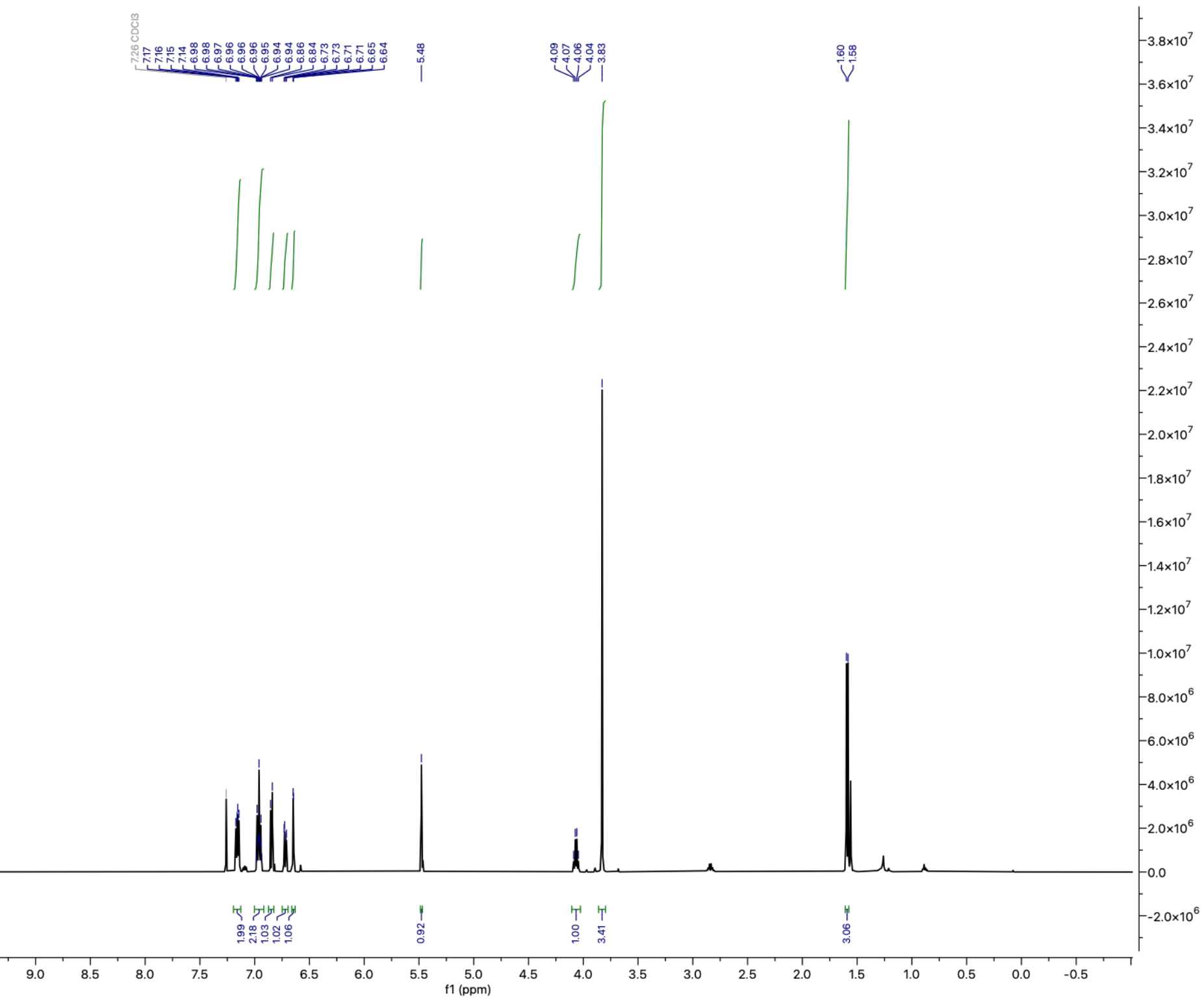

^{13}C NMR (126 MHz, CDCl$_3$):

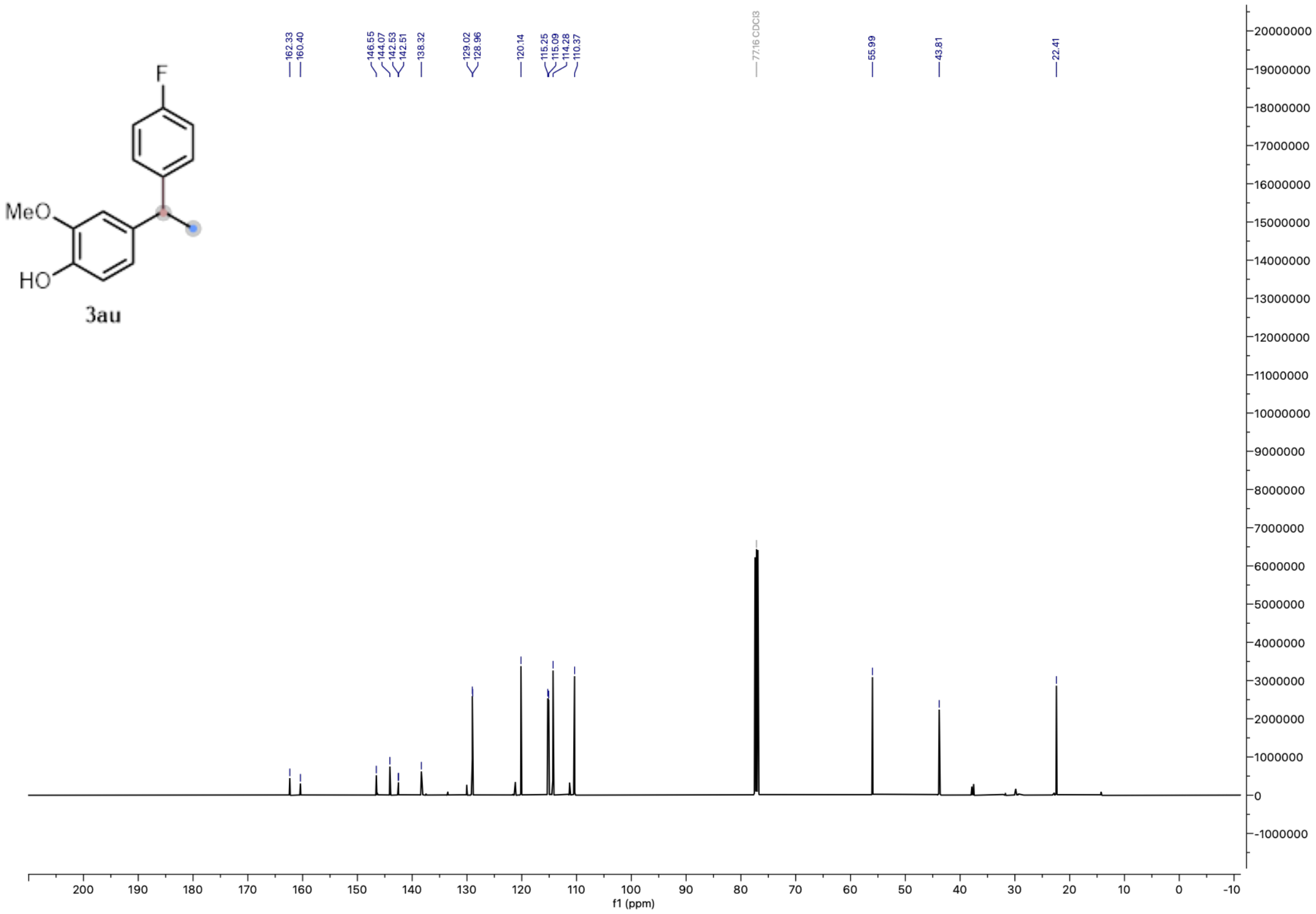

3au

^{19}F NMR (282 MHz, CDCl$_3$):

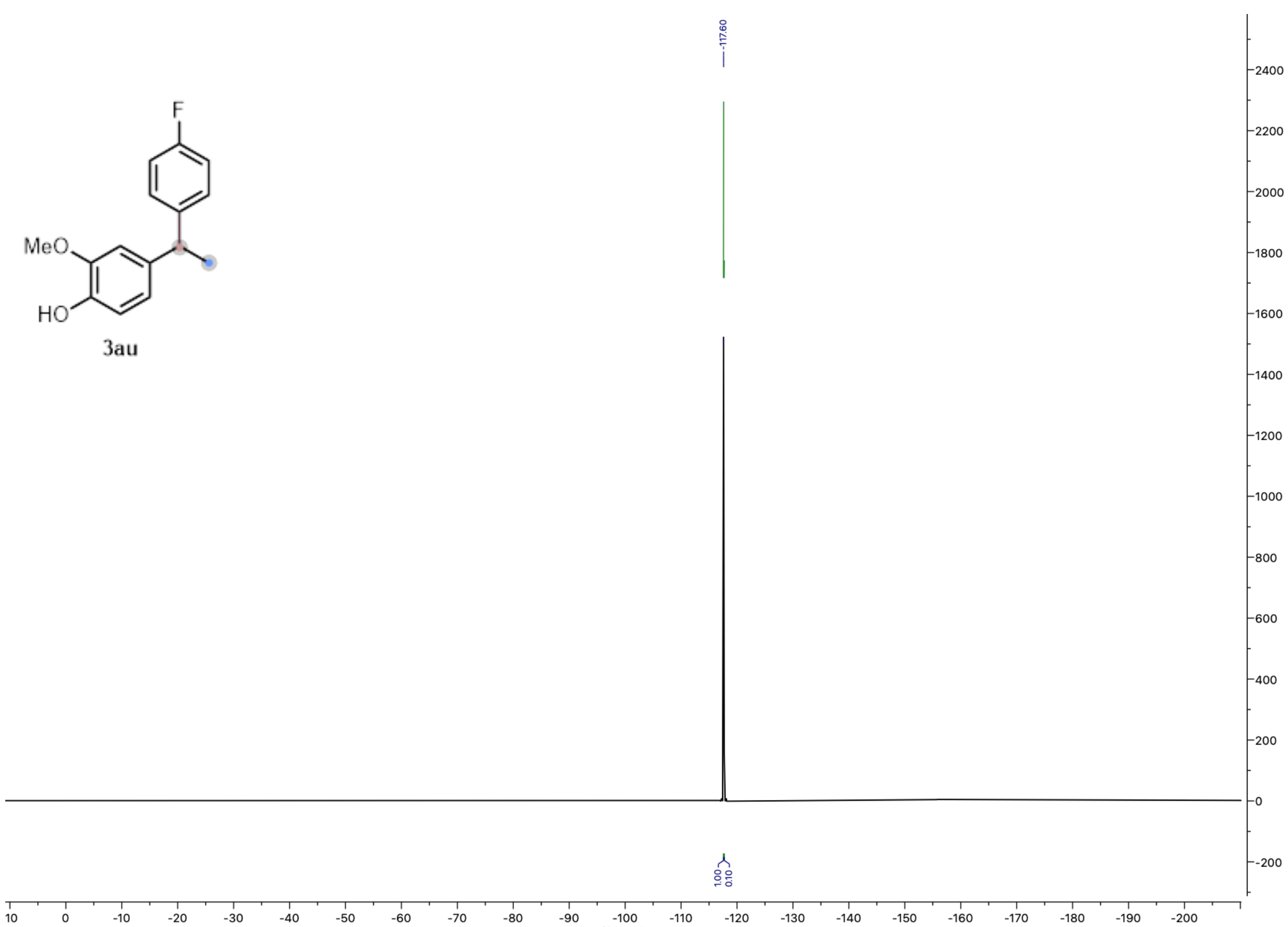

3au

3-(4-fluorophenyl)-3-phenylpropan-1-ol **3av**:

^{1}H NMR (500 MHz, $CDCl_3$):

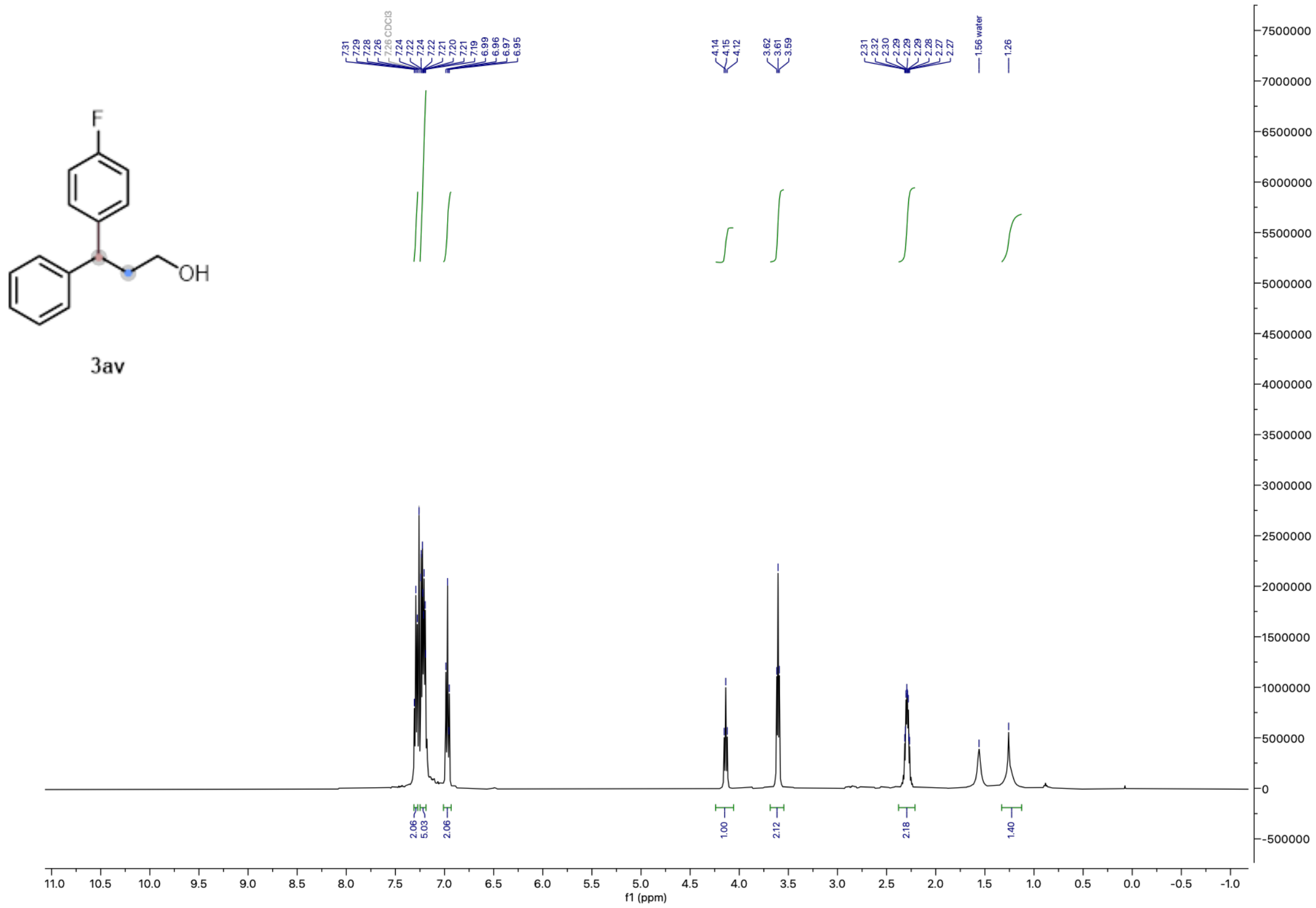

3av

^{13}C NMR (126 MHz, $CDCl_3$)

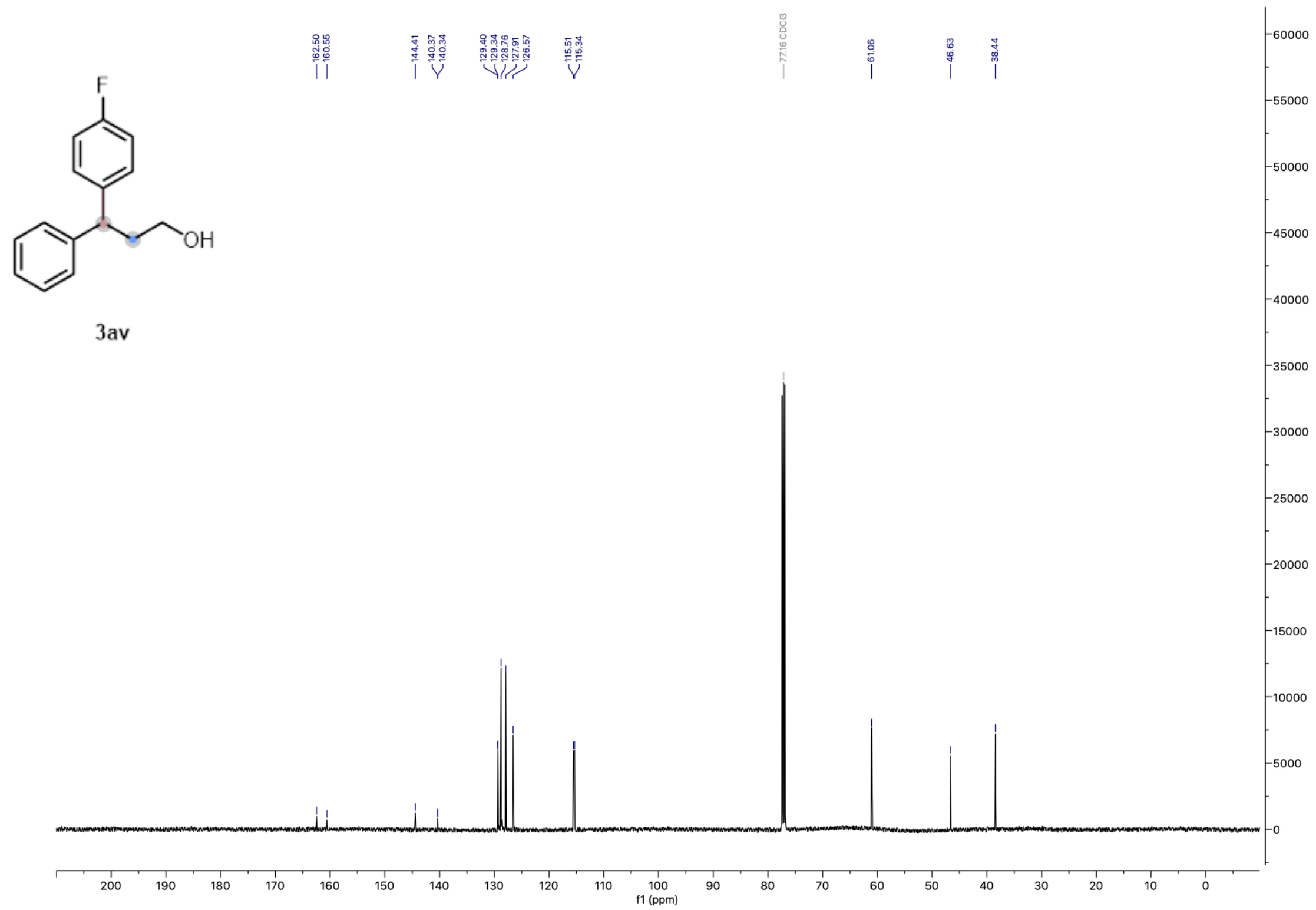

^{19}F NMR (282 MHz, $CDCl_3$):

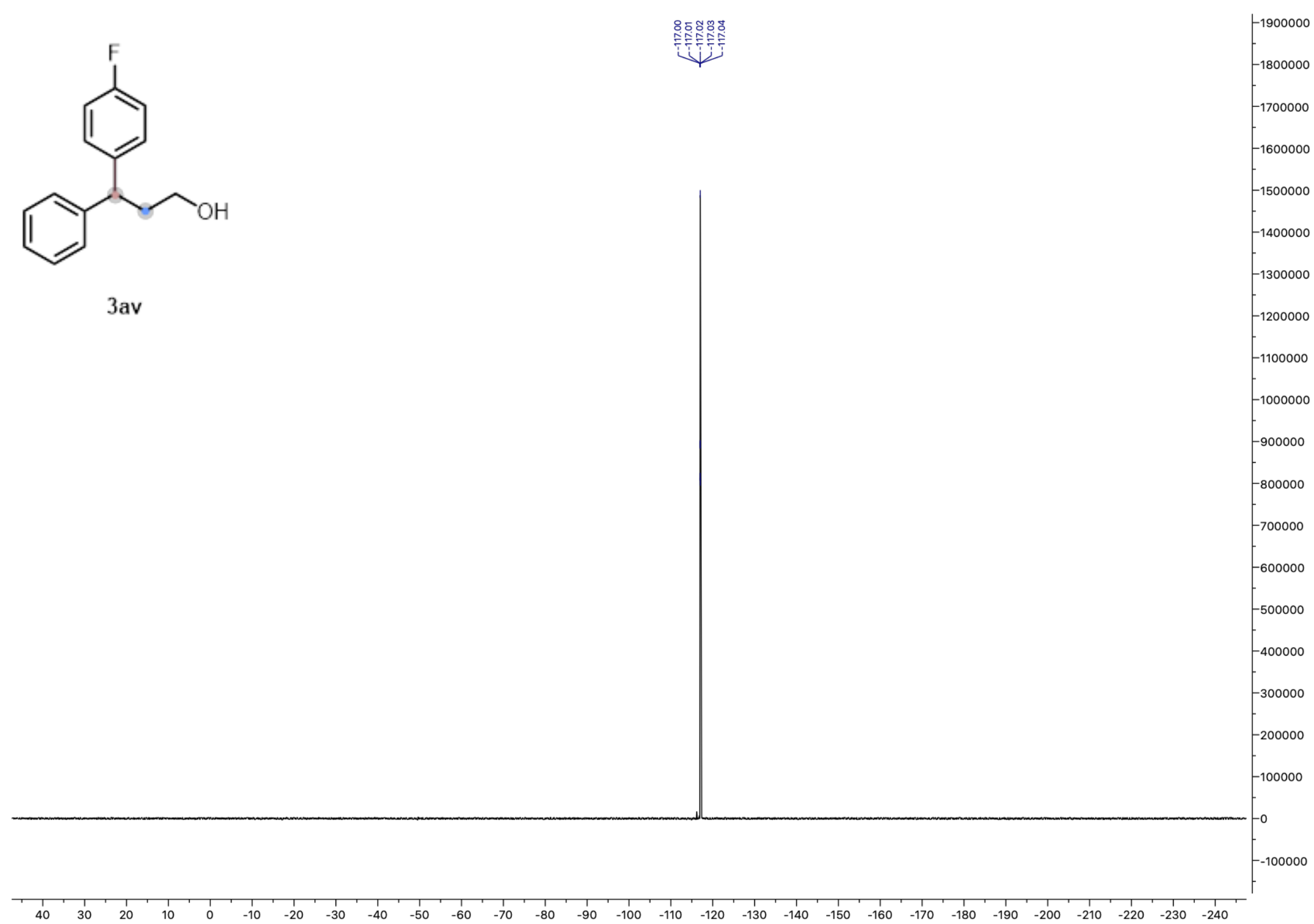

4-(1-(4-Fluorophenyl)butyl)phenyl 3-(6-(1-(2,2-difluorobenzo[*d*][1,3]dioxol-5-yl)cyclopropane-1-carboxamido)-3-methylpyridin-2-yl)benzoate **3aw**:

^{1}H NMR (500 MHz, $CDCl_3$):

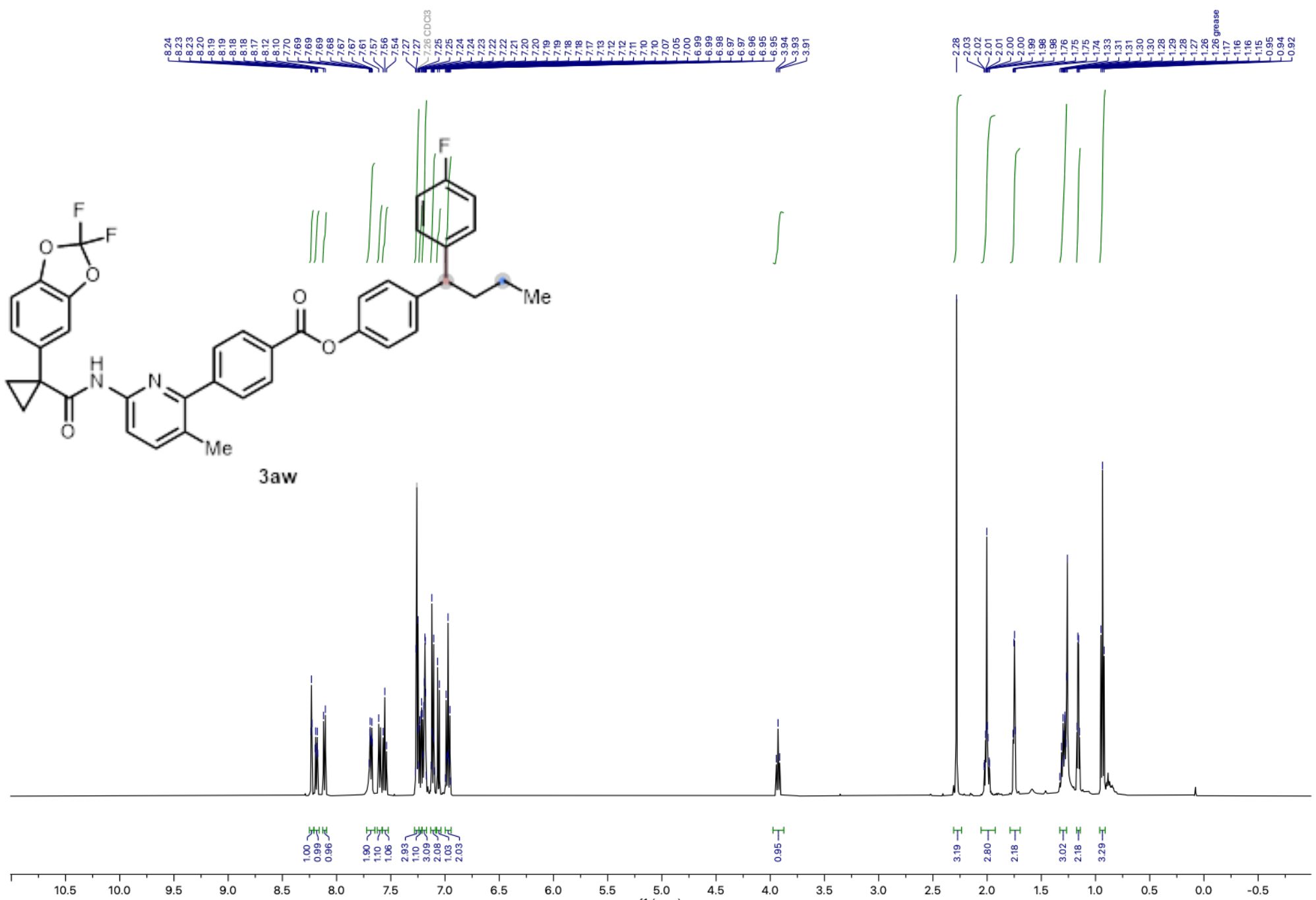

^{13}C NMR (126 MHz, $CDCl_3$):

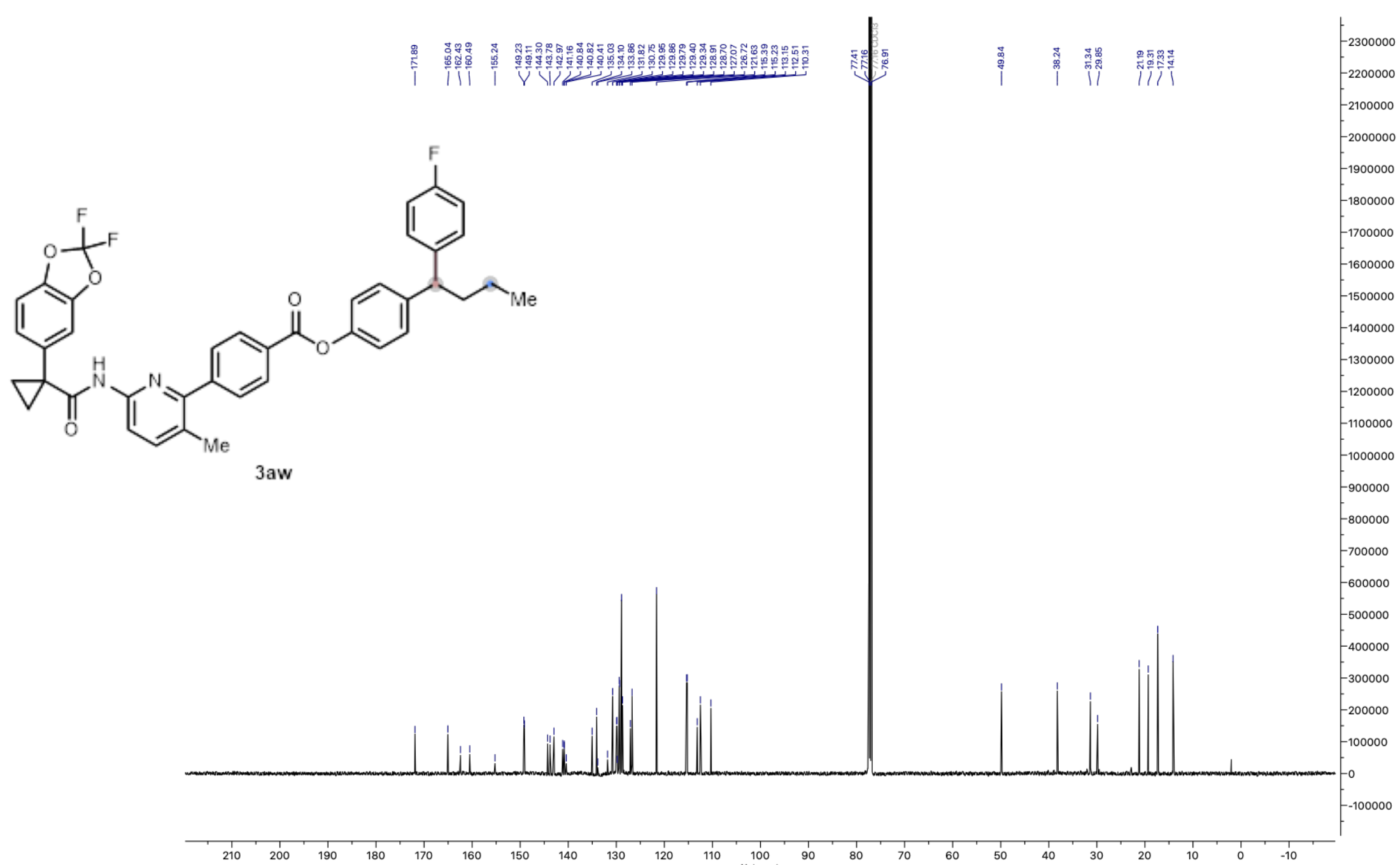

¹⁹F NMR (282 MHz, $CDCl_3$):

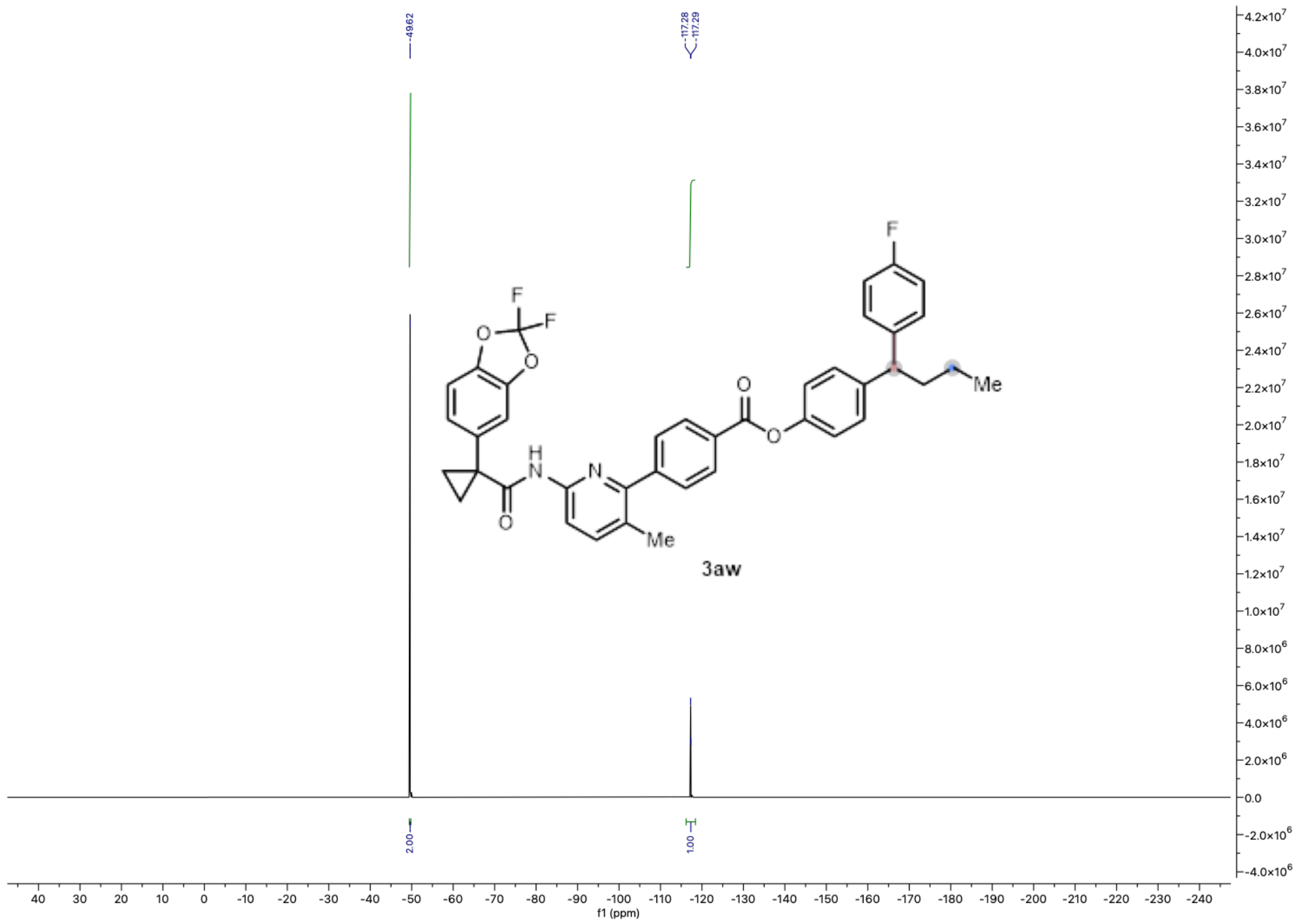

4-(1-(4-Fluorophenyl)butyl)phenyl (2*S*)-2-(6-methoxynaphthalen-2-yl)propanoate **3ax**:

^{1}H NMR (500 MHz, $CDCl_3$):

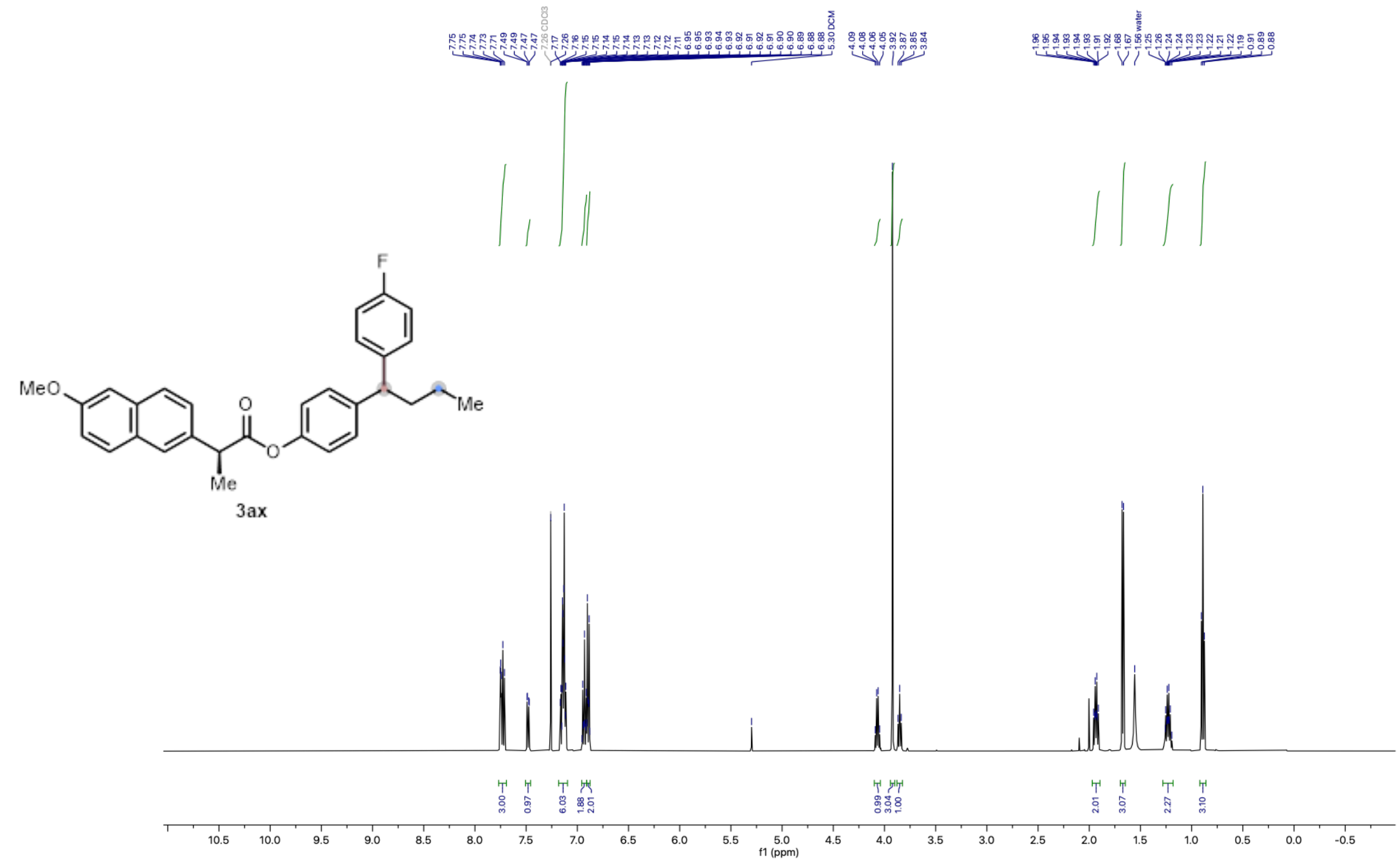

^{13}C NMR (126 MHz, $CDCl_3$):

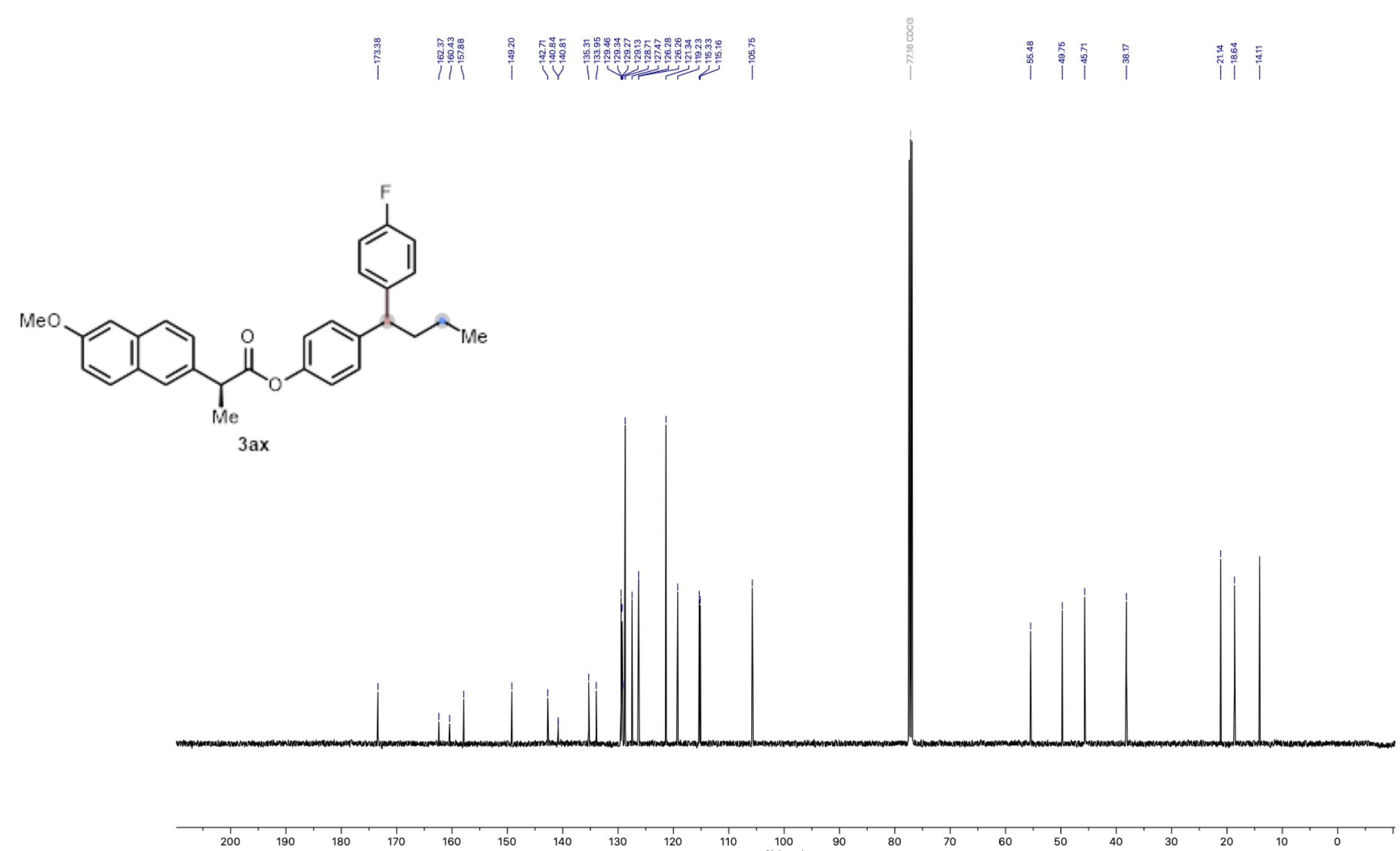

^{19}F NMR (282 MHz, $CDCl_3$):

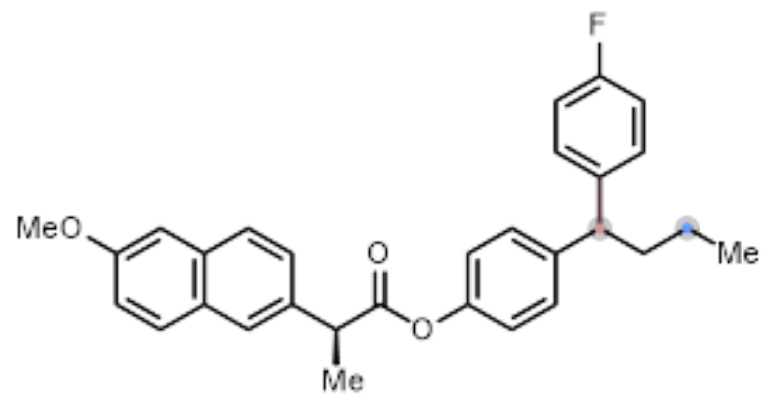

3ax

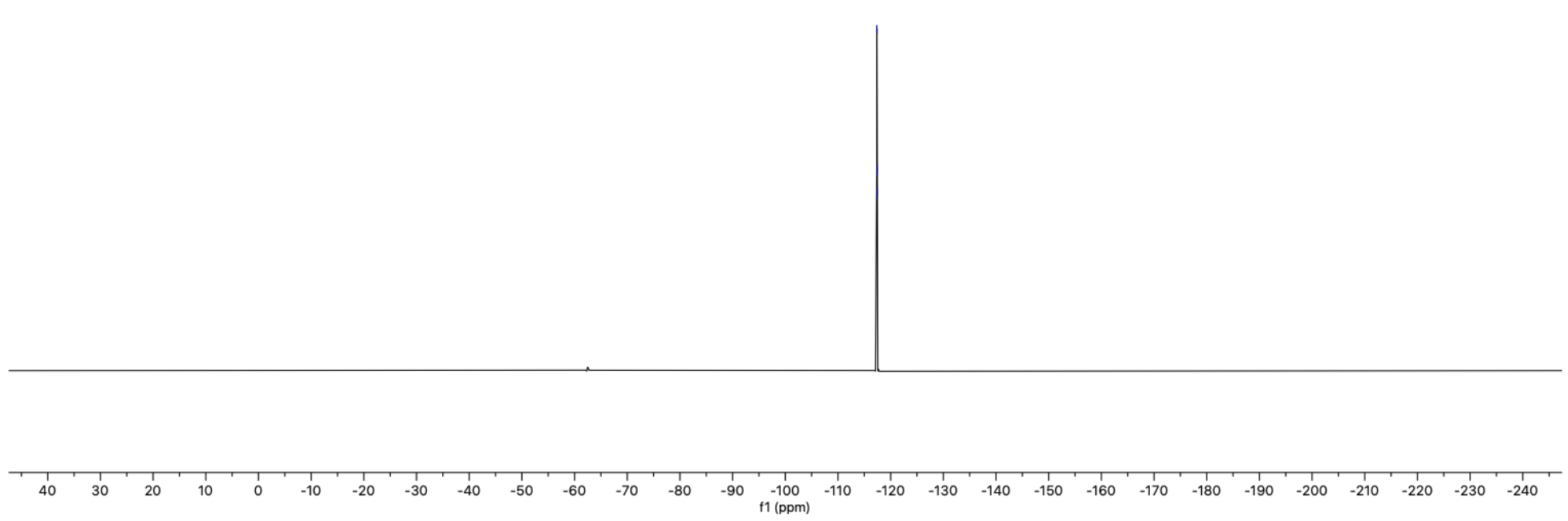

4-(1-(4-Fluorophenyl)butyl)phenyl 2-(3-cyano-4-isobutoxyphenyl)-4-methylthiazole-5-carboxylate **3ay**:

^{1}H NMR (500 MHz, $CDCl_3$):

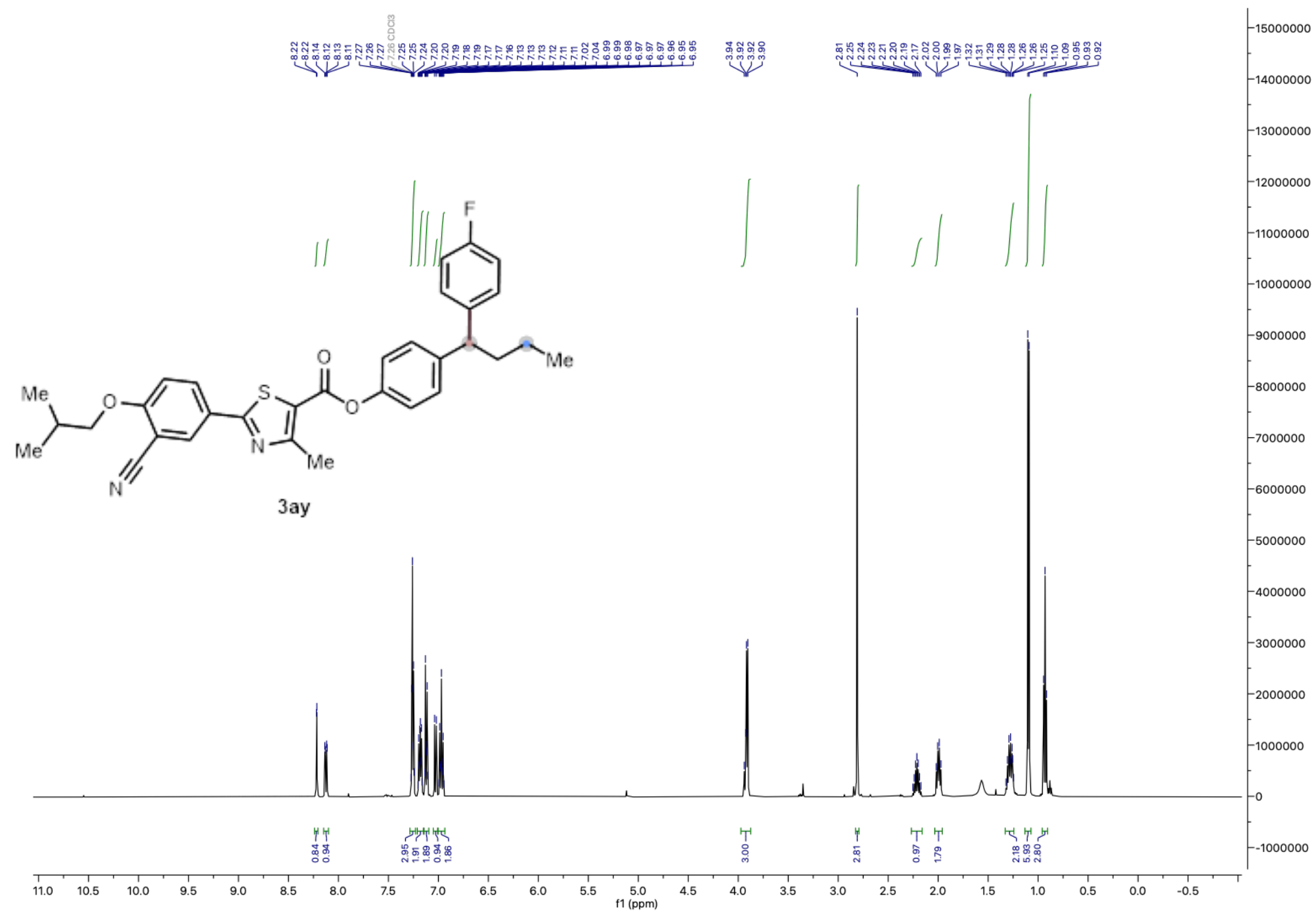

^{13}C NMR (126 MHz, $CDCl_3$):

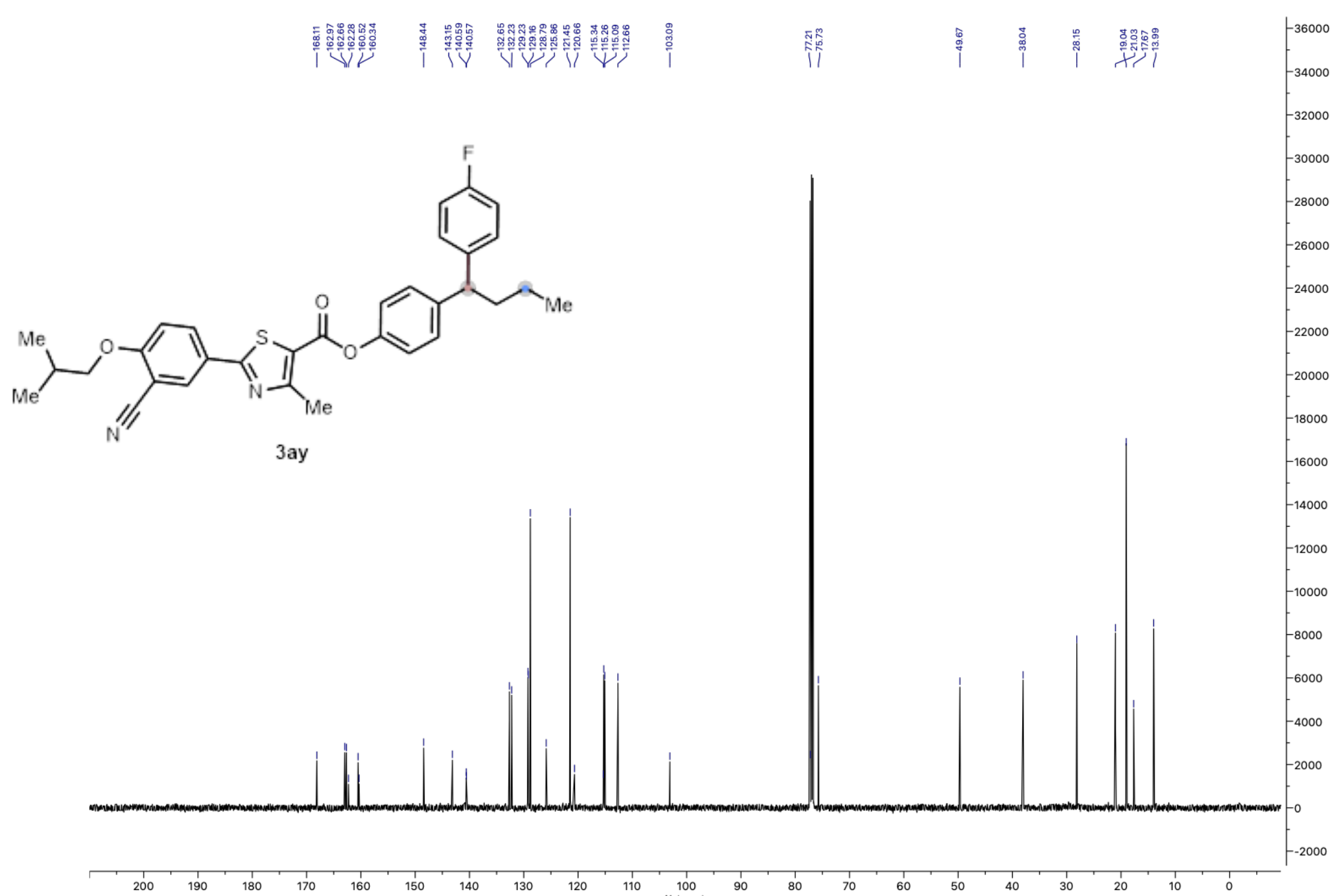

^{19}F NMR (471 MHz, $CDCl_3$):

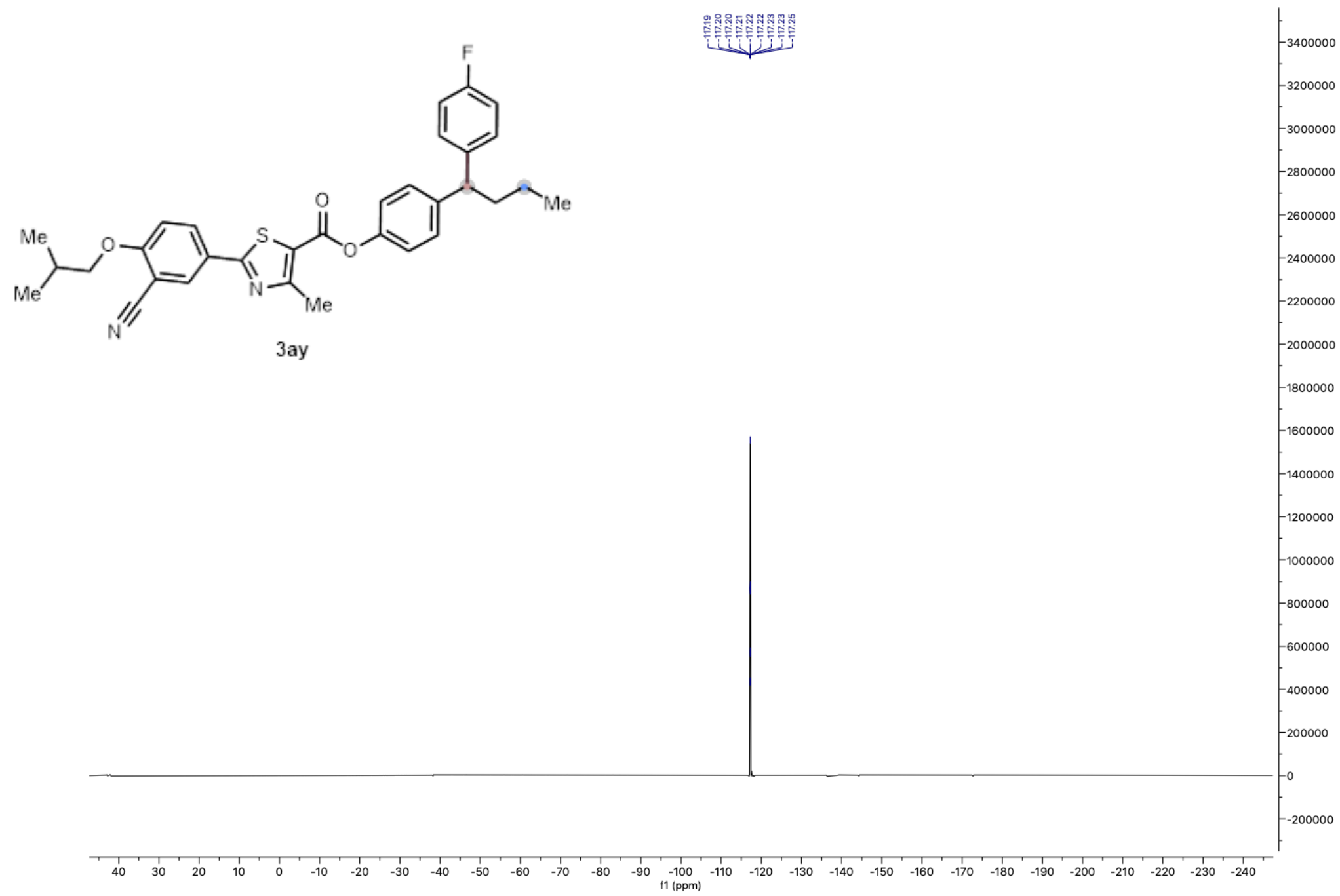

5-Benzoyl-N-(3-bromophenyl)-2,3-dihydro-1H-pyrrolizine-1-carboxamide **2ap**:

^{1}H NMR (500 MHz, $CDCl_3$):

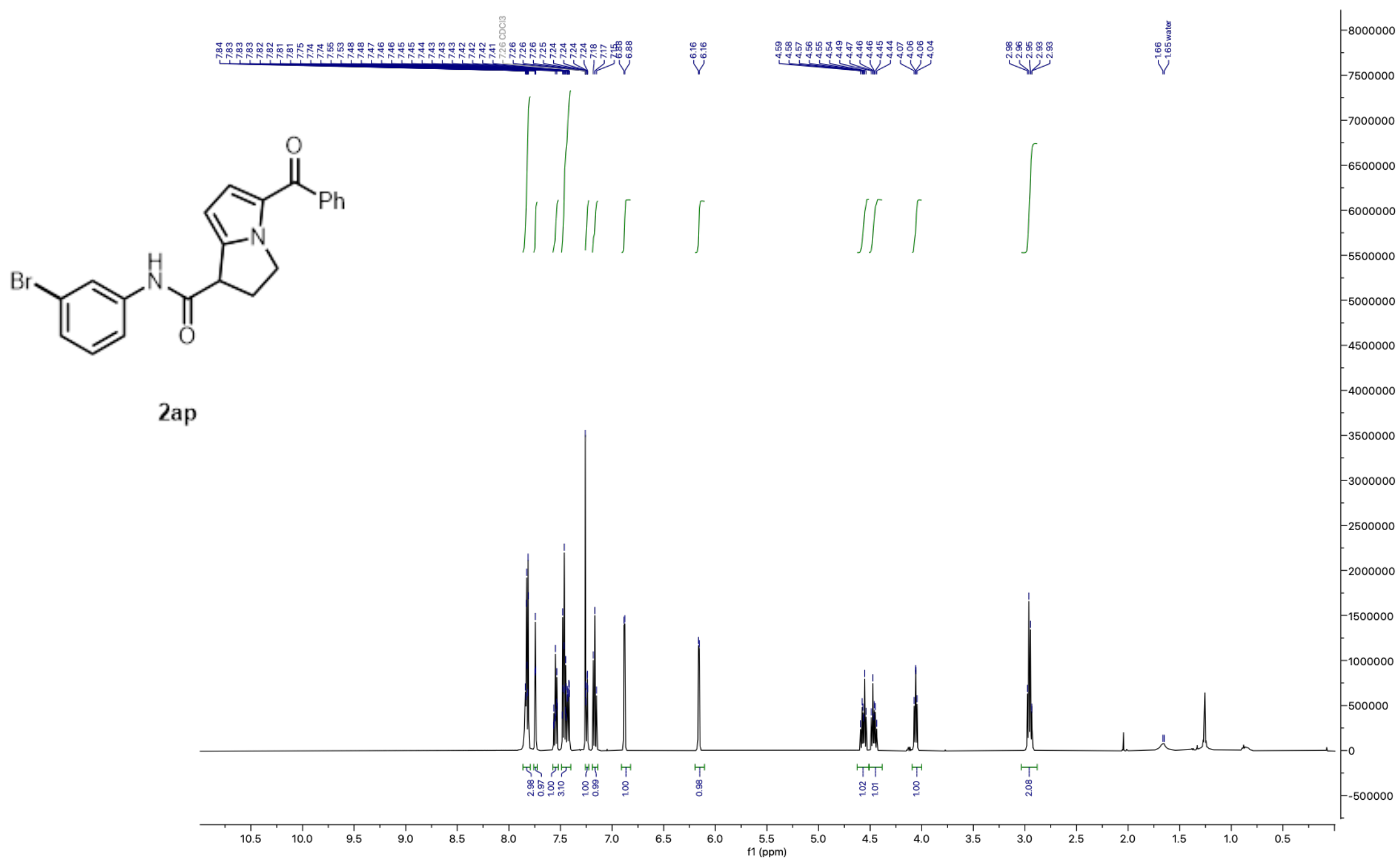

^{13}C NMR (126 MHz, $CDCl_3$):

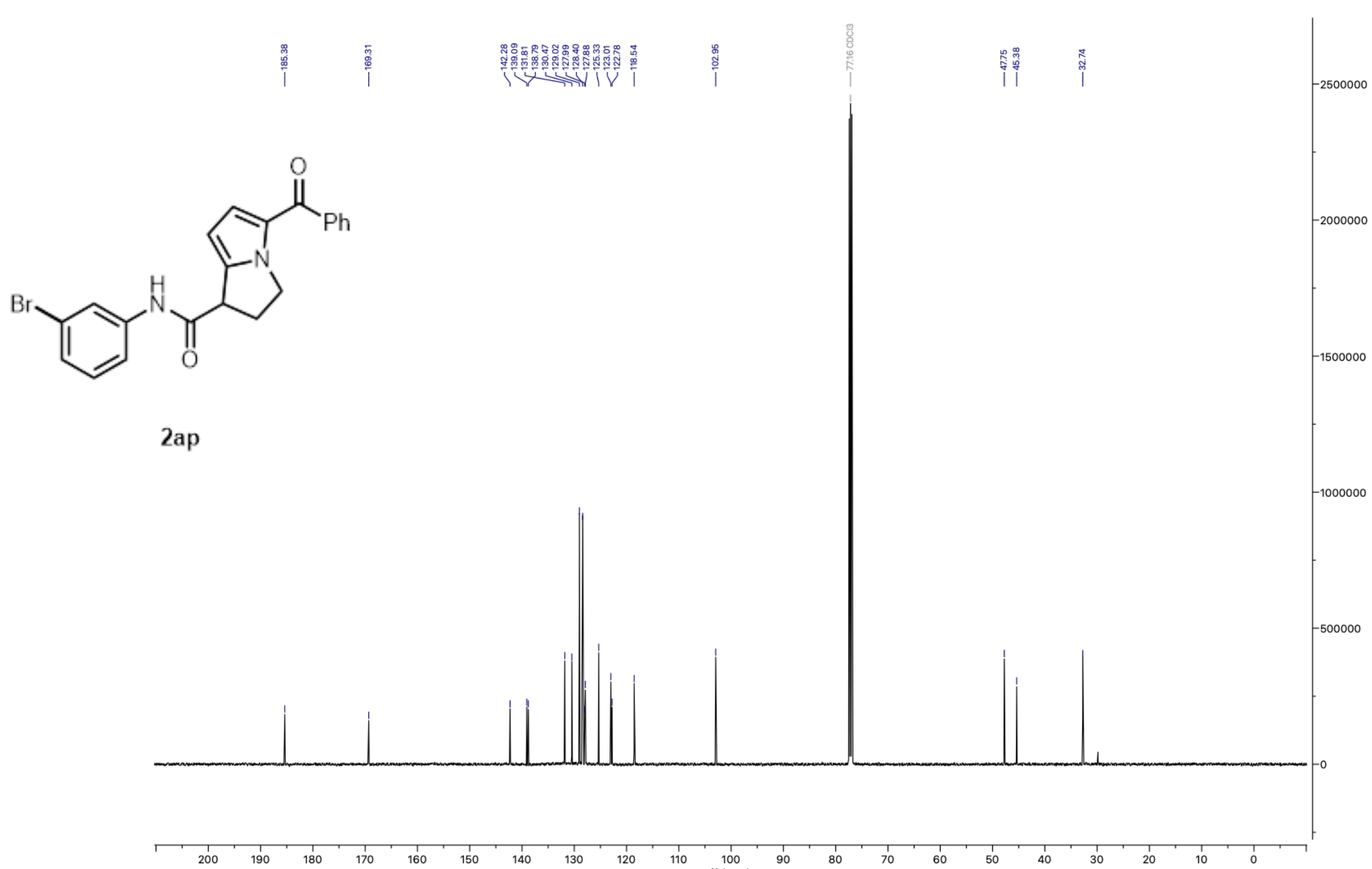

1-(4-(4-Methoxyphenyl)butan-2-yl)-2,4,6-triphenylpyridin-1-ium tetrafluoroborate **1v**:

^{1}H NMR (500 MHz, $CDCl_3$):

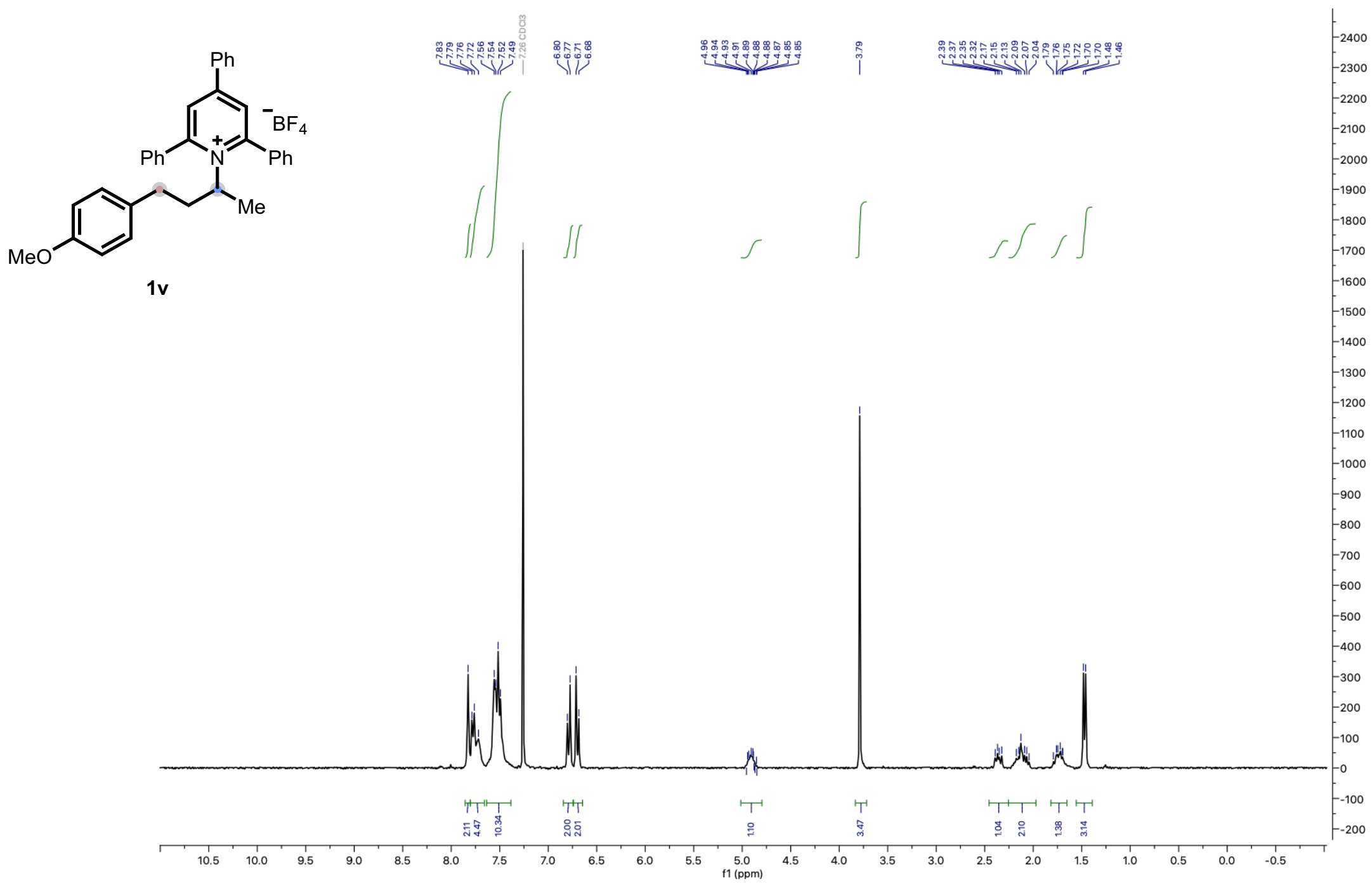

^{13}C NMR (126 MHz, $CDCl_3$):

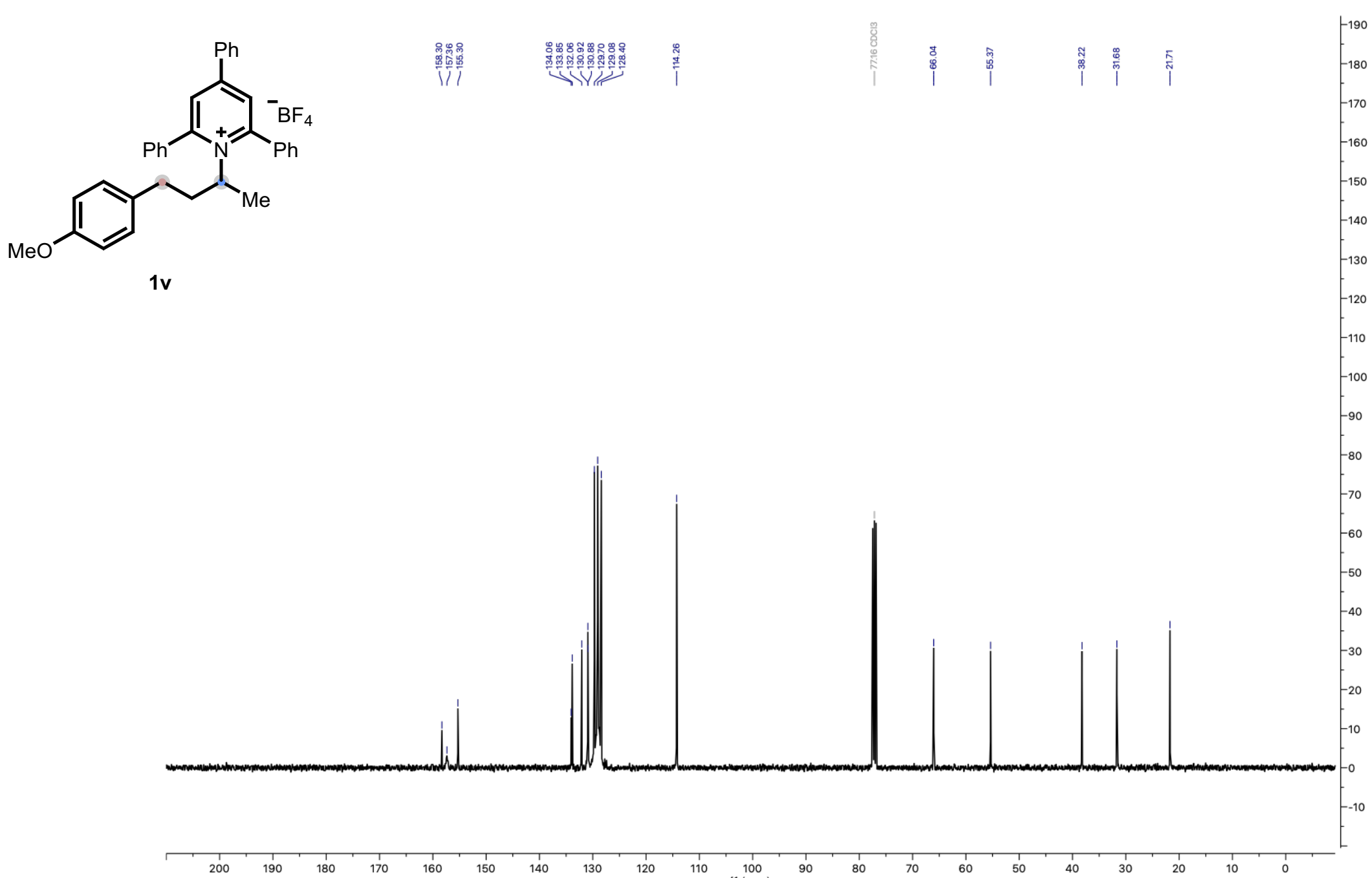

^{19}F NMR (282 MHz, $CDCl_3$):

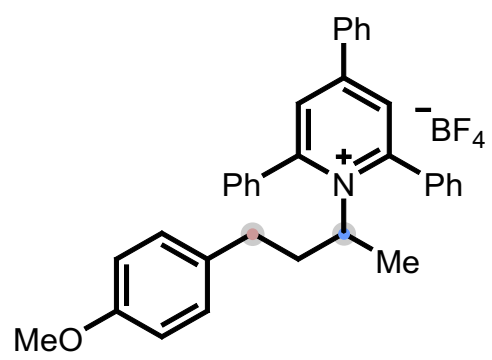

1v

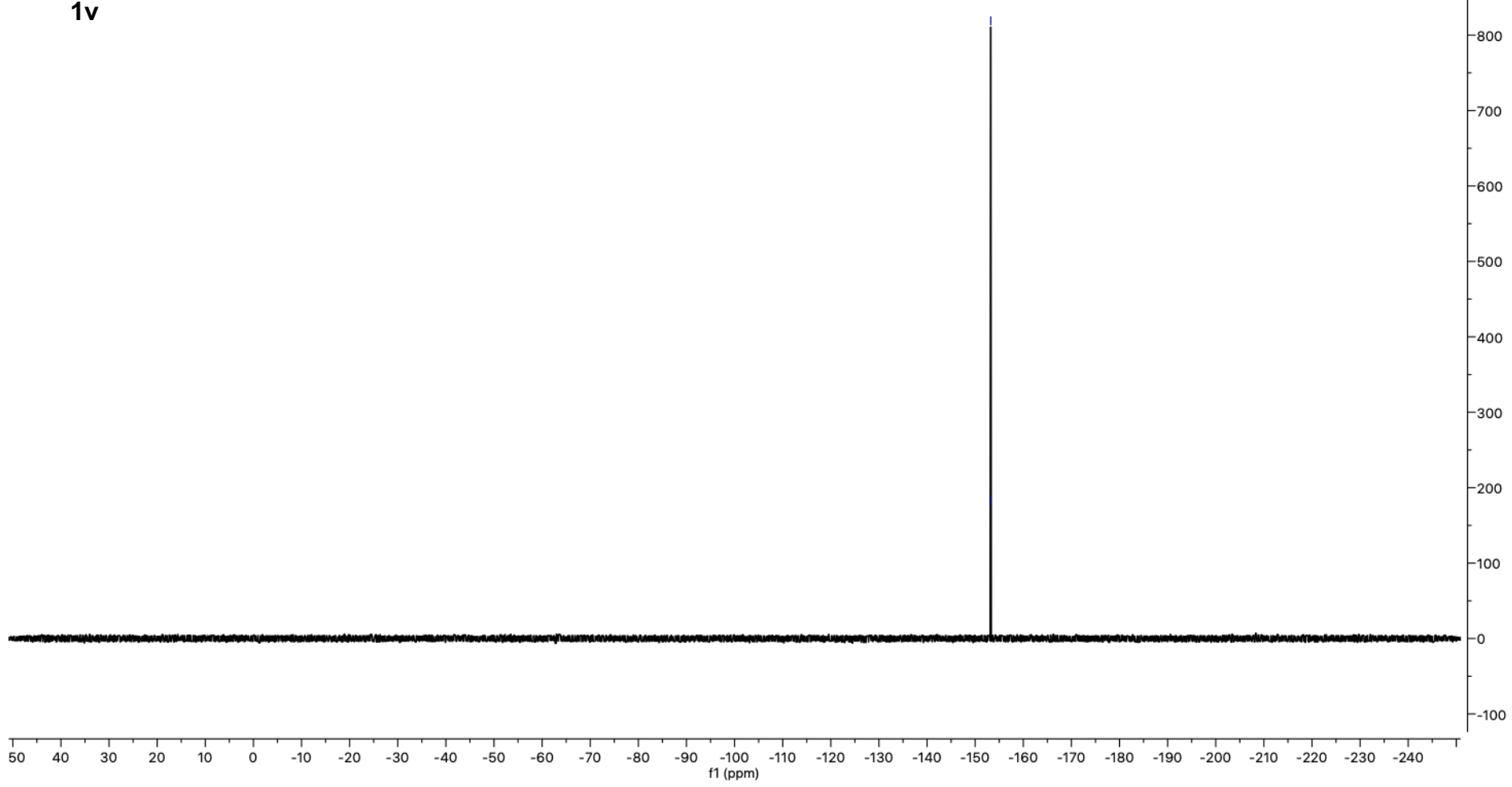

^{11}B NMR (96 MHz, $CDCl_3$):

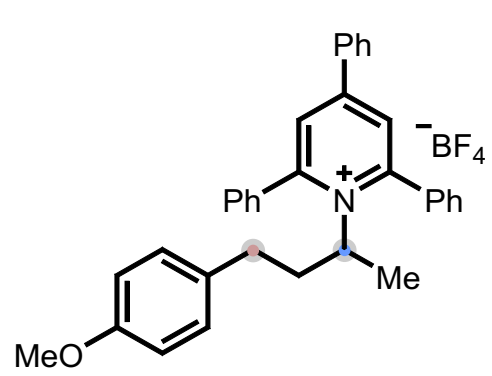

1v

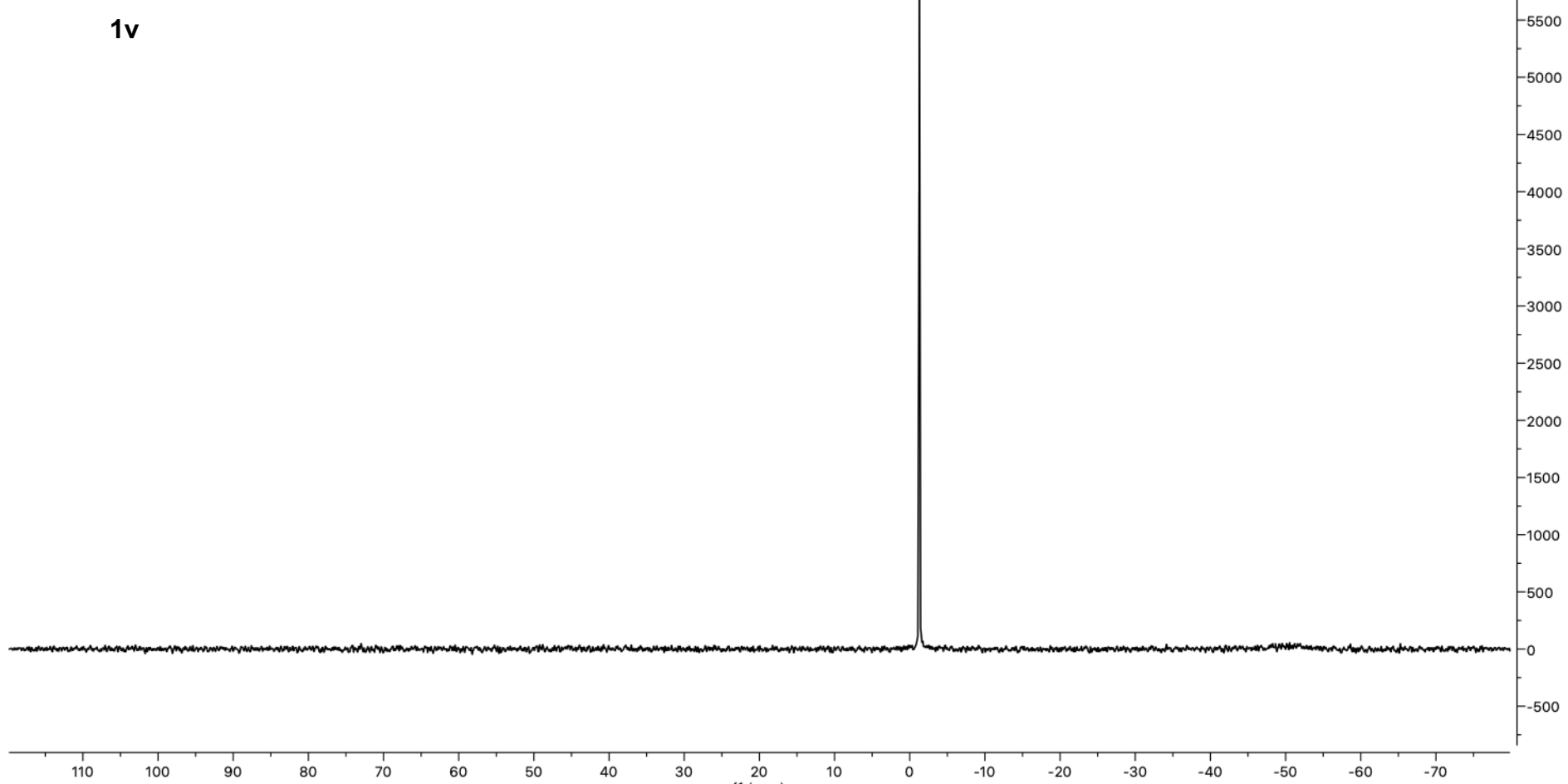

2,4,6-triphenyl-1-(1-phenylheptan-3-yl)pyridin-1-ium tetrafluoroborate **3w:**

^{1}H NMR (500 MHz, $CDCl_3$):

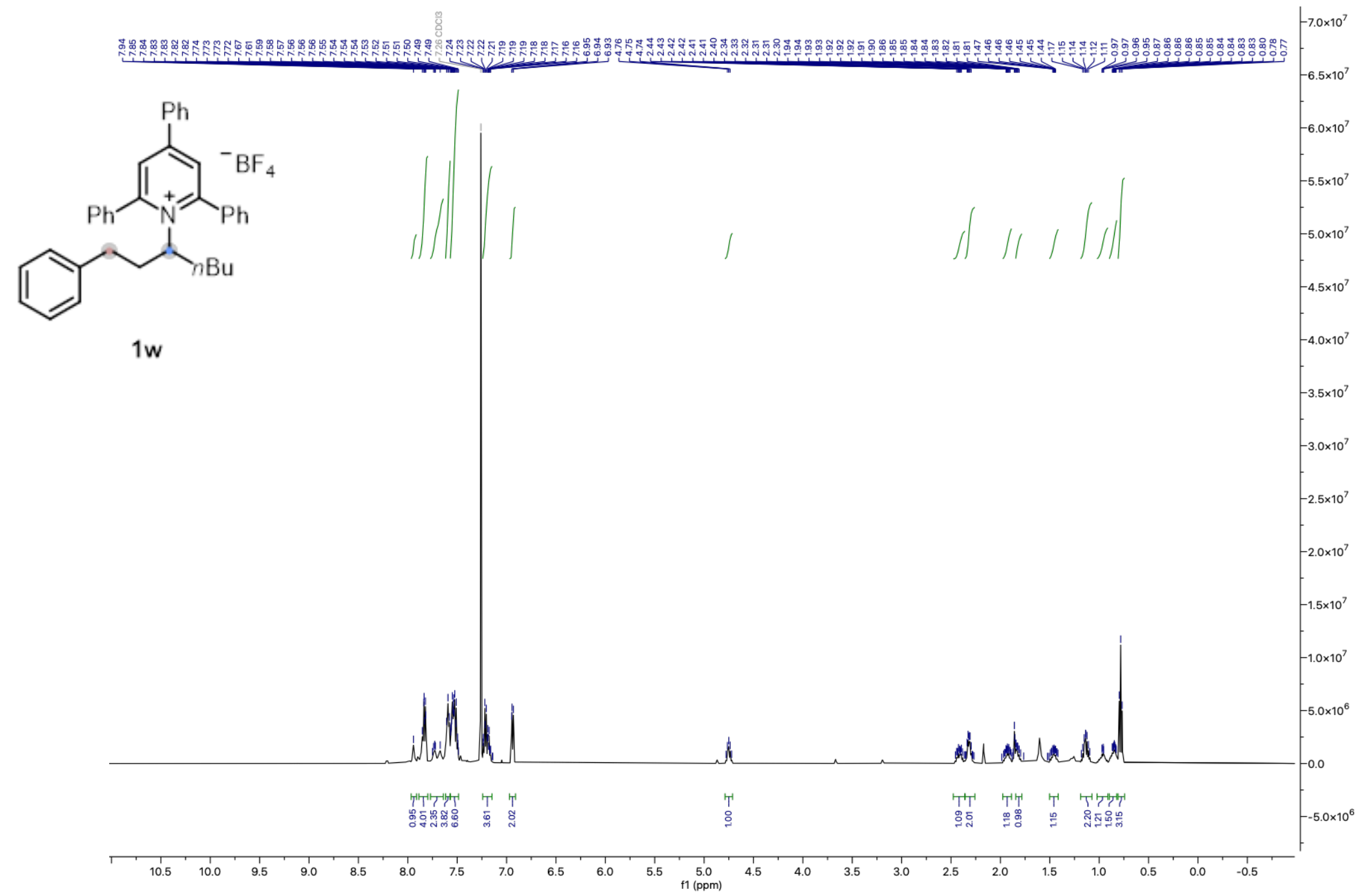

^{13}C NMR (126 MHz, $CDCl_3$):

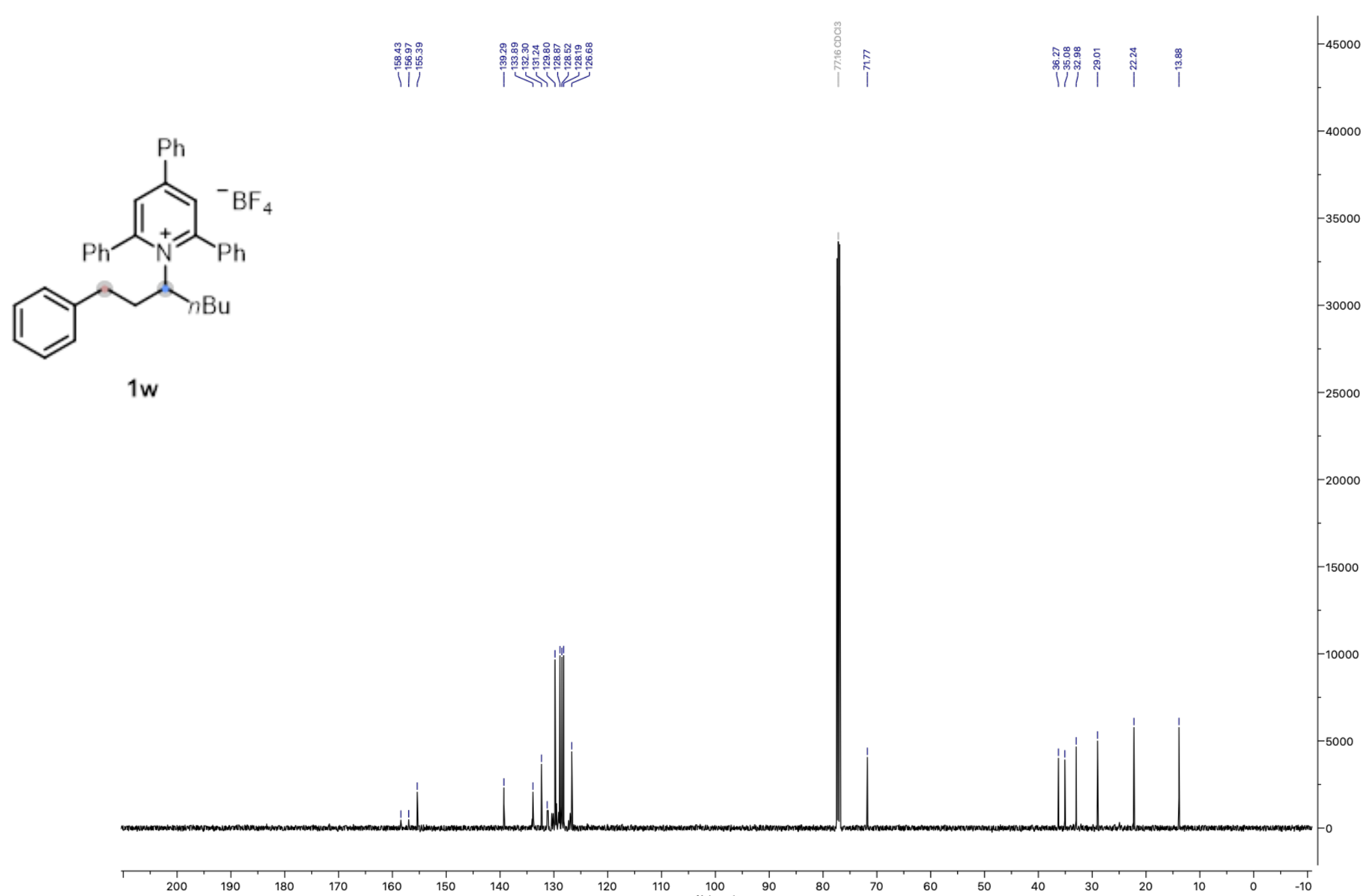

^{19}F NMR (282 MHz, $CDCl_3$):

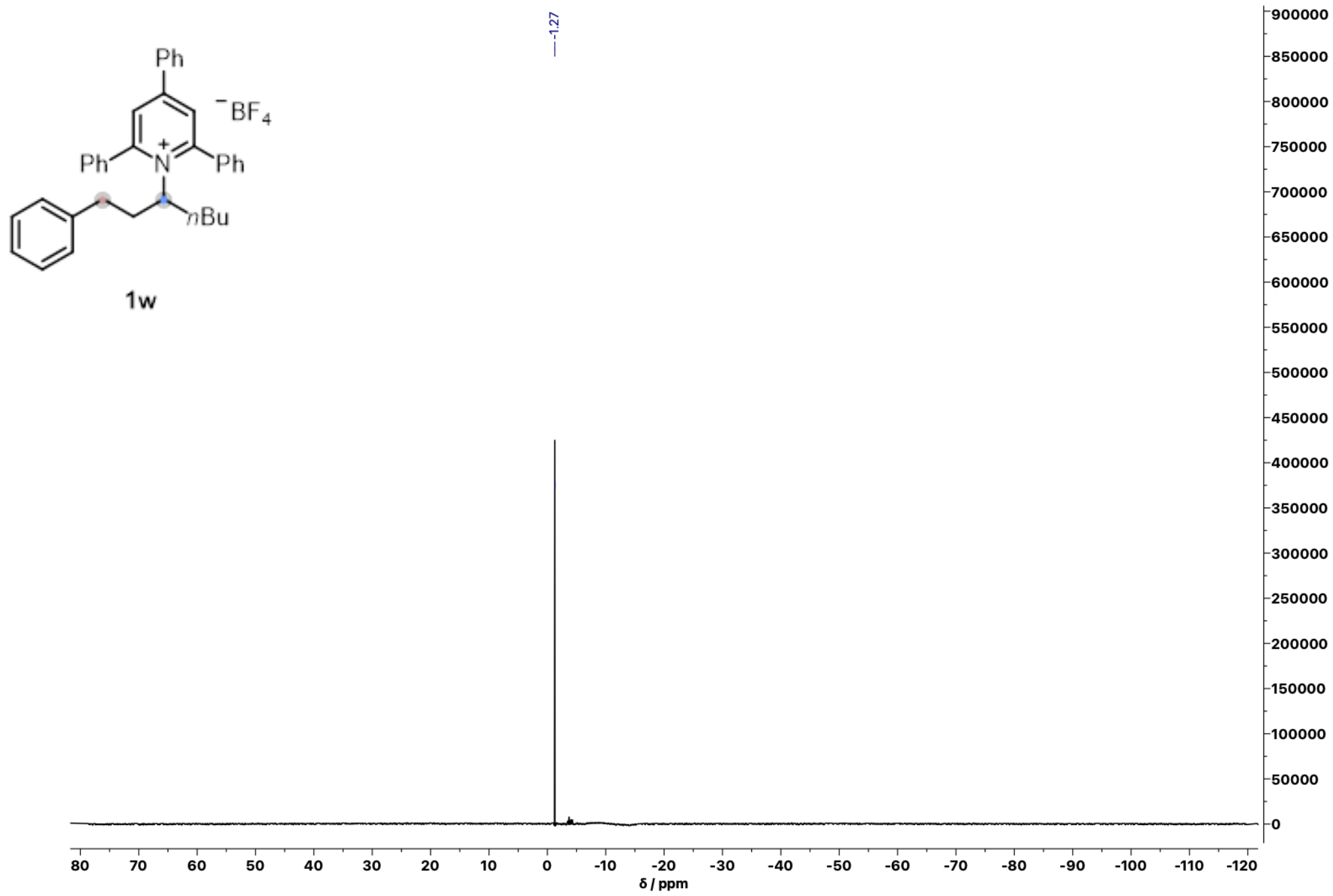

^{11}B NMR (96 MHz, $CDCl_3$):

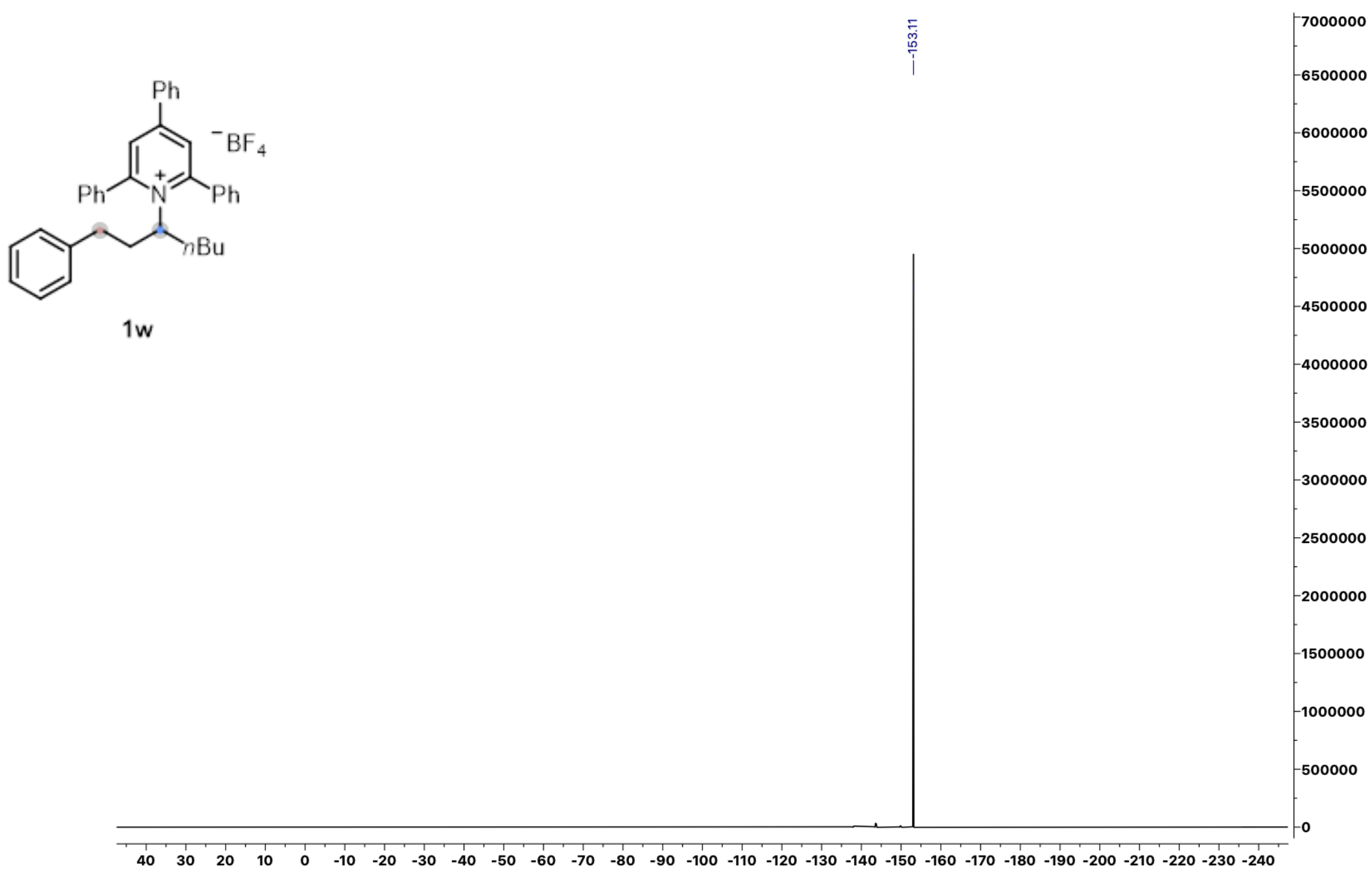

2,4,6-Triphenyl-1-(5-phenylpentan-2-yl)pyridin-1-ium tetrafluoroborate **1x**:

^{1}H NMR (500 MHz, $(CD_3)_2CO$):

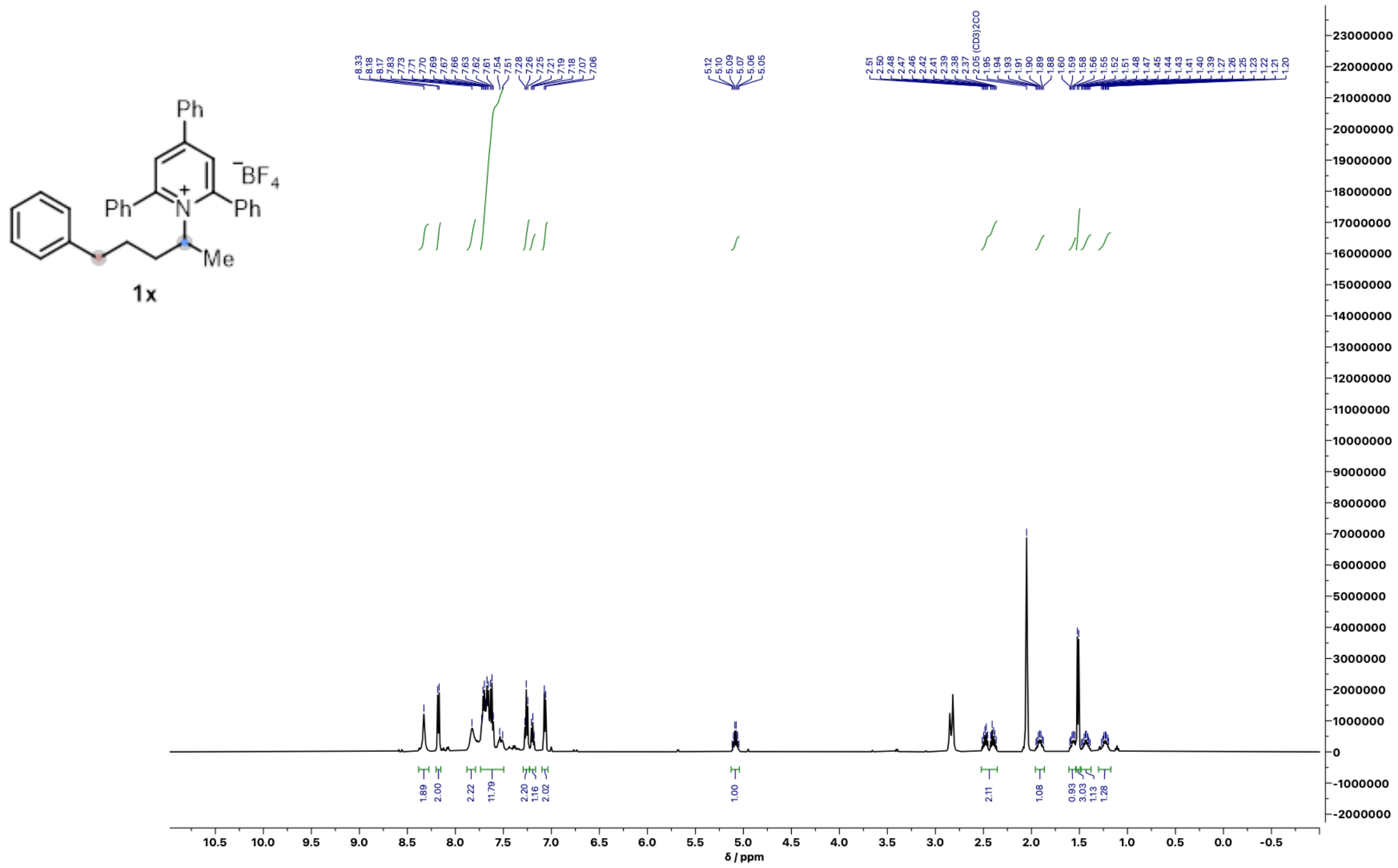

^{13}C NMR (126 MHz, $(CD_3)_2CO$):

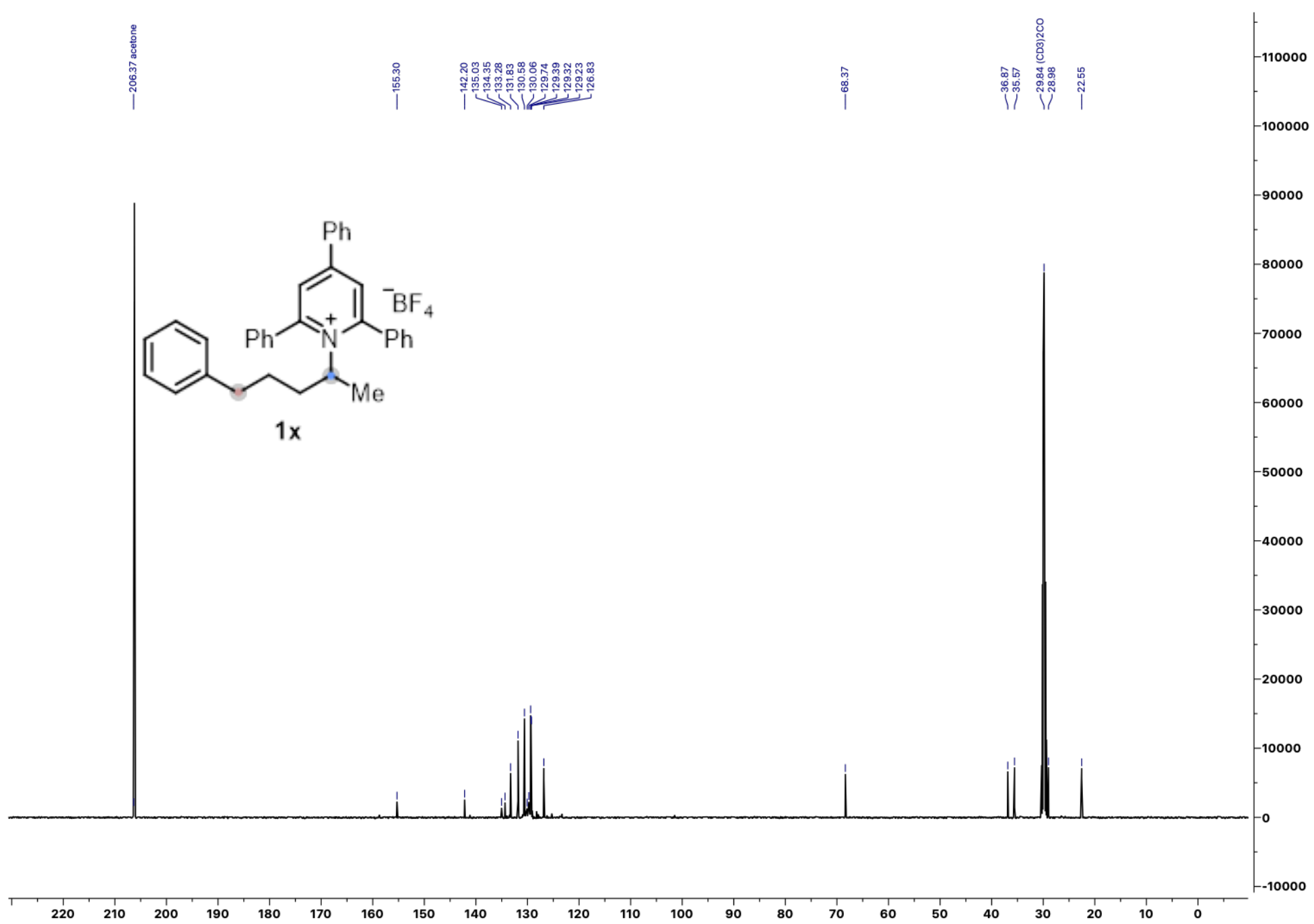

^{19}F NMR (282 MHz, $(CD_3)_2CO$):

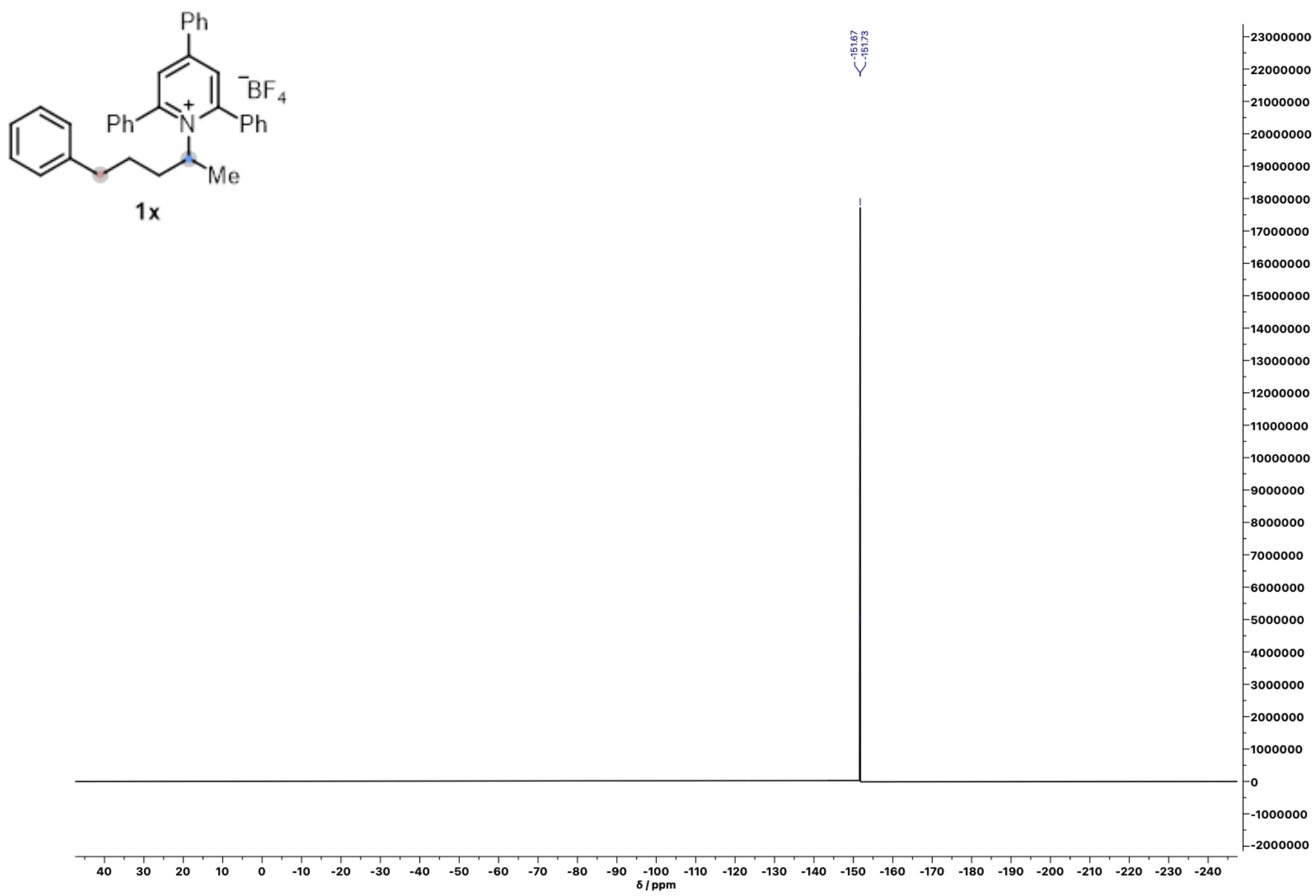

^{11}B NMR (96 MHz, $(CD_3)_2CO$):

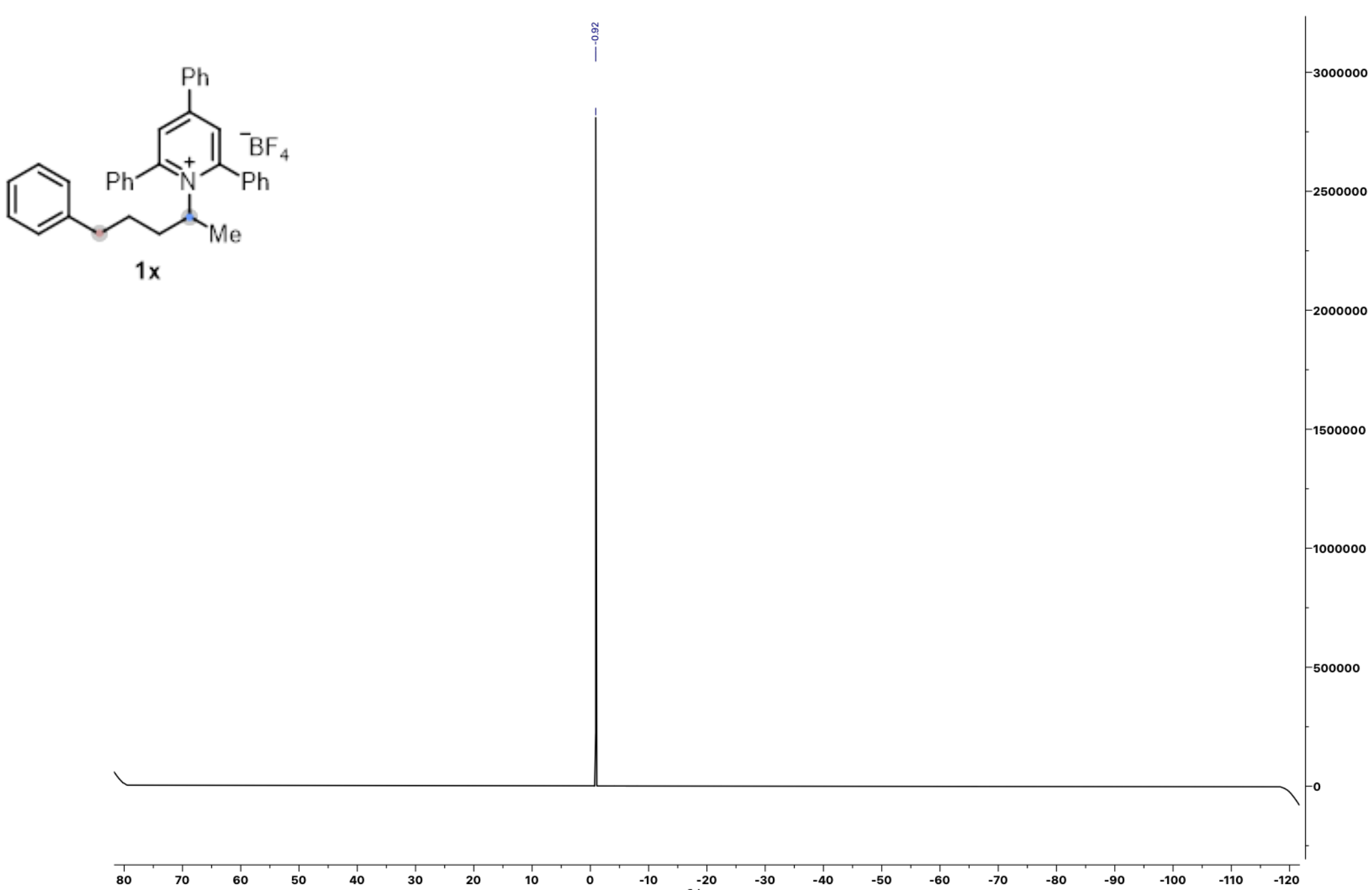

1-(4-(4-Hydroxyphenyl)butan-2-yl)-2,4,6-triphenylpyridin-1-ium tetrafluoroborate **1y**:

^{1}H NMR (500 MHz, $CDCl_3$):

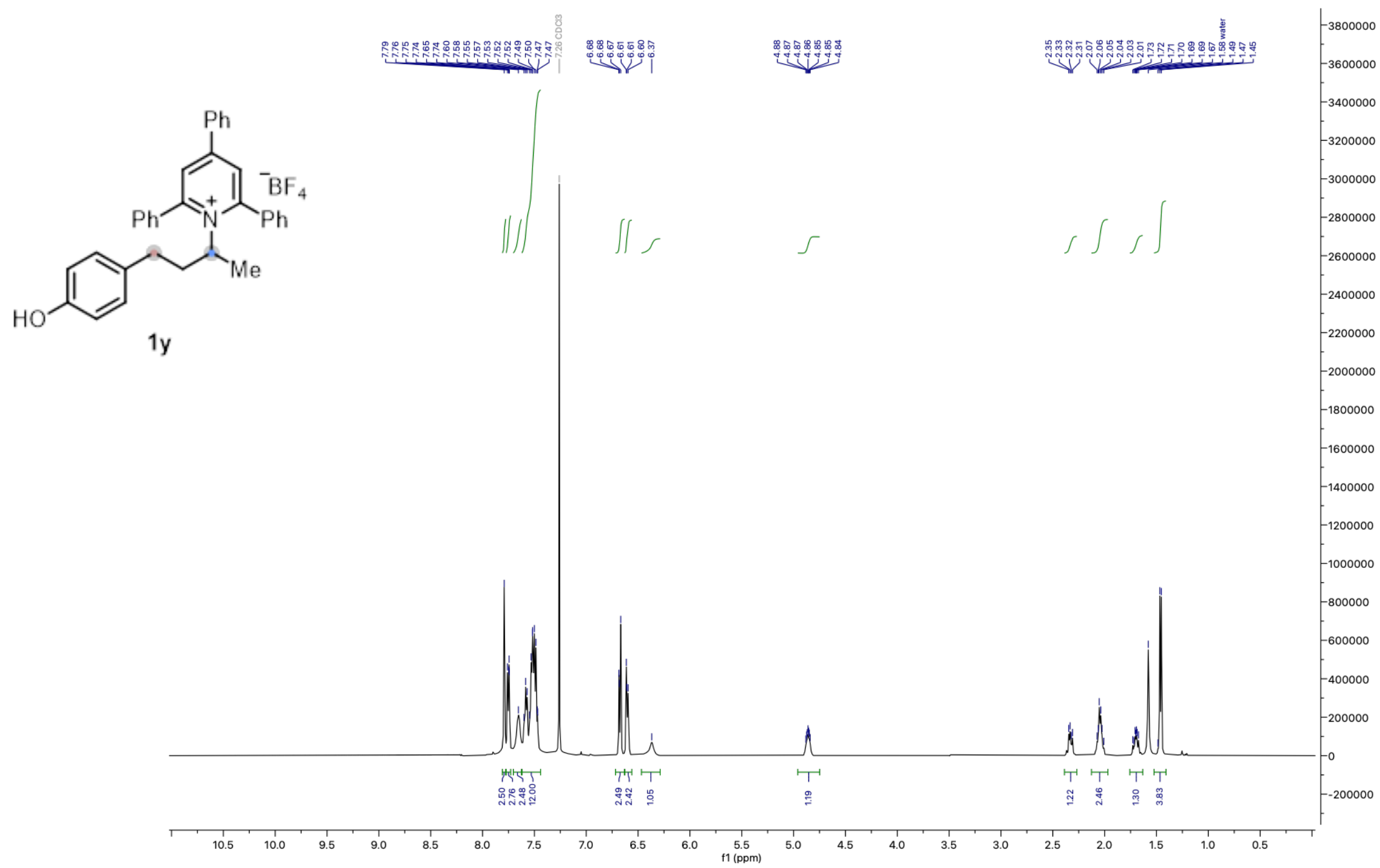

^{13}C NMR (126 MHz, $CDCl_3$):

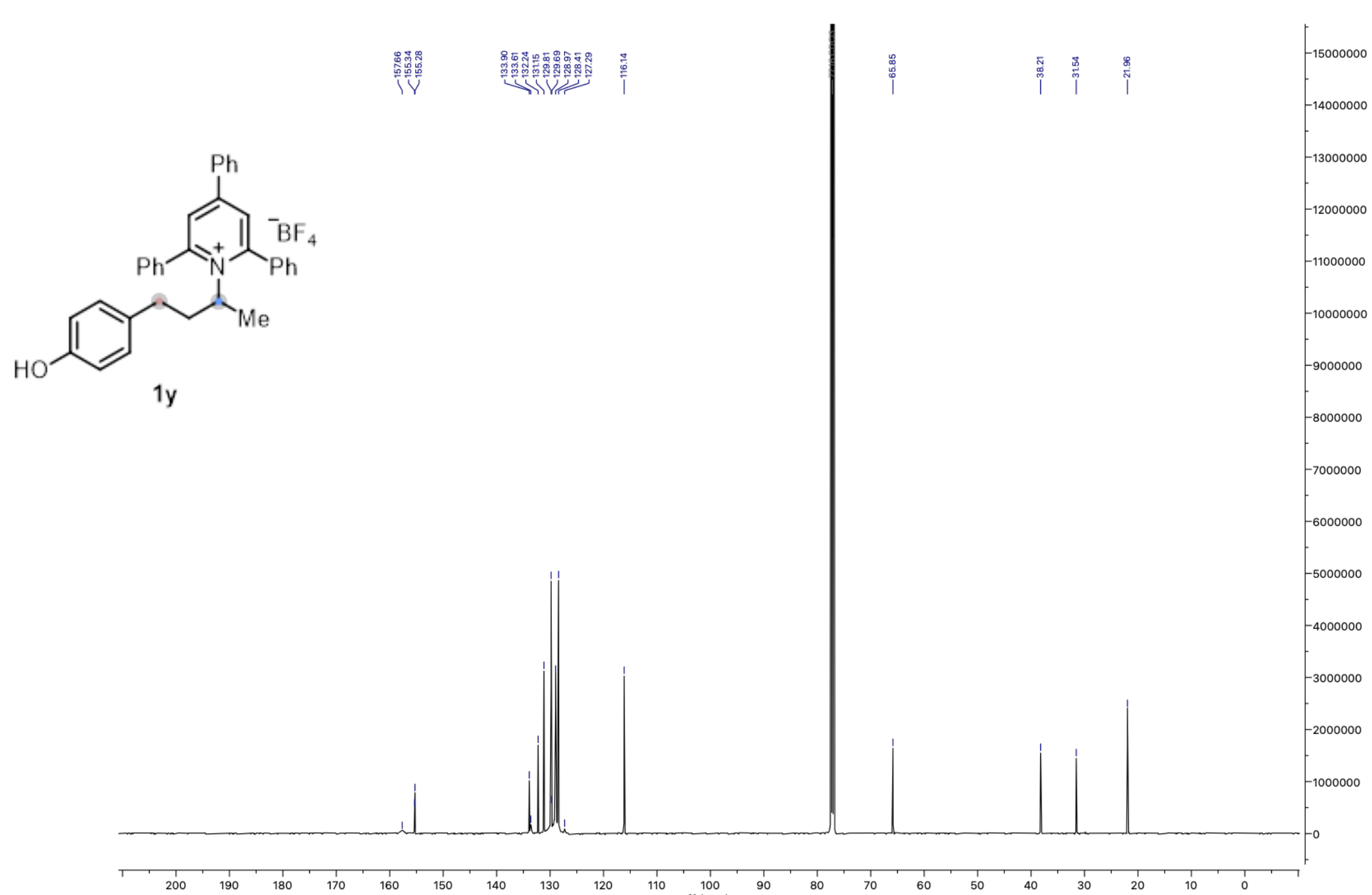

^{19}F NMR (282 MHz, $CDCl_3$):

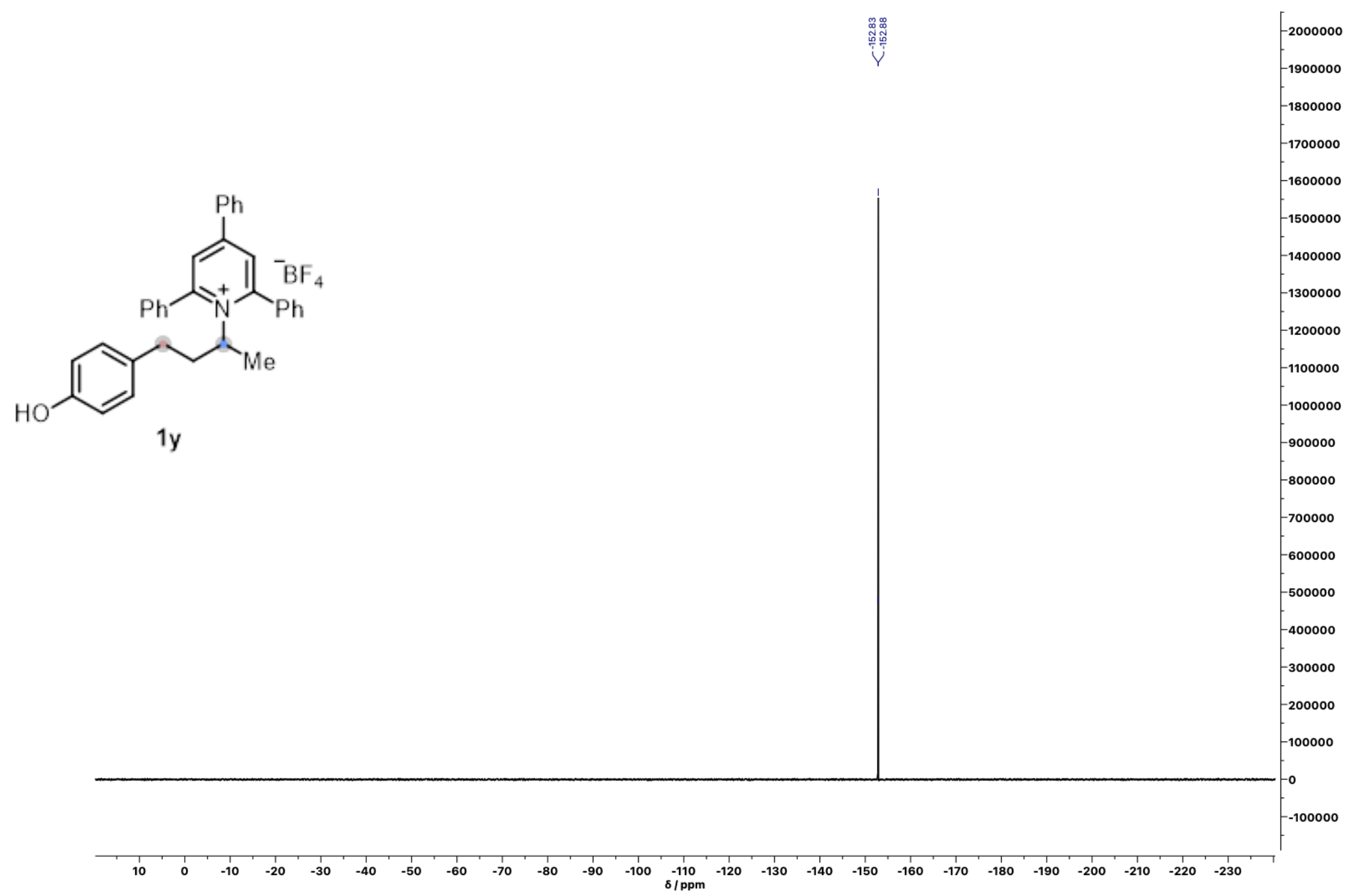

^{11}B NMR (96 MHz, $CDCl_3$):

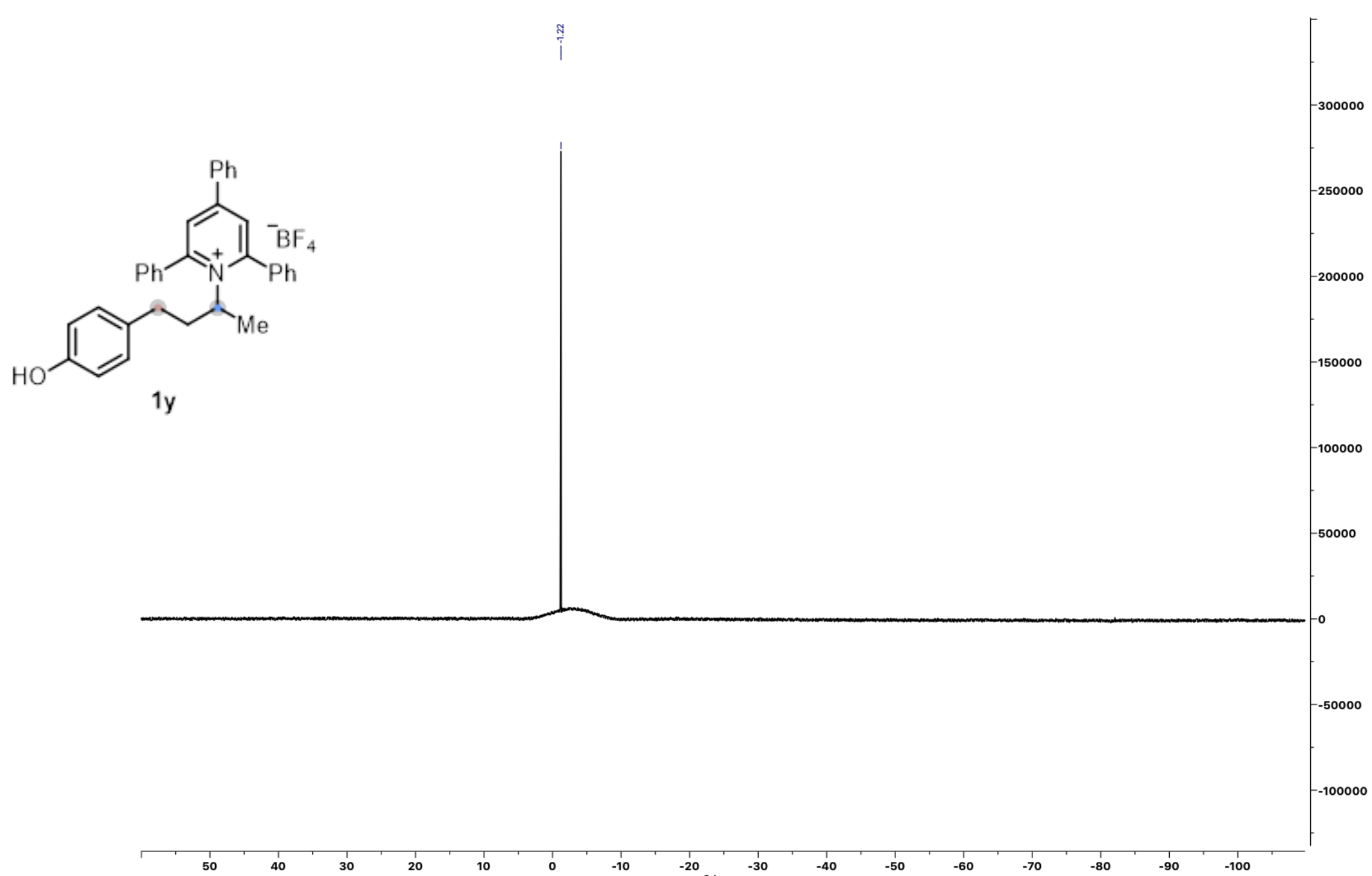

1-(1-(4-Chlorophenyl)propan-2-yl)-2,4,6-triphenylpyridin-1-ium tetrafluoroborate **1z**:

^{1}H NMR (500 MHz, $CDCl_3$):

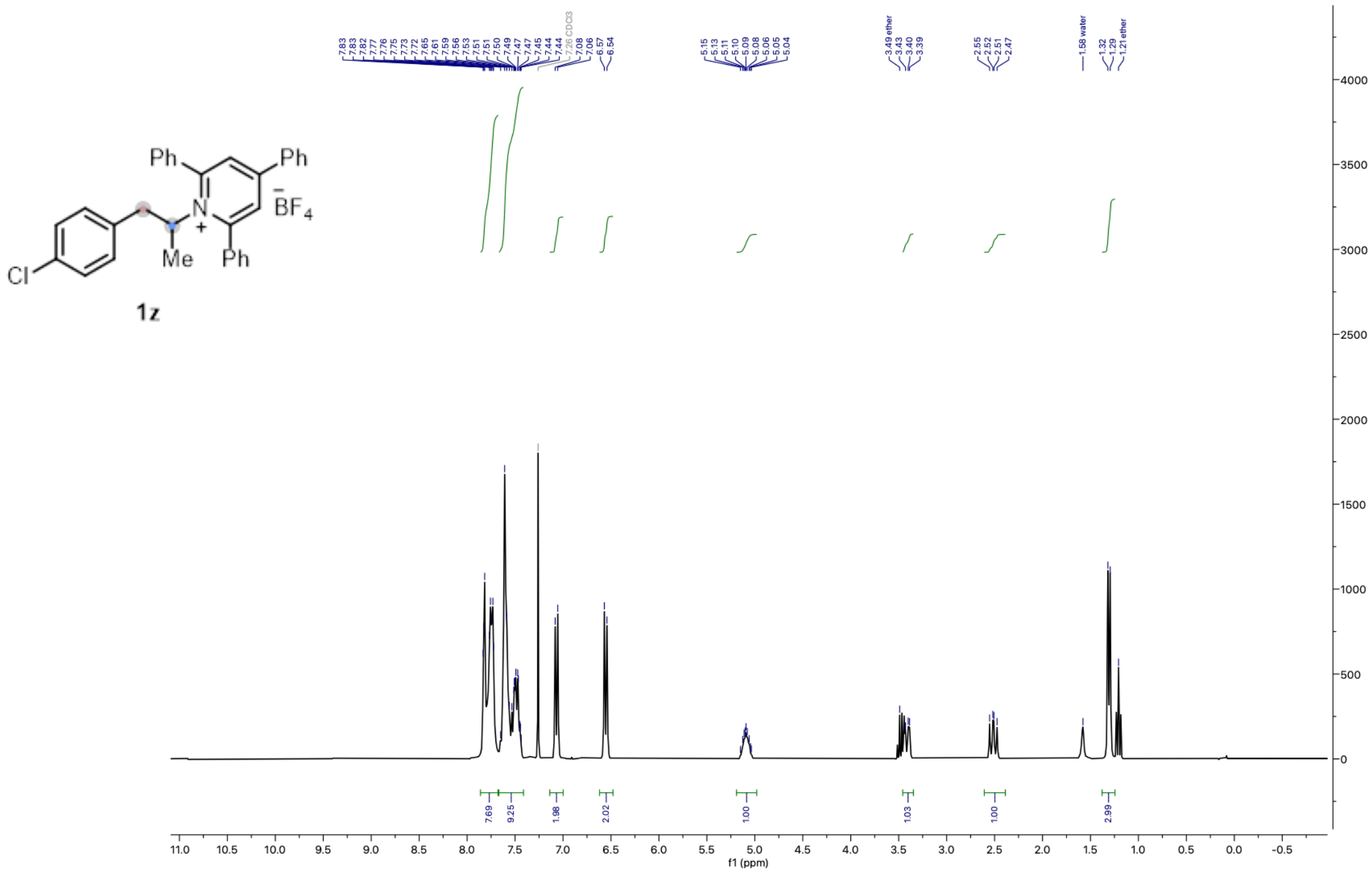

^{13}C NMR (126 MHz, $CDCl_3$):

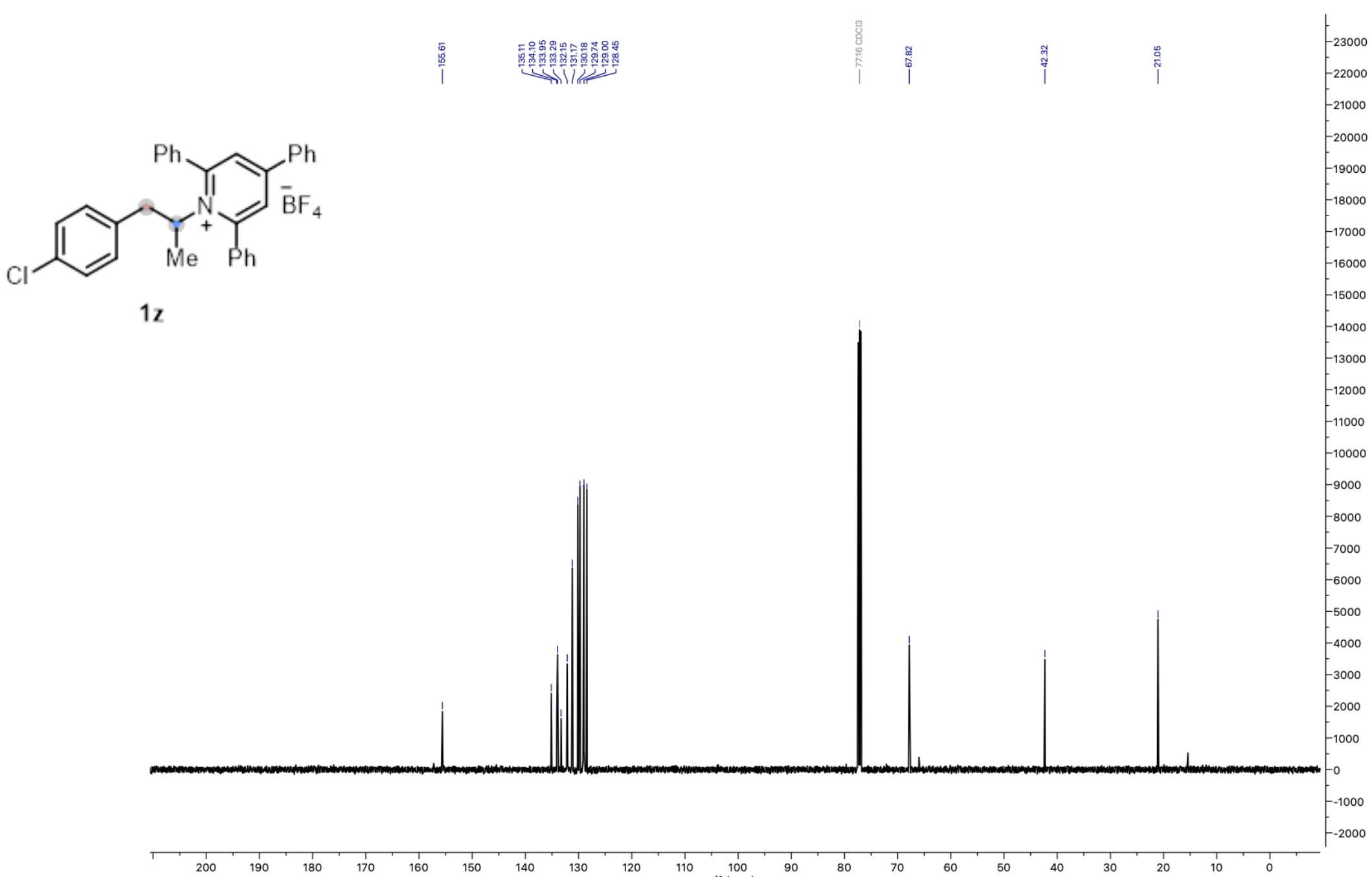

^{19}F NMR (282 MHz, $CDCl_3$):

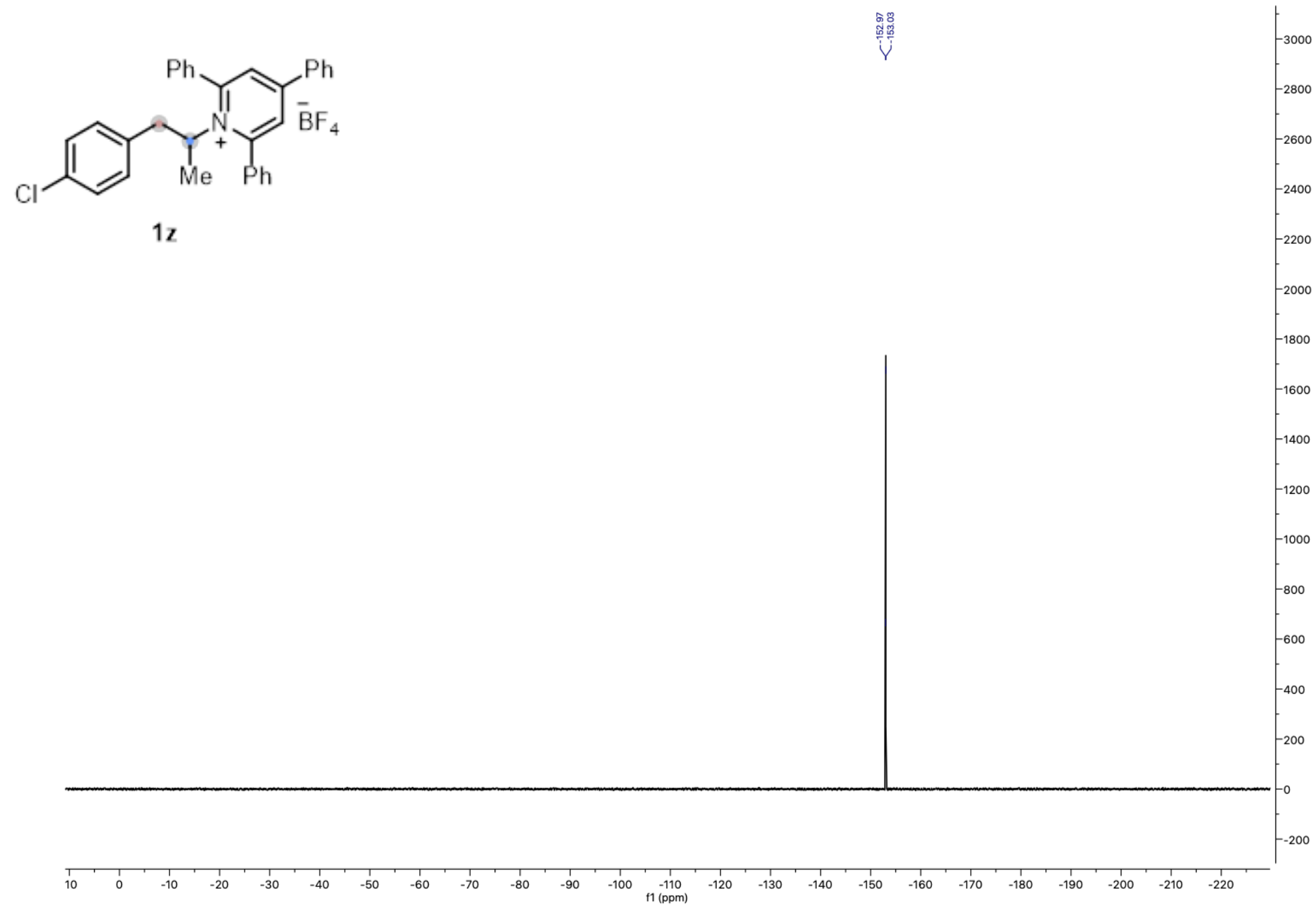

^{11}B NMR (96 MHz, $CDCl_3$):

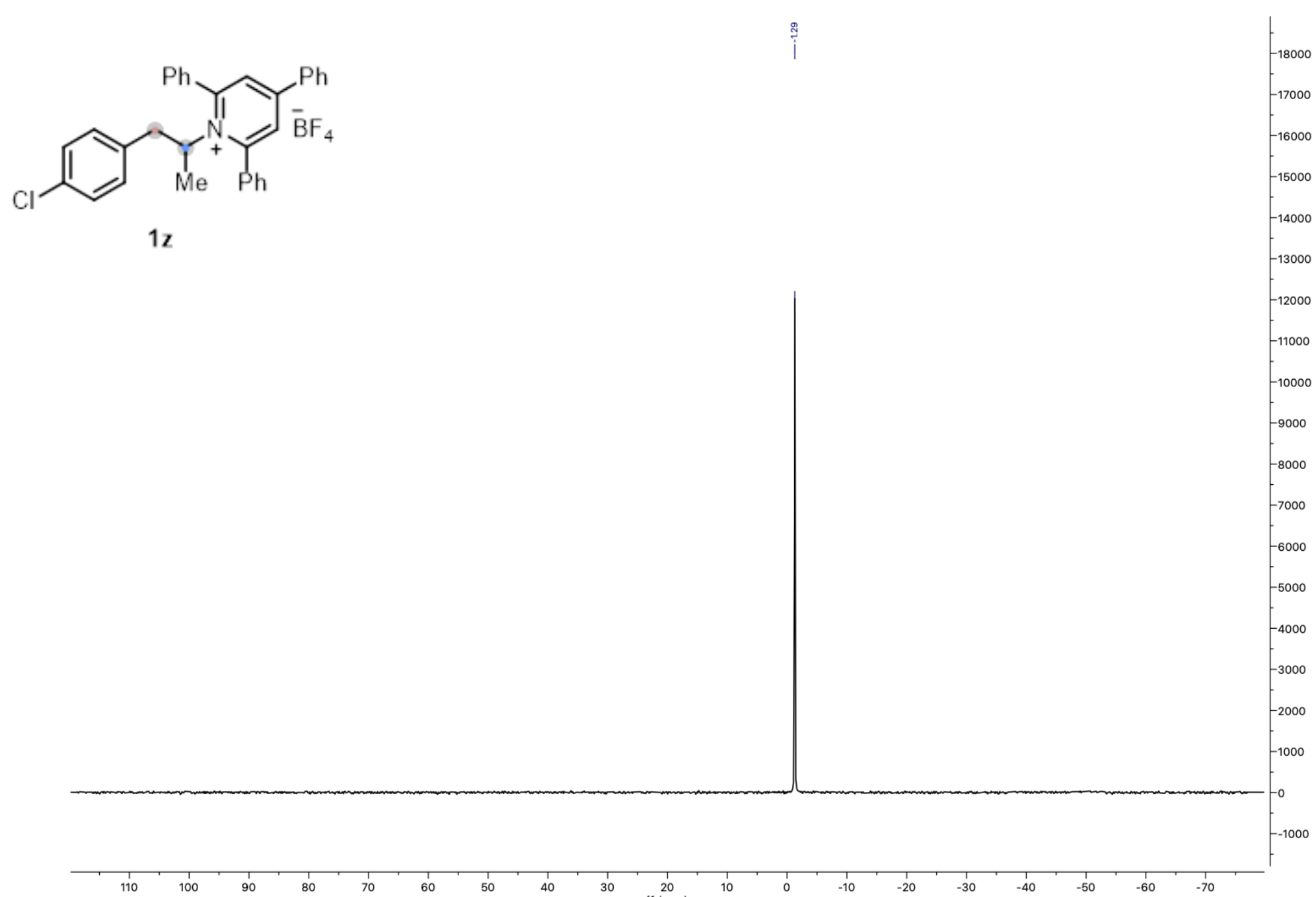

1-(1-(4-Fluorophenyl)propan-2-yl)-2,4,6-triphenylpyridin-1-ium tetrafluoroborate **1z**:

^{1}H NMR (500 MHz, $CDCl_3$):

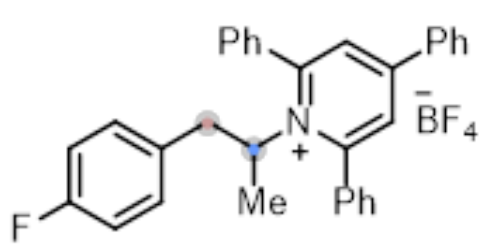

1aa

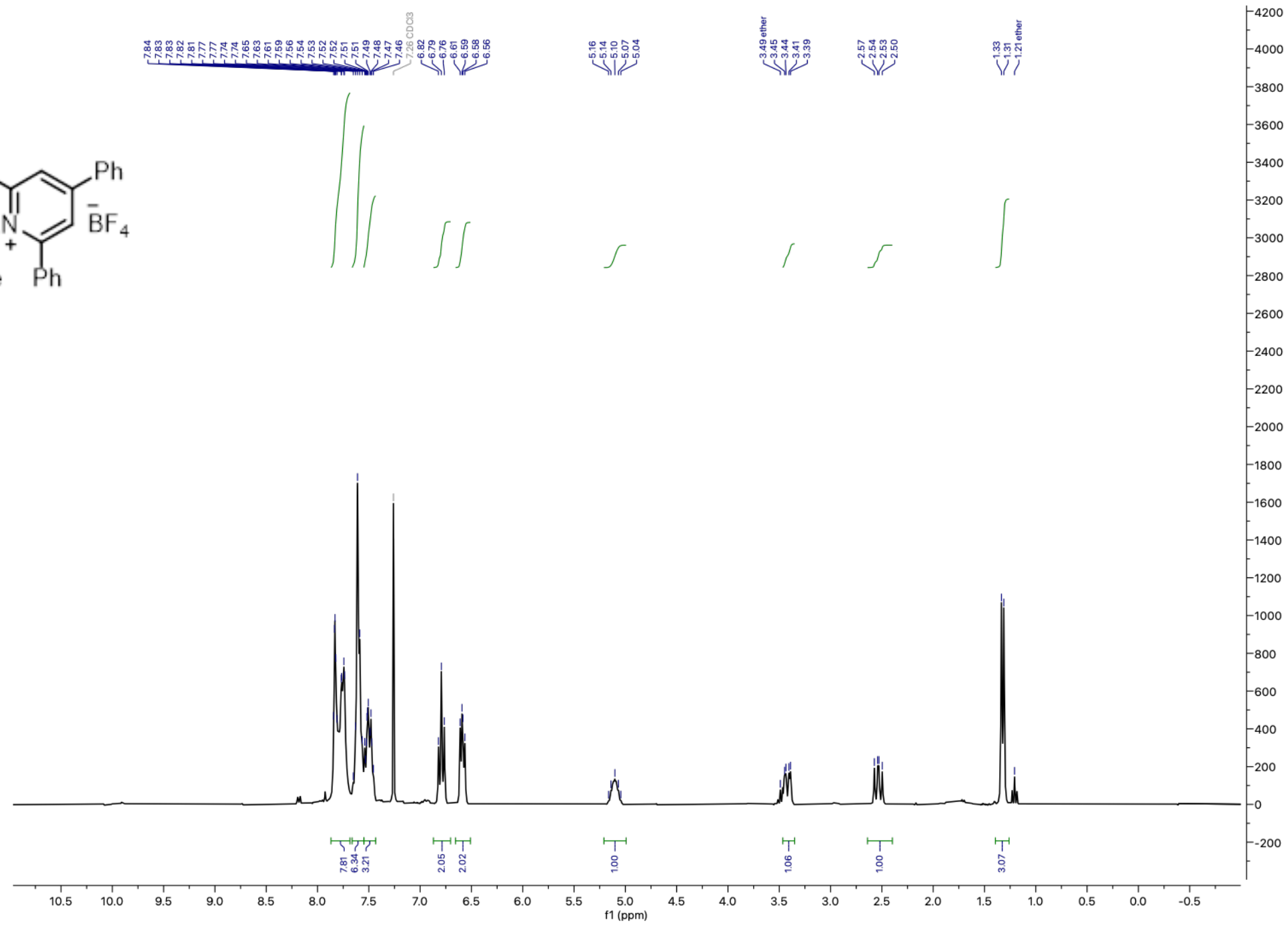

^{13}C NMR (126 MHz, $CDCl_3$):

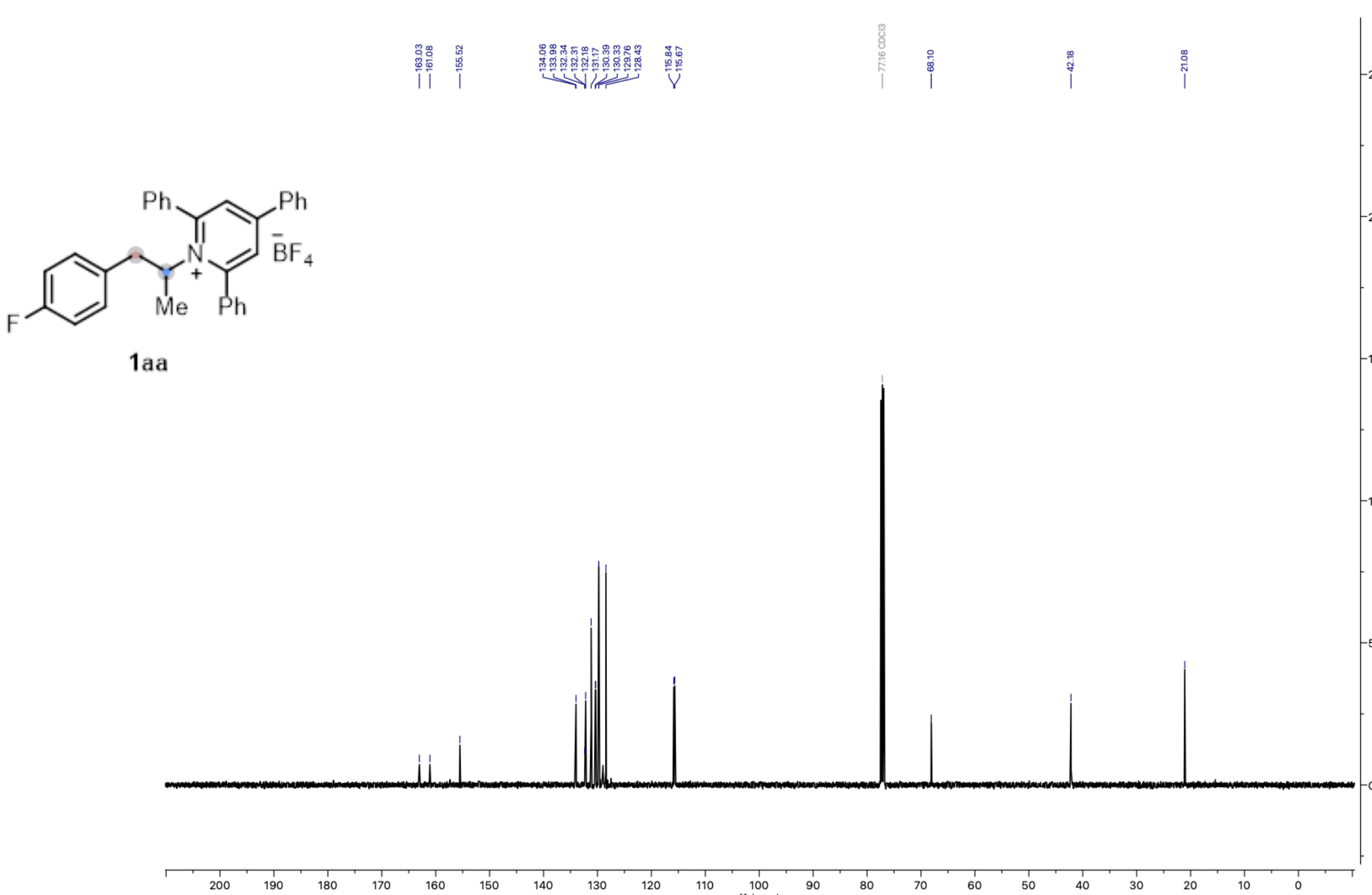

^{19}F NMR (282 MHz, $CDCl_3$):

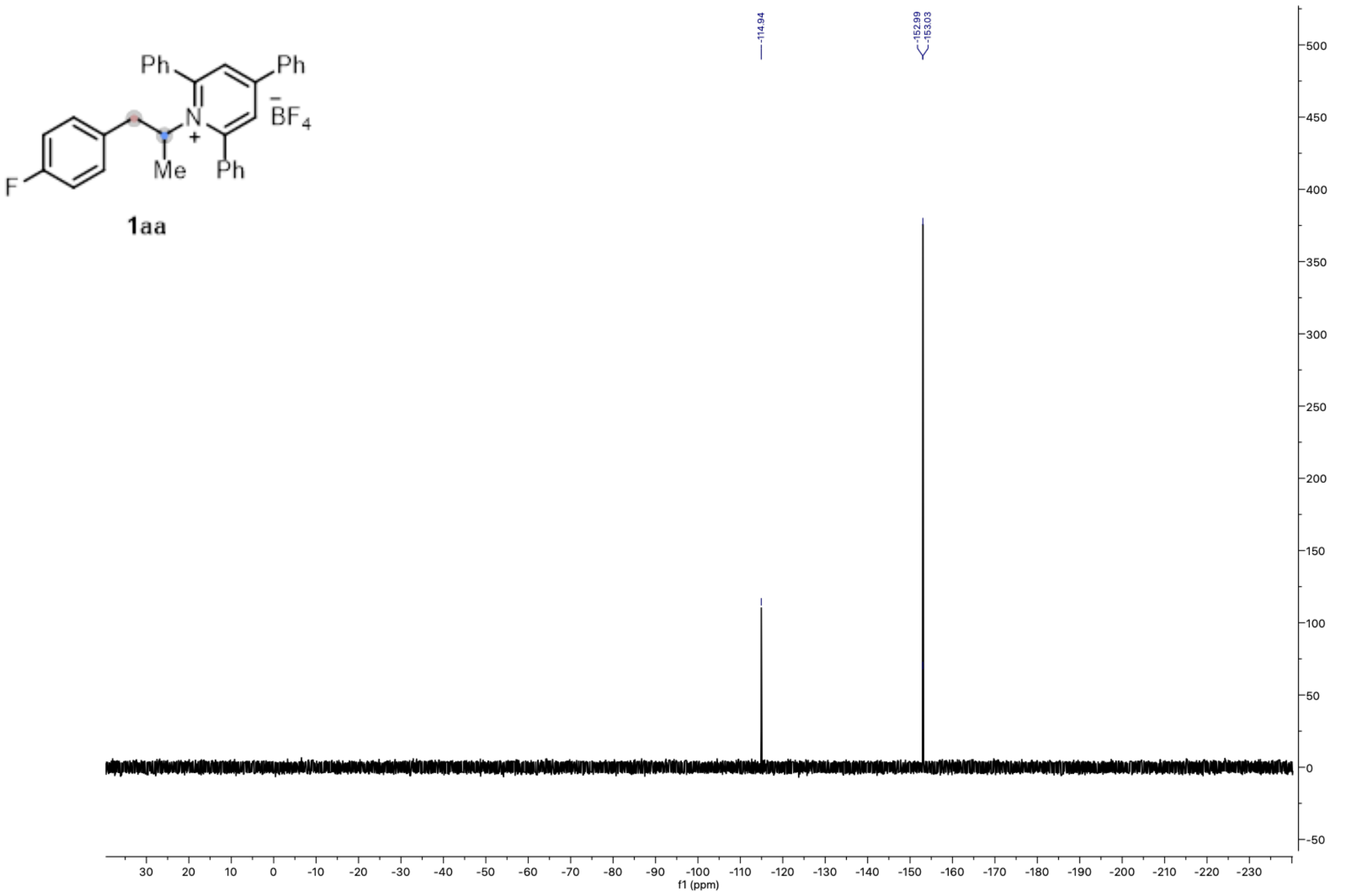

^{11}B NMR (96 MHz, $CDCl_3$):

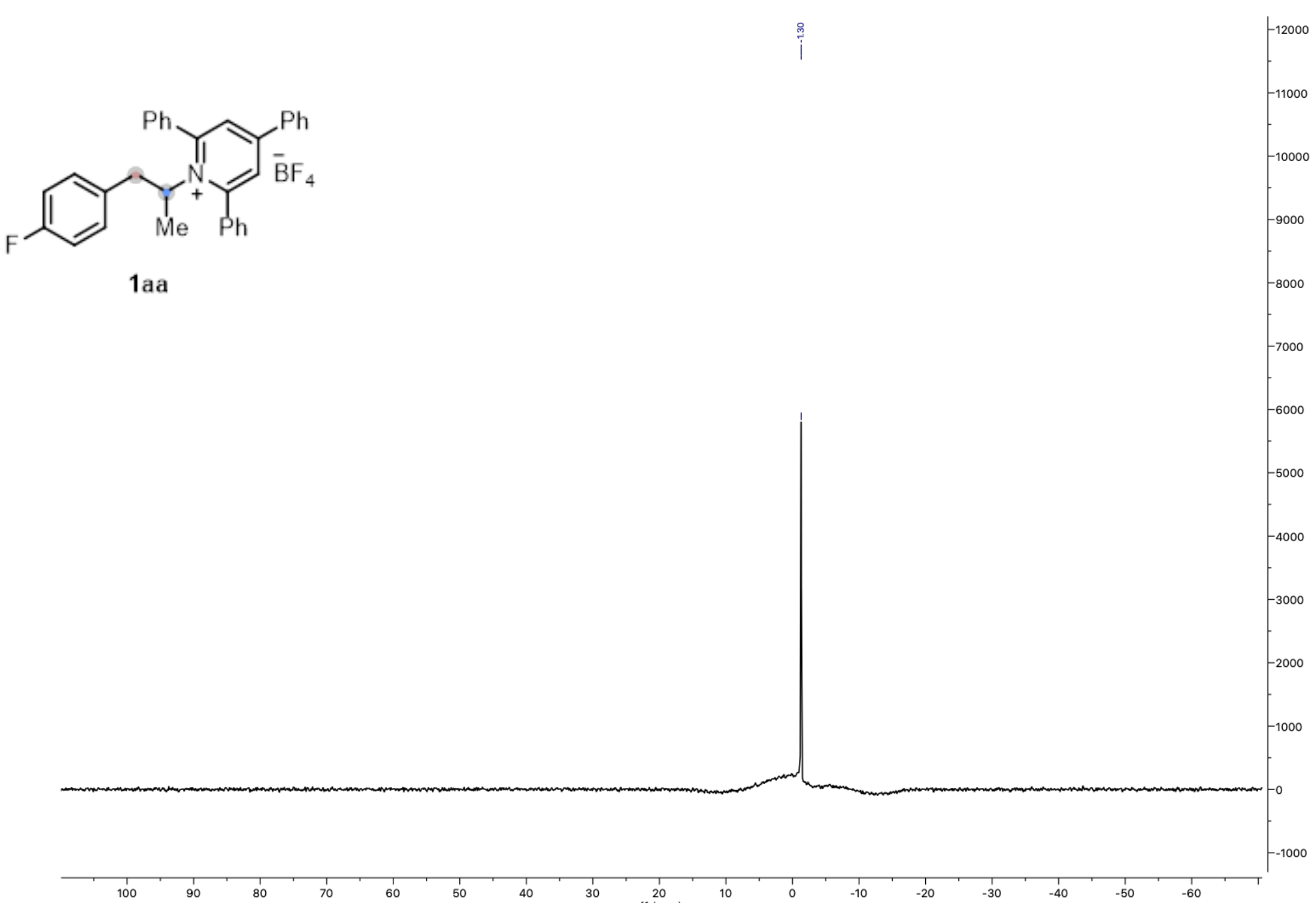

2,4,6-Triphenyl-1-(1-(4-(trifluoromethyl)phenyl)propan-2-yl)pyridin-1-ium tetrafluoroborate **1z**:

^{1}H NMR (500 MHz, $CDCl_3$):

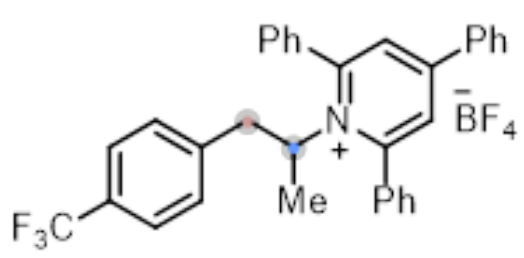

1ab

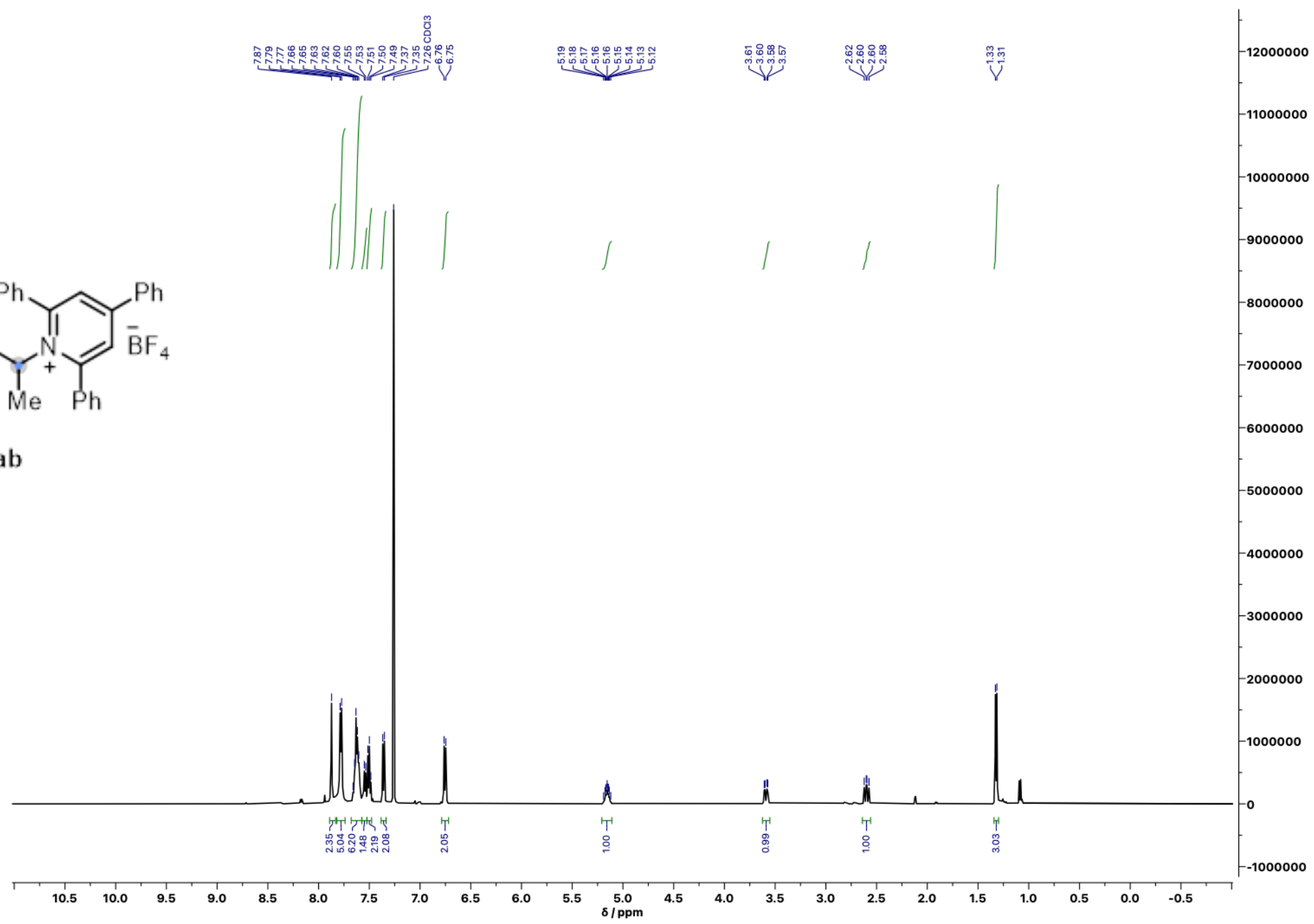

^{13}C NMR (126 MHz, $CDCl_3$):

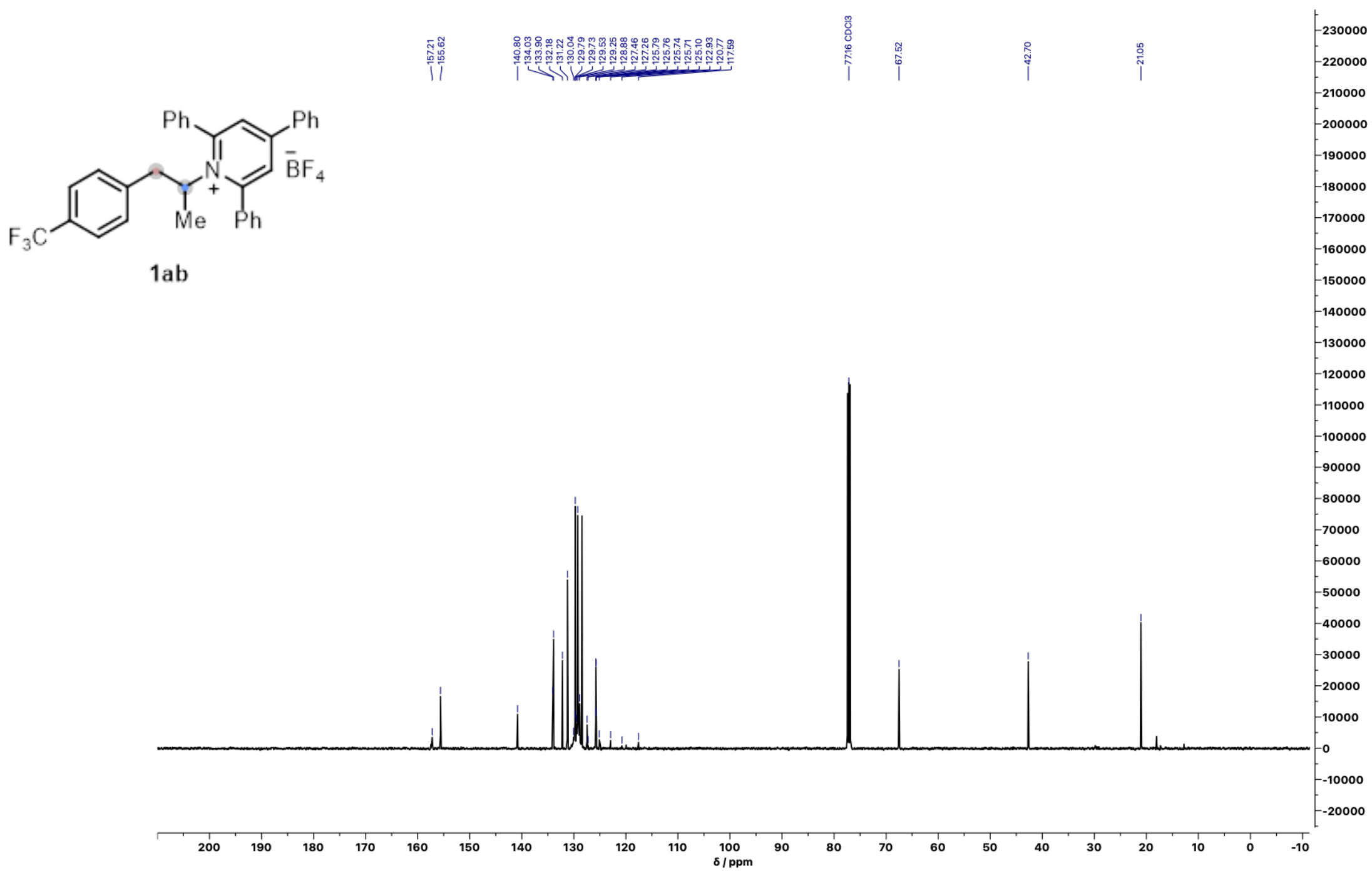

^{19}F NMR (282 MHz, $CDCl_3$):

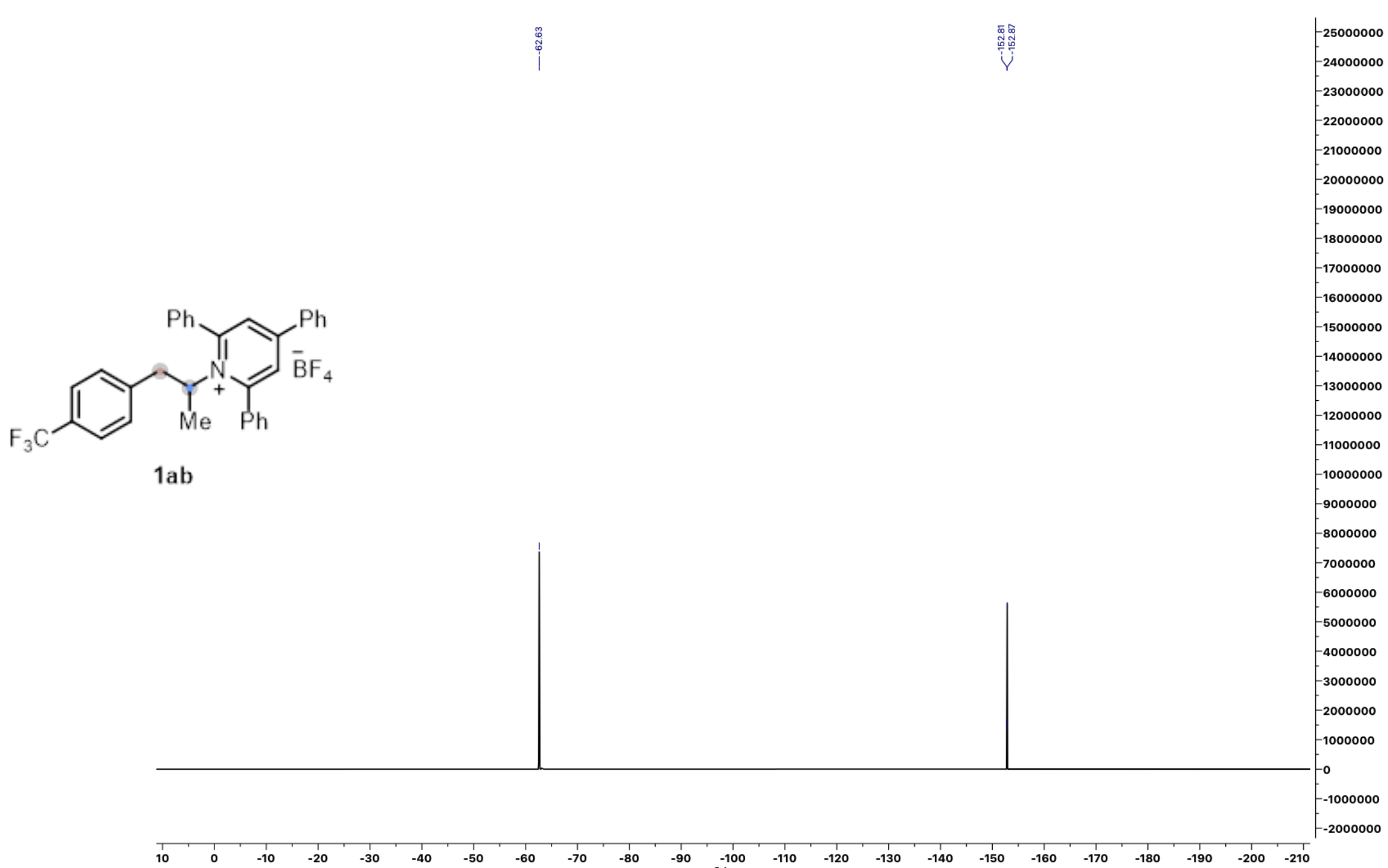

^{11}B NMR (96 MHz, $CDCl_3$):

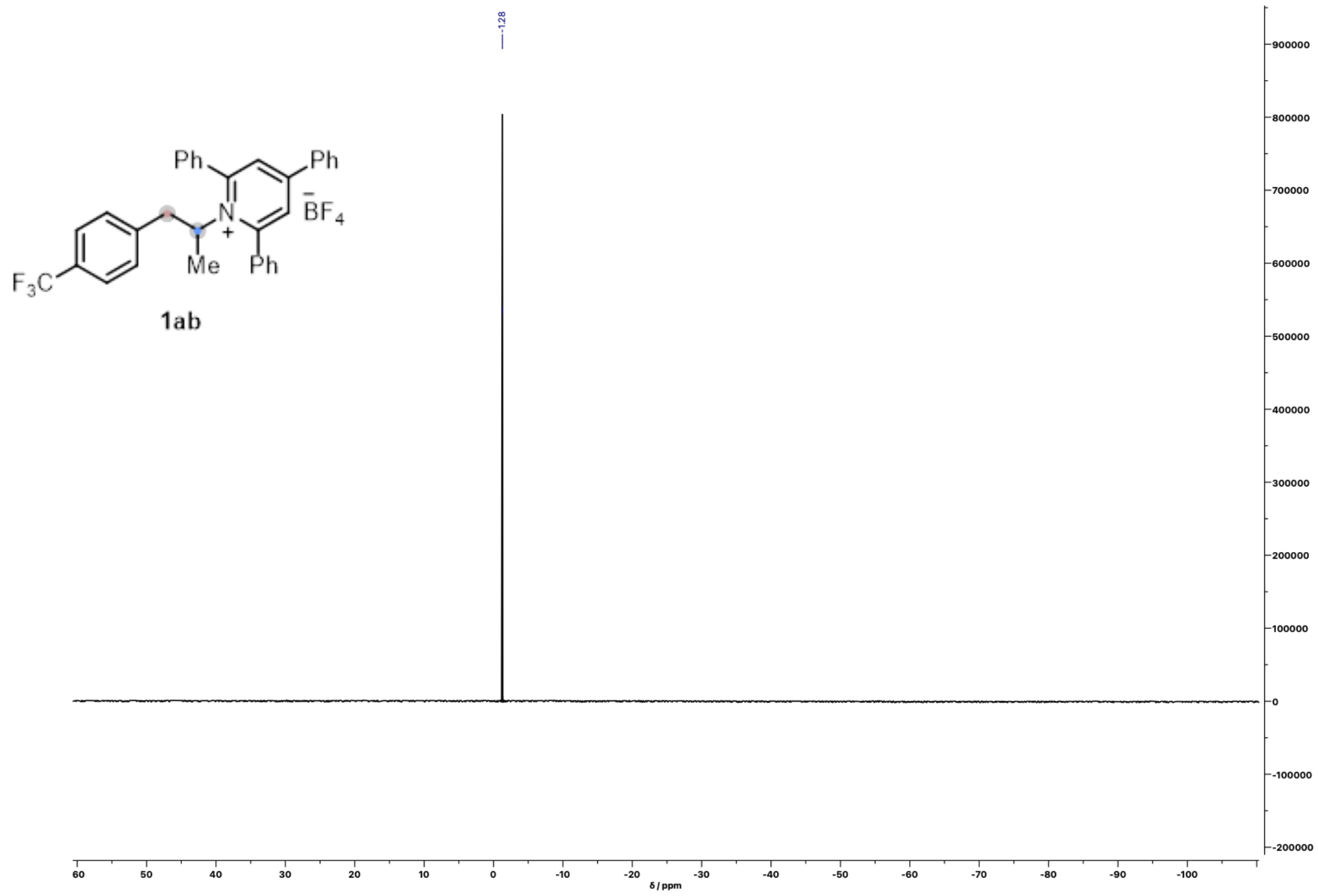

1-(1-(3-Methoxyphenyl)propan-2-yl)-2,4,6-triphenylpyridin-1-ium tetrafluoroborate **1ac**:

^{1}H NMR (500 MHz, $CDCl_3$):

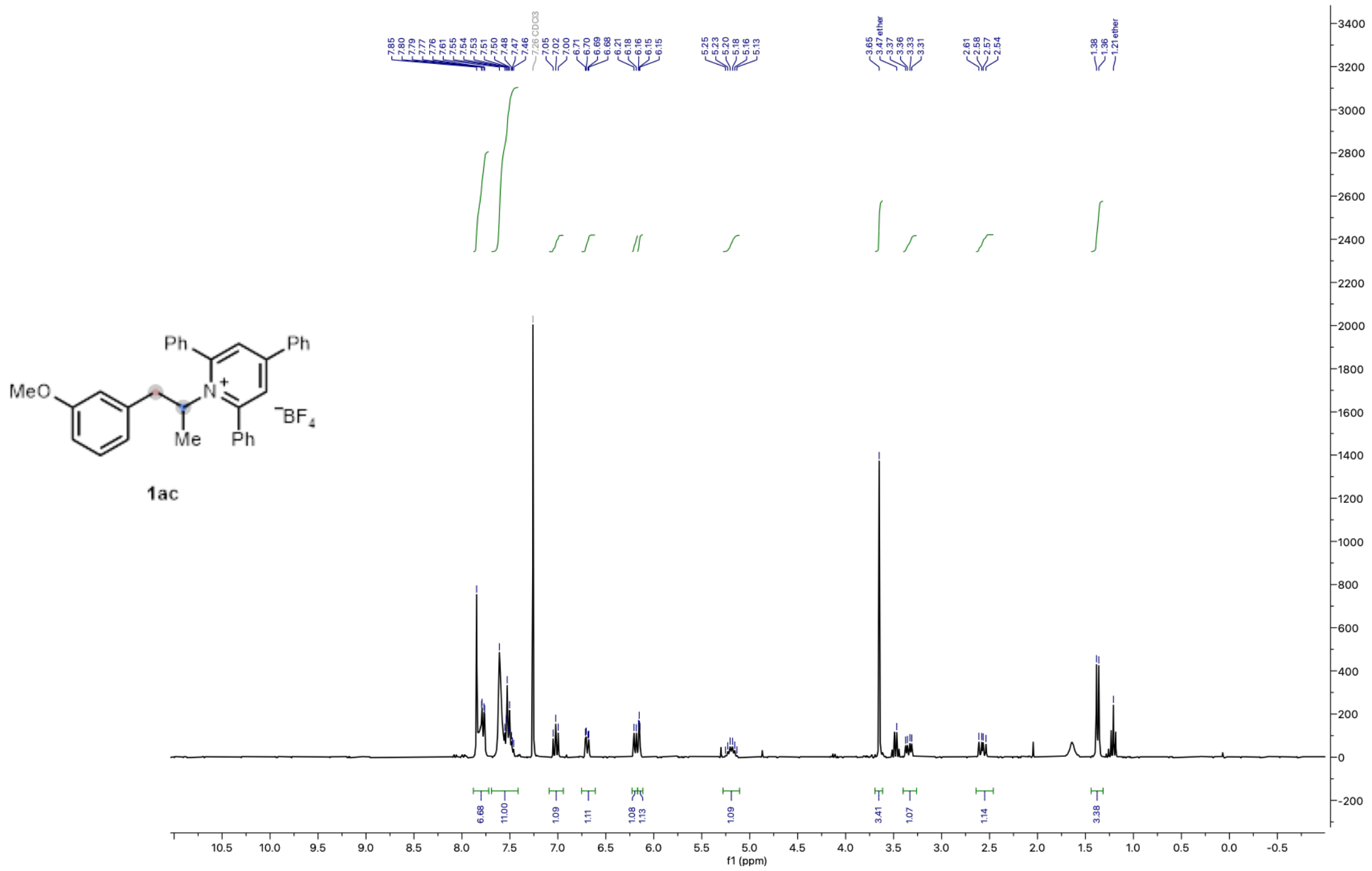

^{13}C NMR (126 MHz, $CDCl_3$):

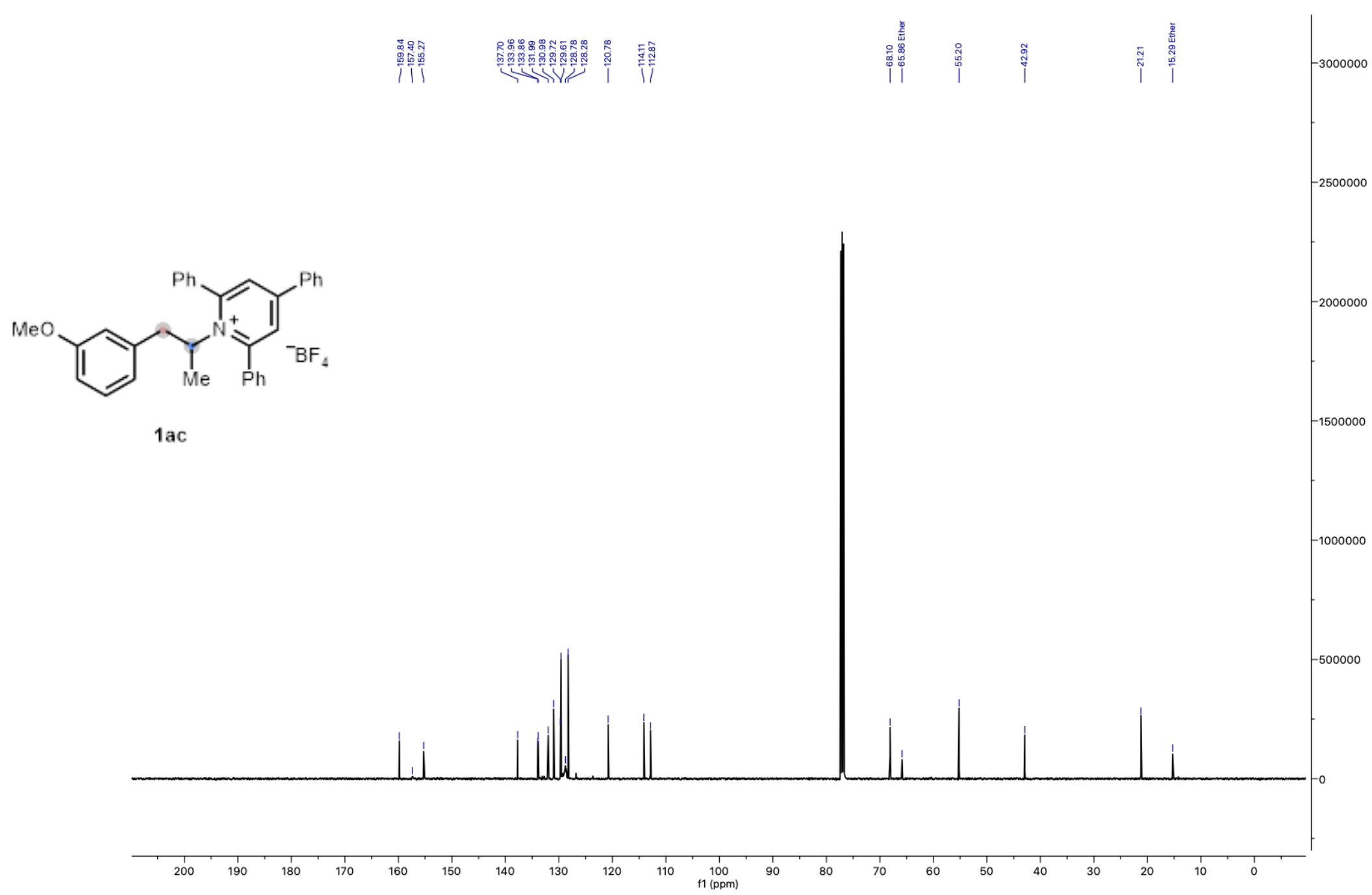

^{19}F NMR (282 MHz, $CDCl_3$):

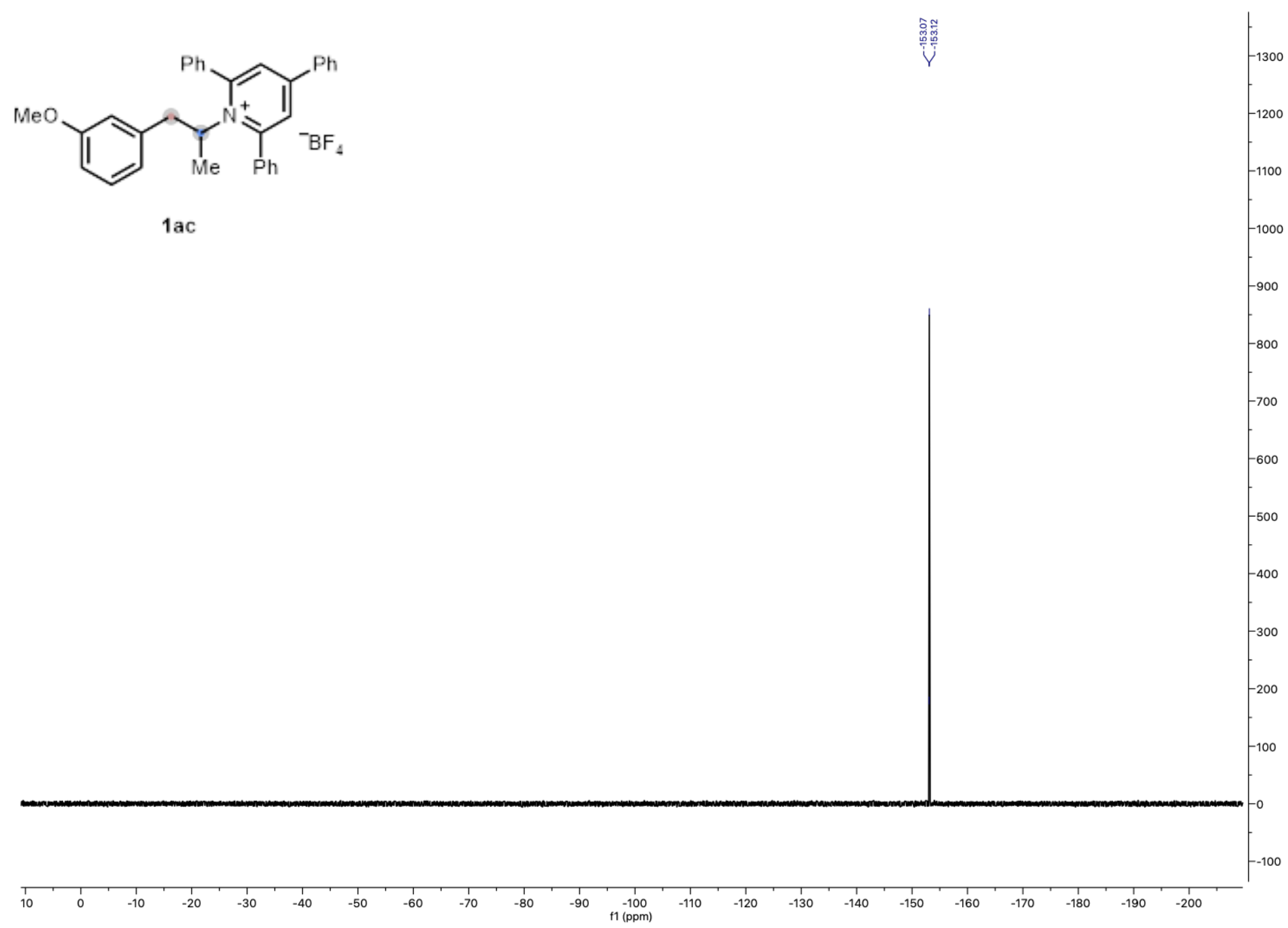

^{11}B NMR (96 MHz, $CDCl_3$):

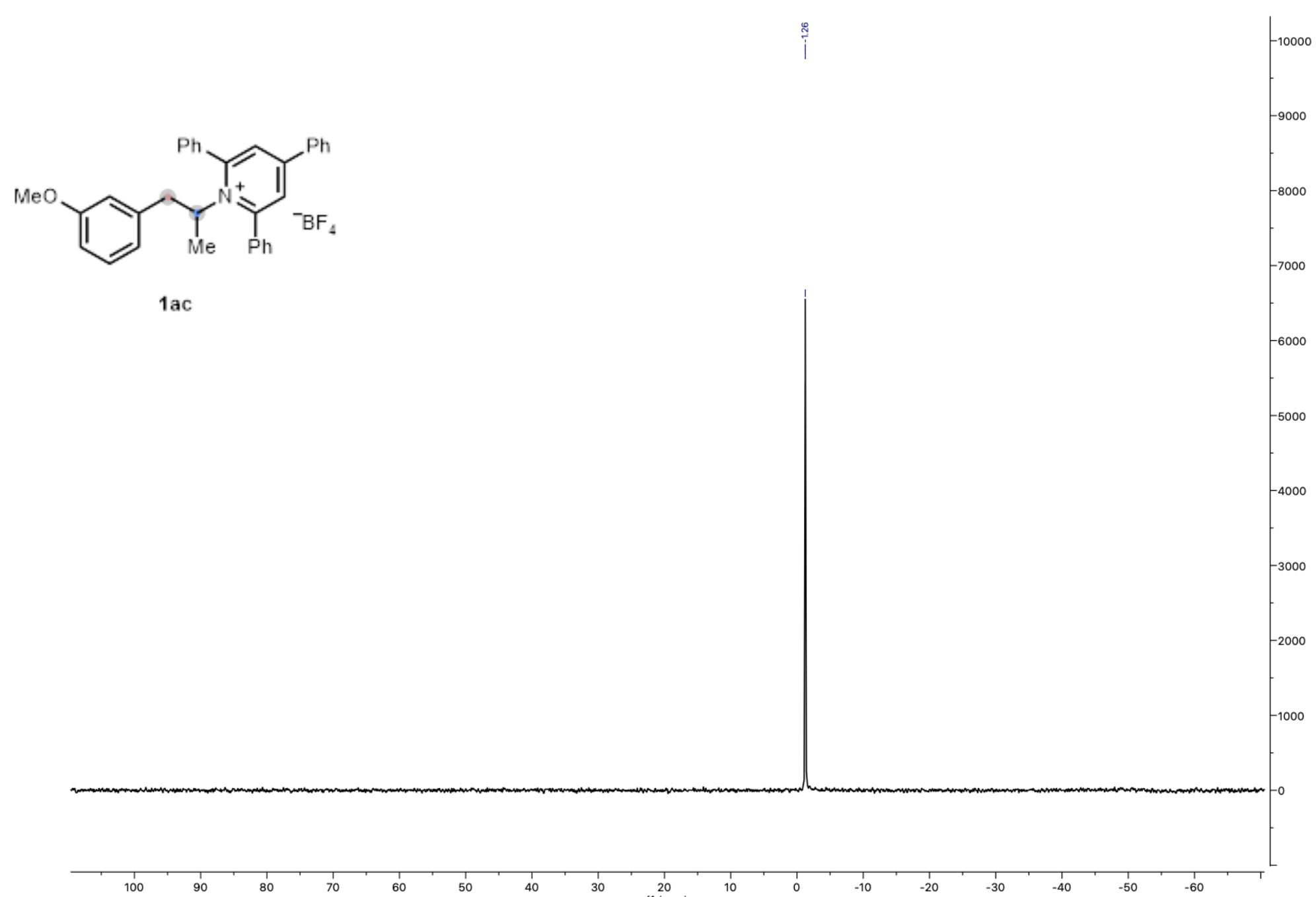

2,4,6-Triphenyl-1-(1-(3-(trifluoromethyl)phenyl)propan-2-yl)pyridin-1-ium tetrafluoroborate **1ad**:

^{1}H NMR (500 MHz, CDCl$_3$):

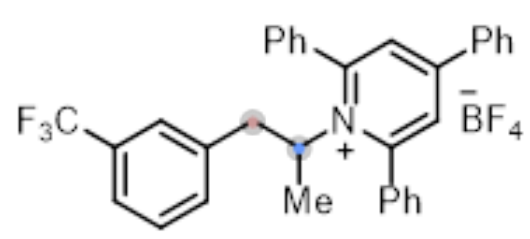

1ad

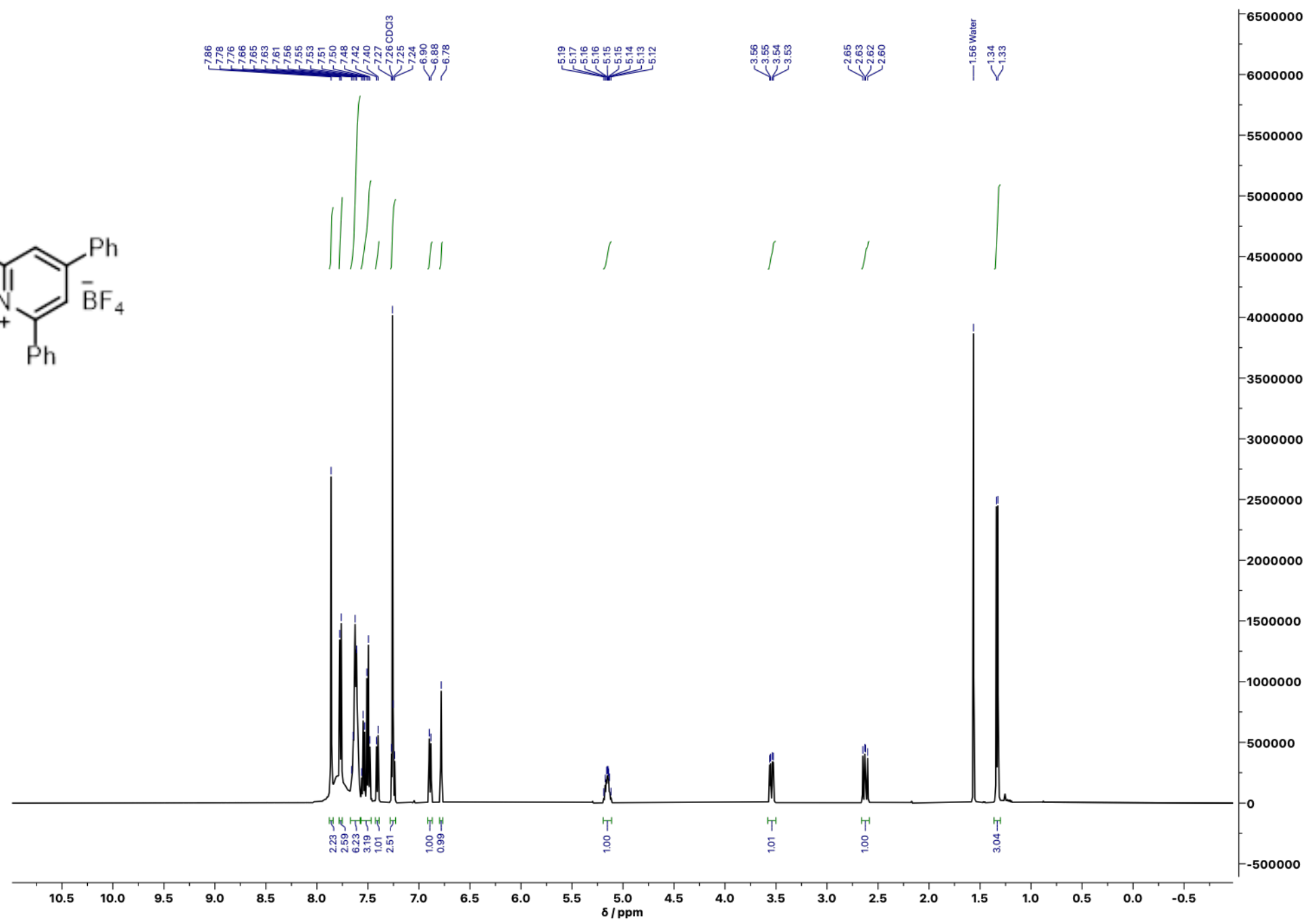

^{13}C NMR (126 MHz, $CDCl_3$):

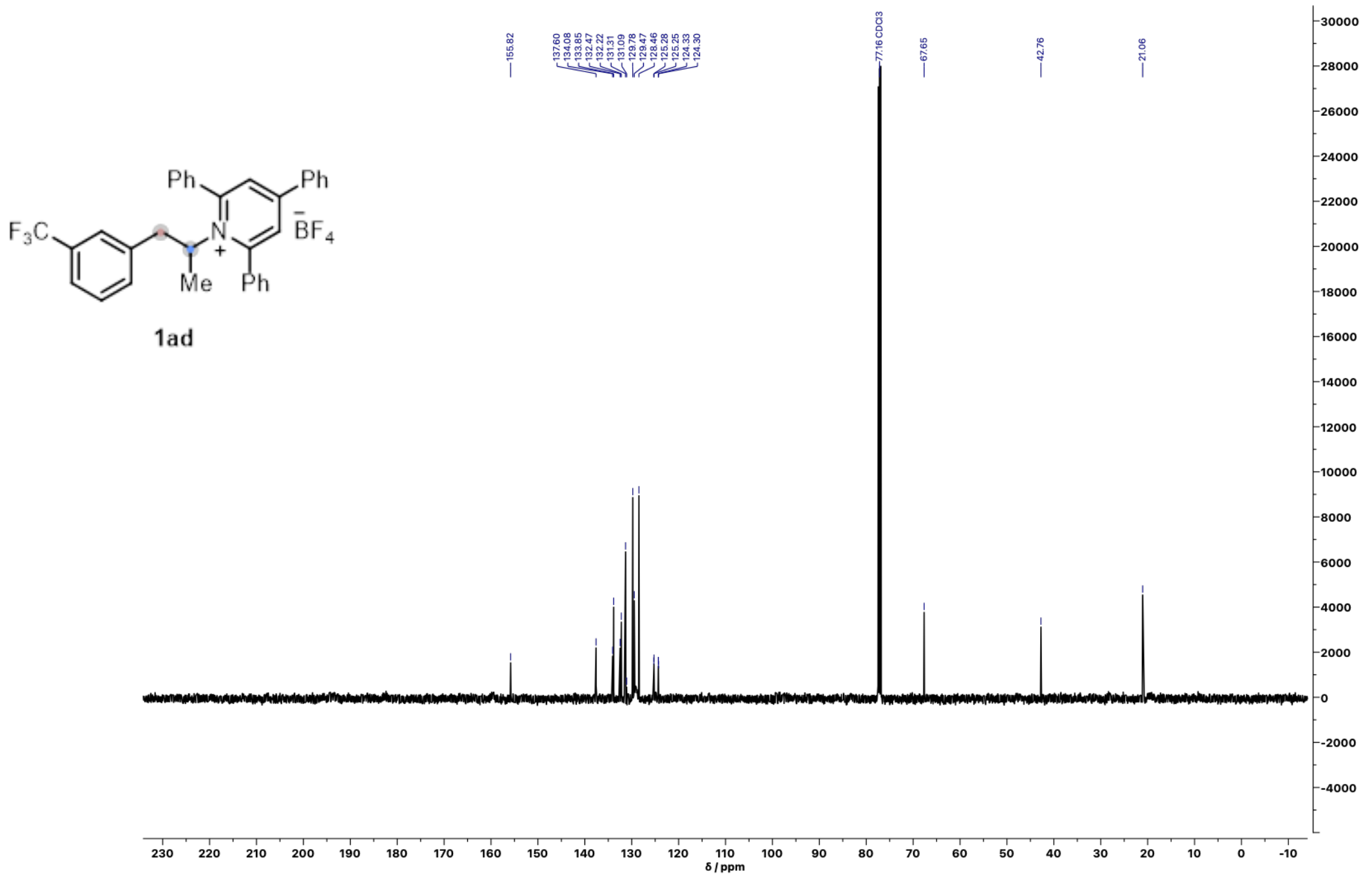

1ad

^{19}F NMR (282 MHz, $CDCl_3$):

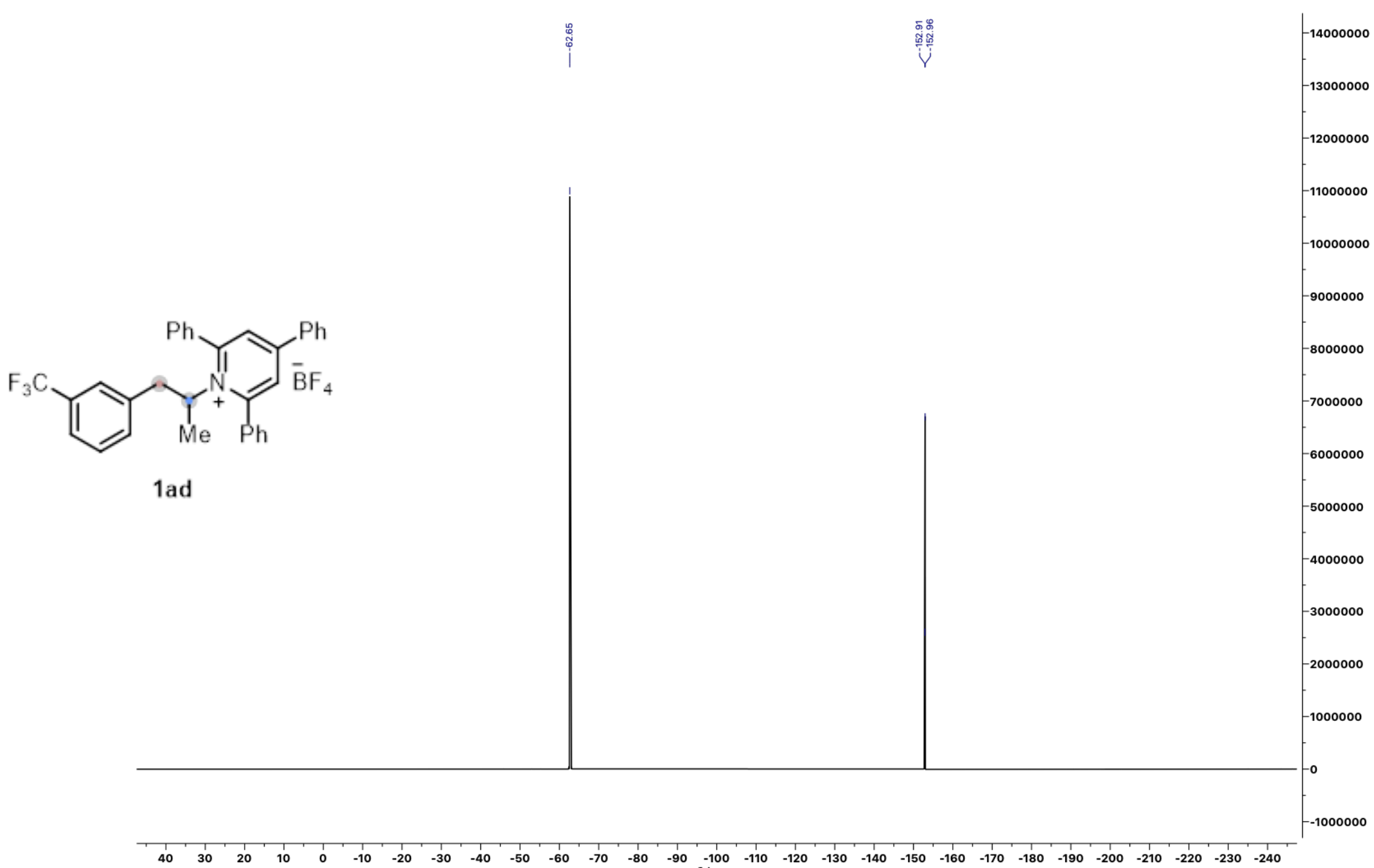

1ad

^{11}B NMR (96 MHz, $CDCl_3$):

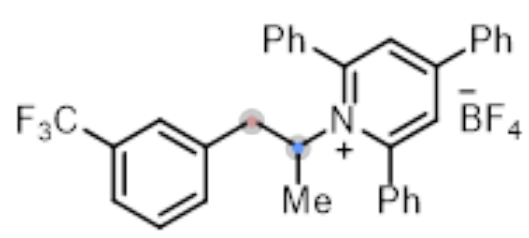

1ad

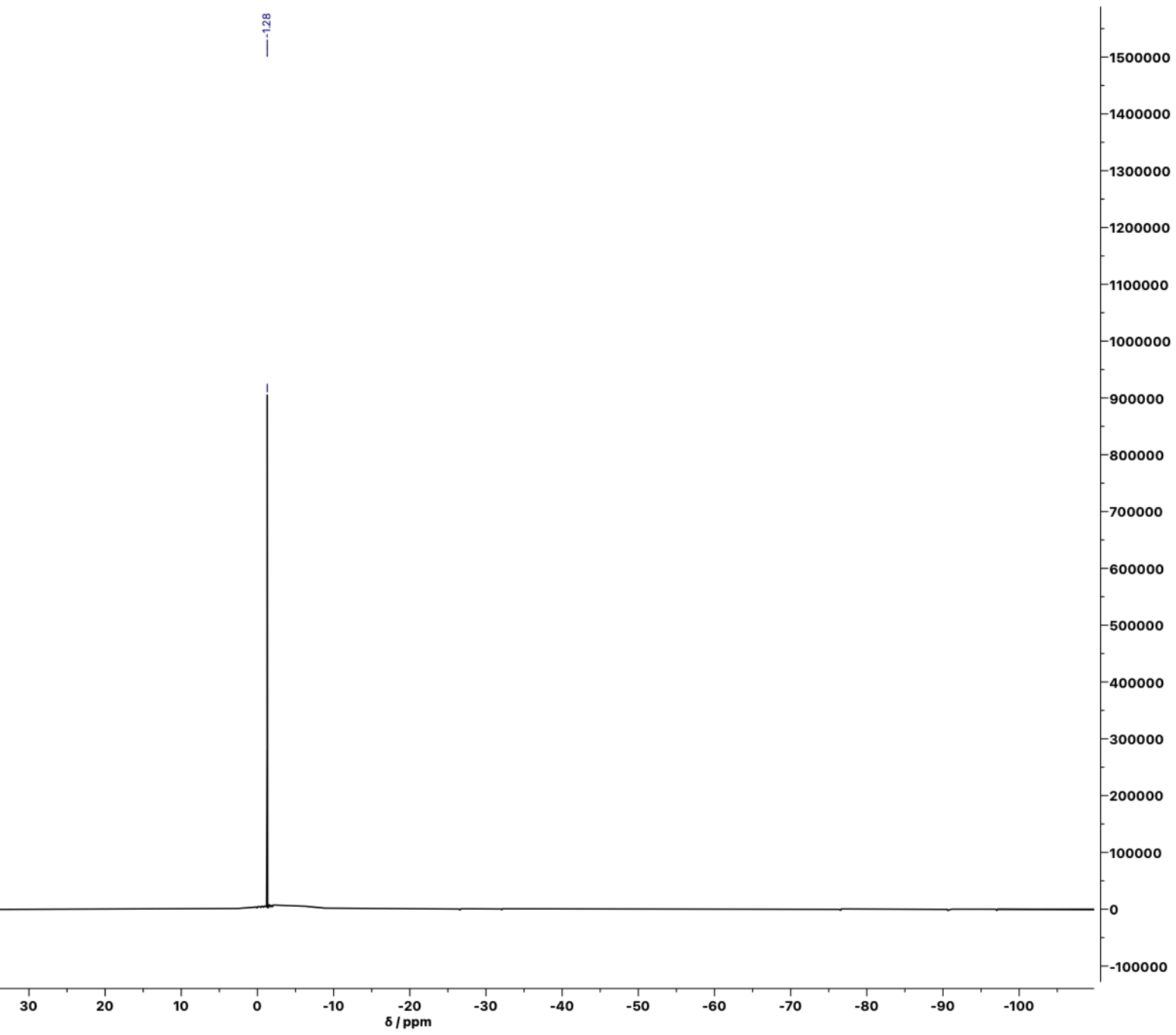

1-(1-(2-Methoxyphenyl)propan-2-yl)-2,4,6-triphenylpyridin-1-ium tetrafluoroborate **1ae**:

^{1}H NMR (500 MHz, CDCl$_3$):

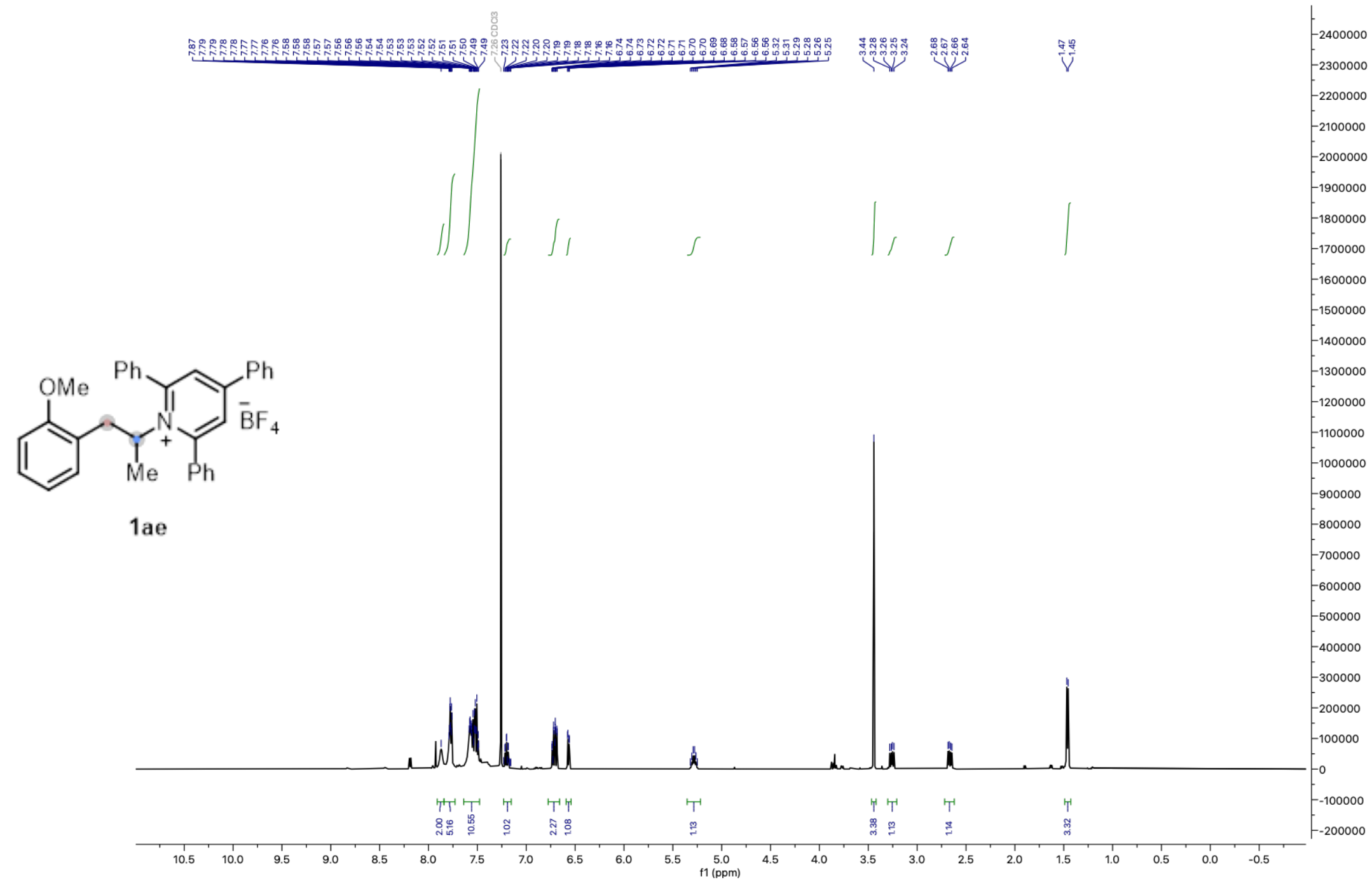

^{13}C NMR (126 MHz, CDCl$_3$):

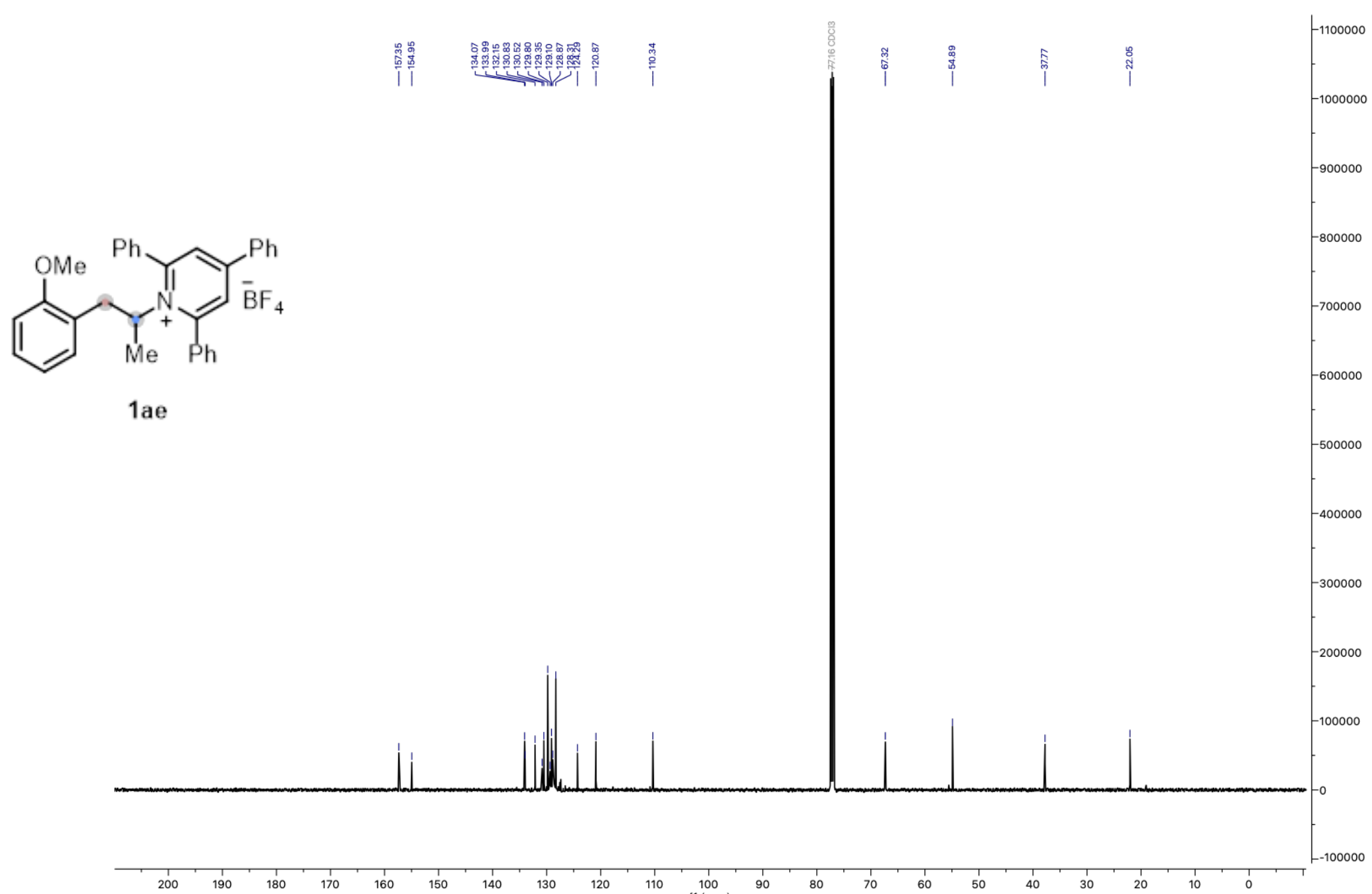

^{19}F NMR (282 MHz, $CDCl_3$):

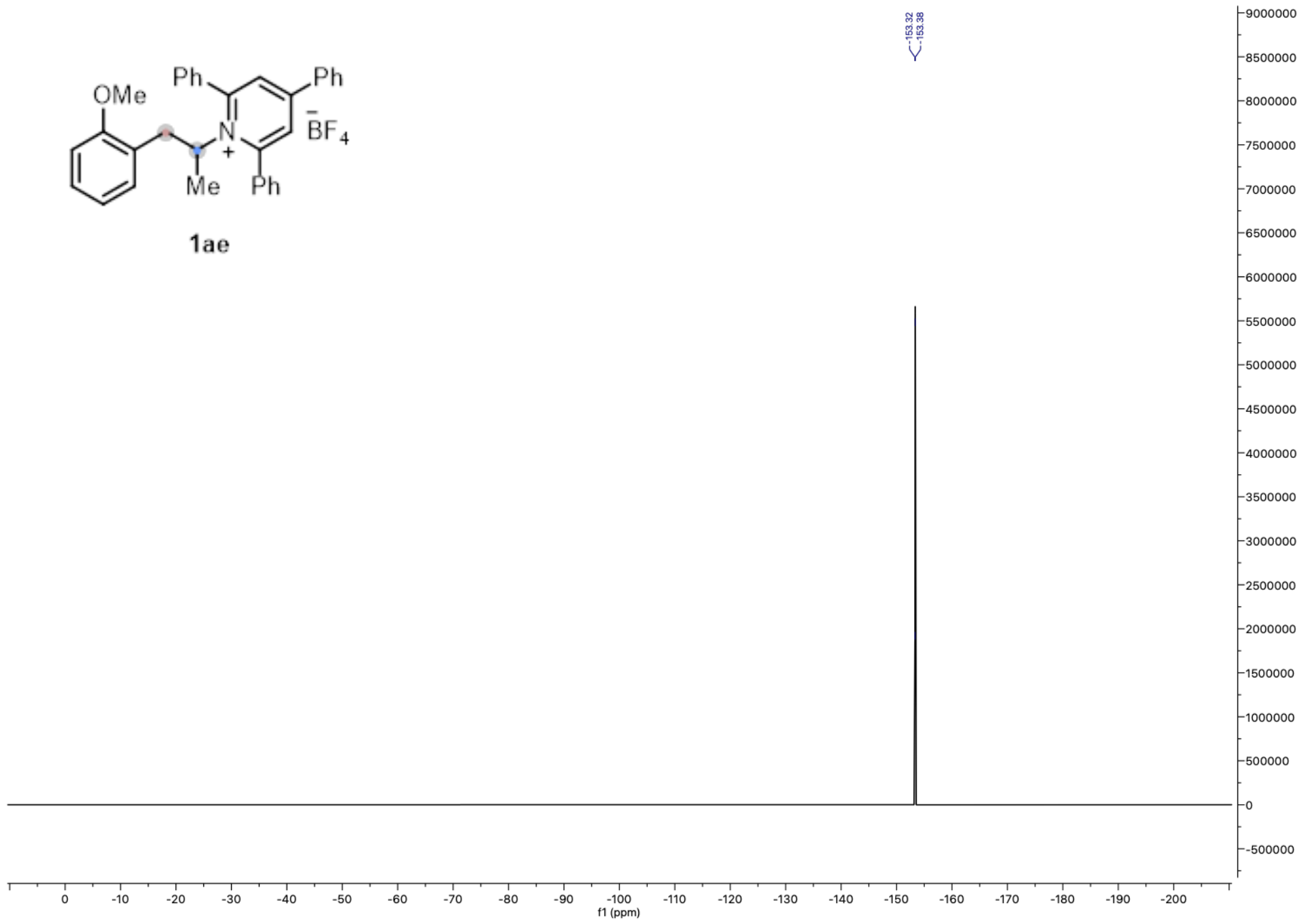

^{11}B NMR (96 MHz, $CDCl_3$):

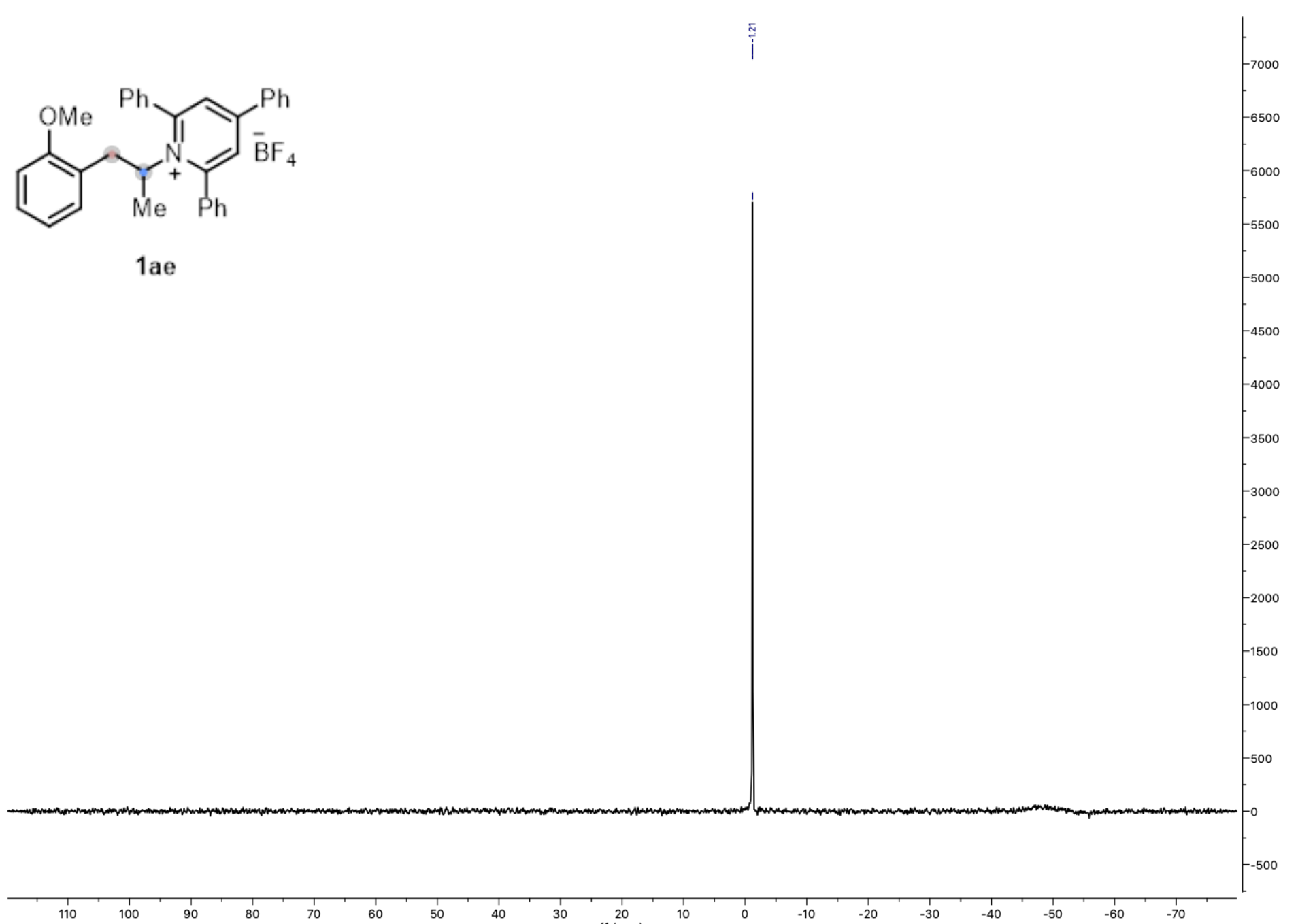

2,4,6-Triphenyl-1-(1,2,3,4-tetrahydronaphthalen-2-yl)pyridin-1-ium tetrafluoroborate

1ag:

^{1}H NMR (500 MHz, $CDCl_3$):

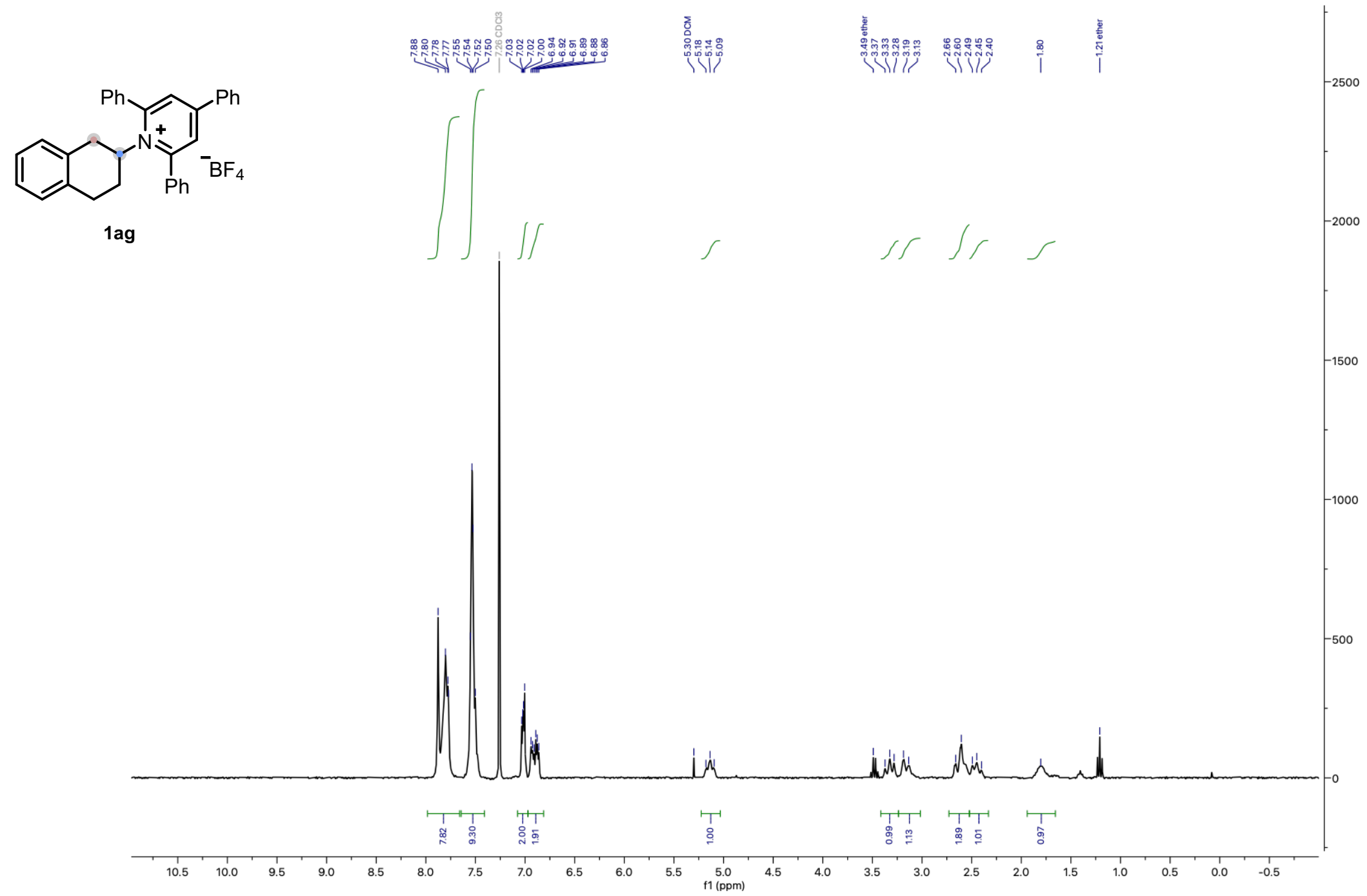

^{13}C NMR (126 MHz, $CDCl_3$):

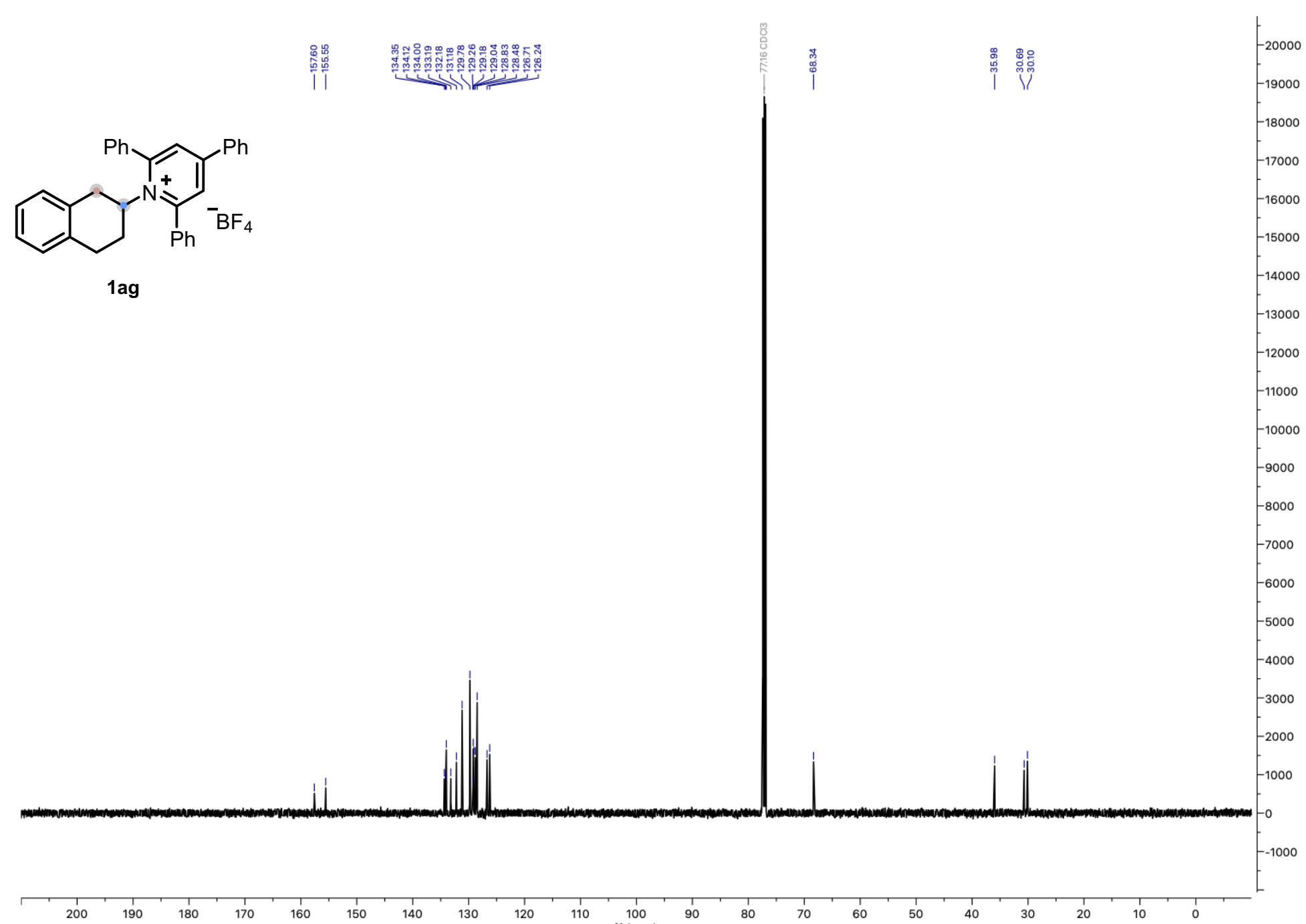

^{19}F NMR (282 MHz, $CDCl_3$):

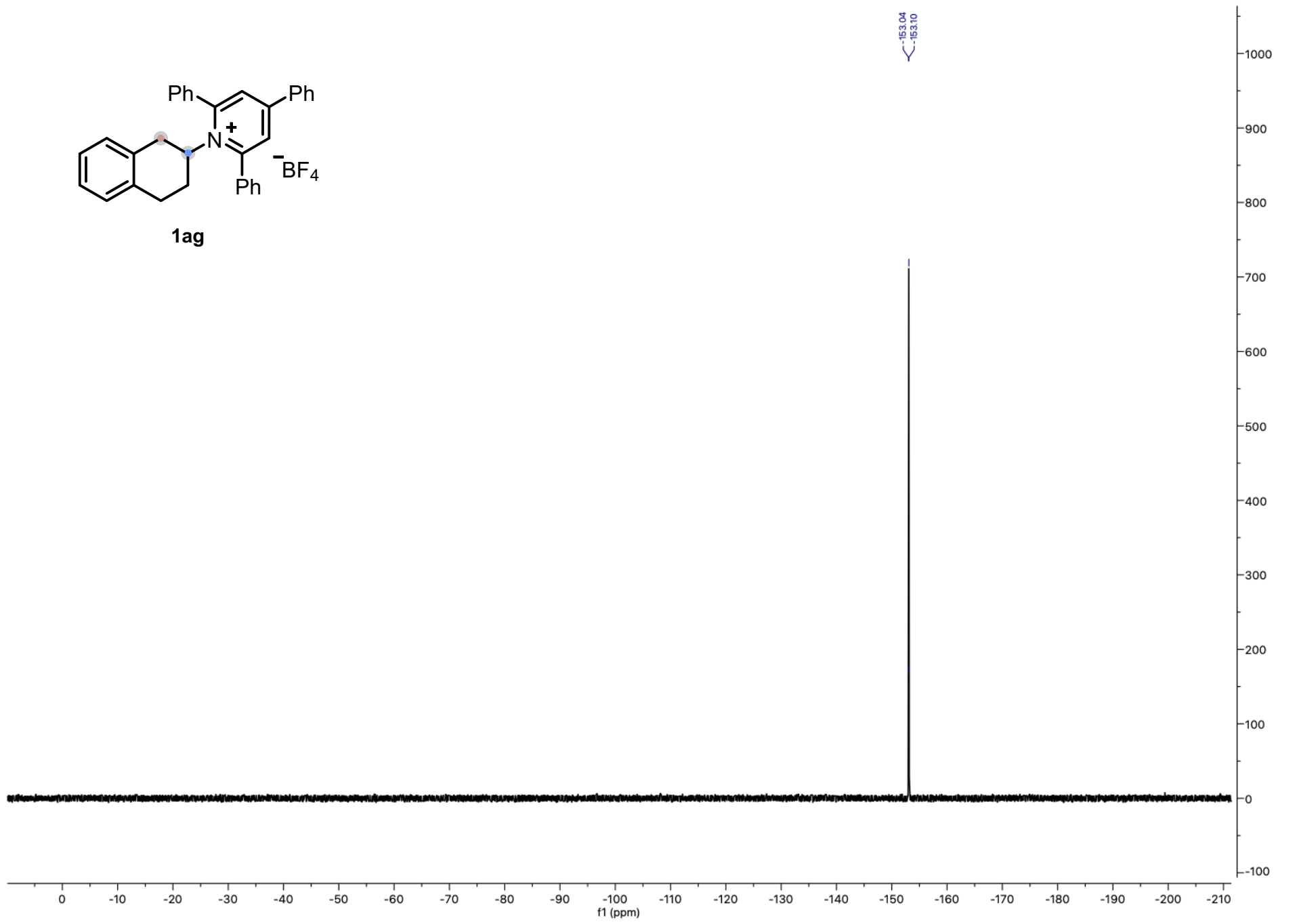

^{11}B NMR (96 MHz, $CDCl_3$):

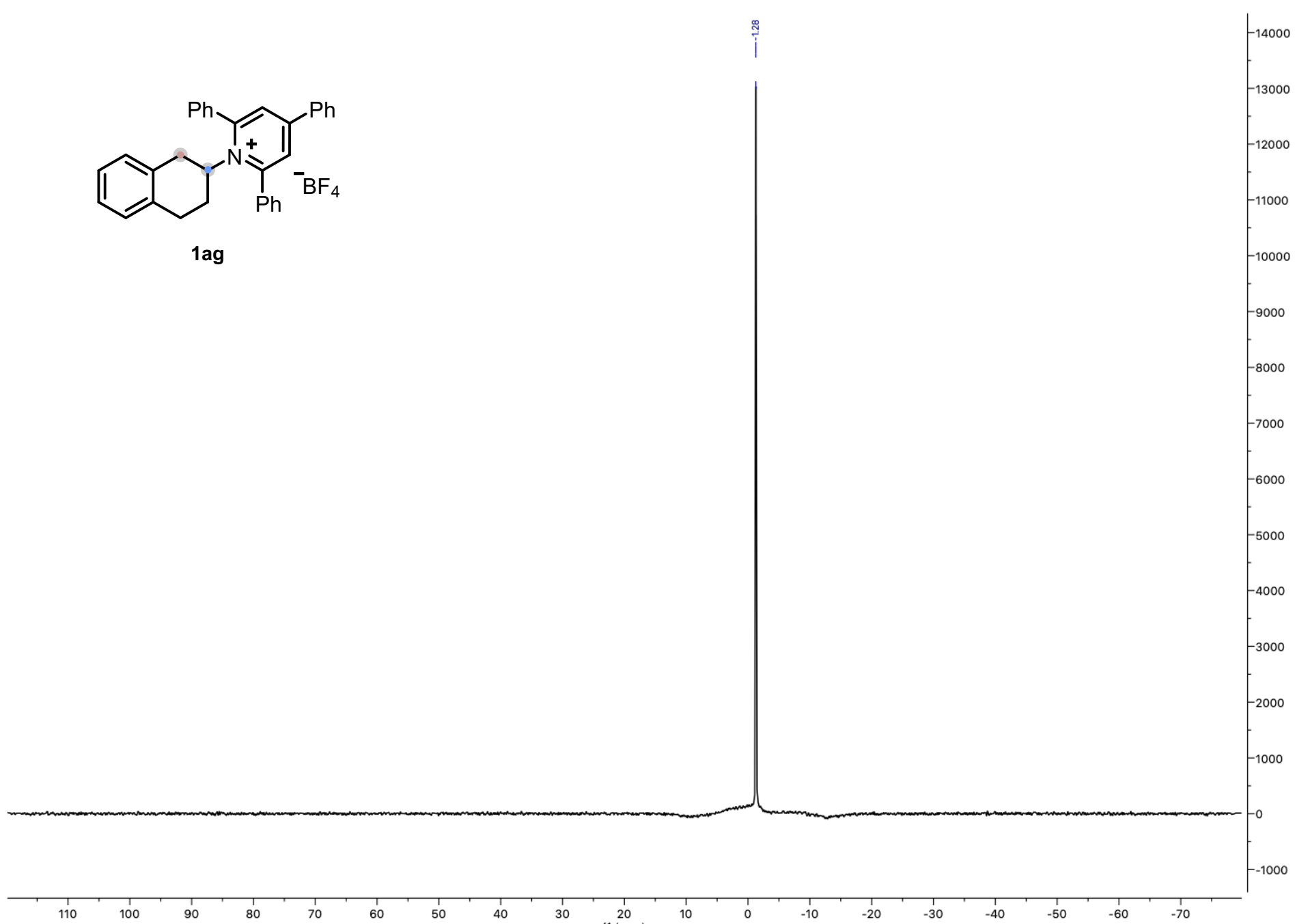

1-(5-Methoxy-1,2,3,4-tetrahydronaphthalen-2-yl)-2,4,6-triphenylpyridin-1-ium tetrafluoroborate **1ah**:

^{1}H NMR (500 MHz, $CDCl_3$):

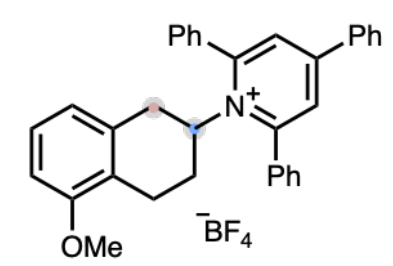

1ah

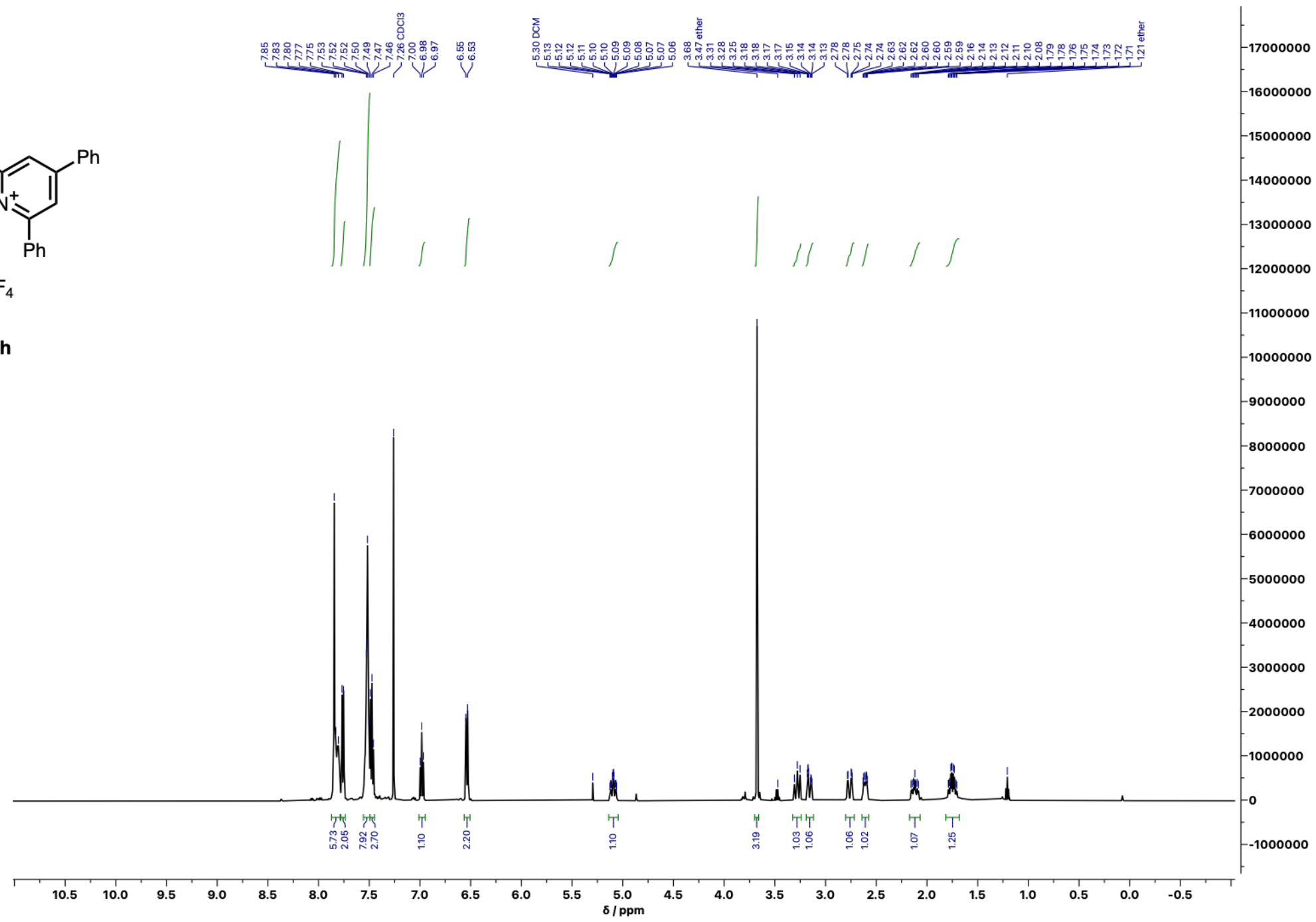

^{13}C NMR (126 MHz, $CDCl_3$):

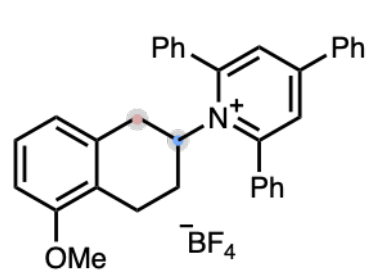

1ah

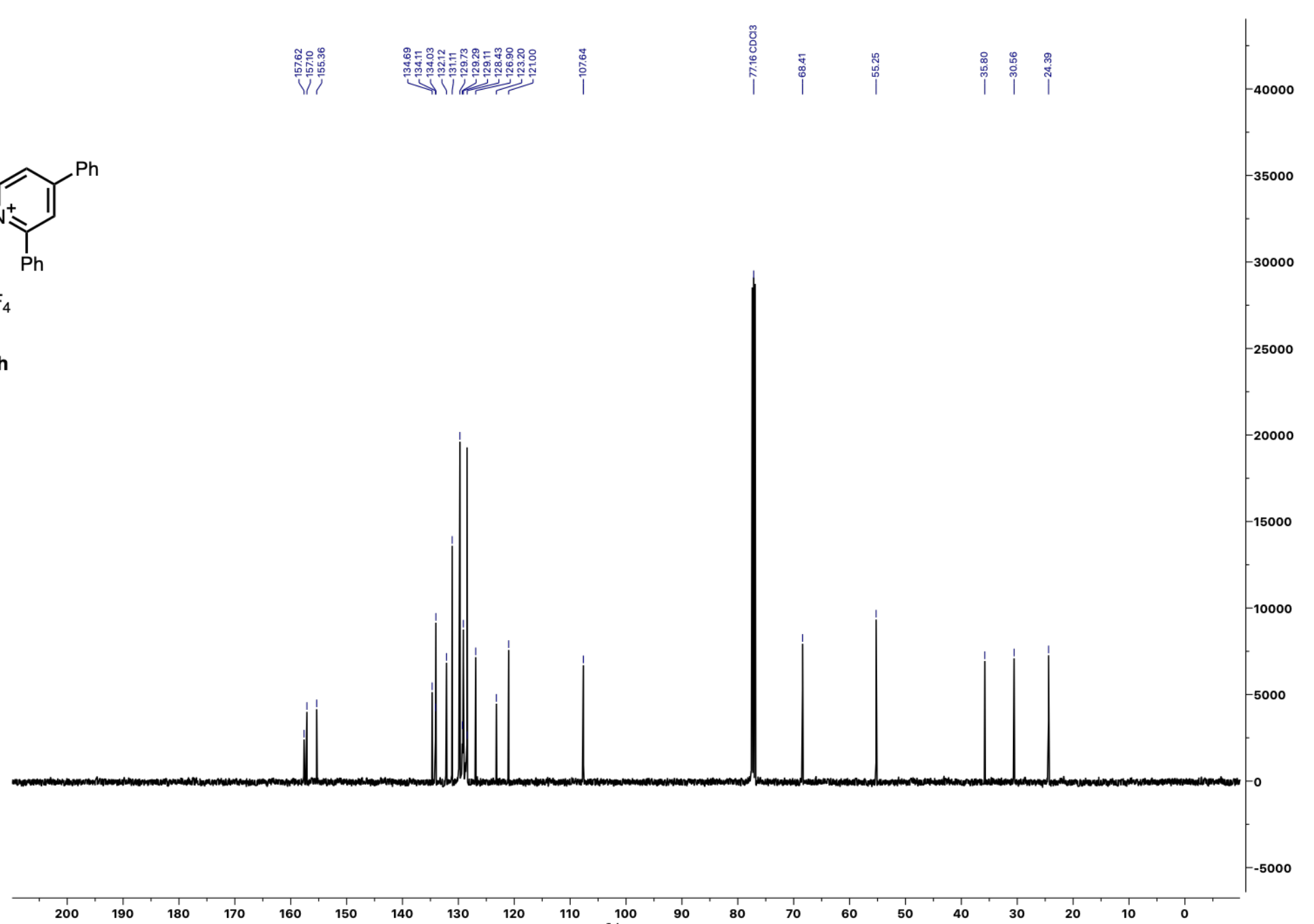

^{19}F NMR (282 MHz, $CDCl_3$):

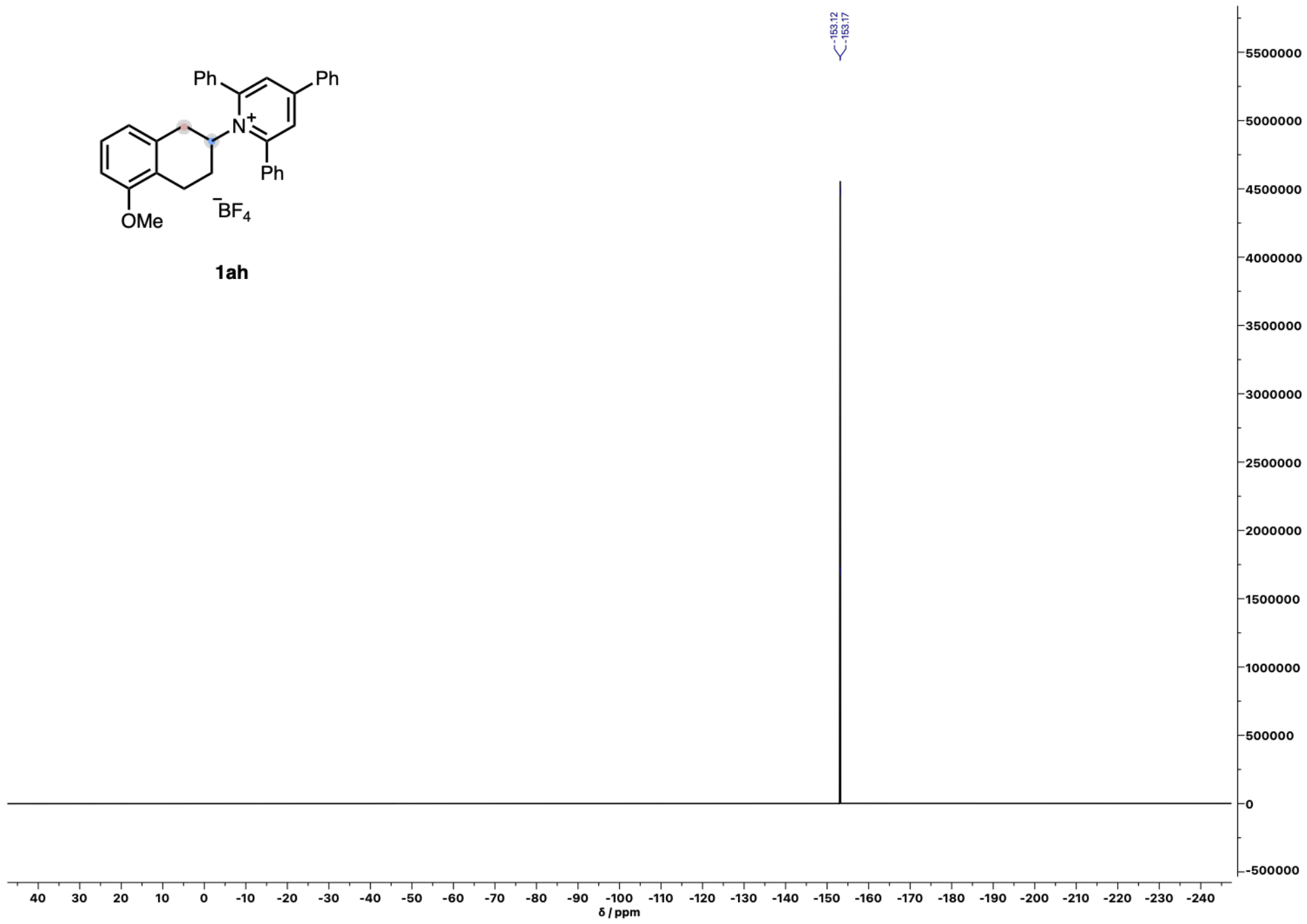

^{11}B NMR (96 MHz, $CDCl_3$):

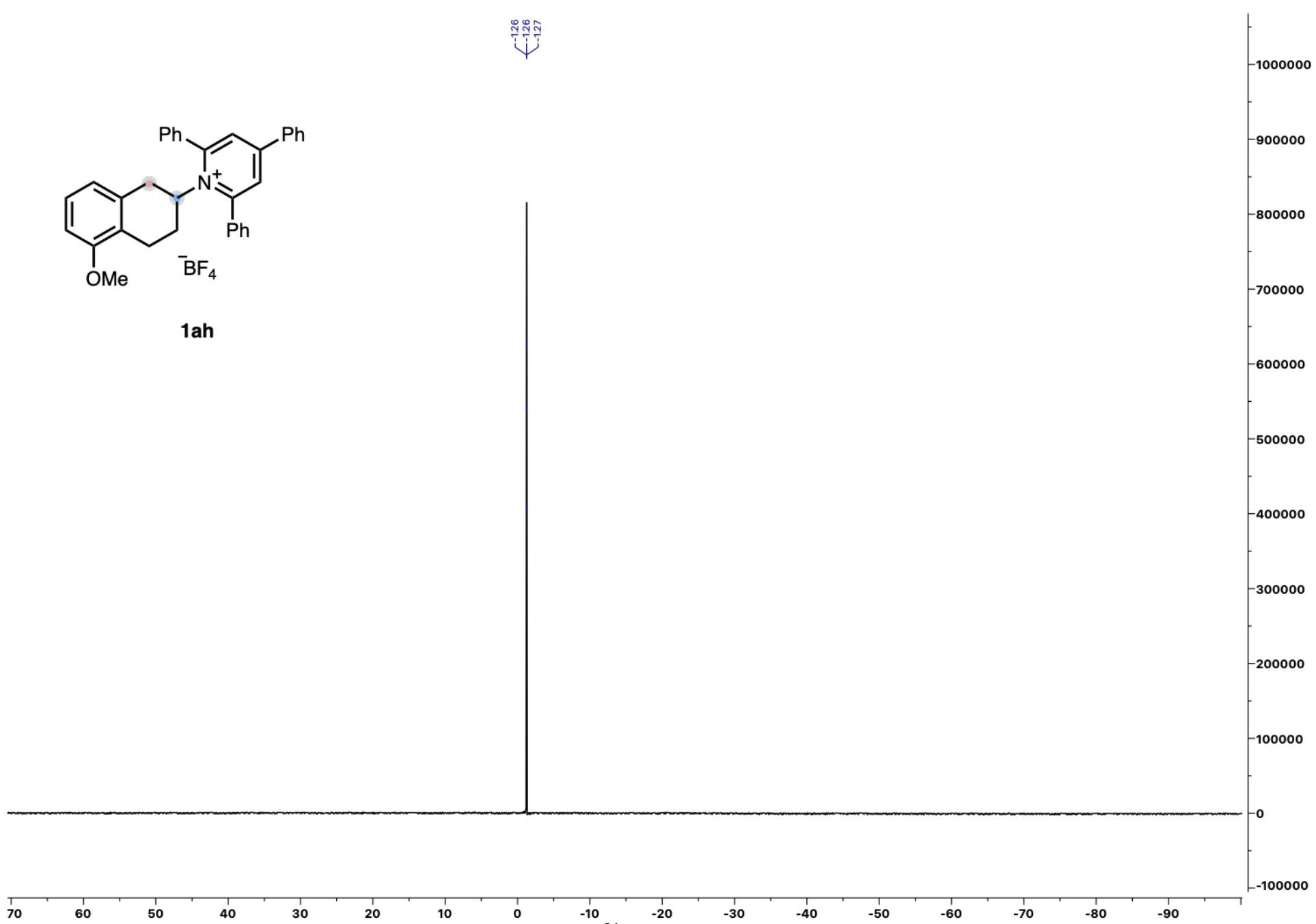

1-(1-(furan-2-yl)propan-2-yl)-2,4,6-triphenylpyridin-1-ium tetrafluoroborate **1ai**:

^{1}H NMR (500 MHz, $CDCl_3$):

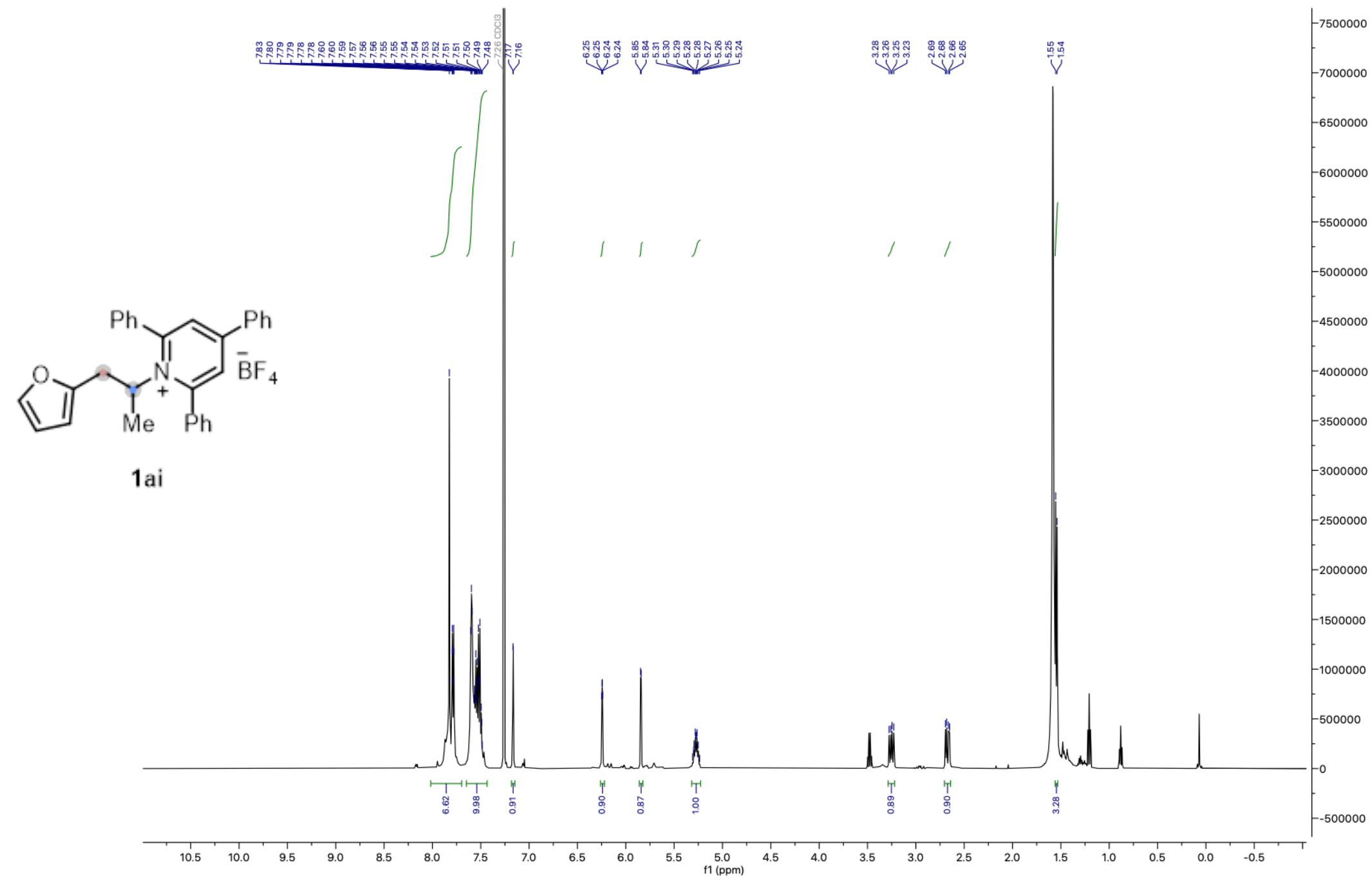

^{13}C NMR (126 MHz, $CDCl_3$):

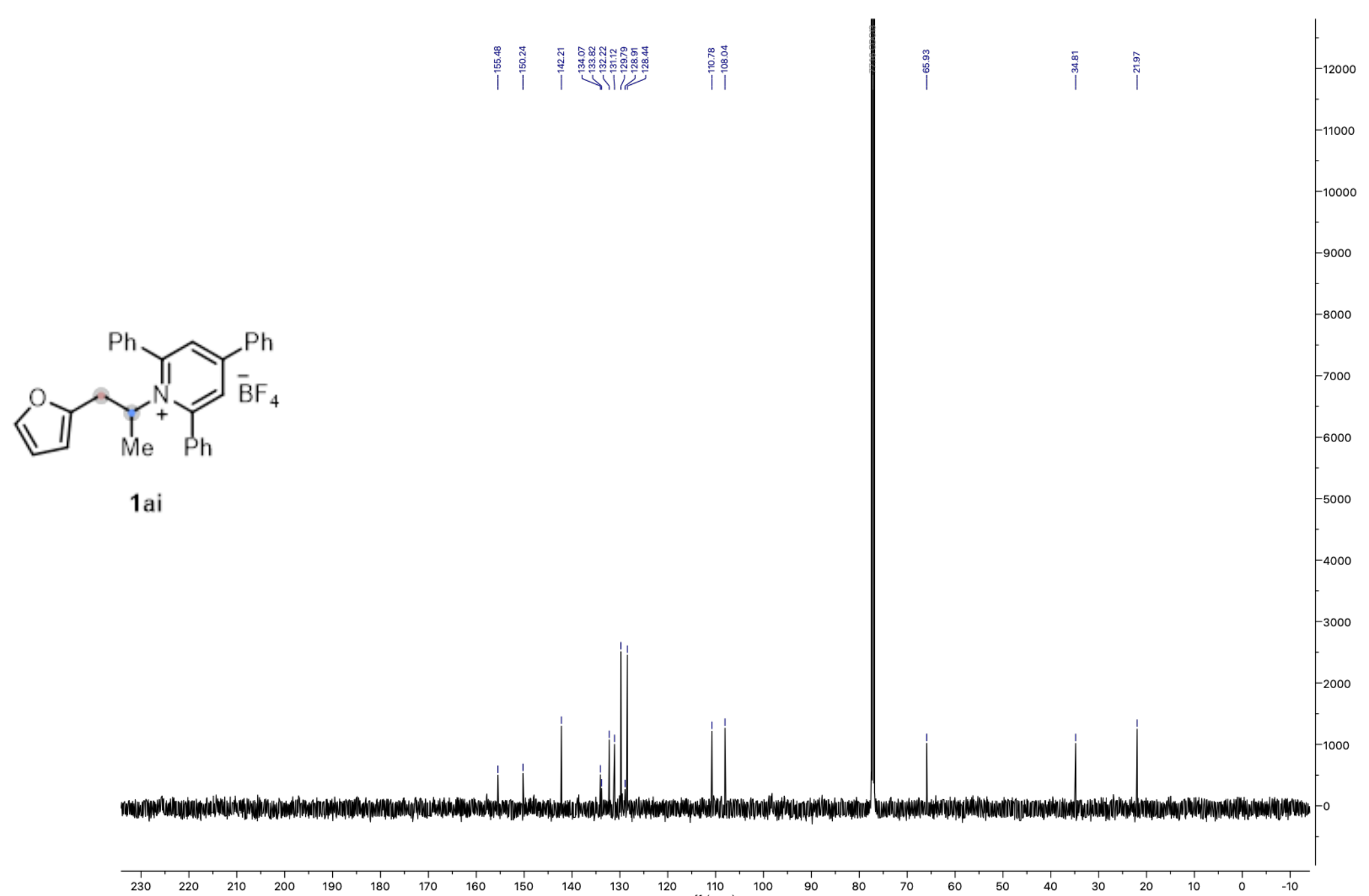

^{19}F NMR (282 MHz, $CDCl_3$):

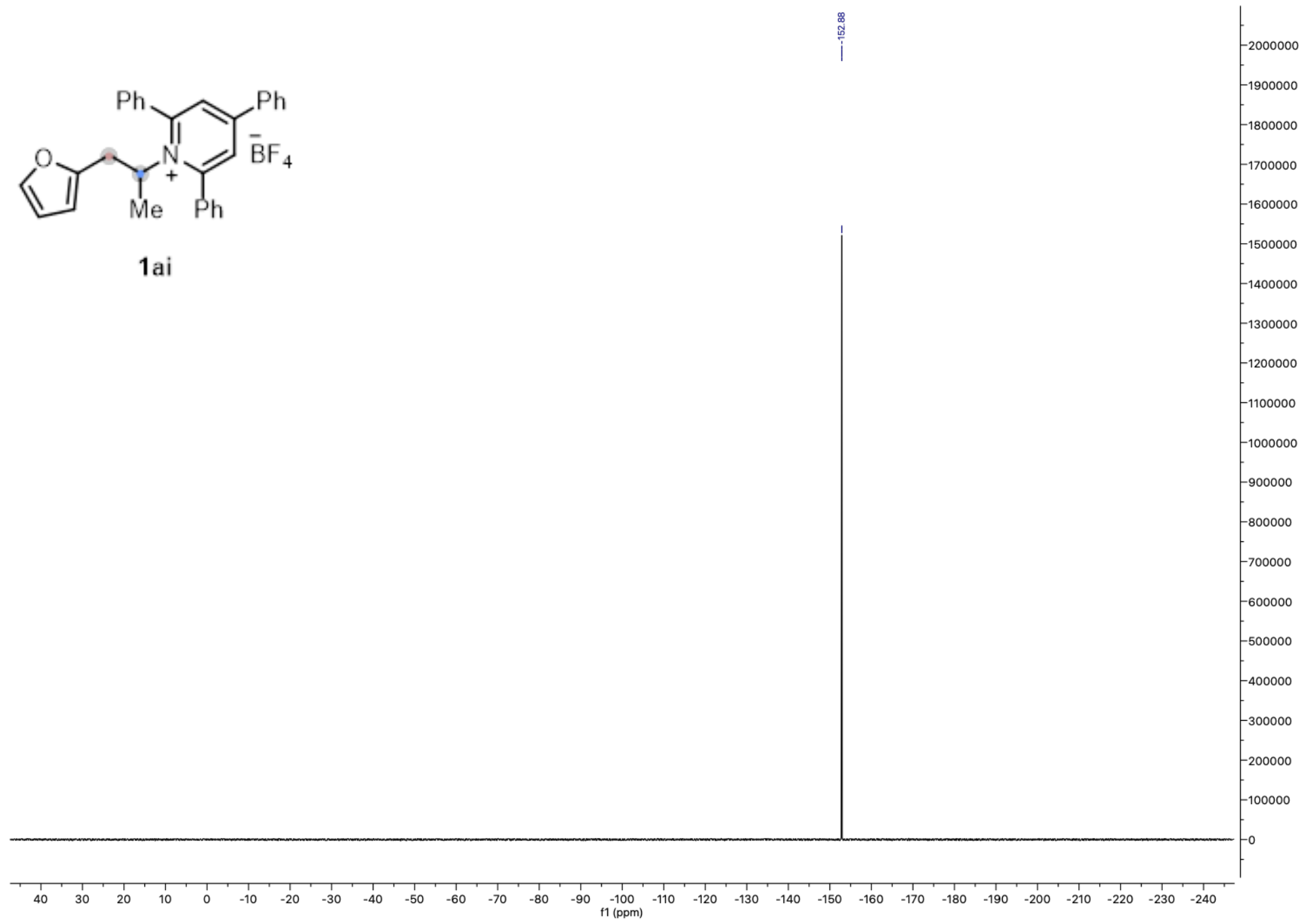

^{11}B NMR (96 MHz, $CDCl_3$):

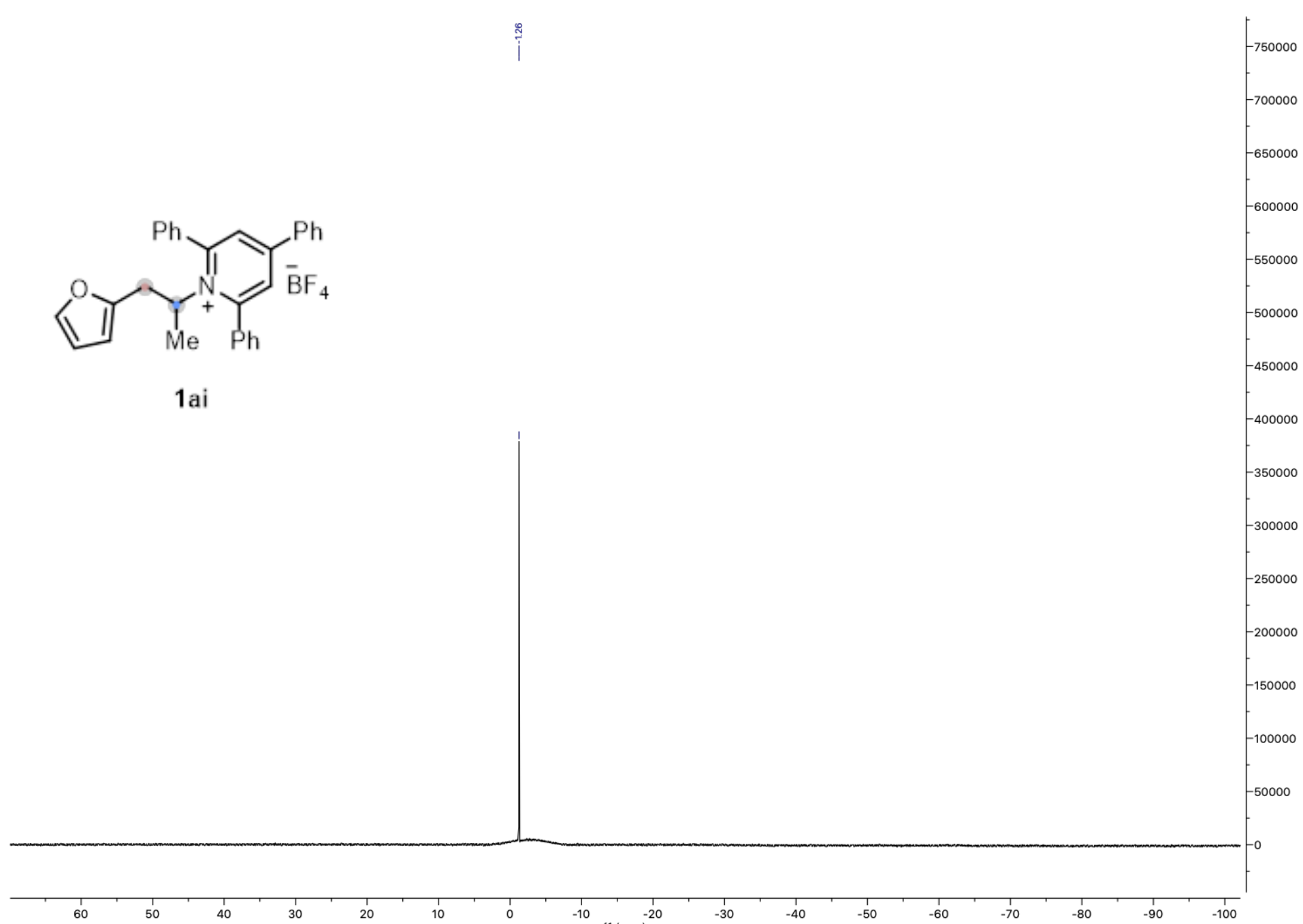

2,4,6-triphenyl-1-(1-(thiophen-2-yl)propan-2-yl)pyridin-1-ium tetrafluoroborate **1aj**:

^{1}H NMR (500 MHz, $CDCl_3$):

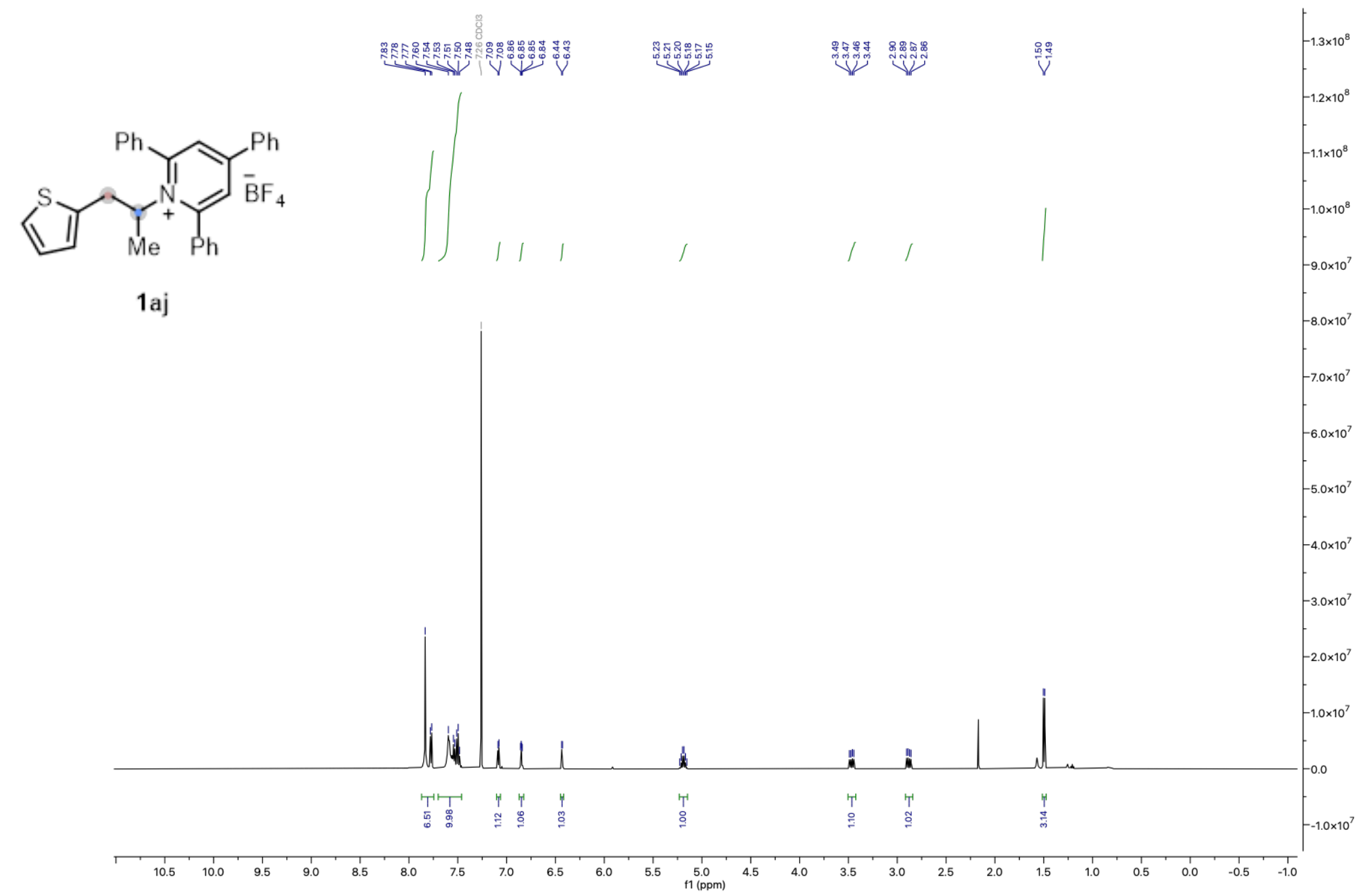

^{13}C NMR (126 MHz, $CDCl_3$):

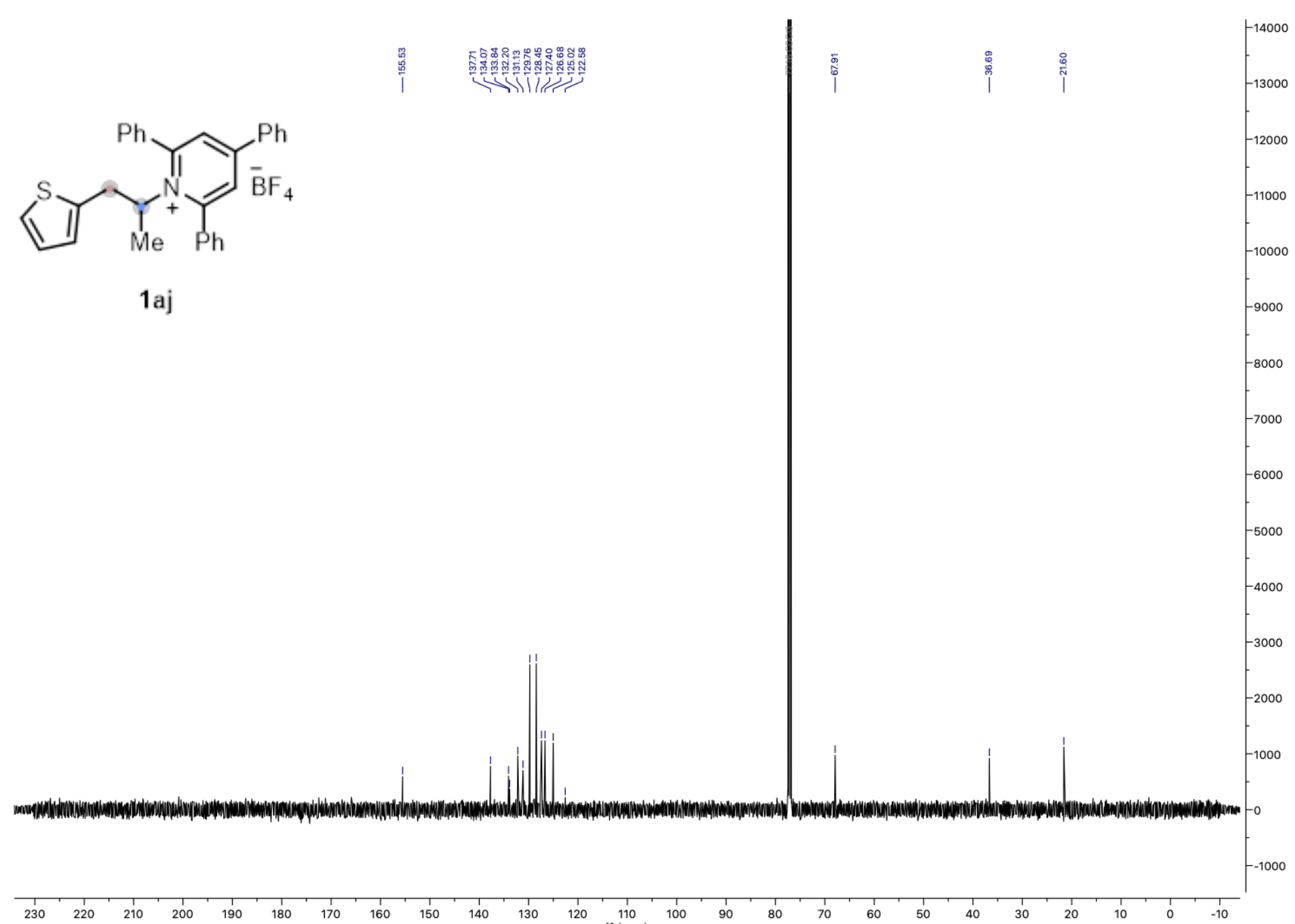

^{19}F NMR (282 MHz, $CDCl_3$):

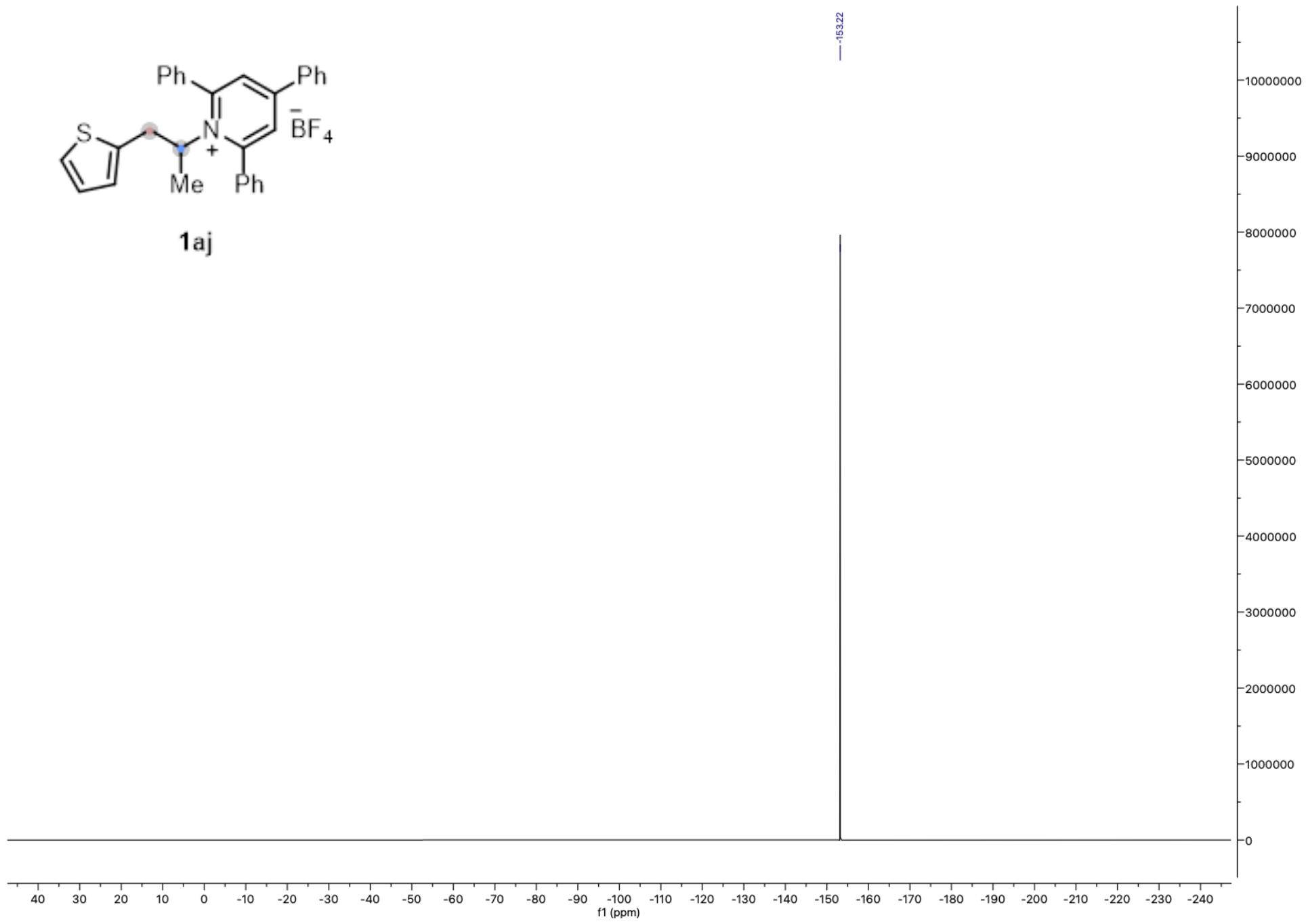

^{11}B NMR (96 MHz, $CDCl_3$):

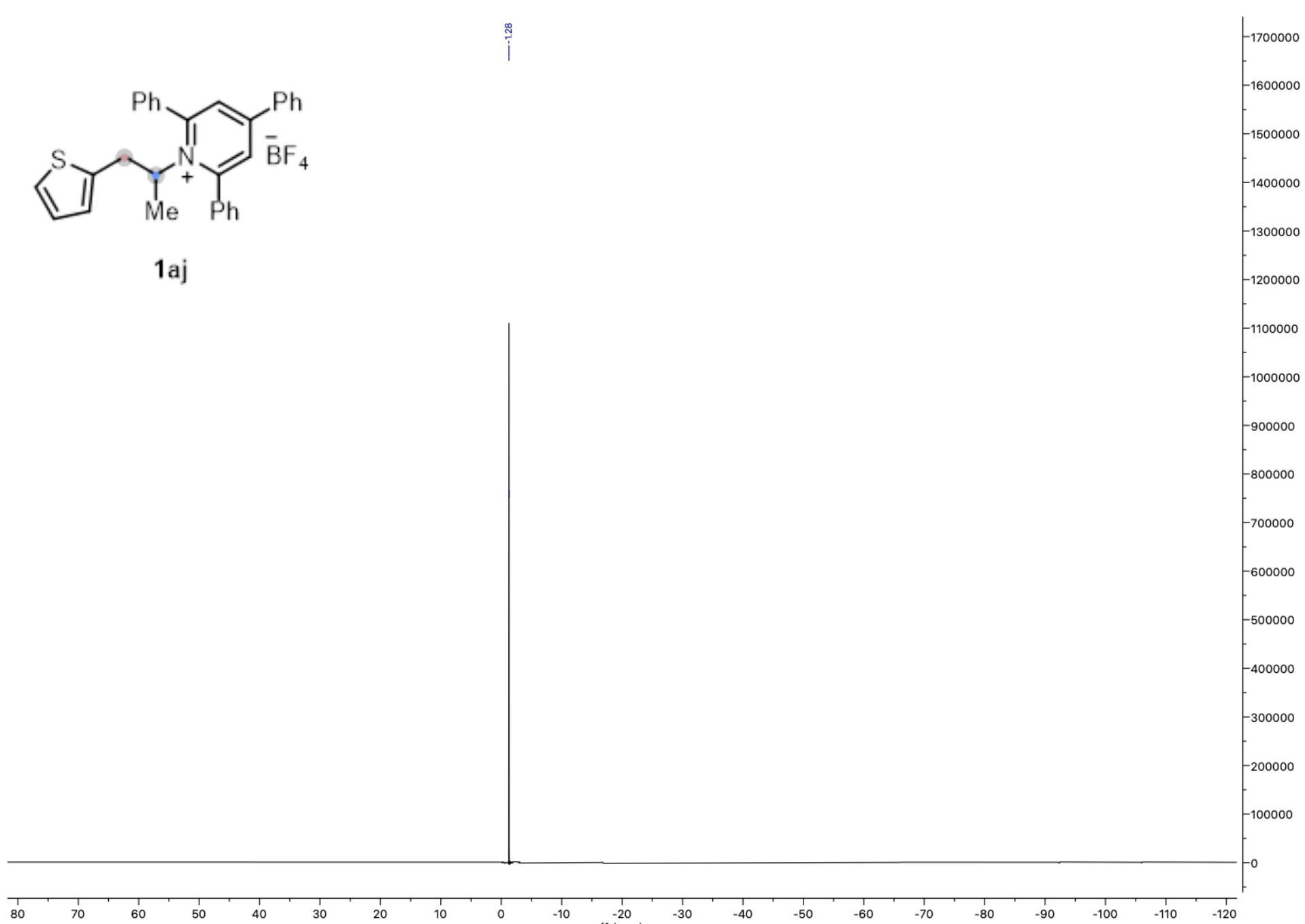

1-(1-(benzofuran-3-yl)propan-2-yl)-2,4,6-triphenylpyridin-1-ium tetrafluoroborate **1ak**:

^{1}H NMR (500 MHz, CDCl$_3$):

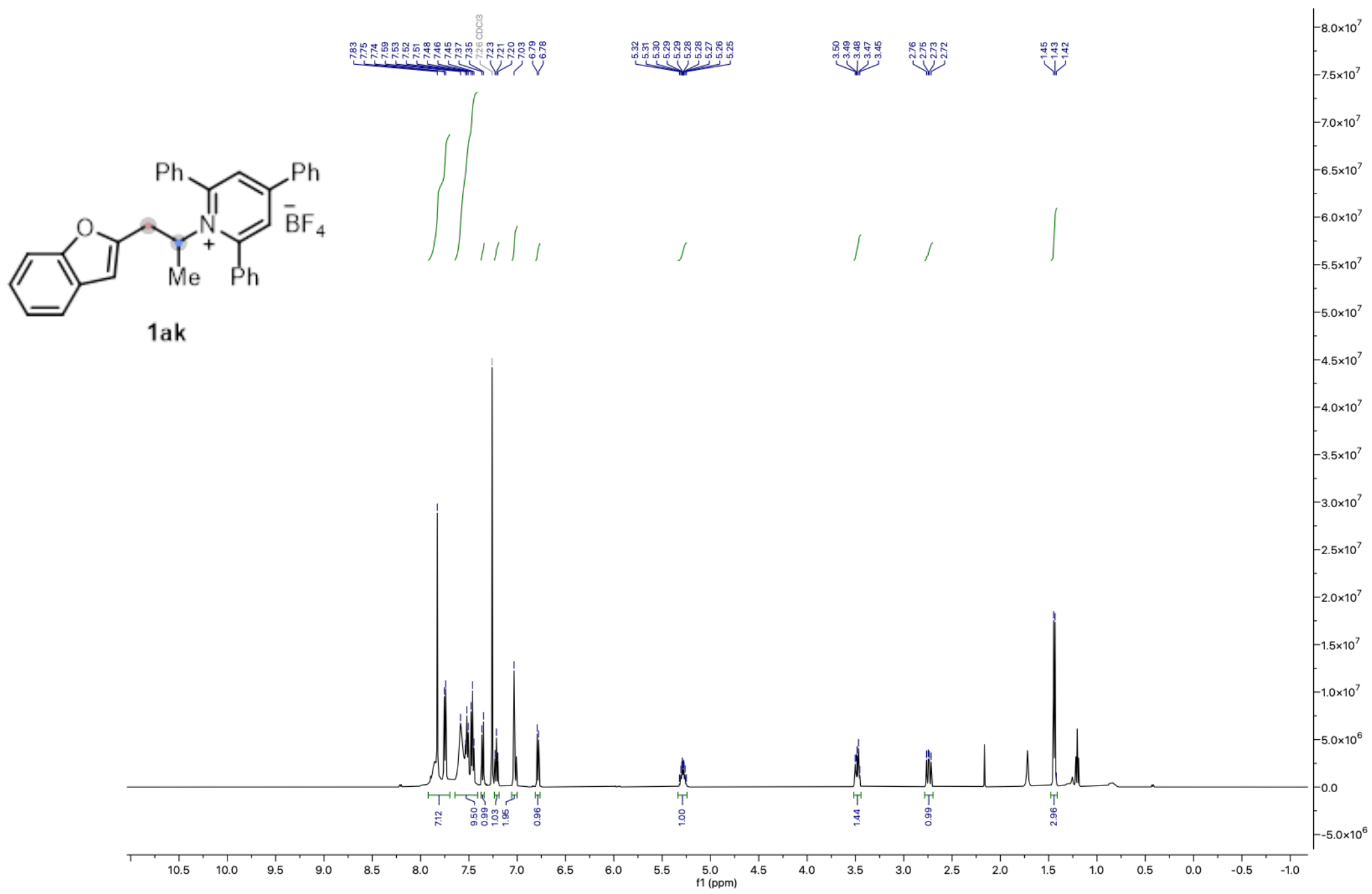

1ak

^{13}C NMR (126 MHz, CDCl$_3$):

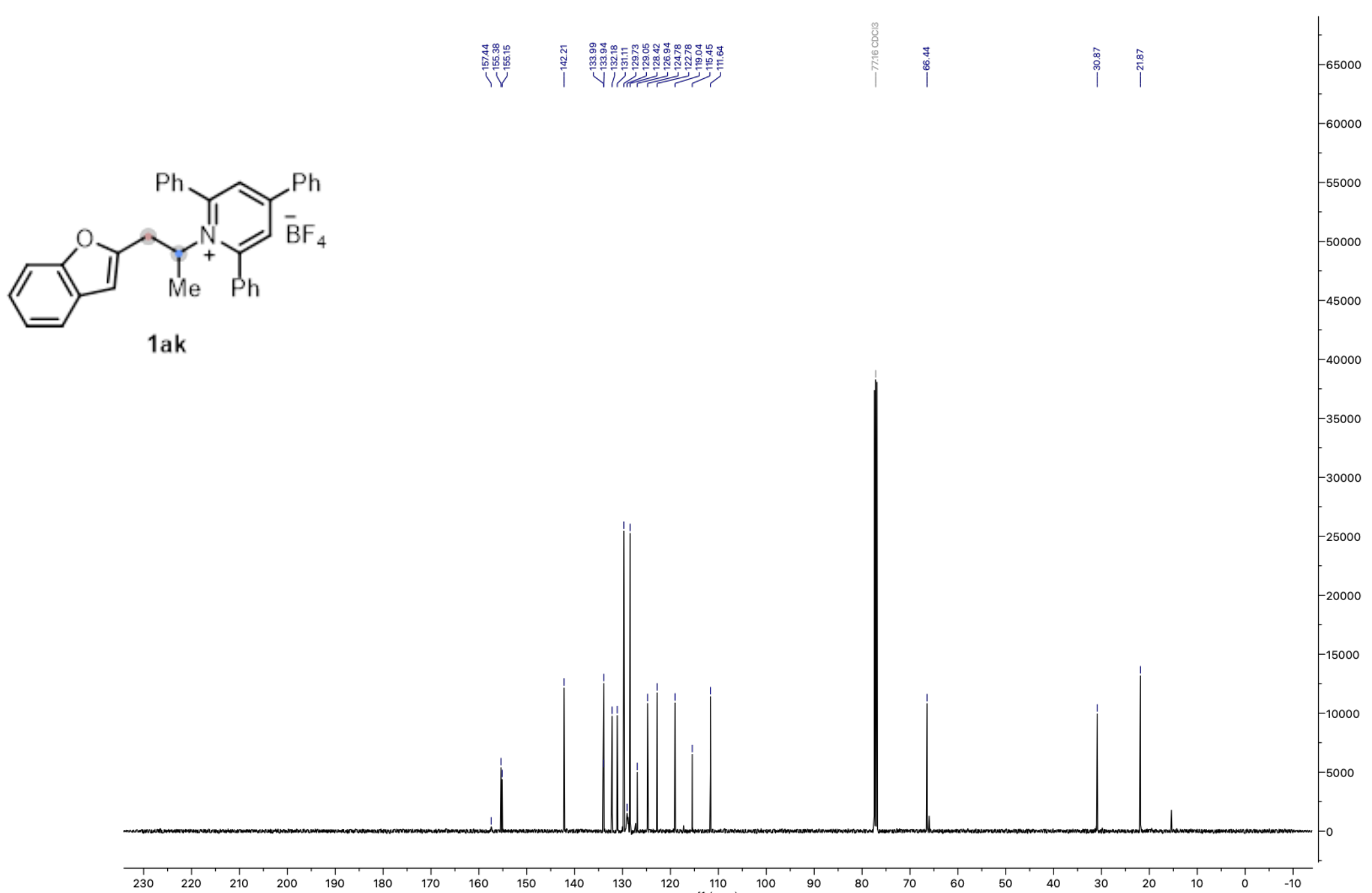

1ak

^{19}F NMR (282 MHz, $CDCl_3$):

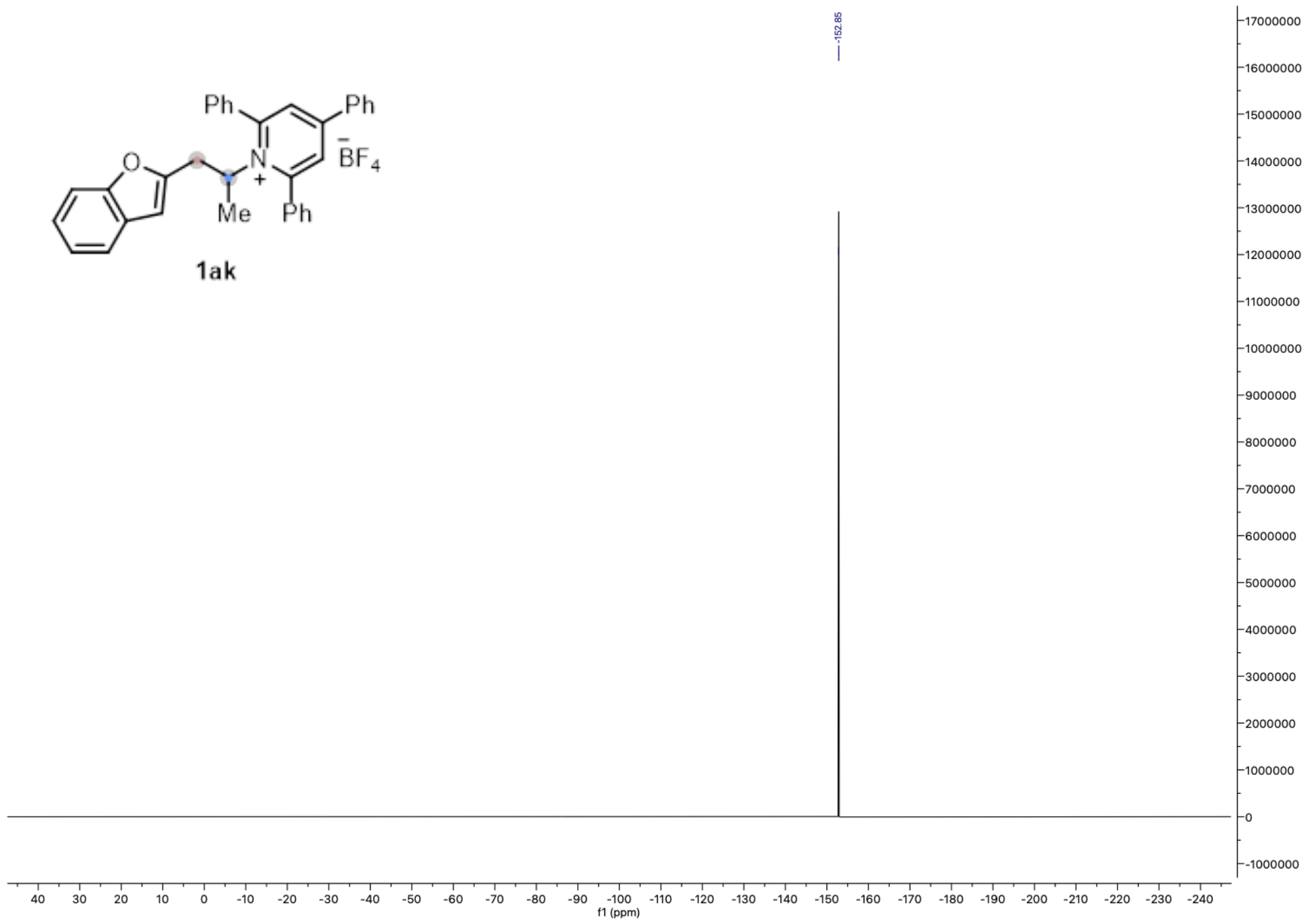

^{11}B NMR (96 MHz, $CDCl_3$):

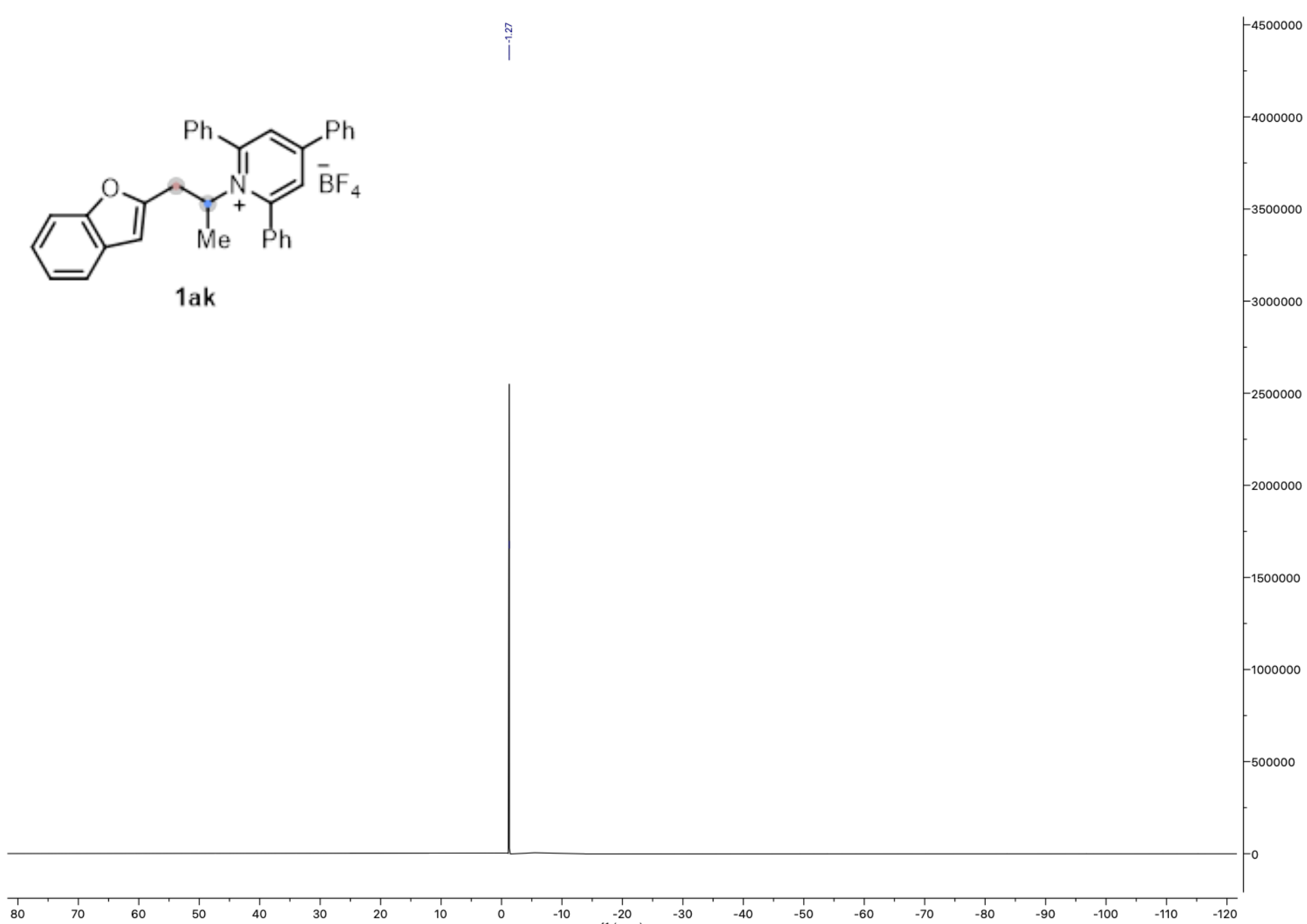

(*Z*)-1-(1-(11-(3-(dimethylamino)propylidene)-6,11-dihydrodibenzo[*b*,*e*]oxepin-2-yl)propan-2-yl)-2,4,6-triphenylpyridin-1-ium tetrafluoroborate **1as:**

^{1}H NMR (500 MHz, $CDCl_3$):

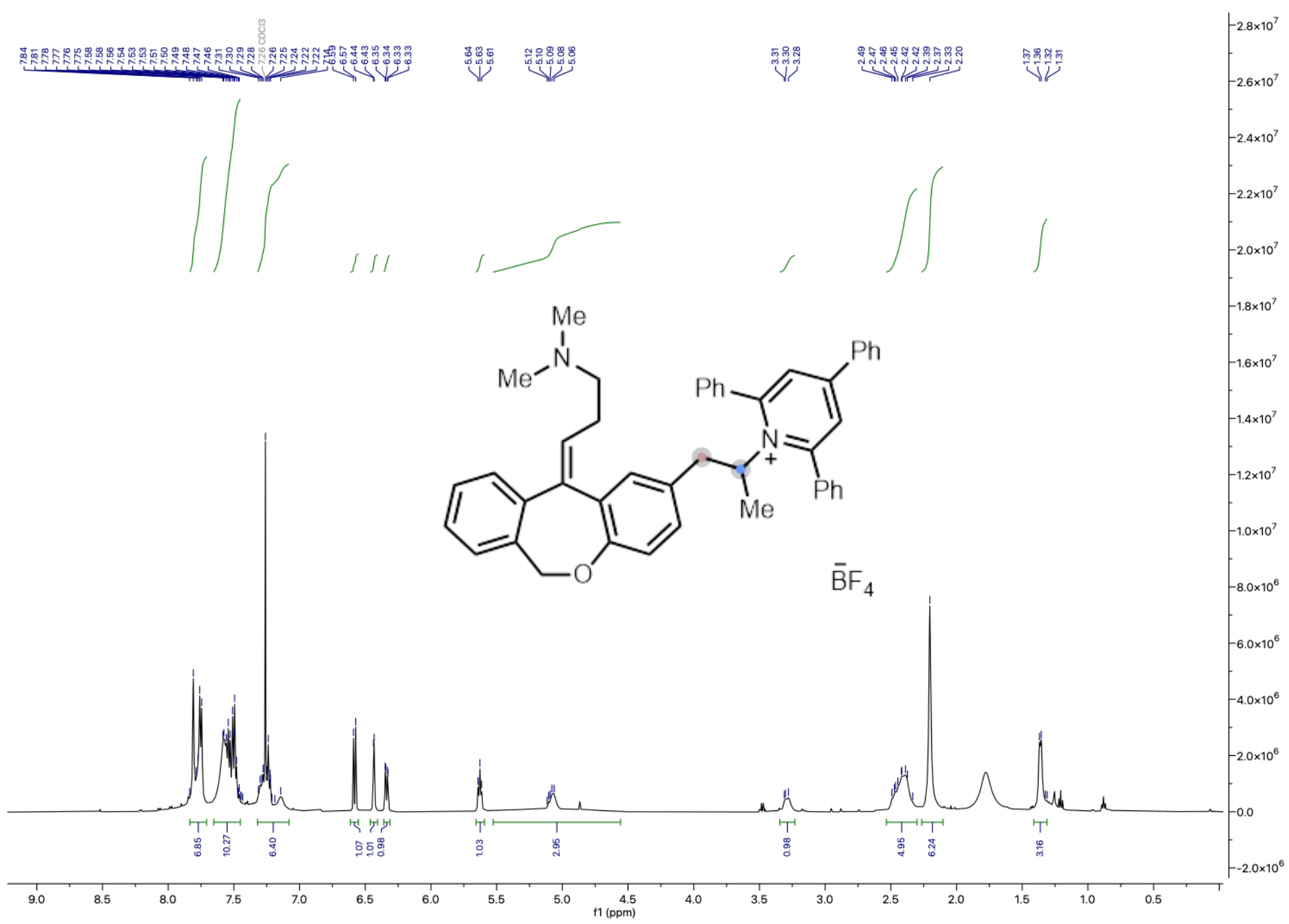

^{13}C NMR (126 MHz, $CDCl_3$):

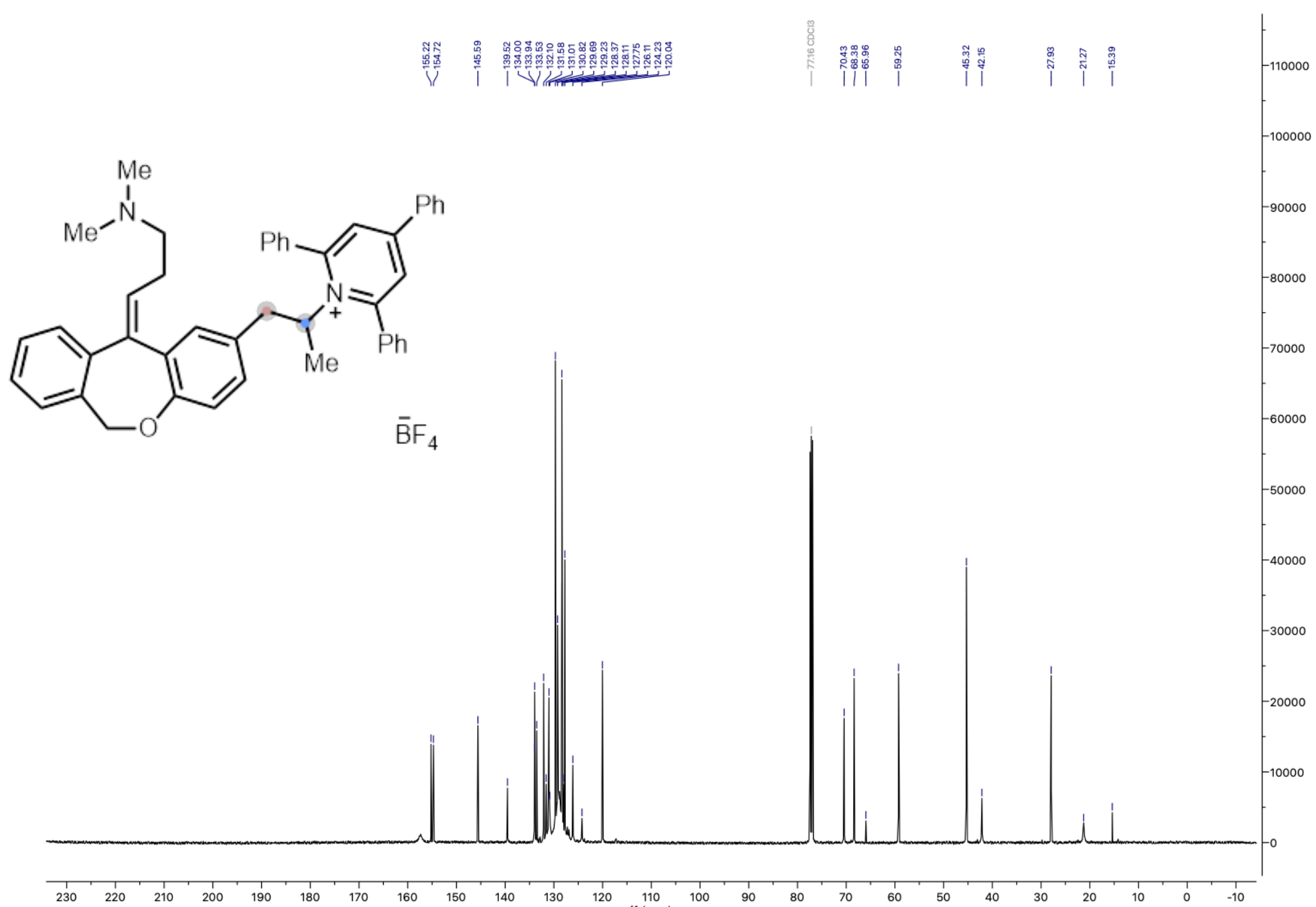

^{19}F NMR (282 MHz, $CDCl_3$):

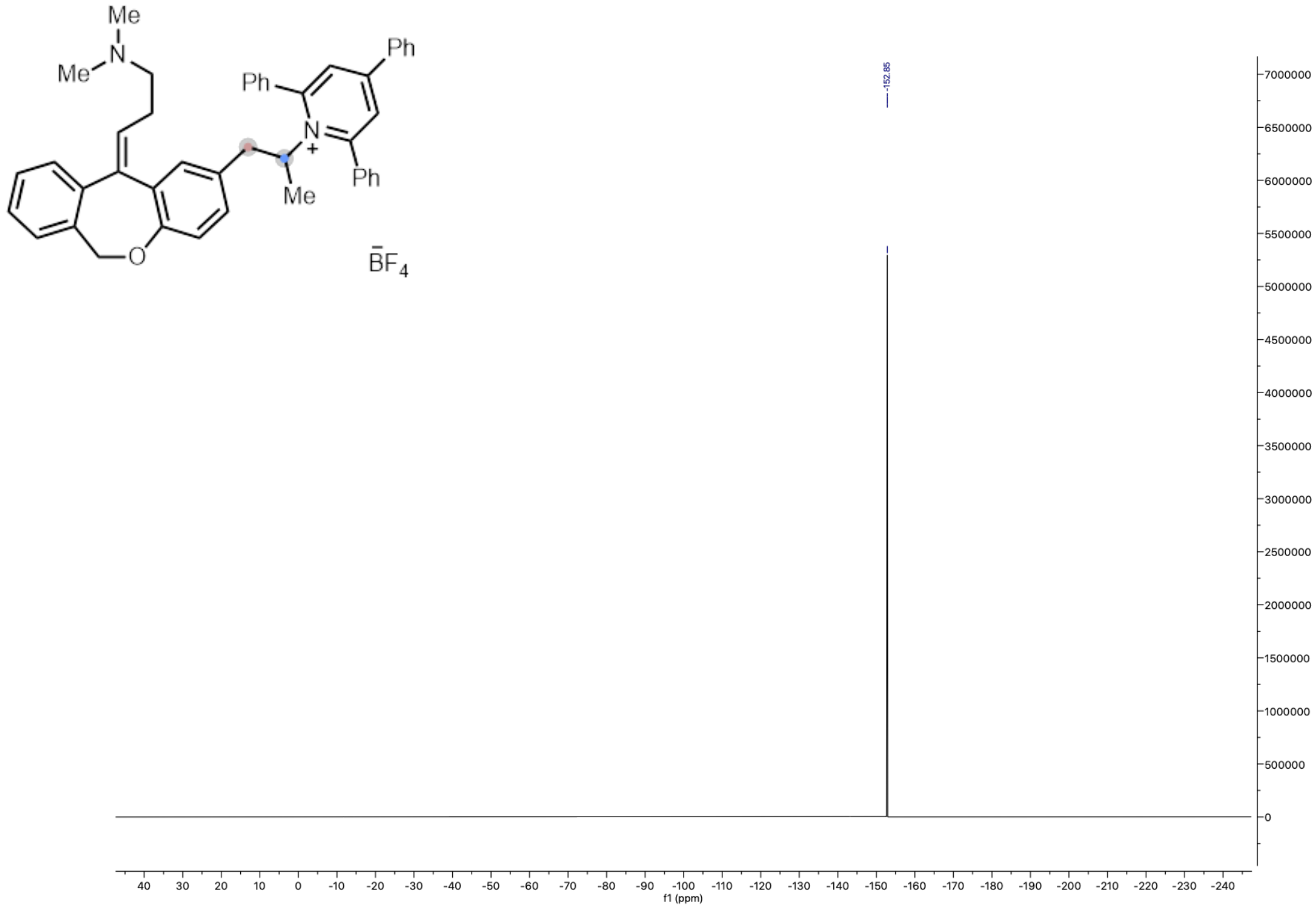

^{11}B NMR (96 MHz, $CDCl_3$):

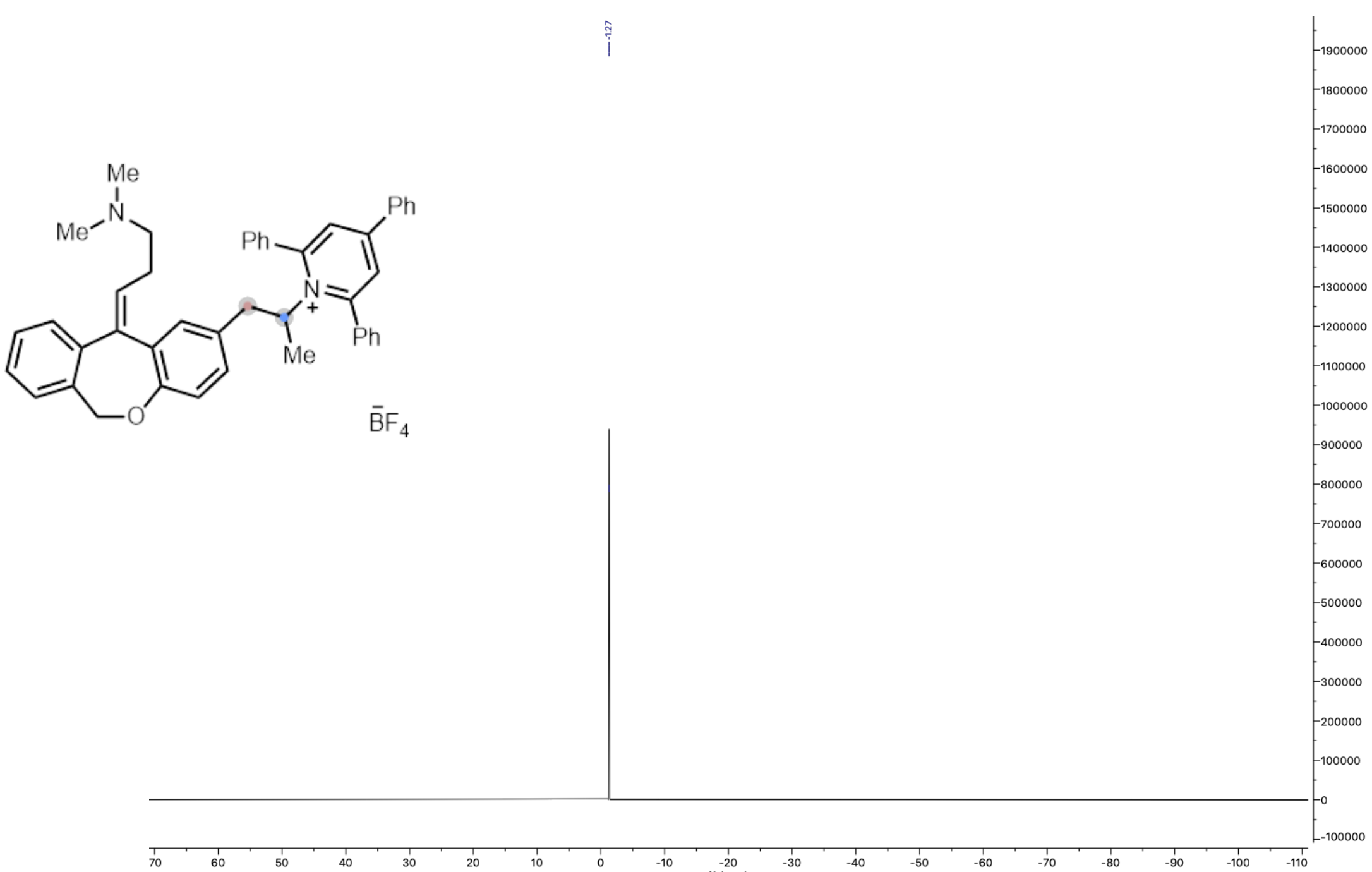

1-(4-(4-((4-(6-(1-(2,2-Difluorobenzo[d][1,3]dioxol-5-yl)cyclopropane-1-carboxamido)-3-methylpyridin-2-yl)benzoyl)oxy)phenyl)butan-2-yl)-2,4,6-triphenylpyridin-1-ium tetrafluoroborate **1aw**:

^{1}H NMR (500 MHz, $(CD_3)_2CO$):

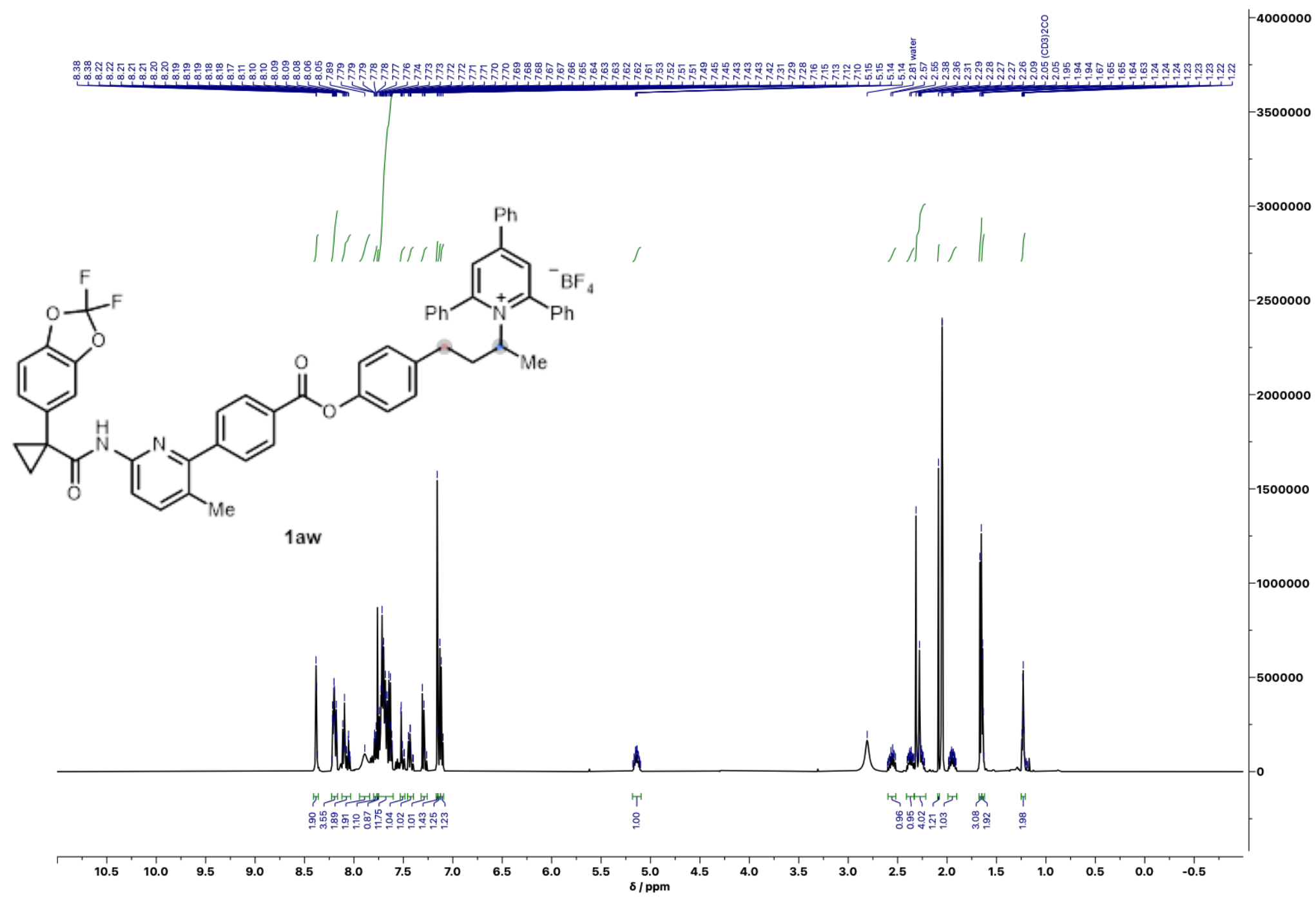

^{13}C NMR (126 MHz, $(CD_3)_2CO$):

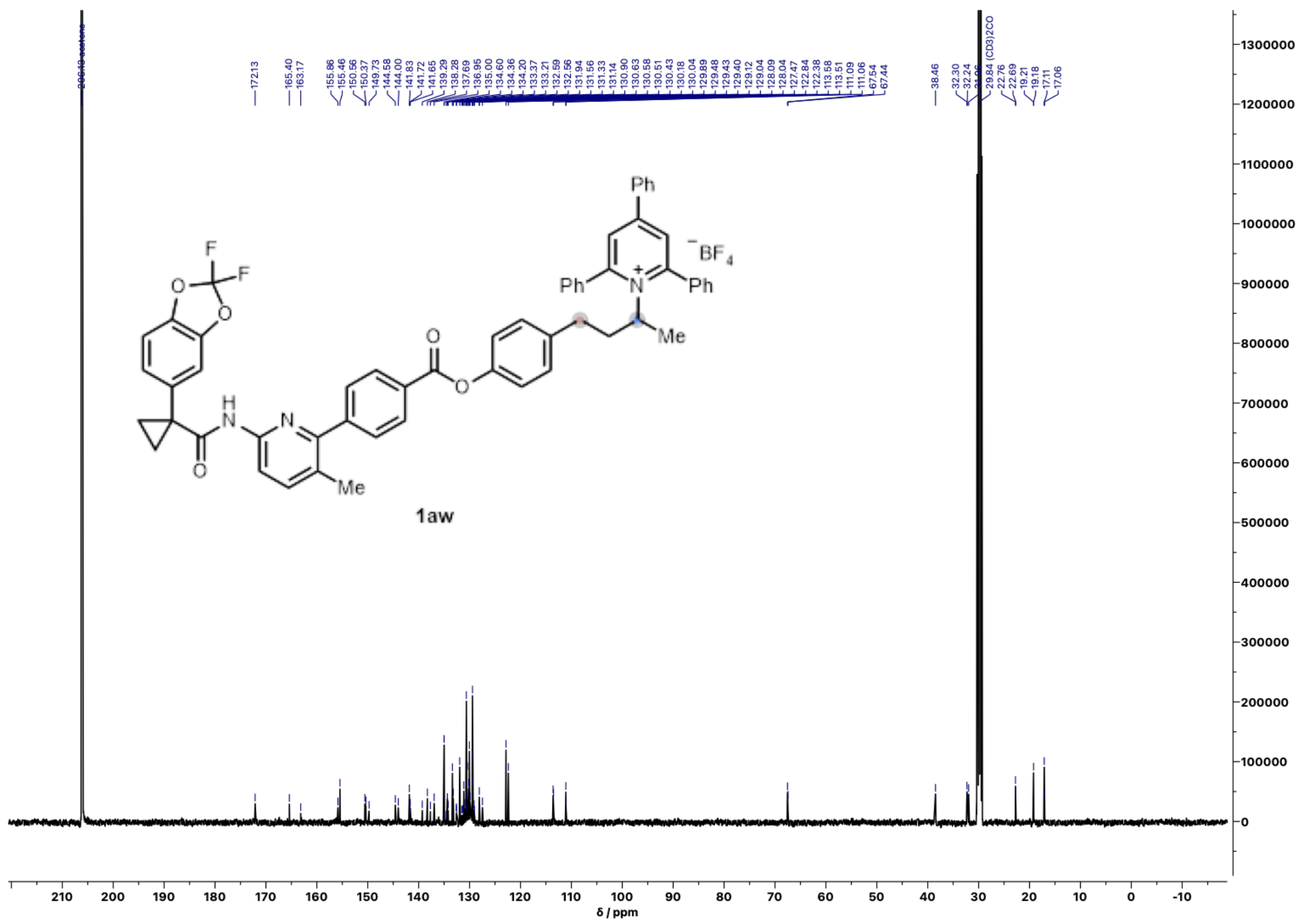

^{19}F NMR (282 MHz, $(CD_3)_2CO$):

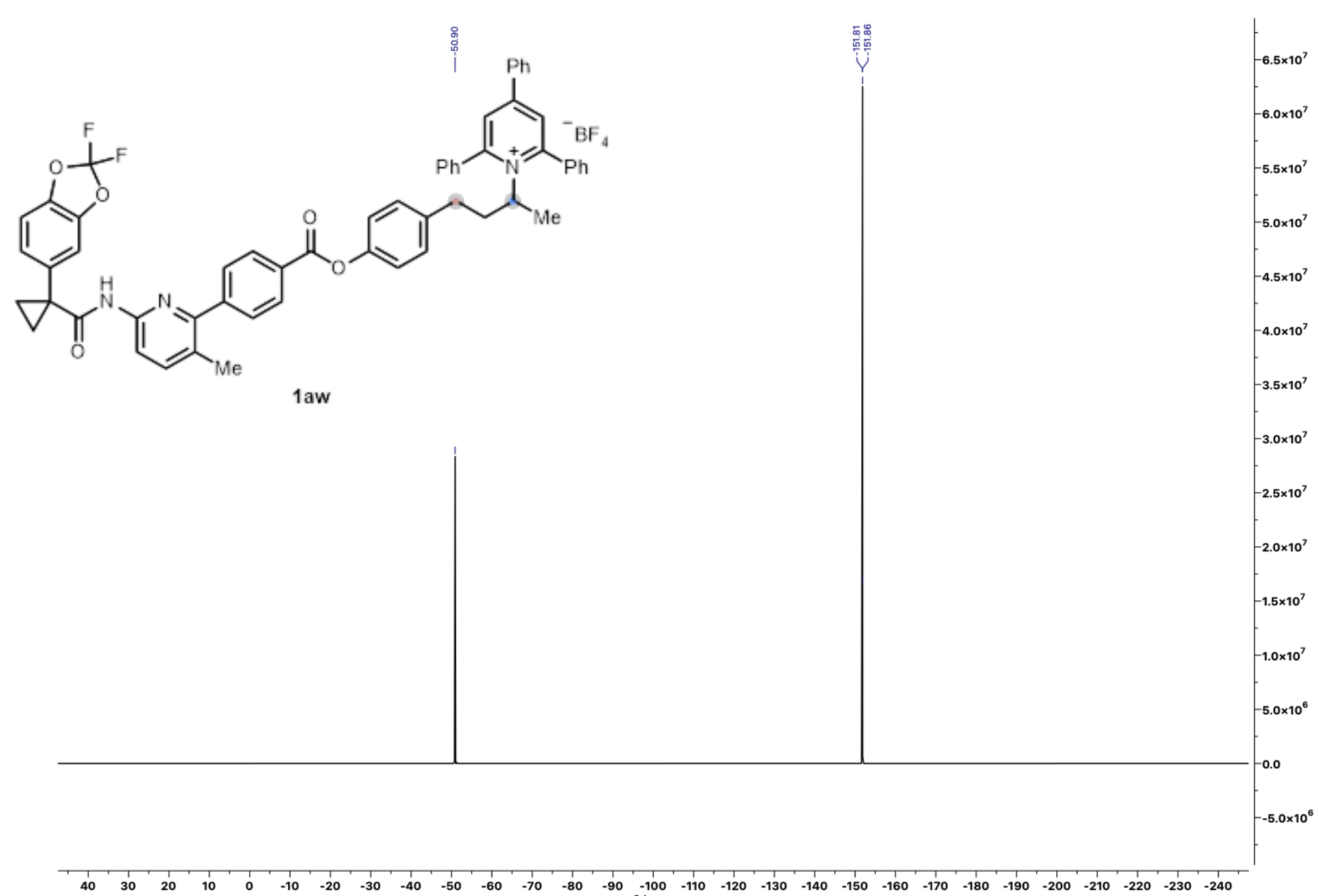

^{11}B NMR (96 MHz, $(CD_3)_2CO$):

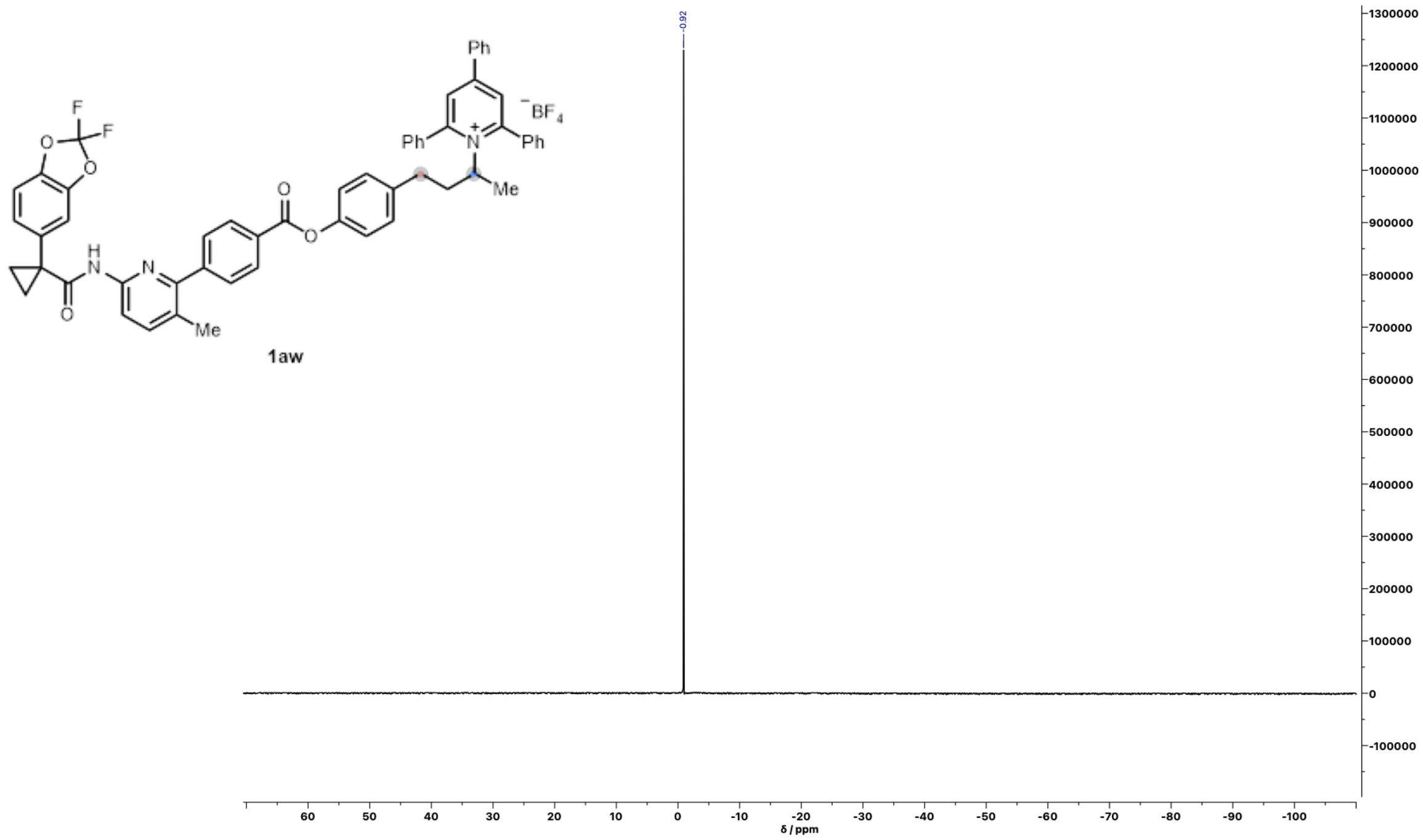

1-(4-(4-(((R)-2-(6-Methoxynaphthalen-2-yl)propanoyl)oxy)phenyl)butan-2-yl)-2,4,6-triphenylpyridin-1-ium tetrafluoroborate **1ax**:

^{1}H NMR (500 MHz, $(CD_3)_2CO$):

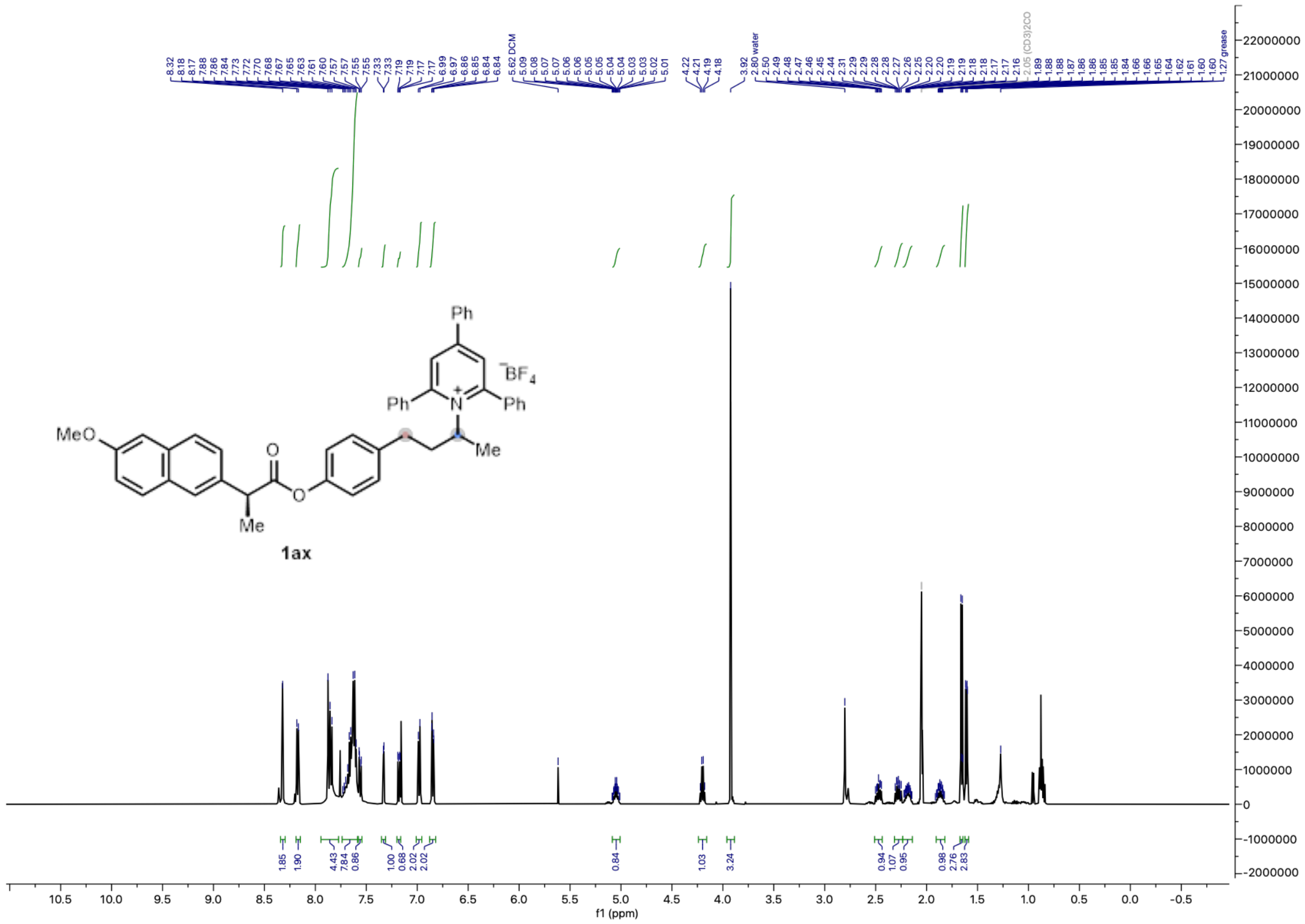

^{13}C NMR (126 MHz, $(CD_3)_2CO$):

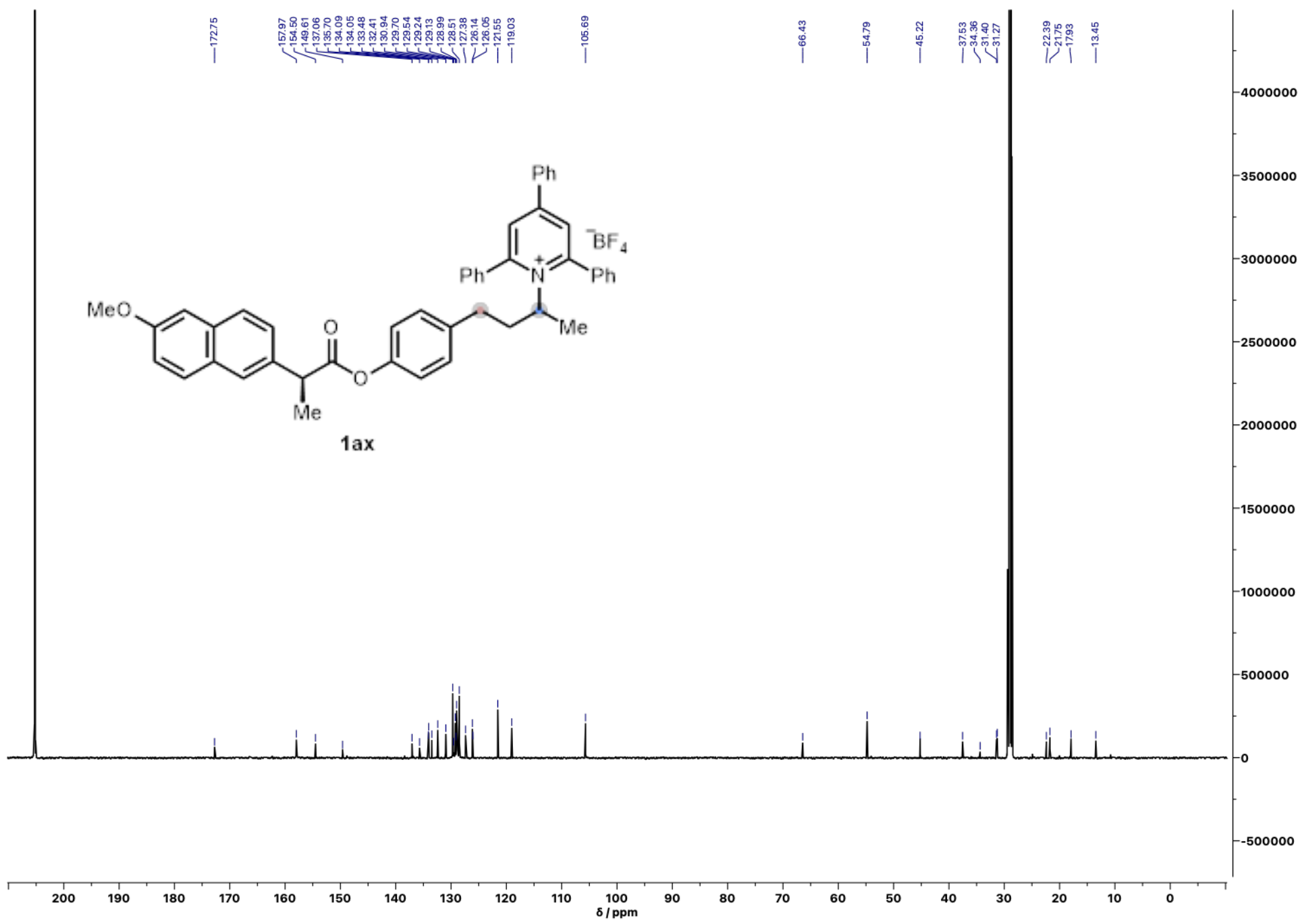

^{19}F NMR (282 MHz, $(CD_3)_2CO$):

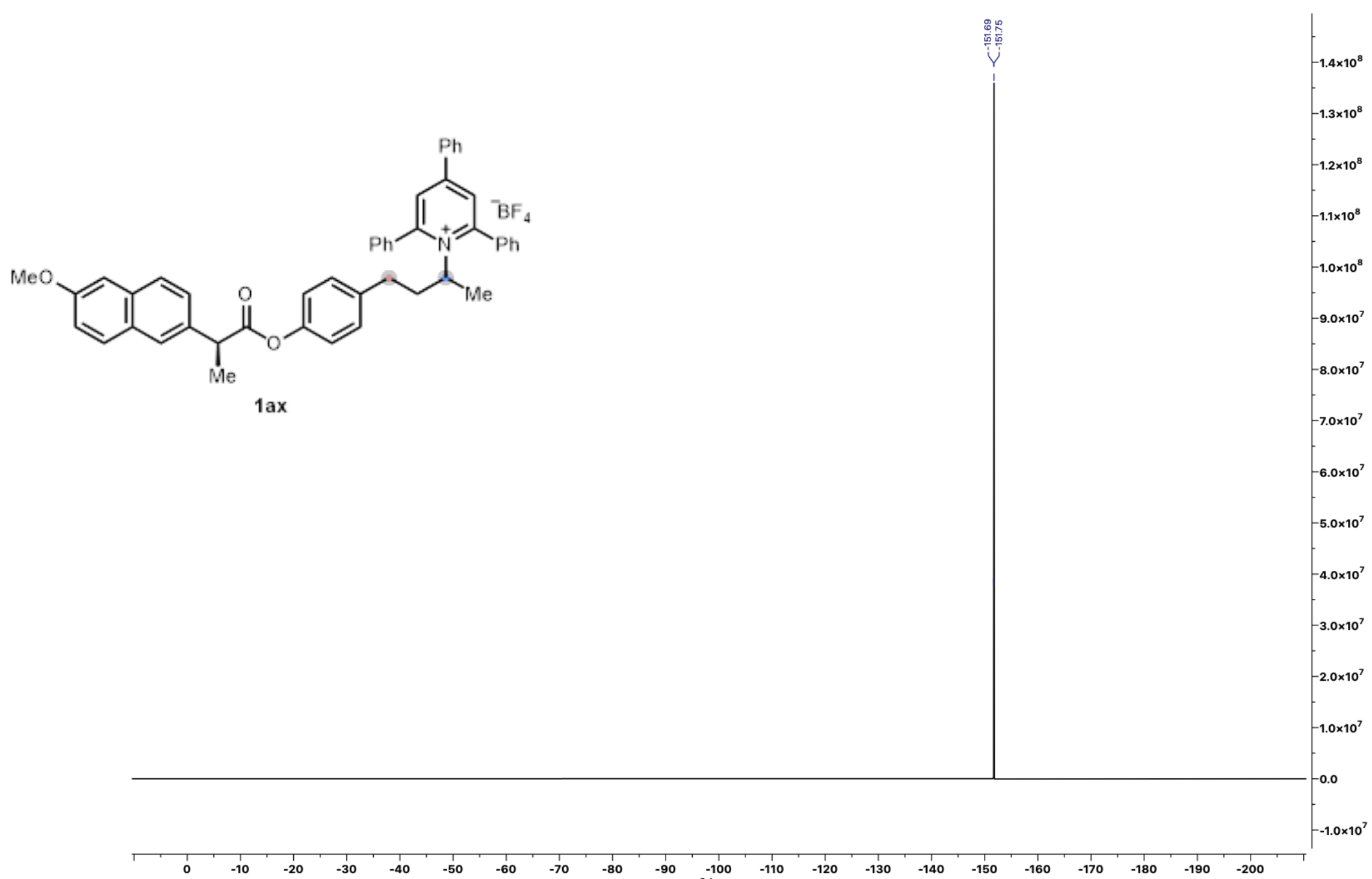

^{11}B NMR (96 MHz, $(CD_3)_2CO$):

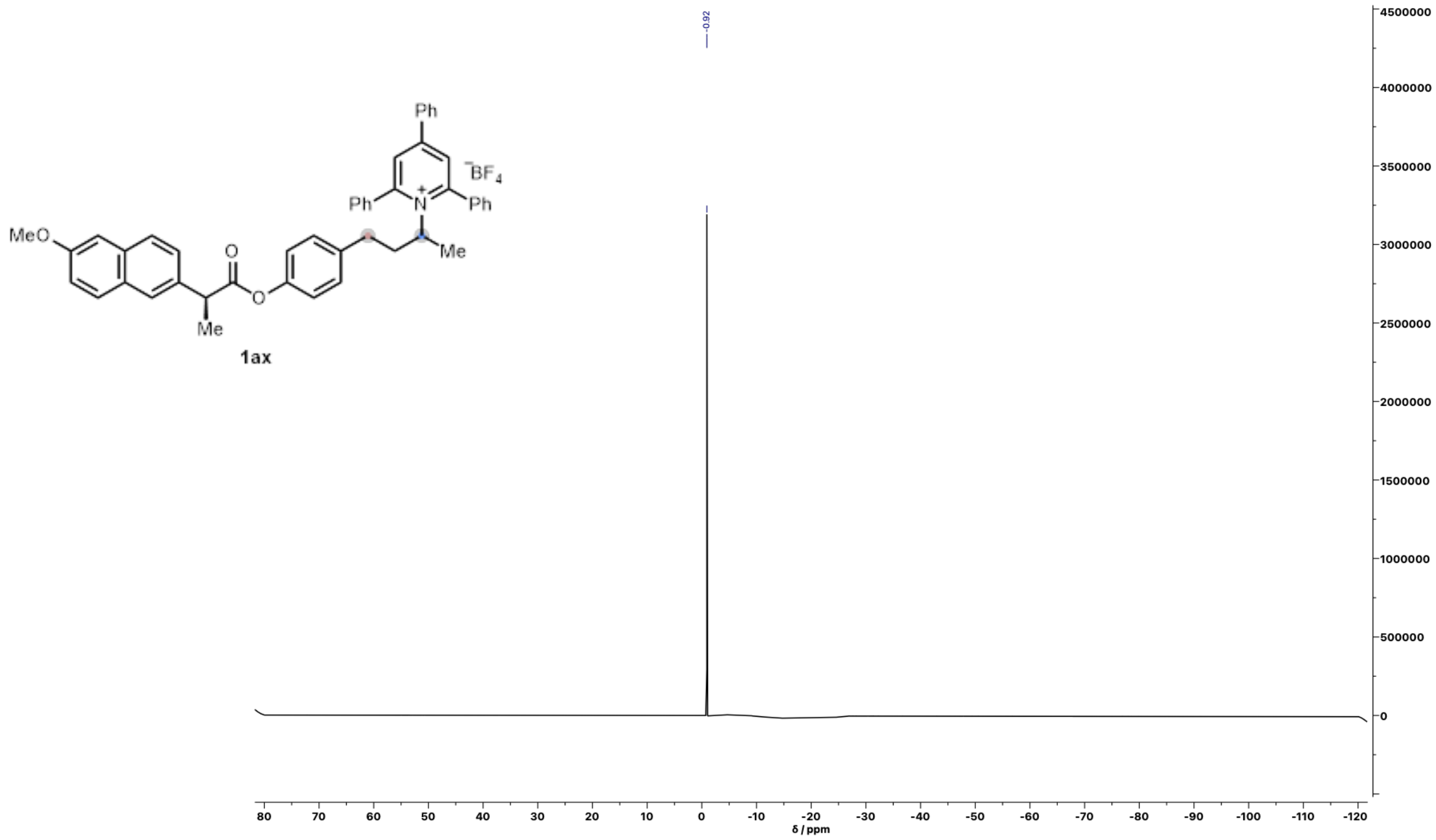

1-(4-(4-((2-(3-Cyano-4-isobutoxyphenyl)-4-methylthiazole-5-carbonyl)oxy)phenyl)butan-2-yl)-2,4,6-triphenylpyridin-1-ium tetrafluoroborate **1ay**:

^{1}H NMR (500 MHz, $(CD_3)_2CO$):

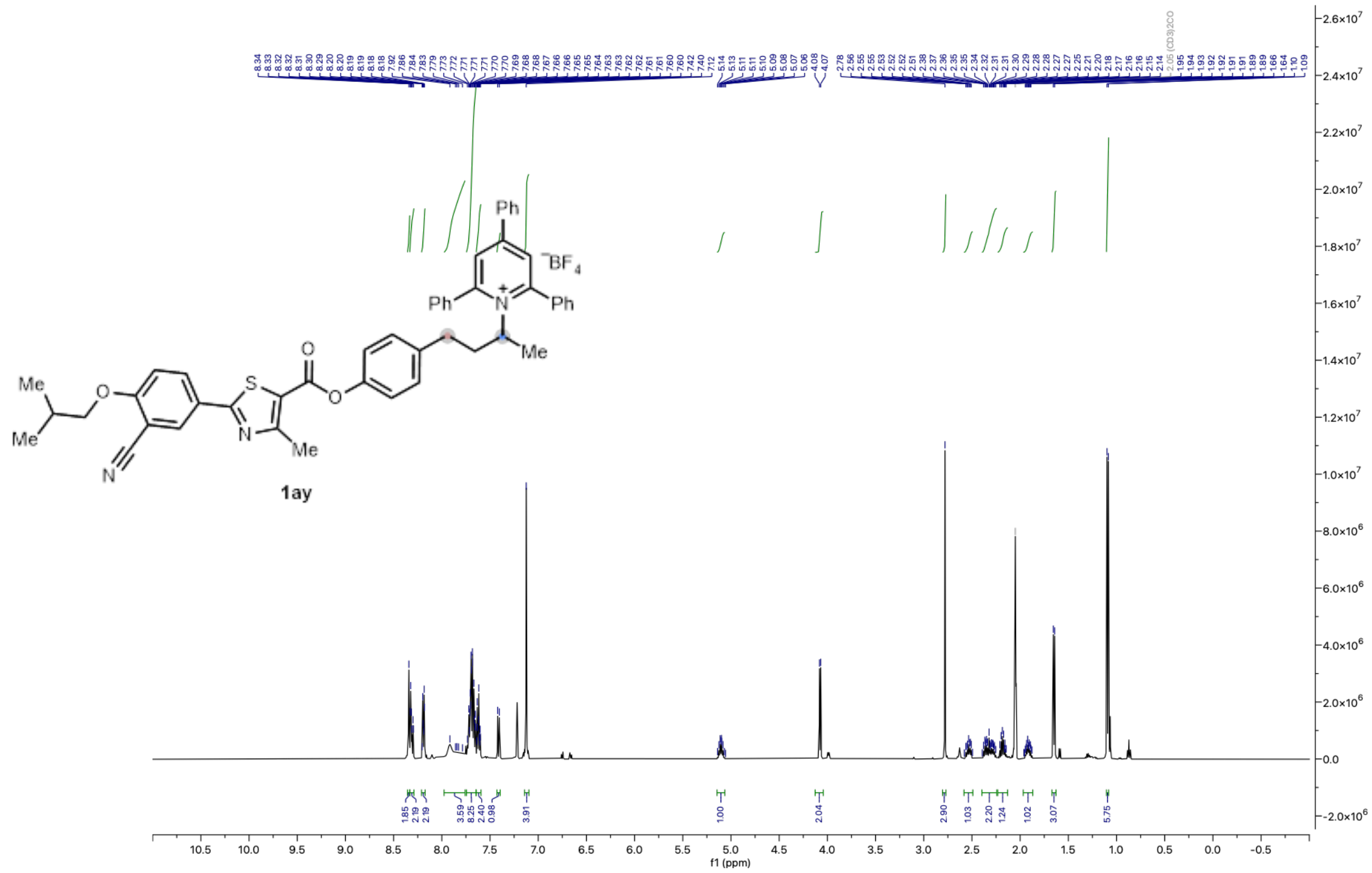

^{13}C NMR (126 MHz, $(CD_3)_2CO$):

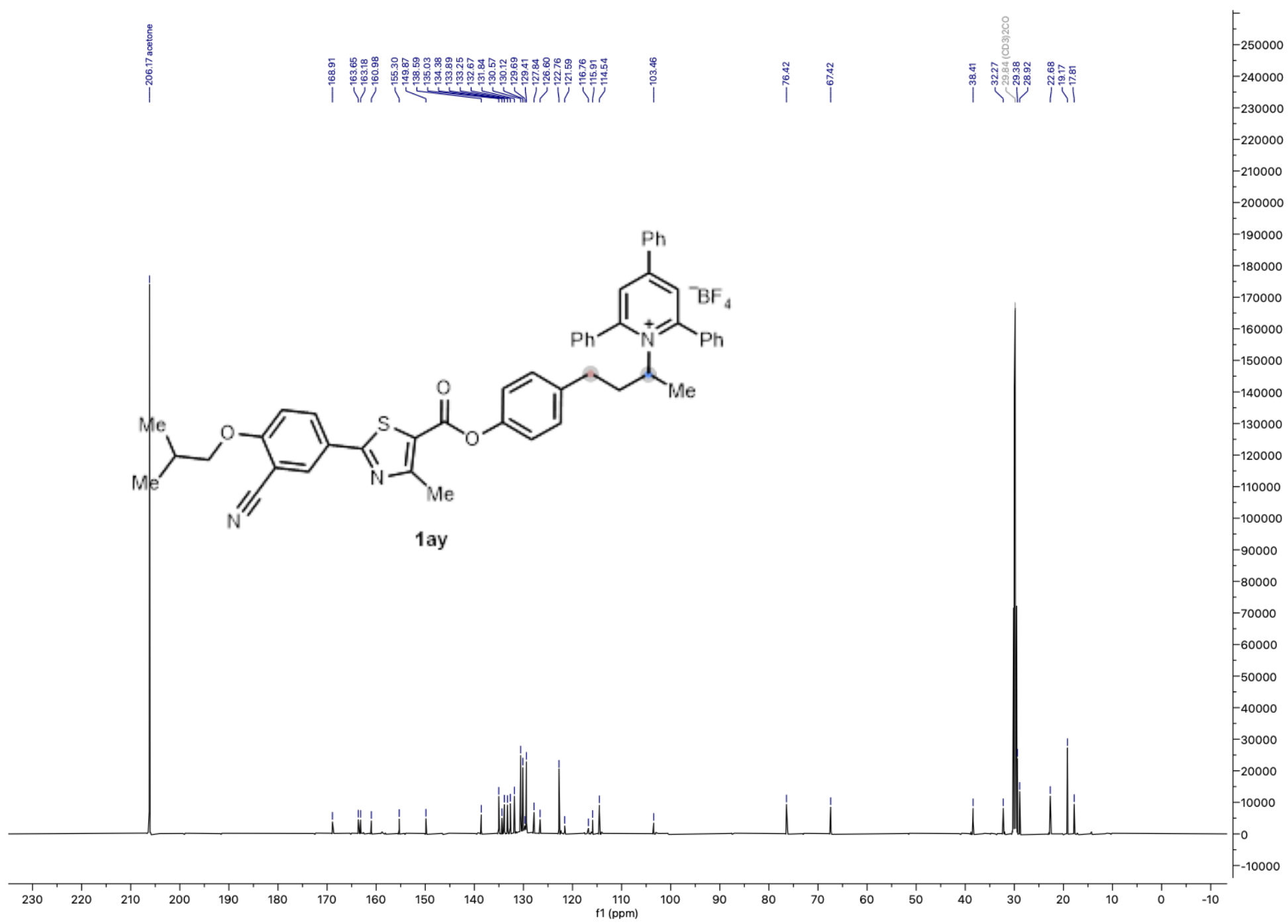

^{19}F NMR (282 MHz, $(CD_3)_2CO$):

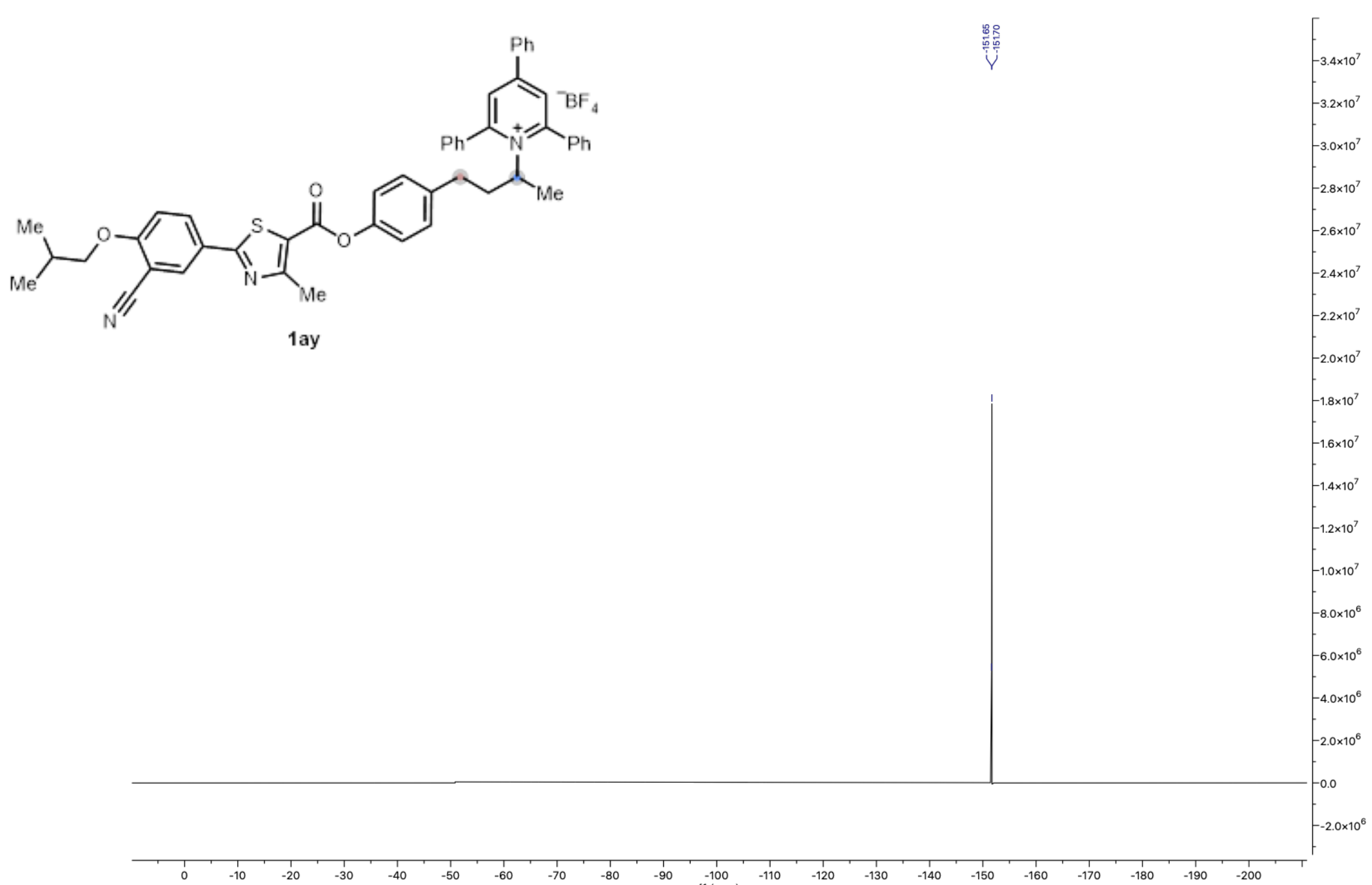

^{11}B NMR (96 MHz, $(CD_3)_2CO$):

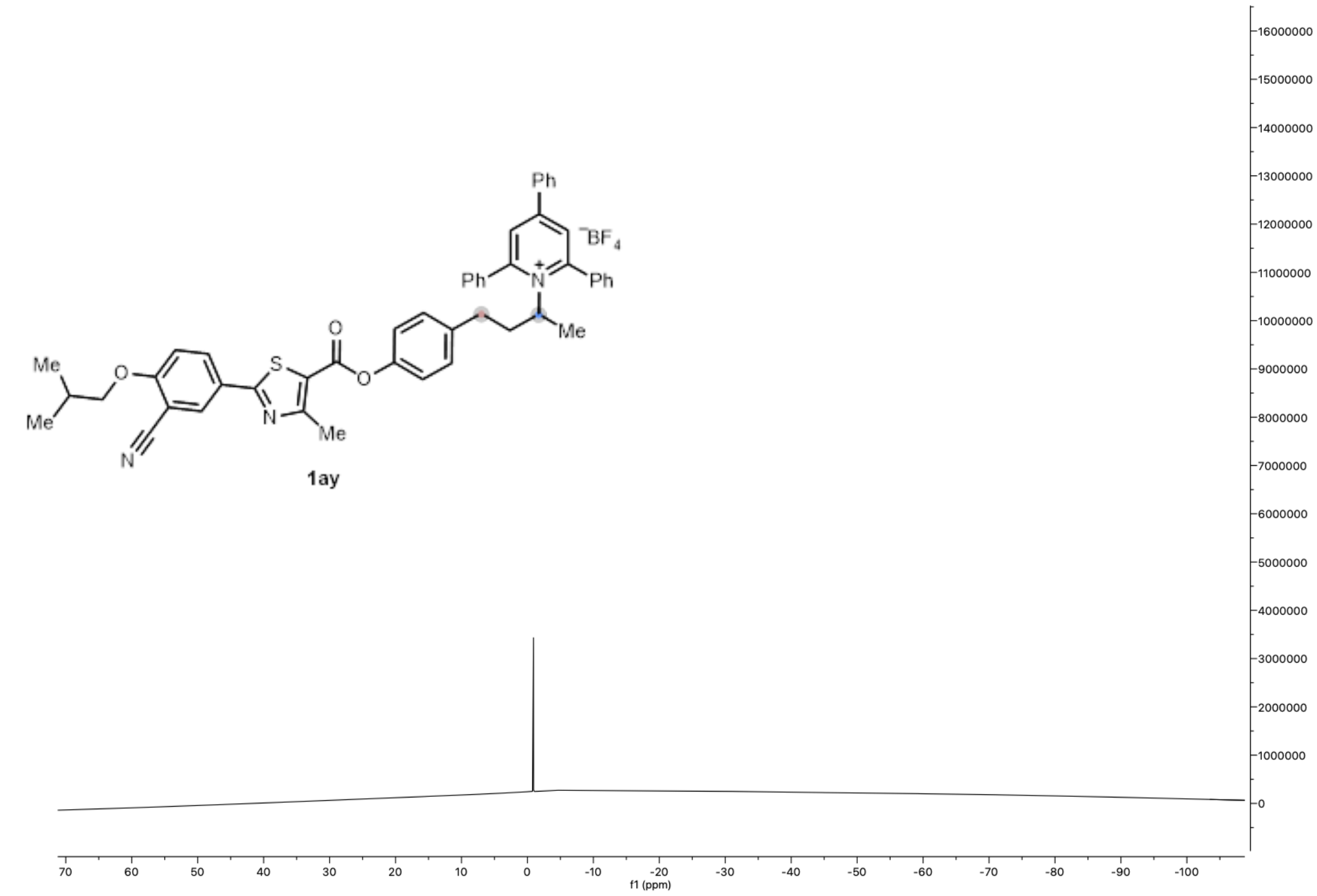

7-phenyl-14-(4-phenylbutyl)-5,6,8,9-tetrahydrodibenzo[*c*,*h*]acridin-14-ium tetrafluoroborate **Acridin1**:

^{1}H NMR (500 MHz, $CDCl_3$):

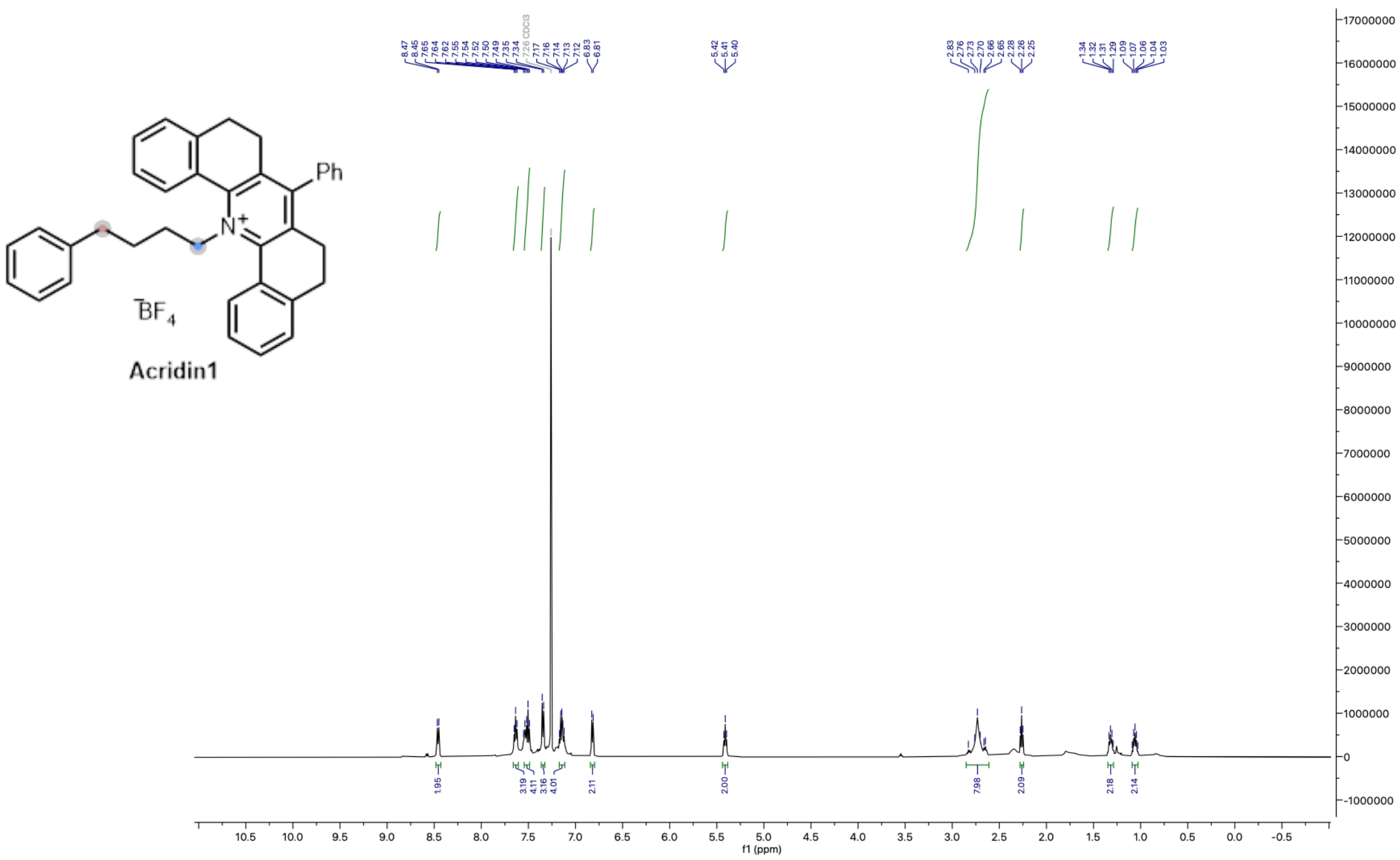

^{13}C NMR (126 MHz, $CDCl_3$):

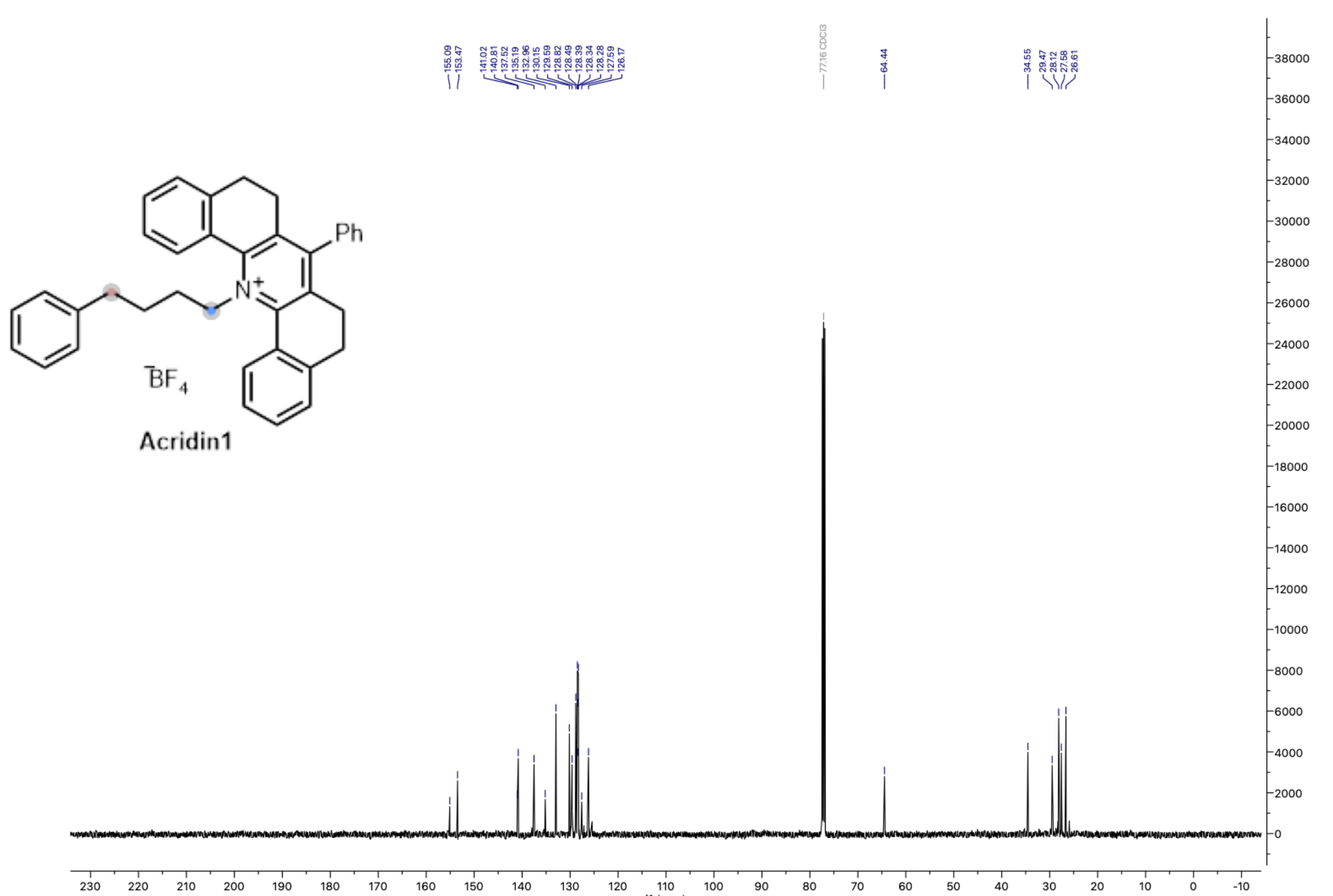

^{19}F NMR (282 MHz, $CDCl_3$):

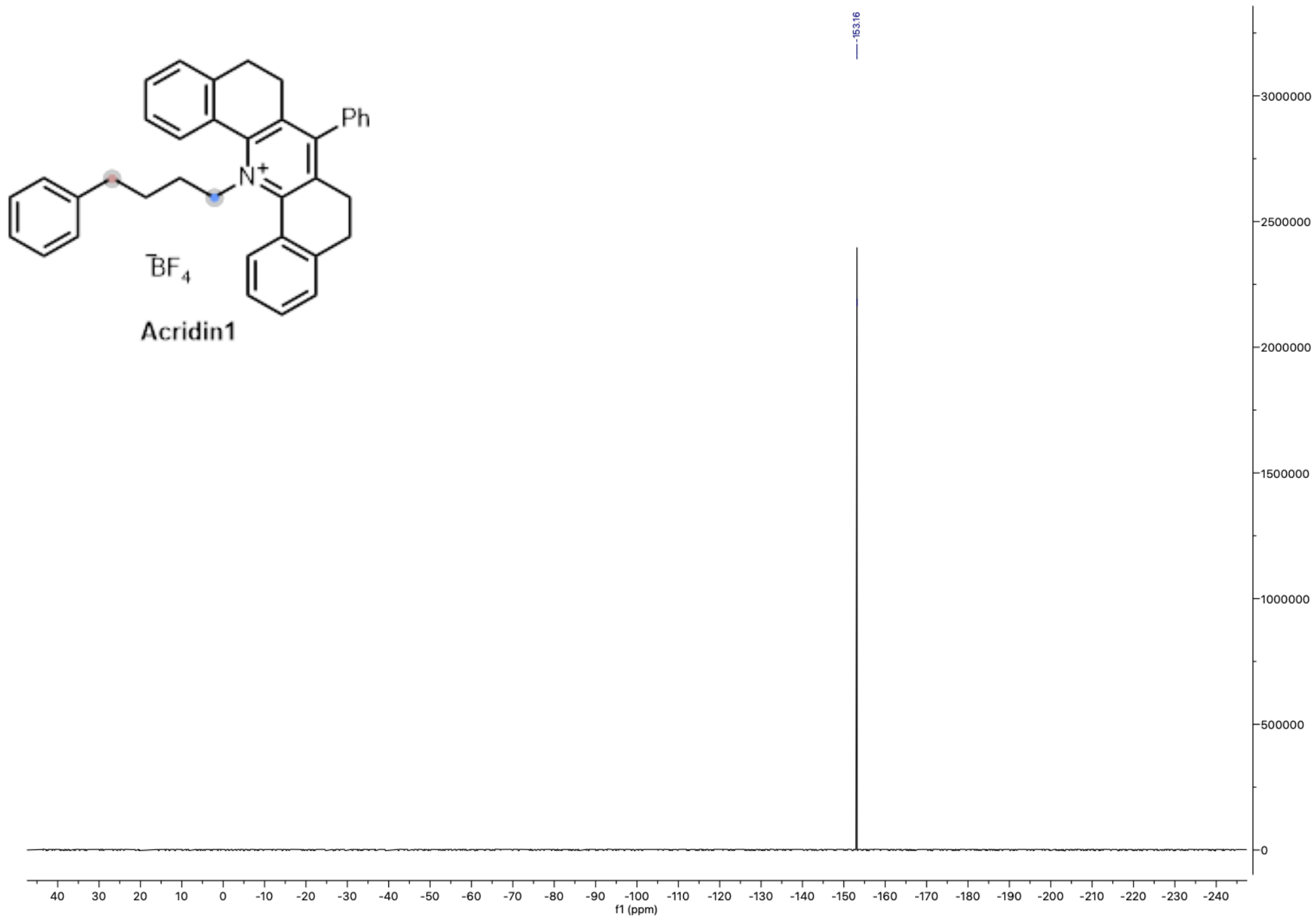

^{11}B NMR (96 MHz, $CDCl_3$):

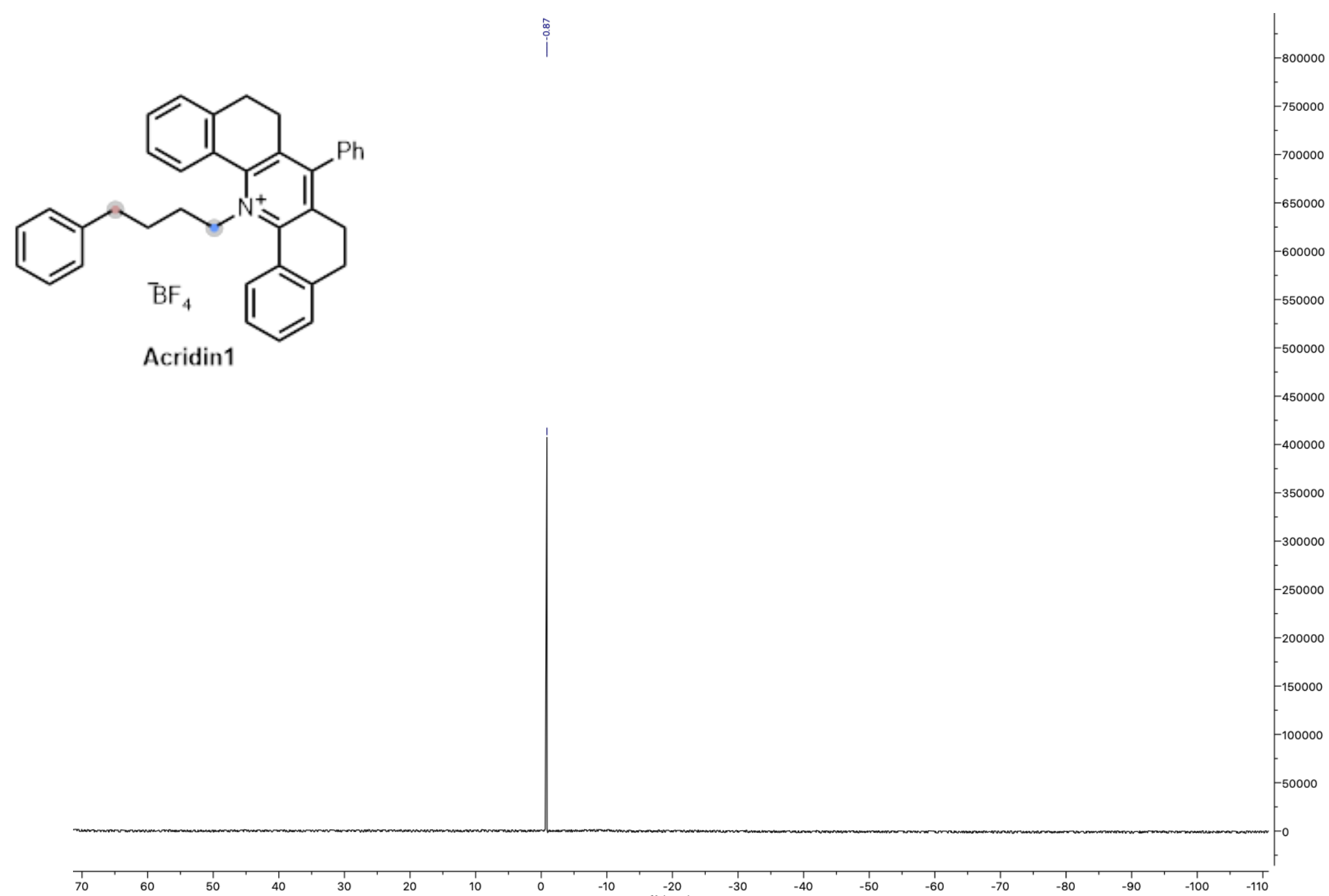

14-(2-(1-phenyl-1*H*-pyrazol-3-yl)ethyl)-7-(4-(trifluoromethyl)phenyl)-5,6,8,9-tetrahydrodibenzo[*c*,*h*]acridin-14-ium trifluoromethylsulfonate **Acridin3:**

^{1}H NMR (500 MHz, $CDCl_3$):

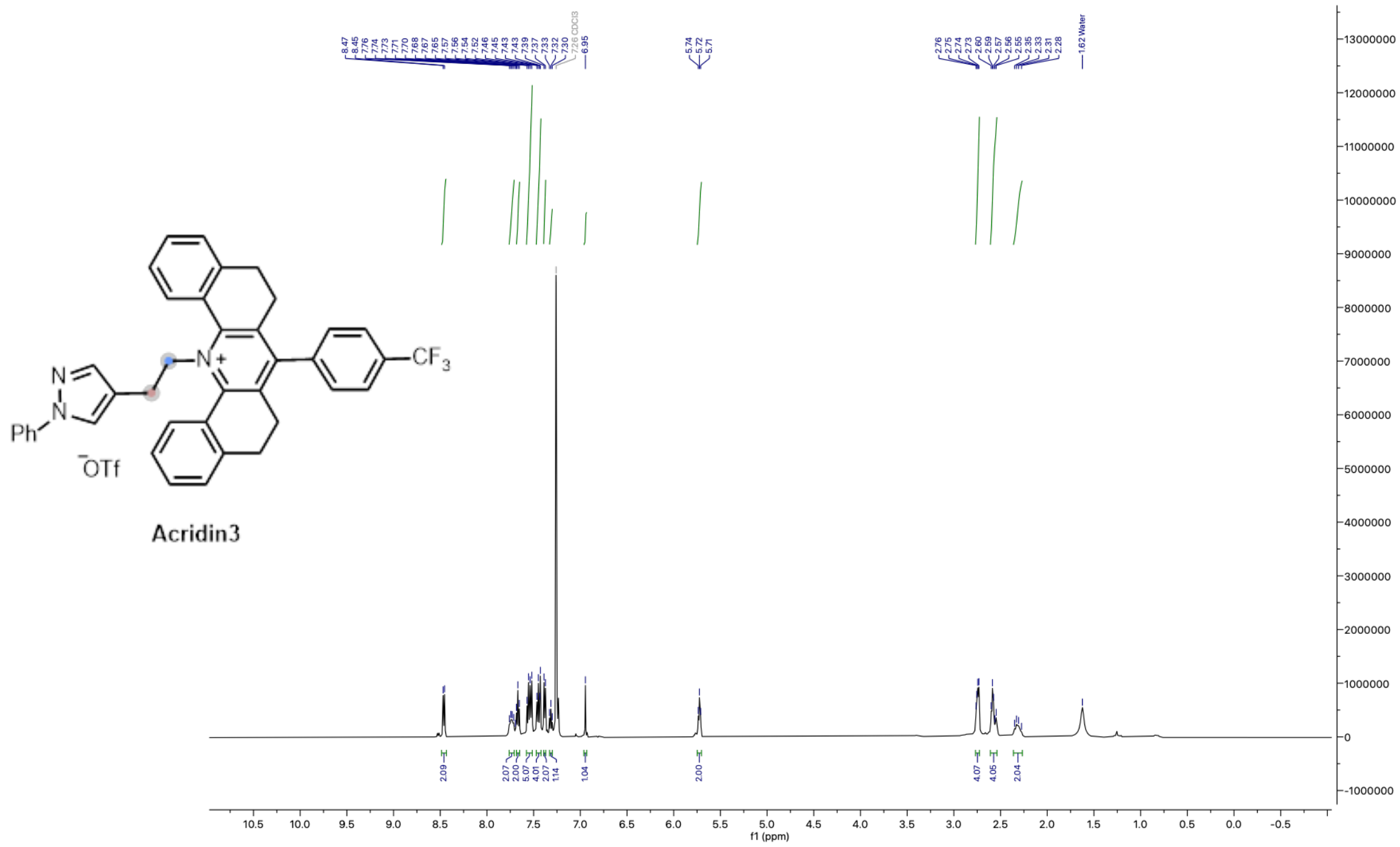

^{13}C NMR (126 MHz, $CDCl_3$):

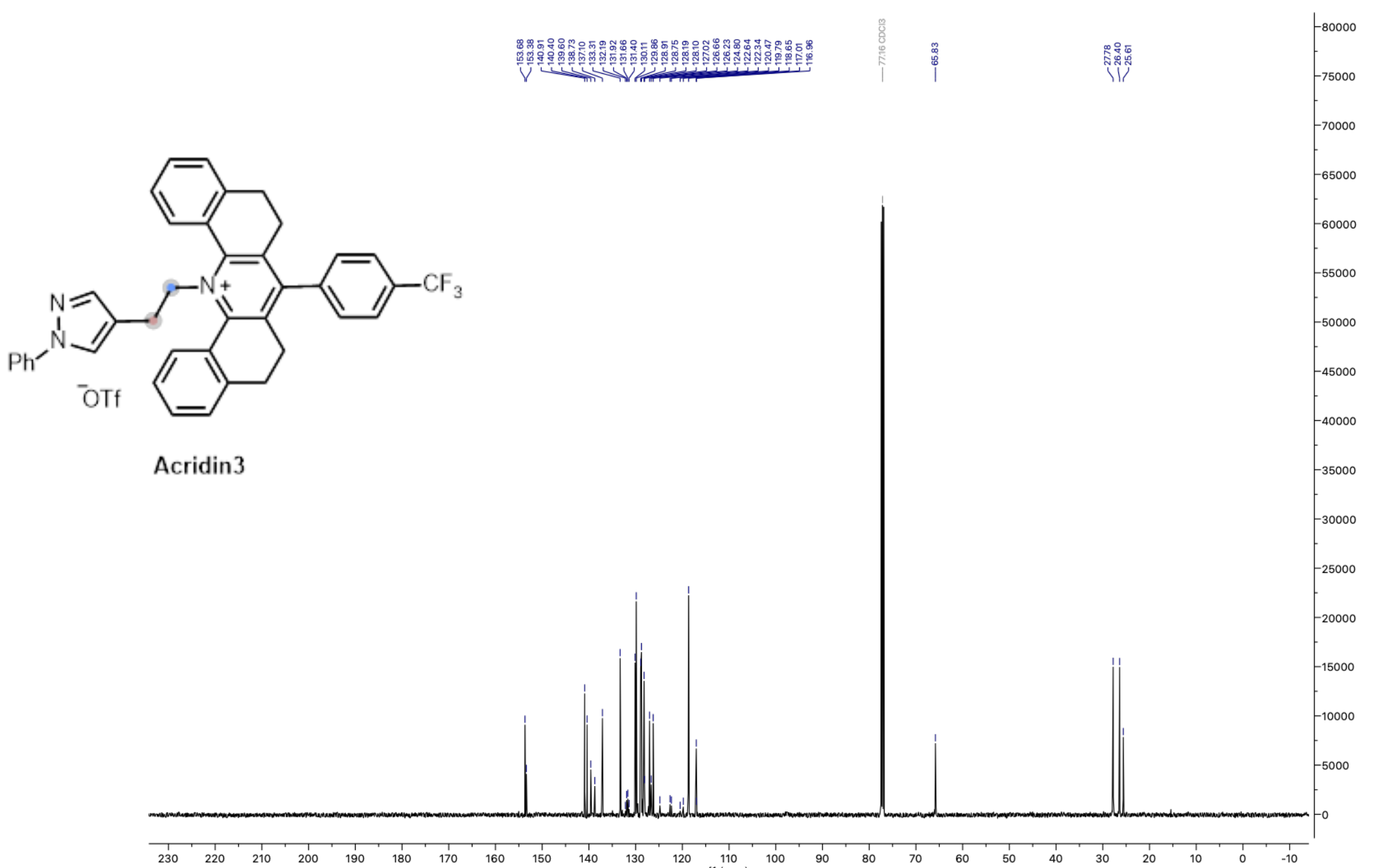

^{19}F NMR (282 MHz, $CDCl_3$):

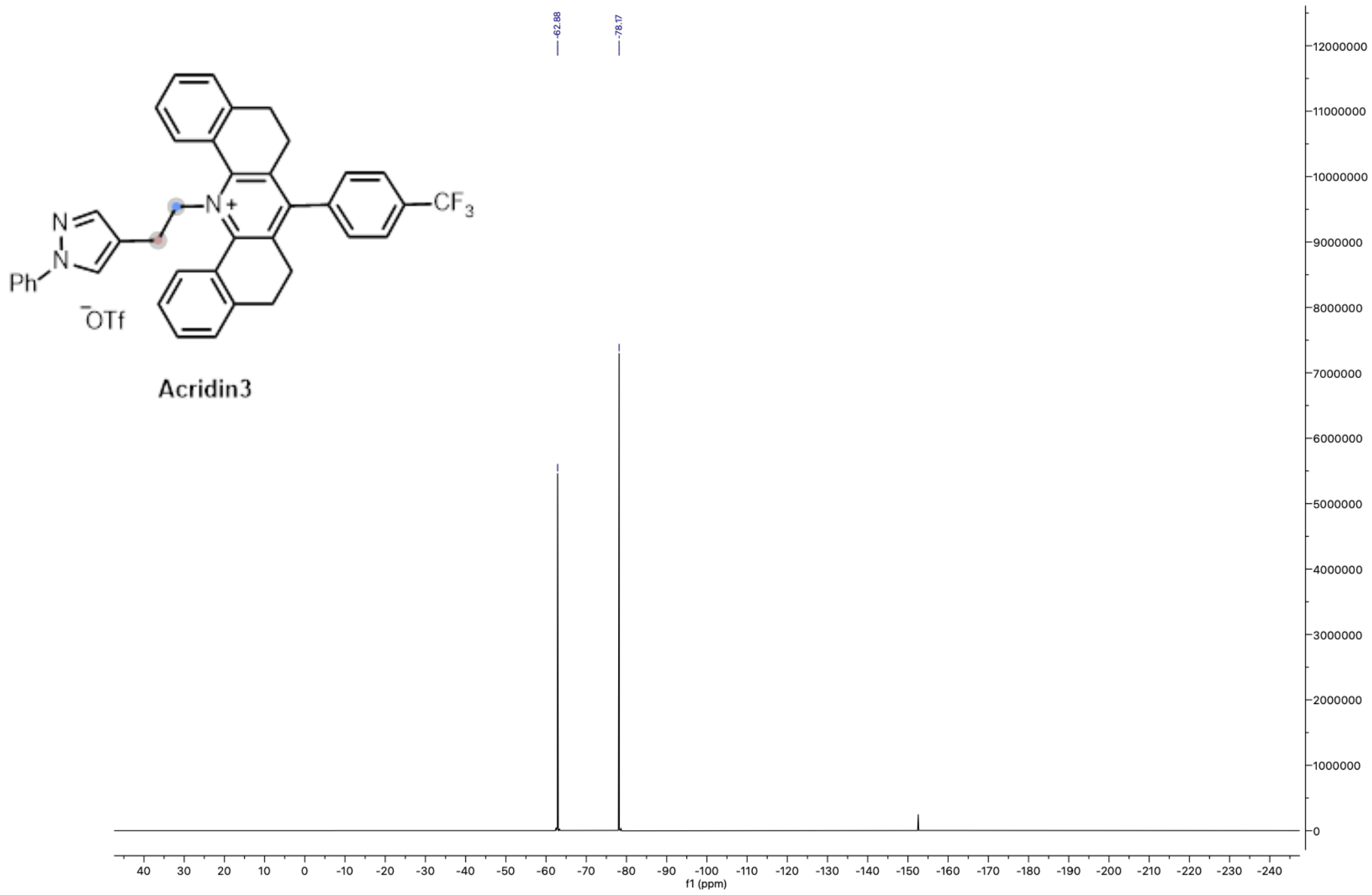

1-(4-hydroxy-3-methoxyphenethyl)-2,4,6-triphenylpyridin-1-ium trifluoromethanesulfonate **Acridin5**:

^{1}H NMR (500 MHz, $CDCl_3$):

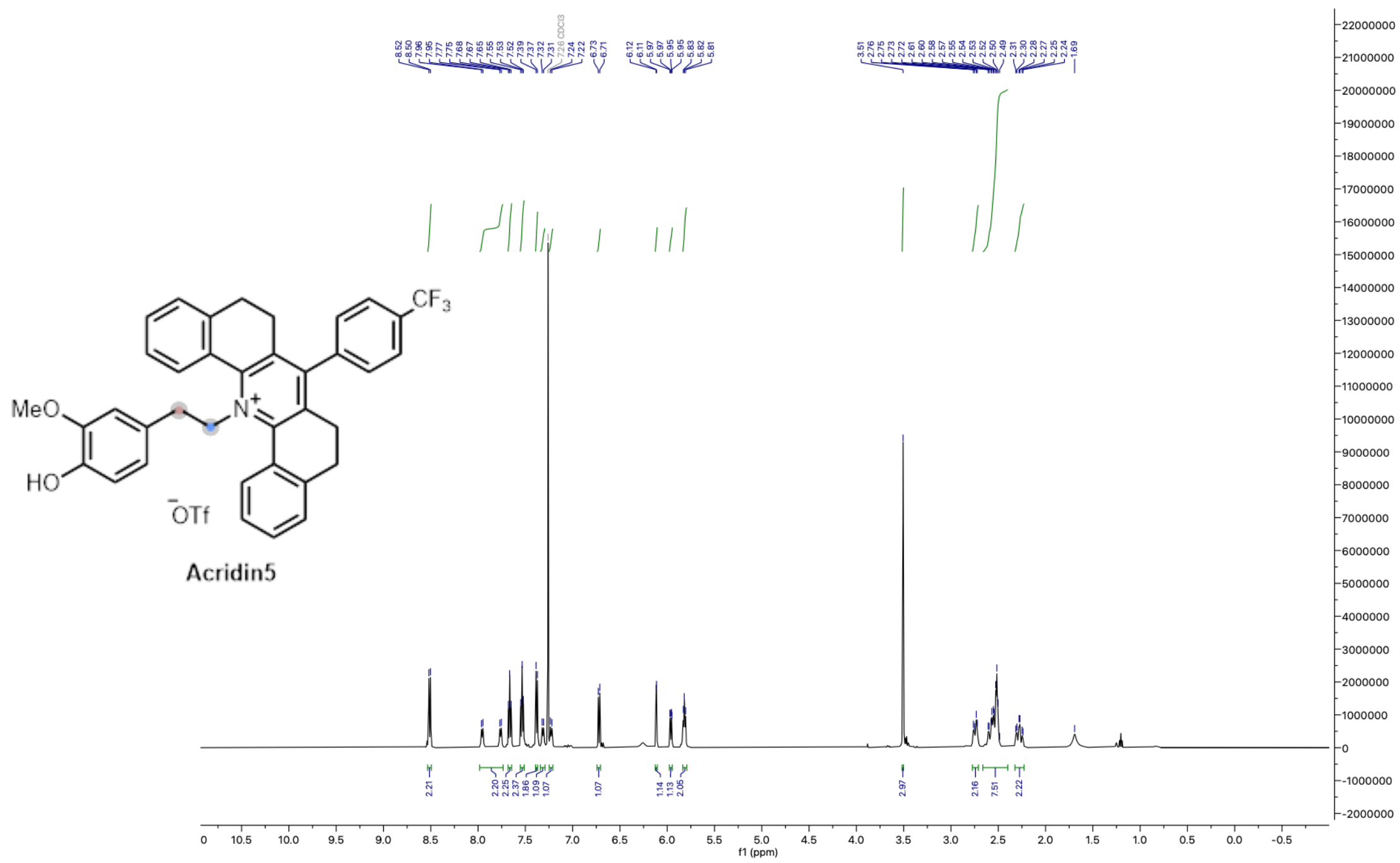

^{13}C NMR (126 MHz, $CDCl_3$):

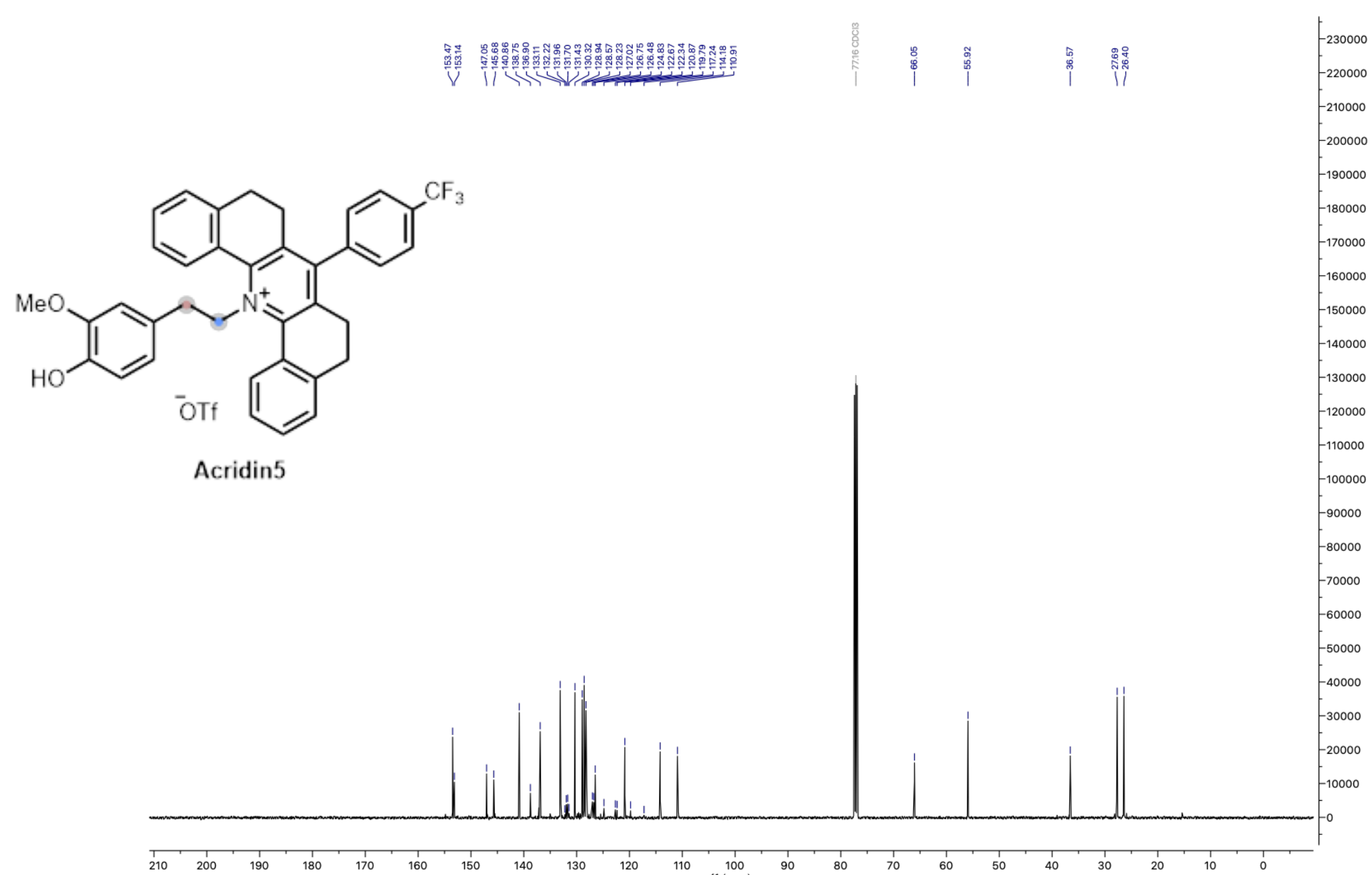

^{19}F NMR (282 MHz, $CDCl_3$):

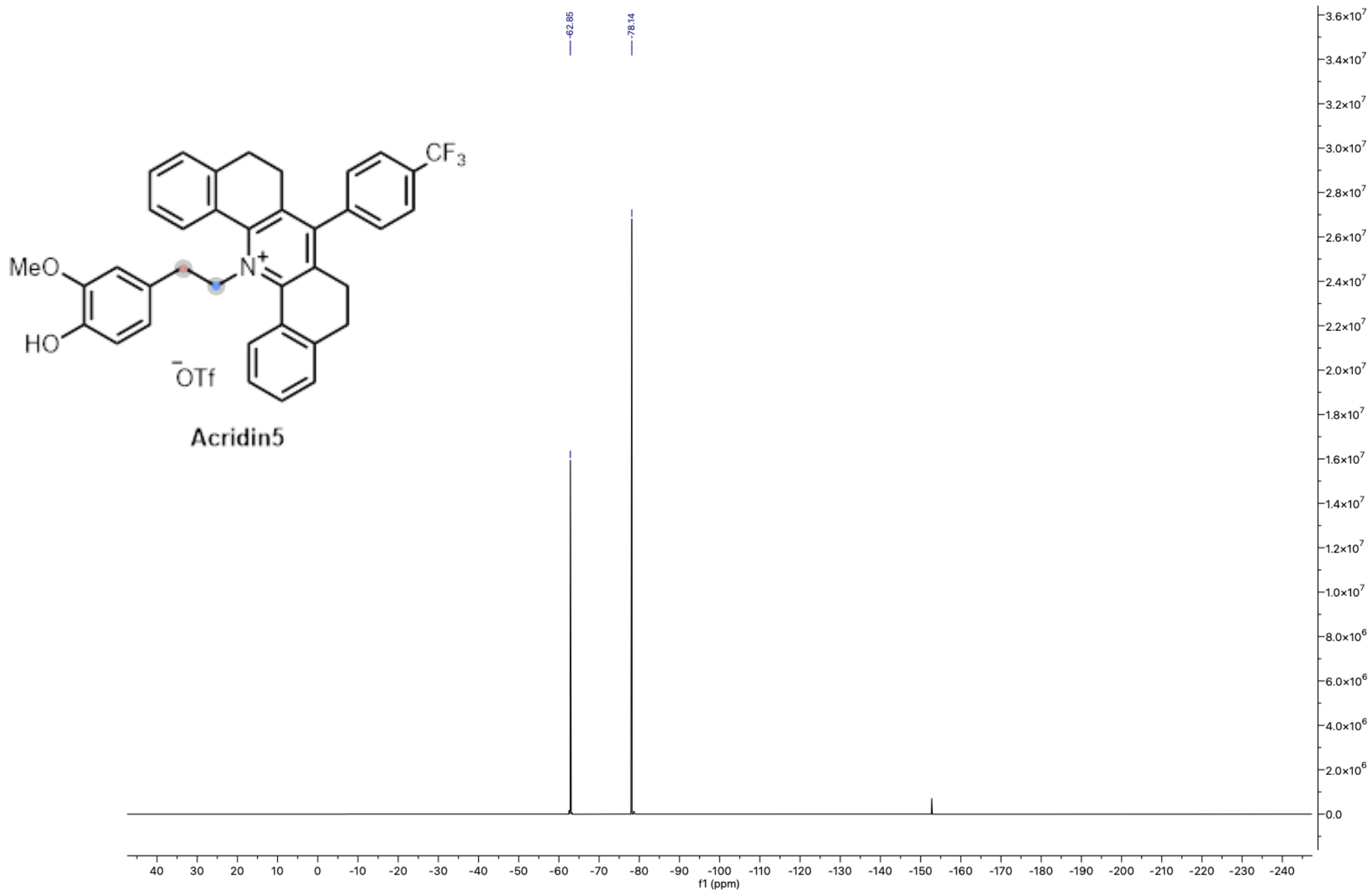

2,4,6-Triphenyl-1,4-bis(4-phenylbutan-2-yl)-1,4-dihydropyridine **6**:

^{1}H NMR (500 MHz, $CDCl_3$)

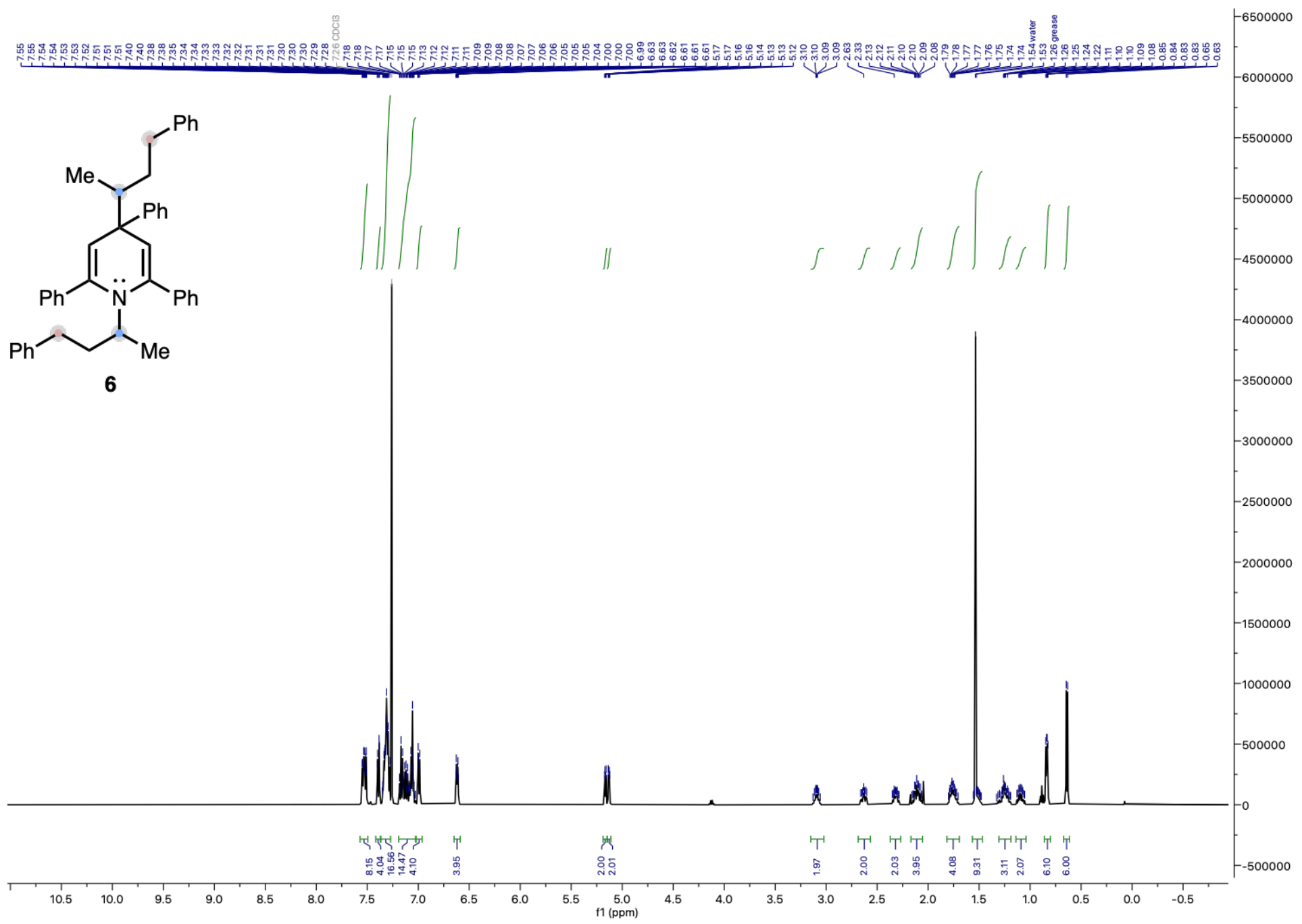

^{13}C NMR (126 MHz, $CDCl_3$):

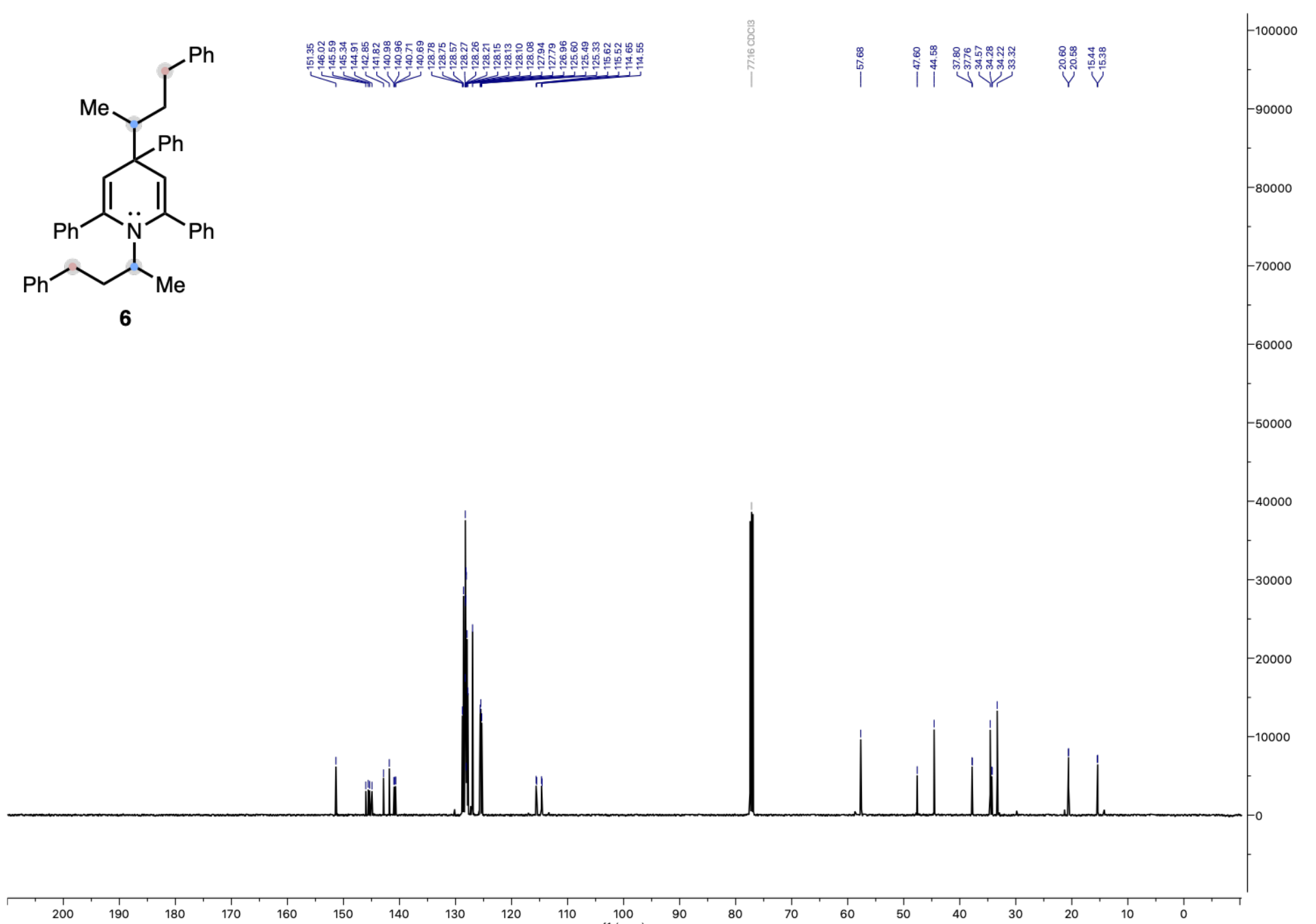

COSY:

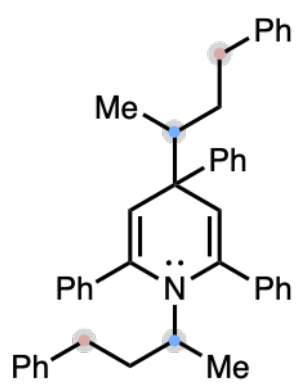

6

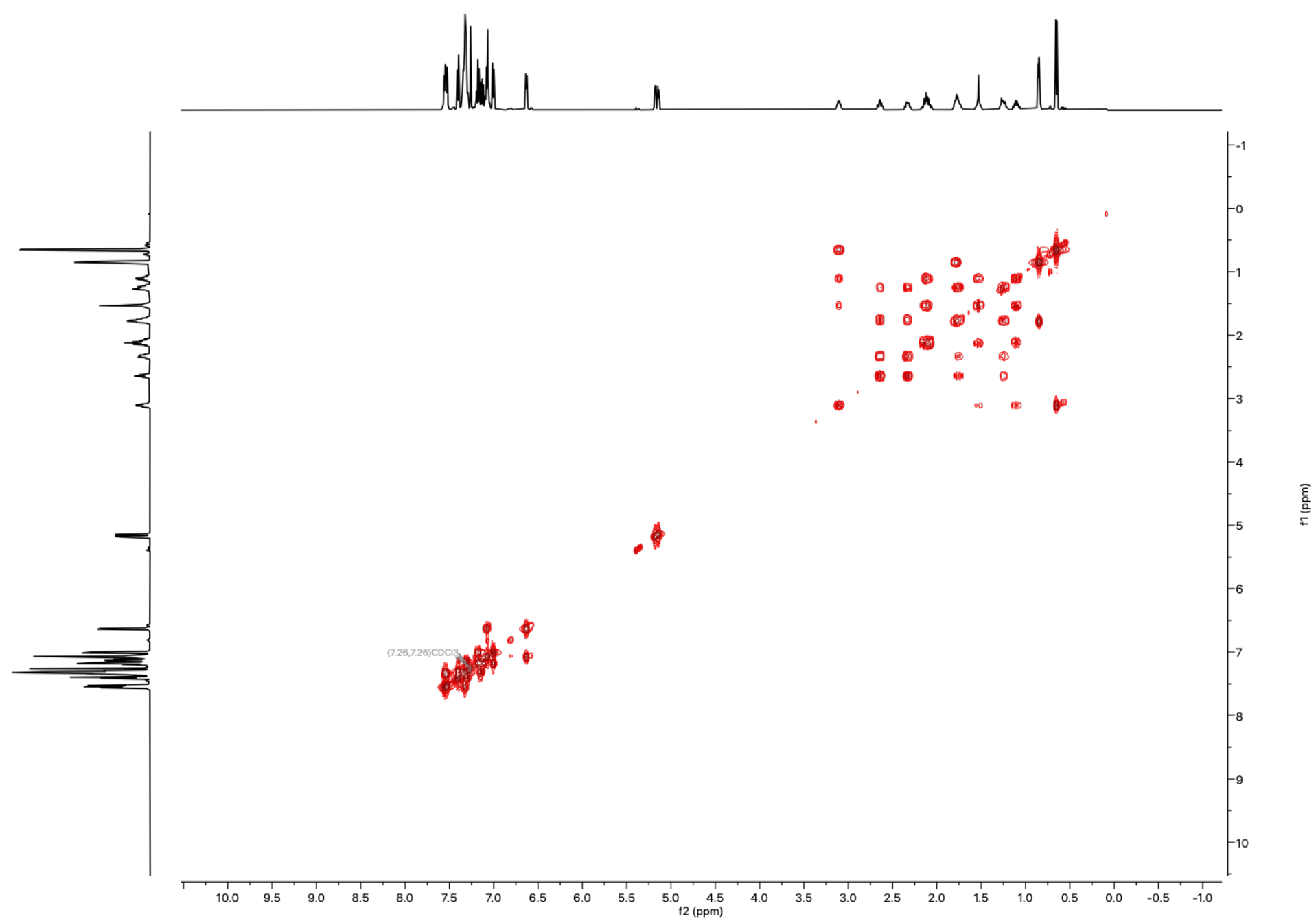

2,4,6-Triphenyl-1-(4-phenylbutan-2-yl)-4-(1-phenylbutyl)-1,4-dihydropyridine **7**:

^{1}H NMR (500 MHz, $CDCl_3$):

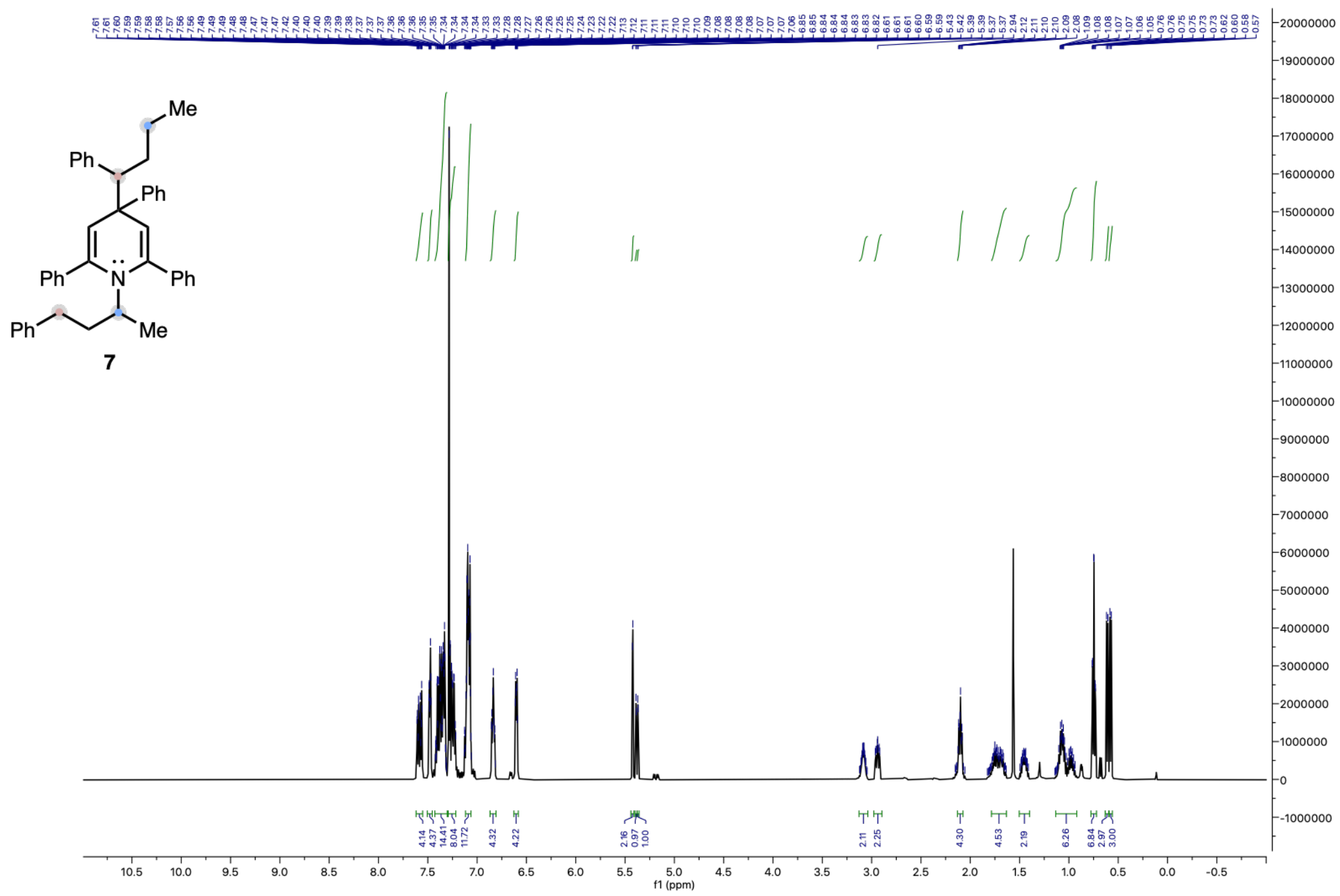

^{13}C NMR (126 MHz, $CDCl_3$):

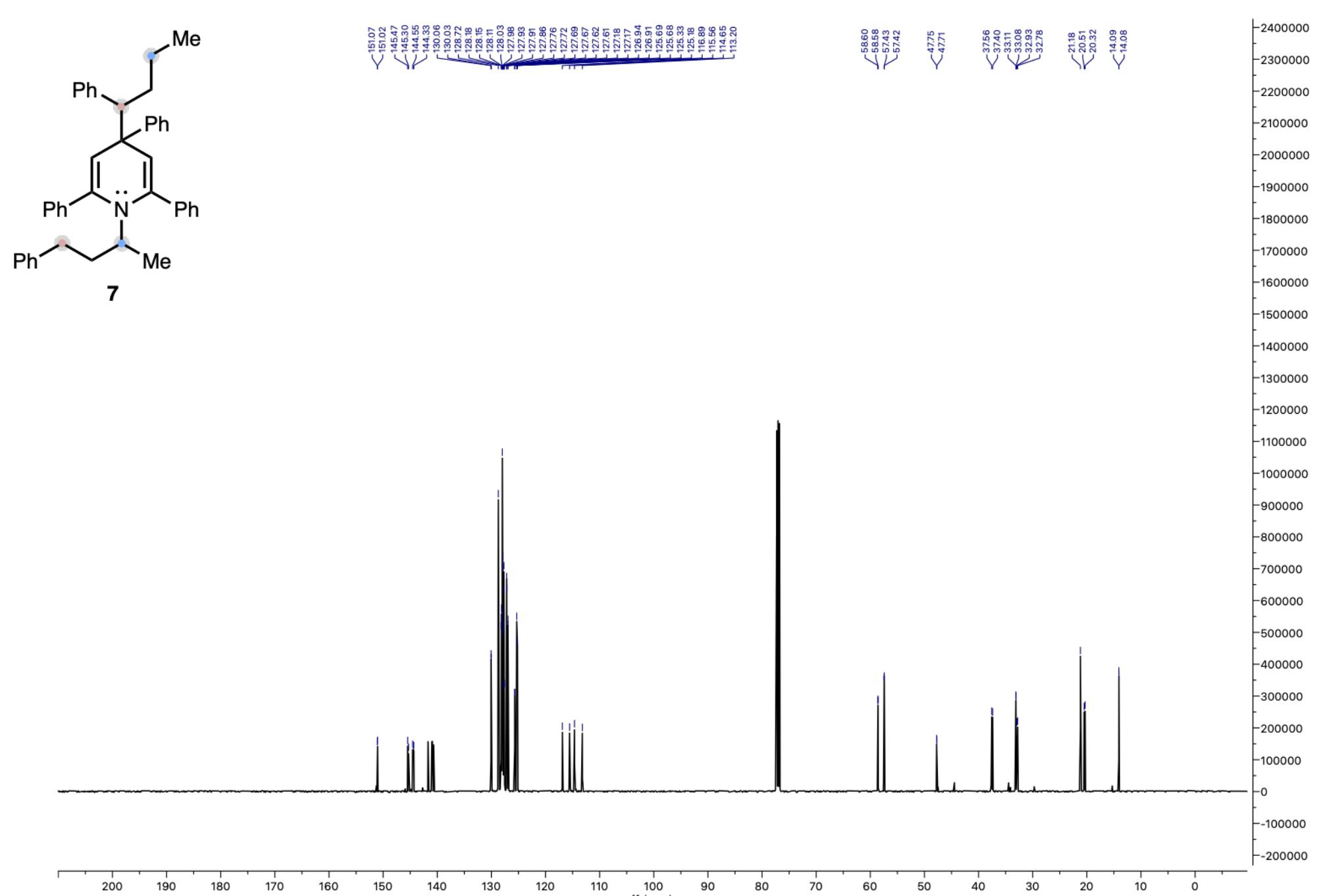

COSY:

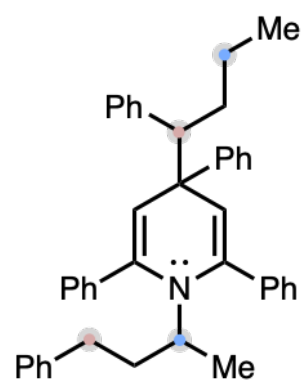

7

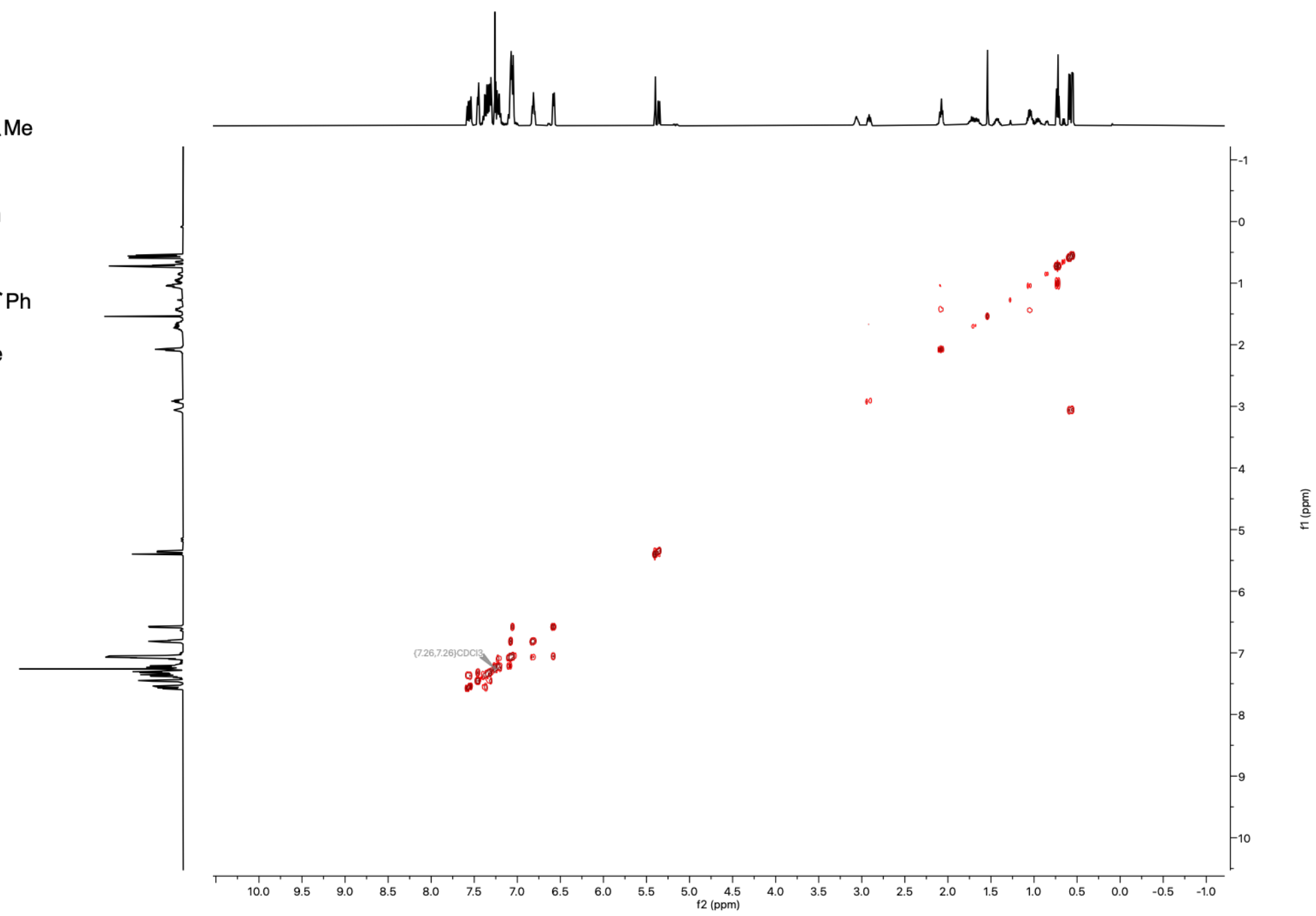

12. References

(1) Harris, R. K.; Becker, E. D.; De Menezes, S. M. C.; Goodfellow, R.; Granger, P. NMR Nomenclature: Nuclear Spin Properties and Conventions for Chemical Shifts - IUPAC Recommendations 2001. *Solid State Nucl. Magn. Reson.* **2002**, *22* (4), 458–483. https://doi.org/10.1006/snmr.2002.0063.

(2) Wu, J.; He, L.; Noble, A.; Aggarwal, V. K. Photoinduced Deaminative Borylation of Alkylamines. *J. Am. Chem. Soc.* **2018**, *140* (34), 10700–10704. https://doi.org/10.1021/jacs.8b07103.

(3) Zhao, F.; Li, C.-L.; Wu, X.-F. Deaminative Carbonylative Coupling of Alkylamines with Styrenes under Transition-Metal-Free Conditions. *Chemical Communications* **2020**, *56* (64), 9182–9185. https://doi.org/10.1039/D0CC04062B.

(4) Das, S.; Chakraborty, S.; Kumar, S.; Sharma, S.; Sahoo, B. Reductant-Controlled Access to Sulfinamides from Aliphatic Primary Amines. *Org. Lett.* **2025**, *27* (45), 12617–12622. https://doi.org/10.1021/acs.orglett.5c04062.

(5) Zhu, Z.-F.; Tu, J.-L.; Liu, F. Ni-Catalyzed Deaminative Hydroalkylation of Internal Alkynes. *Chemical Communications* **2019**, *55* (76), 11478–11481. https://doi.org/10.1039/C9CC05385A.

(6) Plunkett, S.; Basch, C. H.; Santana, S. O.; Watson, M. P. Harnessing Salts as Electrophiles in Deaminative Alkyl–Alkyl Cross-Couplings. *J. Am. Chem. Soc.* **2019**, *141* (6), 2257–2262. https://doi.org/10.1021/jacs.9b00111.

(7) Heijnen, D.; Tosi, F.; Vila, C.; Stuart, M. C. A.; Elsinga, P. H.; Szymanski, W.; Feringa, B. L. Oxygen Activated, Palladium Nanoparticle Catalyzed, Ultrafast Cross-Coupling of Organolithium Reagents. *Angew. Chem. Int. Ed.* **2017**, *56* (12), 3354–3359. https://doi.org/10.1002/anie.201700417.

(8) Bricelj, A.; Dora Ng, Y. L.; Ferber, D.; Kuchta, R.; Müller, S.; Monschke, M.; Wagner, K. G.; Krönke, J.; Sosič, I.; Gütschow, M.; Steinebach, C. Influence of Linker Attachment Points on the Stability and Neosubstrate Degradation of Cereblon Ligands. *ACS Med. Chem. Lett.* **2021**, *12* (11), 1733–1738. https://doi.org/10.1021/acsmedchemlett.1c00368.

(9) Petzold, D.; König, B. Photocatalytic Oxidative Bromination of Electron-Rich Arenes and Heteroarenes by Anthraquinone. *Adv. Synth. Catal.* **2018**, *360* (4), 626–630. https://doi.org/10.1002/adsc.201701276.

(10) Aguirre-Rentería, S. A.; Carrizales-Castillo, J. J. J.; del Rayo Camacho Corona, M.; Hernández-Fernández, E.; Garza-González, E.; Rivas-Galindo, V. M.; Arredondo-

Espinoza, E.; Avalos-Alanís, F. G. Synthesis and in Vitro Evaluation of Antimycobacterial and Cytotoxic Activity of New α,β-Unsaturated Amide, Oxazoline and Oxazole Derivatives from l-Serine. *Bioorg. Med. Chem. Lett.* **2020**, *30* (9), 127074. https://doi.org/10.1016/J.BMCL.2020.127074.

(11) Duan, H.; Meng, L.; Bao, D.; Zhang, H.; Li, Y.; Lei, A. Zinc Chloride Enhanced Arylations of Secondary Benzyl Trifluoroacetates in the Presence of Β-Hydrogen Atoms. *Angew. Chem. Int. Ed.* **2010**, *49* (36), 6387–6390. https://doi.org/10.1002/anie.201002116.

(12) He, Y.; Cai, Y.; Zhu, S. Mild and Regioselective Benzylic C–H Functionalization: Ni-Catalyzed Reductive Arylation of Remote and Proximal Olefins. *J. Am. Chem. Soc.* **2017**, *139* (3), 1061–1064. https://doi.org/10.1021/jacs.6b11962.

(13) He, Y.; Liu, C.; Yu, L.; Zhu, S. Ligand-Enabled Nickel-Catalyzed Redox-Relay Migratory Hydroarylation of Alkenes with Arylborons. *Angew. Chem. Int. Ed.* **2020**, *59* (23), 9186–9191. https://doi.org/10.1002/anie.202001742.

(14) Xu, C.; A, R.; Wu, X. Remote Migratory Reductive Arylation of Unactivated Alkenes Enabled by Electrochemical Nickel Catalysis. *ChemSusChem* **2025**, *18* (6), e202402196. https://doi.org/10.1002/cssc.202402196.

(15) Peng, L.; Li, Z.; Yin, G. Photochemical Nickel-Catalyzed Reductive Migratory Cross-Coupling of Alkyl Bromides with Aryl Bromides. *Org. Lett.* **2018**, *20* (7), 1880–1883. https://doi.org/10.1021/acs.orglett.8b00413.

(16) Liu, J.; Gong, H.; Zhu, S. $BH_3 \cdot Me_2S$: An Alternative Hydride Source for NiH-Catalyzed Reductive Migratory Hydroarylation and Hydroalkenylation of Alkenes. *European J. Org. Chem.* **2021**, *2021* (10), 1543–1546. https://doi.org/10.1002/ejoc.202100005.

(17) Chen, F.; Chen, K.; Zhang, Y.; He, Y.; Wang, Y.-M.; Zhu, S. Remote Migratory Cross-Electrophile Coupling and Olefin Hydroarylation Reactions Enabled by in Situ Generation of NiH. *J. Am. Chem. Soc.* **2017**, *139* (39), 13929–13935. https://doi.org/10.1021/jacs.7b08064.

(18) Wang, D.-M.; She, L.-Q.; Wu, Y.; Zhu, C.; Wang, P. Ligand-Enabled Ni-Catalyzed Hydroarylation and Hydroalkenylation of Internal Alkenes with Organoborons. *Nat. Commun.* **2022**, *13* (1), 6878. https://doi.org/10.1038/s41467-022-34675-x.

(19) Clausen, F.; Kischkewitz, M.; Bergander, K.; Studer, A. Catalytic Protodeboronation of Pinacol Boronic Esters: Formal Anti-Markovnikov

Hydromethylation of Alkenes. *Chem. Sci.* **2019**, *10* (24), 6210–6214. https://doi.org/10.1039/C9SC02067E.

(20) Léonard, N. G.; Chirik, P. J. Air-Stable α-Diimine Nickel Precatalysts for the Hydrogenation of Hindered, Unactivated Alkenes. *ACS Catal.* **2018**, *8* (1), 342–348. https://doi.org/10.1021/acscatal.7b03909.

(21) Li, J.; Chen, J.; Sang, R.; Ham, W. S.; Plutschack, M. B.; Berger, F.; Chabbra, S.; Schnegg, A.; Genicot, C.; Ritter, T. Photoredox Catalysis with Aryl Sulfonium Salts Enables Site-Selective Late-Stage Fluorination. *Nature Chemistry 2019 12:1* **2019**, *12* (1), 56–62. https://doi.org/10.1038/s41557-019-0353-3.

(22) Frisch, M. J.; Trucks, G. W.; Schlegel, H. B.; Scuseria, G. E.; Robb, M. A.; Cheeseman, J. R.; Scalmani, G.; Barone, V.; Petersson, G. A.; Nakatsuji, H.; Li, X.; Caricato, M.; Marenich, A. V.; Bloino, J.; Janesko, B. G.; Gomperts, R.; Mennucci, B.; Hratchian, H. P.; Ortiz, J. V.; Izmaylov, A. F.; Sonnenberg, J. L.; Williams-Young, D.; Ding, F.; Lipparini, F.; Egidi, F.; Goings, J.; Peng, B.; Petrone, A.; Henderson, T.; Ranasinghe, D.; Zakrzewski, V. G.; Gao, J.; Rega, N.; Zheng, G.; Liang, W.; Hada, M.; Ehara, M.; Toyota, K.; Fukuda, R.; Hasegawa, J.; Ishida, M.; Nakajima, T.; Honda, Y.; Kitao, O.; Nakai, H.; Vreven, T.; Throssell, K.; Montgomery, J. A., Jr.; Peralta, J. E.; Ogliaro, F.; Bearpark, M. J.; Heyd, J. J.; Brothers, E. N.; Kudin, K. N.; Staroverov, V. N.; Keith, T. A.; Kobayashi, R.; Normand, J.; Raghavachari, K.; Rendell, A. P.; Burant, J. C.; Iyengar, S. S.; Tomasi, J.; Cossi, M.; Millam, J. M.; Klene, M.; Adamo, C.; Cammi, R.; Ochterski, J. W.; Martin, R. L.; Morokuma, K.; Farkas, O.; Foresman, J. B.; Fox, D. J. Gaussian, Inc., Wallingford CT, 2016.

(23) Zhao, Y.; Truhlar, D. G. A New Local Density Functional for Main-Group Thermochemistry, Transition Metal Bonding, Thermochemical Kinetics, and Noncovalent Interactions. *Journal of Chemical Physics* **2006**, *125* (19). https://doi.org/10.1063/1.2370993/566338.

(24) Weigend, F.; Ahlrichs, R. Balanced Basis Sets of Split Valence, Triple Zeta Valence and Quadruple Zeta Valence Quality for H to Rn: Design and Assessment of Accuracy. *Physical Chemistry Chemical Physics* **2005**, *7* (18), 3297–3305. https://doi.org/10.1039/B508541A.

(25) Barone, V.; Cossi, M. Quantum Calculation of Molecular Energies and Energy Gradients in Solution by a Conductor Solvent Model. *J. Phys. Chem. A* **1998**, *102* (11), 1995–2001. https://doi.org/10.1021/jp9716997.

(26) Cossi, M.; Rega, N.; Scalmani, G.; Barone, V. Energies, Structures, and Electronic Properties of Molecules in Solution with the C-PCM Solvation Model. *J. Comput. Chem.* **2003**, *24* (6), 669–681. https://doi.org/10.1002/jcc.10189.

(27) Takano, Y.; Houk, K. N. Benchmarking the Conductor-like Polarizable Continuum Model (CPCM) for Aqueous Solvation Free Energies of Neutral and Ionic Organic Molecules. *J. Chem. Theory Comput.* **2005**, *1* (1), 70–77. https://doi.org/10.1021/ct049977a.

(28) Saper, N. I.; Ohgi, A.; Small, D. W.; Semba, K.; Nakao, Y.; Hartwig, J. F. Nickel-Catalysed Anti-Markovnikov Hydroarylation of Unactivated Alkenes with Unactivated Arenes Facilitated by Non-Covalent Interactions. *Nat. Chem.* **2020**, *12* (3), 276–283. https://doi.org/10.1038/S41557-019-0409-4;SUBJMETA.

(29) Slinker, J. D.; Gorodetsky, A. A.; Lowry, M. S.; Wang, J.; Parker, S.; Rohl, R.; Bernhard, S.; Malliaras, G. G. Efficient Yellow Electroluminescence from a Single Layer of a Cyclometalated Iridium Complex. *J. Am. Chem. Soc.* **2004**, *126* (9), 2763–2767. https://doi.org/10.1021/ja0345221.

(30) Day, C. S.; Rentería-Gómez, Á.; Ton, S. J.; Gogoi, A. R.; Gutierrez, O.; Martin, R. Elucidating Electron-Transfer Events in Polypyridine Nickel Complexes for Reductive Coupling Reactions. *Nat. Catal.* **2023**, *6* (3), 244–253. https://doi.org/10.1038/s41929-023-00925-4.
